# MACROD2 deficiency promotes hepatocellular carcinoma growth and metastasis by activating GSK-3β/β-catenin signaling

**DOI:** 10.1038/s41525-020-0122-7

**Published:** 2020-04-01

**Authors:** Zheng-Jun Zhou, Chu-Bin Luo, Hao-Yang Xin, Zhi-Qiang Hu, Gui-Qi Zhu, Jia Li, Shao-Lai Zhou

**Affiliations:** 1 Liver Surgery Department, Liver Cancer Institute, Zhongshan Hospital, Fudan University, 200032 Shanghai, China; 2 Key Laboratory of Carcinogenesis and Cancer Invasion (Fudan University), Ministry of Education, 200032 Shanghai, China

**Keywords:** Cancer genomics, Metastasis, Gastrointestinal cancer

## Abstract

Structural variations (SVs) influence the development and progression of multiple types of cancer. The genes affected by SVs in hepatocellular carcinoma (HCC) and their contribution to tumor growth and metastasis remain unknown. In this study, through whole-genome sequencing (WGS), we identified *MACROD2* as the gene most frequently affected by SVs, which were associated with low MACROD2 expression levels. Low MACROD2 expression was predictive of tumor recurrence and poor overall survival. MACROD2 expression was decreased in HCC cell lines, especially those with high metastatic potential. MACROD2 knockdown in HCC cells markedly enhanced proliferation and invasiveness in vitro and tumor progression in vivo and promoted epithelial–mesenchymal transition (EMT). By contrast, MACROD2 overexpression reversed EMT and inhibited HCC growth and metastasis. Mechanistically, MACROD2 deficiency suppressed glycogen synthase kinase-3β (GSK-3β) activity and activated β-catenin signaling, which mediated the effect of MACROD2 on HCC. In clinical HCC samples, decreased MACROD2 expression was correlated with the activation of GSK-3β/β-catenin signaling and the EMT phenotype. Overall, our results revealed that MACROD2 is frequently affected by SVs in HCC, and its deficiency promotes tumor growth and metastasis by activating GSK-3β/β-catenin signaling.

## Introduction

Hepatocellular carcinoma (HCC) is one of the most common cancers worldwide, and its incidence and mortality rate are increasing^1–3^. Curative treatments are available for HCC when the disease is diagnosed in the early stages, but most patients receive a diagnosis only after the disease has progressed to a more advanced stage^4,5^. High rates of recurrence and metastasis limit the long-term survival of patients with HCC despite advances in surgical treatments and patient management that have led to some improvements in patient outcomes^6–8^. Therefore, it is critical to gain an understanding of the molecular mechanisms underlying HCC growth and metastasis so that new treatments can be developed to improve long-term survival rates among patients with HCC.

Over the past decade, new discoveries have shed light on the molecular basis of HCC pathogenesis. Following technological advances, several pioneering studies have delineated the genetic landscape underlying liver carcinogenesis^9–15^, including amplifications on chromosomes 6p21 (*VEGFA*) and 11q13 (*FGF19*/*CNND1*) and deletions on chromosome 9 (*CDKN2A*). Mutations in the coding regions of *TP53* and *CTNNB1* affect 25–30% of patients with HCC and, along with low-frequency mutations in some other genes (e.g., *AXIN1*, *ARID2*, *ARID1A*, *TSC1*/*TSC2*), define core pathways that are commonly de-regulated in HCC. However, it is largely unknown what roles genes affected by structural variations (SVs) play in HCC.

In the present study, we performed whole-genome sequencing (WGS) of pairs of matched tumor and normal tissues from 49 Chinese patients with HCC. We assessed MACROD2 expression in those 49 HCCs and in an additional independent set of 380 formalin-fixed paraffin-embedded (FFPE) samples and analyzed its association with patient outcomes. Then, we explored its biological effect and mechanism of action on HCC growth and metastasis. Finally, we validated our results using clinical HCC samples.

## Results

### MACROD2 SVs are frequent in HCC and are associated with low MACROD2 expression levels

**Fig. 1:**
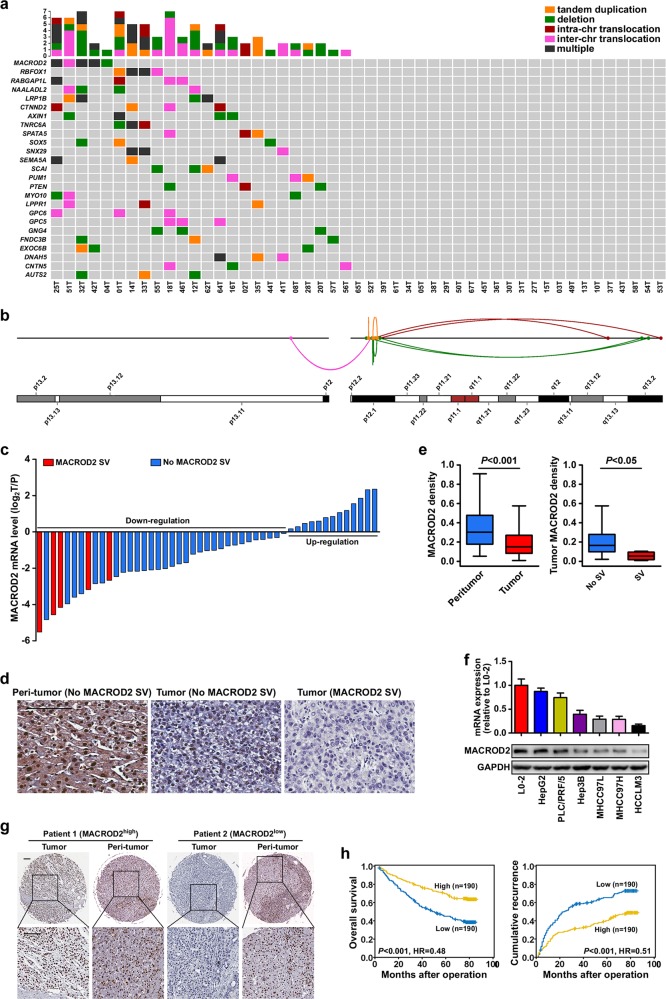
*MACROD2* is frequently affected by SVs in HCC, and down-regulation of MACROD2 correlates with patient poor prognosis. **a** The somatic SV spectrum in 49 HCCs identified by WGS. Genes containing SVs in at least three samples are shown. **b** Different types of SVs affecting MACROD2 are indicated by lines with different colors (also exhibited in Table 1). **c** *MACROD2* mRNA expression in 49 HCC tumor tissues compared with that in adjacent non-tumor tissues. **d** Representative MACROD2 staining in peritumor tissues and tumor tissues with no MACROD2 SV and with MACROD2 SV (25T). Scale bars = 100 μm. **e** The statistics of the MACROD2 staining density among different groups in 49 HCCs involved in WGS. **f** MACROD2 expression examined by qRT-PCR and western blot in one normal liver cell line (L0-2) and six HCC cell lines. **g** Representative HCC tumor and peritumor samples in the FFPE cohort showing the expression of MACROD2: patient 1, high MACROD2 expression; patient 2, low MACROD2 expression. Scale bar = 100 μm. **h** Kaplan–Meier survival analysis showing survival rates and cumulative recurrence rates on the basis of MACROD2 expression in the FFPE cohort. Data are shown as the mean ± standard deviation (SD) and are representative of three independent experiments.

We performed WGS of tumor and matched non-cancerous liver tissue samples from 49 patients with HCC. The average sequencing depth was 54.5-fold for tumors and 36.1-fold for normal tissues (Supplementary Table 4). We mapped the sequence reads to the human reference genome and detected a mean of 43.4 somatic SVs (range = 0–224) per sample among the 49 HCCs subjected to WGS. The SVs comprised 690 deletions, 414 tandem duplications, 3 insertions, 615 inter-chromosomal translocations, and 404 intra-chromosomal translocations (Supplementary Fig. 1 and Supplementary Table 5). *MACROD2*, *RBFOX1*, and *LRP1B* were the genes most frequently affected by SVs. *MACROD2* SVs occurred in 10.2% (5/49) of the patients; four patients harbored *MACROD2* deletions, and three patients harbored multiple types of SVs (Fig. 1a, b and Table 1).

**Table 1:** Different types of SVs affecting MACROD2 in 49 HCCs involved in WGS.

Sample	Chrom1	Start1	Chrom2	End2	Gene	Event	Labeled in Fig. 1B
04T	20	14,768,954	20	14,769,707	*MACROD2*	del_4518	Y
25T	20	14,017,359	20	14,021,879	*MACROD2*	del_insod_100_0/2438_0(del)	Y
25T	20	46,872,864	20	50,658,584	.	del_insod_100_0/2438_0(insod)	N
25T	20	13,981,153	20	14,831,335	*MACROD2*	del_insou_682_0/322_0(insou)	N
25T	20	48,288,758	20	48,293,998	start1: *B4GALT5*; end2: *B4GALT5*	del_insou_682_0/322_0(del)	N
25T	20	15,865,728	20	51,680,077	start1: *MACROD2*; end2: *TSHZ2*	del_715_0	Y
25T	20	1,094,848	20	2,470,268	start1: *PSMF1*; end2: *ZNF343*	del_inssd_71_0/2634_0(del)	N
25T	20	5,971,327	20	15,265,000	start1: *MCM8*; end2: *MACROD2*	del_inssd_71_0/2634_0(inssd)	N
25T	20	11,890,413	20	13,592,149	start1: *BTBD3*; end2: *TASP1*	del_inssd_169_0/884_0(del)	Y
25T	20	14,499,918	20	17,545,445	start1: *MACROD2*; end2: *BFSP1*	del_inssd_169_0/884_0(inssd)	N
25T	20	21,989,044	20	2,252,4254	.	del_inssu_2240_0/1450_0(del)	N
25T	20	11,289,262	20	14,103,596	End2: *MACROD2*	del_inssu_2240_0/1450_0(inssu)	N
25T	20	14,325,201	20	52,594,135	Start1: *MACROD2*; end2: *BCAS1*	del_invers_1235_0/1320_0(del)	Y
25T	20	39,404,181	20	39,842,953	Start1: .; end2: *ZHX3*	del_invers_1235_0/1320_0(invers)	N
25T	20	15,051,355	20	47,058,974	Start1: *MACROD2*; end2: .	transl_intra_118	Y
25T	20	15,859,689	20	54,303,294	Start1: *MACROD2*, end2: .	transl_intra_604	Y
32T	20	15,063,370	20	15,064,551	*MACROD2*	del_5846	Y
32T	20	14,878,395	20	14,914,830	*MACROD2*	del_ins_2165	Y
32T	20	15,184,129	20	15,190,839	*MACROD2*	del_ins_2864	Y
42T	20	14,951,421	20	15,448,407	*MACROD2*	del_629	Y
42T	20	14,973,010	20	15,005,406	*MACROD2*	del_1058	Y
42T	20	14,910,318	20	15,506,385	*MACROD2*	tandem_dup_1249	Y
51T	19	19,268,589	20	14,534,171	Start1: *MEF2B*, *MEF2BNB-MEF2B*; end2: *MACROD2*	transl_inter_1050	Y

We evaluated MACROD2 expression in the 49 HCCs by quantitative real-time polymerase chain reaction (qRT-PCR) and immunohistochemistry. MACROD2 expression levels were lower in the tumor samples than in the paired adjacent non-tumor samples. The difference in the MACROD2 expression level between the tumor and the adjacent normal tissue was greater among the patients with MACROD2 SVs than among those without MACROD2 SVs (Fig. 1c–e).

### Down-regulation of MACROD2 correlates with poor prognosis in HCC

Because *MACROD2* was frequently affected by SVs in HCC, and those SVs were associated with lower expression levels of MACROD2, we examined MACROD2 expression in a panel of six HCC cell lines and in an independent FFPE cohort comprising 380 HCCs. Quantitative PCR and western blots showed that MACROD2 expression was decreased in all six HCC cell lines, especially those with high metastatic potential (MHCC97L, MHCC97H, and HCCLM3), compared with that in the non-cancerous hepatic cell line L0-2 (Fig. 1f). The down-regulation of MACROD2 was correlated with tumor size (*P* = 0.021), tumor encapsulation (*P* = 0.005), vascular invasion (*P* = 0.020), tumor differentiation (*P* = 0.044), and tumor node metastasis (TNM) staging (*P* = 0.011) in the 380 patients with HCC in the FFPE cohort (Supplementary Table 6). Figure 1g shows representative images of the immunohistochemical results.

At the time of the last follow-up, the patients in the FFPE cohort with high MACROD2 expression had significantly better 1-, 3-, and 5-year survival rates than those with low MACROD2 expression (91.6% vs. 85.8%, 78.9% vs. 59.2%, and 69.8% vs. 45.0%, respectively). Similarly, the patients with low MACROD2 expression had worse prognosis with higher 1-, 3-, and 5-year cumulative recurrence rates than the patients with high MACROD2 expression (33.3% vs. 17.4%, 58.9% vs. 31.6%, and 65.5% vs. 40.3%, respectively; Fig. 1h). Univariate and multivariate analyses revealed that, along with γ-glutamyl transferase test results, tumor size, tumor number, and microvascular invasion, the MACROD2 expression level was an independent prognostic factor for both overall survival (OS) [*P* < 0.001, hazard ratio (HR) = 0.502] and time to recurrence (TTR) (*P* < 0.001, HR = 0.567; Table 2).

**Table 2:** Univariate and multivariate analyses of prognostic factors in HCC (FFPE cohort, *n* = 380).

Variable	TTR			OS	
	HR (95% CI)	*P* value		HR (95% CI)	*P* value
Univariate analysis					
Age, year (≤50 vs. >50)	1.229 (0.934–1.615)	0.14	1.036 (0.768–1.398)		0.817
Sex (female vs. male)	1.387 (0.954–2.016)	0.087	1.093 (0.735–1.625)		0.662
HBsAg (negative vs. positive)	1.387 (0.921–2.087)	0.117	1.172 (0.757–1.814)		0.478
AFP (ng/mL) (≤20 vs. >20)	1.302 (0.991–1.710)	0.058	1.607 (1.175–2.199)		0.003
GGT (U/L) (≤54 vs. >54)	1.698 (1.305–2.210)	0	1.853 (1.378–2.492)		0
Liver cirrhosis (no vs. yes)	1.647 (1.101–2.463)	0.015	1.466 (0.939–2.290)		0.092
Tumor size (cm) (≤5 vs. >5)	1.572 (1.208–2.045)	0.001	2.000 (1.495–2.676)		0
Tumor number (single vs. multiple)	1.695 (1.170–2.456)	0.005	1.825 (1.233–2.702)		0.003
Vascular invasion (no vs. yes)	1.767 (1.349–2.313)	0	1.855 (1.378–2.496)		0
Tumor encapsulation (complete vs. none)	1.312 (1.011–1.704)	0.041	1.300 (0.972–1.739)		0.077
Tumor differentiation (I + II vs. III + IV)	1.443 (1.078–1.931)	0.014	1.746 (1.278–2.386)		0
TNM stage (I vs. II + III + IV)	1.739 (1.337–2.262)	0	2.017 (1.508–2.699)		0
MACROD2 (low vs. high)	0.506 (0.387–0.662)	0	0.475 (0.351–0.642)		0
Multivariate analysis					
AFP (ng/mL) (≤20 vs. >20)	1.168 (0.880–1.551)	0.283	1.427 (1.033–1.972)		0.031
GGT (U/L) (≤54 vs. >54)	1.548 (1.181–2.028)	0.002	1.664 (1.229–2.254)		0.001
Liver cirrhosis (no vs. yes)	1.418 (0.939–2.141)	0.097	NA		NA
Tumor size (cm) 0 (≤5 vs. >5)	1.359 (1.032–1.791)	0.029	1.704 (1.262–2.301)		0.001
Tumor number (single vs. multiple)	1.556 (1.069–2.266)	0.021	1.742 (1.171–2.590)		0.006
Vascular invasion (no vs. yes)	1.411 (1.061–1.878)	0.018	1.381 (1.016–1.878)		0.039
Tumor encapsulation (complete vs. none)	1.177 (0.900–1.540)	0.234	NA		NA
Tumor differentiation (I + II vs. III + IV)	1.315 (0.976–1.773)	0.072	1.476 (1.072–2.031)		0.017
MACROD2 (low vs. high)	0.567 (0.432–0.746)	0	0.502 (0.370–0.680)		0

*Note:* Cox proportional hazards regression model.

*AFP* α-fetoprotein, *GGT* γ-glutamyl transferase, *TNM* tumor node metastasis, *HR* hazard ratio, *CI* confidential interval, *NA* not available.

### MACROD2 deficiency promotes proliferation, colony formation, migration, and invasion of HCC cells

**Fig. 2:**
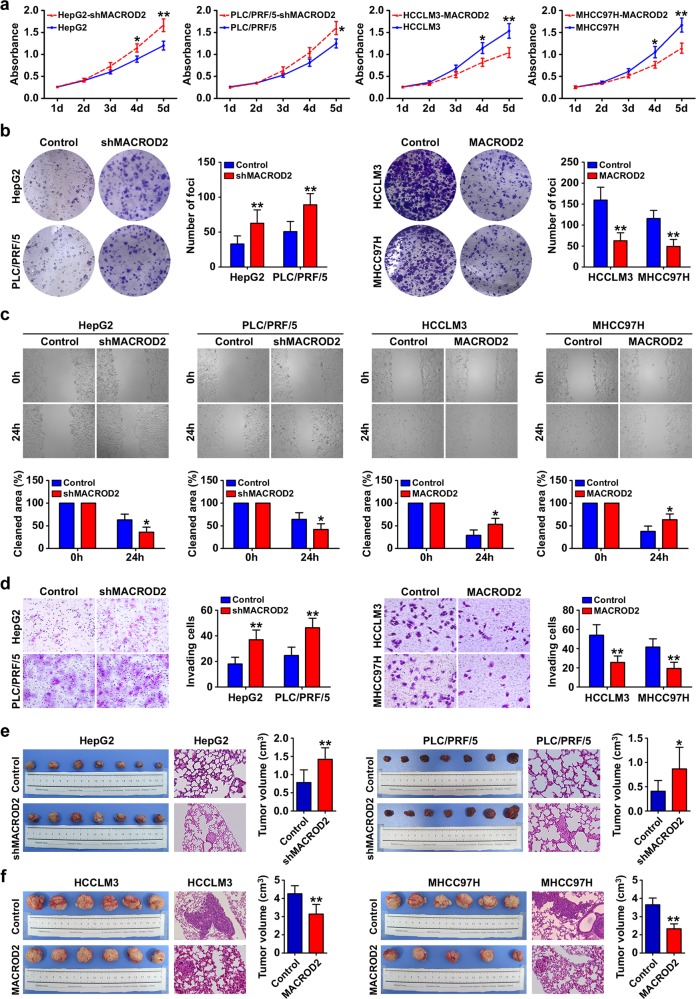
MACROD2 deficiency promotes cell proliferation, colony formation, migration, and invasion in vitro, and tumor growth and metastasis in vivo. **a** Proliferation of MACROD2-knockdown HepG2 and PLC/PRF/5 cells and MACROD2-overexpressing HCCLM3 and MHCC97H cells compared with that of control cells. *P < 0.05, **P < 0.01. **b** Colony formation activity of MACROD2-knockdown HepG2 and PLC/PRF/5 cells and MACROD2-overexpressing HCCLM3 and MHCC97H cells compared with that of control cells. The bar graphs illustrate the quantification of colony formation. **P < 0.01. **c** Cell monolayers were examined microscopically in wound-healing migration assays at 24 h post wounding. *P < 0.05. **d** Invasion of MACROD2-knockdown HepG2 and PLC/PRF/5 cells and MACROD2-overexpressing HCCLM3 and MHCC97H cells compared with that of control cells. The graphs depict the number of invasive cells after 48 h. **P < 0.01. **e**, **f** Macrograph of tumors and H&E-stained images of metastatic nodules in the selected areas of lungs in all groups. *P < 0.05, **P < 0.01. Data are shown as the mean ± SD and are representative of three independent experiments.

To determine the biological effects of MACROD2 expression in HCC cells, we used short hairpin RNA (shRNA) to knock down MACROD2 in HepG2 and PLC/PRF/5 cells, which normally display high levels of MACROD2 expression, and we overexpressed MACROD2 in MHCC97H and HCCLM3 cells, which normally display low levels of MACROD2 expression. We confirmed the stable overexpression or knockdown of MACROD2 in the respective HCC cell lines by qRT-PCR and western blot (Supplementary Fig. 2). The knockdown of MACROD2 in HepG2 and PLC/PRF/5 cells resulted in significant increases the cells’ abilities to proliferate and form colonies. Similarly, the overexpression of MACROD2 in HCCLM3 and MHCC97H cells significantly reduced the proliferation and colony-forming abilities of those cells (Fig. 2a, b). Wound-healing migration assays showed that after 24 h, the rate of wound healing was significantly affected by the level of MACROD2 expression. Compared with the parental cell lines in which the MACROD2 level was unaltered by shRNA or ectopic overexpression, the HCCLM3 and MHCC97H cells with MACROD2 overexpression displayed significantly delayed wound healing, whereas the HepG2 and PLC/PRF/5 cells with MACROD2 knockdown displayed significantly increased rates of wound healing (Fig. 2c). Furthermore, in vitro invasion assays showed that the MACROD2 expression level had an impact on the invasive abilities of the cell lines. The numbers of invasive cells in cultures of HepG2 and PLC/PRF/5 cells with MACROD2 knockdown were significantly higher than those in cultures of parental HepG2 and PLC/PRF/5 cells without MACROD2 knockdown (37.0 ± 7.6 vs. 18.0 ± 5.3 and 46.3 ± 7.4 vs. 24.7 ± 6.5, respectively). Similarly, the number of invasive cells in cultures of HCCLM3 and MHCC97H cells with MACROD2 overexpression was significantly lower than those in cultures of parental HCCLM3 and MHCC97H cells without MACROD2 overexpression (25.7 ± 6.7 vs. 54.0 ± 11.0 and 19.3 ± 6.5 vs. 41.7 ± 8.5, respectively; Fig. 2d).

### MACROD2 deficiency promotes HCC growth and metastasis in vivo

All of the cell lines successfully formed liver tumors after orthotropic transplantation into nude mice. Xenografts of HepG2 and PLC/PRF/5 cells with MACROD2 knockdown formed larger tumors (1.42 ± 0.32 and 0.87 ± 0.44 cm^3^, respectively) than those of parental HepG2 and PLC/PRF/5 cells without MACROD2 knockdown (0.78 ± 0.35 and 0.41 ± 0.22 cm^3^, respectively; Fig. 2e). Similarly, xenografts of HCCLM3 and MHCC97H cells with MACROD2 overexpression formed smaller tumors (4.28 ± 1.06 and 2.66 ± 0.54 cm^3^, respectively) than xenografts derived from parental HCCLM3 and MHCC97H cells without MACROD2 overexpression (6.52 ± 0.88 and 5.31 ± 0.73 cm^3^, respectively; Fig. 2f). Pulmonary metastasis occurred in 100% (6/6) of the mice with xenografts of parental HCCLM3 or MHCC97H cells, but in only two of six mice with xenografts of MACROD2-overexpressing HCCLM3 cells and one of five mice with xenografts of MACROD2-overexpressing MHCC97H cells. The parental HCCLM3 and MHCC97H cells also produced more metastatic nodules of each grade than the MACROD2-overexpressing HCCLM3 and MHCC97H cells (Fig. 2f and Supplementary Fig. 3). Pulmonary metastasis occurred in 57.1% (4/7) and 42.9% (3/7) of the mice with xenografts of HepG2 and PLC/PRF/5 cells with MACROD2 knockdown, respectively, whereas none of the mice with xenografts of parental HepG2 or PLC/PRF/5 cells (0/7 in each case) developed pulmonary metastasis (Fig. 2e and Supplementary Fig. 3).

### MACROD2 deficiency induces EMT of HCC cells

**Fig. 3:**
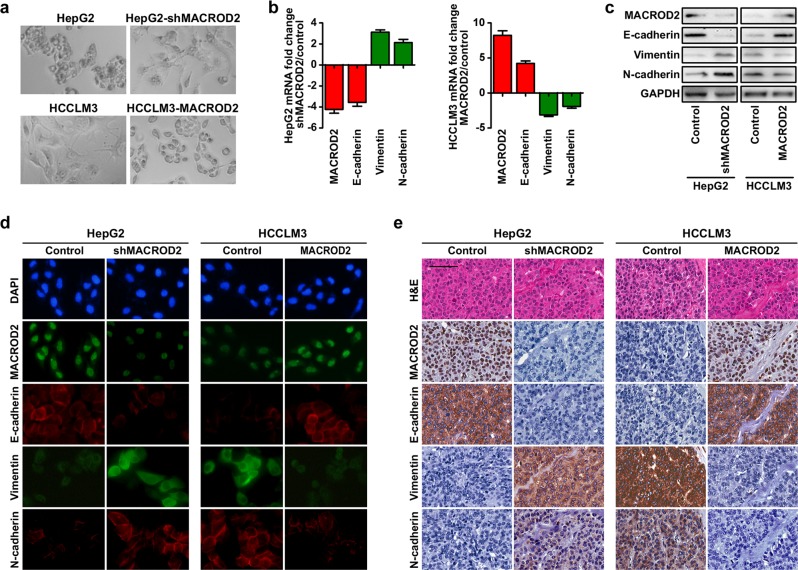
MACROD2 deficiency induces EMT of HCC cells. **a** The cellular morphology of HCC cells with high or low MACROD2 expression. **b** Results of qRT-PCR, **c** western blot, and **d** immunofluorescent staining show changes in EMT marker (E-cadherin, vimentin, and N-cadherin) expression in HCC cell lines. **e** Representative images of xenograft-derived tumor sections. Scale bars = 100 μm. Data are shown as the mean ± SD and are representative of three independent experiments.

Epithelial–mesenchymal transition (EMT) is associated with cancer invasion and metastasis. To determine if there is a relationship between MACROD2 deficiency and the EMT process in HCC cells, we examined the morphology of HCC cells with different levels of MACROD2 expression. As shown in Fig. 3a, parental HepG2 cells and MACROD2-overexpressing HCCLM3 cells, both of which have high MACROD2 expression levels, had a cobblestone-like morphology resembling that of the normal epithelium. By contrast, HCC cells with low MACROD2 expression levels had a spindle-like morphology resembling that of fibroblasts. Screening for epithelial and mesenchymal markers revealed that parental HCCLM3 cells and MACROD2-knockdown HepG2 cells both had phenotypes typical of cells undergoing EMT, including down-regulation of E-cadherin and up-regulation of vimentin and N-cadherin. By contrast, parental HepG2 cells and MACROD2-overexpressing HCCLM3 cells displayed up-regulation of E-cadherin and down-regulation of vimentin and N-cadherin (Fig. 3b, c). Immunofluorescent staining further confirmed that the parental HCCLM3 cells and the MACROD2-knockdown HepG2 cells had the EMT phenotype (Fig. 3d). In line with those results, tumor tissues derived from MACROD2-knockdown HepG2 cells and also those derived from parental HCCLM3 cells expressed high levels of vimentin and N-cadherin and low levels of E-cadherin (Fig. 3e). Taken together, our results strongly suggest that MACROD2 is involved in regulating the EMT in HCC.

### MACROD2 deficiency activates GSK-3β/β-catenin signaling in HCC cells

**Fig. 4:**
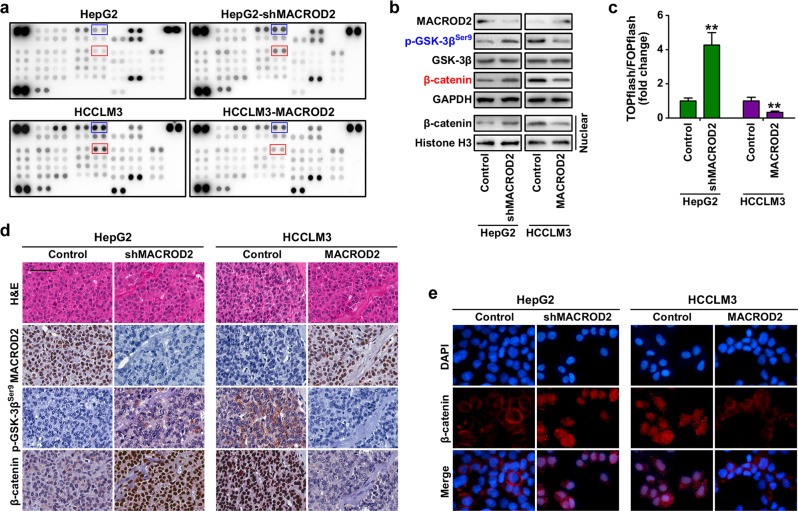
Activation of GSK-3β/β-catenin signaling induced by MACROD2 deficiency in HCC cells. **a** Human phosphokinase array screening of changes in signaling pathways in HCC cells upon alteration of MACROD2 expression. **b** Western blot to validate the expression of p-GSK-3β and β-catenin in HCC cells upon alteration of MACROD2 expression. **c** Results of dual-luciferase assays performed 48 h after transfection of the indicated cells with TOPflash or FOPflash and *Renilla* pRL-TK plasmids. Reporter activity was normalized to *Renilla* luciferase activity. **P < 0.01. **d** Representative images of xenograft-derived tumor sections show p-GSK-3β and β-catenin expression. Scale bars = 100 μm. **e** Immunofluorescence staining showing subcellular β-catenin localization in the indicated cells. Data are shown as the mean ± SD and are representative of three independent experiments.

To investigate the mechanism of MACROD2 function in HCC cells, we used a phosphokinase array and HCC cells with altered MACROD2 expression. We found that phosphorylated-glycogen synthase kinase-3β (p-GSK-3β) and β-catenin were up-regulated by more than 100% in HepG2 cells after knockdown of MACROD2, whereas they were both down-regulated by more than 50% in HCCLM3 cells following MACROD2 overexpression (Fig. 4a). Western blot analysis validated those results (Fig. 4b). The results of a β-catenin reporter assay revealed that MACROD2 knockdown strongly increased the transactivating activity of β-catenin in HepG2 cells. Conversely, MACROD2 overexpression reduced TCF/LEF (T cell factor/lymphoid enhancer-binding factor) activities in HCCLM3 cells (Fig. 4c). Furthermore, subcellular fractionation (Fig. 4b), immunohistochemistry (Fig. 4d), and immunofluorescence (Fig. 4e) assays revealed that MACROD2 knockdown resulted in substantial β-catenin accumulation in the nuclei of HepG2 cells, whereas MACROD2 overexpression led to a decrease in the level of β-catenin in the nuclei of HCCLM3 cells. Those results suggest that MACROD2 deficiency activates GSK-3β/β-catenin signaling in HCC cells.

### Inhibition of GSK-3β/β-catenin signaling attenuates MACROD2 deficiency-mediated HCC progression

**Fig. 5:**
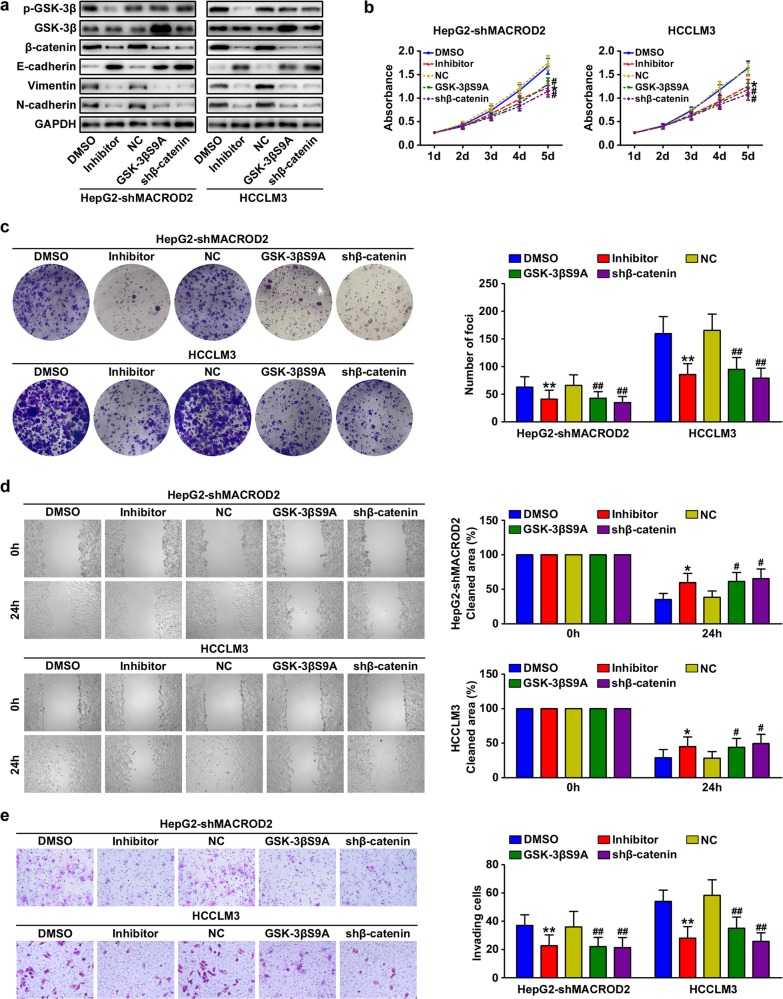
Inhibition of GSK-3β/β-catenin signaling attenuates MACROD2 deficiency-mediated HCC progression. **a** Western blots showing the expression of the indicated molecules in MACROD2-deficient HCC cells treated with the GSK-3β inhibitor CHIR-99021, transfected with GSK-3βS9A, or with knockdown of β-catenin. **b** Proliferation of MACROD2-knockdown HepG2 cells and parental HCCLM3 cells treated with GSK-3β inhibitor, transfected with GSK-3βS9A, or with knockdown of β-catenin. *P < 0.05 compared with DMSO; ^#^*P* < 0.05 compared with NC. **c** Colony formation activity of MACROD2-knockdown HepG2 cells and parental HCCLM3 cells treated with GSK-3β inhibitor, transfected with GSK-3βS9A, or with knockdown of β-catenin. The bar graphs illustrate the quantification of colony formation. **P < 0.01 compared with DMSO; ^##^*P* < 0.01 compared with NC. **d** Results of microscopic examination of cell monolayers in wound-healing migration assays at 24 h post wounding. *P < 0.05 compared with DMSO; ^#^*P* < 0.05 compared with NC. **e** Invasion of MACROD2-knockdown HepG2 cells and parental HCCLM3 cells treated with GSK-3β inhibitor, transfected with GSK-3βS9A, or with knockdown of β-catenin. The bar graphs illustrate the quantification of colony formation. **P < 0.01 compared with DMSO; ^##^*P* < 0.01 compared with NC. Data are shown as the mean ± SD and are representative of three independent experiments.

We explored the role of GSK-3β/β-catenin signaling in MACROD2 deficiency-mediated HCC growth and metastasis. We first knocked down β-catenin in MACROD2-knockdown HepG2 cells and in parental HCCLM3 cells with low MACROD2 expression. The β-catenin-knockdown cells displayed increased E-cadherin expression and decreased vimentin and N-cadherin expression (Fig. 5a). In addition, the β-catenin-knockdown cells displayed reduced proliferation, colony formation, migration, and invasion (Fig. 5b–e). Next, we treated HCC cells with the GSK-3β inhibitor CHIR-99021, and we also introduced a constitutively active mutant GSK-3β (GSK-3βS9A) into the HCC cells. Figure 5a shows the GSK-3β and p-GSK-3β expression in the cells containing GSK-3βS9A and in the cells treated with CHIR-99021. The CHIR-99021 treatment and the introduction of GSK-3βS9A both resulted in reduced β-catenin expression; the suppression of MACROD2-deficient HCC cell proliferation, colony formation, migration, and invasion; and the reversal of the EMT phenotype (Fig. 5a–e). Those results confirm that MACROD2 deficiency promotes HCC progression and induces EMT through the activation of GSK-3β/β-catenin signaling.

### Decreased MACROD2 expression is associated with GSK-3β/β-catenin signaling activation and the EMT phenotype in clinical HCC samples

**Fig. 6:**
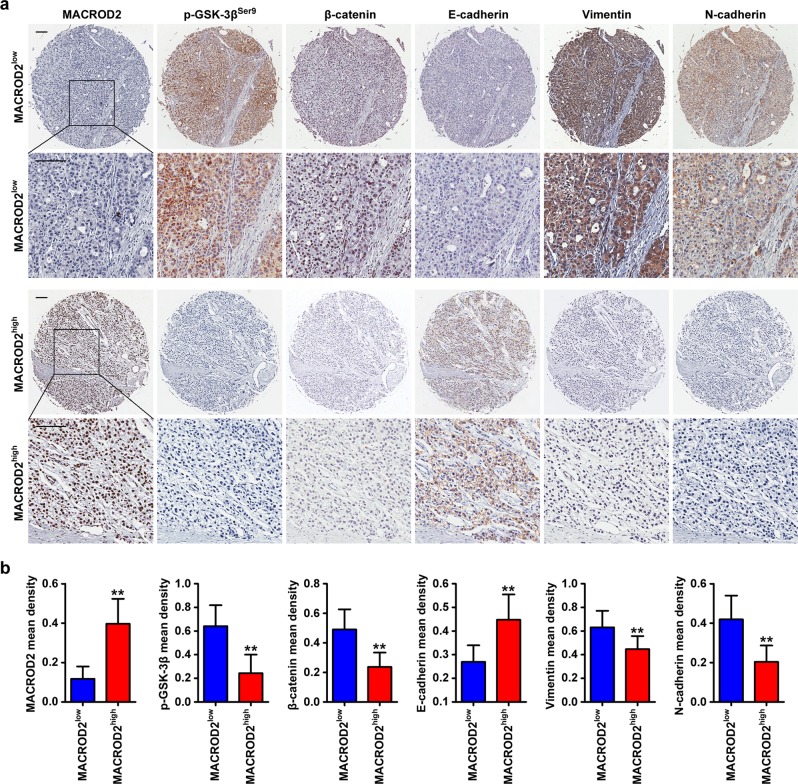
Levels of MACROD2, p-GSK-3β, β-catenin, E-cadherin, vimentin, and N-cadherin in HCC tissues. **a** HCC tumor samples showing MACROD2, p-GSK-3β, β-catenin, E-cadherin, vimentin, and N-cadherin expression. Patient 1: low MACROD2 expression; patient 2: high MACROD2 expression. Scale bar = 100 μm. **b** The bar graphs illustrate the expression levels of p-GSK-3β, β-catenin, E-cadherin, vimentin, and N-cadherin in HCC tumor sections (FFPE cohort, *n* = 380) from patients with low (*n* = 190) or high (*n* = 190) MACROD2 expression. **P < 0.01. Data are representative of three independent experiments.

We performed immunohistochemical staining to measure the expression of MACROD2, p-GSK-3β, β-catenin, and the EMT markers E-cadherin, vimentin, and N-cadherin in primary tumor tissues from the 380 patients in the FFPE cohort (Fig. 6a). Patients with low MACROD2 expression displayed elevated levels of p-GSK-3β and greater nuclear accumulation of β-catenin. The tumors of those patients also displayed a typical EMT phenotype characterized by down-regulation of E-cadherin and up-regulation of vimentin and N-cadherin. Conversely, patients with high MACROD2 expression displayed low levels of p-GSK-3β and β-catenin, up-regulation of E-cadherin, and down-regulation of vimentin and N-cadherin (Fig. 6b).

## Discussion

In this study, we identified a new tumor suppressor gene, *MACROD2*, which was the gene most frequently affected by SVs in HCC. MACROD2 deficiency promoted tumor growth and metastasis and induced EMT in HCC. Mechanistic studies showed that MACROD2 deficiency-induced HCC progression was mediated by the activation of p-GSK-3β/β-catenin signaling. We validated those findings in samples of tumor tissues from patients with HCC and found that low MACROD2 expression was predictive of poor patient outcomes.

Cancer genomes harbor a variety of somatic mutations, ranging in size from a single or a few nucleotides [e.g., point mutations (SNVs) and small insertions/deletions (Indels)] to large chromosomal regions [e.g., SVs and copy-number variations (CNVs)]. Some of the somatic mutations in cancer cells play important roles in tumor development and disease progression^16^. Genomic SVs in particular are a hallmark of cancer progression^16^. Because of their size, SVs affect a greater fraction of the genome than SNPs, resulting in profound phenotypic effects^17^. We found that *MACROD2*, *RBFOX1*, and *LRP1B* were frequently affected by SVs in Chinese patients with HCC, suggesting that SVs in those genes might play a role in tumor development and progression. In a recent study of a Japanese HCC cohort, *LRP1B*, *MACROD2*, and *TTC28* were found to contain SV breakpoints in ≥5% of samples^11^. *MACROD2* was the gene most frequently affected by SVs, so we were interested in evaluating its biological role and mechanistic impact on HCC growth and metastasis.

*MACROD2* is a protein-coding gene located at a fragile site on human chromosome 20. The MACROD2 protein is a deacetylase involved in the removal of ADP-ribose from mono-ADP-ribosylated proteins^18^. ADP-ribosylation, or the addition of ADP moieties to proteins, is a common post-translational modification that plays roles in various biological processes, including DNA damage repair, chromatin reorganization, transcriptional regulation, apoptosis, and mitosis^19,20^. The deletion of MACROD2 in colorectal cancer promotes chromosome instability and intestinal tumor growth^21^. In breast cancer, MACROD2 overexpression mediates estrogen-independent growth and tamoxifen resistance^22^. In this study, we identified *MACROD2* as the gene that was most frequently affected by SVs in 49 patients with HCC. In four of the five samples containing *MACROD2* SVs, the SV was a structural deletion. Although some other studies have revealed frequent CNVs or SNVs of *MACROD2*^21^, that gene was not recurrently amplified or deleted in our samples, nor did it contain SNVs or Indels (data not shown). This suggests that in HCC, SVs are the main type of genetic variation affecting *MACROD2*, which may contribute to its low expression level in HCC. Survival analysis revealed that patients with high MACROD2 expression had greater OS and a lower cumulative recurrence rate than patients with low MACROD2 expression. MACROD2 expression level had also prognostic value for both OS and TTR among patients grouped according to the refined TNM stages (Supplementary Fig. 4).

Through in vitro and in vivo experiments, we uncovered the tumor suppressor role of MACROD2 in HCC growth and metastasis. Its deficiency promotes cell proliferation, colony formation, migration, and invasion in vitro and tumor growth and metastasis in vivo. MACROD2 can reverse the inhibition of GSK-3β catalyzed by mono-ADP-ribosylated ARTD10^18^. Our results are consistent with that; MACROD2 deficiency enhanced the phosphorylation levels of GSK-3β, leading to increased nuclear accumulation of β-catenin in HCC cells. The association between MACROD2 and p-GSK-3β and β-catenin was further validated in clinical HCC samples, suggesting that MACROD2 deficiency induces the activation of GSK-3β/β-catenin signaling in HCC. The alteration of WNT/β-catenin signaling is one of the main pathway alterations that occurs during HCC development and progression^23^. Mutations in genes involved in WNT pathways, such as *CTNNB1*, *AXIN1*, and APC, contribute to the activation of WNT/β-catenin signaling in more than half of patients with HCC^13^. Our results identify *MACROD2* as another WNT-related gene involved in HCC. MACROD2 deficiency, often due to the presence of SVs, leads to WNT/β-catenin pathway activation by altering GSK-3β regulation. Our results also confirmed that the effects of MACROD2 deficiency on HCC growth and metastasis were dependent on the activation of GSK-3β/β-catenin signaling.

The EMT is an essential part of embryonic development. However, it also plays a much more sinister role by promoting tumor invasion and metastasis. EMT in cancer cells allows single carcinoma cells to disseminate from the site of the primary tumor, resulting in disease progression, metastasis, and poor patient outcomes^24^. Although many factors are involved in the EMT process, it remains unclear what role genes affected by SVs play in promoting EMT and HCC invasion and metastasis. The morphological differences that we observed among HCC cells with different levels of MACROD2 expression suggest that MACROD2 may play a role in EMT induction. We tested the hypothesis by measuring mesenchymal and epithelial markers in various HCC cell lines. We found that HCC cell lines with deficient MACROD2 expression had increased levels of mesenchymal markers and decreased levels of epithelial markers. By contrast, HCC cells that expressed high levels of MACROD2 did not present those same profiles of epithelial and mesenchymal markers. Those results were confirmed in tissue samples from patients with HCC, which demonstrated that MACROD2 deficiency triggers EMT in HCC cells and thus plays an important role in HCC progression.

Taken together, our results delineate the SV events that occurred in Chinese HCCs. We identified *MACROD2* as the gene most frequently affected by SVs, its deficiency promotes HCC growth and metastasis by activating GSK-3β/β-catenin signaling.

## Methods

### Patients and follow-up

For WGS, matched tumor and adjacent normal liver tissues were obtained and snap frozen from 49 patients with primary HCC who received curative resection in 2010 or 2011 in the department of liver surgical oncology of Zhongshan Hospital, Fudan University (WGS cohort). For immunohistochemical and prognostic analysis, tumor and adjacent normal liver tissues were consecutively collected from 380 patients with primary HCC who underwent curative resection in 2006 in the same institution (FFPE cohort; Supplementary Table 1). Patients meeting any of the following criteria were excluded from our study: surgery was palliative rather than curative; received prior treatment such as trans-hepatic artery embolization, chemotherapy, or radiotherapy; had other primary malignancies or inflammatory diseases identified during follow-up. All patients were diagnosed with HCC on the basis of the histopathological criteria of the World Health Organization. The classification scheme of Edmondson and Steiner was used to assign the histological grade of tumor differentiation^25^. The patients’ liver function was scored using the Child–Pugh system. The 2017 International Union Against Cancer TNM classification system was used to determine the tumor stage^26^. Each patient included in the study gave informed consent for participation in the research. The Research Ethics Committee of Zhongshan Hospital granted ethical approval for the use of human subjects.

All patients were monitored after surgery until 30 June 2016 as previously described^8^. Recurrence was diagnosed on the basis of computed tomography, magnetic resonance imaging, digital subtraction angiography, and serum α-fetoprotein (AFP) level, with or without histological confirmation^27^. The TTR was defined as the interval between the surgery and any recurrence (intrahepatic recurrence or extrahepatic metastasis)^8^. OS was defined as the interval between the surgery and death or between the surgery and the last follow-up visit. Data for surviving patients were censored at date of the last follow-up. This study was approved by the Research Ethics Committee of Zhongshan Hospital, and all patients gave informed consent to the treatment and the use of their specimens and data for research and for publication. Our study is compliant with the “Guidance of the Ministry of Science and Technology (MOST) for the Review and Approval of Human Genetic Resources.”

### DNA preparation, capture, and sequencing

Snap-frozen samples of tumor and matched non-cancerous liver tissues were embedded in OCT compound, sectioned using a cryostat, and stained with hematoxylin and eosin. The tumor cells were enriched relative to the normal stromal cells and other normal cells by macrodissection. DNA was extracted according to a general protocol. Library preparation and DNA capture were performed according to the manufacturer’s instructions.

### Whole-genome sequencing

Genomic DNA was randomly broken into fragments to facilitate the construction of insert libraries. For human genome re-sequencing, paired-end libraries of 400–500 bp span size were used. The fragments of the template DNA from the constructed libraries were hybridized to the cell surface and then subjected to amplification to form clusters. The DNA fragments were then sequenced using an Illumina HiSeq X sequencing system. A paired-end read length of 150 bp was used for high-throughput WGS.

### Data quality control

Sequence artifacts, including reads containing adapter contamination, low-quality nucleotides, and unrecognizable nucleotides (“N”), are undoubtedly a barrier to subsequent reliable bioinformatics analysis. Hence, quality control is an essential step and is applied to guarantee a meaningful downstream analysis. The steps of data processing were as follows:

1. Discard pairs of reads in which either read contains adapter contamination.
2. Discard pairs of reads that contain poly-N.
3. Discard pairs of reads in which either read has a proportion of low-quality (Phred quality <5) bases over 50%.

### Reads mapping and detection of somatic SVs

After the reads were processed for quality control, the Burrows-Wheeler Aligner^28^ was used to map them to the reference human genome (UCSC hg19) in BAM format. We performed local realignment of the original BAM alignment using GATK2^29^ and then marked duplicate reads using Sambamba^30^. Somatic SVs were then detected using Meerkat^31^.

### Cell lines and animals

Three HCC cell lines with the same genetic background but different metastatic potential were previously established at our institution: MHCC97L, MHCC97H, and HCCLM3^32–34^. We purchased the normal hepatic cell line L-02 and the low-metastatic-potential HCC cell lines HepG2, Hep3B, and PLC/PRF/5 (American Type Culture Collection) from the Institute of Biochemistry and Cell Biology, Chinese Academy of Sciences (Shanghai, China). All cell lines were maintained according to routine protocols. Male BALB/c nu/nu mice (4–6 weeks old, Shanghai Institute of Material Medicine, Chinese Academy of Science) were housed in specific pathogen-free conditions. All animals were treated humanely in accordance with the Guide for the Care and Use of Laboratory Animals from the National Institutes of Health (NIH publication 86-23 revised 1985).

### Lentiviral vectors and cell transfection

We purchased the following lentivirus vectors from Shanghai GeneChem Co.: expression for MACROD2 and control vector (Ubi-MCS-3FLAG-CMV-EGFP); shRNA-MACROD2 and negative control (hU6-MCS-CMV-EGFP); shRNA-β-catenin and negative control (hU6-MCS-CMV-EGFP). Transduction was performed as previously described^8^. The reporter plasmids containing wild-type (CCTTTGATC; TOPflash) or mutated (CCTTTGGCC; FOPflash) TCF/LEF DNA binding sites were also purchased.

### Luciferase reporter assay

Cells were seeded in triplicate in 24-well plates and allowed to settle for 24 h. Indicated plasmids plus 1 ng pRL-TK *Renilla* plasmid were transfected into the cells using Lipofectamine 2000 Reagent (Life Technologies). Forty-eight hours after transfection, Dual-Luciferase Reporter Assay (Promega) was performed according to the manufacturer’s instructions, as previously described^35^.

### Cell proliferation, colony formation, migration, and Matrigel invasion assays

HCC cells were seeded in 100 μL of media in a 96-well plate (2000 cells/well), and 10 μL CCK-8 solution (Dojindo) was added to the cells at the indicated time points. The cells were then incubated for an additional 2 h. The number of viable cells was determined by absorbance measurements (450 nm).

To assess the colony formation abilities of these cells, 500–1000 cells were seeded into each well of 6-well plates and incubated at 37 °C for 12–16 days. Cells were then fixed with 100% methanol before staining with 0.1% crystal violet. Image-Pro Plus v6.2 (Media Cybernetics) was used to count the megascopic cell colonies.

To evaluate cell migration, we used the scratch wound assay. After culturing cells for 2 days to establish a monolayer, cells were serum starved for 16 h, followed by wounding of the cell monolayer with a plastic 10-μL pipette tip. To remove cellular debris, plates were washed with culture medium twice. Cells were then incubated in normal culture medium containing serum at 37 °C. At the indicated times, we photographed migrating cells at the wound front with an inverted microscope (Leica). For each time point, the percentage of the cleared area was compared with the area at time zero and measured using the Image-Pro Plus v6.2 software.

To assess cell invasion, we used 24-well Transwell plates 8-μm pores (Minipore), which were pre-coated with Matrigel (BD Biosciences). The lower chamber contained 600 μL Dulbecco’s modified Eagle’s medium (DMEM) with 10% fetal bovine serum (FBS). Cells were added to the upper chamber (1 × 10^5^ cells) in 100 μL DMEM supplemented with 1% FBS. After 48 h, we removed the remaining cells and Matrigel in the upper chamber. Cells that had invaded the lower membrane surface were fixed with 4% paraformaldehyde and stained with Giemsa. Cells from five microscopic (×200) fields were counted.

### RNA isolation, reverse transcription, and qRT-PCR

Total RNA was extracted from cell lines and frozen tumor specimens using Trizol reagent (Invitrogen, California, USA). Complementary DNA synthesis was performed using PrimeScript Reverse Transcriptase Reagent Kit (Takara, Osaka, Japan) according to the manufacturer’s instructions.

Amplification and detection were performed using the ABI PRISM 7900 Sequence Detection System (Applied Biosystems). Glyceraldehyde 3-phosphate dehydrogenase (GAPDH) was used as an endogenous control. Levels of MACROD2 were normalized to GAPDH, to yield a 2^−ΔΔCt^ value for relative expression of its transcript. The primers were used as presented in Supplementary Table 2.

### Western blot and phosphokinase array analysis

Western blotting was performed as previously described^36^. Briefly, we generated total cell lysates, and proteins were separated on 10% sodium dodecyl sulfate-polyacrylamide gel electrophoresis, and then transferred the proteins to polyvinylidene difluoride membranes. The membranes were washed and blocked. Primary antibodies (Supplementary Table 3) were applied: anti-MACROD2 (Sigma, HPA049076, 1:200), anti-GSK-3β (Cell Signaling Technology, #9832, 1:1000), anti-p-GSK-3β (Cell Signaling Technology, #9323, 1:1000), anti-β-catenin (Cell Signaling Technology, #9562, 1:1000), anti-E-cadherin (Cell Signaling Technology, #3195, 1:2000), anti-Vimentin (Cell Signaling Technology, #5741, 1:1000), anti-N-cadherin (Cell Signaling Technology, #4061, 1:2000), anti-GAPDH (Cell Signaling Technology, #5174, 1:1000), followed by horseradish-peroxidase-conjugated secondary antibodies. Antibody binding was detected by enhanced chemiluminescence assays. All blots derived from the same experiment were processed in parallel.

The phosphokinase array experiment was performed using the human phosphokinase array blot (R&D Systems; catalog number ARY003B). Protein lysate was incubated with the array membrane, and protein signal was visualized using a chemifluorescence detection system (Bio-Rad) according to the manufacturer’s protocol as described previously^37^. The relative intensity of specific protein expression was determined using the Quantity One software.

### Immunofluorescence assay

For immunofluorescence assays, cells cultured on glass slides were fixed in 4% paraformaldehyde for 15 min. Subsequently, the cells were permeabilized with 0.1% Triton X-100 for 15 min at room temperature, washed with phosphate-buffered saline (PBS), and blocked with PBS containing 1% (w/v) bovine serum albumin (BSA) and 0.15% (w/v) glycine (BSA buffer) for 1 h at room temperature. Cells were treated with primary antibody for 2 h at room temperature: anti-MACROD2 (Sigma, HPA049076, 1:50), anti-β-catenin (Cell Signaling Technology, #9562, 1:100), anti-E-cadherin (Cell Signaling Technology, #3195, 1:100), anti-Vimentin (Cell Signaling Technology, #5741, 1:100), and anti-N-cadherin (Cell Signaling Technology, #4061, 1:100). A negative control (primary antibody omitted) was included on every slide. Cells were then washed with BSA buffer and incubated with 2 μg/mL Alexa Fluor 488-conjugated goat anti-mouse antibody (Molecular Probes, Eugene, OR) for 1 h at room temperature. After rinsing in PBS, the slices were counter-stained with 4′,6-diamidino-2-phenylindole and examined by fluorescence microscopy (Leica Microsystems Imaging Solutions, Cambridge, UK).

### Immunohistochemistry and evaluation of immunohistochemical variables

Immunohistochemical staining was performed by the avidin–biotin–peroxidase complex method as previously described^38^. Briefly, after rehydration and microwave antigen retrieval, primary antibodies (Supplementary Table 3) were applied to slides and incubated at 4 °C overnight: anti-MACROD2 (Sigma, HPA049076, 1:100), anti-p-GSK-3β (Cell Signaling Technology, #9323, 1:100), anti-β-catenin (Cell Signaling Technology, #9562, 1:100), anti-E-cadherin (Cell Signaling Technology, #3195, 1:100), anti-Vimentin (Cell Signaling Technology, #5741, 1:100), and anti-N-cadherin (Cell Signaling Technology, #4061, 1:200), followed incubation with secondary antibody (GK500705, Gene Tech, China) at 37 °C for 30 min. Slides were stained with 3,3′-diaminobenzidine and counter-stained with Mayer’s hematoxylin. Negative controls with omission of primary antibody were included in all assays.

Immunohistochemical staining was assessed by three independent investigators who were blinded to patient characteristics. The inter-observer concordance was 93.9% (357/380). Discrepancies were resolved by consensus. Photographs of three representative fields were captured under high-power magnification (×200) using the Leica QWin Plus v3 software; identical settings were used for each photograph. MACROD2 density in tissue microarray was determined using the Image-Pro Plus v6.2 software (Media Cybernetics Inc., Bethesda, MD). Integrated optical density of all positive MACROD2 staining in each photograph was measured and its ratio to the total area of each photograph was calculated as the MACROD2 density. The median MACROD2 density was determined using immunohistochemistry and used as the cut-off in subsequent analyses. HCC patients with a MACROD2 density higher or lower than the median density were defined as MACROD2^high^ or MACROD2^low^, respectively.

### In vivo assays for tumor growth and metastasis

HepG2, HepG2-shRNA-MACROD2, PLC/PRF/5, PLC/PRF/5-shRNA-MACROD2, HCCLM3, HCCLM3-MACROD2, MHCC97H, and MHCC97H-MACROD2 cells (1 × 10^7^) were suspended in 100 μL serum-free DMEM and Matrigel (BD Biosciences) (1:1) and then injected subcutaneously into the upper left flank region of nude mice. When the subcutaneous tumor reached ~1 cm in length (~4 weeks after injection), it was removed, minced into small pieces of equal volume (2 × 2 × 2 mm^3^), and transplanted into the livers of nude mice. All mice were monitored once every 3 days and killed 5 weeks later. The volume of tumors was calculated in mm^3^ as follows: *V* = *ab*^2^/2 (with *a* and *b* representing the largest and smallest tumor diameters measured at necropsy)^39^. Lungs were removed and embedded in paraffin and the total number of lung metastases was counted under the microscope as described previously^40^. The metastases were classified into four grades on the basis of the number of tumor cells present at the maximal section for each metastatic lesion: grade I, ≤20 tumor cells; grade II, 20–50 tumor cells; grade III, 50–100 tumor cells; and grade IV, >100 tumor cells.

### Statistical analysis

Statistical analyses were performed in the R environment or using SPSS 16.0 for Windows. The data were expressed as the mean ± standard deviation of three independent experiments, unless otherwise specified. Quantitative differences between groups were assessed using Student’s *t* test. Categorical data were compared using the *χ*^2^ test or Fisher’s exact test. The rates of OS and cumulative recurrence were calculated using the Kaplan–Meier method and compared using the log-rank test. The Cox proportional hazards model was used for univariate and multivariate analyses. The threshold for statistical significance in all tests was set at *P* < 0.05.

### Reporting summary

Further information on experimental design is available in the Nature Research Reporting Summary linked to this article.

## Supplementary information

Supplementary Information

Reporting Summary

## Data Availability

Data that support the findings of this study have been deposited in the Sequence Read Archive under accession code number PRJNA504942.
