# Supplementary material for: MACROD2 deficiency promotes hepatocellular carcinoma growth and metastasis by activating GSK-3β/β-catenin signaling
Source: NPJ Genom Med. 2020 Apr 1;5:15. doi: 10.1038/s41525-020-0122-7 (PMC7113304; doi:10.1038/s41525-020-0122-7)
Supplement: Supplementary file 1 — Supplementary Information [file 41525_2020_122_MOESM1_ESM.pdf]

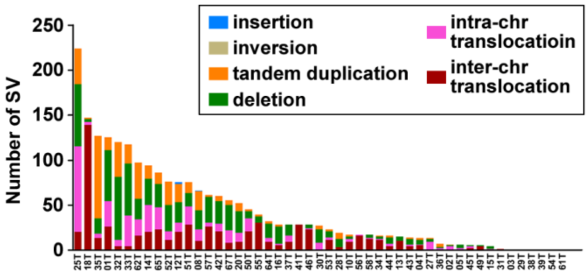


**Supplementary Figure 1** The number of structural variations in 49 HCCs, including insertions, inversions, tandem duplications, deletions, inter-, and intra-chromosomal translocations.

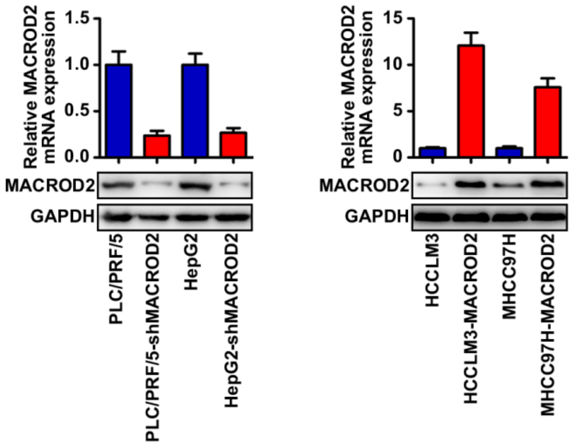


**Supplementary Figure 2** MACROD2 expression in parental cells and stably transfected cells.

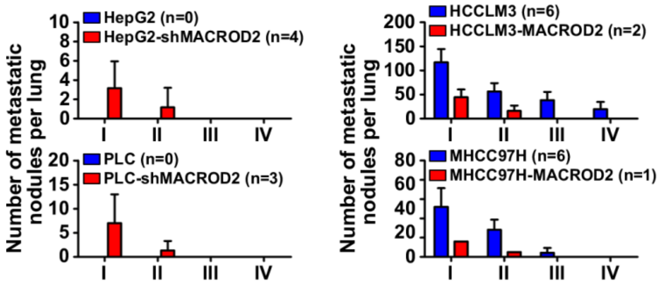


**Supplementary Figure 3** The statistics of pulmonary metastasis grades in each group.

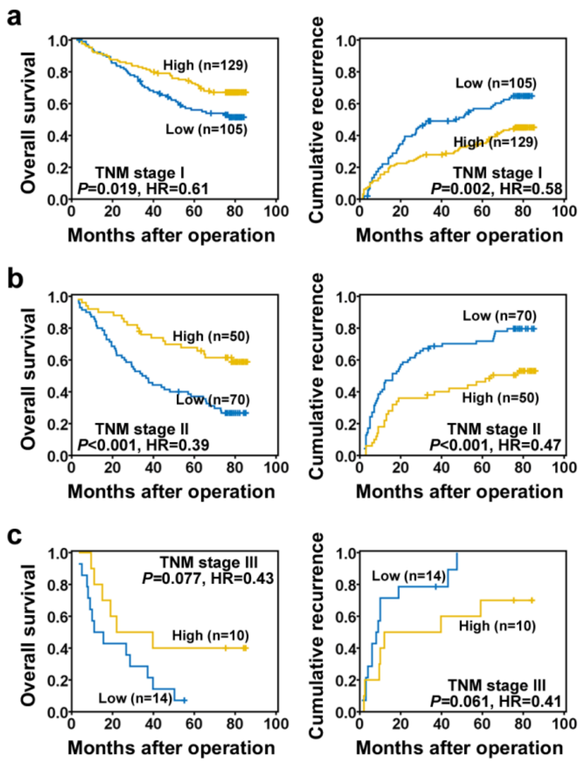


**Supplementary Figure 4** Kaplan-Meier survival analysis showing survival rates and cumulative recurrence rates on the basis of MACROD2 expression among patients grouped according to the refined TNM stages in the FFPE cohort.

**Supplementary Table 1. Clinicopathologic characteristics of two cohorts of patients with HCC**

| Variable | | WGS (n=49) | | FFPE (n=380) | |
|---|---|---|---|---|---|
| | | N | % | N | % |
| Age(year) | ≤50 | 22 | 44.9 | 150 | 39.5 |
| | >50 | 27 | 55.1 | 230 | 60.5 |
| Sex | Female | 3 | 6.1 | 65 | 17.1 |
| | Male | 46 | 93.9 | 315 | 82.9 |
| HBsAg | Negative | 2 | 4.1 | 54 | 14.2 |
| | Positive | 47 | 95.9 | 326 | 85.8 |
| HCV | Negative | 49 | 100 | 377 | 99.2 |
| | Positive | 0 | 0 | 3 | 0.8 |
| AFP(ng/ml) | ≤20 | 24 | 49 | 145 | 38.2 |
| | >20 | 25 | 51 | 235 | 61.8 |
| GGT(U/L) | ≤54 | 19 | 38.8 | 195 | 51.3 |
| | >54 | 30 | 61.2 | 185 | 48.7 |
| Liver cirrhosis | No | 15 | 30.6 | 61 | 16.1 |
| | yes | 34 | 69.4 | 319 | 83.9 |
| Tumor size(cm) | ≤5 | 32 | 65.3 | 232 | 61.1 |
| | >5 | 17 | 34.7 | 148 | 38.9 |
| Tumor number | Single | 44 | 89.8 | 337 | 88.7 |
| | Multiple | 5 | 10.2 | 43 | 11.3 |
| Vascular invasion | No | 34 | 69.4 | 261 | 68.7 |
| | Yes | 15 | 30.6 | 119 | 31.3 |
| Tumor encapsulation | complete | 29 | 59.2 | 211 | 55.5 |
| | none | 20 | 40.8 | 169 | 44.5 |
| Tumor differentiation | I+II | 32 | 65.3 | 285 | 75 |
| | III+IV | 17 | 34.7 | 95 | 25 |
| TNM stage | I | 31 | 63.3 | 234 | 61.6 |
| | II | 15 | 30.6 | 120 | 31.6 |
| | III | 2 | 4.1 | 24 | 6.3 |
| | IV | 1 | 2 | 2 | 0.5 |

**Supplementary Table 2. Primers used in the study**

| **Primers for *q*RT-PCR** | | **sequences (5’------3’)** |
|---|---|---|
| MACROD2 | sense | 5’- TGACCTTAGAAGAGAGACGCAAA -3’ |
| | antisense | 5’- TCTTCACCTGGGATGTTTCC -3’ |
| E-cadherin | sense | 5’-AGCCCCGCCTTATGATTCTCTG-3’ |
| | antisense | 5’-TGCCCCATTCGTTCAAGTAGTCAT-3’ |
| Vimentin | sense. | 5’-CCTTGACATTGAGATTGCCACCTA-3’ |
| | antisense | 5’-TCATCGTGATGCTGAGAAGTTTCG-3’ |
| N-cadherin | sense： | 5’- GATGAAACGCCGGGATAAAGAAC -3’ |
| | antisense： | 5’- GCTGCAGCTGGCTCAAGTCATAG -3’ |
| GAPDH | sense： | 5’-GGTATGACAACGAATTTGGC-3’ |
| | antisense： | 5’-GAGCACAGGGTACTTTATTG-3’ |

**Supplementary Table 3. Primary antibodies for WB, IHC and IF**

| Protein | Concentration for WB | Concentration for IHC | Concentration for IF | Specificity | Company | Catalogue numbers |
|---|---|---|---|---|---|---|
| MACROD2 | 1:200 | 1:100 | 1:50 | Rabbit Polyclonal | Sigma | HPA049076 |
| GSK-3β | 1:1000 | / | / | Rabbit Monoclonal | Cell Signaling Technology | 9832 |
| p-GSK-3β | 1:1000 | 1:100 | / | Rabbit Monoclonal | Cell Signaling Technology | 9323 |
| β-catenin | 1:1000 | 1:100 | 1:100 | Rabbit Monoclonal | Cell Signaling Technology | 9562 |
| E-cadherin | 1: 2000 | 1:100 | 1:100 | Rabbit Monoclonal | Cell Signaling Technology | 3195 |
| Vimentin | 1: 1000 | 1:100 | 1:100 | Rabbit Monoclonal | Cell Signaling Technology | 5741 |
| N-cadherin | 1: 2000 | 1:200 | 1:100 | Rabbit Monoclonal | Cell Signaling Technology | 4061 |
| GAPDH | 1:1000 | / | / | Rabbit Monoclonal | Cell Signaling Technology | 5174 |

Abbreviations: WB, western blot; IHC, immunohistochemistry; IF, immunofluorescence.

**Supplementary Table 4. The average depth WGS in 49 primary HCCs**

| No. | Sample ID | Tumor ID | Depth | Normal ID | Depth | Sequencing |
|---|---|---|---|---|---|---|
| 1 | HCC-1 | 01T | 53.2 | 01P | 36.2 | WGS |
| 2 | HCC-2 | 02T | 53.9 | 02P | 37.5 | WGS |
| 3 | HCC-3 | 03T | 57.1 | 03P | 35.8 | WGS |
| 4 | HCC-4 | 04T | 55.8 | 04P | 35.4 | WGS |
| 5 | HCC-5 | 05T | 52.6 | 05P | 34.4 | WGS |
| 6 | HCC-8 | 08T | 49.8 | 08P | 31.6 | WGS |
| 7 | HCC-10 | 10T | 51.0 | 10P | 34.4 | WGS |
| 8 | HCC-12 | 12T | 50.4 | 12P | 33.8 | WGS |
| 9 | HCC-13 | 13T | 55.3 | 13P | 30.5 | WGS |
| 10 | HCC-14 | 14T | 57.6 | 14P | 34.9 | WGS |
| 11 | HCC-15 | 15T | 50.8 | 15P | 34.0 | WGS |
| 12 | HCC-16 | 16T | 61.0 | 16P | 35.3 | WGS |
| 13 | HCC-18 | 18T | 61.3 | 18P | 34.4 | WGS |
| 14 | HCC-20 | 20T | 59.1 | 20P | 37.4 | WGS |
| 15 | HCC-25 | 25T | 60.4 | 25P | 33.3 | WGS |
| 16 | HCC-27 | 27T | 48.6 | 27P | 34.0 | WGS |
| 17 | HCC-28 | 28T | 54.5 | 28P | 39.9 | WGS |
| 18 | HCC-29 | 29T | 61.4 | 29P | 32.3 | WGS |
| 19 | HCC-30 | 30T | 50.5 | 30P | 31.6 | WGS |
| 20 | HCC-31 | 31T | 56.9 | 31P | 40.9 | WGS |
| 21 | HCC-32 | 32T | 60.9 | 32P | 32.7 | WGS |
| 22 | HCC-33 | 33T | 55.2 | 33P | 34.7 | WGS |
| 23 | HCC-34 | 34T | 49.4 | 34P | 35.4 | WGS |
| 24 | HCC-35 | 35T | 59.6 | 35P | 38.2 | WGS |
| 25 | HCC-36 | 36T | 52.2 | 36P | 35.5 | WGS |
| 26 | HCC-37 | 37T | 49.1 | 37P | 35.9 | WGS |
| 27 | HCC-38 | 38T | 59.9 | 38P | 35.8 | WGS |
| 28 | HCC-39 | 39T | 52.1 | 39P | 35.8 | WGS |
| 29 | HCC-41 | 41T | 63.4 | 41P | 36.5 | WGS |
| 30 | HCC-42 | 42T | 53.7 | 42P | 43.9 | WGS |
| 31 | HCC-43 | 43T | 56.1 | 43P | 36.9 | WGS |
| 32 | HCC-44 | 44T | 55.0 | 44P | 35.3 | WGS |
| 33 | HCC-45 | 45T | 59.5 | 45P | 39.3 | WGS |
| 34 | HCC-46 | 46T | 54.4 | 46P | 44.0 | WGS |
| 35 | HCC-49 | 49T | 31.6 | 49P | 32.7 | WGS |
| 36 | HCC-50 | 50T | 64.0 | 50P | 36.5 | WGS |
| 37 | HCC-51 | 51T | 55.5 | 51P | 37.6 | WGS |
| 38 | HCC-52 | 52T | 64.9 | 52P | 32.6 | WGS |
| 39 | HCC-53 | 53T | 42.3 | 53P | 40.5 | WGS |
| 40 | HCC-54 | 54T | 45.7 | 54P | 34.3 | WGS |
| 41 | HCC-55 | 55T | 61.6 | 55P | 30.7 | WGS |
| 42 | HCC-56 | 56T | 58.4 | 56P | 31.9 | WGS |
| 43 | HCC-57 | 57T | 64.0 | 57P | 43.5 | WGS |
| 44 | HCC-58 | 58T | 35.5 | 58P | 40.9 | WGS |
| 45 | HCC-61 | 61T | 37.6 | 61P | 35.8 | WGS |
| 46 | HCC-62 | 62T | 59.5 | 62P | 37.5 | WGS |
| 47 | HCC-64 | 64T | 52.2 | 64P | 42.2 | WGS |
| 48 | HCC-65 | 65T | 59.2 | 65P | 34.6 | WGS |
| 49 | HCC-67 | 67T | 57.8 | 67P | 40.1 | WGS |

**Supplementary Table 5. The list of somatic SVs in 49 primary HCCs involved in WGS**

| ID | Chr | Start | End | GeneName | Func | cytoBand | Repeat | MATEID | EVENT |
|---|---|---|---|---|---|---|---|---|---|
| 01T | 1 | 45952829 | 45952829 | TESK2 | intronic | 1p34.1 | . | bnd_204 | del_inss_1920_0/3209_0 |
| 01T | 1 | 45952848 | 45952848 | TESK2 | intronic | 1p34.1 | . | bnd_206 | del_inss_1920_0/3209_0 |
| 01T | 1 | 87221044 | 87221044 | . | intergenic | 1p22.3 | Score=2587;Name="182655:AluY(SINE)" | bnd_2 | del_1472 |
| 01T | 1 | 87234322 | 87234322 | . | intergenic | 1p22.3 | Score=1295;Name="182682:MLT1I(LTR)" | bnd_1 | del_1472 |
| 01T | 1 | 150230886 | 150230886 | CA14 | intronic | 1q21.2 | . | bnd_208 | transl_inter_709 |
| 01T | 1 | 166221041 | 166221041 | . | intergenic | 1q24.1 | Score=17320;Name="284276:L1PA3(LINE)" | bnd_142 | transl_intra_480_0 |
| 01T | 1 | 169562978 | 169562978 | SELP | intronic | 1q24.2 | . | bnd_210 | transl_inter_6524 |
| 01T | 1 | 170215676 | 170215676 | . | intergenic | 1q24.2 | Score=5302;Name="291368:L1MA8(LINE)" | bnd_4 | del_2048 |
| 01T | 1 | 170216448 | 170216448 | . | intergenic | 1q24.2 | . | bnd_3 | del_2048 |
| 01T | 1 | 174680217 | 174680217 | RABGAP1L | intronic | 1q25.1 | Score=3012;Name="299193:LTR12C(LTR)" | bnd_141 | transl_intra_480_0 |
| 01T | 1 | 183643458 | 183643458 | RGL1 | intronic | 1q25.3 | . | bnd_109 | del_insou_1837_0/2927_0 |
| 01T | 1 | 184871737 | 184871737 | FAM129A | intronic | 1q25.3 | . | bnd_107 | del_insou_1837_0/2927_0 |
| 01T | 1 | 186723390 | 186723390 | . | intergenic | 1q31.1 | . | bnd_164 | transl_intra_6138 |
| 01T | 1 | 192326860 | 192326860 | RGS21 | intronic | 1q31.2 | . | bnd_163 | transl_intra_6138 |
| 01T | 1 | 194440435 | 194440435 | . | intergenic | 1q31.3 | . | bnd_108 | del_insou_1837_0/2927_0 |
| 01T | 1 | 195495054 | 195495054 | . | intergenic | 1q31.3 | Score=8775;Name="335438:L1M1(LINE)" | bnd_110 | del_insou_1837_0/2927_0 |
| 01T | 1 | 211342135 | 211342135 | . | intergenic | 1q32.2 | Score=10112;Name="363741:MSTA-int(LTR)" | bnd_212 | transl_inter_4491 |
| 01T | 1 | 211360143 | 211360143 | . | intergenic | 1q32.2 | Score=2583;Name="363786:L1MC4(LINE)" | bnd_214 | transl_inter_1440 |
| 01T | 1 | 219419364 | 219419364 | . | intergenic | 1q41 | . | bnd_6 | del_650 |
| 01T | 1 | 219436315 | 219436315 | . | intergenic | 1q41 | Score=15796;Name="377816:L1PBa(LINE)" | bnd_5 | del_650 |
| 01T | 2 | 13574373 | 13574373 | . | intergenic | 2p24.3 | Score=2279;Name="2256401:THE1B(LTR)" | bnd_114 | transl_intra_1127_0 |
| 01T | 2 | 13574375 | 13574375 | . | intergenic | 2p24.3 | Score=2279;Name="2256401:THE1B(LTR)" | bnd_116 | transl_intra_3317_0 |
| 01T | 2 | 18479409 | 18479409 | . | intergenic | 2p24.2 | . | bnd_115 | transl_intra_3317_0 |
| 01T | 2 | 20562530 | 20562530 | . | intergenic | 2p24.1 | . | bnd_113 | transl_intra_1127_0 |
| 01T | 2 | 78727678 | 78727678 | . | intergenic | 2p12 | Score=9850;Name="2375136:L1PA6(LINE)" | bnd_152 | transl_intra_5205_0 |
| 01T | 2 | 116622316 | 116622316 | . | intergenic | 2q14.1 | Score=2735;Name="2433718:Charlie1(DNA)" | bnd_151 | transl_intra_5205_0 |
| 01T | 2 | 138899366 | 138899366 | . | intergenic | 2q22.1 | Score=6291;Name="2472693:L1PA5(LINE)" | bnd_223 | transl_inter_3248 |
| 01T | 2 | 192962956 | 192962956 | TMEFF2 | intronic | 2q32.3 | . | bnd_36 | del_4199 |
| 01T | 2 | 192963441 | 192963441 | TMEFF2 | intronic | 2q32.3 | . | bnd_35 | del_4199 |
| 01T | 3 | 2460666 | 2460666 | CNTN4 | intronic | 3p26.3 | . | bnd_38 | del_2973 |
| 01T | 3 | 2465686 | 2465686 | CNTN4 | intronic | 3p26.3 | Score=800;Name="2912231:L1PA5(LINE)" | bnd_37 | del_2973 |
| 01T | 3 | 56147401 | 56147401 | ERC2 | intronic | 3p14.3 | . | bnd_240 | transl_inter_5243 |
| 01T | 3 | 59823727 | 59823727 | FHIT | intronic | 3p14.2 | . | bnd_40 | del_5212 |
| 01T | 3 | 59824408 | 59824408 | FHIT | intronic | 3p14.2 | Score=338;Name="3019829:MIR(SINE)" | bnd_39 | del_5212 |
| 01T | 3 | 93727997 | 93727997 | ARL13B | intronic | 3q11.1 | . | bnd_136 | transl_intra_4016_0 |
| 01T | 3 | 98908869 | 98908869 | . | intergenic | 3q12.1 | Score=5521;Name="3080459:L1M1(LINE)" | bnd_138 | transl_intra_3045_0 |

| | | | | | | | | | |
|---|---|---|---|---|---|---|---|---|---|
| 01T | 3 | 98914048 | 98914048 | . | intergenic | 3q12.1 | Score=19105;Name="3080461:L1MA2(LINE)" | bnd_242 | transl_inter_5411 |
| 01T | 3 | 100573591 | 100573591 | ABI3BP | intronic | 3q12.2 | . | bnd_102 | del_insod_1397_0/4989_0 |
| 01T | 3 | 101115137 | 101115137 | SENP7 | intronic | 3q12.3 | . | bnd_104 | del_insod_1397_0/4989_0 |
| 01T | 3 | 101327652 | 101327652 | . | intergenic | 3q12.3 | Score=2065;Name="3084420:AluSg4(SINE)" | bnd_154 | transl_intra_4203_0 |
| 01T | 3 | 103165658 | 103165658 | . | intergenic | 3q13.11 | Score=4110;Name="3087533:L1P4d(LINE)" | bnd_42 | del_1310 |
| 01T | 3 | 104072867 | 104072867 | . | intergenic | 3q13.11 | . | bnd_103 | del_insod_1397_0/4989_0 |
| 01T | 3 | 104335547 | 104335547 | . | intergenic | 3q13.11 | . | bnd_44 | del_3033 |
| 01T | 3 | 104335568 | 104335568 | . | intergenic | 3q13.11 | . | bnd_41 | del_1310 |
| 01T | 3 | 104502207 | 104502207 | . | intergenic | 3q13.11 | Score=1630;Name="3089668:AluJo(SINE)" | bnd_101 | del_insod_1397_0/4989_0 |
| 01T | 3 | 104504246 | 104504246 | . | intergenic | 3q13.11 | . | bnd_137 | transl_intra_3045_0 |
| 01T | 3 | 104793859 | 104793859 | . | intergenic | 3q13.11 | Score=1591;Name="3090153:L1MEc(LINE)" | bnd_43 | del_3033 |
| 01T | 3 | 110924376 | 110924376 | . | intergenic | 3q13.13 | Score=2874;Name="3100344:HERV16-int(LTR)" | bnd_46 | del_930 |
| 01T | 3 | 110955370 | 110955370 | . | intergenic | 3q13.13 | Score=602;Name="3100398:MSTA(LTR)" | bnd_45 | del_930 |
| 01T | 3 | 112412539 | 112412539 | . | intergenic | 3q13.2 | Score=9820;Name="3102595:L1PB1(LINE)" | bnd_135 | transl_intra_4016_0 |
| 01T | 3 | 119624746 | 119624746 | GSK3B | intronic | 3q13.33 | . | bnd_48 | del_3372 |
| 01T | 3 | 120802880 | 120802880 | STXBP5L | intronic | 3q13.33 | Score=2490;Name="3116172:L1MD2(LINE)" | bnd_50 | del_1404_0 |
| 01T | 3 | 128996821 | 128996821 | . | upstream;downstream | 3q21.3 | . | bnd_153 | transl_intra_4203_0 |
| 01T | 3 | 144484694 | 144484694 | . | intergenic | 3q24 | . | bnd_244 | transl_inter_3912 |
| 01T | 3 | 150477903 | 150477903 | SIAH2 | intronic | 3q25.1 | . | bnd_192 | tandem_dup_3569 |
| 01T | 3 | 157797965 | 157797965 | . | intergenic | 3q25.32 | Score=446;Name="3182727:L1ME4a(LINE)" | bnd_191 | tandem_dup_3569 |
| 01T | 3 | 163888747 | 163888747 | . | intergenic | 3q26.1 | Score=249;Name="3192887:L2c(LINE)" | bnd_207 | transl_inter_709 |
| 01T | 3 | 164026422 | 164026422 | . | intergenic | 3q26.1 | Score=2126;Name="3193097:THE1B(LTR)" | bnd_246 | transl_inter_2427 |
| 01T | 3 | 165543155 | 165543155 | BCHE | intronic | 3q26.1 | Score=2377;Name="3195692:L1ME2(LINE)" | bnd_47 | del_3372 |
| 01T | 3 | 169791611 | 169791611 | GPR160 | intronic | 3q26.2 | Score=1772;Name="3202437:L1ME2(LINE)" | bnd_156 | transl_intra_962_0 |
| 01T | 3 | 171547755 | 171547755 | . | intergenic | 3q26.31 | Score=251;Name="3205667:LTR88c(LTR)" | bnd_155 | transl_intra_962_0 |
| 01T | 3 | 175300154 | 175300154 | NAALADL2 | intronic | 3q26.31 | . | bnd_52 | del_4837 |
| 01T | 3 | 175328761 | 175328761 | NAALADL2 | intronic | 3q26.31 | Score=419;Name="3212077:MIRb(SINE)" | bnd_51 | del_4837 |
| 01T | 3 | 183508757 | 183508757 | YEATS2 | exonic | 3q27.1 | . | bnd_49 | del_1404_0 |
| 01T | 3 | 195599595 | 195599595 | TNK2 | intronic | 3q29 | Score=218;Name="3250882:MIRc(SINE)" | bnd_221 | transl_inter_6481 |
| 01T | 4 | 5698473 | 5698473 | EVC2 | intronic | 4p16.2 | Score=471;Name="3266873:MLT1K(LTR)" | bnd_194 | tandem_dup_4983_0 |
| 01T | 4 | 6751041 | 6751041 | . | intergenic | 4p16.1 | Score=306;Name="3269178:MIRb(SINE)" | bnd_193 | tandem_dup_4983_0 |
| 01T | 4 | 19185545 | 19185545 | . | intergenic | 4p15.31 | . | bnd_174 | transl_intra_1800_0 |
| 01T | 4 | 22129349 | 22129349 | . | intergenic | 4p15.2 | Score=23831;Name="3297764:L1PA5(LINE)" | bnd_235 | transl_inter_1579 |

| | | | | | | | | |
|---|---|---|---|---|---|---|---|---|
| 01T 4 | 30450641 | 30450641 | . | intergenic | 4p15.1 | Score=2692;Name="3313354:L1ME3A(LINE)" | bnd_54 | del_2579 |
| 01T 4 | 30461520 | 30461520 | . | intergenic | 4p15.1 | . | bnd_53 | del_2579 |
| 01T 4 | 44912270 | 44912270 | . | intergenic | 4p12 | . | bnd_158 | transl_intra_2882_0 |
| 01T 4 | 58950083 | 58950083 | . | intergenic | 4q12 | . | bnd_157 | transl_intra_2882_0 |
| 01T 4 | 67555513 | 67555513 | . | intergenic | 4q13.2 | . | bnd_173 | transl_intra_1800_0 |
| 01T 4 | 137064924 | 137064924 | . | intergenic | 4q28.3 | . | bnd_248 | transl_inter_4422 |
| 01T 4 | 142653993 | 142653993 | IL15 | exonic | 4q31.21 | . | bnd_160 | transl_intra_1980_0 |
| 01T 4 | 142654877 | 142654877 | IL15 | UTR3 | 4q31.21 | Score=1204;Name="3497826:MER30(DNA)" | bnd_159 | transl_intra_1980_0 |
| 01T 5 | 49861040 | 49861040 | . | intergenic | 5q11.1 | . | bnd_247 | transl_inter_4422 |
| 01T 5 | 64186568 | 64186568 | CWC27 | intronic | 5q12.3 | Score=270;Name="3678752:Tigger15a(DNA)" | bnd_94 | del_ins_4903 |
| 01T 5 | 64189504 | 64189504 | CWC27 | intronic | 5q12.3 | . | bnd_93 | del_ins_4903 |
| 01T 5 | 67623923 | 67623923 | . | intergenic | 5q13.1 | . | bnd_56 | del_918 |
| 01T 5 | 67633519 | 67633519 | . | intergenic | 5q13.1 | . | bnd_55 | del_918 |
| 01T 5 | 119927586 | 119927586 | PRR16 | intronic | 5q23.1 | . | bnd_58 | del_1857 |
| 01T 5 | 119928290 | 119928290 | PRR16 | intronic | 5q23.1 | . | bnd_57 | del_1857 |
| 01T 5 | 121251050 | 121251050 | . | intergenic | 5q23.1 | . | bnd_140 | del_4854_0 |
| 01T 5 | 122213707 | 122213707 | SNX24 | intronic | 5q23.2 | . | bnd_162 | transl_intra_2271_0 |
| 01T 5 | 132616563 | 132616563 | FSTL4 | intronic | 5q31.1 | . | bnd_176 | transl_intra_4169 |
| 01T 5 | 132618019 | 132618019 | FSTL4 | intronic | 5q31.1 | . | bnd_175 | transl_intra_4169 |
| 01T 5 | 133257350 | 133257350 | . | intergenic | 5q31.1 | Score=2354;Name="3792354:AluSp(SINE)" | bnd_139 | del_4854_0 |
| 01T 5 | 146908718 | 146908718 | . | intergenic | 5q32 | Score=2053;Name="3818491:AluSx(SINE)" | bnd_196 | tandem_dup_3623 |
| 01T 5 | 146920490 | 146920490 | . | intergenic | 5q32 | Score=1997;Name="3818522:AluSz(SINE)" | bnd_195 | tandem_dup_3623 |
| 01T 5 | 146957167 | 146957167 | JAKMIP2-AS1 | ncRNA_intronic | 5q32 | Score=500;Name="3818619:MIRb(SINE)" | bnd_250 | transl_inter_5266 |
| 01T 5 | 148011779 | 148011779 | HTR4 | intronic | 5q32 | . | bnd_252 | transl_inter_2850 |
| 01T 5 | 148170609 | 148170609 | . | intergenic | 5q32 | . | bnd_254 | transl_inter_3271 |
| 01T 5 | 148587750 | 148587750 | ABLIM3 | intronic | 5q32 | Score=634;Name="3821679:MLT1K(LTR)" | bnd_161 | transl_intra_2271_0 |
| 01T 6 | 6153834 | 6153834 | F13A1 | intronic | 6p25.1 | . | bnd_233 | transl_inter_6965 |
| 01T 6 | 18902508 | 18902508 | . | intergenic | 6p22.3 | . | bnd_256 | transl_inter_4551 |
| 01T 6 | 99221236 | 99221236 | . | intergenic | 6q16.1 | . | bnd_60 | del_3061 |
| 01T 6 | 99221934 | 99221934 | . | intergenic | 6q16.1 | . | bnd_59 | del_3061 |
| 01T 6 | 102843845 | 102843845 | . | intergenic | 6q16.3 | Score=827;Name="4060211:L1ME1(LINE)" | bnd_258 | transl_inter_3302 |
| 01T 6 | 148832603 | 148832603 | SASH1 | intronic | 6q24.3 | . | bnd_239 | transl_inter_5243 |
| 01T 6 | 163308601 | 163308601 | PACRG | intronic | 6q26 | . | bnd_62 | del_4008 |
| 01T 6 | 163309715 | 163309715 | PACRG | intronic | 6q26 | . | bnd_61 | del_4008 |
| 01T 7 | 820222 | 820222 | HEATR2 | intronic | 7p22.3 | Score=1370;Name="4226590:Charlie1a(DNA)" | bnd_64 | del_2711 |
| 01T 7 | 1344318 | 1344318 | . | intergenic | 7p22.3 | Score=2083;Name="4227483:AluJo(SINE)" | bnd_225 | transl_inter_1939 |
| 01T 7 | 1344740 | 1344740 | . | intergenic | 7p22.3 | Score=451;Name="4227485:L1MC4(LINE)" | bnd_231 | transl_inter_3186 |
| 01T 7 | 1344758 | 1344758 | . | intergenic | 7p22.3 | . | bnd_227 | transl_inter_903 |
| 01T 7 | 2499547 | 2499547 | . | intergenic | 7p22.3 | Score=207;Name="4229636:L2c(LINE)" | bnd_229 | transl_inter_5414 |
| 01T 7 | 8760195 | 8760195 | NXPH1 | intronic | 7p21.3 | . | bnd_63 | del_2711 |
| 01T 7 | 19386243 | 19386243 | . | intergenic | 7p21.1 | Score=1452;Name="4259349:AluJb(SINE)" | bnd_96 | del_ins_2398 |
| 01T 7 | 28438045 | 28438045 | CREB5 | intronic | 7p15.1 | . | bnd_95 | del_ins_2398 |
| 01T 7 | 44162713 | 44162713 | POLD2 | intronic | 7p13 | Score=327;Name="4302802:Charlie5(DNA)" | bnd_249 | transl_inter_5266 |
| 01T 7 | 48562637 | 48562637 | ABCA13 | intronic | 7p12.3 | Score=591;Name="4310436:L1ME3A(LINE)" | bnd_253 | transl_inter_3271 |

| | | | | | | | | |
|---|---|---|---|---|---|---|---|---|
| 01T 7 | 50921068 | 50921068 | . | intergenic | 7p12.1 | Score=1487;Name="4314040:LTR33C(LTR)" | bnd_251 | transl_inter_2850 |
| 01T 7 | 140618567 | 140618567 | BRAF | intronic | 7q34 | Score=229;Name="4474742:L2a(LINE)" | bnd_66 | del_2199 |
| 01T 7 | 140619794 | 140619794 | BRAF | intronic | 7q34 | Score=1131;Name="4474744:AluJr(SINE)" | bnd_65 | del_2199 |
| 01T 8 | 38889491 | 38889491 | ADAM9 | intronic | 8p11.22 | Score=10615;Name="4574633:L1P2(LINE)" | bnd_178 | transl_intra_844 |
| 01T 8 | 38889918 | 38889918 | ADAM9 | intronic | 8p11.22 | Score=10615;Name="4574633:L1P2(LINE)" | bnd_177 | transl_intra_844 |
| 01T 8 | 39124040 | 39124040 | ADAM32 | intronic | 8p11.22 | Score=6117;Name="4575028:L1MA5A(LINE)" | bnd_198 | tandem_dup_789_0 |
| 01T 8 | 39167278 | 39167278 | . | intergenic | 8p11.22 | . | bnd_200 | tandem_dup_3533_0 |
| 01T 8 | 39170266 | 39170266 | . | intergenic | 8p11.22 | Score=583;Name="4575093:L1M6(LINE)" | bnd_68 | del_1024 |
| 01T 8 | 39412839 | 39412839 | . | intergenic | 8p11.22 | Score=2789;Name="4575481:MSTA-int(LTR)" | bnd_67 | del_1024 |
| 01T 8 | 41474050 | 41474050 | AGPAT6 | intronic | 8p11.21 | Score=1935;Name="4579471:AluJb(SINE)" | bnd_199 | tandem_dup_3533_0 |
| 01T 8 | 41777320 | 41777320 | . | intergenic | 8p11.21 | Score=726;Name="4580023:L1ME3B(LINE)" | bnd_197 | tandem_dup_789_0 |
| 01T 8 | 53062850 | 53062850 | ST18 | intronic | 8q11.23 | Score=231;Name="4592578:(TG)n(Simple_repeat)" | bnd_70 | del_2634 |
| 01T 8 | 53064594 | 53064594 | ST18 | intronic | 8q11.23 | . | bnd_69 | del_2634 |
| 01T 8 | 55032546 | 55032546 | . | intergenic | 8q11.23 | Score=1248;Name="4595984:MLT1A(LTR)" | bnd_255 | transl_inter_4551 |
| 01T 8 | 56094870 | 56094870 | XKR4 | intronic | 8q12.1 | Score=2173;Name="4597838:L1MCb(LINE)" | bnd_257 | transl_inter_3302 |
| 01T 8 | 57082812 | 57082812 | PLAG1 | intronic | 8q12.1 | . | bnd_72 | del_1259 |
| 01T 8 | 57083623 | 57083623 | PLAG1 | intronic | 8q12.1 | . | bnd_71 | del_1259 |
| 01T 8 | 60962210 | 60962210 | . | intergenic | 8q12.1 | . | bnd_74 | del_5088 |
| 01T 8 | 60963144 | 60963144 | . | intergenic | 8q12.1 | . | bnd_73 | del_5088 |
| 01T 8 | 95649940 | 95649940 | LOC100288748 | ncRNA_exonic | 8q22.1 | Score=1618;Name="4664153:L1MC4(LINE)" | bnd_243 | transl_inter_3912 |
| 01T 8 | 100887860 | 100887860 | VPS13B | exonic | 8q22.2 | . | bnd_237 | transl_inter_2140 |
| 01T 8 | 103133818 | 103133818 | NCALD | intronic | 8q22.3 | . | bnd_245 | transl_inter_2427 |
| 01T 8 | 111107585 | 111107585 | . | intergenic | 8q23.2 | Score=326;Name="4692460:L1M5(LINE)" | bnd_76 | del_2046 |
| 01T 8 | 111108044 | 111108044 | . | intergenic | 8q23.2 | Score=1747;Name="4692461:AluJb(SINE)" | bnd_75 | del_2046 |
| 01T 8 | 112121976 | 112121976 | . | intergenic | 8q23.3 | Score=612;Name="4694174:MIR(SINE)" | bnd_98 | del_ins_2660 |
| 01T 8 | 112132979 | 112132979 | . | intergenic | 8q23.3 | . | bnd_97 | del_ins_2660 |
| 01T 8 | 120718314 | 120718314 | . | intergenic | 8q24.12 | Score=660;Name="4708807:MLT1J(LTR)" | bnd_78 | del_3635 |
| 01T 8 | 120720201 | 120720201 | . | intergenic | 8q24.12 | . | bnd_77 | del_3635 |
| 01T 9 | 2636856 | 2636856 | VLDLR | intronic | 9p24.2 | . | bnd_106 | transl_intra_3551_0 |
| 01T 9 | 2637763 | 2637763 | VLDLR | intronic | 9p24.2 | Score=2501;Name="4763408:AluY(SINE)" | bnd_100 | del_ins_2255 |
| 01T 9 | 2792341 | 2792341 | . | intergenic | 9p24.2 | Score=3011;Name="4763708:MLT2A1(LTR)" | bnd_105 | transl_intra_3551_0 |
| 01T 9 | 3374278 | 3374278 | RFX3 | intronic | 9p24.2 | Score=3751;Name="4764707:L1MB2(LINE)" | bnd_99 | del_ins_2255 |
| 01T 9 | 8369456 | 8369456 | PTPRD | intronic | 9p24.1 | . | bnd_80 | del_2507 |
| 01T 9 | 8799553 | 8799553 | PTPRD | intronic | 9p24.1 | . | bnd_79 | del_2507 |

| | | | | | | | | | |
|---|---|---|---|---|---|---|---|---|---|
| 01T | 9 | 13144938 | 13144938 | MPDZ | intronic | 9p23 | . | bnd_82 | del_2378 |
| 01T | 9 | 13227671 | 13227671 | MPDZ | intronic | 9p23 | . | bnd_81 | del_2378 |
| 01T | 9 | 20942958 | 20942958 | FOCAD | intronic | 9p21.3 | Score=452;Name="4794269:L2a(LINE)" | bnd_84 | del_4986 |
| 01T | 9 | 20989724 | 20989724 | FOCAD | intronic | 9p21.3 | . | bnd_83 | del_4986 |
| 01T | 9 | 26040680 | 26040680 | . | intergenic | 9p21.2 | Score=2407;Name="4802090:L1MEc(LINE)" | bnd_241 | transl_inter_5411 |
| 01T | 9 | 34419293 | 34419293 | FAM219A | intronic | 9p13.3 | . | bnd_130 | del_947_0 |
| 01T | 9 | 35427883 | 35427883 | ATP8B5P | ncRNA_intronic | 9p13.3 | . | bnd_202 | tandem_dup_2568 |
| 01T | 9 | 35697823 | 35697823 | TLN1 | exonic | 9p13.3 | . | bnd_201 | tandem_dup_2568 |
| 01T | 9 | 37574022 | 37574022 | FBXO10 | intronic | 9p13.2 | . | bnd_129 | del_947_0 |
| 01T | 9 | 102848909 | 102848909 | ERP44 | intronic | 9q31.1 | Score=517;Name="4904713:MIRb(SINE)" | bnd_219 | transl_inter_6467 |
| 01T | 10 | 34362789 | 34362789 | . | intergenic | 10p11.22 | Score=1973;Name="495259:AluJb(SINE)" | bnd_166 | transl_intra_2112 |
| 01T | 10 | 34364719 | 34364719 | . | intergenic | 10p11.22 | . | bnd_165 | transl_intra_2112 |
| 01T | 10 | 56535718 | 56535718 | PCDH15 | intronic | 10q21.1 | Score=3662;Name="526986:L1MA5A(LINE)" | bnd_8 | del_2581 |
| 01T | 10 | 56539331 | 56539331 | PCDH15 | intronic | 10q21.1 | . | bnd_7 | del_2581 |
| 01T | 10 | 85384195 | 85384195 | . | intergenic | 10q23.1 | . | bnd_10 | del_581 |
| 01T | 10 | 85384750 | 85384750 | . | intergenic | 10q23.1 | Score=1768;Name="581077:MLT1C(LTR)" | bnd_9 | del_581 |
| 01T | 10 | 126718188 | 126718188 | CTBP2 | intronic | 10q26.13 | . | bnd_12 | del_2382 |
| 01T | 10 | 126718759 | 126718759 | CTBP2 | intronic | 10q26.13 | . | bnd_11 | del_2382 |
| 01T | 11 | 2519449 | 2519449 | KCNQ1 | intronic | 11p15.5 | Score=22085;Name="673991:L1PA8(LINE)" | bnd_14 | del_6158 |
| 01T | 11 | 2551925 | 2551925 | KCNQ1 | intronic | 11p15.5 | Score=365;Name="674025:(TG)n(Simple_repeat)" | bnd_13 | del_6158 |
| 01T | 11 | 66029118 | 66029118 | KLC2 | intronic | 11q13.2 | . | bnd_203 | del_inss_1920_0/3209_0 |
| 01T | 11 | 66030465 | 66030465 | KLC2 | exonic | 11q13.2 | . | bnd_205 | del_inss_1920_0/3209_0 |
| 01T | 11 | 104762598 | 104762598 | CASP12 | intronic | 11q22.3 | . | bnd_16 | del_3559 |
| 01T | 11 | 104765219 | 104765219 | CASP12 | intronic | 11q22.3 | Score=1518;Name="856674:L2a(LINE)" | bnd_15 | del_3559 |
| 01T | 11 | 114401796 | 114401796 | NXPE1 | intronic | 11q23.2 | . | bnd_18 | del_4328 |
| 01T | 11 | 114402715 | 114402715 | NXPE1 | intronic | 11q23.2 | Score=253;Name="874221:MER5B(DNA)" | bnd_17 | del_4328 |
| 01T | 12 | 1337147 | 1337147 | ERC1 | intronic | 12p13.33 | Score=7356;Name="912870:L1MA9(LINE)" | bnd_180 | tandem_dup_5216 |
| 01T | 12 | 1361848 | 1361848 | ERC1 | intronic | 12p13.33 | . | bnd_179 | tandem_dup_5216 |
| 01T | 12 | 4845281 | 4845281 | GALNT8 | intronic | 12p13.32 | Score=2733;Name="919312:L1M4b(LINE)" | bnd_182 | tandem_dup_2734 |
| 01T | 12 | 5077962 | 5077962 | . | intergenic | 12p13.32 | . | bnd_181 | tandem_dup_2734 |
| 01T | 12 | 11910750 | 11910750 | ETV6 | intronic | 12p13.2 | . | bnd_20 | del_1155 |
| 01T | 12 | 11911152 | 11911152 | ETV6 | intronic | 12p13.2 | Score=227;Name="932306:MIRb(SINE)" | bnd_124 | tandem_dup_5035_0 |
| 01T | 12 | 17145776 | 17145776 | . | intergenic | 12p12.3 | . | bnd_22 | del_2668 |
| 01T | 12 | 17146221 | 17146221 | . | intergenic | 12p12.3 | . | bnd_21 | del_2668 |
| 01T | 12 | 18475950 | 18475950 | PIK3C2G | intronic | 12p12.3 | . | bnd_24 | del_2526 |
| 01T | 12 | 18476790 | 18476790 | PIK3C2G | intronic | 12p12.3 | Score=186;Name="943686:MIRc(SINE)" | bnd_23 | del_2526 |
| 01T | 12 | 24649927 | 24649927 | SOX5 | intronic | 12p12.1 | . | bnd_122 | tandem_dup_3102_0 |
| 01T | 12 | 26162861 | 26162861 | RASSF8 | intronic | 12p12.1 | Score=1404;Name="956641:L2c(LINE)" | bnd_19 | del_1155 |
| 01T | 12 | 26881923 | 26881923 | ITPR2 | intronic | 12p11.23 | Score=1386;Name="957777:L1PA13(LINE)" | bnd_168 | transl_intra_3152 |
| 01T | 12 | 26897194 | 26897194 | ITPR2 | intronic | 12p11.23 | Score=1244;Name="957811:L2a(LINE)" | bnd_167 | transl_intra_3152 |

| | | | | | | | | | |
|---|---|---|---|---|---|---|---|---|---|
| 01T | 12 | 31152445 | 31152445 | . | intergenic | 12p11.21 | Score=2026;Name="965058:MER47A(DNA)" | bnd_121 | tandem_dup_3102_0 |
| 01T | 12 | 31519249 | 31519249 | . | intergenic | 12p11.21 | Score=619;Name="965712:U6(snRNA)" | bnd_216 | transl_inter_3924 |
| 01T | 12 | 32163065 | 32163065 | . | intergenic | 12p11.21 | Score=690;Name="967483:AluJo(SINE)" | bnd_218 | transl_inter_2415 |
| 01T | 12 | 33759471 | 33759471 | . | intergenic | 12p11.1 | Score=192;Name="970562:(CAG)n(Simple_repeat)" | bnd_123 | tandem_dup_5035_0 |
| 01T | 12 | 38415929 | 38415929 | . | intergenic | 12q12 | Score=1181;Name="972186:ALR/Alpha(Satellite)" | bnd_26 | del_1960 |
| 01T | 12 | 40037246 | 40037246 | C12orf40 | intronic | 12q12 | . | bnd_25 | del_1960 |
| 01T | 12 | 40552224 | 40552224 | . | intergenic | 12q12 | . | bnd_144 | transl_intra_781_0 |
| 01T | 12 | 40559267 | 40559267 | . | intergenic | 12q12 | Score=481;Name="975573:MER5A1(DNA)" | bnd_143 | transl_intra_781_0 |
| 01T | 12 | 43605150 | 43605150 | . | intergenic | 12q12 | . | bnd_86 | del_ins_4354 |
| 01T | 12 | 43605858 | 43605858 | . | intergenic | 12q12 | . | bnd_85 | del_ins_4354 |
| 01T | 12 | 119674508 | 119674508 | . | intergenic | 12q24.23 | . | bnd_88 | del_ins_4957 |
| 01T | 12 | 119675257 | 119675257 | . | intergenic | 12q24.23 | Score=1926;Name="1129477:L1MEc(LINE)" | bnd_87 | del_ins_4957 |
| 01T | 13 | 65485797 | 65485797 | . | intergenic | 13q21.31 | Score=5985;Name="1241964:L1PREC2(LINE)" | bnd_220 | transl_inter_6467 |
| 01T | 13 | 84313176 | 84313176 | . | intergenic | 13q31.1 | . | bnd_28 | del_3137 |
| 01T | 13 | 84316363 | 84316363 | . | intergenic | 13q31.1 | Score=5811;Name="1272877:L1PA12(LINE)" | bnd_27 | del_3137 |
| 01T | 13 | 94907477 | 94907477 | GPC6 | intronic | 13q31.3 | . | bnd_211 | transl_inter_4491 |
| 01T | 14 | 54140852 | 54140852 | . | intergenic | 14q22.2 | Score=728;Name="1381465:MLT1B(LTR)" | bnd_222 | transl_inter_6481 |
| 01T | 14 | 105972571 | 105972571 | . | intergenic | 14q32.33 | Score=2355;Name="1480247:AluY(SINE)" | bnd_184 | tandem_dup_811 |
| 01T | 14 | 105977419 | 105977419 | . | intergenic | 14q32.33 | Score=1186;Name="1480266:L1MEg(LINE)" | bnd_183 | tandem_dup_811 |
| 01T | 15 | 95239012 | 95239012 | . | intergenic | 15q26.2 | . | bnd_224 | transl_inter_3248 |
| 01T | 16 | 400463 | 400463 | AXIN1 | intronic | 16p13.3 | . | bnd_30 | del_1733 |
| 01T | 16 | 400464 | 400464 | AXIN1 | intronic | 16p13.3 | . | bnd_186 | tandem_dup_2948 |
| 01T | 16 | 1465544 | 1465544 | . | upstream | 16p13.3 | Score=2124;Name="1636668:AluSx(SINE)" | bnd_146 | transl_intra_2692_0 |
| 01T | 16 | 1466430 | 1466430 | . | intergenic | 16p13.3 | Score=2040;Name="1636670:AluSp(SINE)" | bnd_132 | transl_intra_4965_0 |
| 01T | 16 | 2421140 | 2421140 | ABCA17P | ncRNA_intronic | 16p13.3 | Score=492;Name="1638260:LTR33(LTR)" | bnd_188 | tandem_dup_5027 |
| 01T | 16 | 5868771 | 5868771 | . | intergenic | 16p13.3 | . | bnd_226 | transl_inter_1939 |
| 01T | 16 | 6357925 | 6357925 | RBFOX1 | intronic | 16p13.3 | Score=385;Name="1647057:T-rich(Low_complexity)" | bnd_187 | tandem_dup_5027 |
| 01T | 16 | 8283603 | 8283603 | . | intergenic | 16p13.2 | . | bnd_125 | del_inssu_1782_0/2629_0 |
| 01T | 16 | 8342693 | 8342693 | . | intergenic | 16p13.2 | Score=4685;Name="1651129:L1MA8(LINE)" | bnd_190 | tandem_dup_1360_0 |
| 01T | 16 | 8370280 | 8370280 | . | intergenic | 16p13.2 | . | bnd_185 | tandem_dup_2948 |
| 01T | 16 | 8559113 | 8559113 | . | intergenic | 16p13.2 | Score=4227;Name="1651639:L1PA16(LINE)" | bnd_170 | transl_intra_2513_0 |
| 01T | 16 | 15920523 | 15920523 | MYH11 | intronic | 16p13.11 | . | bnd_169 | transl_intra_2513_0 |
| 01T | 16 | 15920524 | 15920524 | MYH11 | intronic | 16p13.11 | . | bnd_189 | tandem_dup_1360_0 |
| 01T | 16 | 16131631 | 16131631 | ABCC1 | intronic | 16p13.11 | . | bnd_29 | del_1733 |
| 01T | 16 | 17071100 | 17071100 | . | intergenic | 16p12.3 | Score=2240;Name="1673290:AluSx(SINE)" | bnd_127 | del_inssu_1782_0/2629_0 |

| | | | | | | | | | |
|---|---|---|---|---|---|---|---|---|---|
| 01T | 16 | 21172093 | 21172093 | TMEM159 | intronic | 16p12.3 | Score=450;Name="1683871:L1MC(LINE)" | bnd_120 | del_4104_0 |
| 01T | 16 | 23359463 | 23359463 | SCNN1B | intronic | 16p12.2 | Score=2975;Name="1689330:MER6A(DNA)" | bnd_112 | transl_intra_1037_0 |
| 01T | 16 | 24747456 | 24747456 | TNRC6A | intronic | 16p12.1 | Score=2264;Name="1693279:AluSq2(SINE)" | bnd_119 | del_4104_0 |
| 01T | 16 | 66302383 | 66302383 | . | intergenic | 16q21 | Score=688;Name="1755420:MIRb(SINE)" | bnd_126 | del_inssu_1782_0/2629_0 |
| 01T | 16 | 66439978 | 66439978 | . | intergenic | 16q21 | . | bnd_228 | transl_inter_903 |
| 01T | 16 | 66444462 | 66444462 | LINC00920 | ncRNA_exonic | 16q21 | Score=679;Name="1755734:MLT1F2(LTR)" | bnd_131 | transl_intra_4965_0 |
| 01T | 16 | 66445084 | 66445084 | . | downstream;downstrea | 16q21 | . | bnd_32 | del_3065 |
| 01T | 16 | 66445084 | 66445084 | . | downstream;downstrea | 16q21 | . | bnd_128 | del_inssu_1782_0/2629_0 |
| 01T | 16 | 69598054 | 69598054 | . | intergenic;intergenic | 16q22.1 | . | bnd_31 | del_3065 |
| 01T | 16 | 69598054 | 69598054 | . | intergenic;intergenic | 16q22.1 | . | bnd_111 | transl_intra_1037_0 |
| 01T | 16 | 69604817 | 69604817 | NFAT5 | intronic | 16q22.1 | Score=608;Name="1763112:MIR(SINE)" | bnd_230 | transl_inter_5414 |
| 01T | 16 | 69604823 | 69604823 | NFAT5 | intronic | 16q22.1 | Score=608;Name="1763112:MIR(SINE)" | bnd_232 | transl_inter_3186 |
| 01T | 16 | 23652456 | 23652456 | PALB2 | exonic | 16p12.2 | . | bnd_145 | transl_intra_2692_0 |
| 01T | 18 | 13598292 | 13598292 | LDLRAD4 | intronic | 18p11.21 | Score=11584;Name="1995990:L1M4b(LINE)" | bnd_234 | transl_inter_6965 |
| 01T | 18 | 34920112 | 34920112 | CELF4 | intronic | 18q12.2 | . | bnd_90 | del_ins_2522 |
| 01T | 18 | 34921003 | 34921003 | CELF4 | intronic | 18q12.2 | . | bnd_89 | del_ins_2522 |
| 01T | 18 | 36648849 | 36648849 | . | intergenic | 18q12.2 | . | bnd_213 | transl_inter_1440 |
| 01T | 19 | 6692351 | 6692351 | C3 | intronic | 19p13.3 | Score=346;Name="2115125:MER5A(DNA)" | bnd_34 | del_4705 |
| 01T | 19 | 6726758 | 6726758 | . | intergenic | 19p13.3 | Score=2409;Name="2115210:AluSx1(SINE)" | bnd_33 | del_4705 |
| 01T | 19 | 15298671 | 15298671 | NOTCH3 | intronic | 19p13.12 | . | bnd_172 | transl_intra_1559 |
| 01T | 19 | 15300757 | 15300757 | NOTCH3 | intronic | 19p13.12 | Score=311;Name="2138852:L1ME2z(LINE)" | bnd_217 | transl_inter_2415 |
| 01T | 19 | 15372510 | 15372510 | BRD4 | intronic | 19p13.12 | Score=1142;Name="2139019:AluJb(SINE)" | bnd_171 | transl_intra_1559 |
| 01T | 19 | 19504544 | 19504544 | GATAD2A | intronic | 19p13.11 | . | bnd_148 | transl_intra_983_0 |
| 01T | 19 | 19507484 | 19507484 | GATAD2A | intronic | 19p13.11 | Score=490;Name="2149782:AluSx(SINE)" | bnd_147 | transl_intra_983_0 |
| 01T | 19 | 19752421 | 19752421 | GMIP | intronic | 19p13.11 | Score=1470;Name="2150261:AluJr(SINE)" | bnd_150 | transl_intra_1653_0 |
| 01T | 19 | 19753255 | 19753255 | GMIP | intronic | 19p13.11 | . | bnd_149 | transl_intra_1653_0 |
| 01T | 19 | 23033203 | 23033203 | . | intergenic | 19p12 | Score=2145;Name="2156471:AluSq2(SINE)" | bnd_215 | transl_inter_3924 |
| 01T | 20 | 60945269 | 60945269 | . | intergenic | 20q13.33 | . | bnd_92 | del_ins_1825 |
| 01T | 20 | 60947180 | 60947180 | . | intergenic | 20q13.33 | . | bnd_91 | del_ins_1825 |
| 01T | 21 | 23889197 | 23889197 | . | intergenic | 21q21.1 | Score=2311;Name="2790144:LTR77(LTR)" | bnd_134 | transl_intra_3016_0 |
| 01T | 21 | 25326067 | 25326067 | . | intergenic | 21q21.2 | . | bnd_133 | transl_intra_3016_0 |
| 01T | 21 | 35169020 | 35169020 | ITSN1 | intronic | 21q22.11 | Score=580;Name="2810747:MIR(SINE)" | bnd_118 | transl_intra_2021_0 |
| 01T | 21 | 35170335 | 35170335 | ITSN1 | intronic | 21q22.11 | . | bnd_117 | transl_intra_2021_0 |
| 01T | 21 | 35173396 | 35173396 | ITSN1 | intronic | 21q22.11 | Score=1574;Name="2810750:AluJb(SINE)" | bnd_236 | transl_inter_1579 |

| | | | | | | | | |
|---|---|---|---|---|---|---|---|---|
| 01T 21 | 48031142 | 48031142 | . | intergenic | 21q22.3 | Score=2695;Name="2832833:AluY(SINE)" | bnd_238 | transl_inter_2140 |
| 01T X | 105936623 | 105936623 | . | upstream | Xq22.3 | Score=351;Name="5168672:(CA)n(Simple_repeat)" | bnd_209 | transl_inter_6524 |
| 02T 2 | 212606049 | 212606049 | ERBB4 | intronic | 2q34 | . | bnd_6 | transl_intra_2202_0 |
| 02T 2 | 212606295 | 212606295 | ERBB4 | intronic | 2q34 | Score=183;Name="2594340:MIR3(SINE)" | bnd_5 | transl_intra_2202_0 |
| 02T 4 | 87813095 | 87813095 | C4orf36 | intronic | 4q21.3 | . | bnd_10 | transl_intra_2033_0 |
| 02T 4 | 123934202 | 123934202 | SPATA5 | intronic | 4q28.1 | Score=2596;Name="3467040:LTR7(LTR)" | bnd_9 | transl_intra_2033_0 |
| 02T 7 | 2172708 | 2172708 | MAD1L1 | intronic | 7p22.3 | Score=334;Name="4228905:L1MC2(LINE)" | bnd_12 | tandem_dup_1249_0 |
| 02T 7 | 96347951 | 96347951 | . | intergenic | 7q21.3 | . | bnd_11 | tandem_dup_1249_0 |
| 02T 10 | 89676709 | 89676709 | PTEN | intronic | 10q23.31 | . | bnd_4 | transl_intra_544_0 |
| 02T 10 | 89677049 | 89677049 | PTEN | intronic | 10q23.31 | Score=444;Name="588733:L1MC4a(LINE)" | bnd_3 | transl_intra_544_0 |
| 02T 13 | 114195516 | 114195516 | TMCO3 | intronic | 13q34 | Score=4544;Name="1321234:L1PA15(LINE)" | bnd_2 | del_5680 |
| 02T 13 | 114195644 | 114195644 | TMCO3 | intronic | 13q34 | . | bnd_1 | del_5680 |
| 02T 20 | 33866877 | 33866877 | EIF6 | UTR3 | 20q11.22 | . | bnd_8 | transl_intra_1402 |
| 02T 20 | 33867221 | 33867221 | EIF6 | intronic | 20q11.22 | . | bnd_7 | transl_intra_1402 |
| 04T 1 | 9335470 | 9335470 | . | intergenic | 1p36.22 | . | bnd_14 | tandem_dup_1079_0 |
| 04T 1 | 9593191 | 9593191 | . | intergenic | 1p36.22 | . | bnd_13 | tandem_dup_1079_0 |
| 04T 2 | 32825253 | 32825253 | BIRC6 | intronic | 2p22.3 | . | bnd_2 | del_3366 |
| 04T 2 | 32871367 | 32871367 | TTC27 | intronic | 2p22.3 | Score=2320;Name="2292701:AluY(SINE)" | bnd_1 | del_3366 |
| 04T 2 | 237835286 | 237835286 | . | intergenic | 2q37.3 | Score=209;Name="2640323:L4(LINE)" | bnd_4 | del_1697 |
| 04T 2 | 237837432 | 237837432 | . | intergenic | 2q37.3 | Score=3724;Name="2640325:LTR73(LTR)" | bnd_3 | del_1697 |
| 04T 3 | 23523004 | 23523004 | MIR548AC,UBE2E2 | ncRNA_intronic | 3p24.3 | . | bnd_8 | del_930 |
| 04T 3 | 23569544 | 23569544 | UBE2E2,MIR548AC | ncRNA_intronic | 3p24.3 | Score=3408;Name="2952143:MER21C(LTR)" | bnd_7 | del_930 |
| 04T 3 | 177969338 | 177969338 | . | intergenic | 3q26.32 | . | bnd_10 | del_1316 |
| 04T 3 | 177970354 | 177970354 | . | intergenic | 3q26.32 | Score=1131;Name="3216671:L1ME3B(LINE)" | bnd_9 | del_1316 |
| 04T 5 | 871423 | 871423 | BRD9 | intronic | 5p15.33 | . | bnd_21 | transl_inter_1164 |
| 04T 5 | 42707699 | 42707699 | GHR | intronic | 5p12 | Score=1848;Name="3649024:L1P4(LINE)" | bnd_19 | transl_inter_2304 |
| 04T 5 | 1295615 | 1295615 | . | upstream | 5p15.33 | . | bnd_23 | transl_inter_2725 |
| 04T 6 | 96059228 | 96059228 | . | intergenic | 6q16.1 | . | bnd_26 | transl_inter_6369 |
| 04T 7 | 142994708 | 142994708 | CASP2 | intronic | 7q34 | Score=2524;Name="4478449:AluSq2(SINE)" | bnd_16 | tandem_dup_2455 |
| 04T 7 | 143117989 | 143117989 | EPHA1-AS1 | ncRNA_intronic | 7q35 | Score=2456;Name="4478635:THE1B(LTR)" | bnd_15 | tandem_dup_2455 |
| 04T 9 | 6828911 | 6828911 | KDM4C | intronic | 9p24.1 | Score=206;Name="4771013:L3(LINE)" | bnd_25 | transl_inter_6369 |
| 04T 10 | 94737085 | 94737085 | EXOC6 | intronic | 10q23.33 | . | bnd_18 | transl_inter_801 |
| 04T 10 | 95243583 | 95243583 | . | intergenic | 10q23.33 | . | bnd_20 | transl_inter_2304 |
| 04T 11 | 57056501 | 57056501 | . | intergenic | 11q12.1 | Score=201;Name="764757:(TG)n(Simple_repeat)" | bnd_17 | transl_inter_801 |
| 04T 11 | 57058352 | 57058352 | . | intergenic | 11q12.1 | . | bnd_22 | transl_inter_1164 |
| 04T 11 | 57530247 | 57530247 | CTNND1,TMX2-CTNND1 | ncRNA_intronic | 11q12.1 | . | bnd_24 | transl_inter_2725 |
| 04T 19 | 8292579 | 8292579 | CERS4 | intronic | 19p13.2 | Score=1075;Name="2119587:L1MB3(LINE)" | bnd_12 | transl_intra_1394_0 |

| | | | | | | | | | |
|---|---|---|---|---|---|---|---|---|---|
| 04T | 19 | 8301534 | 8301534 | CERS4 | intronic | 19p13.2 | Score=1950;Name="2119623:AluJb(SINE)" | bnd_11 | transl_intra_1394_0 |
| 04T | 20 | 14768954 | 14768954 | MACROD2 | intronic | 20p12.1 | Score=1827;Name="2677355:AluJb(SINE)" | bnd_6 | del_4518 |
| 04T | 20 | 14769707 | 14769707 | MACROD2 | intronic | 20p12.1 | Score=2448;Name="2677356:AluSx1(SINE)" | bnd_5 | del_4518 |
| 05T | 3 | 27149719 | 27149719 | . | intergenic | 3p24.1 | . | bnd_12 | transl_intra_1052_0 |
| 05T | 3 | 27150382 | 27150382 | . | intergenic | 3p24.1 | . | bnd_10 | transl_intra_3582_0 |
| 05T | 3 | 67555115 | 67555115 | SUCLG2 | intronic | 3p14.1 | Score=338;Name="3033941:MER20(DNA)" | bnd_11 | transl_intra_1052_0 |
| 05T | 3 | 67555461 | 67555461 | SUCLG2 | intronic | 3p14.1 | . | bnd_9 | transl_intra_3582_0 |
| 05T | 5 | 141129047 | 141129047 | . | intergenic | 5q31.3 | . | bnd_2 | del_686 |
| 05T | 5 | 141147352 | 141147352 | . | intergenic | 5q31.3 | . | bnd_1 | del_686 |
| 05T | 5 | 141203181 | 141203181 | . | intergenic | 5q31.3 | . | bnd_4 | del_1454 |
| 05T | 5 | 141209232 | 141209232 | . | intergenic | 5q31.3 | . | bnd_3 | del_1454 |
| 05T | 6 | 24150934 | 24150934 | . | intergenic | 6p22.3 | Score=1948;Name="3930606:AluSz(SINE)" | bnd_6 | del_1833 |
| 05T | 6 | 24151713 | 24151713 | . | intergenic | 6p22.3 | Score=2101;Name="3930610:L1PA7(LINE)" | bnd_5 | del_1833 |
| 05T | 8 | 14811424 | 14811424 | SGCZ | intronic | 8p22 | . | bnd_8 | del_2341 |
| 05T | 8 | 14816863 | 14816863 | SGCZ | intronic | 8p22 | . | bnd_7 | del_2341 |
| 08T | 1 | 31413474 | 31413474 | PUM1 | intronic | 1p35.2 | . | bnd_122 | transl_inter_1901 |
| 08T | 1 | 43851043 | 43851043 | MED8 | intronic | 1p34.2 | . | bnd_80 | tandem_dup_1641 |
| 08T | 1 | 44686029 | 44686029 | DMAP1 | intronic | 1p34.1 | . | bnd_79 | tandem_dup_1641 |
| 08T | 1 | 72978785 | 72978785 | . | intergenic | 1p31.1 | . | bnd_2 | del_1614 |
| 08T | 1 | 73138105 | 73138105 | . | intergenic | 1p31.1 | . | bnd_47 | del_inssu_4582_0/2990_0 |
| 08T | 1 | 73138136 | 73138136 | . | intergenic | 1p31.1 | . | bnd_1 | del_1614 |
| 08T | 1 | 73213195 | 73213195 | . | intergenic | 1p31.1 | . | bnd_60 | transl_intra_4742_0 |
| 08T | 1 | 73391690 | 73391690 | . | intergenic | 1p31.1 | . | bnd_124 | transl_inter_6105 |
| 08T | 1 | 73391692 | 73391692 | . | intergenic | 1p31.1 | . | bnd_4 | del_1567 |
| 08T | 1 | 73816497 | 73816497 | . | intergenic | 1p31.1 | . | bnd_62 | transl_intra_2094_0 |
| 08T | 1 | 73855698 | 73855698 | . | intergenic | 1p31.1 | Score=399;Name="160168:L1MC(LINE)" | bnd_72 | transl_intra_4336_0 |
| 08T | 1 | 74881302 | 74881302 | TNNI3K,FPGT-TNNI3K | intronic | 1p31.1 | . | bnd_61 | transl_intra_2094_0 |
| 08T | 1 | 74882264 | 74882264 | TNNI3K,FPGT-TNNI3K | intronic | 1p31.1 | Score=437;Name="161805:L1M6(LINE)" | bnd_44 | transl_intra_3471_0 |
| 08T | 1 | 74888260 | 74888260 | TNNI3K,FPGT-TNNI3K | intronic | 1p31.1 | . | bnd_126 | transl_inter_2493 |
| 08T | 1 | 74892084 | 74892084 | FPGT-TNNI3K,TNNI3K | intronic | 1p31.1 | . | bnd_38 | transl_intra_2354_0 |
| 08T | 1 | 75222255 | 75222255 | TYW3 | intronic | 1p31.1 | Score=12086;Name="162386:THE1B-int(LTR)" | bnd_6 | del_4696_0 |
| 08T | 1 | 76967045 | 76967045 | ST6GALNAC3 | intronic | 1p31.1 | . | bnd_128 | transl_inter_483 |
| 08T | 1 | 77873840 | 77873840 | AK5 | intronic | 1p31.1 | Score=6053;Name="166881:L1PA16(LINE)" | bnd_82 | tandem_dup_2687_0 |
| 08T | 1 | 77892337 | 77892337 | AK5 | intronic | 1p31.1 | Score=8716;Name="166915:L1MCa(LINE)" | bnd_130 | transl_inter_2907 |
| 08T | 1 | 79275012 | 79275012 | . | intergenic | 1p31.1 | Score=5853;Name="169518:L1P1(LINE)" | bnd_49 | del_inssu_4582_0/2990_0 |
| 08T | 1 | 80219196 | 80219196 | . | intergenic | 1p31.1 | . | bnd_132 | transl_inter_2822 |
| 08T | 1 | 80241500 | 80241500 | . | intergenic | 1p31.1 | . | bnd_81 | tandem_dup_2687_0 |
| 08T | 1 | 80243598 | 80243598 | . | intergenic | 1p31.1 | . | bnd_43 | transl_intra_3471_0 |
| 08T | 1 | 80350258 | 80350258 | . | intergenic | 1p31.1 | Score=10760;Name="171242:THE1B-int(LTR)" | bnd_3 | del_1567 |
| 08T | 1 | 80350484 | 80350484 | . | intergenic | 1p31.1 | Score=10760;Name="171242:THE1B-int(LTR)" | bnd_134 | transl_inter_3306 |
| 08T | 1 | 80352761 | 80352761 | . | intergenic | 1p31.1 | . | bnd_136 | transl_inter_3394 |
| 08T | 1 | 80352812 | 80352812 | . | intergenic | 1p31.1 | . | bnd_138 | transl_inter_4055 |
| 08T | 1 | 80369208 | 80369208 | . | intergenic | 1p31.1 | . | bnd_8 | del_900 |

| | | | | | | | | |
|---|---|---|---|---|---|---|---|---|
| 08T 1 | 80370426 | 80370426 | . | intergenic | 1p31.1 | Score=1934;Name="171258:THE1B(LTR)" | bnd_7 | del_900 |
| 08T 1 | 80489589 | 80489589 | . | intergenic | 1p31.1 | . | bnd_37 | transl_intra_2354_0 |
| 08T 1 | 80489680 | 80489680 | . | intergenic | 1p31.1 | . | bnd_5 | del_4696_0 |
| 08T 1 | 80500032 | 80500032 | . | intergenic | 1p31.1 | Score=3042;Name="171491:L1MB3(LINE)" | bnd_48 | del_inssu_4582_0/2990_0 |
| 08T 1 | 80500083 | 80500083 | . | intergenic | 1p31.1 | Score=3042;Name="171491:L1MB3(LINE)" | bnd_50 | del_inssu_4582_0/2990_0 |
| 08T 1 | 80536529 | 80536529 | . | intergenic | 1p31.1 | Score=214;Name="171567:L2c(LINE)" | bnd_59 | transl_intra_4742_0 |
| 08T 1 | 112011952 | 112011952 | . | intergenic | 1p13.2 | . | bnd_71 | transl_intra_4336_0 |
| 08T 1 | 175328450 | 175328450 | TNR | intronic | 1q25.1 | Score=2276;Name="300389:AluSz(SINE)" | bnd_84 | tandem_dup_748 |
| 08T 1 | 175339045 | 175339045 | TNR | intronic | 1q25.1 | . | bnd_83 | tandem_dup_748 |
| 08T 1 | 191149336 | 191149336 | . | intergenic | 1q31.2 | Score=30;Name="328122:AT_rich(Low_complexity)" | bnd_36 | del_ins_2369 |
| 08T 1 | 191157181 | 191157181 | . | intergenic | 1q31.2 | . | bnd_35 | del_ins_2369 |
| 08T 1 | 195684338 | 195684338 | . | intergenic | 1q31.3 | Score=23;Name="335752:AT_rich(Low_complexity)" | bnd_86 | tandem_dup_3559 |
| 08T 1 | 195703001 | 195703001 | . | intergenic | 1q31.3 | Score=6175;Name="335788:MER50-int(LTR)" | bnd_85 | tandem_dup_3559 |
| 08T 2 | 941414 | 941414 | . | intergenic | 2p25.3 | Score=3213;Name="2235280:L1M2(LINE)" | bnd_22 | del_1344 |
| 08T 2 | 1024113 | 1024113 | SNTG2 | intronic | 2p25.3 | . | bnd_21 | del_1344 |
| 08T 2 | 150291520 | 150291520 | LYPD6 | intronic | 2q23.2 | Score=1628;Name="2490092:MLT1B(LTR)" | bnd_102 | tandem_dup_615 |
| 08T 2 | 150310233 | 150310233 | LYPD6 | intronic | 2q23.2 | Score=294;Name="2490119:L1MEg(LINE)" | bnd_101 | tandem_dup_615 |
| 08T 2 | 162086053 | 162086053 | TANK | intronic | 2q24.2 | Score=617;Name="2509194:L2c(LINE)" | bnd_104 | tandem_dup_1490 |
| 08T 2 | 162163020 | 162163020 | . | intergenic | 2q24.2 | Score=278;Name="2509328:MADE1(DNA)" | bnd_103 | tandem_dup_1490 |
| 08T 3 | 84591319 | 84591319 | . | intergenic | 3p12.1 | Score=867;Name="3063303:MSTB2(LTR)" | bnd_140 | transl_inter_2474 |
| 08T 4 | 46472218 | 46472218 | . | intergenic | 4p12 | . | bnd_68 | transl_intra_2748_0 |
| 08T 4 | 46473507 | 46473507 | . | intergenic | 4p12 | . | bnd_70 | transl_intra_3039_0 |
| 08T 4 | 46477015 | 46477015 | . | intergenic | 4p12 | . | bnd_139 | transl_inter_2474 |
| 08T 4 | 48368066 | 48368066 | SLAIN2 | intronic | 4p11 | Score=2864;Name="3345946:L1MEc(LINE)" | bnd_51 | del_inssu_2107_0/5743_0 |
| 08T 4 | 48368402 | 48368402 | SLAIN2 | intronic | 4p11 | Score=2864;Name="3345946:L1MEc(LINE)" | bnd_69 | transl_intra_3039_0 |
| 08T 4 | 48368755 | 48368755 | SLAIN2 | intronic | 4p11 | . | bnd_53 | del_inssu_2107_0/5743_0 |
| 08T 4 | 48369050 | 48369050 | SLAIN2 | intronic | 4p11 | . | bnd_52 | del_inssu_2107_0/5743_0 |
| 08T 4 | 52966286 | 52966286 | . | intergenic | 4q12 | . | bnd_76 | transl_intra_4679_0 |
| 08T 4 | 52994445 | 52994445 | . | intergenic | 4q12 | . | bnd_54 | del_inssu_2107_0/5743_0 |
| 08T 4 | 52994636 | 52994636 | . | intergenic | 4q12 | . | bnd_67 | transl_intra_2748_0 |
| 08T 4 | 52994838 | 52994838 | . | intergenic | 4q12 | . | bnd_75 | transl_intra_4679_0 |
| 08T 5 | 16777626 | 16777626 | MYO10 | intronic | 5p15.1 | . | bnd_40 | del_insod_2982_0/1743_0 |
| 08T 5 | 16777823 | 16777823 | MYO10 | intronic | 5p15.1 | Score=333;Name="3604225:MIR(SINE)" | bnd_42 | del_insod_2982_0/1743_0 |
| 08T 5 | 78060693 | 78060693 | . | intergenic | 5q14.1 | . | bnd_108 | tandem_dup_4730 |
| 08T 5 | 78089002 | 78089002 | ARSB | intronic | 5q14.1 | . | bnd_107 | tandem_dup_4730 |
| 08T 5 | 88438672 | 88438672 | . | intergenic | 5q14.3 | . | bnd_110 | tandem_dup_2008 |
| 08T 5 | 88457892 | 88457892 | . | intergenic | 5q14.3 | Score=17608;Name="3721654:L1PA7(LINE)" | bnd_109 | tandem_dup_2008 |
| 08T 5 | 110115732 | 110115732 | . | intergenic | 5q22.1 | Score=4809;Name="3755079:L1PA6(LINE)" | bnd_24 | del_2038 |

| | | | | | | | | | |
|---|---|---|---|---|---|---|---|---|---|
| 08T | 5 | 110225318 | 110225318 | . | intergenic | 5q22.1 | Score=21401;Name="3755241:L1PA7(LINE)" | bnd_41 | del_insod_2982_0/1743_0 |
| 08T | 5 | 110287043 | 110287043 | . | intergenic | 5q22.1 | Score=192;Name="3755293:MIR3(SINE)" | bnd_39 | del_insod_2982_0/1743_0 |
| 08T | 5 | 110355302 | 110355302 | . | intergenic | 5q22.1 | . | bnd_23 | del_2038 |
| 08T | 5 | 116009659 | 116009659 | . | intergenic | 5q23.1 | . | bnd_112 | tandem_dup_2601 |
| 08T | 5 | 116040866 | 116040866 | . | intergenic | 5q23.1 | Score=27874;Name="3764417:Harlequin-int(LTR)" | bnd_111 | tandem_dup_2601 |
| 08T | 5 | 127473696 | 127473696 | SLC12A2 | intronic | 5q23.3 | Score=1824;Name="3782764:AluJb(SINE)" | bnd_26 | del_3003 |
| 08T | 5 | 127496233 | 127496233 | SLC12A2 | intronic | 5q23.3 | . | bnd_25 | del_3003 |
| 08T | 5 | 131314257 | 131314257 | ACSL6 | intronic | 5q31.1 | Score=1583;Name="3789004:LTR78(LTR)" | bnd_114 | tandem_dup_1275 |
| 08T | 5 | 131341567 | 131341567 | ACSL6 | intronic | 5q31.1 | . | bnd_113 | tandem_dup_1275 |
| 08T | 6 | 24165318 | 24165318 | . | intergenic | 6p22.3 | Score=1004;Name="3930637:ERV3-16A3_I-int(LTR)" | bnd_116 | tandem_dup_5506 |
| 08T | 6 | 24416631 | 24416631 | MRS2 | exonic | 6p22.3 | . | bnd_115 | tandem_dup_5506 |
| 08T | 6 | 162113416 | 162113416 | PARK2 | intronic | 6q26 | . | bnd_58 | insod_5752_0/1740_0 |
| 08T | 6 | 162113500 | 162113500 | PARK2 | intronic | 6q26 | . | bnd_56 | insod_5752_0/1740_0 |
| 08T | 6 | 162241008 | 162241008 | PARK2 | intronic | 6q26 | . | bnd_57 | insod_5752_0/1740_0 |
| 08T | 6 | 162241516 | 162241516 | PARK2 | intronic | 6q26 | Score=89;Name="4161583:AT_rich(Low_complexity)" | bnd_55 | insod_5752_0/1740_0 |
| 08T | 7 | 66397599 | 66397599 | TMEM248 | intronic | 7q11.21 | Score=2293;Name="4336266:AluSx(SINE)" | bnd_28 | del_3851 |
| 08T | 7 | 66397945 | 66397945 | TMEM248 | intronic | 7q11.21 | Score=2013;Name="4336267:AluSx3(SINE)" | bnd_27 | del_3851 |
| 08T | 7 | 92819941 | 92819941 | HEPACAM2 | intronic | 7q21.3 | . | bnd_30 | del_2752 |
| 08T | 7 | 92855708 | 92855708 | HEPACAM2 | exonic | 7q21.3 | . | bnd_29 | del_2752 |
| 08T | 8 | 106947674 | 106947674 | . | intergenic | 8q23.1 | Score=2016;Name="4685460:AluSz6(SINE)" | bnd_32 | del_4638 |
| 08T | 8 | 106950307 | 106950307 | . | intergenic | 8q23.1 | . | bnd_31 | del_4638 |
| 08T | 9 | 23159948 | 23159948 | . | intergenic | 9p21.3 | . | bnd_78 | transl_intra_4445 |
| 08T | 9 | 25622215 | 25622215 | . | intergenic | 9p21.2 | Score=564;Name="4801443:(TA)n(Simple_repeat)" | bnd_77 | transl_intra_4445 |
| 08T | 9 | 28273673 | 28273673 | LINGO2 | intronic | 9p21.1 | . | bnd_118 | tandem_dup_2000 |
| 08T | 9 | 28296177 | 28296177 | LINGO2 | intronic | 9p21.1 | . | bnd_117 | tandem_dup_2000 |
| 08T | 10 | 88353104 | 88353104 | . | intergenic | 10q23.2 | . | bnd_88 | tandem_dup_691 |
| 08T | 10 | 88615508 | 88615508 | BMPR1A | intronic | 10q23.2 | . | bnd_87 | tandem_dup_691 |
| 08T | 11 | 1734978 | 1734978 | MOB2 | intronic | 11p15.5 | Score=2207;Name="672910:L1ME1(LINE)" | bnd_74 | transl_intra_3247_0 |
| 08T | 11 | 6796197 | 6796197 | . | intergenic | 11p15.4 | Score=7553;Name="680975:L1MA8(LINE)" | bnd_73 | transl_intra_3247_0 |
| 08T | 11 | 24716584 | 24716584 | LUZP2 | intronic | 11p14.3 | Score=4423;Name="713933:L1PA8(LINE)" | bnd_10 | del_3170 |
| 08T | 11 | 24717913 | 24717913 | LUZP2 | intronic | 11p14.3 | . | bnd_9 | del_3170 |
| 08T | 11 | 24719434 | 24719434 | LUZP2 | intronic | 11p14.3 | Score=1908;Name="713938:LTR8A(LTR)" | bnd_12 | del_1642 |
| 08T | 11 | 24722554 | 24722554 | LUZP2 | intronic | 11p14.3 | . | bnd_11 | del_1642 |
| 08T | 11 | 33636088 | 33636088 | KIAA1549L | intronic | 11p13 | Score=486;Name="730075:(TA)n(Simple_repeat)" | bnd_14 | del_1736 |
| 08T | 11 | 37693847 | 37693847 | . | intergenic | 11p12 | . | bnd_13 | del_1736 |
| 08T | 11 | 103599470 | 103599470 | . | intergenic | 11q22.3 | . | bnd_64 | transl_intra_1584_0 |
| 08T | 11 | 103980846 | 103980846 | PDGFD | intronic | 11q22.3 | . | bnd_63 | transl_intra_1584_0 |
| 08T | 12 | 7530749 | 7530749 | CD163L1 | intronic | 12p13.31 | Score=999;Name="924196:L2c(LINE)" | bnd_90 | tandem_dup_3681 |
| 08T | 12 | 7643444 | 7643444 | CD163 | intronic | 12p13.31 | . | bnd_89 | tandem_dup_3681 |
| 08T | 12 | 117163038 | 117163038 | C12orf49 | intronic | 12q24.22 | Score=1316;Name="1123093:Tigger7(DNA)" | bnd_16 | del_2733 |

| | | | | | | | | |
|---|---|---|---|---|---|---|---|---|
| 08T 12 | 117181359 | 117181359 | RNFT2 | intronic | 12q24.22 | Score=2058;Name="1123149:AluSx(SINE)" | bnd_15 | del_2733 |
| 08T 13 | 47441838 | 47441838 | HTR2A | intronic | 13q14.2 | Score=1008;Name="1211557:LTR40b(LTR)" | bnd_92 | tandem_dup_1018 |
| 08T 13 | 47478175 | 47478175 | . | intergenic | 13q14.2 | . | bnd_91 | tandem_dup_1018 |
| 08T 14 | 25343080 | 25343080 | STXBP6 | intronic | 14q12 | . | bnd_127 | transl_inter_483 |
| 08T 14 | 32273417 | 32273417 | NUBPL | intronic | 14q12 | . | bnd_131 | transl_inter_2822 |
| 08T 14 | 44042771 | 44042771 | . | intergenic | 14q21.2 | Score=243;Name="1364136:L2(LINE)" | bnd_135 | transl_inter_3394 |
| 08T 14 | 44153909 | 44153909 | . | intergenic | 14q21.2 | . | bnd_137 | transl_inter_4055 |
| 08T 14 | 44180331 | 44180331 | . | intergenic | 14q21.2 | . | bnd_125 | transl_inter_2493 |
| 08T 14 | 46184484 | 46184484 | . | intergenic | 14q21.2 | Score=712;Name="1367656:HERVK14-int(LTR)" | bnd_123 | transl_inter_6105 |
| 08T 14 | 46184596 | 46184596 | . | intergenic | 14q21.2 | Score=712;Name="1367656:HERVK14-int(LTR)" | bnd_129 | transl_inter_2907 |
| 08T 14 | 46318088 | 46318088 | . | intergenic | 14q21.2 | . | bnd_133 | transl_inter_3306 |
| 08T 14 | 46319182 | 46319182 | . | intergenic | 14q21.2 | . | bnd_121 | transl_inter_1901 |
| 08T 14 | 47375721 | 47375721 | MDGA2 | intronic | 14q21.3 | . | bnd_94 | tandem_dup_3289 |
| 08T 14 | 47426614 | 47426614 | MDGA2 | exonic | 14q21.3 | . | bnd_93 | tandem_dup_3289 |
| 08T 14 | 94251342 | 94251342 | PRIMA1 | intronic | 14q32.12 | . | bnd_18 | del_4673 |
| 08T 14 | 94251789 | 94251789 | PRIMA1 | intronic | 14q32.12 | . | bnd_17 | del_4673 |
| 08T 15 | 37198532 | 37198532 | MEIS2 | intronic | 15q14 | . | bnd_20 | del_4297 |
| 08T 15 | 37383323 | 37383323 | MEIS2 | intronic | 15q14 | . | bnd_19 | del_4297 |
| 08T 15 | 69686524 | 69686524 | PAQR5 | intronic | 15q23 | . | bnd_46 | transl_intra_3715_0 |
| 08T 15 | 69686679 | 69686679 | PAQR5 | intronic | 15q23 | . | bnd_45 | transl_intra_3715_0 |
| 08T 15 | 100137672 | 100137672 | MEF2A | intronic | 15q26.3 | Score=7533;Name="1630760:MER11C(LTR)" | bnd_96 | tandem_dup_2618 |
| 08T 15 | 100146853 | 100146853 | MEF2A | intronic | 15q26.3 | Score=1736;Name="1630779:L1ME3B(LINE)" | bnd_95 | tandem_dup_2618 |
| 08T 16 | 69717415 | 69717415 | NFAT5 | intronic | 16q22.1 | Score=1872;Name="1763361:AluJb(SINE)" | bnd_98 | tandem_dup_2682 |
| 08T 16 | 69740891 | 69740891 | . | intergenic | 16q22.1 | Score=1135;Name="1763418:MSTC(LTR)" | bnd_97 | tandem_dup_2682 |
| 08T 16 | 73307539 | 73307539 | . | intergenic | 16q22.3 | Score=774;Name="1770750:L1MC4a(LINE)" | bnd_100 | tandem_dup_2370 |
| 08T 16 | 73336219 | 73336219 | . | intergenic | 16q22.3 | . | bnd_99 | tandem_dup_2370 |
| 08T 17 | 7115108 | 7115108 | DLG4 | intronic | 17p13.1 | Score=278;Name="1820470:G-rich(Low_complexity)" | bnd_66 | transl_intra_1473_0 |
| 08T 17 | 7138023 | 7138023 | . | upstream;downstream | 17p13.1 | . | bnd_65 | transl_intra_1473_0 |
| 08T 20 | 33323726 | 33323726 | NCOA6 | intronic | 20q11.22 | . | bnd_106 | tandem_dup_1953 |
| 08T 20 | 33369767 | 33369767 | NCOA6 | intronic | 20q11.22 | . | bnd_105 | tandem_dup_1953 |
| 08T X | 145675132 | 145675132 | . | intergenic | Xq27.3 | Score=324;Name="5238510:L1MC4a(LINE)" | bnd_34 | del_1980 |
| 08T X | 145676061 | 145676061 | . | intergenic | Xq27.3 | Score=19125;Name="5238512:L1MA2(LINE)" | bnd_33 | del_1980 |
| 08T Y | 18571507 | 18571507 | . | intergenic | Yq11.221 | . | bnd_120 | tandem_dup_2702 |
| 08T Y | 18583687 | 18583687 | . | intergenic | Yq11.221 | . | bnd_119 | tandem_dup_2702 |
| 10T 1 | 118587909 | 118587909 | SPAG17 | intronic | 1p12 | Score=2738;Name="238417:MER21C(LTR)" | bnd_24 | transl_inter_634 |
| 10T 2 | 164177808 | 164177808 | . | intergenic | 2q24.3 | Score=2316;Name="2512405:AluSx1(SINE)" | bnd_27 | transl_inter_1868 |
| 10T 2 | 176436293 | 176436293 | . | intergenic | 2q31.1 | Score=264;Name="2533674:(TG)n(Simple_repeat)" | bnd_38 | transl_inter_5612 |
| 10T 4 | 124630432 | 124630432 | LINC01091 | ncRNA_intronic | 4q28.1 | Score=7559;Name="3468238:L1PBa(LINE)" | bnd_21 | transl_inter_3226_0 |
| 10T 5 | 175244047 | 175244047 | CPLX2 | intronic | 5q35.2 | . | bnd_35 | transl_inter_825 |

| | | | | | | | | |
|---|---|---|---|---|---|---|---|---|
| 10T | 5 | 175277673 | 175277673 | CPLX2 | intronic | 5q35.2 | Score=670;Name="3875433:MER103C(DNA)" | bnd_29 | transl_inter_572 |
| 10T | 5 | 180210430 | 180210430 | . | intergenic | 5q35.3 | Score=808;Name="3886095:MER21A(LTR)" | bnd_2 | del_864 |
| 10T | 5 | 180224056 | 180224056 | MGAT1 | intronic | 5q35.3 | . | bnd_1 | del_864 |
| 10T | 6 | 9960645 | 9960645 | . | intergenic | 6p24.3 | . | bnd_33 | transl_inter_1368 |
| 10T | 7 | 23281726 | 23281726 | . | intergenic | 7p15.3 | Score=1899;Name="4266011:AluJr(SINE)" | bnd_14 | transl_intra_1724_0 |
| 10T | 7 | 23433762 | 23433762 | IGF2BP3 | intronic | 7p15.3 | . | bnd_16 | transl_intra_1196 |
| 10T | 7 | 23818298 | 23818298 | STK31 | intronic | 7p15.3 | Score=22263;Name="4267201:L1PA6(LINE)" | bnd_13 | transl_intra_1724_0 |
| 10T | 7 | 24425724 | 24425724 | . | intergenic | 7p15.3 | Score=991;Name="4268220:MER85(DNA)" | bnd_15 | transl_intra_1196 |
| 10T | 7 | 126758598 | 126758598 | GRM8 | intronic | 7q31.33 | . | bnd_37 | transl_inter_5612 |
| 10T | 7 | 139015685 | 139015685 | . | intergenic | 7q34 | Score=2037;Name="4471163:MER21C(LTR)" | bnd_31 | transl_inter_1363 |
| 10T | 8 | 39058191 | 39058191 | ADAM32 | intronic | 8p11.22 | Score=25528;Name="4574956:L1PA5(LINE)" | bnd_23 | transl_inter_634 |
| 10T | 12 | 25784065 | 25784065 | IFLTD1 | intronic | 12p12.1 | Score=21910;Name="955911:L1PB1(LINE)" | bnd_22 | transl_inter_3226_0 |
| 10T | 13 | 78164931 | 78164931 | SCEL | intronic | 13q22.3 | . | bnd_26 | transl_inter_1889 |
| 10T | 18 | 44213470 | 44213470 | LOXHD1 | intronic | 18q21.1 | Score=4846;Name="2042759:L1MB3(LINE)" | bnd_28 | transl_inter_1868 |
| 10T | 18 | 44213512 | 44213512 | LOXHD1 | intronic | 18q21.1 | Score=4846;Name="2042759:L1MB3(LINE)" | bnd_7 | del_inssu_1330_0/1747_0 |
| 10T | 18 | 44225212 | 44225212 | LOXHD1 | intronic | 18q21.1 | . | bnd_9 | del_inssu_1330_0/1747_0 |
| 10T | 18 | 58682270 | 58682270 | . | intergenic | 18q21.32 | . | bnd_8 | del_inssu_1330_0/1747_0 |
| 10T | 18 | 58808435 | 58808435 | . | intergenic | 18q21.32 | . | bnd_30 | transl_inter_572 |
| 10T | 18 | 58867020 | 58867020 | . | intergenic | 18q21.32 | . | bnd_32 | transl_inter_1363 |
| 10T | 18 | 69914871 | 69914871 | . | intergenic | 18q22.3 | Score=2505;Name="2087141:MER101(LTR)" | bnd_34 | transl_inter_1368 |
| 10T | 18 | 69934785 | 69934785 | . | intergenic | 18q22.3 | . | bnd_12 | transl_intra_1427_0 |
| 10T | 18 | 70175474 | 70175474 | . | intergenic | 18q22.3 | Score=1452;Name="2087583:LTR55(LTR)" | bnd_36 | transl_inter_825 |
| 10T | 18 | 70215969 | 70215969 | . | intergenic | 18q22.3 | . | bnd_25 | transl_inter_1889 |
| 10T | 18 | 77743519 | 77743519 | TXNL4A | intronic | 18q23 | . | bnd_10 | del_inssu_1330_0/1747_0 |
| 10T | 18 | 77781959 | 77781959 | . | intergenic | 18q23 | Score=8081;Name="2098229:LTR25-int(LTR)" | bnd_11 | transl_intra_1427_0 |
| 10T | 19 | 4726325 | 4726325 | . | intergenic | 19p13.3 | Score=1896;Name="2109859:AluSz(SINE)" | bnd_18 | tandem_dup_3410 |
| 10T | 19 | 4778178 | 4778178 | . | intergenic | 19p13.3 | Score=1903;Name="2110041:AluSz6(SINE)" | bnd_4 | del_insod_1948_0/1659_0 |
| 10T | 19 | 4891694 | 4891694 | ARRDC5 | intronic | 19p13.3 | Score=2202;Name="2110452:AluSg(SINE)" | bnd_17 | tandem_dup_3410 |
| 10T | 19 | 4891908 | 4891908 | ARRDC5 | intronic | 19p13.3 | Score=2202;Name="2110452:AluSg(SINE)" | bnd_20 | tandem_dup_1826 |
| 10T | 19 | 4983372 | 4983372 | KDM4B | intronic | 19p13.3 | . | bnd_5 | del_insod_1948_0/1659_0 |
| 10T | 19 | 4998104 | 4998104 | KDM4B | intronic | 19p13.3 | . | bnd_19 | tandem_dup_1826 |
| 10T | 19 | 5014770 | 5014770 | KDM4B | intronic | 19p13.3 | Score=2245;Name="2110781:AluSx3(SINE)" | bnd_3 | del_insod_1948_0/1659_0 |
| 10T | 19 | 4891324 | 4891324 | ARRDC5 | exonic | 19p13.3 | . | bnd_6 | del_insod_1948_0/1659_0 |
| 12T | 1 | 27127226 | 27127226 | . | intergenic | 1p36.11 | . | bnd_2 | del_4866 |
| 12T | 1 | 53176627 | 53176627 | . | intergenic | 1p32.3 | Score=1403;Name="118306:AluY(SINE)" | bnd_1 | del_4866 |
| 12T | 1 | 54654380 | 54654380 | CYB5RL | intronic | 1p32.3 | Score=256;Name="121927:MIRb(SINE)" | bnd_48 | transl_intra_615_0 |

| | | | | | | | | |
|---|---|---|---|---|---|---|---|---|
| 12T 1 | 54701633 | 54701633 | SSBP3 | intronic | 1p32.3 | . | bnd_47 | transl_intra_615_0 |
| 12T 1 | 59861566 | 59861566 | FGGY | intronic | 1p32.1 | . | bnd_116 | transl_inter_4266 |
| 12T 1 | 59920992 | 59920992 | FGGY | intronic | 1p32.1 | Score=349;Name="133671:L2c(LINE)" | bnd_54 | transl_intra_2478_0 |
| 12T 1 | 59931777 | 59931777 | FGGY | intronic | 1p32.1 | Score=24552;Name="133683:L1PA6(LINE)" | bnd_53 | transl_intra_2478_0 |
| 12T 1 | 61386509 | 61386509 | . | intergenic | 1p31.3 | . | bnd_118 | transl_inter_1348 |
| 12T 1 | 61401261 | 61401261 | . | intergenic | 1p31.3 | Score=297;Name="136554:(CA)n(Simple_repeat)" | bnd_120 | transl_inter_1490 |
| 12T 1 | 110584454 | 110584454 | STRIP1 | exonic | 1p13.3 | . | bnd_122 | transl_inter_2491 |
| 12T 1 | 165731227 | 165731227 | TMCO1 | intronic | 1q24.1 | . | bnd_4 | del_2089 |
| 12T 1 | 165731508 | 165731508 | TMCO1 | intronic | 1q24.1 | . | bnd_3 | del_2089 |
| 12T 1 | 189134176 | 189134176 | . | intergenic | 1q31.1 | Score=22489;Name="324774:L1PA7(LINE)" | bnd_56 | transl_intra_4047_0 |
| 12T 1 | 189169135 | 189169135 | . | intergenic | 1q31.1 | Score=582;Name="324832:MLT1I(LTR)" | bnd_55 | transl_intra_4047_0 |
| 12T 1 | 196554263 | 196554263 | KCNT2 | intronic | 1q31.3 | . | bnd_72 | tandem_dup_2290 |
| 12T 1 | 196707241 | 196707241 | CFH | intronic | 1q31.3 | Score=1908;Name="337448:THE1C(LTR)" | bnd_71 | tandem_dup_2290 |
| 12T 2 | 3486101 | 3486101 | . | intergenic | 2p25.3 | . | bnd_18 | del_4679 |
| 12T 2 | 3519628 | 3519628 | ADI1 | intronic | 2p25.3 | Score=5429;Name="2238698:MER11A(LTR)" | bnd_17 | del_4679 |
| 12T 2 | 106957928 | 106957928 | . | intergenic | 2q12.2 | . | bnd_150 | transl_inter_1797 |
| 12T 2 | 142277542 | 142277542 | LRP1B | intronic | 2q22.2 | . | bnd_20 | del_2798 |
| 12T 2 | 142891715 | 142891715 | . | intergenic | 2q22.2 | . | bnd_19 | del_2798 |
| 12T 2 | 165468901 | 165468901 | GRB14 | intronic | 2q24.3 | . | bnd_42 | del_ins_1854 |
| 12T 2 | 165501404 | 165501404 | . | intergenic | 2q24.3 | Score=215;Name="2514508:L2c(LINE)" | bnd_41 | del_ins_1854 |
| 12T 2 | 206826230 | 206826230 | . | intergenic | 2q33.3 | . | bnd_88 | tandem_dup_1619 |
| 12T 2 | 206864503 | 206864503 | INO80D | UTR3 | 2q33.3 | . | bnd_87 | tandem_dup_1619 |
| 12T 2 | 241824900 | 241824900 | . | downstream | 2q37.3 | . | bnd_90 | tandem_dup_2980 |
| 12T 2 | 241852867 | 241852867 | . | intergenic | 2q37.3 | . | bnd_89 | tandem_dup_2980 |
| 12T 3 | 38535136 | 38535136 | . | downstream | 3p22.2 | Score=240;Name="2979129:MER5B(DNA)" | bnd_96 | tandem_dup_1060 |
| 12T 3 | 38554977 | 38554977 | EXOG | intronic | 3p22.2 | . | bnd_95 | tandem_dup_1060 |
| 12T 3 | 45224279 | 45224279 | . | intergenic | 3p21.31 | Score=1891;Name="2991367:AluJr(SINE)" | bnd_121 | transl_inter_2491 |
| 12T 3 | 142610165 | 142610165 | . | intergenic | 3q23 | Score=2199;Name="3157200:THE1C(LTR)" | bnd_145 | transl_inter_105 |
| 12T 3 | 142680756 | 142680756 | PAQR9 | UTR3 | 3q23 | . | bnd_147 | transl_inter_87 |
| 12T 3 | 172025671 | 172025671 | FNDC3B | intronic | 3q26.31 | . | bnd_98 | tandem_dup_3495_0 |
| 12T 3 | 172281931 | 172281931 | . | intergenic | 3q26.31 | . | bnd_97 | tandem_dup_3495_0 |
| 12T 3 | 175518909 | 175518909 | NAALADL2 | intronic | 3q26.31 | . | bnd_125 | transl_inter_1984 |
| 12T 3 | 185139741 | 185139741 | MAP3K13 | intronic | 3q27.2 | . | bnd_68 | transl_intra_4056 |
| 12T 3 | 185140162 | 185140162 | MAP3K13 | intronic | 3q27.2 | Score=325;Name="3230720:MIR3(SINE)" | bnd_67 | transl_intra_4056 |
| 12T 3 | 185164192 | 185164192 | MAP3K13 | intronic | 3q27.2 | Score=1387;Name="3230769:AluY(SINE)" | bnd_151 | transl_inter_2545 |
| 12T 3 | 185167139 | 185167139 | MAP3K13 | intronic | 3q27.2 | Score=2409;Name="3230777:AluSz(SINE)" | bnd_153 | transl_inter_990 |
| 12T 3 | 197263722 | 197263722 | BDH1 | intronic | 3q29 | Score=2246;Name="3254800:AluSq(SINE)" | bnd_24 | del_2307 |
| 12T 3 | 197270257 | 197270257 | BDH1 | intronic | 3q29 | Score=473;Name="3254817:L3(LINE)" | bnd_23 | del_2307 |
| 12T 3 | 197270365 | 197270365 | BDH1 | intronic | 3q29 | . | bnd_26 | del_3878 |
| 12T 3 | 197271051 | 197271051 | BDH1 | intronic | 3q29 | . | bnd_25 | del_3878 |
| 12T 4 | 29465117 | 29465117 | . | intergenic | 4p15.1 | . | bnd_28 | del_3127 |
| 12T 4 | 29629933 | 29629933 | . | intergenic | 4p15.1 | Score=2545;Name="3312014:L1P4(LINE)" | bnd_27 | del_3127 |

| | | | | | | | | |
|---|---|---|---|---|---|---|---|---|
| 12T 4 | 69721508 | 69721508 | . | intergenic | 4q13.2 | Score=10787;Name="3377076:Tigger1(DNA)" | bnd_100 | tandem_dup_639 |
| 12T 4 | 69754603 | 69754603 | . | intergenic | 4q13.2 | Score=1912;Name="3377131:MLT2B3(LTR)" | bnd_99 | tandem_dup_639 |
| 12T 5 | 67445617 | 67445617 | . | intergenic | 5q13.1 | Score=1341;Name="3684289:LTR78(LTR)" | bnd_102 | tandem_dup_619 |
| 12T 5 | 67451070 | 67451070 | . | intergenic | 5q13.1 | Score=296;Name="3684301:L3(LINE)" | bnd_30 | del_1163_0 |
| 12T 5 | 75457535 | 75457535 | SV2C | intronic | 5q13.3 | Score=624;Name="3699328:L1MB8(LINE)" | bnd_139 | transl_inter_6113 |
| 12T 5 | 149943734 | 149943734 | . | intergenic | 5q33.1 | Score=6863;Name="3825027:L1MB4(LINE)" | bnd_29 | del_1163_0 |
| 12T 5 | 149999383 | 149999383 | SYNPO | intronic | 5q33.1 | . | bnd_101 | tandem_dup_619 |
| 12T 5 | 176777895 | 176777895 | LMAN2 | intronic | 5q35.3 | Score=560;Name="3878933:MIRb(SINE)" | bnd_104 | tandem_dup_2146 |
| 12T 5 | 176911646 | 176911646 | PDLIM7 | intronic | 5q35.3 | Score=1065;Name="3879217:L2a(LINE)" | bnd_103 | tandem_dup_2146 |
| 12T 6 | 49998942 | 49998942 | . | intergenic | 6p12.3 | Score=10699;Name="3981032:L1P4a(LINE)" | bnd_62 | transl_intra_1746_0 |
| 12T 6 | 50005100 | 50005100 | . | intergenic | 6p12.3 | Score=6746;Name="3981035:L1PA7(LINE)" | bnd_61 | transl_intra_1746_0 |
| 12T 6 | 73160458 | 73160458 | . | intergenic | 6q13 | Score=2200;Name="4012595:AluSc(SINE)" | bnd_149 | transl_inter_1797 |
| 12T 6 | 135130461 | 135130461 | . | intergenic | 6q23.2 | . | bnd_106 | tandem_dup_2425 |
| 12T 6 | 135212550 | 135212550 | . | intergenic | 6q23.3 | . | bnd_105 | tandem_dup_2425 |
| 12T 6 | 166785102 | 166785102 | MPC1 | intronic | 6q27 | . | bnd_143 | transl_inter_1489 |
| 12T 7 | 53811966 | 53811966 | FLJ45974 | ncRNA_intronic | 7p12.1 | Score=16393;Name="4318766:L1PA15(LINE)" | bnd_44 | del_ins_3172 |
| 12T 7 | 53842568 | 53842568 | FLJ45974 | ncRNA_intronic | 7p12.1 | Score=1901;Name="4318799:MLT1A1(LTR)" | bnd_43 | del_ins_3172 |
| 12T 7 | 70130162 | 70130162 | AUTS2 | intronic | 7q11.22 | . | bnd_32 | del_2375 |
| 12T 7 | 70240923 | 70240923 | AUTS2 | intronic | 7q11.22 | . | bnd_31 | del_2375 |
| 12T 7 | 87306428 | 87306428 | ABCB1,RUNDC3B | intronic | 7q21.12 | Score=3941;Name="4379411:L1MA9(LINE)" | bnd_108 | tandem_dup_3986 |
| 12T 7 | 87395253 | 87395253 | RUNDC3B | intronic | 7q21.12 | Score=6457;Name="4379558:L1PA17(LINE)" | bnd_107 | tandem_dup_3986 |
| 12T 8 | 39429653 | 39429653 | LOC100130964 | ncRNA_intronic | 8p11.22 | . | bnd_137 | transl_inter_6207 |
| 12T 8 | 40042729 | 40042729 | . | intergenic | 8p11.21 | . | bnd_70 | transl_intra_1448 |
| 12T 8 | 40043809 | 40043809 | . | intergenic | 8p11.21 | . | bnd_69 | transl_intra_1448 |
| 12T 8 | 40517309 | 40517309 | ZMAT4 | intronic | 8p11.21 | Score=515;Name="4577557:MIR3(SINE)" | bnd_64 | transl_intra_406 |
| 12T 8 | 40519717 | 40519717 | ZMAT4 | intronic | 8p11.21 | Score=1894;Name="4577561:AluJb(SINE)" | bnd_63 | transl_intra_406 |
| 12T 8 | 111770793 | 111770793 | . | intergenic | 8q23.2 | Score=2198;Name="4693631:AluSc(SINE)" | bnd_34 | del_607 |
| 12T 8 | 111884333 | 111884333 | . | intergenic | 8q23.2 | . | bnd_33 | del_607 |
| 12T 8 | 134414346 | 134414346 | . | intergenic | 8q24.22 | Score=847;Name="4737454:MLT1I(LTR)" | bnd_131 | transl_inter_3251 |
| 12T 9 | 112268132 | 112268132 | . | intergenic | 9q31.3 | Score=2502;Name="4922569:AluY(SINE)" | bnd_110 | tandem_dup_1026 |
| 12T 9 | 112283830 | 112283830 | . | intergenic | 9q31.3 | Score=407;Name="4922613:L2c(LINE)" | bnd_109 | tandem_dup_1026 |

| | | | | | | | | | |
|---|---|---|---|---|---|---|---|---|---|
| 12T | 9 | 116145230 | 116145230 | . | intergenic | 9q32 | Score=2157;Name="4930697:AluSx1(SINE)" | bnd_135 | transl_inter_3223 |
| 12T | 9 | 127685796 | 127685796 | GOLGA1 | intronic | 9q33.3 | . | bnd_36 | del_3030 |
| 12T | 9 | 127878946 | 127878946 | SCAI | intronic | 9q33.3 | . | bnd_35 | del_3030 |
| 12T | 9 | 137156721 | 137156721 | . | intergenic | 9q34.2 | . | bnd_133 | transl_inter_2001 |
| 12T | 9 | 137415388 | 137415388 | . | intergenic | 9q34.3 | . | bnd_119 | transl_inter_1490 |
| 12T | 10 | 26560598 | 26560598 | GAD2 | intronic | 10p12.1 | Score=819;Name="480170:Charlie10b(DNA)" | bnd_124 | transl_inter_2238 |
| 12T | 10 | 131495162 | 131495162 | MGMT | intronic | 10q26.3 | . | bnd_6 | del_1134 |
| 12T | 10 | 131517493 | 131517493 | MGMT | intronic | 10q26.3 | . | bnd_5 | del_1134 |
| 12T | 11 | 65200753 | 65200753 | . | intergenic | 11q13.1 | . | bnd_74 | tandem_dup_2983 |
| 12T | 11 | 65317488 | 65317488 | LTBP3 | intronic | 11q13.1 | Score=697;Name="781402:L2a(LINE)" | bnd_73 | tandem_dup_2983 |
| 12T | 11 | 72867909 | 72867909 | . | intergenic | 11q13.4 | . | bnd_113 | inso_4444_0/2892_0 |
| 12T | 11 | 73004875 | 73004875 | P2RY6 | intronic | 11q13.4 | . | bnd_111 | inso_4444_0/2892_0 |
| 12T | 11 | 106210778 | 106210778 | . | intergenic | 11q22.3 | . | bnd_8 | del_1921 |
| 12T | 11 | 106270569 | 106270569 | . | intergenic | 11q22.3 | Score=1666;Name="859108:MSTD(LTR)" | bnd_7 | del_1921 |
| 12T | 11 | 106271983 | 106271983 | . | intergenic | 11q22.3 | Score=330;Name="859111:MamRep564(Unknown)" | bnd_126 | transl_inter_1984 |
| 12T | 12 | 61247465 | 61247465 | . | intergenic | 12q14.1 | . | bnd_128 | transl_inter_3285 |
| 12T | 12 | 92951272 | 92951272 | . | intergenic | 12q22 | Score=714;Name="1068913:L2(LINE)" | bnd_76 | tandem_dup_4212 |
| 12T | 12 | 92955516 | 92955516 | . | intergenic | 12q22 | . | bnd_75 | tandem_dup_4212 |
| 12T | 12 | 109476116 | 109476116 | . | intergenic | 12q24.11 | Score=2580;Name="1103466:L1PA11(LINE)" | bnd_10 | del_2749 |
| 12T | 12 | 109790431 | 109790431 | . | intergenic | 12q24.11 | Score=1134;Name="1104306:L1ME2z(LINE)" | bnd_9 | del_2749 |
| 12T | 12 | 118477294 | 118477294 | WSB2 | intronic | 12q24.23 | . | bnd_130 | transl_inter_2234 |
| 12T | 12 | 125997136 | 125997136 | TMEM132B | intronic | 12q24.32 | . | bnd_78 | tandem_dup_1219 |
| 12T | 12 | 126080258 | 126080258 | TMEM132B | intronic | 12q24.32 | Score=841;Name="1145707:MIR(SINE)" | bnd_77 | tandem_dup_1219 |
| 12T | 13 | 76728249 | 76728249 | . | intergenic | 13q22.2 | . | bnd_49 | inssu_950_0/804_0 |
| 12T | 13 | 76728770 | 76728770 | . | intergenic | 13q22.2 | . | bnd_51 | inssu_950_0/804_0 |
| 12T | 13 | 76729936 | 76729936 | . | intergenic | 13q22.2 | . | bnd_12 | del_936 |
| 12T | 13 | 76731363 | 76731363 | . | intergenic | 13q22.2 | . | bnd_11 | del_936 |
| 12T | 13 | 76731624 | 76731624 | . | intergenic | 13q22.2 | . | bnd_52 | inssu_950_0/804_0 |
| 12T | 13 | 76731627 | 76731627 | . | intergenic | 13q22.2 | . | bnd_50 | inssu_950_0/804_0 |
| 12T | 13 | 113788836 | 113788836 | F10 | intronic | 13q34 | Score=2276;Name="1320522:THE1C(LTR)" | bnd_132 | transl_inter_3251 |
| 12T | 14 | 56239795 | 56239795 | . | intergenic | 14q22.3 | . | bnd_14 | del_3337 |
| 12T | 14 | 56252317 | 56252317 | LINC00520 | ncRNA_intronic | 14q22.3 | . | bnd_13 | del_3337 |
| 12T | 14 | 69110570 | 69110570 | . | intergenic | 14q24.1 | . | bnd_80 | tandem_dup_533 |
| 12T | 14 | 69205082 | 69205082 | . | intergenic | 14q24.1 | . | bnd_79 | tandem_dup_533 |
| 12T | 14 | 75671274 | 75671274 | . | intergenic | 14q24.3 | . | bnd_82 | tandem_dup_2459 |
| 12T | 14 | 75692431 | 75692431 | . | intergenic | 14q24.3 | . | bnd_81 | tandem_dup_2459 |
| 12T | 15 | 41040976 | 41040976 | RMDN3 | intronic | 15q15.1 | Score=2257;Name="1516835:AluSz(SINE)" | bnd_134 | transl_inter_2001 |
| 12T | 15 | 95151054 | 95151054 | . | intergenic | 15q26.2 | . | bnd_138 | transl_inter_6207 |
| 12T | 15 | 41055847 | 41055847 | . | upstream | 15q15.1 | . | bnd_136 | transl_inter_3223 |
| 12T | 16 | 22696892 | 22696892 | . | intergenic | 16p12.2 | . | bnd_16 | del_2638 |
| 12T | 16 | 24132105 | 24132105 | PRKCB | intronic | 16p12.2 | Score=1176;Name="1691449:AluJr(SINE)" | bnd_15 | del_2638 |
| 12T | 16 | 55415330 | 55415330 | . | intergenic | 16q12.2 | . | bnd_140 | transl_inter_6113 |
| 12T | 16 | 67717602 | 67717602 | GFOD2 | UTR3 | 16q22.1 | . | bnd_115 | transl_inter_4266 |
| 12T | 17 | 56254527 | 56254527 | . | intergenic | 17q22 | Score=377;Name="1917946:LTR78B(LTR)" | bnd_142 | transl_inter_5098 |
| 12T | 17 | 79258227 | 79258227 | SLC38A10 | intronic | 17q25.3 | . | bnd_144 | transl_inter_1489 |
| 12T | 17 | 79446634 | 79446634 | . | intergenic | 17q25.3 | Score=189;Name="1965174:MIRb(SINE)" | bnd_146 | transl_inter_105 |

| | | | | | | | | | |
|---|---|---|---|---|---|---|---|---|---|
| 12T | 17 | 79519886 | 79519886 | . | upstream | 17q25.3 | Score=21;Name="1965292:GC_rich(Low_complexity)" | bnd_148 | transl_inter_87 |
| 12T | 18 | 18520061 | 18520061 | . | intergenic | 18q11.1 | Score=1207;Name="1998922:ALR/Alpha(Satellite)" | bnd_66 | transl_intra_2741_0 |
| 12T | 18 | 21650124 | 21650124 | TTC39C | intronic | 18q11.2 | . | bnd_46 | del_1700_0 |
| 12T | 18 | 46704479 | 46704479 | DYM | intronic | 18q21.1 | . | bnd_45 | del_1700_0 |
| 12T | 18 | 71746004 | 71746004 | FBXO15 | intronic | 18q22.3 | . | bnd_84 | tandem_dup_666 |
| 12T | 18 | 71772706 | 71772706 | FBXO15 | intronic | 18q22.3 | Score=2220;Name="2090196:AluSx3(SINE)" | bnd_83 | tandem_dup_666 |
| 12T | 18 | 76596392 | 76596392 | . | intergenic | 18q23 | . | bnd_117 | transl_inter_1348 |
| 12T | 18 | 47813531 | 47813531 | CXXC1 | intronic | 18q21.1 | . | bnd_65 | transl_intra_2741_0 |
| 12T | 19 | 16586897 | 16586897 | . | intergenic | 19p13.11 | Score=2476;Name="2141987:AluY(SINE)" | bnd_141 | transl_inter_5098 |
| 12T | 19 | 30286668 | 30286668 | . | intergenic | 19q12 | . | bnd_86 | tandem_dup_2138 |
| 12T | 19 | 30302550 | 30302550 | . | upstream | 19q12 | Score=367;Name="2162985:(CCCCG)n(Simple_repea | bnd_85 | tandem_dup_2138 |
| 12T | 20 | 25392873 | 25392873 | GINS1 | intronic | 20p11.21 | . | bnd_123 | transl_inter_2238 |
| 12T | 20 | 37016115 | 37016115 | . | intergenic | 20q11.23 | Score=1739;Name="2716273:AluJb(SINE)" | bnd_92 | tandem_dup_948 |
| 12T | 20 | 37031050 | 37031050 | . | intergenic | 20q11.23 | . | bnd_114 | inso_4444_0/2892_0 |
| 12T | 20 | 37031172 | 37031172 | . | intergenic | 20q11.23 | . | bnd_112 | inso_4444_0/2892_0 |
| 12T | 20 | 37032677 | 37032677 | . | intergenic | 20q11.23 | . | bnd_91 | tandem_dup_948 |
| 12T | 20 | 56708699 | 56708699 | . | intergenic | 20q13.32 | . | bnd_58 | transl_intra_3444 |
| 12T | 20 | 56710456 | 56710456 | . | intergenic | 20q13.32 | . | bnd_57 | transl_intra_3444 |
| 12T | 20 | 59546606 | 59546606 | . | intergenic | 20q13.33 | Score=2374;Name="2767375:L1ME2(LINE)" | bnd_60 | transl_intra_3151 |
| 12T | 20 | 59564375 | 59564375 | . | intergenic | 20q13.33 | Score=380;Name="2767407:L2a(LINE)" | bnd_59 | transl_intra_3151 |
| 12T | 21 | 45029863 | 45029863 | HSF2BP,MIR6070 | ncRNA_exonic | 21q22.3 | . | bnd_129 | transl_inter_2234 |
| 12T | 21 | 47663835 | 47663835 | MCM3AP,MCM3AP-AS1 | ncRNA_intronic | 21q22.3 | Score=258;Name="2832171:MER5A(DNA)" | bnd_22 | del_3026 |
| 12T | 21 | 47931411 | 47931411 | DIP2A | exonic | 21q22.3 | . | bnd_21 | del_3026 |
| 12T | 22 | 36006742 | 36006742 | MB | intronic | 22q12.3 | Score=443;Name="2875730:MIRc(SINE)" | bnd_94 | tandem_dup_4983 |
| 12T | 22 | 36123139 | 36123139 | APOL5 | exonic | 22q12.3 | . | bnd_93 | tandem_dup_4983 |
| 12T | 22 | 36558325 | 36558325 | APOL3 | intronic | 22q12.3 | Score=228;Name="2876987:LTR43B(LTR)" | bnd_152 | transl_inter_2545 |
| 12T | 22 | 36559264 | 36559264 | APOL3 | intronic | 22q12.3 | . | bnd_154 | transl_inter_990 |
| 12T | X | 21447425 | 21447425 | CNKSR2 | intronic | Xp22.12 | . | bnd_38 | del_4940 |
| 12T | X | 21482452 | 21482452 | CNKSR2 | intronic | Xp22.12 | Score=4519;Name="5031825:L1M1(LINE)" | bnd_37 | del_4940 |
| 12T | X | 63434888 | 63434888 | . | intergenic | Xq11.2 | Score=34721;Name="5101787:ERVL-B4-int(LTR)" | bnd_40 | del_1881 |
| 12T | X | 63469492 | 63469492 | . | intergenic | Xq11.2 | Score=2255;Name="5101838:L1MA5(LINE)" | bnd_39 | del_1881 |
| 12T | X | 111099183 | 111099183 | TRPC5 | intronic | Xq23 | Score=688;Name="5177545:L2c(LINE)" | bnd_127 | transl_inter_3285 |
| 13T | 1 | 158346106 | 158346106 | . | intergenic | 1q23.1 | Score=13931;Name="270466:L1MA4A(LINE)" | bnd_2 | del_1159 |
| 13T | 1 | 158346933 | 158346933 | . | intergenic | 1q23.1 | Score=13931;Name="270466:L1MA4A(LINE)" | bnd_1 | del_1159 |
| 13T | 2 | 11548509 | 11548509 | . | intergenic | 2p25.1 | . | bnd_22 | transl_inter_1899 |
| 13T | 2 | 84435040 | 84435040 | . | intergenic | 2p11.2 | . | bnd_13 | transl_inter_5485 |
| 13T | 2 | 150999157 | 150999157 | . | intergenic | 2q23.3 | . | bnd_24 | transl_inter_7098 |
| 13T | 2 | 175970400 | 175970400 | ATF2 | intronic | 2q31.1 | . | bnd_6 | del_1637 |
| 13T | 2 | 176034488 | 176034488 | . | intergenic | 2q31.1 | . | bnd_5 | del_1637 |

| | | | | | | | | |
|---|---|---|---|---|---|---|---|---|
| 13T 3 | 97832572 | 97832572 | . | intergenic | 3q11.2 | Score=22973;Name="3078814:MER61-int(LTR)" | bnd_17 | transl_inter_6641 |
| 13T 3 | 134772694 | 134772694 | EPHB1 | intronic | 3q22.2 | Score=1734;Name="3142283:AluJr(SINE)" | bnd_26 | transl_inter_7183 |
| 13T 4 | 46616720 | 46616720 | . | intergenic | 4p12 | Score=14445;Name="3342817:Tigger1(DNA)" | bnd_21 | transl_inter_1899 |
| 13T 4 | 64445008 | 64445008 | . | intergenic | 4q13.1 | Score=31557;Name="3368552:MER52-int(LTR)" | bnd_8 | del_753 |
| 13T 4 | 64447788 | 64447788 | . | intergenic | 4q13.1 | Score=2426;Name="3368562:LOR1a(LTR)" | bnd_7 | del_753 |
| 13T 4 | 105081616 | 105081616 | . | intergenic | 4q24 | Score=184;Name="3436413:GA-rich(Low_complexity)" | bnd_19 | transl_inter_4516 |
| 13T 4 | 164403646 | 164403646 | . | intergenic | 4q32.2 | Score=468;Name="3533991:MLT1J(LTR)" | bnd_28 | transl_inter_6874 |
| 13T 4 | 185301443 | 185301443 | . | intergenic | 4q35.1 | Score=2333;Name="3568268:THE1D(LTR)" | bnd_10 | del_ins_1399 |
| 13T 4 | 185377268 | 185377268 | IRF2 | intronic | 4q35.1 | . | bnd_9 | del_ins_1399 |
| 13T 5 | 88541107 | 88541107 | . | intergenic | 5q14.3 | . | bnd_23 | transl_inter_7098 |
| 13T 6 | 51349024 | 51349024 | . | intergenic | 6p12.3 | Score=24256;Name="3983082:L1PA6(LINE)" | bnd_27 | transl_inter_6874 |
| 13T 6 | 100530008 | 100530008 | . | intergenic | 6q16.2 | . | bnd_30 | transl_inter_2336 |
| 13T 8 | 77285618 | 77285618 | . | intergenic | 8q21.11 | . | bnd_29 | transl_inter_2336 |
| 13T 8 | 97314437 | 97314437 | PTDSS1 | intronic | 8q22.1 | Score=2064;Name="4667316:AluSx(SINE)" | bnd_15 | transl_inter_6791 |
| 13T 8 | 131046469 | 131046469 | . | intergenic | 8q24.21 | Score=2417;Name="4730455:AluSz6(SINE)" | bnd_11 | transl_inter_1000 |
| 13T 12 | 55809106 | 55809106 | . | intergenic | 12q13.2 | Score=18477;Name="1003849:L1PA14(LINE)" | bnd_12 | transl_inter_1000 |
| 13T 12 | 120422389 | 120422389 | . | intergenic | 12q24.23 | Score=5020;Name="1131413:Charlie1a(DNA)" | bnd_4 | del_1241 |
| 13T 12 | 120424009 | 120424009 | . | intergenic | 12q24.23 | . | bnd_3 | del_1241 |
| 13T 13 | 55006474 | 55006474 | . | intergenic | 13q14.3 | Score=3228;Name="1224861:MER54B(LTR)" | bnd_14 | transl_inter_5485 |
| 13T 16 | 60583993 | 60583993 | . | intergenic | 16q21 | Score=9357;Name="1745147:L1MA2(LINE)" | bnd_16 | transl_inter_6791 |
| 13T 17 | 67262967 | 67262967 | ABCA5 | intronic | 17q24.3 | . | bnd_18 | transl_inter_6641 |
| 13T 18 | 25840026 | 25840026 | . | intergenic | 18q12.1 | Score=3074;Name="2012693:L1MA4A(LINE)" | bnd_20 | transl_inter_4516 |
| 13T X | 119531281 | 119531281 | . | intergenic | Xq24 | . | bnd_25 | transl_inter_7183 |
| 14T 1 | 59710081 | 59710081 | . | intergenic | 1p32.1 | Score=1881;Name="133236:MLT1B(LTR)" | bnd_44 | transl_intra_1989_0 |
| 14T 1 | 59712825 | 59712825 | . | intergenic | 1p32.1 | Score=643;Name="133245:MIR(SINE)" | bnd_43 | transl_intra_1989_0 |
| 14T 1 | 154886127 | 154886127 | . | intergenic | 1q21.3 | . | bnd_132 | transl_intra_192_0 |
| 14T 1 | 154888693 | 154888693 | . | intergenic | 1q21.3 | Score=1993;Name="263635:L1M4(LINE)" | bnd_131 | transl_intra_192_0 |
| 14T 1 | 155443373 | 155443373 | ASH1L | intronic | 1q22 | Score=1874;Name="264845:AluJo(SINE)" | bnd_170 | transl_inter_1597 |
| 14T 2 | 48910413 | 48910413 | STON1-GTF2A1L | intronic | 2p16.3 | . | bnd_160 | tandem_dup_2397 |
| 14T 2 | 48910962 | 48910962 | STON1-GTF2A1L | intronic | 2p16.3 | . | bnd_159 | tandem_dup_2397 |
| 14T 2 | 59311313 | 59311313 | . | intergenic | 2p16.1 | . | bnd_204 | transl_inter_1465 |
| 14T 2 | 59311355 | 59311355 | . | intergenic | 2p16.1 | . | bnd_96 | transl_intra_1423_0 |

| | | | | | | | | |
|---|---|---|---|---|---|---|---|---|
| 14T 2 | 73173537 | 73173537 | SFXN5 | intronic | 2p13.2 | Score=415;Name="2365137:L2b(LINE)" | bnd_95 | transl_intra_1423_0 |
| 14T 2 | 73173750 | 73173750 | SFXN5 | intronic | 2p13.2 | Score=406;Name="2365138:L2b(LINE)" | bnd_173 | transl_inter_656 |
| 14T 3 | 143605211 | 143605211 | . | intergenic | 3q24 | Score=1869;Name="3158845:MLT1A0(LTR)" | bnd_206 | transl_inter_5471 |
| 14T 3 | 143607680 | 143607680 | . | intergenic | 3q24 | . | bnd_120 | transl_intra_3447_0 |
| 14T 3 | 143611201 | 143611201 | . | intergenic | 3q24 | . | bnd_119 | transl_intra_3447_0 |
| 14T 3 | 155229403 | 155229403 | PLCH1 | intronic | 3q25.31 | Score=27435;Name="3178405:L1PA2(LINE)" | bnd_177 | transl_inter_6159 |
| 14T 3 | 163516047 | 163516047 | . | intergenic | 3q26.1 | Score=4845;Name="3192286:L1MCa(LINE)" | bnd_208 | transl_inter_8053 |
| 14T 5 | 461596 | 461596 | EXOC3 | intronic | 5p15.33 | Score=2333;Name="3580211:AluSg(SINE)" | bnd_144 | transl_intra_305 |
| 14T 5 | 462497 | 462497 | EXOC3 | intronic | 5p15.33 | . | bnd_143 | transl_intra_305 |
| 14T 5 | 547114 | 547114 | . | intergenic | 5p15.33 | . | bnd_187 | transl_inter_786 |
| 14T 5 | 570901 | 570901 | . | intergenic | 5p15.33 | . | bnd_122 | transl_intra_2076_0 |
| 14T 5 | 7760690 | 7760690 | ADCY2 | intronic | 5p15.31 | . | bnd_98 | transl_intra_1214_0 |
| 14T 5 | 7814627 | 7814627 | ADCY2 | intronic | 5p15.31 | Score=857;Name="3590066:MER20(DNA)" | bnd_199 | transl_inter_1749 |
| 14T 5 | 7814807 | 7814807 | ADCY2 | intronic | 5p15.31 | . | bnd_18 | del_1204 |
| 14T 5 | 8037299 | 8037299 | . | intergenic | 5p15.31 | Score=2860;Name="3590383:HERVK9-int(LTR)" | bnd_17 | del_1204 |
| 14T 5 | 8151665 | 8151665 | . | intergenic | 5p15.31 | . | bnd_124 | transl_intra_1122_0 |
| 14T 5 | 8230098 | 8230098 | . | intergenic | 5p15.31 | . | bnd_201 | transl_inter_1033 |
| 14T 5 | 8373713 | 8373713 | LOC729506 | ncRNA_intronic | 5p15.31 | . | bnd_169 | transl_inter_1597 |
| 14T 5 | 8374443 | 8374443 | LOC729506 | ncRNA_intronic | 5p15.31 | . | bnd_146 | transl_intra_972_0 |
| 14T 5 | 9471050 | 9471050 | SEMA5A | intronic | 5p15.31 | . | bnd_162 | tandem_dup_1070 |
| 14T 5 | 10890465 | 10890465 | . | intergenic | 5p15.2 | Score=2497;Name="3594846:AluSc(SINE)" | bnd_197 | transl_inter_1905 |
| 14T 5 | 11236362 | 11236362 | CTNND2 | intronic | 5p15.2 | . | bnd_161 | tandem_dup_1070 |
| 14T 5 | 12139832 | 12139832 | . | intergenic | 5p15.2 | . | bnd_126 | transl_intra_2271_0 |
| 14T 5 | 18605133 | 18605133 | . | intergenic | 5p14.3 | Score=779;Name="3607531:LTR79(LTR)" | bnd_189 | transl_inter_1604 |
| 14T 5 | 19442561 | 19442561 | . | intergenic | 5p14.3 | . | bnd_193 | transl_inter_5303 |
| 14T 5 | 20584049 | 20584049 | . | intergenic | 5p14.3 | Score=6446;Name="3610788:L1MB3(LINE)" | bnd_164 | tandem_dup_1693_0 |
| 14T 5 | 22067267 | 22067267 | CDH12 | intronic | 5p14.3 | Score=25009;Name="3613356:ERVL-B4-int(LTR)" | bnd_128 | transl_intra_6793_0 |
| 14T 5 | 22089575 | 22089575 | CDH12 | intronic | 5p14.3 | Score=1660;Name="3613398:L1MB8(LINE)" | bnd_123 | transl_intra_1122_0 |
| 14T 5 | 23372061 | 23372061 | . | intergenic | 5p14.2 | . | bnd_51 | del_insou_7008_0/6023_0/2713 |
| 14T 5 | 23372189 | 23372189 | . | intergenic | 5p14.2 | . | bnd_49 | del_insou_7008_0/6023_0/2713 |
| 14T 5 | 23372203 | 23372203 | . | intergenic | 5p14.2 | . | bnd_20 | del_2815 |
| 14T 5 | 24968521 | 24968521 | . | intergenic | 5p14.1 | Score=3306;Name="3618314:L1MD(LINE)" | bnd_97 | transl_intra_1214_0 |
| 14T 5 | 24989017 | 24989017 | . | intergenic | 5p14.1 | Score=3925;Name="3618337:L1MA9(LINE)" | bnd_50 | del_insou_7008_0/6023_0/2713 |
| 14T 5 | 24989957 | 24989957 | . | intergenic | 5p14.1 | Score=3925;Name="3618337:L1MA9(LINE)" | bnd_127 | transl_intra_6793_0 |
| 14T 5 | 24990968 | 24990968 | . | intergenic | 5p14.1 | Score=6199;Name="3618339:L1PA15(LINE)" | bnd_145 | transl_intra_972_0 |
| 14T 5 | 25371674 | 25371674 | . | intergenic | 5p14.1 | Score=16487;Name="3618982:L1MA2(LINE)" | bnd_163 | tandem_dup_1693_0 |
| 14T 5 | 25915164 | 25915164 | . | intergenic | 5p14.1 | . | bnd_191 | transl_inter_2100 |
| 14T 5 | 26684206 | 26684206 | . | intergenic | 5p14.1 | . | bnd_52 | del_insou_7008_0/6023_0/2713 |

| | | | | | | | | | |
|---|---|---|---|---|---|---|---|---|---|
| 14T | 5 | 26706225 | 26706225 | . | intergenic | 5p14.1 | . | bnd_22 | del_2555 |
| 14T | 5 | 26779364 | 26779364 | . | intergenic | 5p14.1 | . | bnd_21 | del_2555 |
| 14T | 5 | 26790233 | 26790233 | . | intergenic | 5p14.1 | . | bnd_19 | del_2815 |
| 14T | 5 | 28767756 | 28767756 | . | intergenic | 5p14.1 | . | bnd_121 | transl_intra_2076_0 |
| 14T | 5 | 28817252 | 28817252 | . | intergenic | 5p14.1 | Score=2015;Name="3624821:LTR9(LTR)" | bnd_183 | transl_inter_1355 |
| 14T | 5 | 28863597 | 28863597 | . | intergenic | 5p14.1 | . | bnd_125 | transl_intra_2271_0 |
| 14T | 5 | 159420483 | 159420483 | . | intergenic | 5q33.3 | Score=2746;Name="3844265:MER70A(LTR)" | bnd_166 | tandem_dup_3518 |
| 14T | 5 | 159420601 | 159420601 | . | intergenic | 5q33.3 | Score=2746;Name="3844265:MER70A(LTR)" | bnd_181 | transl_inter_4537 |
| 14T | 5 | 159420748 | 159420748 | . | intergenic | 5q33.3 | Score=758;Name="3844267:AluJr4(SINE)" | bnd_165 | tandem_dup_3518 |
| 14T | 6 | 26002634 | 26002634 | . | intergenic | 6p22.2 | . | bnd_24 | del_3090 |
| 14T | 6 | 26006187 | 26006187 | . | intergenic | 6p22.2 | Score=376;Name="3934125:LTR50(LTR)" | bnd_102 | transl_intra_1632_0 |
| 14T | 6 | 26006702 | 26006702 | . | intergenic | 6p22.2 | . | bnd_179 | transl_inter_376 |
| 14T | 6 | 26242947 | 26242947 | . | intergenic | 6p22.2 | . | bnd_175 | transl_inter_1144 |
| 14T | 6 | 26243650 | 26243650 | . | intergenic | 6p22.2 | Score=357;Name="3934598:MLT1N2(LTR)" | bnd_203 | transl_inter_1465 |
| 14T | 6 | 26244656 | 26244656 | . | intergenic | 6p22.2 | . | bnd_23 | del_3090 |
| 14T | 6 | 34789643 | 34789643 | UHRF1BP1 | intronic | 6p21.31 | . | bnd_101 | transl_intra_1632_0 |
| 14T | 6 | 53924658 | 53924658 | MLIP | intronic | 6p12.1 | . | bnd_82 | tandem_dup_3952_0 |
| 14T | 6 | 53928709 | 53928709 | MLIP | intronic | 6p12.1 | . | bnd_81 | tandem_dup_3952_0 |
| 14T | 6 | 79628998 | 79628998 | . | intergenic | 6q14.1 | Score=9753;Name="4023167:L1PA15(LINE)" | bnd_207 | transl_inter_8053 |
| 14T | 7 | 94703378 | 94703378 | PPP1R9A | intronic | 7q21.3 | Score=664;Name="4391265:MIR(SINE)" | bnd_171 | transl_inter_7881 |
| 14T | 7 | 98411921 | 98411921 | . | intergenic | 7q22.1 | . | bnd_195 | transl_inter_7638 |
| 14T | 8 | 31613647 | 31613647 | NRG1 | intronic | 8p12 | . | bnd_205 | transl_inter_5471 |
| 14T | 8 | 34645497 | 34645497 | . | intergenic | 8p12 | Score=897;Name="4566751:L2a(LINE)" | bnd_68 | del_2466_0 |
| 14T | 8 | 34650969 | 34650969 | . | intergenic | 8p12 | Score=2170;Name="4566767:AluSx(SINE)" | bnd_40 | del_insod_3204_0/4126_0 |
| 14T | 8 | 36758042 | 36758042 | KCNU1 | intronic | 8p11.23 | . | bnd_55 | del_insou_3499_0/4036_0 |
| 14T | 8 | 36768294 | 36768294 | KCNU1 | intronic | 8p11.23 | . | bnd_53 | del_insou_3499_0/4036_0 |
| 14T | 8 | 36771835 | 36771835 | KCNU1 | intronic | 8p11.23 | Score=740;Name="4570205:MER20(DNA)" | bnd_168 | tandem_dup_3876 |
| 14T | 8 | 36776825 | 36776825 | KCNU1 | intronic | 8p11.23 | . | bnd_54 | del_insou_3499_0/4036_0 |
| 14T | 8 | 36782250 | 36782250 | KCNU1 | intronic | 8p11.23 | . | bnd_42 | del_insod_3204_0/4126_0 |
| 14T | 8 | 36788227 | 36788227 | KCNU1 | intronic | 8p11.23 | Score=200;Name="4570236:L2b(LINE)" | bnd_130 | transl_intra_4127_0 |
| 14T | 8 | 36788245 | 36788245 | KCNU1 | intronic | 8p11.23 | Score=200;Name="4570236:L2b(LINE)" | bnd_100 | transl_intra_3080_0 |
| 14T | 8 | 36788676 | 36788676 | KCNU1 | intronic | 8p11.23 | . | bnd_129 | transl_intra_4127_0 |
| 14T | 8 | 36796365 | 36796365 | . | intergenic | 8p11.23 | Score=320;Name="4570250:L2(LINE)" | bnd_56 | del_insou_3499_0/4036_0 |
| 14T | 8 | 37945685 | 37945685 | . | intergenic | 8p11.23 | Score=44200;Name="4572544:HERV1 I-int(LTR)" | bnd_99 | transl_intra_3080_0 |
| 14T | 8 | 37961955 | 37961955 | . | intergenic | 8p11.23 | Score=548;Name="4572590:L1MD3(LINE)" | bnd_167 | tandem_dup_3876 |
| 14T | 8 | 39554810 | 39554810 | ADAM18 | intronic | 8p11.22 | Score=4484;Name="4575729:L1MB3(LINE)" | bnd_67 | del_2466_0 |
| 14T | 8 | 39559579 | 39559579 | ADAM18 | intronic | 8p11.22 | Score=9124;Name="4575740:L1PA12(LINE)" | bnd_41 | del_insod_3204_0/4126_0 |
| 14T | 8 | 39594159 | 39594159 | . | intergenic | 8p11.22 | Score=3642;Name="4575798:THE1B-int(LTR)" | bnd_39 | del_insod_3204_0/4126_0 |

| | | | | | | | | | |
|---|---|---|---|---|---|---|---|---|---|
| 14T | 11 | 80668966 | 80668966 | . | intergenic | 11q14.1 | Score=257;Name="815100:MIRb(SINE)" | bnd_2 | del_2278 |
| 14T | 11 | 80726671 | 80726671 | . | intergenic | 11q14.1 | Score=699;Name="815210:L2a(LINE)" | bnd_1 | del_2278 |
| 14T | 13 | 54242945 | 54242945 | . | intergenic | 13q14.3 | Score=226;Name="1223724:MIRb(SINE)" | bnd_172 | transl_inter_7881 |
| 14T | 16 | 137662 | 137662 | NPRL3 | intronic | 16p13.3 | Score=2170;Name="1634682:AluY(SINE)" | bnd_4 | del_1639 |
| 14T | 16 | 850095 | 850095 | GNG13 | intronic | 16p13.3 | . | bnd_148 | tandem_dup_3890_0 |
| 14T | 16 | 859073 | 859073 | PRR25 | intronic | 16p13.3 | Score=1989;Name="1635892:AluSz(SINE)" | bnd_84 | del_invers_2243_0/3617_0 |
| 14T | 16 | 888844 | 888844 | . | intergenic | 16p13.3 | . | bnd_174 | transl_inter_656 |
| 14T | 16 | 895258 | 895258 | . | intergenic | 16p13.3 | Score=378;Name="1635955:MER63A(DNA)" | bnd_176 | transl_inter_1144 |
| 14T | 16 | 901316 | 901316 | . | intergenic | 16p13.3 | . | bnd_3 | del_1639 |
| 14T | 16 | 1944144 | 1944144 | . | intergenic | 16p13.3 | Score=2273;Name="1637485:AluSc8(SINE)" | bnd_60 | del_inssd_944_0/1287_0 |
| 14T | 16 | 1960231 | 1960231 | . | intergenic | 16p13.3 | . | bnd_78 | tandem_dup_914_0 |
| 14T | 16 | 2885096 | 2885096 | . | intergenic | 16p13.3 | Score=876;Name="1639044:(TGGA)n(Simple_repeat)" | bnd_150 | tandem_dup_1816 |
| 14T | 16 | 2889636 | 2889636 | PRSS30P | ncRNA_exonic | 16p13.3 | . | bnd_149 | tandem_dup_1816 |
| 14T | 16 | 3039891 | 3039891 | LINC00514 | ncRNA_intronic | 16p13.3 | . | bnd_58 | del_inssd_944_0/1287_0 |
| 14T | 16 | 3062996 | 3062996 | CLDN9 | UTR5 | 16p13.3 | . | bnd_88 | transl_intra_489_0 |
| 14T | 16 | 3358741 | 3358741 | ZNF75A | intronic | 16p13.3 | . | bnd_104 | transl_intra_2218_0 |
| 14T | 16 | 3359412 | 3359412 | ZNF75A | intronic | 16p13.3 | Score=2054;Name="1640120:AluSx1(SINE)" | bnd_103 | transl_intra_2218_0 |
| 14T | 16 | 3630323 | 3630323 | . | downstream | 16p13.3 | . | bnd_47 | del_insou_2420_0/2039_0 |
| 14T | 16 | 3632658 | 3632658 | SLX4 | exonic | 16p13.3 | . | bnd_106 | transl_intra_2070_0 |
| 14T | 16 | 3632870 | 3632870 | SLX4 | intronic | 16p13.3 | . | bnd_34 | transl_intra_1369_0 |
| 14T | 16 | 3640513 | 3640513 | SLX4 | exonic | 16p13.3 | . | bnd_69 | del_inssu_890_0/2485_0 |
| 14T | 16 | 3669710 | 3669710 | . | intergenic | 16p13.3 | Score=851;Name="1640780:AluJo(SINE)" | bnd_36 | del_insod_1972_0/2544_0/11925 |
| 14T | 16 | 3983142 | 3983142 | . | intergenic | 16p13.3 | Score=2091;Name="1641477:AluSx(SINE)" | bnd_38 | del_insod_1972_0/2544_0/11925 |
| 14T | 16 | 4789110 | 4789110 | C16orf71 | intronic | 16p13.3 | . | bnd_6 | del_2686 |
| 14T | 16 | 4895604 | 4895604 | GLYR1 | intronic | 16p13.3 | . | bnd_5 | del_2686 |
| 14T | 16 | 4917108 | 4917108 | UBN1 | intronic | 16p13.3 | Score=221;Name="1643665:L2c(LINE)" | bnd_45 | del_insou_2420_0/2039_0 |
| 14T | 16 | 5415449 | 5415449 | . | intergenic | 16p13.3 | Score=2123;Name="1644897:AluSx(SINE)" | bnd_46 | del_insou_2420_0/2039_0 |
| 14T | 16 | 5774558 | 5774558 | . | intergenic | 16p13.3 | Score=401;Name="1645764:MIR(SINE)" | bnd_134 | transl_intra_800 |
| 14T | 16 | 5793591 | 5793591 | . | intergenic | 16p13.3 | Score=454;Name="1645808:MER33(DNA)" | bnd_85 | del_invers_2243_0/3617_0 |
| 14T | 16 | 5794171 | 5794171 | . | intergenic | 16p13.3 | . | bnd_76 | del_1682_0 |
| 14T | 16 | 6271756 | 6271756 | RBFOX1 | intronic | 16p13.3 | Score=2106;Name="1646881:THE1B(LTR)" | bnd_83 | del_invers_2243_0/3617_0 |
| 14T | 16 | 6296777 | 6296777 | RBFOX1 | intronic | 16p13.3 | . | bnd_71 | del_inssu_890_0/2485_0 |
| 14T | 16 | 6747710 | 6747710 | RBFOX1 | intronic | 16p13.3 | Score=457;Name="1647850:MLT1K(LTR)" | bnd_136 | transl_intra_2005 |
| 14T | 16 | 6749271 | 6749271 | RBFOX1 | intronic | 16p13.3 | . | bnd_108 | transl_intra_1696_0 |
| 14T | 16 | 6768817 | 6768817 | RBFOX1 | intronic | 16p13.3 | Score=420;Name="1647893:L1MCc(LINE)" | bnd_48 | del_insou_2420_0/2039_0 |
| 14T | 16 | 6769332 | 6769332 | RBFOX1 | intronic | 16p13.3 | Score=502;Name="1647895:L1MCc(LINE)" | bnd_107 | transl_intra_1696_0 |

| | | | | | | | | |
|---|---|---|---|---|---|---|---|---|
| 14T 16 | 7099936 | 7099936 | RBFOX1 | intronic | 16p13.3 | Score=915;Name="1648633:L1MB5(LINE)" | bnd_138 | transl_intra_3525 |
| 14T 16 | 7307254 | 7307254 | RBFOX1 | intronic | 16p13.3 | . | bnd_70 | del_inssu_890_0/2485_0 |
| 14T 16 | 8332993 | 8332993 | . | intergenic | 16p13.2 | . | bnd_90 | del_invers_1699_0/1935_0 |
| 14T 16 | 8352741 | 8352741 | . | intergenic | 16p13.2 | Score=1170;Name="1651152:AluJr(SINE)" | bnd_74 | tandem_dup_966_0 |
| 14T 16 | 9102886 | 9102886 | . | intergenic | 16p13.2 | . | bnd_110 | transl_intra_2142_0 |
| 14T 16 | 9104917 | 9104917 | . | intergenic | 16p13.2 | . | bnd_152 | tandem_dup_3205_0 |
| 14T 16 | 9710733 | 9710733 | . | intergenic | 16p13.2 | Score=480;Name="1654581:L2(LINE)" | bnd_73 | tandem_dup_966_0 |
| 14T 16 | 10438988 | 10438988 | . | intergenic | 16p13.2 | Score=1916;Name="1656356:AluSz(SINE)" | bnd_86 | del_invers_2243_0/3617_0 |
| 14T 16 | 10446196 | 10446196 | . | intergenic | 16p13.2 | . | bnd_151 | tandem_dup_3205_0 |
| 14T 16 | 10955575 | 10955575 | . | intergenic | 16p13.13 | . | bnd_8 | del_4954 |
| 14T 16 | 10956502 | 10956502 | . | intergenic | 16p13.13 | Score=527;Name="1657734:MIR3(SINE)" | bnd_7 | del_4954 |
| 14T 16 | 11495269 | 11495269 | . | intergenic | 16p13.13 | . | bnd_87 | transl_intra_489_0 |
| 14T 16 | 11587540 | 11587540 | . | intergenic | 16p13.13 | . | bnd_26 | del_ins_920 |
| 14T 16 | 12262894 | 12262894 | SNX29 | intronic | 16p13.13 | . | bnd_133 | transl_intra_800 |
| 14T 16 | 12263726 | 12263726 | SNX29 | intronic | 16p13.13 | Score=610;Name="1661049:AluJb(SINE)" | bnd_28 | del_ins_5045 |
| 14T 16 | 12373143 | 12373143 | SNX29 | intronic | 16p13.13 | . | bnd_62 | tandem_dup_2845_0 |
| 14T 16 | 12375911 | 12375911 | SNX29 | intronic | 16p13.13 | Score=1878;Name="1661310:AluSz(SINE)" | bnd_112 | transl_intra_2537_0 |
| 14T 16 | 12611999 | 12611999 | SNX29 | intronic | 16p13.12 | . | bnd_91 | del_invers_1699_0/1935_0 |
| 14T 16 | 12646294 | 12646294 | SNX29 | intronic | 16p13.12 | . | bnd_89 | del_invers_1699_0/1935_0 |
| 14T 16 | 12664953 | 12664953 | SNX29 | UTR3 | 16p13.12 | . | bnd_72 | del_inssu_890_0/2485_0 |
| 14T 16 | 13678175 | 13678175 | . | intergenic | 16p13.12 | . | bnd_109 | transl_intra_2142_0 |
| 14T 16 | 13682147 | 13682147 | . | intergenic | 16p13.12 | Score=775;Name="1664534:L3(LINE)" | bnd_178 | transl_inter_6159 |
| 14T 16 | 13748404 | 13748404 | . | intergenic | 16p13.12 | Score=1837;Name="1664723:MER21C(LTR)" | bnd_92 | del_invers_1699_0/1935_0 |
| 14T 16 | 13783186 | 13783186 | . | intergenic | 16p13.12 | . | bnd_114 | transl_intra_1968_0 |
| 14T 16 | 14036997 | 14036997 | ERCC4 | intronic | 16p13.12 | . | bnd_80 | del_ins_872_0 |
| 14T 16 | 14471624 | 14471624 | . | intergenic | 16p13.12 | Score=1161;Name="1666379:AluSz6(SINE)" | bnd_94 | transl_intra_2689_0 |
| 14T 16 | 14621851 | 14621851 | PARN | intronic | 16p13.12 | Score=3056;Name="1666779:MER82(DNA)" | bnd_154 | tandem_dup_1342_0 |
| 14T 16 | 15690899 | 15690899 | KIAA0430 | intronic | 16p13.11 | . | bnd_105 | transl_intra_2070_0 |
| 14T 16 | 16034305 | 16034305 | . | intergenic | 16p13.11 | Score=878;Name="1670496:LTR65(LTR)" | bnd_27 | del_ins_5045 |
| 14T 16 | 16035408 | 16035408 | . | intergenic | 16p13.11 | Score=731;Name="1670503:MER58B(DNA)" | bnd_113 | transl_intra_1968_0 |
| 14T 16 | 16039217 | 16039217 | . | intergenic | 16p13.11 | Score=2180;Name="1670524:AluSx3(SINE)" | bnd_156 | tandem_dup_1448_0 |
| 14T 16 | 16078040 | 16078040 | ABCC1 | intronic | 16p13.11 | Score=588;Name="1670634:L1MC4a(LINE)" | bnd_93 | transl_intra_2689_0 |
| 14T 16 | 16079126 | 16079126 | ABCC1 | intronic | 16p13.11 | . | bnd_10 | del_1438 |
| 14T 16 | 17409092 | 17409092 | XYLT1 | intronic | 16p12.3 | Score=601;Name="1674130:L2a(LINE)" | bnd_37 | del_insod_1972_0/2544_0/11925 |
| 14T 16 | 17409351 | 17409351 | XYLT1 | intronic | 16p12.3 | Score=601;Name="1674130:L2a(LINE)" | bnd_35 | del_insod_1972_0/2544_0/11925 |
| 14T 16 | 17412019 | 17412019 | XYLT1 | intronic | 16p12.3 | Score=540;Name="1674135:L2c(LINE)" | bnd_9 | del_1438 |
| 14T 16 | 17430825 | 17430825 | XYLT1 | intronic | 16p12.3 | . | bnd_153 | tandem_dup_1342_0 |
| 14T 16 | 18806223 | 18806223 | ARL6IP1 | intronic | 16p12.3 | . | bnd_59 | del_inssd_944_0/1287_0 |
| 14T 16 | 18815175 | 18815175 | . | intergenic | 16p12.3 | . | bnd_25 | del_ins_920 |
| 14T 16 | 18825871 | 18825871 | SMG1 | intronic | 16p12.3 | . | bnd_66 | del_inssd_1852_0/2204_0 |

| | | | | | | | | | |
|---|---|---|---|---|---|---|---|---|---|
| 14T | 16 | 18839116 | 18839116 | SMG1 | intronic | 16p12.3 | . | bnd_64 | del_inssd_1852_0/2204_0 |
| 14T | 16 | 19213348 | 19213348 | SYT17 | intronic | 16p12.3 | Score=3250;Name="1678805:MER21B(LTR)" | bnd_65 | del_inssd_1852_0/2204_0 |
| 14T | 16 | 19213886 | 19213886 | SYT17 | intronic | 16p12.3 | Score=3250;Name="1678805:MER21B(LTR)" | bnd_12 | del_1344_0 |
| 14T | 16 | 19223326 | 19223326 | SYT17 | intronic | 16p12.3 | Score=276;Name="1678840:L2c(LINE)" | bnd_147 | tandem_dup_3890_0 |
| 14T | 16 | 19234595 | 19234595 | SYT17 | intronic | 16p12.3 | . | bnd_140 | transl_intra_1393 |
| 14T | 16 | 19267202 | 19267202 | SYT17 | intronic | 16p12.3 | Score=539;Name="1678964:MER115(DNA)" | bnd_63 | del_inssd_1852_0/2204_0 |
| 14T | 16 | 19276781 | 19276781 | SYT17 | intronic | 16p12.3 | . | bnd_11 | del_1344_0 |
| 14T | 16 | 19858342 | 19858342 | IQCK | intronic | 16p12.3 | Score=870;Name="1680617:MIRb(SINE)" | bnd_75 | del_1682_0 |
| 14T | 16 | 19888669 | 19888669 | GPRC5B | intronic | 16p12.3 | . | bnd_57 | del_inssd_944_0/1287_0 |
| 14T | 16 | 20414136 | 20414136 | PDILT | intronic | 16p12.3 | . | bnd_155 | tandem_dup_1448_0 |
| 14T | 16 | 22862319 | 22862319 | HS3ST2 | intronic | 16p12.2 | Score=942;Name="1688035:MLT1G3(LTR)" | bnd_77 | tandem_dup_914_0 |
| 14T | 16 | 22906434 | 22906434 | HS3ST2 | intronic | 16p12.2 | Score=2224;Name="1688134:AluSx1(SINE)" | bnd_79 | del_ins_872_0 |
| 14T | 16 | 23317090 | 23317090 | SCNN1B | intronic | 16p12.2 | Score=1380;Name="1689188:AluJb(SINE)" | bnd_135 | transl_intra_2005 |
| 14T | 16 | 23449445 | 23449445 | COG7 | intronic | 16p12.2 | Score=2229;Name="1689566:L1M4(LINE)" | bnd_30 | del_ins_1482 |
| 14T | 16 | 23465669 | 23465669 | . | intergenic | 16p12.2 | . | bnd_116 | transl_intra_1522_0 |
| 14T | 16 | 24628787 | 24628787 | . | intergenic | 16p12.1 | . | bnd_33 | transl_intra_1369_0 |
| 14T | 16 | 24765599 | 24765599 | TNRC6A | intronic | 16p12.1 | . | bnd_29 | del_ins_1482 |
| 14T | 16 | 24767489 | 24767489 | TNRC6A | intronic | 16p12.1 | Score=1008;Name="1693314:MER33(DNA)" | bnd_139 | transl_intra_1393 |
| 14T | 16 | 24781060 | 24781060 | TNRC6A | intronic | 16p12.1 | . | bnd_115 | transl_intra_1522_0 |
| 14T | 16 | 25383457 | 25383457 | . | intergenic | 16p12.1 | Score=4123;Name="1694954:LTR8(LTR)" | bnd_118 | transl_intra_1014_0 |
| 14T | 16 | 25384380 | 25384380 | . | intergenic | 16p12.1 | Score=4228;Name="1694957:MER4D(LTR)" | bnd_117 | transl_intra_1014_0 |
| 14T | 16 | 26824414 | 26824414 | . | intergenic | 16p12.1 | . | bnd_14 | del_3901 |
| 14T | 16 | 26824615 | 26824615 | . | intergenic | 16p12.1 | Score=202;Name="1698317:(GTGTG)n(Simple_repeat)" | bnd_13 | del_3901 |
| 14T | 16 | 30885320 | 30885320 | BCL7C | intronic | 16p11.2 | Score=2350;Name="1709025:AluSz(SINE)" | bnd_32 | del_ins_922 |
| 14T | 16 | 30889095 | 30889095 | BCL7C | intronic | 16p11.2 | Score=288;Name="1709033:MER102a(DNA)" | bnd_31 | del_ins_922 |
| 14T | 16 | 33919549 | 33919549 | . | intergenic | 16p11.2 | Score=1101;Name="1714619:ALR/Alpha(Satellite)" | bnd_111 | transl_intra_2537_0 |
| 14T | 16 | 34009253 | 34009253 | . | intergenic | 16p11.2 | Score=1168;Name="1714723:ALR/Alpha(Satellite)" | bnd_61 | tandem_dup_2845_0 |
| 14T | 16 | 34009902 | 34009902 | . | intergenic | 16p11.2 | Score=1168;Name="1714723:ALR/Alpha(Satellite)" | bnd_137 | transl_intra_3525 |
| 14T | 16 | 63253319 | 63253319 | . | intergenic | 16q21 | Score=305;Name="1749859:HAL1(LINE)" | bnd_142 | transl_intra_2669 |
| 14T | 16 | 64064772 | 64064772 | . | intergenic | 16q21 | Score=11174;Name="1751270:L1MCa(LINE)" | bnd_141 | transl_intra_2669 |
| 14T | 16 | 67984240 | 67984240 | SLC12A4 | exonic | 16q22.1 | . | bnd_180 | transl_inter_376 |
| 14T | 17 | 13663702 | 13663702 | . | intergenic | 17p12 | . | bnd_182 | transl_inter_4537 |
| 14T | 18 | 453318 | 453318 | COLEC12 | intronic | 18p11.32 | Score=273;Name="1973022:GA-rich(Low_complexity)" | bnd_158 | tandem_dup_7305 |

| | | | | | | | | | |
|---|---|---|---|---|---|---|---|---|---|
| 14T | 18 | 694986 | 694986 | ENOSF1 | intronic | 18p11.32 | Score=2097;Name="1973521:L1MC4(LINE)" | bnd_184 | transl_inter_1355 |
| 14T | 18 | 1091448 | 1091448 | . | intergenic | 18p11.32 | . | bnd_157 | tandem_dup_7305 |
| 14T | 18 | 3844698 | 3844698 | DLGAP1 | intronic | 18p11.31 | . | bnd_186 | transl_inter_1608 |
| 14T | 18 | 3908454 | 3908454 | DLGAP1 | intronic | 18p11.31 | Score=330;Name="1979547:MER5B(DNA)" | bnd_188 | transl_inter_786 |
| 14T | 18 | 9809741 | 9809741 | RAB31 | intronic | 18p11.22 | Score=834;Name="1989509:L1ME4a(LINE)" | bnd_190 | transl_inter_1604 |
| 14T | 18 | 9830743 | 9830743 | RAB31 | intronic | 18p11.22 | Score=1235;Name="1989528:L1ME3B(LINE)" | bnd_192 | transl_inter_2100 |
| 14T | 19 | 434214 | 434214 | SHC2 | intronic | 19p13.3 | . | bnd_194 | transl_inter_5303 |
| 14T | 19 | 17548564 | 17548564 | TMEM221 | intronic | 19p13.11 | Score=316;Name="2144614:L2b(LINE)" | bnd_196 | transl_inter_7638 |
| 14T | 19 | 18587371 | 18587371 | ELL | intronic | 19p13.11 | . | bnd_16 | del_1977 |
| 14T | 19 | 18588048 | 18588048 | ELL | intronic | 19p13.11 | Score=373;Name="2147558:L1MEg(LINE)" | bnd_15 | del_1977 |
| 14T | 19 | 50649749 | 50649749 | . | intergenic | 19q13.33 | Score=225;Name="2212310:(TTA)n(Simple_repeat)" | bnd_185 | transl_inter_1608 |
| 14T | 19 | 53346472 | 53346472 | ZNF468 | intronic | 19q13.41 | . | bnd_198 | transl_inter_1905 |
| 14T | 19 | 54671221 | 54671221 | TMC4 | intronic | 19q13.42 | Score=2402;Name="2222191:AluSx1(SINE)" | bnd_200 | transl_inter_1749 |
| 14T | 19 | 54674648 | 54674648 | TMC4 | intronic | 19q13.42 | . | bnd_202 | transl_inter_1033 |
| 15T | 3 | 8560986 | 8560986 | LMCD1 | intronic | 3p26.1 | Score=542;Name="2923200:L2a(LINE)" | bnd_4 | del_4574 |
| 15T | 3 | 18854375 | 18854375 | . | intergenic | 3p24.3 | Score=762;Name="2944373:LTR79(LTR)" | bnd_3 | del_4574 |
| 15T | 8 | 91147107 | 91147107 | . | intergenic | 8q21.3 | . | bnd_8 | transl_intra_4033_0 |
| 15T | 8 | 91654585 | 91654585 | TMEM64 | intronic | 8q21.3 | . | bnd_7 | transl_intra_4033_0 |
| 15T | 9 | 21130526 | 21130526 | . | intergenic | 9p21.3 | Score=2561;Name="4794560:L1ME1(LINE)" | bnd_6 | del_5668 |
| 15T | 9 | 22709304 | 22709304 | FLJ35282 | ncRNA_intronic | 9p21.3 | Score=4739;Name="4796942:L1MB7(LINE)" | bnd_5 | del_5668 |
| 15T | 10 | 101270930 | 101270930 | . | intergenic | 10q24.2 | Score=7058;Name="611562:L1MA3(LINE)" | bnd_2 | del_4100 |
| 15T | 10 | 101282918 | 101282918 | . | intergenic | 10q24.2 | . | bnd_1 | del_4100 |
| 16T | 1 | 31478127 | 31478127 | PUM1 | intronic | 1p35.2 | . | bnd_54 | transl_inter_7890 |
| 16T | 2 | 48118341 | 48118341 | FBXO11 | intronic | 2p16.3 | Score=4555;Name="2320917:L1MB1(LINE)" | bnd_30 | del_ins_1944_0 |
| 16T | 2 | 55847775 | 55847775 | . | intergenic | 2p16.1 | Score=778;Name="2334103:Tigger3c(DNA)" | bnd_29 | del_ins_1944_0 |
| 16T | 2 | 189765562 | 189765562 | . | intergenic | 2q32.2 | Score=554;Name="2554373:L2a(LINE)" | bnd_20 | del_1329 |
| 16T | 2 | 189766257 | 189766257 | . | intergenic | 2q32.2 | . | bnd_19 | del_1329 |
| 16T | 3 | 172855599 | 172855599 | SPATA16 | intronic | 3q26.31 | . | bnd_22 | del_1537 |
| 16T | 3 | 172857904 | 172857904 | SPATA16 | intronic | 3q26.31 | . | bnd_21 | del_1537 |
| 16T | 5 | 8577856 | 8577856 | . | intergenic | 5p15.31 | . | bnd_57 | transl_inter_4543 |
| 16T | 6 | 19865047 | 19865047 | . | intergenic | 6p22.3 | . | bnd_24 | del_1092 |
| 16T | 6 | 19868659 | 19868659 | . | intergenic | 6p22.3 | . | bnd_23 | del_1092 |
| 16T | 6 | 64835034 | 64835034 | EYS | intronic | 6q12 | Score=1271;Name="3999462:LTR52(LTR)" | bnd_55 | transl_inter_7824 |
| 16T | 6 | 109234140 | 109234140 | ARMC2-AS1,ARMC2 | ncRNA_intronic | 6q21 | . | bnd_32 | del_ins_7552 |
| 16T | 6 | 109234467 | 109234467 | ARMC2,ARMC2-AS1 | ncRNA_intronic | 6q21 | . | bnd_31 | del_ins_7552 |
| 16T | 6 | 152262105 | 152262105 | ESR1 | intronic | 6q25.1 | . | bnd_26 | del_1680 |
| 16T | 6 | 152390506 | 152390506 | ESR1 | intronic | 6q25.1 | Score=1200;Name="4144976:LTR26(LTR)" | bnd_25 | del_1680 |

| | | | | | | | | |
|---|---|---|---|---|---|---|---|---|
| 16T 7 | 146670184 | 146670184 | CNTNAP2 | intronic | 7q35 | Score=2139;Name="4484361:AluSq2(SINE)" | bnd_50 | tandem_dup_5627 |
| 16T 7 | 146885603 | 146885603 | CNTNAP2 | intronic | 7q35 | . | bnd_49 | tandem_dup_5627 |
| 16T 8 | 85722830 | 85722830 | RALYL | intronic | 8q21.2 | Score=23392;Name="4647777:L1PB1(LINE)" | bnd_28 | del_4840_0 |
| 16T 8 | 128096944 | 128096944 | PRNCR1 | ncRNA_exonic | 8q24.21 | . | bnd_27 | del_4840_0 |
| 16T 9 | 30088117 | 30088117 | . | intergenic | 9p21.1 | Score=13462;Name="4808696:HERVL-int(LTR)" | bnd_34 | del_ins_1208 |
| 16T 9 | 30092676 | 30092676 | . | intergenic | 9p21.1 | . | bnd_33 | del_ins_1208 |
| 16T 9 | 37691788 | 37691788 | FRMPD1 | intronic | 9p13.2 | Score=2218;Name="4823583:AluSz(SINE)" | bnd_39 | del_insou_2122_0/1640_0 |
| 16T 9 | 38311205 | 38311205 | . | intergenic | 9p13.2 | . | bnd_44 | del_inssd_1554_0/1722_0 |
| 16T 9 | 38317620 | 38317620 | . | intergenic | 9p13.2 | Score=964;Name="4824947:LTR37A(LTR)" | bnd_37 | del_insou_2122_0/1640_0 |
| 16T 9 | 38317642 | 38317642 | . | intergenic | 9p13.2 | Score=964;Name="4824947:LTR37A(LTR)" | bnd_42 | del_inssd_1554_0/1722_0 |
| 16T 9 | 38319442 | 38319442 | . | intergenic | 9p13.2 | . | bnd_38 | del_insou_2122_0/1640_0 |
| 16T 9 | 38667448 | 38667448 | . | intergenic | 9p13.1 | . | bnd_43 | del_inssd_1554_0/1722_0 |
| 16T 9 | 38670813 | 38670813 | . | intergenic | 9p13.1 | . | bnd_40 | del_insou_2122_0/1640_0 |
| 16T 9 | 38674600 | 38674600 | . | intergenic | 9p13.1 | Score=783;Name="4825677:L1M4(LINE)" | bnd_41 | del_inssd_1554_0/1722_0 |
| 16T 11 | 60200840 | 60200840 | MS4A5 | intronic | 11q12.2 | . | bnd_2 | del_1599 |
| 16T 11 | 60202430 | 60202430 | MS4A5 | intronic | 11q12.2 | . | bnd_1 | del_1599 |
| 16T 11 | 99941275 | 99941275 | CNTN5 | exonic | 11q22.1 | . | bnd_4 | del_2365 |
| 16T 11 | 99946294 | 99946294 | CNTN5 | intronic | 11q22.1 | Score=2026;Name="848331:MER39(LTR)" | bnd_3 | del_2365 |
| 16T 11 | 103721169 | 103721169 | . | downstream | 11q22.3 | Score=1656;Name="854868:MLT1E1(LTR)" | bnd_6 | del_951 |
| 16T 11 | 103729043 | 103729043 | . | intergenic | 11q22.3 | . | bnd_5 | del_951 |
| 16T 11 | 109200744 | 109200744 | . | intergenic | 11q22.3 | Score=1414;Name="864717:LTR16A(LTR)" | bnd_8 | del_1743 |
| 16T 11 | 109204210 | 109204210 | . | intergenic | 11q22.3 | Score=1084;Name="864727:L1MA6(LINE)" | bnd_7 | del_1743 |
| 16T 12 | 53909784 | 53909784 | RP11-793H13.3,ATF7 | ncRNA_exonic | 12q13.13 | . | bnd_52 | transl_inter_1278_0 |
| 16T 13 | 99803438 | 99803438 | . | intergenic | 13q32.3 | . | bnd_10 | del_3619 |
| 16T 13 | 99804346 | 99804346 | . | intergenic | 13q32.3 | . | bnd_9 | del_3619 |
| 16T 13 | 111656051 | 111656051 | . | intergenic | 13q34 | Score=234;Name="1317987:(TG)n(Simple_repeat)" | bnd_56 | transl_inter_7824 |
| 16T 14 | 82418034 | 82418034 | . | intergenic | 14q31.1 | . | bnd_48 | tandem_dup_1939 |
| 16T 14 | 82422450 | 82422450 | . | intergenic | 14q31.1 | . | bnd_47 | tandem_dup_1939 |
| 16T 15 | 51978864 | 51978864 | SCG3 | intronic | 15q21.2 | . | bnd_36 | transl_intra_3144_0 |
| 16T 15 | 51986995 | 51986995 | SCG3 | intronic | 15q21.2 | Score=2479;Name="1537897:AluSx(SINE)" | bnd_35 | transl_intra_3144_0 |
| 16T 15 | 78584966 | 78584966 | WDR61 | exonic | 15q25.1 | . | bnd_51 | transl_inter_1278_0 |
| 16T 16 | 339923 | 339923 | AXIN1 | intronic | 16p13.3 | . | bnd_12 | del_1114 |
| 16T 16 | 340680 | 340680 | AXIN1 | intronic | 16p13.3 | . | bnd_11 | del_1114 |
| 16T 16 | 360813 | 360813 | AXIN1 | intronic | 16p13.3 | Score=425;Name="1635145:MER58C(DNA)" | bnd_13 | del_1056 |
| 16T 16 | 23076037 | 23076037 | USP31 | UTR3 | 16p12.2 | . | bnd_16 | del_1827 |
| 16T 16 | 23080618 | 23080618 | USP31 | exonic | 16p12.2 | . | bnd_15 | del_1827 |
| 16T 16 | 25445826 | 25445826 | . | intergenic | 16p12.1 | . | bnd_18 | del_1978 |
| 16T 16 | 25551946 | 25551946 | . | intergenic | 16p12.1 | . | bnd_58 | transl_inter_4543 |
| 16T 16 | 27286371 | 27286371 | FLJ21408 | ncRNA_intronic | 16p12.1 | Score=825;Name="1699535:L1ME3D(LINE)" | bnd_17 | del_1978 |
| 16T 16 | 58878367 | 58878367 | . | intergenic | 16q21 | . | bnd_60 | transl_inter_8222 |
| 16T 16 | 348149 | 348149 | AXIN1 | exonic | 16p13.3 | . | bnd_14 | del_1056 |

| | | | | | | | | |
|---|---|---|---|---|---|---|---|---|
| 16T 20 | 12625958 | 12625958 | . | intergenic | 20p12.1 | Score=208;Name="2673604:GA-rich(Low_complexity)" | bnd_53 | transl_inter_7890 |
| 16T 20 | 20434587 | 20434587 | RALGAPA2 | intronic | 20p11.23 | . | bnd_46 | transl_intra_5597 |
| 16T 20 | 20436895 | 20436895 | RALGAPA2 | intronic | 20p11.23 | Score=367;Name="2687519:L2c(LINE)" | bnd_45 | transl_intra_5597 |
| 16T 22 | 44009628 | 44009628 | EFCAB6 | intronic | 22q13.2 | Score=3254;Name="2895210:L1PREC2(LINE)" | bnd_59 | transl_inter_8222 |
| 18T 1 | 31402054 | 31402054 | . | intergenic | 1p35.2 | Score=304;Name="68423:MIRc(SINE)" | bnd_20 | transl_inter_6939 |
| 18T 1 | 37000774 | 37000774 | . | intergenic | 1p34.3 | . | bnd_22 | transl_inter_7751 |
| 18T 1 | 39588866 | 39588866 | MACF1 | intronic | 1p34.3 | Score=269;Name="87907:MER5A1(DNA)" | bnd_24 | transl_inter_7636 |
| 18T 1 | 55857969 | 55857969 | . | intergenic | 1p32.3 | Score=18757;Name="124774:L1PB3(LINE)" | bnd_26 | transl_inter_7452 |
| 18T 1 | 62531694 | 62531694 | INADL | intronic | 1p31.3 | . | bnd_28 | transl_inter_5353 |
| 18T 1 | 70037105 | 70037105 | . | intergenic | 1p31.1 | . | bnd_30 | transl_inter_4407 |
| 18T 1 | 76075731 | 76075731 | SLC44A5 | intronic | 1p31.1 | Score=1357;Name="163717:MLT1F(LTR)" | bnd_32 | transl_inter_7143 |
| 18T 1 | 90129893 | 90129893 | LRRC8C | intronic | 1p22.2 | Score=11171;Name="187405:L1PA7(LINE)" | bnd_34 | transl_inter_5633 |
| 18T 1 | 108066720 | 108066720 | . | intergenic | 1p13.3 | . | bnd_36 | transl_inter_7599 |
| 18T 1 | 112125146 | 112125146 | . | intergenic | 1p13.2 | . | bnd_38 | transl_inter_4383 |
| 18T 1 | 156571014 | 156571014 | GPATCH4 | intronic | 1q23.1 | . | bnd_40 | transl_inter_5582 |
| 18T 1 | 157406549 | 157406549 | . | intergenic | 1q23.1 | . | bnd_42 | transl_inter_3119 |
| 18T 1 | 159992679 | 159992679 | . | intergenic | 1q23.2 | . | bnd_44 | transl_inter_7510 |
| 18T 1 | 164102766 | 164102766 | . | intergenic | 1q23.3 | Score=1395;Name="280558:MLT1D(LTR)" | bnd_46 | transl_inter_7556 |
| 18T 1 | 167889395 | 167889395 | MPC2 | intronic | 1q24.2 | . | bnd_48 | transl_inter_7688 |
| 18T 1 | 169590274 | 169590274 | SELP | intronic | 1q24.2 | . | bnd_50 | transl_inter_7543 |
| 18T 1 | 170282494 | 170282494 | . | intergenic | 1q24.2 | Score=265;Name="291476:MIRb(SINE)" | bnd_52 | transl_inter_7256 |
| 18T 1 | 174289827 | 174289827 | RABGAP1L | intronic | 1q25.1 | Score=6524;Name="298583:MER11B(LTR)" | bnd_54 | transl_inter_5634 |
| 18T 1 | 211533024 | 211533024 | TRAF5 | intronic | 1q32.3 | . | bnd_56 | transl_inter_6979 |
| 18T 1 | 220534454 | 220534454 | . | intergenic | 1q41 | Score=2465;Name="379809:MLT1B(LTR)" | bnd_58 | transl_inter_7290 |
| 18T 1 | 228446447 | 228446447 | OBSCN | intronic | 1q42.13 | Score=794;Name="394486:L1M5(LINE)" | bnd_60 | transl_inter_5368 |
| 18T 1 | 240855570 | 240855570 | . | intergenic | 1q43 | . | bnd_62 | transl_inter_7659 |
| 18T 2 | 3888457 | 3888457 | DCDC2C | intronic | 2p25.3 | . | bnd_111 | transl_inter_7896 |
| 18T 2 | 8942864 | 8942864 | KIDINS220 | intronic | 2p25.1 | . | bnd_83 | transl_inter_5295 |
| 18T 2 | 15689241 | 15689241 | NBAS | intronic | 2p24.3 | Score=666;Name="2260142:MIRb(SINE)" | bnd_202 | transl_inter_4480 |
| 18T 2 | 16408144 | 16408144 | . | intergenic | 2p24.3 | . | bnd_8 | tandem_dup_332_0 |
| 18T 2 | 16408272 | 16408272 | . | intergenic | 2p24.3 | . | bnd_7 | tandem_dup_332_0 |
| 18T 2 | 50371491 | 50371491 | NRXN1 | intronic | 2p16.3 | . | bnd_197 | transl_inter_5758 |
| 18T 2 | 51049845 | 51049845 | NRXN1 | intronic | 2p16.3 | . | bnd_204 | transl_inter_7590 |
| 18T 2 | 56864750 | 56864750 | . | intergenic | 2p16.1 | . | bnd_206 | transl_inter_6963 |
| 18T 2 | 61100581 | 61100581 | LINC01185 | ncRNA_intronic | 2p16.1 | . | bnd_208 | transl_inter_4566 |
| 18T 2 | 68812965 | 68812965 | . | intergenic | 2p13.3 | . | bnd_137 | transl_inter_7713 |
| 18T 2 | 85778994 | 85778994 | GGCX | exonic | 2p11.2 | . | bnd_210 | transl_inter_7873 |
| 18T 2 | 97660720 | 97660720 | . | intergenic | 2q11.2 | . | bnd_212 | transl_inter_7817 |
| 18T 2 | 99668954 | 99668954 | TSGA10 | intronic | 2q11.2 | Score=2351;Name="2404537:AluY(SINE)" | bnd_171 | transl_inter_5335 |
| 18T 2 | 117229709 | 117229709 | . | intergenic | 2q14.1 | . | bnd_105 | transl_inter_7386 |
| 18T 2 | 120399841 | 120399841 | PCDP1 | intronic | 2q14.2 | . | bnd_143 | transl_inter_5495 |
| 18T 2 | 121464606 | 121464606 | . | intergenic | 2q14.2 | Score=612;Name="2442149:L1M5(LINE)" | bnd_214 | transl_inter_5489 |

| | | | | | | | | |
|---|---|---|---|---|---|---|---|---|
| 18T | 2 | 124273489 | 124273489 | . | intergenic | 2q14.3 | . | bnd_131 | transl_inter_7527 |
| 18T | 2 | 134220122 | 134220122 | NCKAP5 | intronic | 2q21.2 | . | bnd_216 | transl_inter_2728 |
| 18T | 2 | 139984896 | 139984896 | . | intergenic | 2q22.1 | . | bnd_218 | transl_inter_7783 |
| 18T | 2 | 151604775 | 151604775 | . | intergenic | 2q23.3 | Score=2742;Name="2492131:L1MEf(LINE)" | bnd_220 | transl_inter_3110 |
| 18T | 2 | 160144216 | 160144216 | . | upstream | 2q24.2 | . | bnd_222 | transl_inter_7189 |
| 18T | 2 | 167092067 | 167092067 | SCN9A,AC010127.3 | ncRNA_intronic | 2q24.3 | Score=575;Name="2517009:MER5A(DNA)" | bnd_224 | transl_inter_5694 |
| 18T | 2 | 167251839 | 167251839 | . | intergenic | 2q24.3 | Score=2359;Name="2517262:AluSg(SINE)" | bnd_127 | transl_inter_4534 |
| 18T | 2 | 172523951 | 172523951 | . | intergenic | 2q31.1 | . | bnd_91 | transl_inter_7673 |
| 18T | 2 | 173964562 | 173964562 | ZAK | intronic | 2q31.1 | Score=517;Name="2529233:MIR(SINE)" | bnd_185 | transl_inter_4394 |
| 18T | 2 | 200696725 | 200696725 | FTCDNL1 | ncRNA_intronic | 2q33.1 | . | bnd_226 | transl_inter_6821 |
| 18T | 2 | 230288479 | 230288479 | DNER | intronic | 2q36.3 | Score=987;Name="2625918:MER20B(DNA)" | bnd_67 | transl_inter_6777 |
| 18T | 2 | 241028190 | 241028190 | . | intergenic | 2q37.3 | . | bnd_228 | transl_inter_6925 |
| 18T | 3 | 31627729 | 31627729 | STT3B | intronic | 3p23 | Score=2665;Name="2966106:L1ME1(LINE)" | bnd_129 | transl_inter_7720 |
| 18T | 3 | 49452020 | 49452020 | TCTA | intronic | 3p21.31 | . | bnd_167 | transl_inter_7174 |
| 18T | 3 | 53791753 | 53791753 | CACNA1D | intronic | 3p21.1 | Score=213;Name="3008471:L2b(LINE)" | bnd_242 | transl_inter_3674 |
| 18T | 3 | 61394006 | 61394006 | . | intergenic | 3p14.2 | Score=761;Name="3022493:L2c(LINE)" | bnd_101 | transl_inter_7412 |
| 18T | 3 | 73369419 | 73369419 | . | intergenic | 3p13 | Score=597;Name="3045295:MLT1E2(LTR)" | bnd_189 | transl_inter_5605 |
| 18T | 3 | 76279045 | 76279045 | . | intergenic | 3p12.3 | . | bnd_159 | transl_inter_3734 |
| 18T | 3 | 82359439 | 82359439 | . | intergenic | 3p12.2 | . | bnd_244 | transl_inter_5686 |
| 18T | 3 | 89343886 | 89343886 | EPHA3 | intronic | 3p11.1 | Score=3475;Name="3070633:L1MEc(LINE)" | bnd_246 | transl_inter_5553 |
| 18T | 3 | 106473408 | 106473408 | . | intergenic | 3q13.12 | Score=15906;Name="3092801:L1MA3(LINE)" | bnd_165 | transl_inter_5536 |
| 18T | 3 | 109086623 | 109086623 | . | intergenic | 3q13.13 | Score=2235;Name="3097291:L1M3(LINE)" | bnd_248 | transl_inter_5482 |
| 18T | 3 | 110611657 | 110611657 | . | intergenic | 3q13.13 | Score=2480;Name="3099860:AluY(SINE)" | bnd_119 | transl_inter_4573 |
| 18T | 3 | 117330277 | 117330277 | . | intergenic | 3q13.32 | . | bnd_89 | transl_inter_5635 |
| 18T | 3 | 126584988 | 126584988 | CHCHD6 | intronic | 3q21.3 | Score=386;Name="3126881:MIR(SINE)" | bnd_125 | transl_inter_4402 |
| 18T | 3 | 127088043 | 127088043 | RP11-88I21.2 | ncRNA_intronic | 3q21.3 | Score=1797;Name="3127718:MER74A(LTR)" | bnd_123 | transl_inter_7326 |
| 18T | 3 | 130948266 | 130948266 | NEK11 | intronic | 3q22.1 | . | bnd_113 | transl_inter_4513 |
| 18T | 3 | 131430659 | 131430659 | CPNE4 | intronic | 3q22.1 | . | bnd_250 | transl_inter_7665 |
| 18T | 3 | 148650430 | 148650430 | . | intergenic | 3q24 | . | bnd_252 | transl_inter_7377 |
| 18T | 3 | 156743549 | 156743549 | LEKR1 | intronic | 3q25.31 | Score=1562;Name="3181036:AluJr(SINE)" | bnd_254 | transl_inter_7277 |
| 18T | 3 | 160068683 | 160068683 | IFT80 | intronic | 3q25.33 | . | bnd_141 | transl_inter_7838 |
| 18T | 3 | 166139173 | 166139173 | . | intergenic | 3q26.1 | Score=17206;Name="3196611:L1PA11(LINE)" | bnd_107 | transl_inter_7283 |
| 18T | 3 | 167541975 | 167541975 | SERPINI1 | intronic | 3q26.1 | . | bnd_237 | transl_inter_7463 |
| 18T | 3 | 189905120 | 189905120 | . | intergenic | 3q28 | . | bnd_256 | transl_inter_4297 |
| 18T | 4 | 5601859 | 5601859 | EVC2 | intronic | 4p16.2 | . | bnd_235 | transl_inter_4417 |
| 18T | 4 | 34297813 | 34297813 | . | intergenic | 4p15.1 | . | bnd_258 | transl_inter_7839 |
| 18T | 4 | 55389039 | 55389039 | . | intergenic | 4q12 | Score=590;Name="3353025:MLT1B(LTR)" | bnd_63 | transl_inter_7537 |
| 18T | 4 | 56860003 | 56860003 | CEP135 | intronic | 4q12 | . | bnd_245 | transl_inter_5553 |
| 18T | 4 | 63888022 | 63888022 | . | intergenic | 4q13.1 | . | bnd_135 | transl_inter_5401 |

| | | | | | | | | |
|---|---|---|---|---|---|---|---|---|
| 18T 4 | 78537196 | 78537196 | . | intergenic | 4q21.1 | Score=498;Name="3391889:L2a(LINE)" | bnd_233 | transl_inter_7722 |
| 18T 4 | 89433095 | 89433095 | . | intergenic | 4q22.1 | Score=252;Name="3411286:(TG)n(Simple_repeat)" | bnd_260 | transl_inter_7137 |
| 18T 4 | 93226135 | 93226135 | GRID2 | intronic | 4q22.1 | . | bnd_205 | transl_inter_6963 |
| 18T 4 | 99420620 | 99420620 | TSPAN5 | intronic | 4q23 | Score=373;Name="3427035:LTR78(LTR)" | bnd_187 | transl_inter_5755 |
| 18T 4 | 100890346 | 100890346 | . | intergenic | 4q23 | Score=1670;Name="3429622:MER2(DNA)" | bnd_262 | transl_inter_7809 |
| 18T 4 | 107464900 | 107464900 | . | intergenic | 4q24 | Score=1433;Name="3440103:L1ME3(LINE)" | bnd_71 | transl_inter_6778 |
| 18T 4 | 111037497 | 111037497 | ELOVL6 | intronic | 4q25 | . | bnd_264 | transl_inter_7464 |
| 18T 4 | 123132177 | 123132177 | KIAA1109 | exonic | 4q27 | . | bnd_2 | del_4902 |
| 18T 4 | 123132754 | 123132754 | KIAA1109 | intronic | 4q27 | . | bnd_1 | del_4902 |
| 18T 4 | 124110397 | 124110397 | SPATA5 | intronic | 4q28.1 | . | bnd_266 | transl_inter_7946 |
| 18T 4 | 138513412 | 138513412 | . | intergenic | 4q28.3 | . | bnd_217 | transl_inter_7783 |
| 18T 4 | 139454400 | 139454400 | . | intergenic | 4q28.3 | Score=13758;Name="3491872:L1MA5(LINE)" | bnd_268 | transl_inter_4557 |
| 18T 4 | 153767034 | 153767034 | ARFIP1 | intronic | 4q31.3 | Score=5827;Name="3516348:L1PA16(LINE)" | bnd_239 | transl_inter_7337 |
| 18T 4 | 163122945 | 163122945 | . | intergenic | 4q32.2 | . | bnd_270 | transl_inter_7766 |
| 18T 4 | 169473909 | 169473909 | PALLD | intronic | 4q32.3 | Score=2129;Name="3542425:AluSx(SINE)" | bnd_17 | transl_inter_3175_0 |
| 18T 4 | 174038936 | 174038936 | . | intergenic | 4q34.1 | Score=2726;Name="3549882:THE1B(LTR)" | bnd_117 | transl_inter_7779 |
| 18T 4 | 174104194 | 174104194 | GALNT7 | intronic | 4q34.1 | Score=477;Name="3550016:(CA)n(Simple_repeat)" | bnd_177 | transl_inter_7403 |
| 18T 4 | 179947198 | 179947198 | . | intergenic | 4q34.3 | . | bnd_272 | transl_inter_7498 |
| 18T 4 | 181829986 | 181829986 | . | intergenic | 4q34.3 | . | bnd_203 | transl_inter_7590 |
| 18T 4 | 184590700 | 184590700 | TRAPPC11 | intronic | 4q35.1 | . | bnd_61 | transl_inter_7659 |
| 18T 4 | 187266574 | 187266574 | F11-AS1 | ncRNA_intronic | 4q35.2 | . | bnd_173 | transl_inter_5414 |
| 18T 5 | 11662163 | 11662163 | CTNND2 | intronic | 5p15.2 | Score=369;Name="3595966:(CA)n(Simple_repeat)" | bnd_193 | transl_inter_5460 |
| 18T 5 | 12125235 | 12125235 | . | intergenic | 5p15.2 | . | bnd_81 | transl_inter_7861 |
| 18T 5 | 38304402 | 38304402 | EGFLAM | intronic | 5p13.2 | Score=3882;Name="3641867:HAL1(LINE)" | bnd_153 | transl_inter_5476 |
| 18T 5 | 84489591 | 84489591 | . | intergenic | 5q14.3 | Score=258;Name="3715445:(TAA)n(Simple_repeat)" | bnd_191 | transl_inter_5480 |
| 18T 5 | 84605865 | 84605865 | . | intergenic | 5q14.3 | . | bnd_231 | transl_inter_7815 |
| 18T 5 | 87728669 | 87728669 | LOC102546226,TMEM161B-AS1 | ncRNA_intronic | 5q14.3 | Score=444;Name="3720620:MIRb(SINE)" | bnd_247 | transl_inter_5482 |
| 18T 5 | 104326137 | 104326137 | . | intergenic | 5q21.2 | . | bnd_274 | transl_inter_7334 |
| 18T 5 | 111028563 | 111028563 | STARD4-AS1 | ncRNA_intronic | 5q22.1 | . | bnd_103 | transl_inter_3749 |
| 18T 5 | 112622185 | 112622185 | MCC | intronic | 5q22.2 | . | bnd_276 | transl_inter_5453 |
| 18T 5 | 124515736 | 124515736 | RP11-257I8.2 | ncRNA_intronic | 5q23.2 | . | bnd_278 | transl_inter_7476 |
| 18T 5 | 128562606 | 128562606 | . | intergenic | 5q23.3 | Score=892;Name="3784347:L1MC(LINE)" | bnd_249 | transl_inter_7665 |
| 18T 5 | 143282650 | 143282650 | . | intergenic | 5q31.3 | Score=531;Name="3811629:MIRb(SINE)" | bnd_201 | transl_inter_4480 |
| 18T 5 | 152353845 | 152353845 | . | intergenic | 5q33.1 | . | bnd_215 | transl_inter_2728 |
| 18T 5 | 155279918 | 155279918 | . | intergenic | 5q33.2 | . | bnd_280 | transl_inter_7089 |
| 18T 5 | 155485505 | 155485505 | . | intergenic | 5q33.2 | Score=731;Name="3836497:MER5A1(DNA)" | bnd_282 | transl_inter_7770 |
| 18T 6 | 22394935 | 22394935 | . | intergenic | 6p22.3 | . | bnd_169 | transl_inter_6804 |

| | | | | | | | | | |
|---|---|---|---|---|---|---|---|---|---|
| 18T | 6 | 43622193 | 43622193 | RSPH9 | intronic | 6p21.1 | Score=1852;Name="3970173:AluJb(SINE)" | bnd_253 | transl_inter_7277 |
| 18T | 6 | 68108955 | 68108955 | . | intergenic | 6q12 | . | bnd_23 | transl_inter_7636 |
| 18T | 6 | 77069406 | 77069406 | . | intergenic | 6q14.1 | . | bnd_284 | transl_inter_7724 |
| 18T | 6 | 81257247 | 81257247 | . | intergenic | 6q14.1 | Score=2362;Name="4025808:THE1D(LTR)" | bnd_286 | transl_inter_7065 |
| 18T | 6 | 82016501 | 82016501 | . | intergenic | 6q14.1 | . | bnd_225 | transl_inter_6821 |
| 18T | 6 | 88129909 | 88129909 | C6orf165 | intronic | 6q15 | Score=199;Name="4036628:L4(LINE)" | bnd_241 | transl_inter_3674 |
| 18T | 6 | 93359647 | 93359647 | . | intergenic | 6q16.1 | Score=972;Name="4045675:L1ME3(LINE)" | bnd_227 | transl_inter_6925 |
| 18T | 6 | 102952932 | 102952932 | . | intergenic | 6q16.3 | Score=3301;Name="4060383:L1MB3(LINE)" | bnd_288 | transl_inter_7212 |
| 18T | 6 | 103161699 | 103161699 | . | intergenic | 6q16.3 | . | bnd_290 | transl_inter_7640 |
| 18T | 6 | 116046995 | 116046995 | . | intergenic | 6q22.1 | Score=21932;Name="4083772:L1PB1(LINE)" | bnd_243 | transl_inter_5686 |
| 18T | 6 | 150641995 | 150641995 | . | intergenic | 6q25.1 | . | bnd_19 | transl_inter_6939 |
| 18T | 6 | 153179055 | 153179055 | . | intergenic | 6q25.2 | Score=2901;Name="4146279:L1ME1(LINE)" | bnd_292 | transl_inter_7116 |
| 18T | 6 | 154608455 | 154608455 | IPCEF1 | intronic | 6q25.2 | Score=1636;Name="4148688:MSTA(LTR)" | bnd_93 | transl_inter_7810 |
| 18T | 6 | 165369374 | 165369374 | . | intergenic | 6q27 | . | bnd_219 | transl_inter_3110 |
| 18T | 7 | 6251042 | 6251042 | CYTH3 | intronic | 7p22.1 | Score=284;Name="4238293:MIR(SINE)" | bnd_283 | transl_inter_7724 |
| 18T | 7 | 10888679 | 10888679 | . | intergenic | 7p21.3 | . | bnd_97 | transl_inter_7500 |
| 18T | 7 | 35680702 | 35680702 | HERPUD2 | intronic | 7p14.2 | . | bnd_291 | transl_inter_7116 |
| 18T | 7 | 61243713 | 61243713 | . | intergenic | 7q11.1 | Score=1309;Name="4326838:ALR/Alpha(Satellite)" | bnd_161 | transl_inter_7572 |
| 18T | 7 | 102677010 | 102677010 | FBXL13 | intronic | 7q22.1 | Score=1990;Name="4408079:AluSz6(SINE)" | bnd_55 | transl_inter_6979 |
| 18T | 7 | 117649561 | 117649561 | . | intergenic | 7q31.31 | . | bnd_147 | transl_inter_5578 |
| 18T | 7 | 125341050 | 125341050 | . | intergenic | 7q31.33 | . | bnd_271 | transl_inter_7498 |
| 18T | 7 | 140745720 | 140745720 | . | intergenic | 7q34 | . | bnd_155 | transl_inter_5576 |
| 18T | 7 | 144139609 | 144139609 | . | intergenic | 7q35 | Score=3977;Name="4480191:L2a(LINE)" | bnd_273 | transl_inter_7334 |
| 18T | 8 | 9472690 | 9472690 | TNKS | intronic | 8p23.1 | . | bnd_229 | transl_inter_5378 |
| 18T | 8 | 19608763 | 19608763 | . | intergenic | 8p21.3 | Score=565;Name="4537794:MIR(SINE)" | bnd_139 | transl_inter_7237 |
| 18T | 8 | 21039849 | 21039849 | . | intergenic | 8p21.3 | . | bnd_294 | transl_inter_7485 |
| 18T | 8 | 35008109 | 35008109 | . | intergenic | 8p12 | . | bnd_151 | transl_inter_7496 |
| 18T | 8 | 39422912 | 39422912 | LOC100130964 | ncRNA_intronic | 8p11.22 | Score=4910;Name="4575501:L1PA13(LINE)" | bnd_25 | transl_inter_7452 |
| 18T | 8 | 41058092 | 41058092 | . | intergenic | 8p11.21 | . | bnd_281 | transl_inter_7770 |
| 18T | 8 | 61795309 | 61795309 | . | intergenic | 8q12.2 | Score=1196;Name="4607116:L1ME5(LINE)" | bnd_259 | transl_inter_7137 |
| 18T | 8 | 64637257 | 64637257 | . | intergenic | 8q12.3 | Score=2691;Name="4611662:L1ME3A(LINE)" | bnd_59 | transl_inter_5368 |
| 18T | 8 | 71673960 | 71673960 | . | intergenic | 8q13.3 | Score=6199;Name="4624383:L1PA12(LINE)" | bnd_47 | transl_inter_7688 |
| 18T | 8 | 72342758 | 72342758 | . | intergenic | 8q13.3 | . | bnd_211 | transl_inter_7817 |
| 18T | 8 | 75417855 | 75417855 | . | intergenic | 8q21.11 | Score=4393;Name="4630835:MER4D1(LTR)" | bnd_145 | transl_inter_7948 |
| 18T | 8 | 78704131 | 78704131 | . | intergenic | 8q21.12 | . | bnd_199 | transl_inter_3723 |
| 18T | 8 | 80320568 | 80320568 | . | intergenic | 8q21.13 | . | bnd_277 | transl_inter_7476 |
| 18T | 8 | 91167538 | 91167538 | . | intergenic | 8q21.3 | Score=348;Name="4656597:MLT1H(LTR)" | bnd_287 | transl_inter_7212 |

| | | | | | | | | | |
|---|---|---|---|---|---|---|---|---|---|
| 18T | 8 | 93789253 | 93789253 | FLJ46284 | ncRNA_intronic | 8q22.1 | . | bnd_267 | transl_inter_4557 |
| 18T | 8 | 96679386 | 96679386 | LOC100616530 | ncRNA_intronic | 8q22.1 | Score=1944;Name="4666238:MLT1G1(LTR)" | bnd_263 | transl_inter_7464 |
| 18T | 8 | 106401764 | 106401764 | ZFPM2 | intronic | 8q23.1 | . | bnd_149 | transl_inter_7687 |
| 18T | 8 | 106459923 | 106459923 | ZFPM2 | intronic | 8q23.1 | . | bnd_289 | transl_inter_7640 |
| 18T | 8 | 108150850 | 108150850 | . | intergenic | 8q23.1 | Score=6853;Name="4687531:Tigger1(DNA)" | bnd_221 | transl_inter_7189 |
| 18T | 8 | 109318981 | 109318981 | . | intergenic | 8q23.1 | . | bnd_53 | transl_inter_5634 |
| 18T | 8 | 109904174 | 109904174 | . | intergenic | 8q23.1 | Score=480;Name="4690436:L2(LINE)" | bnd_175 | transl_inter_7887 |
| 18T | 8 | 121029592 | 121029592 | DEPTOR | intronic | 8q24.12 | Score=407;Name="4709561:MIRb(SINE)" | bnd_179 | transl_inter_5498 |
| 18T | 8 | 126098151 | 126098151 | KIAA0196 | intronic | 8q24.13 | . | bnd_6 | del_ins_4694 |
| 18T | 8 | 126305487 | 126305487 | NSMCE2 | intronic | 8q24.13 | . | bnd_5 | del_ins_4694 |
| 18T | 8 | 127838489 | 127838489 | . | intergenic | 8q24.21 | . | bnd_223 | transl_inter_5694 |
| 18T | 8 | 127976087 | 127976087 | . | intergenic | 8q24.21 | Score=363;Name="4724033:L2c(LINE)" | bnd_115 | transl_inter_5620 |
| 18T | 8 | 134866063 | 134866063 | . | intergenic | 8q24.22 | . | bnd_269 | transl_inter_7766 |
| 18T | 8 | 138384380 | 138384380 | . | intergenic | 8q24.23 | . | bnd_261 | transl_inter_7809 |
| 18T | 9 | 9409133 | 9409133 | PTPRD | intronic | 9p23 | . | bnd_121 | transl_inter_7255 |
| 18T | 9 | 10436986 | 10436986 | PTPRD | intronic | 9p23 | . | bnd_133 | transl_inter_7548 |
| 18T | 9 | 21471977 | 21471977 | MIR31HG | ncRNA_intronic | 9p21.3 | . | bnd_109 | transl_inter_5409 |
| 18T | 9 | 31978573 | 31978573 | . | intergenic | 9p21.1 | Score=954;Name="4811611:Tigger5(DNA)" | bnd_251 | transl_inter_7377 |
| 18T | 9 | 79168548 | 79168548 | . | intergenic | 9q21.13 | . | bnd_285 | transl_inter_7065 |
| 18T | 9 | 91119288 | 91119288 | . | intergenic | 9q22.1 | Score=335;Name="4882854:(TG)n(Simple_repeat)" | bnd_255 | transl_inter_4297 |
| 18T | 9 | 95976597 | 95976597 | WNK2 | intronic | 9q22.31 | Score=2226;Name="4890891:AluSx(SINE)" | bnd_181 | transl_inter_5501 |
| 18T | 9 | 96889562 | 96889562 | . | intergenic | 9q22.32 | Score=1008;Name="4892354:L1M7(LINE)" | bnd_265 | transl_inter_7946 |
| 18T | 9 | 119652218 | 119652218 | ASTN2 | intronic | 9q33.1 | Score=1697;Name="4938412:AluJb(SINE)" | bnd_195 | transl_inter_4560 |
| 18T | 9 | 132000095 | 132000095 | . | intergenic | 9q34.11 | . | bnd_279 | transl_inter_7089 |
| 18T | 10 | 3634066 | 3634066 | . | intergenic | 10p15.2 | . | bnd_29 | transl_inter_4407 |
| 18T | 10 | 19457500 | 19457500 | MALRD1 | intronic | 10p12.31 | . | bnd_64 | transl_inter_7537 |
| 18T | 10 | 19800307 | 19800307 | MALRD1 | intronic | 10p12.31 | . | bnd_66 | transl_inter_5563 |
| 18T | 10 | 25290764 | 25290764 | ENKUR | intronic | 10p12.1 | Score=3664;Name="477995:L1MEc(LINE)" | bnd_68 | transl_inter_6777 |
| 18T | 10 | 56622822 | 56622822 | . | intergenic | 10q21.1 | . | bnd_70 | transl_inter_7651 |
| 18T | 10 | 59886369 | 59886369 | . | intergenic | 10q21.1 | Score=2466;Name="532462:L1ME2z(LINE)" | bnd_72 | transl_inter_6778 |
| 18T | 10 | 69964206 | 69964206 | MYPN | intronic | 10q21.3 | Score=2276;Name="550177:AluSx(SINE)" | bnd_74 | transl_inter_7006 |
| 18T | 10 | 72435237 | 72435237 | ADAMTS14 | intronic | 10q22.1 | . | bnd_76 | transl_inter_7409 |
| 18T | 10 | 76057069 | 76057069 | ADK | intronic | 10q22.2 | . | bnd_78 | transl_inter_7200 |
| 18T | 10 | 89720376 | 89720376 | PTEN | intronic | 10q23.31 | . | bnd_4 | del_ins_4674 |
| 18T | 10 | 89720982 | 89720982 | PTEN | intronic | 10q23.31 | . | bnd_3 | del_ins_4674 |
| 18T | 10 | 92640721 | 92640721 | RPP30 | intronic | 10q23.31 | . | bnd_80 | transl_inter_4528 |
| 18T | 10 | 97075646 | 97075646 | SORBS1 | intronic | 10q24.1 | Score=384;Name="603013:MER117(DNA)" | bnd_82 | transl_inter_7861 |
| 18T | 10 | 101451837 | 101451837 | ENTPD7 | intronic | 10q24.2 | . | bnd_84 | transl_inter_5295 |
| 18T | 10 | 115175465 | 115175465 | . | intergenic | 10q25.3 | Score=642;Name="637488:L1ME5(LINE)" | bnd_86 | transl_inter_6618 |
| 18T | 10 | 125804726 | 125804726 | CHST15 | intronic | 10q26.13 | . | bnd_88 | transl_inter_5778 |
| 18T | 10 | 129596960 | 129596960 | . | intergenic | 10q26.2 | . | bnd_90 | transl_inter_5635 |
| 18T | 11 | 3976090 | 3976090 | STIM1 | intronic | 11p15.4 | Score=2864;Name="676499:L1ME2(LINE)" | bnd_92 | transl_inter_7673 |

| | | | | | | | | | |
|---|---|---|---|---|---|---|---|---|---|
| 18T | 11 | 15282406 | 15282406 | . | intergenic | 11p15.2 | . | bnd_94 | transl_inter_7810 |
| 18T | 11 | 16402668 | 16402668 | SOX6 | intronic | 11p15.1 | . | bnd_21 | transl_inter_7751 |
| 18T | 11 | 16410013 | 16410013 | SOX6 | intronic | 11p15.1 | Score=3436;Name="698699:L1MEf(LINE)" | bnd_37 | transl_inter_4383 |
| 18T | 11 | 30078902 | 30078902 | . | intergenic | 11p14.1 | . | bnd_96 | transl_inter_4510 |
| 18T | 11 | 47113705 | 47113705 | C11orf49 | intronic | 11p11.2 | . | bnd_98 | transl_inter_7500 |
| 18T | 11 | 99994558 | 99994558 | CNTN5 | intronic | 11q22.1 | . | bnd_100 | transl_inter_3740 |
| 18T | 11 | 109206448 | 109206448 | . | intergenic | 11q22.3 | Score=456;Name="864735:L3(LINE)" | bnd_102 | transl_inter_7412 |
| 18T | 11 | 117621931 | 117621931 | DSCAML1 | intronic | 11q23.3 | . | bnd_104 | transl_inter_3749 |
| 18T | 11 | 125065074 | 125065074 | PKNOX2 | intronic | 11q24.2 | . | bnd_106 | transl_inter_7386 |
| 18T | 12 | 881290 | 881290 | WNK1 | intronic | 12p13.33 | . | bnd_108 | transl_inter_7283 |
| 18T | 12 | 28463433 | 28463433 | CCDC91 | intronic | 12p11.22 | . | bnd_69 | transl_inter_7651 |
| 18T | 12 | 56862587 | 56862587 | SPRYD4 | intronic | 12q13.3 | . | bnd_110 | transl_inter_5409 |
| 18T | 12 | 71038061 | 71038061 | PTPRR | intronic | 12q15 | . | bnd_112 | transl_inter_7896 |
| 18T | 12 | 71177575 | 71177575 | PTPRR | intronic | 12q15 | Score=3222;Name="1031989:MER52A(LTR)" | bnd_114 | transl_inter_4513 |
| 18T | 12 | 91851890 | 91851890 | . | intergenic | 12q21.33 | . | bnd_45 | transl_inter_7556 |
| 18T | 12 | 94045494 | 94045494 | . | intergenic | 12q22 | Score=201;Name="1071407:(CA)n(Simple_repeat)" | bnd_87 | transl_inter_5778 |
| 18T | 12 | 97670938 | 97670938 | . | intergenic | 12q23.1 | . | bnd_116 | transl_inter_5620 |
| 18T | 12 | 110585072 | 110585072 | IFT81 | intronic | 12q24.11 | . | bnd_118 | transl_inter_7779 |
| 18T | 12 | 116860725 | 116860725 | . | intergenic | 12q24.22 | Score=946;Name="1122228:AluJo(SINE)" | bnd_120 | transl_inter_4573 |
| 18T | 12 | 119826842 | 119826842 | CCDC60 | intronic | 12q24.23 | . | bnd_33 | transl_inter_5633 |
| 18T | 12 | 120946669 | 120946669 | COQ5 | intronic | 12q24.31 | Score=2344;Name="1132793:AluSc(SINE)" | bnd_122 | transl_inter_7255 |
| 18T | 12 | 122962738 | 122962738 | ZCCHC8 | intronic | 12q24.31 | . | bnd_79 | transl_inter_4528 |
| 18T | 13 | 33927471 | 33927471 | STARD13 | intronic | 13q13.1 | Score=248;Name="1187714:L2c(LINE)" | bnd_65 | transl_inter_5563 |
| 18T | 13 | 43846318 | 43846318 | ENOX1 | intronic | 13q14.11 | . | bnd_124 | transl_inter_7326 |
| 18T | 13 | 45331155 | 45331155 | . | intergenic | 13q14.12 | Score=708;Name="1207427:MIR(SINE)" | bnd_126 | transl_inter_4402 |
| 18T | 13 | 53841887 | 53841887 | . | intergenic | 13q14.3 | Score=6372;Name="1223080:L1PA7(LINE)" | bnd_128 | transl_inter_4534 |
| 18T | 13 | 57521225 | 57521225 | . | intergenic | 13q21.1 | . | bnd_130 | transl_inter_7720 |
| 18T | 13 | 73666693 | 73666693 | . | intergenic | 13q22.1 | Score=2075;Name="1255225:AluSx(SINE)" | bnd_132 | transl_inter_7527 |
| 18T | 13 | 78851237 | 78851237 | RNF219-AS1 | ncRNA_intronic | 13q22.3 | . | bnd_134 | transl_inter_7548 |
| 18T | 13 | 86945399 | 86945399 | . | intergenic | 13q31.1 | . | bnd_136 | transl_inter_5401 |
| 18T | 13 | 90971784 | 90971784 | . | intergenic | 13q31.3 | . | bnd_31 | transl_inter_7143 |
| 18T | 13 | 92148616 | 92148616 | GPC5 | intronic | 13q31.3 | Score=4191;Name="1285780:L1MA2(LINE)" | bnd_138 | transl_inter_7713 |
| 18T | 13 | 92273400 | 92273400 | GPC5 | intronic | 13q31.3 | . | bnd_140 | transl_inter_7237 |
| 18T | 13 | 93965310 | 93965310 | GPC6 | intronic | 13q31.3 | . | bnd_75 | transl_inter_7409 |
| 18T | 13 | 94868451 | 94868451 | GPC6 | intronic | 13q31.3 | . | bnd_142 | transl_inter_7838 |
| 18T | 13 | 109576846 | 109576846 | MYO16 | intronic | 13q33.3 | . | bnd_144 | transl_inter_5495 |
| 18T | 14 | 27856134 | 27856134 | . | intergenic | 14q12 | . | bnd_146 | transl_inter_7948 |
| 18T | 14 | 34547140 | 34547140 | . | intergenic | 14q13.1 | Score=307;Name="1347542:L2(LINE)" | bnd_148 | transl_inter_5578 |
| 18T | 14 | 47035596 | 47035596 | . | intergenic | 14q21.2 | Score=907;Name="1368983:MSTB1(LTR)" | bnd_150 | transl_inter_7687 |
| 18T | 14 | 47504056 | 47504056 | MDGA2 | intronic | 14q21.3 | . | bnd_27 | transl_inter_5353 |
| 18T | 14 | 48594346 | 48594346 | . | intergenic | 14q21.3 | Score=348;Name="1371409:MIR(SINE)" | bnd_35 | transl_inter_7599 |
| 18T | 14 | 51217488 | 51217488 | NIN | intronic | 14q22.1 | Score=6572;Name="1376340:L1MC4a(LINE)" | bnd_152 | transl_inter_7496 |
| 18T | 14 | 60515974 | 60515974 | LRRC9 | ncRNA_intronic | 14q23.1 | Score=11786;Name="1392599:L1PA11(LINE)" | bnd_154 | transl_inter_5476 |

| | | | | | | | | |
|---|---|---|---|---|---|---|---|---|
| 18T 14 | 60561164 | 60561164 | PCNXL4 | intronic | 14q23.1 | Score=2129;Name="1392665:LTR46(LTR)" | bnd_85 | transl_inter_6618 |
| 18T 14 | 68226115 | 68226115 | ZFYVE26 | intronic | 14q24.1 | Score=2255;Name="1407042:AluSc(SINE)" | bnd_156 | transl_inter_5576 |
| 18T 14 | 71205193 | 71205193 | MAP3K9 | intronic | 14q24.2 | . | bnd_158 | transl_inter_7360 |
| 18T 14 | 72562430 | 72562430 | RGS6 | intronic | 14q24.2 | . | bnd_77 | transl_inter_7200 |
| 18T 14 | 76221108 | 76221108 | TTLL5 | intronic | 14q24.3 | Score=1153;Name="1423494:Charlie4a(DNA)" | bnd_160 | transl_inter_3734 |
| 18T 14 | 83153021 | 83153021 | . | intergenic | 14q31.1 | Score=2542;Name="1436384:L1MA1(LINE)" | bnd_162 | transl_inter_7572 |
| 18T 14 | 87496332 | 87496332 | . | intergenic | 14q31.3 | Score=640;Name="1443545:MIR(SINE)" | bnd_39 | transl_inter_5582 |
| 18T 14 | 101393225 | 101393225 | . | upstream | 14q32.2 | . | bnd_164 | transl_inter_7401 |
| 18T 14 | 105052145 | 105052145 | C14orf180 | intronic | 14q32.33 | Score=229;Name="1478800:GA-rich(Low_complexity)" | bnd_166 | transl_inter_5536 |
| 18T 15 | 38288772 | 38288772 | . | intergenic | 15q14 | . | bnd_168 | transl_inter_7174 |
| 18T 15 | 58180705 | 58180705 | . | intergenic | 15q21.3 | Score=329;Name="1549025:MER94(DNA)" | bnd_41 | transl_inter_3119 |
| 18T 15 | 61182849 | 61182849 | RORA | intronic | 15q22.2 | . | bnd_170 | transl_inter_6804 |
| 18T 15 | 66837333 | 66837333 | ZWILCH | intronic | 15q22.31 | Score=2475;Name="1566525:AluSx(SINE)" | bnd_172 | transl_inter_5335 |
| 18T 15 | 83950256 | 83950256 | BNC1 | intronic | 15q25.2 | . | bnd_174 | transl_inter_5414 |
| 18T 16 | 13274927 | 13274927 | SHISA9 | intronic | 16p13.12 | Score=276;Name="1663564:L2b(LINE)" | bnd_176 | transl_inter_7887 |
| 18T 16 | 17055173 | 17055173 | . | intergenic | 16p12.3 | . | bnd_178 | transl_inter_7403 |
| 18T 16 | 26416596 | 26416596 | . | intergenic | 16p12.1 | Score=2032;Name="1697369:AluSx1(SINE)" | bnd_180 | transl_inter_5498 |
| 18T 16 | 53893672 | 53893672 | FTO | intronic | 16q12.2 | Score=4825;Name="1731377:L1MA9(LINE)" | bnd_182 | transl_inter_5501 |
| 18T 16 | 58032002 | 58032002 | ZNF319 | exonic | 16q21 | . | bnd_184 | transl_inter_7242 |
| 18T 16 | 74976944 | 74976944 | WDR59 | intronic | 16q23.1 | . | bnd_186 | transl_inter_4394 |
| 18T 16 | 76443904 | 76443904 | CNTNAP4 | intronic | 16q23.1 | Score=388;Name="1777352:MIRb(SINE)" | bnd_188 | transl_inter_5755 |
| 18T 17 | 6700907 | 6700907 | . | intergenic | 17p13.1 | . | bnd_190 | transl_inter_5605 |
| 18T 17 | 61294116 | 61294116 | TANC2 | intronic | 17q23.3 | . | bnd_192 | transl_inter_5480 |
| 18T 18 | 12603391 | 12603391 | SPIRE1 | intronic | 18p11.21 | Score=3679;Name="1994214:MER21B(LTR)" | bnd_194 | transl_inter_5460 |
| 18T 18 | 47261968 | 47261968 | . | intergenic | 18q21.1 | Score=1423;Name="2048568:MLT1E(LTR)" | bnd_43 | transl_inter_7510 |
| 18T 18 | 55625811 | 55625811 | . | intergenic | 18q21.31 | Score=1940;Name="2062678:AluJo(SINE)" | bnd_196 | transl_inter_4560 |
| 18T 18 | 62688988 | 62688988 | . | intergenic | 18q22.1 | . | bnd_198 | transl_inter_5758 |
| 18T 18 | 69976768 | 69976768 | . | intergenic | 18q22.3 | . | bnd_200 | transl_inter_3723 |
| 18T 19 | 5007705 | 5007705 | KDM4B | intronic | 19p13.3 | Score=2025;Name="2110765:AluJo(SINE)" | bnd_73 | transl_inter_7006 |
| 18T 19 | 28288491 | 28288491 | . | intergenic | 19q11 | . | bnd_10 | transl_intra_2853 |
| 18T 19 | 28289060 | 28289060 | . | intergenic | 19q11 | . | bnd_9 | transl_intra_2853 |
| 18T 19 | 31091035 | 31091035 | . | intergenic | 19q12 | Score=1992;Name="2164361:AluSz(SINE)" | bnd_16 | tandem_dup_3501 |
| 18T 19 | 31123713 | 31123713 | . | intergenic | 19q12 | . | bnd_14 | transl_intra_1200 |
| 18T 19 | 31125077 | 31125077 | . | intergenic | 19q12 | . | bnd_13 | transl_intra_1200 |
| 18T 19 | 31749158 | 31749158 | . | intergenic | 19q12 | Score=507;Name="2165404:LTR16C(LTR)" | bnd_12 | transl_intra_2152_0 |
| 18T 19 | 31750737 | 31750737 | . | intergenic | 19q12 | . | bnd_11 | transl_intra_2152_0 |
| 18T 19 | 32427996 | 32427996 | . | intergenic | 19q13.11 | . | bnd_15 | tandem_dup_3501 |
| 18T 19 | 33838653 | 33838653 | . | intergenic | 19q13.11 | . | bnd_183 | transl_inter_7242 |

| | | | | | | | | |
|---|---|---|---|---|---|---|---|---|
| 18T 19 | 38295359 | 38295359 | . | intergenic | 19q13.12 | Score=1394;Name="2180007:MER70C(LTR)" | bnd_18 | transl_inter_3175_0 |
| 18T 20 | 11848122 | 11848122 | . | intergenic | 20p12.2 | Score=3999;Name="2672342:Tigger6a(DNA)" | bnd_57 | transl_inter_7290 |
| 18T 20 | 13219288 | 13219288 | ISM1,ISM1-AS1 | ncRNA_intronic | 20p12.1 | . | bnd_230 | transl_inter_5378 |
| 18T 20 | 25569799 | 25569799 | . | intergenic | 20p11.21 | Score=2799;Name="2695498:MER21A(LTR)" | bnd_213 | transl_inter_5489 |
| 18T 20 | 34424480 | 34424480 | PHF20 | intronic | 20q11.23 | Score=21;Name="2709547:AT_rich(Low_complexity)" | bnd_232 | transl_inter_7815 |
| 18T 20 | 39401959 | 39401959 | . | intergenic | 20q12 | . | bnd_234 | transl_inter_7722 |
| 18T 20 | 60621000 | 60621000 | TAF4 | intronic | 20q13.33 | . | bnd_163 | transl_inter_7401 |
| 18T 21 | 39015902 | 39015902 | KCNJ6 | intronic | 21q22.13 | . | bnd_236 | transl_inter_4417 |
| 18T 21 | 45920658 | 45920658 | TSPEAR | intronic | 21q22.3 | Score=317;Name="2829796:L1M5(LINE)" | bnd_207 | transl_inter_4566 |
| 18T 22 | 23579330 | 23579330 | BCR | intronic | 22q11.23 | . | bnd_238 | transl_inter_7463 |
| 18T 22 | 34762123 | 34762123 | . | intergenic | 22q12.3 | Score=18536;Name="2872829:L1PA13(LINE)" | bnd_240 | transl_inter_7337 |
| 18T X | 4515902 | 4515902 | . | intergenic | Xp22.32 | . | bnd_257 | transl_inter_7839 |
| 18T X | 11992444 | 11992444 | . | intergenic | Xp22.2 | Score=2579;Name="5013849:MSTB1(LTR)" | bnd_51 | transl_inter_7256 |
| 18T X | 17612043 | 17612043 | NHS | intronic | Xp22.13 | . | bnd_49 | transl_inter_7543 |
| 18T X | 76220650 | 76220650 | LOC101928469 | ncRNA_intronic | Xq21.1 | Score=5241;Name="5122984:L1PA8A(LINE)" | bnd_275 | transl_inter_5453 |
| 18T X | 78782593 | 78782593 | . | intergenic | Xq21.1 | Score=6140;Name="5126787:L1PA15-16(LINE)" | bnd_209 | transl_inter_7873 |
| 18T X | 83701514 | 83701514 | HDX | intronic | Xq21.1 | . | bnd_157 | transl_inter_7360 |
| 18T X | 112228775 | 112228775 | . | intergenic | Xq23 | . | bnd_95 | transl_inter_4510 |
| 18T X | 125851166 | 125851166 | . | intergenic | Xq25 | . | bnd_99 | transl_inter_3740 |
| 18T X | 131321794 | 131321794 | . | intergenic | Xq26.2 | Score=379;Name="5212427:L2c(LINE)" | bnd_293 | transl_inter_7485 |
| 20T 1 | 49178225 | 49178225 | AGBL4 | intronic | 1p33 | Score=186;Name="109166:MIR(SINE)" | bnd_2 | del_1524 |
| 20T 1 | 49187034 | 49187034 | AGBL4 | intronic | 1p33 | . | bnd_1 | del_1524 |
| 20T 1 | 60656239 | 60656239 | . | intergenic | 1p32.1 | Score=2443;Name="135102:MSTB(LTR)" | bnd_4 | del_6606_0 |
| 20T 1 | 72343683 | 72343683 | NEGR1 | intronic | 1p31.1 | . | bnd_40 | del_ins_3675 |
| 20T 1 | 72344745 | 72344745 | NEGR1 | intronic | 1p31.1 | Score=1740;Name="157805:AluSp(SINE)" | bnd_39 | del_ins_3675 |
| 20T 1 | 84670559 | 84670559 | PRKACB | UTR3 | 1p31.1 | . | bnd_72 | tandem_dup_1226 |
| 20T 1 | 84880543 | 84880543 | DNASE2B | exonic | 1p31.1 | . | bnd_71 | tandem_dup_1226 |
| 20T 1 | 116470170 | 116470170 | . | intergenic | 1p13.1 | . | bnd_90 | transl_inter_7436 |
| 20T 1 | 190127747 | 190127747 | BRINP3 | intronic | 1q31.1 | Score=2435;Name="326453:L1PA16(LINE)" | bnd_6 | del_2025 |
| 20T 1 | 190129305 | 190129305 | BRINP3 | intronic | 1q31.1 | . | bnd_5 | del_2025 |
| 20T 1 | 235798886 | 235798886 | GNG4 | intronic | 1q42.3 | Score=912;Name="408049:MSTB1(LTR)" | bnd_3 | del_6606_0 |
| 20T 2 | 57972907 | 57972907 | . | intergenic | 2p16.1 | . | bnd_22 | del_2893 |
| 20T 2 | 58431255 | 58431255 | FANCL | intronic | 2p16.1 | . | bnd_21 | del_2893 |
| 20T 2 | 121082278 | 121082278 | . | intergenic | 2q14.2 | . | bnd_42 | del_ins_856 |
| 20T 2 | 176535888 | 176535888 | . | intergenic | 2q31.1 | Score=21743;Name="2533799:L1PA7(LINE)" | bnd_91 | transl_inter_7172 |
| 20T 2 | 208541443 | 208541443 | . | intergenic | 2q33.3 | Score=2368;Name="2587706:AluSx1(SINE)" | bnd_76 | tandem_dup_2065 |
| 20T 2 | 208800481 | 208800481 | PLEKHM3 | intronic | 2q33.3 | . | bnd_75 | tandem_dup_2065 |
| 20T 2 | 121101952 | 121101952 | . | intergenic | 2q14.2 | . | bnd_41 | del_ins_856 |
| 20T 3 | 62277598 | 62277598 | PTPRG,PTPRG-AS1 | ncRNA_intronic | 3p14.2 | . | bnd_24 | del_2941 |

| | | | | | | | | |
|---|---|---|---|---|---|---|---|---|
| 20T 3 | 62278557 | 62278557 | PTPRG,PTPRG-AS1 | ncRNA_intronic | 3p14.2 | . | bnd_23 | del_2941 |
| 20T 3 | 177166915 | 177166915 | LINC00578 | ncRNA_intronic | 3q26.32 | . | bnd_104 | transl_inter_7380 |
| 20T 4 | 26665928 | 26665928 | TBC1D19 | intronic | 4p15.2 | . | bnd_44 | del_ins_1521 |
| 20T 4 | 26668158 | 26668158 | TBC1D19 | intronic | 4p15.2 | . | bnd_43 | del_ins_1521 |
| 20T 4 | 95407314 | 95407314 | PDLIM5 | intronic | 4q22.3 | . | bnd_26 | del_1177 |
| 20T 4 | 95654554 | 95654554 | . | intergenic | 4q22.3 | Score=2103;Name="3421186:AluSz(SINE)" | bnd_25 | del_1177 |
| 20T 4 | 103761699 | 103761699 | UBE2D3 | intronic | 4q24 | Score=4458;Name="3434262:L1MA4A(LINE)" | bnd_28 | del_2349 |
| 20T 4 | 103780319 | 103780319 | UBE2D3 | intronic | 4q24 | Score=2439;Name="3434315:L1M3c(LINE)" | bnd_27 | del_2349 |
| 20T 5 | 41038205 | 41038205 | MROH2B | intronic | 5p13.1 | . | bnd_78 | tandem_dup_1069 |
| 20T 5 | 41289679 | 41289679 | . | intergenic | 5p13.1 | Score=724;Name="3646921:L2c(LINE)" | bnd_77 | tandem_dup_1069 |
| 20T 5 | 51309154 | 51309154 | . | intergenic | 5q11.2 | . | bnd_80 | tandem_dup_3313_0 |
| 20T 5 | 51788270 | 51788270 | . | intergenic | 5q11.2 | . | bnd_82 | tandem_dup_3783 |
| 20T 5 | 51788279 | 51788279 | . | intergenic | 5q11.2 | . | bnd_60 | transl_intra_4305_0 |
| 20T 5 | 51788598 | 51788598 | . | intergenic | 5q11.2 | . | bnd_59 | transl_intra_4305_0 |
| 20T 5 | 51788606 | 51788606 | . | intergenic | 5q11.2 | . | bnd_66 | transl_intra_2041_0 |
| 20T 5 | 51792885 | 51792885 | . | intergenic | 5q11.2 | . | bnd_81 | tandem_dup_3783 |
| 20T 5 | 51817394 | 51817394 | . | intergenic | 5q11.2 | . | bnd_68 | transl_intra_2576 |
| 20T 5 | 52000992 | 52000992 | . | intergenic | 5q11.2 | . | bnd_67 | transl_intra_2576 |
| 20T 5 | 52055861 | 52055861 | . | intergenic | 5q11.2 | Score=1002;Name="3657986:MER3(DNA)" | bnd_79 | tandem_dup_3313_0 |
| 20T 5 | 54745933 | 54745933 | PPAP2A | intronic | 5q11.2 | . | bnd_65 | transl_intra_2041_0 |
| 20T 5 | 87635404 | 87635404 | TMEM161B-AS1 | ncRNA_intronic | 5q14.3 | Score=18922;Name="3720472:L1MA3(LINE)" | bnd_52 | del_invers_2039_0/1485_0 |
| 20T 5 | 87635419 | 87635419 | TMEM161B-AS1 | ncRNA_intronic | 5q14.3 | Score=18922;Name="3720472:L1MA3(LINE)" | bnd_53 | del_invers_2039_0/1485_0 |
| 20T 5 | 87648860 | 87648860 | TMEM161B-AS1 | ncRNA_intronic | 5q14.3 | Score=2703;Name="3720490:L1M4(LINE)" | bnd_51 | del_invers_2039_0/1485_0 |
| 20T 5 | 87649338 | 87649338 | TMEM161B-AS1 | ncRNA_intronic | 5q14.3 | Score=2703;Name="3720490:L1M4(LINE)" | bnd_54 | del_invers_2039_0/1485_0 |
| 20T 6 | 12672311 | 12672311 | . | intergenic | 6p24.1 | Score=1371;Name="3909178:HERVH-int(LTR)" | bnd_30 | del_2348 |
| 20T 6 | 12686175 | 12686175 | . | intergenic | 6p24.1 | Score=1818;Name="3909210:L1MC5(LINE)" | bnd_29 | del_2348 |
| 20T 6 | 35858864 | 35858864 | SRPK1 | intronic | 6p21.31 | . | bnd_106 | transl_inter_7241 |
| 20T 6 | 70069615 | 70069615 | BAI3 | intronic | 6q13 | . | bnd_32 | del_1994 |
| 20T 6 | 70075093 | 70075093 | BAI3 | intronic | 6q13 | Score=881;Name="4007779:LTR33A_(LTR)" | bnd_31 | del_1994 |
| 20T 7 | 36302083 | 36302083 | EEPD1 | intronic | 7p14.2 | Score=4459;Name="4288941:MER50(LTR)" | bnd_34 | del_2379 |
| 20T 7 | 36332660 | 36332660 | EEPD1 | intronic | 7p14.2 | Score=572;Name="4289006:L1M5(LINE)" | bnd_33 | del_2379 |
| 20T 8 | 61116868 | 61116868 | CA8 | intronic | 8q12.1 | . | bnd_70 | transl_intra_1449 |
| 20T 8 | 61240829 | 61240829 | . | intergenic | 8q12.1 | Score=2292;Name="4606109:L1MA9(LINE)" | bnd_69 | transl_intra_1449 |
| 20T 8 | 61240834 | 61240834 | . | intergenic | 8q12.1 | Score=2292;Name="4606109:L1MA9(LINE)" | bnd_95 | transl_inter_4041 |
| 20T 8 | 61241308 | 61241308 | . | intergenic | 8q12.1 | Score=239;Name="4606110:L2b(LINE)" | bnd_93 | transl_inter_1122 |
| 20T 8 | 80124743 | 80124743 | . | intergenic | 8q21.13 | . | bnd_103 | transl_inter_7380 |
| 20T 8 | 105016510 | 105016510 | RIMS2 | intronic | 8q22.3 | Score=220;Name="4682299:L2c(LINE)" | bnd_105 | transl_inter_7241 |
| 20T 9 | 21961781 | 21961781 | . | intergenic | 9p21.3 | . | bnd_36 | del_6566 |

| | | | | | | | | |
|---|---|---|---|---|---|---|---|---|
| 20T 9 | 22104435 | 22104435 | CDKN2B-AS1 | ncRNA_intronic | 9p21.3 | . | bnd_35 | del_6566 |
| 20T 9 | 22104562 | 22104562 | CDKN2B-AS1 | ncRNA_intronic | 9p21.3 | . | bnd_38 | del_4654 |
| 20T 9 | 22123866 | 22123866 | . | intergenic | 9p21.3 | Score=699;Name="4796080:L3(LINE)" | bnd_37 | del_4654 |
| 20T 10 | 36491753 | 36491753 | . | intergenic | 10p11.21 | Score=10678;Name="499186:L1MC1(LINE)" | bnd_8 | del_4534 |
| 20T 10 | 38429162 | 38429162 | . | intergenic | 10p11.1 | Score=3448;Name="502808:L1MD3(LINE)" | bnd_7 | del_4534 |
| 20T 10 | 89645634 | 89645634 | PTEN | intronic | 10q23.31 | . | bnd_10 | del_1063 |
| 20T 10 | 89655346 | 89655346 | PTEN | intronic | 10q23.31 | . | bnd_9 | del_1063 |
| 20T 11 | 2542569 | 2542569 | KCNQ1 | intronic | 11p15.5 | . | bnd_12 | del_2494 |
| 20T 11 | 2793183 | 2793183 | KCNQ1 | intronic | 11p15.5 | . | bnd_11 | del_2494 |
| 20T 11 | 18726722 | 18726722 | IGSF22 | intronic | 11p15.1 | . | bnd_14 | del_2251 |
| 20T 11 | 18736120 | 18736120 | IGSF22 | exonic | 11p15.1 | . | bnd_13 | del_2251 |
| 20T 11 | 25426334 | 25426334 | . | intergenic | 11p14.3 | . | bnd_89 | transl_inter_7436 |
| 20T 11 | 67131644 | 67131644 | CLCF1,LOC100130987 | ncRNA_intronic | 11q13.2 | . | bnd_64 | transl_intra_3986 |
| 20T 11 | 67182110 | 67182110 | . | intergenic | 11q13.2 | Score=2259;Name="785946:AluSx(SINE)" | bnd_63 | transl_intra_3986 |
| 20T 12 | 6598294 | 6598294 | . | intergenic | 12p13.31 | Score=2049;Name="922504:AluJb(SINE)" | bnd_56 | transl_intra_902_0 |
| 20T 12 | 46055204 | 46055204 | . | intergenic | 12q12 | Score=1267;Name="984777:AluJb(SINE)" | bnd_55 | transl_intra_902_0 |
| 20T 12 | 46222024 | 46222024 | ARID2 | intronic | 12q12 | Score=2090;Name="985136:L1ME3A(LINE)" | bnd_74 | tandem_dup_3244 |
| 20T 12 | 46222363 | 46222363 | ARID2 | intronic | 12q12 | Score=2090;Name="985136:L1ME3A(LINE)" | bnd_16 | del_1648 |
| 20T 12 | 46270113 | 46270113 | ARID2 | intronic | 12q12 | Score=2439;Name="985240:AluSp(SINE)" | bnd_15 | del_1648 |
| 20T 12 | 46301849 | 46301849 | . | downstream | 12q12 | . | bnd_73 | tandem_dup_3244 |
| 20T 12 | 129683083 | 129683083 | TMEM132D | intronic | 12q24.33 | Score=4324;Name="1152886:L1MA7(LINE)" | bnd_18 | del_1601 |
| 20T 12 | 129684129 | 129684129 | TMEM132D | intronic | 12q24.33 | Score=2622;Name="1152889:THE1B(LTR)" | bnd_17 | del_1601 |
| 20T 13 | 40073282 | 40073282 | LHFP | intronic | 13q13.3 | Score=2127;Name="1197947:AluSx(SINE)" | bnd_92 | transl_inter_7172 |
| 20T 14 | 103330417 | 103330417 | TRAF3 | intronic | 14q32.32 | Score=6523;Name="1475547:L1MB5(LINE)" | bnd_94 | transl_inter_1122 |
| 20T 14 | 103455978 | 103455978 | CDC42BPB | intronic | 14q32.32 | Score=1997;Name="1475727:AluSp(SINE)" | bnd_96 | transl_inter_4041 |
| 20T 15 | 77315082 | 77315082 | PSTPIP1 | intronic | 15q24.3 | . | bnd_20 | del_6681 |
| 20T 15 | 77317896 | 77317896 | PSTPIP1 | exonic | 15q24.3 | . | bnd_19 | del_6681 |
| 20T 16 | 78001327 | 78001327 | VAT1L | intronic | 16q23.1 | . | bnd_98 | transl_inter_1554 |
| 20T 17 | 19166607 | 19166607 | EPN2 | intronic | 17p11.2 | . | bnd_58 | transl_intra_1459_0 |
| 20T 17 | 20929471 | 20929471 | USP22 | intronic | 17p11.2 | Score=1923;Name="1849478:AluSq2(SINE)" | bnd_57 | transl_intra_1459_0 |
| 20T 17 | 67792113 | 67792113 | AC003051.1 | ncRNA_intronic | 17q24.3 | . | bnd_50 | transl_intra_2250_0 |
| 20T 17 | 67792321 | 67792321 | AC003051.1 | ncRNA_intronic | 17q24.3 | . | bnd_48 | transl_intra_3800_0 |
| 20T 17 | 67792838 | 67792838 | AC003051.1 | ncRNA_intronic | 17q24.3 | Score=360;Name="1942809:L1ME4a(LINE)" | bnd_47 | transl_intra_3800_0 |
| 20T 17 | 67792845 | 67792845 | AC003051.1 | ncRNA_intronic | 17q24.3 | Score=360;Name="1942809:L1ME4a(LINE)" | bnd_49 | transl_intra_2250_0 |
| 20T 21 | 35648400 | 35648400 | . | intergenic | 21q22.11 | Score=290;Name="2811606:MIRc(SINE)" | bnd_100 | transl_inter_1539 |

| | | | | | | | | | |
|---|---|---|---|---|---|---|---|---|---|
| 20T | 21 | 36441721 | 36441721 | . | intergenic | 21q22.12 | . | bnd_102 | transl_inter_3598 |
| 20T | X | 8591143 | 8591143 | KAL1 | intronic | Xp22.31 | . | bnd_84 | tandem_dup_1565 |
| 20T | X | 8863909 | 8863909 | . | intergenic | Xp22.31 | Score=1714;Name="5008326:MLT1C(LTR)" | bnd_62 | transl_intra_1842_0 |
| 20T | X | 9455191 | 9455191 | TBL1X | intronic | Xp22.31 | Score=1549;Name="5009487:L1MB7(LINE)" | bnd_46 | del_ins_4717 |
| 20T | X | 9480481 | 9480481 | TBL1X | intronic | Xp22.31 | . | bnd_45 | del_ins_4717 |
| 20T | X | 25312390 | 25312390 | . | intergenic | Xp21.3 | . | bnd_86 | tandem_dup_1214 |
| 20T | X | 25325691 | 25325691 | . | intergenic | Xp21.3 | . | bnd_83 | tandem_dup_1565 |
| 20T | X | 25328138 | 25328138 | . | intergenic | Xp21.3 | Score=13682;Name="5039259:L1PA15(LINE)" | bnd_101 | transl_inter_3598 |
| 20T | X | 26077892 | 26077892 | . | intergenic | Xp21.3 | . | bnd_61 | transl_intra_1842_0 |
| 20T | X | 36349940 | 36349940 | CXorf30 | intronic | Xp21.1 | Score=2257;Name="5057922:L2(LINE)" | bnd_88 | tandem_dup_1301 |
| 20T | X | 36352237 | 36352237 | CXorf30 | intronic | Xp21.1 | Score=3959;Name="5057923:L2(LINE)" | bnd_85 | tandem_dup_1214 |
| 20T | X | 36580434 | 36580434 | . | intergenic | Xp21.1 | Score=6105;Name="5058285:Tigger3b(DNA)" | bnd_99 | transl_inter_1539 |
| 20T | X | 36581455 | 36581455 | . | intergenic | Xp21.1 | . | bnd_87 | tandem_dup_1301 |
| 20T | X | 74722795 | 74722795 | ZDHHC15 | intronic | Xq13.3 | Score=1503;Name="5121348:L1MA3(LINE)" | bnd_97 | transl_inter_1554 |
| 25T | 1 | 153175042 | 153175042 | . | upstream | 1q21.3 | . | bnd_326 | transl_intra_4332_0 |
| 25T | 1 | 153204367 | 153204367 | . | intergenic | 1q21.3 | . | bnd_56 | del_insod_5900_0/3093_0 |
| 25T | 1 | 153260373 | 153260373 | . | intergenic | 1q21.3 | Score=2311;Name="259986:AluSx1(SINE)" | bnd_2 | del_2162_0 |
| 25T | 1 | 153275989 | 153275989 | PGLYRP3 | intronic | 1q21.3 | Score=763;Name="260012:L2a(LINE)" | bnd_58 | del_insod_5900_0/3093_0 |
| 25T | 1 | 153407200 | 153407200 | . | intergenic | 1q21.3 | Score=1189;Name="260204:ERV3-16A3_I-int(LTR)" | bnd_100 | del_ins_2046_0 |
| 25T | 1 | 153609098 | 153609098 | CHTOP | exonic | 1q21.3 | . | bnd_76 | transl_intra_1697_0 |
| 25T | 1 | 155467083 | 155467083 | ASH1L | intronic | 1q22 | Score=1980;Name="264902:L1MB3(LINE)" | bnd_418 | tandem_dup_2715_0 |
| 25T | 1 | 156083664 | 156083664 | MIR7851,LMNA | ncRNA_intronic | 1q22 | . | bnd_99 | del_ins_2046_0 |
| 25T | 1 | 156653453 | 156653453 | . | intergenic | 1q23.1 | . | bnd_420 | tandem_dup_2179_0 |
| 25T | 1 | 163634938 | 163634938 | . | intergenic | 1q23.3 | . | bnd_248 | transl_intra_277_0 |
| 25T | 1 | 164484832 | 164484832 | . | intergenic | 1q23.3 | . | bnd_328 | transl_intra_1424_0 |
| 25T | 1 | 166861599 | 166861599 | . | intergenic | 1q24.1 | Score=2225;Name="285319:AluSx(SINE)" | bnd_325 | transl_intra_4332_0 |
| 25T | 1 | 166861718 | 166861718 | . | intergenic | 1q24.1 | Score=495;Name="285320:LTR33(LTR)" | bnd_4 | del_5050 |
| 25T | 1 | 166863996 | 166863996 | . | intergenic | 1q24.1 | Score=4810;Name="285325:L1MC1(LINE)" | bnd_3 | del_5050 |
| 25T | 1 | 169097904 | 169097904 | ATP1B1 | intronic | 1q24.2 | . | bnd_1 | del_2162_0 |
| 25T | 1 | 169495663 | 169495663 | F5 | intronic | 1q24.2 | Score=21;Name="290188:AT_rich(Low_complexity)" | bnd_417 | tandem_dup_2715_0 |
| 25T | 1 | 169535491 | 169535491 | F5 | intronic | 1q24.2 | . | bnd_57 | del_insod_5900_0/3093_0 |
| 25T | 1 | 169644308 | 169644308 | . | intergenic | 1q24.2 | Score=244;Name="290426:L2c(LINE)" | bnd_419 | tandem_dup_2179_0 |
| 25T | 1 | 170909382 | 170909382 | MROH9 | intronic | 1q24.3 | Score=29693;Name="292476:ERVL-B4-int(LTR)" | bnd_244 | del_4311_0 |
| 25T | 1 | 170910698 | 170910698 | MROH9 | intronic | 1q24.3 | Score=29693;Name="292476:ERVL-B4-int(LTR)" | bnd_422 | tandem_dup_3809_0 |
| 25T | 1 | 170911361 | 170911361 | MROH9 | intronic | 1q24.3 | Score=29693;Name="292476:ERVL-B4-int(LTR)" | bnd_243 | del_4311_0 |
| 25T | 1 | 173866480 | 173866480 | . | intergenic | 1q25.1 | . | bnd_421 | tandem_dup_3809_0 |

| | | | | | | | | |
|---|---|---|---|---|---|---|---|---|
| 25T | 1 | 174226510 | 174226510 | RABGAP1L | intronic | 1q25.1 | Score=1154;Name="298465:Kanga2a(DNA)" | bnd_55 | del_insod_5900_0/3093_0 |
| 25T | 1 | 174720150 | 174720150 | RABGAP1L | intronic | 1q25.1 | . | bnd_247 | transl_intra_277_0 |
| 25T | 1 | 177332708 | 177332708 | . | intergenic | 1q25.2 | . | bnd_327 | transl_intra_1424_0 |
| 25T | 1 | 178418400 | 178418400 | RASAL2 | intronic | 1q25.2 | . | bnd_486 | transl_inter_8411 |
| 25T | 1 | 178420853 | 178420853 | RASAL2 | splicing | 1q25.2 | . | bnd_424 | tandem_dup_2683 |
| 25T | 1 | 178676398 | 178676398 | . | intergenic | 1q25.2 | Score=2386;Name="306071:AluSx(SINE)" | bnd_423 | tandem_dup_2683 |
| 25T | 1 | 179710289 | 179710289 | . | intergenic | 1q25.2 | Score=3446;Name="308110:L1ME3B(LINE)" | bnd_245 | transl_intra_2639_0 |
| 25T | 1 | 179711887 | 179711887 | . | upstream | 1q25.2 | . | bnd_75 | transl_intra_1697_0 |
| 25T | 1 | 180355889 | 180355889 | ACBD6 | intronic | 1q25.3 | Score=4083;Name="309345:L1MDa(LINE)" | bnd_6 | del_1041 |
| 25T | 1 | 180727099 | 180727099 | XPR1 | intronic | 1q25.3 | Score=1418;Name="310096:Tigger4(DNA)" | bnd_5 | del_1041 |
| 25T | 1 | 153606413 | 153606413 | S100A13 | intronic | 1q21.3 | . | bnd_246 | transl_intra_2639_0 |
| 25T | 2 | 205246946 | 205246946 | . | intergenic | 2q33.3 | Score=6122;Name="2582124:L1PA10(LINE)" | bnd_518 | transl_inter_6444 |
| 25T | 2 | 210891808 | 210891808 | KANSL1L | intronic | 2q34 | . | bnd_495 | transl_inter_8088 |
| 25T | 2 | 214173664 | 214173664 | SPAG16 | intronic | 2q34 | Score=1383;Name="2596719:Tigger4b(DNA)" | bnd_520 | transl_inter_8112 |
| 25T | 3 | 150173344 | 150173344 | TSC22D2 | intronic | 3q25.1 | Score=330;Name="3169733:MIR3(SINE)" | bnd_511 | transl_inter_7906 |
| 25T | 4 | 47484489 | 47484489 | . | intergenic | 4p12 | Score=492;Name="3344285:(TTC)n(Simple_repeat)" | bnd_44 | del_ins_6323 |
| 25T | 4 | 47484908 | 47484908 | . | intergenic | 4p12 | Score=501;Name="3344286:MIRb(SINE)" | bnd_43 | del_ins_6323 |
| 25T | 4 | 153261003 | 153261003 | FBXW7 | intronic | 4q31.3 | Score=1816;Name="3515387:AluJo(SINE)" | bnd_513 | transl_inter_8469 |
| 25T | 5 | 428764 | 428764 | AHRR | intronic | 5p15.33 | Score=3274;Name="3580184:MER1A(DNA)" | bnd_232 | del_invers_167_0/1058_0 |
| 25T | 5 | 1018029 | 1018029 | NKD2 | intronic | 5p15.33 | . | bnd_236 | del_invers_246_0/119_0 |
| 25T | 5 | 1680657 | 1680657 | . | intergenic | 5p15.33 | Score=13312;Name="3581634:L1MA4A(LINE)" | bnd_46 | del_ins_376 |
| 25T | 5 | 3814900 | 3814900 | . | intergenic | 5p15.33 | Score=2016;Name="3584288:MLT1A-int(LTR)" | bnd_308 | transl_intra_143_0 |
| 25T | 5 | 4901021 | 4901021 | . | intergenic | 5p15.32 | Score=987;Name="3585679:Arthur1B(DNA)" | bnd_193 | del_inssu_155_0/557_0 |
| 25T | 5 | 4918374 | 4918374 | . | intergenic | 5p15.32 | Score=3902;Name="3585697:L1MB2(LINE)" | bnd_138 | del_inssd_800_0/863_0 |
| 25T | 5 | 4918887 | 4918887 | . | intergenic | 5p15.32 | Score=3902;Name="3585697:L1MB2(LINE)" | bnd_45 | del_ins_376 |
| 25T | 5 | 4930659 | 4930659 | . | intergenic | 5p15.32 | . | bnd_466 | tandem_dup_371_0 |
| 25T | 5 | 5224980 | 5224980 | ADAMTS16 | intronic | 5p15.32 | . | bnd_136 | del_inssd_800_0/863_0 |
| 25T | 5 | 5257975 | 5257975 | ADAMTS16 | intronic | 5p15.32 | Score=1659;Name="3586196:MER21C(LTR)" | bnd_240 | transl_intra_1522_0 |
| 25T | 5 | 5846589 | 5846589 | . | intergenic | 5p15.32 | . | bnd_400 | transl_intra_403_0 |
| 25T | 5 | 6039447 | 6039447 | . | intergenic | 5p15.32 | . | bnd_310 | transl_intra_527_0 |
| 25T | 5 | 6240600 | 6240600 | . | intergenic | 5p15.32 | Score=644;Name="3587654:L1M5(LINE)" | bnd_468 | tandem_dup_278_0 |
| 25T | 5 | 6486029 | 6486029 | UBE2QL1 | intronic | 5p15.31 | . | bnd_465 | tandem_dup_371_0 |
| 25T | 5 | 7464047 | 7464047 | ADCY2 | intronic | 5p15.31 | . | bnd_470 | tandem_dup_168_0 |
| 25T | 5 | 7485433 | 7485433 | ADCY2 | intronic | 5p15.31 | . | bnd_237 | del_invers_246_0/119_0 |
| 25T | 5 | 7505158 | 7505158 | ADCY2 | intronic | 5p15.31 | Score=2080;Name="3589663:AluJb(SINE)" | bnd_235 | del_invers_246_0/119_0 |
| 25T | 5 | 7668774 | 7668774 | ADCY2 | intronic | 5p15.31 | . | bnd_72 | del_insod_354_0/811_0 |

| | | | | | | | | |
|---|---|---|---|---|---|---|---|---|
| 25T 5 | 7668989 | 7668989 | ADCY2 | intronic | 5p15.31 | . | bnd_402 | transl_intra_284_0 |
| 25T 5 | 7854753 | 7854753 | . | intergenic | 5p15.31 | Score=2185;Name="3590122:THE1A(LTR)" | bnd_74 | del_insod_354_0/811_0 |
| 25T 5 | 9229055 | 9229055 | SEMA5A | intronic | 5p15.31 | . | bnd_192 | tandem_dup_767_0 |
| 25T 5 | 9232031 | 9232031 | SEMA5A | intronic | 5p15.31 | . | bnd_30 | del_279_0 |
| 25T 5 | 9391613 | 9391613 | SEMA5A | intronic | 5p15.31 | Score=614;Name="3592291:L2b(LINE)" | bnd_401 | transl_intra_284_0 |
| 25T 5 | 9834937 | 9834937 | LOC285692 | ncRNA_intronic | 5p15.2 | Score=249;Name="3592946:UCON2(Unknown)" | bnd_472 | tandem_dup_1585_0 |
| 25T 5 | 9835603 | 9835603 | LOC285692 | ncRNA_intronic | 5p15.2 | . | bnd_312 | transl_intra_1510_0 |
| 25T 5 | 9837848 | 9837848 | LOC285692 | ncRNA_intronic | 5p15.2 | Score=284;Name="3592953:L2a(LINE)" | bnd_311 | transl_intra_1510_0 |
| 25T 5 | 9915343 | 9915343 | . | intergenic | 5p15.2 | . | bnd_404 | transl_intra_1038 |
| 25T 5 | 10039917 | 10039917 | . | intergenic | 5p15.2 | . | bnd_137 | del_inssd_800_0/863_0 |
| 25T 5 | 10122391 | 10122391 | . | intergenic | 5p15.2 | Score=1645;Name="3593386:Charlie7(DNA)" | bnd_32 | del_753 |
| 25T 5 | 10122554 | 10122554 | . | intergenic | 5p15.2 | Score=1645;Name="3593386:Charlie7(DNA)" | bnd_97 | del_insou_699_0/399_0 |
| 25T 5 | 10601348 | 10601348 | ANKRD33B | intronic | 5p15.2 | . | bnd_406 | transl_intra_172_0 |
| 25T 5 | 10603631 | 10603631 | ANKRD33B | intronic | 5p15.2 | Score=2177;Name="3594386:AluSx(SINE)" | bnd_474 | tandem_dup_1087_0 |
| 25T 5 | 10603890 | 10603890 | ANKRD33B | intronic | 5p15.2 | . | bnd_471 | tandem_dup_1585_0 |
| 25T 5 | 10604585 | 10604585 | ANKRD33B | intronic | 5p15.2 | Score=2218;Name="3594389:AluSz(SINE)" | bnd_239 | transl_intra_1522_0 |
| 25T 5 | 10605013 | 10605013 | ANKRD33B | intronic | 5p15.2 | Score=2238;Name="3594393:MLT1F(LTR)" | bnd_314 | transl_intra_3080_0 |
| 25T 5 | 10609289 | 10609289 | ANKRD33B | intronic | 5p15.2 | Score=1008;Name="3594404:MER33(DNA)" | bnd_408 | transl_intra_180_0 |
| 25T 5 | 10610915 | 10610915 | ANKRD33B | intronic | 5p15.2 | Score=2153;Name="3594412:AluSx1(SINE)" | bnd_95 | del_insou_699_0/399_0 |
| 25T 5 | 10679920 | 10679920 | DAP | UTR3 | 5p15.2 | . | bnd_316 | transl_intra_357_0 |
| 25T 5 | 11017880 | 11017880 | CTNND2 | intronic | 5p15.2 | . | bnd_410 | transl_intra_95 |
| 25T 5 | 11092465 | 11092465 | CTNND2 | intronic | 5p15.2 | . | bnd_242 | transl_intra_2922_0 |
| 25T 5 | 11092567 | 11092567 | CTNND2 | intronic | 5p15.2 | . | bnd_241 | transl_intra_2922_0 |
| 25T 5 | 11093078 | 11093078 | CTNND2 | intronic | 5p15.2 | . | bnd_318 | transl_intra_285_0 |
| 25T 5 | 11093357 | 11093357 | CTNND2 | intronic | 5p15.2 | . | bnd_313 | transl_intra_3080_0 |
| 25T 5 | 11095364 | 11095364 | CTNND2 | intronic | 5p15.2 | . | bnd_317 | transl_intra_285_0 |
| 25T 5 | 12104878 | 12104878 | . | intergenic | 5p15.2 | . | bnd_73 | del_insod_354_0/811_0 |
| 25T 5 | 12107314 | 12107314 | . | intergenic | 5p15.2 | . | bnd_320 | transl_intra_1209_0 |
| 25T 5 | 12108045 | 12108045 | . | intergenic | 5p15.2 | . | bnd_190 | tandem_dup_461_0 |
| 25T 5 | 13036384 | 13036384 | . | intergenic | 5p15.2 | Score=306;Name="3598141:(TG)n(Simple_repeat)" | bnd_71 | del_insod_354_0/811_0 |
| 25T 5 | 13348436 | 13348436 | . | intergenic | 5p15.2 | . | bnd_412 | transl_intra_355 |
| 25T 5 | 13349493 | 13349493 | . | intergenic | 5p15.2 | . | bnd_414 | transl_intra_1007_0 |
| 25T 5 | 13465970 | 13465970 | . | intergenic | 5p15.2 | Score=4353;Name="3598829:L1M4(LINE)" | bnd_48 | del_ins_1515 |
| 25T 5 | 13949057 | 13949057 | . | intergenic | 5p15.2 | Score=270;Name="3599615:MamRep1879(DNA)" | bnd_399 | transl_intra_403_0 |
| 25T 5 | 14568804 | 14568804 | . | intergenic | 5p15.2 | Score=2104;Name="3600564:AluSx4(SINE)" | bnd_47 | del_ins_1515 |
| 25T 5 | 14695013 | 14695013 | FAM105B | UTR3 | 5p15.2 | . | bnd_189 | tandem_dup_461_0 |
| 25T 5 | 15593794 | 15593794 | FBXL7 | intronic | 5p15.1 | . | bnd_233 | del_invers_167_0/1058_0 |
| 25T 5 | 15644473 | 15644473 | FBXL7 | intronic | 5p15.1 | Score=2286;Name="3602317:AluSp(SINE)" | bnd_319 | transl_intra_1209_0 |
| 25T 5 | 15846739 | 15846739 | FBXL7 | intronic | 5p15.1 | Score=1622;Name="3602656:MER57F(LTR)" | bnd_238 | del_invers_246_0/119_0 |
| 25T 5 | 15888177 | 15888177 | FBXL7 | intronic | 5p15.1 | . | bnd_231 | del_invers_167_0/1058_0 |
| 25T 5 | 16615004 | 16615004 | FAM134B | intronic | 5p15.1 | . | bnd_31 | del_753 |

| | | | | | | | | |
|---|---|---|---|---|---|---|---|---|
| 25T 5 | 16617930 | 16617930 | RP11-260E18.1 | ncRNA_intronic | 5p15.1 | . | bnd_309 | transl_intra_527_0 |
| 25T 5 | 16618707 | 16618707 | RP11-260E18.1 | ncRNA_intronic | 5p15.1 | . | bnd_50 | del_ins_491 |
| 25T 5 | 16622665 | 16622665 | RP11-260E18.1 | ncRNA_intronic | 5p15.1 | . | bnd_49 | del_ins_491 |
| 25T 5 | 16888031 | 16888031 | MYO10 | intronic | 5p15.1 | . | bnd_135 | del_inssd_800_0/863_0 |
| 25T 5 | 17008204 | 17008204 | . | intergenic | 5p15.1 | Score=2299;Name="3604779:AluSq(SINE)" | bnd_476 | tandem_dup_1037_0 |
| 25T 5 | 17098938 | 17098938 | . | intergenic | 5p15.1 | Score=2240;Name="3605021:AluSx1(SINE)" | bnd_52 | del_ins_1384 |
| 25T 5 | 18440973 | 18440973 | . | intergenic | 5p14.3 | Score=811;Name="3607248:AluJr(SINE)" | bnd_413 | transl_intra_1007_0 |
| 25T 5 | 18519491 | 18519491 | . | intergenic | 5p14.3 | Score=1240;Name="3607377:AluJo(SINE)" | bnd_195 | del_inssu_155_0/557_0 |
| 25T 5 | 18605580 | 18605580 | . | intergenic | 5p14.3 | . | bnd_403 | transl_intra_1038 |
| 25T 5 | 20315647 | 20315647 | . | intergenic | 5p14.3 | Score=715;Name="3610380:L2a(LINE)" | bnd_29 | del_279_0 |
| 25T 5 | 20424756 | 20424756 | . | intergenic | 5p14.3 | Score=2192;Name="3610553:AluSz6(SINE)" | bnd_191 | tandem_dup_767_0 |
| 25T 5 | 21206224 | 21206224 | . | intergenic | 5p14.3 | . | bnd_51 | del_ins_1384 |
| 25T 5 | 21218785 | 21218785 | . | intergenic | 5p14.3 | . | bnd_411 | transl_intra_355 |
| 25T 5 | 21248841 | 21248841 | . | intergenic | 5p14.3 | . | bnd_475 | tandem_dup_1037_0 |
| 25T 5 | 22042906 | 22042906 | CDH12 | intronic | 5p14.3 | Score=1674;Name="3613326:AluJb(SINE)" | bnd_322 | transl_intra_608_0 |
| 25T 5 | 23769097 | 23769097 | . | intergenic | 5p14.2 | Score=2100;Name="3616255:AluSx(SINE)" | bnd_324 | transl_intra_2567_0 |
| 25T 5 | 23770996 | 23770996 | . | intergenic | 5p14.2 | Score=1476;Name="3616261:MLT1B(LTR)" | bnd_323 | transl_intra_2567_0 |
| 25T 5 | 23771286 | 23771286 | . | intergenic | 5p14.2 | . | bnd_405 | transl_intra_172_0 |
| 25T 5 | 23771671 | 23771671 | . | intergenic | 5p14.2 | Score=286;Name="3616263:L1MEg1(LINE)" | bnd_473 | tandem_dup_1087_0 |
| 25T 5 | 24582284 | 24582284 | CDH10 | intronic | 5p14.2 | Score=528;Name="3617640:L1ME3B(LINE)" | bnd_478 | tandem_dup_212 |
| 25T 5 | 24623076 | 24623076 | CDH10 | intronic | 5p14.1 | . | bnd_477 | tandem_dup_212 |
| 25T 5 | 24623120 | 24623120 | CDH10 | intronic | 5p14.1 | . | bnd_480 | tandem_dup_58 |
| 25T 5 | 24624754 | 24624754 | CDH10 | intronic | 5p14.1 | . | bnd_315 | transl_intra_357_0 |
| 25T 5 | 26344510 | 26344510 | . | intergenic | 5p14.1 | . | bnd_409 | transl_intra_95 |
| 25T 5 | 26359044 | 26359044 | . | intergenic | 5p14.1 | Score=10605;Name="3620702:L1PA10(LINE)" | bnd_479 | tandem_dup_58 |
| 25T 5 | 26439128 | 26439128 | . | intergenic | 5p14.1 | Score=15376;Name="3620826:L1M1(LINE)" | bnd_482 | tandem_dup_783_0 |
| 25T 5 | 26451833 | 26451833 | . | intergenic | 5p14.1 | . | bnd_469 | tandem_dup_168_0 |
| 25T 5 | 27859936 | 27859936 | . | intergenic | 5p14.1 | Score=7239;Name="3623254:MER4-int(LTR)" | bnd_467 | tandem_dup_278_0 |
| 25T 5 | 28061371 | 28061371 | . | intergenic | 5p14.1 | . | bnd_416 | transl_intra_224_0 |
| 25T 5 | 33290204 | 33290204 | . | intergenic | 5p13.3 | . | bnd_415 | transl_intra_224_0 |
| 25T 5 | 33388144 | 33388144 | . | intergenic | 5p13.3 | . | bnd_481 | tandem_dup_783_0 |
| 25T 5 | 33824993 | 33824993 | ADAMTS12 | intronic | 5p13.2 | Score=362;Name="3633661:MLT1J2(LTR)" | bnd_194 | del_inssu_155_0/557_0 |
| 25T 5 | 34594424 | 34594424 | . | intergenic | 5p13.2 | Score=2317;Name="3635036:MSTC(LTR)" | bnd_484 | tandem_dup_484_0 |
| 25T 5 | 34794015 | 34794015 | RAI14 | intronic | 5p13.2 | . | bnd_307 | transl_intra_143_0 |
| 25T 5 | 35530951 | 35530951 | . | intergenic | 5p13.2 | . | bnd_140 | tandem_dup_389_0 |
| 25T 5 | 35928944 | 35928944 | CAPSL | intronic | 5p13.2 | Score=180;Name="3637375:MER5B(DNA)" | bnd_197 | del_inssu_187_0/1306_0 |
| 25T 5 | 35962306 | 35962306 | UGT3A1 | UTR3 | 5p13.2 | Score=16934;Name="3637423:HUERS-P2-int(LTR)" | bnd_96 | del_insou_699_0/399_0 |

| | | | | | | | | | |
|---|---|---|---|---|---|---|---|---|---|
| 25T | 5 | 36635403 | 36635403 | SLC1A3 | intronic | 5p13.2 | Score=366;Name="3638633:MIRb(SINE)" | bnd_98 | del_insou_699_0/399_0 |
| 25T | 5 | 37918039 | 37918039 | . | intergenic | 5p13.2 | . | bnd_196 | del_inssu_155_0/557_0 |
| 25T | 5 | 38473622 | 38473622 | . | intergenic | 5p13.1 | . | bnd_234 | del_invers_167_0/1058_0 |
| 25T | 5 | 38564521 | 38564521 | LIFR,LIFR-AS1 | ncRNA_intronic | 5p13.1 | Score=1844;Name="3642337:AluJo(SINE)" | bnd_321 | transl_intra_608_0 |
| 25T | 5 | 38945886 | 38945886 | RICTOR | intronic | 5p13.1 | . | bnd_199 | del_inssu_187_0/1306_0 |
| 25T | 5 | 39628684 | 39628684 | . | intergenic | 5p13.1 | Score=679;Name="3644107:L3(LINE)" | bnd_139 | tandem_dup_389_0 |
| 25T | 5 | 45195352 | 45195352 | . | intergenic | 5p12 | . | bnd_407 | transl_intra_180_0 |
| 25T | 5 | 45270572 | 45270572 | HCN1 | intronic | 5p12 | . | bnd_198 | del_inssu_187_0/1306_0 |
| 25T | 5 | 45271513 | 45271513 | HCN1 | intronic | 5p12 | . | bnd_200 | del_inssu_187_0/1306_0 |
| 25T | 5 | 45307805 | 45307805 | HCN1 | intronic | 5p12 | Score=3939;Name="3653279:L1MC3(LINE)" | bnd_483 | tandem_dup_484_0 |
| 25T | 5 | 83113683 | 83113683 | . | intergenic | 5q14.3 | . | bnd_509 | transl_inter_5436 |
| 25T | 5 | 84859740 | 84859740 | . | intergenic | 5q14.3 | . | bnd_493 | transl_inter_8461 |
| 25T | 6 | 24319469 | 24319469 | DCDC2 | intronic | 6p22.3 | Score=5042;Name="3930873:L1MC4a(LINE)" | bnd_517 | transl_inter_6444 |
| 25T | 6 | 79139590 | 79139590 | . | intergenic | 6q14.1 | Score=5702;Name="4022433:L1MC1(LINE)" | bnd_519 | transl_inter_8112 |
| 25T | 7 | 94332396 | 94332396 | . | intergenic | 7q21.3 | Score=4532;Name="4390653:L1MC(LINE)" | bnd_499 | transl_inter_7907 |
| 25T | 8 | 79081398 | 79081398 | . | intergenic | 8q21.12 | Score=1207;Name="4636459:L1M5(LINE)" | bnd_34 | del_2174 |
| 25T | 8 | 79082873 | 79082873 | . | intergenic | 8q21.12 | Score=507;Name="4636462:Charlie1b(DNA)" | bnd_33 | del_2174 |
| 25T | 8 | 88526501 | 88526501 | . | intergenic | 8q21.3 | Score=1509;Name="4652389:THE1D(LTR)" | bnd_523 | transl_inter_8326 |
| 25T | 8 | 143676966 | 143676966 | . | intergenic | 8q24.3 | . | bnd_54 | del_ins_5181 |
| 25T | 8 | 143677346 | 143677346 | . | intergenic | 8q24.3 | . | bnd_53 | del_ins_5181 |
| 25T | 10 | 23496043 | 23496043 | C10orf115 | ncRNA_intronic | 10p12.2 | . | bnd_488 | transl_inter_1434 |
| 25T | 10 | 88239006 | 88239006 | WAPAL | intronic | 10q23.2 | Score=2310;Name="585796:AluSq(SINE)" | bnd_490 | transl_inter_7983 |
| 25T | 10 | 88423803 | 88423803 | OPN4 | intronic | 10q23.2 | . | bnd_492 | transl_inter_8513 |
| 25T | 10 | 121317243 | 121317243 | . | intergenic | 10q26.11 | Score=1922;Name="648781:AluSx1(SINE)" | bnd_494 | transl_inter_8461 |
| 25T | 11 | 26434486 | 26434486 | ANO3 | intronic | 11p14.2 | . | bnd_491 | transl_inter_8513 |
| 25T | 11 | 56249070 | 56249070 | . | intergenic | 11q12.1 | . | bnd_496 | transl_inter_8088 |
| 25T | 11 | 73566508 | 73566508 | MRPL48 | intronic | 11q13.4 | Score=443;Name="799212:L2a(LINE)" | bnd_498 | transl_inter_8300 |
| 25T | 11 | 117675301 | 117675301 | . | intergenic | 11q23.3 | Score=653;Name="880242:L3(LINE)" | bnd_500 | transl_inter_7907 |
| 25T | 12 | 132188892 | 132188892 | . | intergenic | 12q24.33 | Score=230;Name="1157469:(CAAA)n(Simple_repeat)" | bnd_489 | transl_inter_7983 |
| 25T | 13 | 41373926 | 41373926 | TPTE2P5,SLC25A15 | ncRNA_exonic | 13q14.11 | . | bnd_487 | transl_inter_1434 |
| 25T | 13 | 94762707 | 94762707 | GPC6 | intronic | 13q31.3 | . | bnd_485 | transl_inter_8411 |
| 25T | 13 | 108854069 | 108854069 | . | intergenic | 13q33.3 | . | bnd_426 | tandem_dup_1160 |
| 25T | 13 | 109068670 | 109068670 | . | intergenic | 13q33.3 | Score=2164;Name="1314139:THE1B(LTR)" | bnd_502 | transl_inter_6881 |
| 25T | 13 | 109068825 | 109068825 | . | intergenic | 13q33.3 | Score=2164;Name="1314139:THE1B(LTR)" | bnd_504 | transl_inter_1168 |
| 25T | 13 | 109069743 | 109069743 | . | intergenic | 13q33.3 | Score=2443;Name="1314141:L1PA10(LINE)" | bnd_330 | transl_intra_2704 |
| 25T | 13 | 109070226 | 109070226 | . | intergenic | 13q33.3 | Score=2080;Name="1314142:L1PA15(LINE)" | bnd_425 | tandem_dup_1160 |

| | | | | | | | | |
|---|---|---|---|---|---|---|---|---|
| 25T | 13 | 109070496 | 109070496 | . | intergenic | 13q33.3 | Score=2080;Name="1314142:L1PA15(LINE)" | bnd_329 | transl_intra_2704 |
| 25T | 13 | 109070615 | 109070615 | . | intergenic | 13q33.3 | Score=286;Name="1314143:L1ME3(LINE)" | bnd_506 | transl_inter_6428 |
| 25T | 13 | 109188448 | 109188448 | . | intergenic | 13q33.3 | . | bnd_508 | transl_inter_2138 |
| 25T | 14 | 39802544 | 39802544 | CTAGE5 | intronic | 14q21.1 | Score=2584;Name="1357472:L1MEb(LINE)" | bnd_510 | transl_inter_5436 |
| 25T | 14 | 80095047 | 80095047 | NRXN3 | intronic | 14q31.1 | . | bnd_512 | transl_inter_7906 |
| 25T | 15 | 92432115 | 92432115 | SLCO3A1 | intronic | 15q26.1 | Score=1388;Name="1617719:AluJb(SINE)" | bnd_514 | transl_inter_8469 |
| 25T | 16 | 59084830 | 59084830 | . | intergenic | 16q21 | . | bnd_516 | transl_inter_7994 |
| 25T | 19 | 15724104 | 15724104 | . | intergenic | 19p13.12 | . | bnd_332 | transl_intra_1871 |
| 25T | 19 | 15725642 | 15725642 | . | upstream | 19p13.12 | . | bnd_331 | transl_intra_1871 |
| 25T | 19 | 15750616 | 15750616 | . | intergenic | 19p13.12 | . | bnd_501 | transl_inter_6881 |
| 25T | 19 | 15751935 | 15751935 | CYP4F3 | intronic | 19p13.12 | . | bnd_503 | transl_inter_1168 |
| 25T | 19 | 16906694 | 16906694 | NWD1 | intronic | 19p13.11 | Score=3131;Name="2142909:LTR28(LTR)" | bnd_507 | transl_inter_2138 |
| 25T | 19 | 17028702 | 17028702 | CPAMD8 | intronic | 19p13.11 | Score=1630;Name="2143224:MER2B(DNA)" | bnd_497 | transl_inter_8300 |
| 25T | 19 | 20834899 | 20834899 | ZNF626 | intronic | 19p12 | . | bnd_505 | transl_inter_6428 |
| 25T | 20 | 712943 | 712943 | . | intergenic | 20p13 | . | bnd_428 | tandem_dup_525_0 |
| 25T | 20 | 1094848 | 1094848 | PSMF1 | intronic | 20p13 | Score=987;Name="2651056:LTR16C(LTR)" | bnd_104 | del_inssd_71_0/2634_0 |
| 25T | 20 | 1130738 | 1130738 | PSMF1 | intronic | 20p13 | Score=1181;Name="2651130:AluJr(SINE)" | bnd_250 | transl_intra_1002_0 |
| 25T | 20 | 1327138 | 1327138 | SDCBP2-AS1,FKBP1A-SDCBP2 | ncRNA_intronic | 20p13 | Score=7248;Name="2651553:L1MC2(LINE)" | bnd_334 | transl_intra_550 |
| 25T | 20 | 1392423 | 1392423 | . | intergenic | 20p13 | Score=4691;Name="2651676:L1M4(LINE)" | bnd_336 | transl_intra_3287 |
| 25T | 20 | 1437188 | 1437188 | NSFL1C | intronic | 20p13 | . | bnd_202 | transl_intra_311_0 |
| 25T | 20 | 1437269 | 1437269 | NSFL1C | intronic | 20p13 | . | bnd_338 | transl_intra_140_0 |
| 25T | 20 | 1437590 | 1437590 | NSFL1C | intronic | 20p13 | . | bnd_204 | del_invers_421_0/19_0 |
| 25T | 20 | 2470268 | 2470268 | ZNF343 | intronic | 20p13 | Score=649;Name="2654095:MLT1I(LTR)" | bnd_102 | del_inssd_71_0/2634_0 |
| 25T | 20 | 2888767 | 2888767 | PTPRA | intronic | 20p13 | Score=7606;Name="2655043:Cheshire(DNA)" | bnd_252 | transl_intra_170_0 |
| 25T | 20 | 3588465 | 3588465 | ATRN | intronic | 20p13 | Score=7101;Name="2656703:L1MA4(LINE)" | bnd_430 | tandem_dup_54_0 |
| 25T | 20 | 4542782 | 4542782 | . | intergenic | 20p13 | Score=1505;Name="2658750:MER21C(LTR)" | bnd_172 | tandem_dup_98_0 |
| 25T | 20 | 4594003 | 4594003 | . | intergenic | 20p13 | . | bnd_251 | transl_intra_170_0 |
| 25T | 20 | 4595133 | 4595133 | . | intergenic | 20p13 | . | bnd_159 | del_inssu_888_0/1782_0 |
| 25T | 20 | 5400626 | 5400626 | . | intergenic | 20p12.3 | Score=2518;Name="2660642:MER34B(LTR)" | bnd_340 | transl_intra_397_0 |
| 25T | 20 | 5448709 | 5448709 | . | intergenic | 20p12.3 | Score=3437;Name="2660743:L1MB3(LINE)" | bnd_342 | transl_intra_103 |
| 25T | 20 | 5635926 | 5635926 | . | intergenic | 20p12.3 | Score=2291;Name="2661174:AluSc(SINE)" | bnd_141 | del_inssu_2996_0/138_0 |
| 25T | 20 | 5736877 | 5736877 | C20orf196 | intronic | 20p12.3 | Score=853;Name="2661470:FLAM_C(SINE)" | bnd_344 | transl_intra_3864 |
| 25T | 20 | 5738477 | 5738477 | C20orf196 | intronic | 20p12.3 | Score=2356;Name="2661476:AluSq(SINE)" | bnd_343 | transl_intra_3864 |
| 25T | 20 | 5822548 | 5822548 | C20orf196 | intronic | 20p12.3 | . | bnd_8 | del_3420 |
| 25T | 20 | 5828883 | 5828883 | C20orf196 | intronic | 20p12.3 | . | bnd_254 | transl_intra_4316_0 |
| 25T | 20 | 5971327 | 5971327 | MCM8 | intronic | 20p12.3 | . | bnd_103 | del_inssd_71_0/2634_0 |

| | | | | | | | | |
|---|---|---|---|---|---|---|---|---|
| 25T 20 | 6315041 | 6315041 | . | intergenic | 20p12.3 | Score=628;Name="2662662:L1MEe(LINE)" | bnd_346 | transl_intra_1971 |
| 25T 20 | 6334675 | 6334675 | . | intergenic | 20p12.3 | . | bnd_256 | transl_intra_237_0 |
| 25T 20 | 6534579 | 6534579 | . | intergenic | 20p12.3 | Score=9458;Name="2663028:L1MC1(LINE)" | bnd_89 | del_insou_708_0/145_0 |
| 25T 20 | 6857484 | 6857484 | . | intergenic | 20p12.3 | Score=194;Name="2663578:L2a(LINE)" | bnd_143 | del_inssu_2996_0/138_0 |
| 25T 20 | 6977079 | 6977079 | . | intergenic | 20p12.3 | . | bnd_348 | transl_intra_685 |
| 25T 20 | 6991790 | 6991790 | . | intergenic | 20p12.3 | . | bnd_87 | del_insou_708_0/145_0 |
| 25T 20 | 7000729 | 7000729 | . | intergenic | 20p12.3 | Score=1531;Name="2663821:AluJr(SINE)" | bnd_79 | del_insou_2754_0/144_0 |
| 25T 20 | 7134743 | 7134743 | . | intergenic | 20p12.3 | . | bnd_429 | tandem_dup_54_0 |
| 25T 20 | 7137425 | 7137425 | . | intergenic | 20p12.3 | . | bnd_106 | tandem_dup_111_0 |
| 25T 20 | 7203617 | 7203617 | . | intergenic | 20p12.3 | Score=1834;Name="2664151:AluSx(SINE)" | bnd_258 | transl_intra_2235_0 |
| 25T 20 | 7217594 | 7217594 | . | intergenic | 20p12.3 | Score=1784;Name="2664172:ERVL-E-int(LTR)" | bnd_350 | transl_intra_2355 |
| 25T 20 | 7262270 | 7262270 | . | intergenic | 20p12.3 | Score=1219;Name="2664242:L1MB8(LINE)" | bnd_77 | del_insou_2754_0/144_0 |
| 25T 20 | 7332700 | 7332700 | . | intergenic | 20p12.3 | . | bnd_260 | transl_intra_1549_0 |
| 25T 20 | 7923546 | 7923546 | . | intergenic | 20p12.3 | Score=2423;Name="2665348:AluSc(SINE)" | bnd_36 | del_ins_1366_0 |
| 25T 20 | 8244243 | 8244243 | PLCB1 | intronic | 20p12.3 | Score=6974;Name="2665841:Tigger3b(DNA)" | bnd_352 | transl_intra_1724 |
| 25T 20 | 8244416 | 8244416 | PLCB1 | intronic | 20p12.3 | Score=6974;Name="2665841:Tigger3b(DNA)" | bnd_161 | del_inssu_888_0/1782_0 |
| 25T 20 | 8324144 | 8324144 | PLCB1 | intronic | 20p12.3 | . | bnd_142 | del_inssu_2996_0/138_0 |
| 25T 20 | 8537540 | 8537540 | PLCB1 | intronic | 20p12.3 | Score=4100;Name="2666373:L1MB3(LINE)" | bnd_210 | transl_intra_432_0 |
| 25T 20 | 9051461 | 9051461 | PLCB4 | intronic | 20p12.3 | . | bnd_432 | tandem_dup_738_0 |
| 25T 20 | 9072250 | 9072250 | PLCB4 | intronic | 20p12.3 | Score=431;Name="2667423:MIRb(SINE)" | bnd_262 | transl_intra_975_0 |
| 25T 20 | 9079283 | 9079283 | PLCB4 | intronic | 20p12.3 | Score=557;Name="2667429:(TA)n(Simple_repeat)" | bnd_144 | del_inssu_2996_0/138_0 |
| 25T 20 | 9145539 | 9145539 | PLCB4 | intronic | 20p12.3 | . | bnd_427 | tandem_dup_525_0 |
| 25T 20 | 9303705 | 9303705 | PLCB4 | intronic | 20p12.2 | Score=740;Name="2667825:L2a(LINE)" | bnd_354 | transl_intra_3403_0 |
| 25T 20 | 9352390 | 9352390 | PLCB4 | intronic | 20p12.2 | . | bnd_145 | del_inssu_3454_0/69_0 |
| 25T 20 | 9813492 | 9813492 | PAK7 | intronic | 20p12.2 | Score=828;Name="2668729:L1ME4a(LINE)" | bnd_434 | tandem_dup_551_0 |
| 25T 20 | 9924706 | 9924706 | . | intergenic | 20p12.2 | Score=1963;Name="2668961:MSTA(LTR)" | bnd_436 | tandem_dup_859_0 |
| 25T 20 | 9928654 | 9928654 | . | intergenic | 20p12.2 | Score=9663;Name="2668967:MER57A-int(LTR)" | bnd_264 | transl_intra_504_0 |
| 25T 20 | 10223528 | 10223528 | SNAP25 | intronic | 20p12.2 | . | bnd_339 | transl_intra_397_0 |
| 25T 20 | 10298748 | 10298748 | . | intergenic | 20p12.2 | Score=1316;Name="2669641:LTR33(LTR)" | bnd_212 | del_invers_49_0/502_0 |
| 25T 20 | 10410922 | 10410922 | MKKS | intronic | 20p12.2 | Score=656;Name="2669882:MIR(SINE)" | bnd_147 | del_inssu_3454_0/69_0 |
| 25T 20 | 10416434 | 10416434 | SLX4IP | intronic | 20p12.2 | . | bnd_438 | tandem_dup_582_0 |
| 25T 20 | 10502028 | 10502028 | SLX4IP | intronic | 20p12.2 | Score=425;Name="2670080:HAL1(LINE)" | bnd_209 | transl_intra_432_0 |
| 25T 20 | 10699973 | 10699973 | . | intergenic | 20p12.2 | . | bnd_353 | transl_intra_3403_0 |
| 25T 20 | 11289262 | 11289262 | . | intergenic | 20p12.2 | Score=3795;Name="2671337:L1MB7(LINE)" | bnd_149 | del_inssu_2240_0/1450_0 |

| | | | | | | | | | |
|---|---|---|---|---|---|---|---|---|---|
| 25T | 20 | 11290332 | 11290332 | . | intergenic | 20p12.2 | Score=3795;Name="2671337:L1MB7(LINE)" | bnd_266 | transl_intra_2017_0 |
| 25T | 20 | 11890413 | 11890413 | BTBD3 | intronic | 20p12.2 | . | bnd_110 | del_inssd_169_0/884_0 |
| 25T | 20 | 12234265 | 12234265 | . | intergenic | 20p12.1 | Score=899;Name="2673009:MLT1E1A(LTR)" | bnd_356 | transl_intra_254 |
| 25T | 20 | 12336359 | 12336359 | . | intergenic | 20p12.1 | . | bnd_216 | del_invers_196_0/146_0 |
| 25T | 20 | 12660750 | 12660750 | . | intergenic | 20p12.1 | . | bnd_358 | transl_intra_207 |
| 25T | 20 | 12689677 | 12689677 | . | intergenic | 20p12.1 | Score=1674;Name="2673701:L1MC3(LINE)" | bnd_268 | transl_intra_298_0 |
| 25T | 20 | 12821283 | 12821283 | . | intergenic | 20p12.1 | Score=416;Name="2673936:L1ME3A(LINE)" | bnd_440 | tandem_dup_1956 |
| 25T | 20 | 13172279 | 13172279 | . | intergenic | 20p12.1 | Score=5836;Name="2674535:L1MA5(LINE)" | bnd_439 | tandem_dup_1956 |
| 25T | 20 | 13173964 | 13173964 | . | intergenic | 20p12.1 | Score=1364;Name="2674541:L1MCb(LINE)" | bnd_360 | transl_intra_1647 |
| 25T | 20 | 13588398 | 13588398 | TASP1 | intronic | 20p12.1 | . | bnd_270 | transl_intra_3048_0 |
| 25T | 20 | 13592149 | 13592149 | TASP1 | intronic | 20p12.1 | . | bnd_108 | del_inssd_169_0/884_0 |
| 25T | 20 | 13981153 | 13981153 | MACROD2 | intronic | 20p12.1 | Score=732;Name="2676070:Charlie7(DNA)" | bnd_85 | del_insou_682_0/322_0 |
| 25T | 20 | 14017359 | 14017359 | MACROD2 | intronic | 20p12.1 | Score=1196;Name="2676131:AluSx3(SINE)" | bnd_60 | del_insod_100_0/2438_0 |
| 25T | 20 | 14021879 | 14021879 | MACROD2 | intronic | 20p12.1 | . | bnd_62 | del_insod_100_0/2438_0 |
| 25T | 20 | 14103596 | 14103596 | MACROD2 | intronic | 20p12.1 | . | bnd_151 | del_inssu_2240_0/1450_0 |
| 25T | 20 | 14325201 | 14325201 | MACROD2 | intronic | 20p12.1 | . | bnd_220 | del_invers_1235_0/1320_0 |
| 25T | 20 | 14499918 | 14499918 | MACROD2 | intronic | 20p12.1 | . | bnd_109 | del_inssd_169_0/884_0 |
| 25T | 20 | 14831335 | 14831335 | MACROD2 | intronic | 20p12.1 | . | bnd_83 | del_insou_682_0/322_0 |
| 25T | 20 | 15051355 | 15051355 | MACROD2 | intronic | 20p12.1 | Score=2051;Name="2677839:MSTB(LTR)" | bnd_362 | transl_intra_118 |
| 25T | 20 | 15265000 | 15265000 | MACROD2 | intronic | 20p12.1 | Score=232;Name="2678214:L1MC5(LINE)" | bnd_101 | del_inssd_71_0/2634_0 |
| 25T | 20 | 15859689 | 15859689 | MACROD2 | intronic | 20p12.1 | . | bnd_364 | transl_intra_604 |
| 25T | 20 | 15865728 | 15865728 | MACROD2 | intronic | 20p12.1 | Score=2064;Name="2679212:MLT1A0(LTR)" | bnd_10 | del_715_0 |
| 25T | 20 | 16217450 | 16217450 | . | intergenic | 20p12.1 | . | bnd_442 | tandem_dup_684_0 |
| 25T | 20 | 16271889 | 16271889 | KIF16B | intronic | 20p12.1 | . | bnd_153 | del_inssu_857_0/586_0 |
| 25T | 20 | 16300105 | 16300105 | KIF16B | intronic | 20p12.1 | Score=1928;Name="2679938:L1MCb(LINE)" | bnd_272 | transl_intra_1035_0 |
| 25T | 20 | 16304293 | 16304293 | KIF16B | intronic | 20p12.1 | . | bnd_444 | tandem_dup_1019_0 |
| 25T | 20 | 16310024 | 16310024 | KIF16B | intronic | 20p12.1 | . | bnd_114 | del_inssd_2957_0/281_0 |
| 25T | 20 | 16310653 | 16310653 | KIF16B | intronic | 20p12.1 | . | bnd_366 | transl_intra_394_0 |
| 25T | 20 | 16601855 | 16601855 | . | intergenic | 20p12.1 | . | bnd_155 | del_inssu_857_0/586_0 |
| 25T | 20 | 16616055 | 16616055 | . | intergenic | 20p12.1 | Score=1980;Name="2680489:MLT1C(LTR)" | bnd_118 | del_inssd_3467_0/956_0 |
| 25T | 20 | 16616512 | 16616512 | . | intergenic | 20p12.1 | Score=1980;Name="2680491:MLT1C(LTR)" | bnd_265 | transl_intra_2017_0 |
| 25T | 20 | 16696417 | 16696417 | . | intergenic | 20p12.1 | Score=2277;Name="2680670:L1ME3A(LINE)" | bnd_217 | del_invers_196_0/146_0 |
| 25T | 20 | 16724410 | 16724410 | . | intergenic | 20p12.1 | . | bnd_215 | del_invers_196_0/146_0 |
| 25T | 20 | 16762488 | 16762488 | . | intergenic | 20p12.1 | Score=2160;Name="2680780:L1PA15(LINE)" | bnd_163 | del_inssu_1103_0/37_0 |
| 25T | 20 | 16815053 | 16815053 | . | intergenic | 20p12.1 | Score=729;Name="2680855:L1MC1(LINE)" | bnd_224 | del_invers_901_0/1085_0 |
| 25T | 20 | 17057538 | 17057538 | . | intergenic | 20p12.1 | . | bnd_112 | del_inssd_2957_0/281_0 |
| 25T | 20 | 17072711 | 17072711 | . | intergenic | 20p12.1 | . | bnd_274 | transl_intra_1760_0 |
| 25T | 20 | 17180924 | 17180924 | . | intergenic | 20p12.1 | . | bnd_368 | transl_intra_56 |
| 25T | 20 | 17401219 | 17401219 | PCSK2 | intronic | 20p12.1 | . | bnd_435 | tandem_dup_859_0 |
| 25T | 20 | 17420015 | 17420015 | PCSK2 | intronic | 20p12.1 | Score=1833;Name="2681888:AluJb(SINE)" | bnd_337 | transl_intra_140_0 |

| | | | | | | | | | |
|---|---|---|---|---|---|---|---|---|---|
| 25T | 20 | 17545445 | 17545445 | BFSP1 | intronic | 20p12.1 | Score=2317;Name="2682168:AluSx1(SINE)" | bnd_107 | del_inssd_169_0/884_0 |
| 25T | 20 | 17926828 | 17926828 | SNX5 | intronic | 20p11.23 | . | bnd_269 | transl_intra_3048_0 |
| 25T | 20 | 18007243 | 18007243 | OVOL2 | intronic | 20p11.23 | Score=2526;Name="2683160:AluY(SINE)" | bnd_365 | transl_intra_394_0 |
| 25T | 20 | 18148552 | 18148552 | CSRP2BP | intronic | 20p11.23 | . | bnd_165 | del_inssu_1103_0/37_0 |
| 25T | 20 | 18327791 | 18327791 | . | intergenic | 20p11.23 | . | bnd_370 | transl_intra_2057 |
| 25T | 20 | 18743222 | 18743222 | DTD1 | intronic | 20p11.23 | . | bnd_276 | transl_intra_3353_0 |
| 25T | 20 | 18744171 | 18744171 | DTD1 | UTR3 | 20p11.23 | . | bnd_446 | tandem_dup_1130_0 |
| 25T | 20 | 18744565 | 18744565 | . | downstream | 20p11.23 | . | bnd_345 | transl_intra_1971 |
| 25T | 20 | 18754629 | 18754629 | . | intergenic | 20p11.23 | Score=1593;Name="2684678:AluJb(SINE)" | bnd_437 | tandem_dup_582_0 |
| 25T | 20 | 19483762 | 19483762 | SLC24A3 | intronic | 20p11.23 | . | bnd_372 | transl_intra_695 |
| 25T | 20 | 19485980 | 19485980 | SLC24A3 | intronic | 20p11.23 | . | bnd_431 | tandem_dup_738_0 |
| 25T | 20 | 20502588 | 20502588 | RALGAPA2 | intronic | 20p11.23 | . | bnd_433 | tandem_dup_551_0 |
| 25T | 20 | 21181148 | 21181148 | PLK1S1,RP4-777D9.2 | ncRNA_intronic | 20p11.23 | Score=3408;Name="2688842:MER1A(DNA)" | bnd_448 | tandem_dup_407_0 |
| 25T | 20 | 21475710 | 21475710 | . | intergenic | 20p11.22 | . | bnd_146 | del_inssu_3454_0/69_0 |
| 25T | 20 | 21940782 | 21940782 | . | intergenic | 20p11.22 | Score=8996;Name="2690077:L1M4c(LINE)" | bnd_450 | tandem_dup_1400_0 |
| 25T | 20 | 21988789 | 21988789 | . | intergenic | 20p11.22 | . | bnd_208 | transl_intra_1226_0 |
| 25T | 20 | 21989040 | 21989040 | . | intergenic | 20p11.22 | Score=754;Name="2690148:MIR(SINE)" | bnd_207 | transl_intra_1226_0 |
| 25T | 20 | 21989044 | 21989044 | . | intergenic | 20p11.22 | Score=754;Name="2690148:MIR(SINE)" | bnd_150 | del_inssu_2240_0/1450_0 |
| 25T | 20 | 22082793 | 22082793 | . | intergenic | 20p11.22 | Score=207;Name="2690285:L2c(LINE)" | bnd_267 | transl_intra_298_0 |
| 25T | 20 | 22181378 | 22181378 | . | intergenic | 20p11.22 | Score=13527;Name="2690406:L1PA12(LINE)" | bnd_452 | tandem_dup_391_0 |
| 25T | 20 | 22201278 | 22201278 | . | intergenic | 20p11.22 | . | bnd_78 | del_insou_2754_0/144_0 |
| 25T | 20 | 22220643 | 22220643 | . | intergenic | 20p11.22 | . | bnd_116 | del_inssd_3467_0/956_0 |
| 25T | 20 | 22234075 | 22234075 | . | intergenic | 20p11.22 | Score=459;Name="2690481:L2c(LINE)" | bnd_259 | transl_intra_1549_0 |
| 25T | 20 | 22240193 | 22240193 | . | intergenic | 20p11.22 | . | bnd_454 | tandem_dup_2628_0 |
| 25T | 20 | 22257471 | 22257471 | . | intergenic | 20p11.22 | . | bnd_278 | transl_intra_165_0 |
| 25T | 20 | 22353474 | 22353474 | . | intergenic | 20p11.21 | Score=789;Name="2690673:FLAM_A(SINE)" | bnd_80 | del_insou_2754_0/144_0 |
| 25T | 20 | 22512327 | 22512327 | . | intergenic | 20p11.21 | . | bnd_374 | transl_intra_3323 |
| 25T | 20 | 22524254 | 22524254 | . | intergenic | 20p11.21 | Score=345;Name="2690945:MLT1J2(LTR)" | bnd_152 | del_inssu_2240_0/1450_0 |
| 25T | 20 | 23476628 | 23476628 | CST8 | UTR3 | 20p11.21 | . | bnd_280 | transl_intra_1609_0 |
| 25T | 20 | 23515660 | 23515660 | CST13P | ncRNA_intronic | 20p11.21 | Score=3623;Name="2692419:MER52A(LTR)" | bnd_154 | del_inssu_857_0/586_0 |
| 25T | 20 | 23525640 | 23525640 | . | intergenic | 20p11.21 | Score=1000;Name="2692439:MSTD(LTR)" | bnd_156 | del_inssu_857_0/586_0 |
| 25T | 20 | 23543534 | 23543534 | . | intergenic | 20p11.21 | Score=4360;Name="2692457:L1M3e(LINE)" | bnd_445 | tandem_dup_1130_0 |
| 25T | 20 | 24184761 | 24184761 | FLJ33581 | ncRNA_intronic | 20p11.21 | . | bnd_122 | del_inssd_3379_0/4895_0 |
| 25T | 20 | 24209119 | 24209119 | . | intergenic | 20p11.21 | Score=2150;Name="2693383:AluSg(SINE)" | bnd_120 | del_inssd_3379_0/4895_0 |
| 25T | 20 | 24209269 | 24209269 | . | intergenic | 20p11.21 | Score=2150;Name="2693383:AluSg(SINE)" | bnd_376 | transl_intra_1357_0 |
| 25T | 20 | 24262534 | 24262534 | . | intergenic | 20p11.21 | Score=8741;Name="2693479:MER66-int(LTR)" | bnd_449 | tandem_dup_1400_0 |
| 25T | 20 | 24271613 | 24271613 | . | intergenic | 20p11.21 | . | bnd_378 | transl_intra_419 |

| | | | | | | | | | |
|---|---|---|---|---|---|---|---|---|---|
| 25T | 20 | 24286676 | 24286676 | . | intergenic | 20p11.21 | Score=4296;Name="2693507:L1MC3(LINE)" | bnd_282 | transl_intra_347_0 |
| 25T | 20 | 24611243 | 24611243 | SYNDIG1 | intronic | 20p11.21 | . | bnd_213 | del_invers_49_0/502_0 |
| 25T | 20 | 24805489 | 24805489 | . | intergenic | 20p11.21 | Score=1106;Name="2694234:MLT1E2(LTR)" | bnd_38 | del_ins_2062 |
| 25T | 20 | 24809593 | 24809593 | . | intergenic | 20p11.21 | Score=1137;Name="2694246:MLT1E2(LTR)" | bnd_148 | del_inssu_3454_0/69_0 |
| 25T | 20 | 24810373 | 24810373 | . | intergenic | 20p11.21 | Score=1822;Name="2694248:MLT1D(LTR)" | bnd_211 | del_invers_49_0/502_0 |
| 25T | 20 | 24810884 | 24810884 | . | intergenic | 20p11.21 | . | bnd_359 | transl_intra_1647 |
| 25T | 20 | 24821203 | 24821203 | . | intergenic | 20p11.21 | . | bnd_279 | transl_intra_1609_0 |
| 25T | 20 | 25264827 | 25264827 | PYGB | exonic | 20p11.21 | . | bnd_12 | del_2955_0 |
| 25T | 20 | 25269430 | 25269430 | PYGB | intronic | 20p11.21 | . | bnd_380 | transl_intra_998_0 |
| 25T | 20 | 25399216 | 25399216 | GINS1 | intronic | 20p11.21 | Score=379;Name="2695140:MIRb(SINE)" | bnd_158 | del_ins_2600_0 |
| 25T | 20 | 30306705 | 30306705 | BCL2L1 | intronic | 20q11.21 | . | bnd_379 | transl_intra_998_0 |
| 25T | 20 | 30605743 | 30605743 | CCM2L | intronic | 20q11.21 | . | bnd_14 | del_1176 |
| 25T | 20 | 30812537 | 30812537 | POFUT1 | intronic | 20q11.21 | . | bnd_382 | transl_intra_4256_0 |
| 25T | 20 | 31365577 | 31365577 | DNMT3B | intronic | 20q11.21 | . | bnd_456 | tandem_dup_1144_0 |
| 25T | 20 | 31423084 | 31423084 | MAPRE1 | intronic | 20q11.21 | . | bnd_384 | transl_intra_2430 |
| 25T | 20 | 31909382 | 31909382 | . | intergenic | 20q11.21 | Score=496;Name="2702780:Charlie8(DNA)" | bnd_284 | transl_intra_788_0 |
| 25T | 20 | 31972694 | 31972694 | CDK5RAP1 | intronic | 20q11.21 | Score=1930;Name="2702953:AluJo(SINE)" | bnd_64 | transl_intra_1717_0 |
| 25T | 20 | 32070767 | 32070767 | . | intergenic | 20q11.21 | . | bnd_157 | del_ins_2600_0 |
| 25T | 20 | 32071052 | 32071052 | . | intergenic | 20q11.21 | . | bnd_286 | transl_intra_4414_0 |
| 25T | 20 | 32072475 | 32072475 | . | intergenic | 20q11.21 | . | bnd_381 | transl_intra_4256_0 |
| 25T | 20 | 32166616 | 32166616 | CBFA2T2 | intronic | 20q11.22 | . | bnd_225 | del_invers_901_0/1085_0 |
| 25T | 20 | 32952312 | 32952312 | ITCH | intronic | 20q11.22 | . | bnd_223 | del_invers_901_0/1085_0 |
| 25T | 20 | 33140352 | 33140352 | MAP1LC3A | intronic | 20q11.22 | Score=931;Name="2706102:L2a(LINE)" | bnd_275 | transl_intra_3353_0 |
| 25T | 20 | 33180435 | 33180435 | PIGU | intronic | 20q11.22 | . | bnd_367 | transl_intra_56 |
| 25T | 20 | 33220929 | 33220929 | PIGU | intronic | 20q11.22 | Score=3424;Name="2706308:HAL1(LINE)" | bnd_288 | transl_intra_132_0 |
| 25T | 20 | 33959735 | 33959735 | UQCC1 | intronic | 20q11.22 | Score=1607;Name="2708267:AluJr(SINE)" | bnd_181 | del_inssu_328_0/941_0 |
| 25T | 20 | 34541129 | 34541129 | . | downstream | 20q11.23 | . | bnd_290 | transl_intra_236_0 |
| 25T | 20 | 37393864 | 37393864 | ACTR5 | intronic | 20q11.23 | . | bnd_386 | transl_intra_358 |
| 25T | 20 | 37465130 | 37465130 | PPP1R16B | intronic | 20q11.23 | Score=1676;Name="2717321:AluJb(SINE)" | bnd_167 | del_inssu_308_0/34_0 |
| 25T | 20 | 37983353 | 37983353 | . | intergenic | 20q12 | Score=13571;Name="2718454:L1MA9(LINE)" | bnd_16 | del_3322 |
| 25T | 20 | 37986456 | 37986456 | . | intergenic | 20q12 | . | bnd_15 | del_3322 |
| 25T | 20 | 37992097 | 37992097 | . | intergenic | 20q12 | Score=278;Name="2718475:MIR(SINE)" | bnd_169 | del_inssu_308_0/34_0 |
| 25T | 20 | 37994950 | 37994950 | . | intergenic | 20q12 | Score=375;Name="2718483:MIR(SINE)" | bnd_369 | transl_intra_2057 |
| 25T | 20 | 38104865 | 38104865 | . | intergenic | 20q12 | . | bnd_66 | transl_intra_3792_0 |
| 25T | 20 | 38112442 | 38112442 | . | intergenic | 20q12 | Score=15446;Name="2718737:L1PA4(LINE)" | bnd_173 | del_inssu_1503_0/102_0 |
| 25T | 20 | 38146117 | 38146117 | . | intergenic | 20q12 | Score=658;Name="2718783:L2a(LINE)" | bnd_375 | transl_intra_1357_0 |
| 25T | 20 | 38146589 | 38146589 | . | intergenic | 20q12 | Score=658;Name="2718785:L2a(LINE)" | bnd_253 | transl_intra_4316_0 |
| 25T | 20 | 38154553 | 38154553 | . | intergenic | 20q12 | Score=2573;Name="2718802:AluY(SINE)" | bnd_294 | transl_intra_601_0 |

| | | | | | | | | | |
|---|---|---|---|---|---|---|---|---|---|
| 25T | 20 | 38168443 | 38168443 | . | intergenic | 20q12 | Score=1580;Name="2718825:MSTB1(LTR)" | bnd_201 | transl_intra_311_0 |
| 25T | 20 | 38214180 | 38214180 | . | intergenic | 20q12 | Score=10268;Name="2718911:L1PA3(LINE)" | bnd_388 | transl_intra_996_0 |
| 25T | 20 | 38332735 | 38332735 | . | intergenic | 20q12 | Score=430;Name="2719161:L2c(LINE)" | bnd_160 | del_inssu_888_0/1782_0 |
| 25T | 20 | 38334278 | 38334278 | . | intergenic | 20q12 | . | bnd_355 | transl_intra_254 |
| 25T | 20 | 38365769 | 38365769 | . | intergenic | 20q12 | . | bnd_183 | del_inssu_328_0/941_0 |
| 25T | 20 | 38367109 | 38367109 | . | intergenic | 20q12 | Score=2198;Name="2719210:AluSq(SINE)" | bnd_458 | tandem_dup_142_0 |
| 25T | 20 | 38404142 | 38404142 | . | intergenic | 20q12 | . | bnd_18 | del_92 |
| 25T | 20 | 38891425 | 38891425 | . | intergenic | 20q12 | . | bnd_522 | transl_inter_5548 |
| 25T | 20 | 38978212 | 38978212 | . | intergenic | 20q12 | Score=21648;Name="2720306:L1MA2(LINE)" | bnd_40 | del_ins_2602 |
| 25T | 20 | 39161335 | 39161335 | . | intergenic | 20q12 | Score=197;Name="2720687:MIRb(SINE)" | bnd_17 | del_92 |
| 25T | 20 | 39402464 | 39402464 | . | intergenic | 20q12 | . | bnd_175 | del_inssu_1503_0/102_0 |
| 25T | 20 | 39404181 | 39404181 | . | intergenic | 20q12 | . | bnd_221 | del_invers_1235_0/1320_0 |
| 25T | 20 | 39842953 | 39842953 | ZHX3 | intronic | 20q12 | Score=684;Name="2722064:MIR(SINE)" | bnd_219 | del_invers_1235_0/1320_0 |
| 25T | 20 | 39941412 | 39941412 | . | intergenic | 20q12 | Score=750;Name="2722271:L2a(LINE)" | bnd_162 | del_inssu_888_0/1782_0 |
| 25T | 20 | 40060331 | 40060331 | CHD6 | intronic | 20q12 | Score=2176;Name="2722490:L1MD1(LINE)" | bnd_20 | del_1204_0 |
| 25T | 20 | 40370829 | 40370829 | . | intergenic | 20q12 | Score=19205;Name="2723088:L1PA16(LINE)" | bnd_390 | transl_intra_1225 |
| 25T | 20 | 40604321 | 40604321 | . | intergenic | 20q12 | . | bnd_126 | del_inssd_670_0/587_0 |
| 25T | 20 | 40605891 | 40605891 | . | intergenic | 20q12 | . | bnd_82 | transl_intra_536_0 |
| 25T | 20 | 40670499 | 40670499 | . | intergenic | 20q12 | . | bnd_128 | del_1260_0 |
| 25T | 20 | 40819248 | 40819248 | PTPRT | intronic | 20q12 | Score=1026;Name="2724027:MER5A(DNA)" | bnd_127 | del_1260_0 |
| 25T | 20 | 40880081 | 40880081 | PTPRT | intronic | 20q12 | Score=1210;Name="2724147:L2a(LINE)" | bnd_228 | del_invers_1142_0/1867_0 |
| 25T | 20 | 40918160 | 40918160 | PTPRT | intronic | 20q12 | Score=3402;Name="2724216:L1PA17(LINE)" | bnd_347 | transl_intra_685 |
| 25T | 20 | 40981093 | 40981093 | PTPRT | intronic | 20q12 | . | bnd_22 | del_4282 |
| 25T | 20 | 40991882 | 40991882 | PTPRT | intronic | 20q12 | . | bnd_65 | transl_intra_3792_0 |
| 25T | 20 | 40995333 | 40995333 | PTPRT | intronic | 20q12 | . | bnd_333 | transl_intra_550 |
| 25T | 20 | 41040319 | 41040319 | PTPRT | intronic | 20q12 | Score=816;Name="2724465:L1MC(LINE)" | bnd_271 | transl_intra_1035_0 |
| 25T | 20 | 41114108 | 41114108 | PTPRT | intronic | 20q12 | . | bnd_21 | del_4282 |
| 25T | 20 | 41130313 | 41130313 | PTPRT | intronic | 20q12 | Score=932;Name="2724653:MER58A(DNA)" | bnd_39 | del_ins_2602 |
| 25T | 20 | 41140999 | 41140999 | PTPRT | intronic | 20q12 | Score=1851;Name="2724680:L1MD2(LINE)" | bnd_273 | transl_intra_1760_0 |
| 25T | 20 | 41272984 | 41272984 | PTPRT | intronic | 20q12 | . | bnd_261 | transl_intra_975_0 |
| 25T | 20 | 41494795 | 41494795 | PTPRT | intronic | 20q12 | . | bnd_35 | del_ins_1366_0 |
| 25T | 20 | 41496370 | 41496370 | PTPRT | intronic | 20q12 | Score=2354;Name="2725370:AluSx1(SINE)" | bnd_24 | del_1354 |
| 25T | 20 | 41514513 | 41514513 | PTPRT | exonic | 20q12 | . | bnd_229 | del_invers_1142_0/1867_0 |
| 25T | 20 | 41515231 | 41515231 | PTPRT | intronic | 20q12 | . | bnd_227 | del_invers_1142_0/1867_0 |
| 25T | 20 | 41515255 | 41515255 | PTPRT | intronic | 20q12 | . | bnd_230 | del_invers_1142_0/1867_0 |
| 25T | 20 | 41515484 | 41515484 | PTPRT | intronic | 20q12 | . | bnd_68 | del_insod_1121_0/50_0 |
| 25T | 20 | 42031148 | 42031148 | . | intergenic | 20q13.11 | Score=17961;Name="2726572:L1PA5(LINE)" | bnd_19 | del_1204_0 |
| 25T | 20 | 42208641 | 42208641 | SGK2 | exonic | 20q13.12 | . | bnd_296 | transl_intra_1288_0 |

| | | | | | | | | | |
|---|---|---|---|---|---|---|---|---|---|
| 25T | 20 | 42209816 | 42209816 | SGK2 | intronic | 20q13.12 | Score=762;Name="2727074:L1ME4a(LINE)" | bnd_392 | transl_intra_77 |
| 25T | 20 | 42643921 | 42643921 | TOX2 | intronic | 20q13.12 | . | bnd_443 | tandem_dup_1019_0 |
| 25T | 20 | 43237402 | 43237402 | PKIG | intronic | 20q13.12 | Score=1695;Name="2729886:AluJb(SINE)" | bnd_81 | transl_intra_536_0 |
| 25T | 20 | 43624608 | 43624608 | STK4 | intronic | 20q13.12 | Score=360;Name="2730915:Charlie8(DNA)" | bnd_298 | transl_intra_589_0 |
| 25T | 20 | 43688015 | 43688015 | STK4 | intronic | 20q13.12 | Score=3856;Name="2731052:MER44C(DNA)" | bnd_441 | tandem_dup_684_0 |
| 25T | 20 | 43922658 | 43922658 | MATN4 | splicing | 20q13.12 | . | bnd_394 | transl_intra_2274_0 |
| 25T | 20 | 43979047 | 43979047 | . | intergenic | 20q13.12 | Score=804;Name="2731656:Charlie2a(DNA)" | bnd_164 | del_inssu_1103_0/37_0 |
| 25T | 20 | 44075285 | 44075285 | . | intergenic | 20q13.12 | . | bnd_70 | del_insod_1121_0/50_0 |
| 25T | 20 | 44833113 | 44833113 | CDH22 | intronic | 20q13.12 | Score=656;Name="2733627:MIR(SINE)" | bnd_168 | del_inssu_308_0/34_0 |
| 25T | 20 | 44833549 | 44833549 | CDH22 | intronic | 20q13.12 | . | bnd_185 | del_inssu_261_0/301_0 |
| 25T | 20 | 44976588 | 44976588 | . | intergenic | 20q13.12 | . | bnd_187 | del_inssu_261_0/301_0 |
| 25T | 20 | 44978850 | 44978850 | SLC35C2 | UTR3 | 20q13.12 | . | bnd_170 | del_inssu_308_0/34_0 |
| 25T | 20 | 45153444 | 45153444 | . | intergenic | 20q13.12 | Score=4389;Name="2734344:L1MC3(LINE)" | bnd_391 | transl_intra_77 |
| 25T | 20 | 45287025 | 45287025 | SLC13A3 | intronic | 20q13.12 | Score=1178;Name="2734740:L1ME1(LINE)" | bnd_457 | tandem_dup_142_0 |
| 25T | 20 | 45437671 | 45437671 | . | intergenic | 20q13.12 | Score=821;Name="2735160:Charlie4a(DNA)" | bnd_166 | del_inssu_1103_0/37_0 |
| 25T | 20 | 45612253 | 45612253 | EYA2 | intronic | 20q13.12 | Score=2821;Name="2735619:L1MEg(LINE)" | bnd_396 | transl_intra_3766_0 |
| 25T | 20 | 45672619 | 45672619 | EYA2 | intronic | 20q13.12 | . | bnd_335 | transl_intra_3287 |
| 25T | 20 | 45716133 | 45716133 | EYA2 | intronic | 20q13.12 | Score=302;Name="2735922:MIRc(SINE)" | bnd_26 | del_1695 |
| 25T | 20 | 45733132 | 45733132 | EYA2 | intronic | 20q13.12 | Score=3676;Name="2735963:L1ME1(LINE)" | bnd_7 | del_3420 |
| 25T | 20 | 46619863 | 46619863 | . | intergenic | 20q13.13 | . | bnd_205 | del_invers_421_0/19_0 |
| 25T | 20 | 46693548 | 46693548 | . | intergenic | 20q13.13 | . | bnd_447 | tandem_dup_407_0 |
| 25T | 20 | 46772680 | 46772680 | . | intergenic | 20q13.13 | . | bnd_113 | del_inssd_2957_0/281_0 |
| 25T | 20 | 46776532 | 46776532 | . | intergenic | 20q13.13 | . | bnd_283 | transl_intra_788_0 |
| 25T | 20 | 46789123 | 46789123 | . | intergenic | 20q13.13 | . | bnd_383 | transl_intra_2430 |
| 25T | 20 | 46793466 | 46793466 | . | intergenic | 20q13.13 | Score=502;Name="2738694:L2a(LINE)" | bnd_297 | transl_intra_589_0 |
| 25T | 20 | 46794996 | 46794996 | . | intergenic | 20q13.13 | Score=698;Name="2738697:MIR(SINE)" | bnd_23 | del_1354 |
| 25T | 20 | 46797617 | 46797617 | . | intergenic | 20q13.13 | Score=332;Name="2738701:MER113(DNA)" | bnd_455 | tandem_dup_1144_0 |
| 25T | 20 | 46806613 | 46806613 | . | intergenic | 20q13.13 | Score=727;Name="2738723:MLT1K(LTR)" | bnd_351 | transl_intra_1724 |
| 25T | 20 | 46871593 | 46871593 | . | intergenic | 20q13.13 | Score=828;Name="2738864:L2(LINE)" | bnd_300 | transl_intra_83_0 |
| 25T | 20 | 46872864 | 46872864 | . | intergenic | 20q13.13 | . | bnd_61 | del_insod_100_0/2438_0 |
| 25T | 20 | 47018318 | 47018318 | . | intergenic | 20q13.13 | . | bnd_218 | del_invers_196_0/146_0 |
| 25T | 20 | 47058615 | 47058615 | . | intergenic | 20q13.13 | . | bnd_171 | tandem_dup_98_0 |
| 25T | 20 | 47058974 | 47058974 | . | intergenic | 20q13.13 | . | bnd_361 | transl_intra_118 |
| 25T | 20 | 47166104 | 47166104 | . | intergenic | 20q13.13 | Score=728;Name="2739509:L2a(LINE)" | bnd_257 | transl_intra_2235_0 |
| 25T | 20 | 47225555 | 47225555 | . | intergenic | 20q13.13 | . | bnd_349 | transl_intra_2355 |
| 25T | 20 | 47226791 | 47226791 | . | intergenic | 20q13.13 | Score=342;Name="2739642:T-rich(Low_complexity)" | bnd_302 | transl_intra_2406_0 |
| 25T | 20 | 47380825 | 47380825 | PREX1 | intronic | 20q13.13 | . | bnd_299 | transl_intra_83_0 |

| | | | | | | | | | |
|---|---|---|---|---|---|---|---|---|---|
| 25T | 20 | 47579018 | 47579018 | ARFGEF2 | intronic | 20q13.13 | Score=622;Name="2740627:MER113A(DNA)" | bnd_341 | transl_intra_103 |
| 25T | 20 | 47624217 | 47624217 | ARFGEF2 | intronic | 20q13.13 | . | bnd_203 | del_invers_421_0/19_0 |
| 25T | 20 | 47887598 | 47887598 | ZNFX1 | exonic | 20q13.13 | . | bnd_287 | transl_intra_132_0 |
| 25T | 20 | 47889418 | 47889418 | ZNFX1 | intronic | 20q13.13 | . | bnd_357 | transl_intra_207 |
| 25T | 20 | 47942016 | 47942016 | . | intergenic | 20q13.13 | Score=298;Name="2741549:LTR33(LTR)" | bnd_177 | del_inssu_1755_0/414_0 |
| 25T | 20 | 48288758 | 48288758 | B4GALT5 | intronic | 20q13.13 | . | bnd_84 | del_insou_682_0/322_0 |
| 25T | 20 | 48293998 | 48293998 | B4GALT5 | intronic | 20q13.13 | . | bnd_86 | del_insou_682_0/322_0 |
| 25T | 20 | 48298775 | 48298775 | B4GALT5 | intronic | 20q13.13 | Score=408;Name="2742594:MER58A(DNA)" | bnd_304 | transl_intra_919_0 |
| 25T | 20 | 48300158 | 48300158 | B4GALT5 | intronic | 20q13.13 | Score=2214;Name="2742598:AluSx1(SINE)" | bnd_37 | del_ins_2062 |
| 25T | 20 | 48719162 | 48719162 | UBE2V1,TMEM189-UBE2V1 | intronic | 20q13.13 | Score=1191;Name="2743776:AluSx(SINE)" | bnd_289 | transl_intra_236_0 |
| 25T | 20 | 50658584 | 50658584 | . | intergenic | 20q13.2 | Score=1837;Name="2748942:AluJr(SINE)" | bnd_59 | del_insod_100_0/2438_0 |
| 25T | 20 | 50769289 | 50769289 | ZFP64 | exonic | 20q13.2 | . | bnd_460 | tandem_dup_1415_0 |
| 25T | 20 | 51138867 | 51138867 | . | intergenic | 20q13.2 | Score=952;Name="2750181:L1MD1(LINE)" | bnd_249 | transl_intra_1002_0 |
| 25T | 20 | 51316216 | 51316216 | . | intergenic | 20q13.2 | Score=261;Name="2750593:L4(LINE)" | bnd_11 | del_2955_0 |
| 25T | 20 | 51337051 | 51337051 | . | intergenic | 20q13.2 | Score=399;Name="2750649:L2a(LINE)" | bnd_462 | tandem_dup_594_0 |
| 25T | 20 | 51360425 | 51360425 | . | intergenic | 20q13.2 | Score=565;Name="2750690:L2b(LINE)" | bnd_174 | del_inssu_1503_0/102_0 |
| 25T | 20 | 51652666 | 51652666 | TSHZ2 | intronic | 20q13.2 | . | bnd_464 | tandem_dup_106_0 |
| 25T | 20 | 51674369 | 51674369 | TSHZ2 | intronic | 20q13.2 | Score=272;Name="2751363:L2b(LINE)" | bnd_111 | del_inssd_2957_0/281_0 |
| 25T | 20 | 51674481 | 51674481 | TSHZ2 | intronic | 20q13.2 | Score=1917;Name="2751364:MER1B(DNA)" | bnd_255 | transl_intra_237_0 |
| 25T | 20 | 51680077 | 51680077 | TSHZ2 | intronic | 20q13.2 | . | bnd_9 | del_715_0 |
| 25T | 20 | 51927345 | 51927345 | TSHZ2 | intronic | 20q13.2 | Score=1566;Name="2751868:MLT1F2(LTR)" | bnd_306 | transl_intra_735_0 |
| 25T | 20 | 52194784 | 52194784 | ZNF217 | intronic | 20q13.2 | . | bnd_398 | transl_intra_163_0 |
| 25T | 20 | 52407318 | 52407318 | . | intergenic | 20q13.2 | . | bnd_179 | del_inssu_1755_0/414_0 |
| 25T | 20 | 52408324 | 52408324 | . | intergenic | 20q13.2 | . | bnd_373 | transl_intra_3323 |
| 25T | 20 | 52592230 | 52592230 | BCAS1 | intronic | 20q13.2 | Score=214;Name="2753639:L4(LINE)" | bnd_285 | transl_intra_4414_0 |
| 25T | 20 | 52594135 | 52594135 | BCAS1 | intronic | 20q13.2 | . | bnd_222 | del_invers_1235_0/1320_0 |
| 25T | 20 | 52598716 | 52598716 | BCAS1 | intronic | 20q13.2 | . | bnd_134 | del_inssd_176_0/437_0 |
| 25T | 20 | 52620728 | 52620728 | BCAS1 | intronic | 20q13.2 | Score=1726;Name="2753696:MLT1A0(LTR)" | bnd_124 | del_inssd_670_0/587_0 |
| 25T | 20 | 52741377 | 52741377 | . | intergenic | 20q13.2 | . | bnd_397 | transl_intra_163_0 |
| 25T | 20 | 52839960 | 52839960 | . | intergenic | 20q13.2 | . | bnd_459 | tandem_dup_1415_0 |
| 25T | 20 | 52881213 | 52881213 | . | intergenic | 20q13.2 | Score=842;Name="2754304:L2a(LINE)" | bnd_461 | tandem_dup_594_0 |
| 25T | 20 | 53170023 | 53170023 | DOK5 | intronic | 20q13.2 | Score=2633;Name="2754947:AluY(SINE)" | bnd_393 | transl_intra_2274_0 |
| 25T | 20 | 53528279 | 53528279 | . | intergenic | 20q13.2 | Score=1446;Name="2755628:L1ME3(LINE)" | bnd_93 | del_insou_472_0/2252_0 |
| 25T | 20 | 53535761 | 53535761 | . | intergenic | 20q13.2 | Score=2589;Name="2755644:AluY(SINE)" | bnd_451 | tandem_dup_391_0 |
| 25T | 20 | 53538634 | 53538634 | . | intergenic | 20q13.2 | Score=2829;Name="2755650:L1MC3(LINE)" | bnd_206 | del_invers_421_0/19_0 |

| | | | | | | | | | |
|---|---|---|---|---|---|---|---|---|---|
| 25T | 20 | 53685385 | 53685385 | . | intergenic | 20q13.2 | Score=305;Name="2755936:Tigger12c(DNA)" | bnd_42 | del_ins_80 |
| 25T | 20 | 53729391 | 53729391 | . | intergenic | 20q13.2 | . | bnd_41 | del_ins_80 |
| 25T | 20 | 53792159 | 53792159 | . | intergenic | 20q13.2 | Score=306;Name="2756152:(TG)n(Simple_repeat)" | bnd_463 | tandem_dup_106_0 |
| 25T | 20 | 53794518 | 53794518 | . | intergenic | 20q13.2 | . | bnd_214 | del_invers_49_0/502_0 |
| 25T | 20 | 53861667 | 53861667 | . | intergenic | 20q13.2 | Score=22428;Name="2756278:L1PA8(LINE)" | bnd_178 | del_inssu_1755_0/414_0 |
| 25T | 20 | 54303294 | 54303294 | . | intergenic | 20q13.2 | Score=580;Name="2757089:LTR37A(LTR)" | bnd_363 | transl_intra_604 |
| 25T | 20 | 54354105 | 54354105 | . | intergenic | 20q13.2 | . | bnd_371 | transl_intra_695 |
| 25T | 20 | 54367100 | 54367100 | . | intergenic | 20q13.2 | Score=2377;Name="2757221:AluSx(SINE)" | bnd_91 | del_insou_472_0/2252_0 |
| 25T | 20 | 54465140 | 54465140 | . | intergenic | 20q13.2 | Score=590;Name="2757397:MIRb(SINE)" | bnd_125 | del_inssd_670_0/587_0 |
| 25T | 20 | 54870609 | 54870609 | . | intergenic | 20q13.2 | . | bnd_377 | transl_intra_419 |
| 25T | 20 | 54881132 | 54881132 | . | intergenic | 20q13.2 | Score=299;Name="2758266:L2c(LINE)" | bnd_293 | transl_intra_601_0 |
| 25T | 20 | 55480124 | 55480124 | . | intergenic | 20q13.31 | . | bnd_453 | tandem_dup_2628_0 |
| 25T | 20 | 55524486 | 55524486 | . | intergenic | 20q13.31 | . | bnd_295 | transl_intra_1288_0 |
| 25T | 20 | 55526032 | 55526032 | . | intergenic | 20q13.31 | Score=307;Name="2759958:G-rich(Low_complexity)" | bnd_176 | del_inssu_1503_0/102_0 |
| 25T | 20 | 55561325 | 55561325 | . | intergenic | 20q13.31 | Score=357;Name="2760036:LTR67B(LTR)" | bnd_182 | del_inssu_328_0/941_0 |
| 25T | 20 | 56946119 | 56946119 | . | intergenic | 20q13.32 | . | bnd_13 | del_1176 |
| 25T | 20 | 57145843 | 57145843 | APCDD1L-AS1 | ncRNA_intronic | 20q13.32 | . | bnd_28 | del_976 |
| 25T | 20 | 57577122 | 57577122 | CTSZ | intronic | 20q13.32 | . | bnd_63 | transl_intra_1717_0 |
| 25T | 20 | 57709204 | 57709204 | . | intergenic | 20q13.32 | . | bnd_385 | transl_intra_358 |
| 25T | 20 | 57922162 | 57922162 | . | intergenic | 20q13.32 | Score=280;Name="2764543:MamRep38(DNA)" | bnd_186 | del_inssu_261_0/301_0 |
| 25T | 20 | 57922744 | 57922744 | . | intergenic | 20q13.32 | Score=4714;Name="2764545:L1MC3(LINE)" | bnd_27 | del_976 |
| 25T | 20 | 58164372 | 58164372 | PHACTR3 | intronic | 20q13.32 | . | bnd_123 | del_inssd_670_0/587_0 |
| 25T | 20 | 58181619 | 58181619 | PHACTR3 | intronic | 20q13.32 | . | bnd_226 | del_invers_901_0/1085_0 |
| 25T | 20 | 58182186 | 58182186 | PHACTR3 | intronic | 20q13.32 | . | bnd_184 | del_inssu_328_0/941_0 |
| 25T | 20 | 58191969 | 58191969 | PHACTR3 | intronic | 20q13.32 | . | bnd_305 | transl_intra_735_0 |
| 25T | 20 | 58195500 | 58195500 | PHACTR3 | intronic | 20q13.32 | Score=2452;Name="2765050:L1MC4a(LINE)" | bnd_188 | del_inssu_261_0/301_0 |
| 25T | 20 | 58325136 | 58325136 | PHACTR3 | intronic | 20q13.32 | Score=331;Name="2765303:L2b(LINE)" | bnd_281 | transl_intra_347_0 |
| 25T | 20 | 58396462 | 58396462 | PHACTR3 | intronic | 20q13.32 | Score=374;Name="2765424:MLT1J(LTR)" | bnd_130 | tandem_dup_173_0 |
| 25T | 20 | 58749562 | 58749562 | RP5-1043L13.1 | ncRNA_intronic | 20q13.33 | Score=735;Name="2766025:MER33(DNA)" | bnd_303 | transl_intra_919_0 |
| 25T | 20 | 58751794 | 58751794 | RP5-1043L13.1 | ncRNA_intronic | 20q13.33 | Score=192;Name="2766029:(TG)n(Simple_repeat)" | bnd_387 | transl_intra_996_0 |
| 25T | 20 | 58756097 | 58756097 | RP5-1043L13.1 | ncRNA_intronic | 20q13.33 | . | bnd_88 | del_insou_708_0/145_0 |
| 25T | 20 | 58989560 | 58989560 | . | intergenic | 20q13.33 | . | bnd_90 | del_insou_708_0/145_0 |
| 25T | 20 | 59101821 | 59101821 | . | intergenic | 20q13.33 | . | bnd_129 | tandem_dup_173_0 |
| 25T | 20 | 59189478 | 59189478 | . | intergenic | 20q13.33 | . | bnd_132 | del_inssd_176_0/437_0 |
| 25T | 20 | 59199322 | 59199322 | . | intergenic | 20q13.33 | . | bnd_277 | transl_intra_165_0 |
| 25T | 20 | 59324742 | 59324742 | . | intergenic | 20q13.33 | . | bnd_133 | del_inssd_176_0/437_0 |
| 25T | 20 | 59366119 | 59366119 | . | intergenic | 20q13.33 | . | bnd_180 | del_inssu_1755_0/414_0 |
| 25T | 20 | 59620631 | 59620631 | . | intergenic | 20q13.33 | Score=232;Name="2767496:L2a(LINE)" | bnd_92 | del_insou_472_0/2252_0 |
| 25T | 20 | 59628702 | 59628702 | . | intergenic | 20q13.33 | . | bnd_94 | del_insou_472_0/2252_0 |

| | | | | | | | | | |
|---|---|---|---|---|---|---|---|---|---|
| 25T | 20 | 59637619 | 59637619 | . | intergenic | 20q13.33 | Score=1832;Name="2767519:L1ME1(LINE)" | bnd_131 | del_inssd_176_0/437_0 |
| 25T | 20 | 59639234 | 59639234 | . | intergenic | 20q13.33 | Score=4375;Name="2767525:L1PB2(LINE)" | bnd_389 | transl_intra_1225 |
| 25T | 20 | 59702464 | 59702464 | . | intergenic | 20q13.33 | . | bnd_263 | transl_intra_504_0 |
| 25T | 20 | 60885332 | 60885332 | LAMA5 | exonic | 20q13.33 | . | bnd_121 | del_inssd_3379_0/4895_0 |
| 25T | 20 | 61381171 | 61381171 | NTSR1 | intronic | 20q13.33 | Score=5297;Name="2769807:L1MA9(LINE)" | bnd_69 | del_insod_1121_0/50_0 |
| 25T | 20 | 61849959 | 61849959 | . | intergenic | 20q13.33 | . | bnd_395 | transl_intra_3766_0 |
| 25T | 20 | 61999378 | 61999378 | . | intergenic | 20q13.33 | . | bnd_301 | transl_intra_2406_0 |
| 25T | 20 | 62001649 | 62001649 | . | intergenic | 20q13.33 | . | bnd_25 | del_1695 |
| 25T | 20 | 62005493 | 62005493 | . | intergenic | 20q13.33 | . | bnd_67 | del_insod_1121_0/50_0 |
| 25T | 20 | 62006312 | 62006312 | . | intergenic | 20q13.33 | . | bnd_119 | del_inssd_3379_0/4895_0 |
| 25T | 20 | 62847148 | 62847148 | MYT1 | intronic | 20q13.33 | Score=1429;Name="2771844:AluJr4(SINE)" | bnd_115 | del_inssd_3467_0/956_0 |
| 25T | 20 | 62903206 | 62903206 | PCMTD2 | intronic | 20q13.33 | . | bnd_105 | tandem_dup_111_0 |
| 25T | 20 | 62680307 | 62680307 | SOX18 | exonic | 20q13.33 | . | bnd_117 | del_inssd_3467_0/956_0 |
| 25T | 20 | 37464538 | 37464538 | PPP1R16B | UTR5 | 20q11.23 | . | bnd_292 | transl_intra_2631_0 |
| 25T | 20 | 37464648 | 37464648 | PPP1R16B | exonic | 20q11.23 | . | bnd_291 | transl_intra_2631_0 |
| 25T | 21 | 39416451 | 39416451 | . | intergenic | 21q22.13 | Score=2235;Name="2818647:THE1B(LTR)" | bnd_524 | transl_inter_8326 |
| 25T | 22 | 45539892 | 45539892 | LOC100506714 | ncRNA_intronic | 22q13.31 | Score=1369;Name="2898588:L1MEg(LINE)" | bnd_521 | transl_inter_5548 |
| 25T | X | 149151510 | 149151510 | LINC00894 | ncRNA_intronic | Xq28 | . | bnd_515 | transl_inter_7994 |
| 27T | 1 | 29265574 | 29265574 | EPB41 | intronic | 1p35.3 | Score=8146;Name="64013:L1MC3(LINE)" | bnd_20 | transl_intra_2023 |
| 27T | 1 | 29271210 | 29271210 | EPB41 | intronic | 1p35.3 | Score=2181;Name="64024:AluSz(SINE)" | bnd_19 | transl_intra_2023 |
| 27T | 4 | 61803169 | 61803169 | . | intergenic | 4q13.1 | . | bnd_26 | tandem_dup_1021_0 |
| 27T | 4 | 64078546 | 64078546 | . | intergenic | 4q13.1 | . | bnd_25 | tandem_dup_1021_0 |
| 27T | 6 | 93596485 | 93596485 | . | intergenic | 6q16.1 | . | bnd_24 | transl_intra_1344_0 |
| 27T | 6 | 105503299 | 105503299 | LIN28B | intronic | 6q21 | Score=247;Name="4064539:L1MEc(LINE)" | bnd_23 | transl_intra_1344_0 |
| 27T | 8 | 35610704 | 35610704 | UNC5D | intronic | 8p12 | . | bnd_6 | del_1662 |
| 27T | 8 | 35642943 | 35642943 | UNC5D | intronic | 8p12 | Score=2166;Name="4568331:AluSx(SINE)" | bnd_5 | del_1662 |
| 27T | 8 | 40970222 | 40970222 | . | intergenic | 8p11.21 | Score=3196;Name="4578399:MER82(DNA)" | bnd_16 | transl_intra_1582_0 |
| 27T | 8 | 50756539 | 50756539 | . | intergenic | 8q11.21 | . | bnd_15 | transl_intra_1582_0 |
| 27T | 9 | 31407294 | 31407294 | . | intergenic | 9p21.1 | Score=1999;Name="4810733:Tigger7(DNA)" | bnd_18 | transl_intra_729_0 |
| 27T | 9 | 31408664 | 31408664 | . | intergenic | 9p21.1 | Score=2073;Name="4810736:L2a(LINE)" | bnd_17 | transl_intra_729_0 |
| 27T | 12 | 44235822 | 44235822 | TMEM117 | intronic | 12q12 | . | bnd_2 | del_4735 |
| 27T | 12 | 44495063 | 44495063 | TMEM117 | intronic | 12q12 | . | bnd_1 | del_4735 |
| 27T | 15 | 83807846 | 83807846 | HDGFRP3 | UTR3 | 15q25.2 | . | bnd_4 | del_1558 |
| 27T | 15 | 83808441 | 83808441 | HDGFRP3 | intronic | 15q25.2 | . | bnd_3 | del_1558 |
| 27T | 19 | 18277169 | 18277169 | PIK3R2 | intronic | 19p13.11 | Score=241;Name="2146803:L2b(LINE)" | bnd_22 | transl_intra_2 |
| 27T | 19 | 18280369 | 18280369 | PIK3R2 | UTR3 | 19p13.11 | . | bnd_21 | transl_intra_2 |
| 27T | 19 | 18423724 | 18423724 | LSM4 | intronic | 19p13.11 | . | bnd_10 | transl_intra_468_0 |
| 27T | 19 | 18649885 | 18649885 | FKBP8 | intronic | 19p13.11 | Score=2551;Name="2147684:AluY(SINE)" | bnd_9 | transl_intra_468_0 |
| 27T | 19 | 20121319 | 20121319 | ZNF682 | intronic | 19p12 | Score=2264;Name="2151054:AluSx(SINE)" | bnd_8 | transl_intra_6_0 |
| 27T | 19 | 20138282 | 20138282 | ZNF682 | intronic | 19p12 | . | bnd_7 | transl_intra_6_0 |
| 27T | 19 | 21281108 | 21281108 | ZNF714 | exonic | 19p12 | . | bnd_12 | transl_intra_214_0 |

| | | | | | | | | | |
|---|---|---|---|---|---|---|---|---|---|
| 27T | 19 | 21283200 | 21283200 | ZNF714 | intronic | 19p12 | Score=1972;Name="2153236:AluSp(SINE)" | bnd_11 | transl_intra_214_0 |
| 27T | 19 | 24575914 | 24575914 | . | intergenic | 19p11 | Score=1186;Name="2159169:ALR/Alpha(Satellite)" | bnd_14 | transl_intra_97 |
| 27T | 19 | 24577135 | 24577135 | . | intergenic | 19p11 | Score=21474;Name="2159170:L1PA2(LINE)" | bnd_13 | transl_intra_97 |
| 28T | 1 | 31251198 | 31251198 | . | intergenic | 1p35.2 | Score=520;Name="67999:MIR(SINE)" | bnd_20 | tandem_dup_959 |
| 28T | 1 | 31412696 | 31412696 | PUM1 | intronic | 1p35.2 | Score=932;Name="68441:AluJr(SINE)" | bnd_19 | tandem_dup_959 |
| 28T | 1 | 33122163 | 33122163 | RBBP4 | intronic | 1p35.1 | Score=2284;Name="73105:AluSz(SINE)" | bnd_22 | tandem_dup_2179 |
| 28T | 1 | 33177835 | 33177835 | . | intergenic | 1p35.1 | . | bnd_21 | tandem_dup_2179 |
| 28T | 1 | 182240709 | 182240709 | GS1-122H1.2 | ncRNA_intronic | 1q25.3 | Score=5384;Name="312660:L1MC3(LINE)" | bnd_2 | del_889 |
| 28T | 1 | 182249728 | 182249728 | GS1-122H1.2 | ncRNA_intronic | 1q25.3 | . | bnd_1 | del_889 |
| 28T | 1 | 182363367 | 182363367 | . | intergenic | 1q25.3 | . | bnd_4 | del_1491 |
| 28T | 1 | 182410556 | 182410556 | . | intergenic | 1q25.3 | . | bnd_3 | del_1491 |
| 28T | 1 | 244311294 | 244311294 | . | intergenic | 1q44 | . | bnd_6 | del_896 |
| 28T | 1 | 244316579 | 244316579 | . | intergenic | 1q44 | . | bnd_5 | del_896 |
| 28T | 2 | 72434043 | 72434043 | EXOC6B | intronic | 2p13.2 | Score=374;Name="2363766:L1ME4a(LINE)" | bnd_14 | del_1507 |
| 28T | 2 | 72860220 | 72860220 | EXOC6B | intronic | 2p13.2 | . | bnd_13 | del_1507 |
| 28T | 3 | 15747241 | 15747241 | ANKRD28 | intronic | 3p25.1 | Score=2145;Name="2939031:AluSx1(SINE)" | bnd_16 | del_2816 |
| 28T | 3 | 15928812 | 15928812 | . | intergenic | 3p25.1 | Score=4607;Name="2939368:MLT1A0-int(LTR)" | bnd_15 | del_2816 |
| 28T | 4 | 151171254 | 151171254 | DCLK2 | intronic | 4q31.3 | . | bnd_33 | transl_inter_1117 |
| 28T | 6 | 33180266 | 33180266 | RING1 | UTR3 | 6p21.32 | . | bnd_37 | transl_inter_5979 |
| 28T | 7 | 117868679 | 117868679 | ANKRD7 | intronic | 7q31.31 | Score=1974;Name="4433600:L2a(LINE)" | bnd_30 | tandem_dup_1126 |
| 28T | 7 | 117892011 | 117892011 | . | intergenic | 7q31.31 | . | bnd_29 | tandem_dup_1126 |
| 28T | 9 | 21967646 | 21967646 | C9orf53 | ncRNA_exonic | 9p21.3 | Score=282;Name="4795835:MER104(DNA)" | bnd_18 | del_4035 |
| 28T | 9 | 22157675 | 22157675 | . | intergenic | 9p21.3 | . | bnd_17 | del_4035 |
| 28T | 9 | 110010580 | 110010580 | . | intergenic | 9q31.2 | . | bnd_32 | tandem_dup_3189 |
| 28T | 9 | 110085770 | 110085770 | RAD23B | intronic | 9q31.2 | . | bnd_31 | tandem_dup_3189 |
| 28T | 10 | 26220776 | 26220776 | . | intergenic | 10p12.1 | Score=2475;Name="479666:AluSc(SINE)" | bnd_34 | transl_inter_1117 |
| 28T | 11 | 35895736 | 35895736 | . | intergenic | 11p13 | Score=2433;Name="734182:AluSq(SINE)" | bnd_8 | del_1287 |
| 28T | 11 | 36036039 | 36036039 | LDLRAD3 | intronic | 11p13 | . | bnd_7 | del_1287 |
| 28T | 12 | 106948753 | 106948753 | LOC100287944 | ncRNA_intronic | 12q23.3 | Score=2358;Name="1097525:MSTA(LTR)" | bnd_36 | transl_inter_2690 |
| 28T | 15 | 89724795 | 89724795 | ABHD2 | intronic | 15q26.1 | Score=226;Name="1611702:L3(LINE)" | bnd_24 | tandem_dup_1191 |
| 28T | 15 | 89860757 | 89860757 | POLG | exonic | 15q26.1 | . | bnd_23 | tandem_dup_1191 |
| 28T | 17 | 74687086 | 74687086 | MXRA7 | intronic | 17q25.1 | Score=2986;Name="1956366:MER21C(LTR)" | bnd_26 | tandem_dup_1174 |
| 28T | 17 | 74742511 | 74742511 | MFSD11 | intronic | 17q25.1 | Score=6524;Name="1956460:Tigger1(DNA)" | bnd_25 | tandem_dup_1174 |
| 28T | 17 | 76443247 | 76443247 | DNAH17 | intronic | 17q25.3 | Score=1614;Name="1960137:AluJr(SINE)" | bnd_10 | del_499 |
| 28T | 17 | 76444175 | 76444175 | DNAH17 | intronic | 17q25.3 | Score=2346;Name="1960139:AluSp(SINE)" | bnd_9 | del_499 |

| | | | | | | | | | |
|---|---|---|---|---|---|---|---|---|---|
| 28T | 18 | 19461486 | 19461486 | . | intergenic | 18q11.2 | Score=2193;Name="2001099:L1MC2(LINE)" | bnd_28 | tandem_dup_2505 |
| 28T | 18 | 22627010 | 22627010 | . | intergenic | 18q11.2 | . | bnd_27 | tandem_dup_2505 |
| 28T | 19 | 47477117 | 47477117 | ARHGAP35 | intronic | 19q13.32 | Score=2080;Name="2203292:AluSg4(SINE)" | bnd_35 | transl_inter_2690 |
| 28T | 19 | 57001597 | 57001597 | ZNF667-AS1 | ncRNA_intronic | 19q13.43 | Score=317;Name="2227718:L1MC4a(LINE)" | bnd_12 | del_2491 |
| 28T | 19 | 57102620 | 57102620 | . | intergenic | 19q13.43 | Score=4276;Name="2227913:L1MC(LINE)" | bnd_11 | del_2491 |
| 28T | 21 | 29949295 | 29949295 | . | intergenic | 21q21.3 | Score=2271;Name="2800704:AluSq2(SINE)" | bnd_38 | transl_inter_5979 |
| 30T | 2 | 4194003 | 4194003 | . | intergenic | 2p25.3 | Score=6111;Name="2239672:L1ME1(LINE)" | bnd_16 | del_2375 |
| 30T | 2 | 4201862 | 4201862 | . | intergenic | 2p25.3 | . | bnd_31 | del_inssu_1565_0/4203_0 |
| 30T | 2 | 4230312 | 4230312 | . | intergenic | 2p25.3 | . | bnd_33 | del_inssu_1565_0/4203_0 |
| 30T | 2 | 5415328 | 5415328 | . | intergenic | 2p25.2 | Score=3312;Name="2241532:L1PB3(LINE)" | bnd_15 | del_2375 |
| 30T | 2 | 5505144 | 5505144 | . | intergenic | 2p25.2 | . | bnd_18 | del_1862 |
| 30T | 2 | 5509513 | 5509513 | . | intergenic | 2p25.2 | Score=201;Name="2241696:GA-rich(Low_complexity)" | bnd_17 | del_1862 |
| 30T | 2 | 5510261 | 5510261 | . | intergenic | 2p25.2 | . | bnd_32 | del_inssu_1565_0/4203_0 |
| 30T | 2 | 5514657 | 5514657 | . | intergenic | 2p25.2 | . | bnd_34 | del_inssu_1565_0/4203_0 |
| 30T | 2 | 191912675 | 191912675 | STAT4 | intronic | 2q32.3 | Score=2324;Name="2558117:AluSz(SINE)" | bnd_56 | tandem_dup_4144 |
| 30T | 2 | 192518861 | 192518861 | . | intergenic | 2q32.3 | Score=3188;Name="2559189:HAL1(LINE)" | bnd_55 | tandem_dup_4144 |
| 30T | 2 | 201417732 | 201417732 | SGOL2 | intronic | 2q33.1 | Score=12692;Name="2573939:MER41-int(LTR)" | bnd_48 | transl_intra_979 |
| 30T | 2 | 201452943 | 201452943 | AOX1 | intronic | 2q33.1 | Score=1096;Name="2573989:L2a(LINE)" | bnd_47 | transl_intra_979 |
| 30T | 4 | 54320360 | 54320360 | FIP1L1 | intronic | 4q12 | Score=1765;Name="3351038:Tigger3a(DNA)" | bnd_50 | transl_intra_1365 |
| 30T | 4 | 67767770 | 67767770 | . | intergenic | 4q13.2 | . | bnd_58 | tandem_dup_2292_0 |
| 30T | 4 | 126200582 | 126200582 | . | intergenic | 4q28.1 | Score=17535;Name="3470666:Charlie3(DNA)" | bnd_49 | transl_intra_1365 |
| 30T | 4 | 126209155 | 126209155 | . | intergenic | 4q28.1 | . | bnd_24 | del_2933 |
| 30T | 4 | 126209695 | 126209695 | . | intergenic | 4q28.1 | Score=15381;Name="3470681:L1PA17(LINE)" | bnd_23 | del_2933 |
| 30T | 4 | 132961424 | 132961424 | . | intergenic | 4q28.3 | . | bnd_57 | tandem_dup_2292_0 |
| 30T | 9 | 140720032 | 140720032 | EHMT1 | intronic | 9q34.3 | . | bnd_26 | del_1091 |
| 30T | 9 | 140721016 | 140721016 | EHMT1 | intronic | 9q34.3 | Score=583;Name="4982100:MLT1A0(LTR)" | bnd_25 | del_1091 |
| 30T | 10 | 59671580 | 59671580 | . | intergenic | 10q21.1 | Score=236;Name="532072:L2(LINE)" | bnd_2 | del_2376 |
| 30T | 10 | 59673460 | 59673460 | . | intergenic | 10q21.1 | Score=579;Name="532074:MLT1J2(LTR)" | bnd_1 | del_2376 |
| 30T | 11 | 108799253 | 108799253 | DDX10 | intronic | 11q22.3 | . | bnd_4 | del_3297 |
| 30T | 11 | 108799992 | 108799992 | DDX10 | intronic | 11q22.3 | . | bnd_3 | del_3297 |
| 30T | 11 | 129356531 | 129356531 | . | intergenic | 11q24.3 | Score=4832;Name="901344:L1MA9(LINE)" | bnd_52 | tandem_dup_3805 |
| 30T | 11 | 129554582 | 129554582 | . | intergenic | 11q24.3 | Score=533;Name="901702:L1ME1(LINE)" | bnd_51 | tandem_dup_3805 |
| 30T | 12 | 12705023 | 12705023 | DUSP16 | intronic | 12p13.2 | Score=4280;Name="933874:Charlie1a(DNA)" | bnd_6 | del_1351 |

| | | | | | | | | | |
|---|---|---|---|---|---|---|---|---|---|
| 30T | 12 | 20090142 | 20090142 | . | intergenic | 12p12.2 | Score=2359;Name="946597:L2a(LINE)" | bnd_5 | del_1351 |
| 30T | 12 | 62960274 | 62960274 | MON2 | intronic | 12q14.1 | . | bnd_28 | del_insod_3426_0/1243_0 |
| 30T | 12 | 64213412 | 64213412 | . | intergenic | 12q14.2 | Score=9836;Name="1018652:L1M1(LINE)" | bnd_30 | del_insod_3426_0/1243_0 |
| 30T | 12 | 64219977 | 64219977 | . | intergenic | 12q14.2 | . | bnd_29 | del_insod_3426_0/1243_0 |
| 30T | 12 | 72956524 | 72956524 | TRHDE | intronic | 12q21.1 | . | bnd_27 | del_insod_3426_0/1243_0 |
| 30T | 17 | 39313318 | 39313318 | . | intergenic | 17q21.2 | Score=343;Name="1882413:MIRb(SINE)" | bnd_8 | del_2170 |
| 30T | 17 | 39322252 | 39322252 | . | intergenic | 17q21.2 | Score=6280;Name="1882432:L1PA2(LINE)" | bnd_7 | del_2170 |
| 30T | 17 | 72387865 | 72387865 | . | intergenic | 17q25.1 | Score=1837;Name="1951429:MLT1E2(LTR)" | bnd_54 | tandem_dup_3446 |
| 30T | 17 | 72546115 | 72546115 | . | intergenic | 17q25.1 | . | bnd_53 | tandem_dup_3446 |
| 30T | 19 | 7736623 | 7736623 | . | intergenic | 19p13.2 | Score=2218;Name="2118035:AluSx1(SINE)" | bnd_36 | transl_intra_3888_0 |
| 30T | 19 | 12837757 | 12837757 | . | intergenic | 19p13.2 | . | bnd_44 | transl_intra_901_0 |
| 30T | 19 | 13664831 | 13664831 | . | intergenic | 19p13.2 | Score=281;Name="2133936:MER21C(LTR)" | bnd_10 | del_2072 |
| 30T | 19 | 16945780 | 16945780 | SIN3B | intronic | 19p13.11 | Score=2246;Name="2143055:AluSp(SINE)" | bnd_43 | transl_intra_901_0 |
| 30T | 19 | 20029378 | 20029378 | ZNF93 | intronic | 19p12 | Score=24718;Name="2150890:L1PA5(LINE)" | bnd_12 | del_2039_0 |
| 30T | 19 | 21664992 | 21664992 | . | intergenic | 19p12 | . | bnd_38 | transl_intra_1184_0 |
| 30T | 19 | 23948205 | 23948205 | RPSAP58 | ncRNA_intronic | 19p12 | Score=4078;Name="2158318:L1PA13(LINE)" | bnd_37 | transl_intra_1184_0 |
| 30T | 19 | 32264525 | 32264525 | . | intergenic | 19q12 | . | bnd_14 | del_2273 |
| 30T | 19 | 32265102 | 32265102 | . | intergenic | 19q12 | Score=1659;Name="2166275:L1MA9(LINE)" | bnd_13 | del_2273 |
| 30T | 19 | 34650137 | 34650137 | . | intergenic | 19q13.11 | Score=2153;Name="2171444:MSTA(LTR)" | bnd_40 | transl_intra_1044_0 |
| 30T | 19 | 35142499 | 35142499 | . | intergenic | 19q13.11 | Score=10181;Name="2172515:L1MA5(LINE)" | bnd_11 | del_2039_0 |
| 30T | 19 | 40589284 | 40589284 | ZNF780A | intronic | 19q13.2 | . | bnd_42 | transl_intra_1158_0 |
| 30T | 19 | 42753888 | 42753888 | ERF | exonic | 19q13.2 | . | bnd_46 | transl_intra_2848 |
| 30T | 19 | 47105882 | 47105882 | CALM3 | intronic | 19q13.32 | . | bnd_39 | transl_intra_1044_0 |
| 30T | 19 | 52732261 | 52732261 | . | intergenic | 19q13.41 | . | bnd_41 | transl_intra_1158_0 |
| 30T | 19 | 54384816 | 54384816 | . | upstream | 19q13.42 | . | bnd_9 | del_2072 |
| 30T | 19 | 7926622 | 7926622 | EVI5L | intronic | 19p13.2 | . | bnd_35 | transl_intra_3888_0 |
| 30T | 19 | 52800655 | 52800655 | ZNF480 | intronic | 19q13.41 | . | bnd_45 | transl_intra_2848 |
| 30T | 20 | 3437805 | 3437805 | . | intergenic | 20p13 | . | bnd_20 | del_2323 |
| 30T | 20 | 17197290 | 17197290 | . | intergenic | 20p12.1 | . | bnd_19 | del_2323 |
| 30T | 22 | 40890535 | 40890535 | MKL1 | intronic | 22q13.1 | Score=1924;Name="2887294:AluJb(SINE)" | bnd_22 | del_1510 |
| 30T | 22 | 40891641 | 40891641 | MKL1 | intronic | 22q13.1 | Score=2234;Name="2887298:AluSx1(SINE)" | bnd_21 | del_1510 |
| 31T | 5 | 42505121 | 42505121 | GHR | intronic | 5p12 | . | bnd_1 | transl_inter_3686_0 |
| 31T | 10 | 11918062 | 11918062 | PROSER2-AS1 | ncRNA_intronic | 10p14 | . | bnd_2 | transl_inter_3686_0 |
| 32T | 1 | 34661569 | 34661569 | C1orf94 | intronic | 1p34.3 | . | bnd_142 | del_inssd_1538_0/2408_0 |
| 32T | 1 | 34661832 | 34661832 | C1orf94 | intronic | 1p34.3 | . | bnd_140 | del_inssd_1538_0/2408_0 |
| 32T | 1 | 34676827 | 34676827 | C1orf94 | intronic | 1p34.3 | . | bnd_148 | transl_intra_1100_0 |
| 32T | 1 | 35663882 | 35663882 | . | intergenic | 1p34.3 | Score=312;Name="78774:L2a(LINE)" | bnd_143 | del_inssu_2656_0/974_0 |
| 32T | 1 | 36026850 | 36026850 | NCDN | exonic | 1p34.3 | . | bnd_238 | transl_inter_6389 |
| 32T | 1 | 36747244 | 36747244 | THRAP3 | intronic | 1p34.3 | . | bnd_2 | del_2623 |
| 32T | 1 | 38272835 | 38272835 | YRDC | exonic | 1p34.3 | . | bnd_1 | del_2623 |
| 32T | 1 | 38809876 | 38809876 | . | intergenic | 1p34.3 | Score=214;Name="86029:L2c(LINE)" | bnd_145 | del_inssu_2656_0/974_0 |
| 32T | 1 | 39458322 | 39458322 | AKIRIN1 | intronic | 1p34.3 | . | bnd_141 | del_inssd_1538_0/2408_0 |
| 32T | 1 | 44088883 | 44088883 | PTPRF | UTR3 | 1p34.2 | . | bnd_147 | transl_intra_1100_0 |

| | | | | | | | | |
|---|---|---|---|---|---|---|---|---|
| 32T | 1 | 46002698 | 46002698 | . | intergenic | 1p34.1 | . | bnd_160 | tandem_dup_2179_0 |
| 32T | 1 | 46006923 | 46006923 | . | intergenic | 1p34.1 | Score=1868;Name="102505:AluJb(SINE)" | bnd_240 | transl_inter_1185 |
| 32T | 1 | 47013078 | 47013078 | KNCN,MKNK1-AS1 | ncRNA_intronic | 1p33 | . | bnd_146 | del_inssu_2656_0/974_0 |
| 32T | 1 | 48529325 | 48529325 | . | intergenic | 1p33 | . | bnd_139 | del_inssd_1538_0/2408_0 |
| 32T | 1 | 119362522 | 119362522 | . | intergenic | 1p12 | Score=5016;Name="239613:L1PA16(LINE)" | bnd_154 | transl_intra_469 |
| 32T | 1 | 119365926 | 119365926 | . | intergenic | 1p12 | . | bnd_153 | transl_intra_469 |
| 32T | 1 | 119789961 | 119789961 | . | intergenic | 1p12 | . | bnd_150 | transl_intra_2671_0 |
| 32T | 1 | 119791444 | 119791444 | . | intergenic | 1p12 | . | bnd_149 | transl_intra_2671_0 |
| 32T | 1 | 145035575 | 145035575 | NBPF9,PDE4DIP,NBPF20 | intronic | 1q21.1 | . | bnd_242 | transl_inter_3131 |
| 32T | 1 | 160264736 | 160264736 | COPA | intronic | 1q23.2 | . | bnd_4 | del_2067 |
| 32T | 1 | 160271174 | 160271174 | COPA | intronic | 1q23.2 | Score=1096;Name="273470:Tigger4a(DNA)" | bnd_3 | del_2067 |
| 32T | 1 | 160275532 | 160275532 | COPA | exonic | 1q23.2 | . | bnd_159 | tandem_dup_2179_0 |
| 32T | 1 | 198304519 | 198304519 | . | intergenic | 1q31.3 | . | bnd_6 | del_22 |
| 32T | 1 | 198329722 | 198329722 | . | intergenic | 1q31.3 | . | bnd_5 | del_22 |
| 32T | 1 | 214416733 | 214416733 | . | intergenic | 1q32.3 | . | bnd_162 | tandem_dup_1839 |
| 32T | 1 | 214456038 | 214456038 | SMYD2 | intronic | 1q32.3 | . | bnd_161 | tandem_dup_1839 |
| 32T | 1 | 236163417 | 236163417 | NID1 | intronic | 1q42.3 | Score=2235;Name="408812:AluSx1(SINE)" | bnd_164 | tandem_dup_3773 |
| 32T | 1 | 236451834 | 236451834 | . | intergenic | 1q42.3 | Score=3588;Name="409431:LTR9(LTR)" | bnd_163 | tandem_dup_3773 |
| 32T | 1 | 246249826 | 246249826 | SMYD3 | intronic | 1q44 | . | bnd_166 | tandem_dup_2028 |
| 32T | 1 | 246303482 | 246303482 | SMYD3 | intronic | 1q44 | . | bnd_165 | tandem_dup_2028 |
| 32T | 1 | 44444798 | 44444798 | . | upstream;downstream | 1p34.1 | Score=205;Name="98846:G-rich(Low_complexity)" | bnd_144 | del_inssu_2656_0/974_0 |
| 32T | 2 | 38572108 | 38572108 | ATL2 | intronic | 2p22.2 | . | bnd_22 | del_4296 |
| 32T | 2 | 38660911 | 38660911 | . | intergenic | 2p22.1 | Score=370;Name="2302831:L2c(LINE)" | bnd_21 | del_4296 |
| 32T | 2 | 41448418 | 41448418 | . | intergenic | 2p22.1 | Score=1067;Name="2307920:L1MC1(LINE)" | bnd_24 | del_4170 |
| 32T | 2 | 41548392 | 41548392 | . | intergenic | 2p22.1 | . | bnd_23 | del_4170 |
| 32T | 2 | 72842783 | 72842783 | EXOC6B | intronic | 2p13.2 | Score=2557;Name="2364445:Tigger3b(DNA)" | bnd_186 | tandem_dup_2793 |
| 32T | 2 | 72882582 | 72882582 | EXOC6B | intronic | 2p13.2 | Score=517;Name="2364529:L1M6(LINE)" | bnd_185 | tandem_dup_2793 |
| 32T | 2 | 109886396 | 109886396 | SH3RF3 | intronic | 2q12.3 | . | bnd_26 | del_498 |
| 32T | 2 | 109914769 | 109914769 | SH3RF3 | intronic | 2q12.3 | . | bnd_25 | del_498 |
| 32T | 2 | 133584948 | 133584948 | MIR7853,NCKAP5 | ncRNA_intronic | 2q21.2 | Score=477;Name="2463484:L1M(LINE)" | bnd_28 | del_315 |
| 32T | 2 | 134185880 | 134185880 | MIR7853,NCKAP5 | ncRNA_intronic | 2q21.2 | Score=708;Name="2464419:L1ME4a(LINE)" | bnd_27 | del_315 |
| 32T | 2 | 134895289 | 134895289 | . | intergenic | 2q21.2 | . | bnd_30 | del_3805 |
| 32T | 2 | 135015836 | 135015836 | MGAT5 | intronic | 2q21.2 | . | bnd_29 | del_3805 |
| 32T | 2 | 141225273 | 141225273 | LRP1B | intronic | 2q22.1 | Score=5556;Name="2476480:MLT1A0-int(LTR)" | bnd_188 | tandem_dup_3202 |
| 32T | 2 | 141264491 | 141264491 | LRP1B | splicing | 2q22.1 | . | bnd_187 | tandem_dup_3202 |
| 32T | 2 | 141592075 | 141592075 | LRP1B | intronic | 2q22.1 | Score=2239;Name="2477077:AluSc(SINE)" | bnd_190 | tandem_dup_4063 |
| 32T | 2 | 141608831 | 141608831 | LRP1B | intronic | 2q22.1 | . | bnd_189 | tandem_dup_4063 |
| 32T | 2 | 141797390 | 141797390 | LRP1B | intronic | 2q22.1 | . | bnd_114 | del_ins_1159 |
| 32T | 2 | 141958020 | 141958020 | LRP1B | intronic | 2q22.1 | Score=3923;Name="2477674:L1PA10(LINE)" | bnd_113 | del_ins_1159 |
| 32T | 2 | 197484728 | 197484728 | . | intergenic | 2q33.1 | Score=273;Name="2567220:L2(LINE)" | bnd_32 | del_424 |

| | | | | | | | | |
|---|---|---|---|---|---|---|---|---|
| 32T | 2 | 197494618 | 197494618 | . | intergenic | 2q33.1 | Score=7871;Name="2567244:L1MA9(LINE)" | bnd_31 | del_424 |
| 32T | 2 | 227444450 | 227444450 | . | intergenic | 2q36.3 | Score=1304;Name="2620835:L1MD(LINE)" | bnd_192 | tandem_dup_2199 |
| 32T | 2 | 227456521 | 227456521 | . | intergenic | 2q36.3 | Score=22024;Name="2620847:L1PA11(LINE)" | bnd_191 | tandem_dup_2199 |
| 32T | 3 | 11740767 | 11740767 | VGLL4 | intronic | 3p25.3 | . | bnd_42 | del_834 |
| 32T | 3 | 11754729 | 11754729 | VGLL4 | intronic | 3p25.3 | . | bnd_41 | del_834 |
| 32T | 3 | 18402383 | 18402383 | SATB1 | intronic | 3p24.3 | . | bnd_44 | del_532 |
| 32T | 3 | 18444999 | 18444999 | SATB1 | intronic | 3p24.3 | . | bnd_43 | del_532 |
| 32T | 3 | 36993609 | 36993609 | . | intergenic | 3p22.2 | Score=2198;Name="2976216:AluSq2(SINE)" | bnd_204 | tandem_dup_3785 |
| 32T | 3 | 37090955 | 37090955 | MLH1 | intronic | 3p22.2 | . | bnd_203 | tandem_dup_3785 |
| 32T | 3 | 42654155 | 42654155 | NKTR | intronic | 3p22.1 | Score=350;Name="2986648:HAL1b(LINE)" | bnd_46 | del_3757 |
| 32T | 3 | 42679844 | 42679844 | NKTR | exonic | 3p22.1 | . | bnd_45 | del_3757 |
| 32T | 3 | 42680554 | 42680554 | NKTR | exonic | 3p22.1 | . | bnd_48 | del_3808 |
| 32T | 3 | 42707391 | 42707391 | ZBTB47 | UTR3 | 3p22.1 | . | bnd_47 | del_3808 |
| 32T | 3 | 71132897 | 71132897 | FOXP1 | intronic | 3p13 | Score=1297;Name="3040394:MLT1K(LTR)" | bnd_50 | del_1482 |
| 32T | 3 | 71624978 | 71624978 | FOXP1 | intronic | 3p13 | . | bnd_49 | del_1482 |
| 32T | 3 | 85328834 | 85328834 | CADM2 | intronic | 3p12.1 | Score=27;Name="3064455:AT_rich(Low_complexity)" | bnd_52 | del_1526 |
| 32T | 3 | 85333745 | 85333745 | CADM2 | intronic | 3p12.1 | Score=1323;Name="3064463:L2a(LINE)" | bnd_51 | del_1526 |
| 32T | 3 | 171852413 | 171852413 | FNDC3B | intronic | 3q26.31 | . | bnd_54 | del_891 |
| 32T | 3 | 171857141 | 171857141 | FNDC3B | intronic | 3q26.31 | . | bnd_53 | del_891 |
| 32T | 3 | 174534789 | 174534789 | . | intergenic | 3q26.31 | . | bnd_56 | del_4117 |
| 32T | 3 | 174578113 | 174578113 | NAALADL2 | intronic | 3q26.31 | . | bnd_55 | del_4117 |
| 32T | 4 | 55894102 | 55894102 | . | intergenic | 4q12 | Score=1387;Name="3353922:L1MD2(LINE)" | bnd_58 | del_862 |
| 32T | 4 | 55951631 | 55951631 | KDR | intronic | 4q12 | . | bnd_57 | del_862 |
| 32T | 4 | 106441567 | 106441567 | . | intergenic | 4q24 | Score=1196;Name="3438505:MER2(DNA)" | bnd_206 | tandem_dup_3024 |
| 32T | 4 | 106621557 | 106621557 | INTS12 | intronic | 4q24 | . | bnd_205 | tandem_dup_3024 |
| 32T | 4 | 149036029 | 149036029 | NR3C2 | intronic | 4q31.23 | . | bnd_208 | tandem_dup_2575 |
| 32T | 4 | 149070262 | 149070262 | NR3C2 | intronic | 4q31.23 | . | bnd_207 | tandem_dup_2575 |
| 32T | 4 | 181654699 | 181654699 | . | intergenic | 4q34.3 | . | bnd_210 | tandem_dup_4867 |
| 32T | 4 | 181761165 | 181761165 | . | intergenic | 4q34.3 | Score=1687;Name="3562464:MLT2D(LTR)" | bnd_209 | tandem_dup_4867 |
| 32T | 4 | 182185830 | 182185830 | . | intergenic | 4q34.3 | . | bnd_60 | del_4830 |
| 32T | 4 | 182392491 | 182392491 | . | intergenic | 4q34.3 | . | bnd_59 | del_4830 |
| 32T | 5 | 26381570 | 26381570 | . | intergenic | 5p14.1 | Score=1978;Name="3620739:MER65A(LTR)" | bnd_62 | del_1469 |
| 32T | 5 | 26403292 | 26403292 | . | intergenic | 5p14.1 | Score=41580;Name="3620768:HERVL-int(LTR)" | bnd_61 | del_1469 |
| 32T | 5 | 63316967 | 63316967 | . | intergenic | 5q12.3 | . | bnd_64 | del_1342 |
| 32T | 5 | 63326593 | 63326593 | . | intergenic | 5q12.3 | . | bnd_63 | del_1342 |
| 32T | 5 | 76746373 | 76746373 | WDR41 | intronic | 5q13.3 | Score=259;Name="3701764:L2c(LINE)" | bnd_212 | tandem_dup_3004_0 |
| 32T | 5 | 76866590 | 76866590 | . | intergenic | 5q13.3 | . | bnd_211 | tandem_dup_3004_0 |
| 32T | 5 | 78674178 | 78674178 | HOMER1 | intronic | 5q14.1 | . | bnd_214 | tandem_dup_2175 |
| 32T | 5 | 78831178 | 78831178 | . | intergenic | 5q14.1 | . | bnd_213 | tandem_dup_2175 |
| 32T | 5 | 113470726 | 113470726 | . | intergenic | 5q22.3 | Score=816;Name="3760541:HAL1(LINE)" | bnd_66 | del_2979 |
| 32T | 5 | 113518704 | 113518704 | . | intergenic | 5q22.3 | Score=5383;Name="3760606:L1ME2z(LINE)" | bnd_65 | del_2979 |
| 32T | 5 | 128074880 | 128074880 | . | intergenic | 5q23.3 | Score=2362;Name="3783638:L1ME1(LINE)" | bnd_68 | del_3286 |

| | | | | | | | | | |
|---|---|---|---|---|---|---|---|---|---|
| 32T | 5 | 128079534 | 128079534 | . | intergenic | 5q23.3 | Score=2346;Name="3783648:AluSq2(SINE)" | bnd_67 | del_3286 |
| 32T | 5 | 179283534 | 179283534 | C5orf45 | intronic | 5q35.3 | Score=1758;Name="3884157:AluJb(SINE)" | bnd_124 | del_ins_2210 |
| 32T | 5 | 179338814 | 179338814 | . | intergenic | 5q35.3 | . | bnd_123 | del_ins_2210 |
| 32T | 6 | 5382171 | 5382171 | FARS2 | intronic | 6p25.1 | . | bnd_216 | tandem_dup_2883 |
| 32T | 6 | 5492937 | 5492937 | FARS2 | intronic | 6p25.1 | Score=257;Name="3896677:L2a(LINE)" | bnd_215 | tandem_dup_2883 |
| 32T | 6 | 25060117 | 25060117 | . | intergenic | 6p22.3 | . | bnd_218 | tandem_dup_2490 |
| 32T | 6 | 25195463 | 25195463 | . | intergenic | 6p22.3 | . | bnd_217 | tandem_dup_2490 |
| 32T | 6 | 43784893 | 43784893 | . | intergenic | 6p21.1 | Score=441;Name="3970528:L3(LINE)" | bnd_220 | tandem_dup_4124 |
| 32T | 6 | 44003036 | 44003036 | . | intergenic | 6p21.1 | . | bnd_219 | tandem_dup_4124 |
| 32T | 6 | 86395693 | 86395693 | . | intergenic | 6q14.3 | . | bnd_126 | del_ins_2513 |
| 32T | 6 | 86398082 | 86398082 | . | intergenic | 6q14.3 | Score=4030;Name="4033964:MER70-int(LTR)" | bnd_125 | del_ins_2513 |
| 32T | 6 | 145127691 | 145127691 | UTRN | intronic | 6q24.2 | . | bnd_128 | del_ins_4777 |
| 32T | 6 | 145128022 | 145128022 | UTRN | intronic | 6q24.2 | . | bnd_127 | del_ins_4777 |
| 32T | 6 | 162792600 | 162792600 | PARK2 | intronic | 6q26 | Score=636;Name="4162482:MIR(SINE)" | bnd_70 | del_1892 |
| 32T | 6 | 162802671 | 162802671 | PARK2 | intronic | 6q26 | . | bnd_69 | del_1892 |
| 32T | 7 | 4811852 | 4811852 | . | downstream | 7p22.1 | . | bnd_72 | del_2786 |
| 32T | 7 | 4812670 | 4812670 | . | intergenic | 7p22.1 | Score=2379;Name="4234484:AluSz(SINE)" | bnd_71 | del_2786 |
| 32T | 7 | 33052338 | 33052338 | . | intergenic | 7p14.3 | . | bnd_222 | tandem_dup_5044 |
| 32T | 7 | 33094598 | 33094598 | NT5C3A | intronic | 7p14.3 | . | bnd_221 | tandem_dup_5044 |
| 32T | 7 | 40894941 | 40894941 | SUGCT | intronic | 7p14.1 | . | bnd_74 | del_2483 |
| 32T | 7 | 41029306 | 41029306 | . | intergenic | 7p14.1 | Score=2189;Name="4297347:AluSx(SINE)" | bnd_73 | del_2483 |
| 32T | 7 | 69178273 | 69178273 | AUTS2 | intronic | 7q11.22 | Score=1339;Name="4343537:L1MA6(LINE)" | bnd_76 | del_2956 |
| 32T | 7 | 69486574 | 69486574 | AUTS2 | intronic | 7q11.22 | . | bnd_75 | del_2956 |
| 32T | 7 | 86013473 | 86013473 | . | intergenic | 7q21.11 | Score=2972;Name="4377379:L1MD2(LINE)" | bnd_78 | del_1596 |
| 32T | 7 | 86034449 | 86034449 | . | intergenic | 7q21.11 | . | bnd_77 | del_1596 |
| 32T | 7 | 134645373 | 134645373 | CALD1 | exonic | 7q33 | . | bnd_243 | transl_inter_7520 |
| 32T | 8 | 1839934 | 1839934 | ARHGEF10 | intronic | 8p23.3 | . | bnd_224 | tandem_dup_3284 |
| 32T | 8 | 1888165 | 1888165 | ARHGEF10 | intronic | 8p23.3 | . | bnd_223 | tandem_dup_3284 |
| 32T | 8 | 3358636 | 3358636 | CSMD1 | intronic | 8p23.2 | . | bnd_80 | del_1219 |
| 32T | 8 | 3359293 | 3359293 | CSMD1 | intronic | 8p23.2 | . | bnd_79 | del_1219 |
| 32T | 8 | 3359574 | 3359574 | CSMD1 | intronic | 8p23.2 | Score=3432;Name="4510360:L1MC1(LINE)" | bnd_82 | del_2753 |
| 32T | 8 | 3361912 | 3361912 | CSMD1 | intronic | 8p23.2 | Score=341;Name="4510365:MIRb(SINE)" | bnd_158 | transl_intra_1549_0 |
| 32T | 8 | 3460598 | 3460598 | CSMD1 | intronic | 8p23.2 | . | bnd_157 | transl_intra_1549_0 |
| 32T | 8 | 3465235 | 3465235 | CSMD1 | intronic | 8p23.2 | . | bnd_81 | del_2753 |
| 32T | 8 | 3634169 | 3634169 | CSMD1 | intronic | 8p23.2 | . | bnd_84 | del_1046 |
| 32T | 8 | 3642466 | 3642466 | CSMD1 | intronic | 8p23.2 | . | bnd_83 | del_1046 |
| 32T | 8 | 3667046 | 3667046 | CSMD1 | intronic | 8p23.2 | . | bnd_86 | del_1667 |
| 32T | 8 | 3716969 | 3716969 | CSMD1 | intronic | 8p23.2 | Score=1783;Name="4510919:MER77B(LTR)" | bnd_130 | del_ins_1724 |
| 32T | 8 | 3790153 | 3790153 | CSMD1 | intronic | 8p23.2 | . | bnd_129 | del_ins_1724 |
| 32T | 8 | 3839962 | 3839962 | CSMD1 | intronic | 8p23.2 | Score=617;Name="4511090:MIR(SINE)" | bnd_85 | del_1667 |
| 32T | 8 | 4148240 | 4148240 | CSMD1 | intronic | 8p23.2 | . | bnd_237 | transl_inter_6389 |
| 32T | 8 | 11060570 | 11060570 | . | intergenic | 8p23.1 | . | bnd_87 | del_7002 |
| 32T | 8 | 16850365 | 16850365 | FGF20 | UTR3 | 8p22 | . | bnd_226 | tandem_dup_1849 |
| 32T | 8 | 17671119 | 17671119 | . | intergenic | 8p22 | Score=2137;Name="4534323:THE1B(LTR)" | bnd_228 | tandem_dup_975 |
| 32T | 8 | 17760770 | 17760770 | . | intergenic | 8p22 | . | bnd_225 | tandem_dup_1849 |

| | | | | | | | | | | |
|---|---|---|---|---|---|---|---|---|---|---|
| 32T | 8 | 17766786 | 17766786 | . | intergenic | 8p22 | Score=19127;Name="4534532:HERVK-int(LTR)" | bnd_227 | tandem_dup_975 |
| 32T | 8 | 17768408 | 17768408 | . | intergenic | 8p22 | Score=19127;Name="4534532:HERVK-int(LTR)" | bnd_239 | transl_inter_1185 |
| 32T | 8 | 43008993 | 43008993 | HGSNAT | intronic | 8p11.21 | Score=3118;Name="4582802:L1MA6(LINE)" | bnd_90 | del_5961 |
| 32T | 8 | 43159549 | 43159549 | POTEA | intronic | 8p11.1 | . | bnd_89 | del_5961 |
| 32T | 8 | 68198315 | 68198315 | ARFGEF1 | intronic | 8q13.2 | Score=2440;Name="4618099:L1MC4(LINE)" | bnd_132 | del_ins_4901 |
| 32T | 8 | 68215044 | 68215044 | ARFGEF1 | intronic | 8q13.2 | Score=1458;Name="4618131:AluJr4(SINE)" | bnd_131 | del_ins_4901 |
| 32T | 8 | 90505557 | 90505557 | . | intergenic | 8q21.3 | Score=314;Name="4655453:HAL1(LINE)" | bnd_92 | del_3266 |
| 32T | 8 | 90633859 | 90633859 | . | intergenic | 8q21.3 | Score=2144;Name="4655667:Tigger10(DNA)" | bnd_91 | del_3266 |
| 32T | 8 | 99734994 | 99734994 | STK3 | intronic | 8q22.2 | . | bnd_230 | tandem_dup_3676 |
| 32T | 8 | 99786438 | 99786438 | STK3 | intronic | 8q22.2 | . | bnd_229 | tandem_dup_3676 |
| 32T | 8 | 145843262 | 145843262 | . | intergenic | 8q24.3 | Score=1983;Name="4757915:AluJb(SINE)" | bnd_94 | del_3223 |
| 32T | 8 | 145904660 | 145904660 | . | intergenic | 8q24.3 | . | bnd_93 | del_3223 |
| 32T | 8 | 11058630 | 11058630 | XKR6 | exonic | 8p23.1 | . | bnd_88 | del_7002 |
| 32T | 9 | 3408476 | 3408476 | RFX3 | intronic | 9p24.2 | . | bnd_96 | del_4150 |
| 32T | 9 | 3409003 | 3409003 | RFX3 | intronic | 9p24.2 | . | bnd_95 | del_4150 |
| 32T | 9 | 3410741 | 3410741 | RFX3 | intronic | 9p24.2 | . | bnd_138 | transl_intra_3450_0 |
| 32T | 9 | 3583190 | 3583190 | . | intergenic | 9p24.2 | Score=3871;Name="4765060:L1MDb(LINE)" | bnd_137 | transl_intra_3450_0 |
| 32T | 9 | 3604862 | 3604862 | . | intergenic | 9p24.2 | Score=4431;Name="4765112:MER39B(LTR)" | bnd_232 | tandem_dup_4892 |
| 32T | 9 | 3846412 | 3846412 | GLIS3 | intronic | 9p24.2 | . | bnd_231 | tandem_dup_4892 |
| 32T | 9 | 21949526 | 21949526 | . | intergenic | 9p21.3 | Score=2588;Name="4795802:MSTA(LTR)" | bnd_134 | del_ins_1070 |
| 32T | 9 | 22024843 | 22024843 | CDKN2B-AS1 | ncRNA_intronic | 9p21.3 | Score=12632;Name="4795919:L1PA13(LINE)" | bnd_133 | del_ins_1070 |
| 32T | 9 | 97768708 | 97768708 | C9orf3 | intronic | 9q22.32 | . | bnd_98 | del_842 |
| 32T | 9 | 97869735 | 97869735 | FANCC | intronic | 9q22.32 | . | bnd_97 | del_842 |
| 32T | 9 | 119066095 | 119066095 | PAPPA | intronic | 9q33.1 | . | bnd_100 | del_4990 |
| 32T | 9 | 119168019 | 119168019 | . | intergenic | 9q33.1 | . | bnd_99 | del_4990 |
| 32T | 9 | 127062082 | 127062082 | NEK6 | intronic | 9q33.3 | Score=234;Name="4954484:MADE2(DNA)" | bnd_234 | tandem_dup_2372 |
| 32T | 9 | 127084654 | 127084654 | NEK6 | intronic | 9q33.3 | . | bnd_233 | tandem_dup_2372 |
| 32T | 10 | 5328828 | 5328828 | . | intergenic | 10p15.1 | Score=461;Name="440620:MER8(DNA)" | bnd_168 | tandem_dup_4310 |
| 32T | 10 | 5374600 | 5374600 | . | intergenic | 10p15.1 | Score=3844;Name="440716:L1MA6(LINE)" | bnd_167 | tandem_dup_4310 |
| 32T | 10 | 90202103 | 90202103 | RNLS | intronic | 10q23.31 | . | bnd_8 | del_2708 |
| 32T | 10 | 90226486 | 90226486 | RNLS | intronic | 10q23.31 | . | bnd_7 | del_2708 |
| 32T | 10 | 96791423 | 96791423 | . | intergenic | 10q23.33 | . | bnd_170 | tandem_dup_3863 |
| 32T | 10 | 96998436 | 96998436 | PDLIM1 | exonic | 10q23.33 | . | bnd_169 | tandem_dup_3863 |
| 32T | 11 | 19823475 | 19823475 | NAV2 | intronic | 11p15.1 | . | bnd_10 | del_1648 |
| 32T | 11 | 19846530 | 19846530 | NAV2 | intronic | 11p15.1 | . | bnd_9 | del_1648 |
| 32T | 11 | 35146062 | 35146062 | . | intergenic | 11p13 | Score=593;Name="732880:MIRb(SINE)" | bnd_244 | transl_inter_7520 |
| 32T | 11 | 47428557 | 47428557 | . | intergenic | 11p11.2 | . | bnd_241 | transl_inter_3131 |
| 32T | 11 | 81255883 | 81255883 | . | intergenic | 11q14.1 | Score=885;Name="816187:MER3(DNA)" | bnd_108 | del_ins_2117 |
| 32T | 11 | 81261970 | 81261970 | . | intergenic | 11q14.1 | Score=3087;Name="816200:L1P4(LINE)" | bnd_107 | del_ins_2117 |
| 32T | 11 | 100748308 | 100748308 | ARHGAP42 | intronic | 11q22.1 | . | bnd_12 | del_4097 |

| | | | | | | | | |
|---|---|---|---|---|---|---|---|---|
| 32T 11 | 100804960 | 100804960 | ARHGAP42 | intronic | 11q22.1 | . | bnd_11 | del_4097 |
| 32T 12 | 23871327 | 23871327 | SOX5 | intronic | 12p12.1 | . | bnd_14 | del_1245 |
| 32T 12 | 23875114 | 23875114 | SOX5 | intronic | 12p12.1 | . | bnd_110 | del_ins_3410 |
| 32T 12 | 23875568 | 23875568 | SOX5 | intronic | 12p12.1 | . | bnd_109 | del_ins_3410 |
| 32T 12 | 24212440 | 24212440 | SOX5 | intronic | 12p12.1 | . | bnd_13 | del_1245 |
| 32T 12 | 118752068 | 118752068 | TAOK3 | intronic | 12q24.23 | Score=793;Name="1127218:Tigger16a(DNA)" | bnd_172 | tandem_dup_3276 |
| 32T 12 | 118762467 | 118762467 | TAOK3 | intronic | 12q24.23 | Score=5538;Name="1127248:L1MA3(LINE)" | bnd_171 | tandem_dup_3276 |
| 32T 12 | 123132374 | 123132374 | . | intergenic | 12q24.31 | Score=658;Name="1138625:MLT1C(LTR)" | bnd_174 | tandem_dup_5815 |
| 32T 12 | 123252228 | 123252228 | DENR | intronic | 12q24.31 | Score=258;Name="1138985:(TC)n(Simple_repeat)" | bnd_173 | tandem_dup_5815 |
| 32T 14 | 29285812 | 29285812 | . | intergenic | 14q12 | . | bnd_176 | tandem_dup_4045 |
| 32T 14 | 29298890 | 29298890 | . | intergenic | 14q12 | Score=417;Name="1338833:(TA)n(Simple_repeat)" | bnd_175 | tandem_dup_4045 |
| 32T 14 | 37846090 | 37846090 | MIPOL1 | intronic | 14q21.1 | Score=2621;Name="1354149:L1MC1(LINE)" | bnd_16 | del_1604 |
| 32T 14 | 37855499 | 37855499 | MIPOL1 | intronic | 14q21.1 | . | bnd_15 | del_1604 |
| 32T 15 | 35737333 | 35737333 | DPH6 | intronic | 15q14 | . | bnd_18 | del_995 |
| 32T 15 | 35806860 | 35806860 | DPH6 | intronic | 15q14 | . | bnd_17 | del_995 |
| 32T 15 | 58726490 | 58726490 | LIPC | intronic | 15q21.3 | . | bnd_156 | transl_intra_1705 |
| 32T 15 | 58759768 | 58759768 | LIPC | intronic | 15q21.3 | Score=249;Name="1550001:CR1_Mam(LINE)" | bnd_152 | transl_intra_1399_0 |
| 32T 15 | 58940174 | 58940174 | ADAM10 | intronic | 15q21.3 | . | bnd_155 | transl_intra_1705 |
| 32T 15 | 58950167 | 58950167 | ADAM10 | intronic | 15q21.3 | Score=4542;Name="1550381:L1M5(LINE)" | bnd_151 | transl_intra_1399_0 |
| 32T 15 | 83450659 | 83450659 | FSD2 | intronic | 15q25.2 | Score=2391;Name="1600020:AluSg(SINE)" | bnd_178 | tandem_dup_2860 |
| 32T 15 | 83590677 | 83590677 | HOMER2 | intronic | 15q25.2 | Score=320;Name="1600303:HAL1(LINE)" | bnd_177 | tandem_dup_2860 |
| 32T 15 | 97963227 | 97963227 | . | intergenic | 15q26.2 | . | bnd_112 | del_ins_1301 |
| 32T 15 | 97964387 | 97964387 | . | intergenic | 15q26.2 | Score=4057;Name="1627060:L1M4b(LINE)" | bnd_111 | del_ins_1301 |
| 32T 16 | 78667199 | 78667199 | WWOX | intronic | 16q23.1 | Score=329;Name="1781527:MIR(SINE)" | bnd_20 | del_3588 |
| 32T 16 | 78779758 | 78779758 | WWOX | intronic | 16q23.1 | . | bnd_19 | del_3588 |
| 32T 17 | 80636342 | 80636342 | RAB40B | intronic | 17q25.3 | . | bnd_180 | tandem_dup_3404 |
| 32T 17 | 80687356 | 80687356 | . | intergenic | 17q25.3 | . | bnd_179 | tandem_dup_3404 |
| 32T 19 | 685237 | 685237 | . | downstream | 19p13.3 | . | bnd_182 | tandem_dup_2016 |
| 32T 19 | 753295 | 753295 | MISP | intronic | 19p13.3 | . | bnd_181 | tandem_dup_2016 |
| 32T 19 | 39362796 | 39362796 | RINL | intronic | 19q13.2 | Score=473;Name="2182898:L2a(LINE)" | bnd_184 | tandem_dup_6056 |
| 32T 19 | 39364789 | 39364789 | RINL | intronic | 19q13.2 | Score=918;Name="2182907:MLT1K(LTR)" | bnd_183 | tandem_dup_6056 |
| 32T 20 | 3621340 | 3621340 | ATRN | intronic | 20p13 | Score=3596;Name="2656765:L1MB3(LINE)" | bnd_194 | tandem_dup_3106 |
| 32T 20 | 3755959 | 3755959 | . | intergenic | 20p13 | Score=25;Name="2657013:AT_rich(Low_complexity)" | bnd_193 | tandem_dup_3106 |
| 32T 20 | 14298147 | 14298147 | MACROD2 | intronic | 20p12.1 | Score=2274;Name="2676625:AluSc(SINE)" | bnd_196 | tandem_dup_4387 |
| 32T 20 | 14322363 | 14322363 | MACROD2 | intronic | 20p12.1 | Score=862;Name="2676649:MLT1K(LTR)" | bnd_195 | tandem_dup_4387 |
| 32T 20 | 14878395 | 14878395 | MACROD2-AS1,MACROD2 | ncRNA_intronic | 20p12.1 | Score=1811;Name="2677519:MLT1A0(LTR)" | bnd_116 | del_ins_2165 |

| | | | | | | | | | |
|---|---|---|---|---|---|---|---|---|---|
| 32T | 20 | 14914830 | 14914830 | MACROD2 | intronic | 20p12.1 | Score=182;Name="2677576:L3(LINE)" | bnd_115 | del_ins_2165 |
| 32T | 20 | 15063370 | 15063370 | MACROD2 | intronic | 20p12.1 | . | bnd_34 | del_5846 |
| 32T | 20 | 15064551 | 15064551 | MACROD2 | intronic | 20p12.1 | . | bnd_33 | del_5846 |
| 32T | 20 | 15086426 | 15086426 | MACROD2 | intronic | 20p12.1 | Score=294;Name="2677899:MIRb(SINE)" | bnd_198 | tandem_dup_2029 |
| 32T | 20 | 15094721 | 15094721 | MACROD2 | intronic | 20p12.1 | . | bnd_197 | tandem_dup_2029 |
| 32T | 20 | 15184129 | 15184129 | MACROD2 | intronic | 20p12.1 | . | bnd_118 | del_ins_2864 |
| 32T | 20 | 15190839 | 15190839 | MACROD2 | intronic | 20p12.1 | . | bnd_117 | del_ins_2864 |
| 32T | 20 | 35886682 | 35886682 | . | intergenic | 20q11.23 | . | bnd_200 | tandem_dup_3325 |
| 32T | 20 | 35978573 | 35978573 | SRC | intronic | 20q11.23 | . | bnd_199 | tandem_dup_3325 |
| 32T | 21 | 17909882 | 17909882 | LINC00478 | ncRNA_intronic | 21q21.1 | . | bnd_36 | del_2510 |
| 32T | 21 | 18076594 | 18076594 | . | intergenic | 21q21.1 | . | bnd_35 | del_2510 |
| 32T | 21 | 41689109 | 41689109 | DSCAM | intronic | 21q22.2 | . | bnd_38 | del_4944 |
| 32T | 21 | 41711443 | 41711443 | DSCAM | intronic | 21q22.2 | . | bnd_37 | del_4944 |
| 32T | 22 | 18047132 | 18047132 | SLC25A18 | intronic | 22q11.21 | Score=183;Name="2836231:MIR(SINE)" | bnd_120 | del_ins_1321 |
| 32T | 22 | 18361170 | 18361170 | MICAL3 | intronic | 22q11.21 | . | bnd_119 | del_ins_1321 |
| 32T | 22 | 28448240 | 28448240 | TTC28 | intronic | 22q12.1 | . | bnd_122 | del_ins_3897 |
| 32T | 22 | 28670091 | 28670091 | TTC28 | intronic | 22q12.1 | Score=1040;Name="2858221:L1ME3A(LINE)" | bnd_121 | del_ins_3897 |
| 32T | 22 | 29341062 | 29341062 | ZNRF3 | intronic | 22q12.1 | Score=842;Name="2859803:FLAM_C(SINE)" | bnd_40 | del_3077 |
| 32T | 22 | 29578632 | 29578632 | . | intergenic | 22q12.1 | . | bnd_39 | del_3077 |
| 32T | 22 | 36688969 | 36688969 | MYH9 | intronic | 22q12.3 | Score=791;Name="2877235:Charlie4a(DNA)" | bnd_202 | tandem_dup_2381 |
| 32T | 22 | 36925583 | 36925583 | . | upstream | 22q12.3 | . | bnd_201 | tandem_dup_2381 |
| 32T | X | 9758328 | 9758328 | SHROOM2 | intronic | Xp22.2 | . | bnd_102 | del_1645 |
| 32T | X | 9762428 | 9762428 | SHROOM2 | intronic | Xp22.2 | Score=2365;Name="5010026:AluSp(SINE)" | bnd_101 | del_1645 |
| 32T | X | 20169830 | 20169830 | RPS6KA3 | UTR3 | Xp22.12 | Score=294;Name="5029571:Tigger12A(DNA)" | bnd_104 | del_1009 |
| 32T | X | 20207871 | 20207871 | RPS6KA3 | intronic | Xp22.12 | . | bnd_103 | del_1009 |
| 32T | X | 118889155 | 118889155 | . | intergenic | Xq24 | . | bnd_236 | tandem_dup_4156 |
| 32T | X | 118983153 | 118983153 | UPF3B | intronic | Xq24 | . | bnd_235 | tandem_dup_4156 |
| 32T | X | 128281381 | 128281381 | . | intergenic | Xq25 | Score=1574;Name="5206866:L1ME3C(LINE)" | bnd_136 | del_ins_3679 |
| 32T | X | 128286052 | 128286052 | . | intergenic | Xq25 | Score=2462;Name="5206876:AluSx1(SINE)" | bnd_135 | del_ins_3679 |
| 32T | X | 128417466 | 128417466 | . | intergenic | Xq25 | Score=9785;Name="5207071:L1PB1(LINE)" | bnd_106 | del_3616 |
| 32T | X | 128577067 | 128577067 | . | intergenic | Xq25 | Score=1703;Name="5207330:LTR16C(LTR)" | bnd_105 | del_3616 |
| 33T | 1 | 14215662 | 14215662 | . | intergenic | 1p36.21 | . | bnd_208 | tandem_dup_1745_0 |
| 33T | 1 | 22994508 | 22994508 | . | intergenic | 1p36.12 | . | bnd_207 | tandem_dup_1745_0 |
| 33T | 1 | 89155214 | 89155214 | PKN2 | intronic | 1p22.2 | Score=2213;Name="185813:AluSx(SINE)" | bnd_250 | transl_inter_6604 |
| 33T | 1 | 104500533 | 104500533 | . | intergenic | 1p21.1 | Score=417;Name="213028:LTR86B1(LTR)" | bnd_2 | del_1231 |
| 33T | 1 | 104596328 | 104596328 | . | intergenic | 1p21.1 | Score=3809;Name="213176:L1M4c(LINE)" | bnd_1 | del_1231 |
| 33T | 1 | 176483686 | 176483686 | PAPPA2 | intronic | 1q25.2 | . | bnd_4 | del_1642 |
| 33T | 1 | 176487401 | 176487401 | PAPPA2 | intronic | 1q25.2 | . | bnd_3 | del_1642 |
| 33T | 1 | 236044145 | 236044145 | . | intergenic | 1q42.3 | Score=1648;Name="408519:AluSx1(SINE)" | bnd_209 | tandem_dup_543 |
| 33T | 1 | 236029825 | 236029825 | LYST | intronic | 1q42.3 | . | bnd_210 | tandem_dup_543 |
| 33T | 2 | 49660252 | 49660252 | . | intergenic | 2p16.3 | . | bnd_86 | del_ins_937 |

| | | | | | | | | |
|---|---|---|---|---|---|---|---|---|
| 33T 2 | 49662032 | 49662032 | . | intergenic | 2p16.3 | Score=1935;Name="2323727:L1ME3A(LINE)" | bnd_85 | del_ins_937 |
| 33T 2 | 159832888 | 159832888 | TANC1 | intronic | 2q24.2 | Score=947;Name="2505452:Charlie7(DNA)" | bnd_52 | del_994 |
| 33T 2 | 159833335 | 159833335 | TANC1 | intronic | 2q24.2 | Score=2237;Name="2505453:AluSx(SINE)" | bnd_51 | del_994 |
| 33T 2 | 190350401 | 190350401 | . | intergenic | 2q32.2 | . | bnd_242 | tandem_dup_1444 |
| 33T 2 | 190435519 | 190435519 | SLC40A1 | intronic | 2q32.2 | . | bnd_241 | tandem_dup_1444 |
| 33T 2 | 210122171 | 210122171 | . | intergenic | 2q34 | Score=2663;Name="2590496:MER65A(LTR)" | bnd_54 | del_2070 |
| 33T 2 | 210122665 | 210122665 | . | intergenic | 2q34 | . | bnd_53 | del_2070 |
| 33T 3 | 100780084 | 100780084 | . | intergenic | 3q12.2 | Score=22887;Name="3083452:L1PBa(LINE)" | bnd_56 | del_1944 |
| 33T 3 | 100786302 | 100786302 | . | intergenic | 3q12.2 | . | bnd_55 | del_1944 |
| 33T 4 | 43409779 | 43409779 | . | intergenic | 4p13 | Score=1735;Name="3337576:THE1B(LTR)" | bnd_58 | del_870 |
| 33T 4 | 43410391 | 43410391 | . | intergenic | 4p13 | Score=2090;Name="3337579:THE1C(LTR)" | bnd_57 | del_870 |
| 33T 4 | 71000366 | 71000366 | CSN1S2BP | ncRNA_intronic | 4q13.3 | . | bnd_60 | del_1118 |
| 33T 4 | 136994020 | 136994020 | . | intergenic | 4q28.3 | . | bnd_59 | del_1118 |
| 33T 5 | 55349467 | 55349467 | . | intergenic | 5q11.2 | Score=3060;Name="3663815:LTR1D(LTR)" | bnd_62 | del_1701 |
| 33T 5 | 55349998 | 55349998 | . | intergenic | 5q11.2 | Score=1510;Name="3663816:L1ME1(LINE)" | bnd_61 | del_1701 |
| 33T 5 | 57848861 | 57848861 | LOC101928600 | ncRNA_intronic | 5q11.2 | Score=26461;Name="3668295:L1PA2(LINE)" | bnd_64 | del_1309 |
| 33T 5 | 64397756 | 64397756 | . | intergenic | 5q12.3 | . | bnd_174 | transl_intra_1831_0 |
| 33T 5 | 64401729 | 64401729 | . | intergenic | 5q12.3 | Score=323;Name="3679104:MLT1K(LTR)" | bnd_106 | transl_intra_3638_0 |
| 33T 5 | 64778009 | 64778009 | . | upstream | 5q12.3 | . | bnd_252 | transl_inter_958 |
| 33T 5 | 64825260 | 64825260 | CENPK | intronic | 5q12.3 | . | bnd_105 | transl_intra_3638_0 |
| 33T 5 | 64852649 | 64852649 | CENPK | intronic | 5q12.3 | Score=3014;Name="3679901:Tigger3(DNA)" | bnd_176 | transl_intra_1510_0 |
| 33T 5 | 64852752 | 64852752 | CENPK | intronic | 5q12.3 | Score=3014;Name="3679901:Tigger3(DNA)" | bnd_63 | del_1309 |
| 33T 5 | 76736809 | 76736809 | WDR41 | exonic | 5q13.3 | . | bnd_66 | del_1090 |
| 33T 5 | 76736825 | 76736825 | WDR41 | splicing | 5q13.3 | . | bnd_254 | transl_inter_1246 |
| 33T 5 | 77187234 | 77187234 | LOC101929154 | ncRNA_intronic | 5q14.1 | Score=2617;Name="3702523:AluY(SINE)" | bnd_173 | transl_intra_1831_0 |
| 33T 5 | 77314583 | 77314583 | AP3B1 | intronic | 5q14.1 | . | bnd_175 | transl_intra_1510_0 |
| 33T 5 | 87560150 | 87560150 | TMEM161B | intronic | 5q14.3 | Score=7062;Name="3720323:L1PA8(LINE)" | bnd_68 | del_762 |
| 33T 5 | 90209801 | 90209801 | GPR98 | intronic | 5q14.3 | . | bnd_178 | transl_intra_1340_0 |
| 33T 5 | 90210165 | 90210165 | GPR98 | intronic | 5q14.3 | Score=523;Name="3724186:MIR(SINE)" | bnd_67 | del_762 |
| 33T 5 | 90948807 | 90948807 | . | intergenic | 5q14.3 | Score=3866;Name="3725606:L1PB4(LINE)" | bnd_256 | transl_inter_3108 |
| 33T 5 | 91037773 | 91037773 | . | intergenic | 5q14.3 | . | bnd_65 | del_1090 |
| 33T 5 | 91038065 | 91038065 | . | intergenic | 5q14.3 | . | bnd_177 | transl_intra_1340_0 |
| 33T 6 | 128599260 | 128599260 | PTPRK | intronic | 6q22.33 | Score=1947;Name="4103535:AluSx(SINE)" | bnd_70 | del_1061 |
| 33T 6 | 128600898 | 128600898 | PTPRK | intronic | 6q22.33 | . | bnd_69 | del_1061 |
| 33T 7 | 11472639 | 11472639 | THSD7A | intronic | 7p21.3 | . | bnd_72 | del_1552 |
| 33T 7 | 11567928 | 11567928 | THSD7A | intronic | 7p21.3 | Score=274;Name="4247304:MLT1K(LTR)" | bnd_71 | del_1552 |

| | | | | | | | | |
|---|---|---|---|---|---|---|---|---|
| 33T 7 | 22381517 | 22381517 | RAPGEF5 | intronic | 7p15.3 | Score=11480;Name="4264229:THE1B-int(LTR)" | bnd_74 | del_1906 |
| 33T 7 | 22409734 | 22409734 | . | intergenic | 7p15.3 | Score=2337;Name="4264278:AluSx1(SINE)" | bnd_73 | del_1906 |
| 33T 7 | 70242013 | 70242013 | AUTS2 | intronic | 7q11.22 | . | bnd_246 | tandem_dup_1224 |
| 33T 7 | 70367516 | 70367516 | . | intergenic | 7q11.22 | Score=1960;Name="4345524:AluJo(SINE)" | bnd_245 | tandem_dup_1224 |
| 33T 9 | 103993937 | 103993937 | LPPR1 | intronic | 9q31.1 | . | bnd_180 | transl_intra_710_0 |
| 33T 9 | 104089183 | 104089183 | . | intergenic | 9q31.1 | . | bnd_206 | transl_intra_1034_0 |
| 33T 9 | 104089264 | 104089264 | . | intergenic | 9q31.1 | . | bnd_253 | transl_inter_1246 |
| 33T 9 | 104117011 | 104117011 | . | intergenic | 9q31.1 | Score=6219;Name="4907141:MLT1A0-int(LTR)" | bnd_251 | transl_inter_958 |
| 33T 9 | 104117045 | 104117045 | . | intergenic | 9q31.1 | Score=6219;Name="4907141:MLT1A0-int(LTR)" | bnd_255 | transl_inter_3108 |
| 33T 9 | 121132467 | 121132467 | . | intergenic | 9q33.1 | Score=627;Name="4941567:MER5A1(DNA)" | bnd_179 | transl_intra_710_0 |
| 33T 9 | 121132578 | 121132578 | . | intergenic | 9q33.1 | Score=627;Name="4941567:MER5A1(DNA)" | bnd_205 | transl_intra_1034_0 |
| 33T 9 | 130688178 | 130688178 | PIP5KL1 | exonic | 9q34.11 | . | bnd_248 | tandem_dup_1686 |
| 33T 9 | 130819538 | 130819538 | . | intergenic | 9q34.11 | Score=1072;Name="4962975:MER76(LTR)" | bnd_247 | tandem_dup_1686 |
| 33T 11 | 103060146 | 103060146 | DYNC2H1 | intronic | 11q22.3 | . | bnd_6 | del_748 |
| 33T 11 | 103063324 | 103063324 | DYNC2H1 | intronic | 11q22.3 | . | bnd_5 | del_748 |
| 33T 11 | 132512320 | 132512320 | OPCML | intronic | 11q25 | . | bnd_212 | tandem_dup_1679 |
| 33T 11 | 132512736 | 132512736 | OPCML | intronic | 11q25 | Score=376;Name="906513:MIRc(SINE)" | bnd_211 | tandem_dup_1679 |
| 33T 12 | 98824558 | 98824558 | . | intergenic | 12q23.1 | Score=442;Name="1081100:L1M5(LINE)" | bnd_214 | tandem_dup_719 |
| 33T 12 | 98919836 | 98919836 | TMPO | intronic | 12q23.1 | . | bnd_213 | tandem_dup_719 |
| 33T 16 | 154784 | 154784 | NPRL3 | intronic | 16p13.3 | . | bnd_8 | del_2283_0 |
| 33T 16 | 157494 | 157494 | NPRL3 | intronic | 16p13.3 | Score=1702;Name="1634712:AluSx4(SINE)" | bnd_216 | tandem_dup_2878_0 |
| 33T 16 | 174987 | 174987 | NPRL3 | UTR5 | 16p13.3 | Score=2246;Name="1634740:AluSx1(SINE)" | bnd_10 | del_864 |
| 33T 16 | 229890 | 229890 | . | upstream | 16p13.3 | Score=225;Name="1634869:MIRb(SINE)" | bnd_9 | del_864 |
| 33T 16 | 402957 | 402957 | . | upstream | 16p13.3 | . | bnd_12 | del_1216 |
| 33T 16 | 486533 | 486533 | RAB11FIP3 | intronic | 16p13.3 | Score=1650;Name="1635402:AluSx1(SINE)" | bnd_182 | transl_intra_1595 |
| 33T 16 | 638404 | 638404 | . | upstream | 16p13.3 | Score=1456;Name="1635633:AluJb(SINE)" | bnd_110 | del_inssd_1525_0/754_0 |
| 33T 16 | 728026 | 728026 | RHBDL1 | exonic | 16p13.3 | . | bnd_114 | del_inssd_822_0/1322_0 |
| 33T 16 | 728658 | 728658 | . | downstream | 16p13.3 | . | bnd_11 | del_1216 |
| 33T 16 | 1163181 | 1163181 | . | intergenic | 16p13.3 | . | bnd_218 | tandem_dup_1955_0 |
| 33T 16 | 1242537 | 1242537 | CACNA1H | intronic | 16p13.3 | . | bnd_146 | transl_intra_1318_0 |
| 33T 16 | 1257931 | 1257931 | CACNA1H | intronic | 16p13.3 | . | bnd_138 | del_675_0 |
| 33T 16 | 1264131 | 1264131 | CACNA1H | intronic | 16p13.3 | . | bnd_184 | transl_intra_1800 |
| 33T 16 | 1670798 | 1670798 | CRAMP1L | intronic | 16p13.3 | . | bnd_112 | del_inssd_822_0/1322_0 |
| 33T 16 | 3163990 | 3163990 | ZNF205-AS1,ZNF205 | ncRNA_intronic | 16p13.3 | Score=329;Name="1639669:MIRc(SINE)" | bnd_220 | tandem_dup_3760_0 |
| 33T 16 | 3164052 | 3164052 | ZNF205,ZNF205-AS1 | ncRNA_intronic | 16p13.3 | Score=329;Name="1639669:MIRc(SINE)" | bnd_222 | tandem_dup_1815_0 |
| 33T 16 | 3198312 | 3198312 | . | intergenic | 16p13.3 | . | bnd_224 | tandem_dup_2353_0 |
| 33T 16 | 3299790 | 3299790 | MEFV | intronic | 16p13.3 | . | bnd_226 | tandem_dup_2090_0 |
| 33T 16 | 3326423 | 3326423 | . | intergenic | 16p13.3 | Score=2803;Name="1640070:LTR45C(LTR)" | bnd_186 | transl_intra_791 |

| | | | | | | | | |
|---|---|---|---|---|---|---|---|---|
| 33T 16 | 3540751 | 3540751 | . | intergenic | 16p13.3 | Score=1747;Name="1640505:AluJr(SINE)" | bnd_16 | del_2009 |
| 33T 16 | 3559001 | 3559001 | CLUAP1 | intronic | 16p13.3 | Score=1637;Name="1640546:AluJr(SINE)" | bnd_150 | transl_intra_1104_0 |
| 33T 16 | 3814718 | 3814718 | CREBBP | intronic | 16p13.3 | Score=1975;Name="1641090:AluJr(SINE)" | bnd_152 | transl_intra_1023_0 |
| 33T 16 | 3820447 | 3820447 | CREBBP | intronic | 16p13.3 | Score=1682;Name="1641101:AluJb(SINE)" | bnd_91 | del_insou_1433_0/897_0 |
| 33T 16 | 3857036 | 3857036 | CREBBP | intronic | 16p13.3 | . | bnd_18 | del_1715 |
| 33T 16 | 3858010 | 3858010 | CREBBP | intronic | 16p13.3 | . | bnd_188 | transl_intra_2449_0 |
| 33T 16 | 3922425 | 3922425 | CREBBP | intronic | 16p13.3 | . | bnd_89 | del_insou_1433_0/897_0 |
| 33T 16 | 4049760 | 4049760 | ADCY9 | intronic | 16p13.3 | . | bnd_190 | transl_intra_1417 |
| 33T 16 | 4060190 | 4060190 | ADCY9 | intronic | 16p13.3 | . | bnd_189 | transl_intra_1417 |
| 33T 16 | 4149828 | 4149828 | ADCY9 | intronic | 16p13.3 | Score=1582;Name="1641842:AluJb(SINE)" | bnd_78 | del_ins_1798 |
| 33T 16 | 4168287 | 4168287 | . | intergenic | 16p13.3 | Score=1418;Name="1641888:L1MC4a(LINE)" | bnd_20 | del_1785_0 |
| 33T 16 | 4197666 | 4197666 | . | intergenic | 16p13.3 | . | bnd_7 | del_2283_0 |
| 33T 16 | 4374074 | 4374074 | . | intergenic | 16p13.3 | . | bnd_145 | transl_intra_1318_0 |
| 33T 16 | 4387624 | 4387624 | GLIS2 | UTR3 | 16p13.3 | . | bnd_192 | transl_intra_2770 |
| 33T 16 | 4387911 | 4387911 | GLIS2 | UTR3 | 16p13.3 | . | bnd_191 | transl_intra_2770 |
| 33T 16 | 4899666 | 4899666 | UBN1 | intronic | 16p13.3 | Score=1563;Name="1643636:AluJb(SINE)" | bnd_194 | transl_intra_3720 |
| 33T 16 | 4899808 | 4899808 | UBN1 | intronic | 16p13.3 | Score=1563;Name="1643636:AluJb(SINE)" | bnd_193 | transl_intra_3720 |
| 33T 16 | 4928402 | 4928402 | UBN1 | intronic | 16p13.3 | . | bnd_196 | transl_intra_969 |
| 33T 16 | 4937518 | 4937518 | PPL | intronic | 16p13.3 | Score=284;Name="1643682:MIRc(SINE)" | bnd_127 | del_inssu_2608_0/2057_0 |
| 33T 16 | 5349172 | 5349172 | . | intergenic | 16p13.3 | Score=2602;Name="1644732:L1ME2(LINE)" | bnd_158 | transl_intra_2281_0 |
| 33T 16 | 5910442 | 5910442 | . | intergenic | 16p13.3 | . | bnd_129 | del_inssu_2608_0/2057_0 |
| 33T 16 | 5919213 | 5919213 | . | intergenic | 16p13.3 | . | bnd_119 | del_inssu_1027_0/1696_0 |
| 33T 16 | 6147850 | 6147850 | RBFOX1 | intronic | 16p13.3 | . | bnd_121 | del_inssu_1027_0/1696_0 |
| 33T 16 | 6151701 | 6151701 | RBFOX1 | intronic | 16p13.3 | Score=945;Name="1646640:AluSx(SINE)" | bnd_195 | transl_intra_969 |
| 33T 16 | 6247305 | 6247305 | RBFOX1 | intronic | 16p13.3 | . | bnd_22 | del_746 |
| 33T 16 | 6558142 | 6558142 | RBFOX1 | intronic | 16p13.3 | Score=3708;Name="1647451:L1PA13(LINE)" | bnd_77 | del_ins_1798 |
| 33T 16 | 6692206 | 6692206 | RBFOX1 | intronic | 16p13.3 | . | bnd_120 | del_inssu_1027_0/1696_0 |
| 33T 16 | 6908386 | 6908386 | RBFOX1 | intronic | 16p13.3 | . | bnd_122 | del_inssu_1027_0/1696_0 |
| 33T 16 | 7308887 | 7308887 | RBFOX1 | intronic | 16p13.3 | . | bnd_141 | del_inssu_1570_0/4945_0 |
| 33T 16 | 7383158 | 7383158 | RBFOX1 | intronic | 16p13.3 | . | bnd_143 | del_inssu_1570_0/4945_0 |
| 33T 16 | 7576284 | 7576284 | RBFOX1 | intronic | 16p13.3 | . | bnd_151 | transl_intra_1023_0 |
| 33T 16 | 7577052 | 7577052 | RBFOX1 | intronic | 16p13.3 | . | bnd_187 | transl_intra_2449_0 |
| 33T 16 | 7751452 | 7751452 | RBFOX1 | intronic | 16p13.3 | Score=1556;Name="1649850:MER1B(DNA)" | bnd_80 | del_ins_1253 |
| 33T 16 | 7827992 | 7827992 | . | intergenic | 16p13.3 | . | bnd_118 | del_inssd_2854_0/2534_0 |
| 33T 16 | 7882453 | 7882453 | . | intergenic | 16p13.3 | . | bnd_116 | del_inssd_2854_0/2534_0 |
| 33T 16 | 7977493 | 7977493 | . | intergenic | 16p13.2 | . | bnd_228 | tandem_dup_1629_0 |
| 33T 16 | 8331627 | 8331627 | . | intergenic | 16p13.2 | Score=3455;Name="1651105:MER4A1(LTR)" | bnd_219 | tandem_dup_3760_0 |
| 33T 16 | 8387598 | 8387598 | . | intergenic | 16p13.2 | . | bnd_123 | del_inssu_1784_0/1506_0 |
| 33T 16 | 8709372 | 8709372 | . | intergenic | 16p13.2 | Score=1145;Name="1651930:AluJo(SINE)" | bnd_136 | del_1123_0 |
| 33T 16 | 8721931 | 8721931 | METTL22 | intronic | 16p13.2 | Score=986;Name="1651972:MER58A(DNA)" | bnd_109 | del_inssd_1525_0/754_0 |
| 33T 16 | 8919475 | 8919475 | PMM2 | intronic | 16p13.2 | Score=2114;Name="1652524:AluSq2(SINE)" | bnd_24 | del_1648 |

| | | | | | | | | |
|---|---|---|---|---|---|---|---|---|
| 33T 16 | 8920708 | 8920708 | PMM2 | intronic | 16p13.2 | Score=13826;Name="1652527:HERV35I-int(LTR)" | bnd_23 | del_1648 |
| 33T 16 | 8934108 | 8934108 | PMM2 | intronic | 16p13.2 | Score=25108;Name="1652549:HERVH-int(LTR)" | bnd_160 | transl_intra_1362_0 |
| 33T 16 | 8950363 | 8950363 | CARHSP1 | intronic | 16p13.2 | . | bnd_79 | del_ins_1253 |
| 33T 16 | 9417793 | 9417793 | . | intergenic | 16p13.2 | . | bnd_159 | transl_intra_1362_0 |
| 33T 16 | 9486028 | 9486028 | . | intergenic | 16p13.2 | Score=260;Name="1654048:MLT1K(LTR)" | bnd_217 | tandem_dup_1955_0 |
| 33T 16 | 10515613 | 10515613 | ATF7IP2 | intronic | 16p13.13 | Score=26057;Name="1656585:HERVK9-int(LTR)" | bnd_107 | del_inssd_1525_0/754_0 |
| 33T 16 | 11043272 | 11043272 | CLEC16A | intronic | 16p13.13 | Score=373;Name="1657963:L2b(LINE)" | bnd_26 | del_1805 |
| 33T 16 | 11044551 | 11044551 | CLEC16A | intronic | 16p13.13 | . | bnd_117 | del_inssd_2854_0/2534_0 |
| 33T 16 | 11718667 | 11718667 | . | intergenic | 16p13.13 | Score=910;Name="1659691:MLT1H(LTR)" | bnd_149 | transl_intra_1104_0 |
| 33T 16 | 11719960 | 11719960 | . | intergenic | 16p13.13 | . | bnd_17 | del_1715 |
| 33T 16 | 11731432 | 11731432 | . | intergenic | 16p13.13 | Score=217;Name="1659733:MIRb(SINE)" | bnd_15 | del_2009 |
| 33T 16 | 11981099 | 11981099 | GSPT1 | intronic | 16p13.13 | . | bnd_28 | del_1902 |
| 33T 16 | 11981376 | 11981376 | GSPT1 | intronic | 16p13.13 | . | bnd_230 | tandem_dup_1052_0 |
| 33T 16 | 12440309 | 12440309 | SNX29 | intronic | 16p13.13 | Score=581;Name="1661459:MIR(SINE)" | bnd_162 | transl_intra_3882_0 |
| 33T 16 | 12451128 | 12451128 | SNX29 | intronic | 16p13.13 | Score=1331;Name="1661483:L1MC5(LINE)" | bnd_27 | del_1902 |
| 33T 16 | 12456393 | 12456393 | SNX29 | intronic | 16p13.13 | . | bnd_115 | del_inssd_2854_0/2534_0 |
| 33T 16 | 12466783 | 12466783 | SNX29 | intronic | 16p13.13 | Score=363;Name="1661519:MIR(SINE)" | bnd_198 | transl_intra_540 |
| 33T 16 | 12715307 | 12715307 | . | intergenic | 16p13.12 | . | bnd_164 | transl_intra_669_0 |
| 33T 16 | 12718588 | 12718588 | . | intergenic | 16p13.12 | Score=1484;Name="1662072:AluJb(SINE)" | bnd_183 | transl_intra_1800 |
| 33T 16 | 13082197 | 13082197 | SHISA9 | intronic | 16p13.12 | . | bnd_125 | del_inssu_1784_0/1506_0 |
| 33T 16 | 13115076 | 13115076 | SHISA9 | intronic | 16p13.12 | Score=789;Name="1663164:MER20(DNA)" | bnd_113 | del_inssd_822_0/1322_0 |
| 33T 16 | 13161821 | 13161821 | SHISA9 | intronic | 16p13.12 | . | bnd_82 | del_ins_1562 |
| 33T 16 | 13557971 | 13557971 | . | intergenic | 16p13.12 | Score=343;Name="1664220:MIRb(SINE)" | bnd_140 | del_1321_0 |
| 33T 16 | 13563686 | 13563686 | . | intergenic | 16p13.12 | . | bnd_81 | del_ins_1562 |
| 33T 16 | 13624225 | 13624225 | . | intergenic | 16p13.12 | Score=694;Name="1664383:MIR(SINE)" | bnd_181 | transl_intra_1595 |
| 33T 16 | 13963447 | 13963447 | . | intergenic | 16p13.12 | Score=5725;Name="1665250:L1MC1(LINE)" | bnd_90 | del_insou_1433_0/897_0 |
| 33T 16 | 13990314 | 13990314 | . | intergenic | 16p13.12 | . | bnd_30 | del_1575_0 |
| 33T 16 | 14080491 | 14080491 | . | intergenic | 16p13.12 | Score=2800;Name="1665506:L1ME1(LINE)" | bnd_166 | transl_intra_1341_0 |
| 33T 16 | 14288265 | 14288265 | MKL2 | intronic | 16p13.12 | Score=1001;Name="1665971:MLT1A(LTR)" | bnd_92 | del_insou_1433_0/897_0 |
| 33T 16 | 14346867 | 14346867 | MKL2 | intronic | 16p13.12 | . | bnd_32 | del_1009_0 |
| 33T 16 | 15523582 | 15523582 | . | intergenic | 16p13.11 | Score=1773;Name="1669035:AluJb(SINE)" | bnd_229 | tandem_dup_1052_0 |
| 33T 16 | 15525417 | 15525417 | . | intergenic | 16p13.11 | Score=239;Name="1669042:(TA)n(Simple_repeat)" | bnd_25 | del_1805 |
| 33T 16 | 15607476 | 15607476 | C16orf45 | intronic | 16p13.11 | Score=2202;Name="1669287:AluSg(SINE)" | bnd_157 | transl_intra_2281_0 |
| 33T 16 | 15910524 | 15910524 | MYH11 | intronic | 16p13.11 | Score=4010;Name="1670094:HAL1(LINE)" | bnd_225 | tandem_dup_2090_0 |

| | | | | | | | | | |
|---|---|---|---|---|---|---|---|---|---|
| 33T | 16 | 16945014 | 16945014 | . | intergenic | 16p12.3 | . | bnd_124 | del_inssu_1784_0/1506_0 |
| 33T | 16 | 17149670 | 17149670 | . | intergenic | 16p12.3 | Score=3181;Name="1673465:L1MA9(LINE)" | bnd_215 | tandem_dup_2878_0 |
| 33T | 16 | 17151725 | 17151725 | . | intergenic | 16p12.3 | Score=1383;Name="1673468:AluJb(SINE)" | bnd_126 | del_inssu_1784_0/1506_0 |
| 33T | 16 | 17315191 | 17315191 | XYLT1 | intronic | 16p12.3 | Score=599;Name="1673881:MIR(SINE)" | bnd_200 | transl_intra_1500 |
| 33T | 16 | 17427592 | 17427592 | XYLT1 | intronic | 16p12.3 | Score=2281;Name="1674182:AluSz(SINE)" | bnd_128 | del_inssu_2608_0/2057_0 |
| 33T | 16 | 17482823 | 17482823 | XYLT1 | intronic | 16p12.3 | Score=2389;Name="1674305:MER92B(LTR)" | bnd_130 | del_inssu_2608_0/2057_0 |
| 33T | 16 | 18125018 | 18125018 | . | intergenic | 16p12.3 | Score=2643;Name="1675870:LTR17(LTR)" | bnd_111 | del_inssd_822_0/1322_0 |
| 33T | 16 | 18141987 | 18141987 | . | intergenic | 16p12.3 | Score=349;Name="1675916:(TA)n(Simple_repeat)" | bnd_168 | transl_intra_1597_0 |
| 33T | 16 | 19215472 | 19215472 | SYT17 | intronic | 16p12.3 | Score=896;Name="1678810:MER20(DNA)" | bnd_29 | del_1575_0 |
| 33T | 16 | 19223092 | 19223092 | SYT17 | intronic | 16p12.3 | Score=2441;Name="1678839:AluY(SINE)" | bnd_227 | tandem_dup_1629_0 |
| 33T | 16 | 19234384 | 19234384 | SYT17 | exonic | 16p12.3 | . | bnd_232 | tandem_dup_1841_0 |
| 33T | 16 | 19490851 | 19490851 | TMC5 | splicing | 16p12.3 | . | bnd_163 | transl_intra_669_0 |
| 33T | 16 | 19519679 | 19519679 | GDE1 | intronic | 16p12.3 | . | bnd_131 | del_inssu_1538_0/2469_0 |
| 33T | 16 | 19562864 | 19562864 | CCP110 | UTR3 | 16p12.3 | . | bnd_34 | del_1542 |
| 33T | 16 | 19638256 | 19638256 | C16orf62 | intronic | 16p12.3 | . | bnd_101 | del_insou_617_0/1942_0 |
| 33T | 16 | 19833062 | 19833062 | IQCK | intronic | 16p12.3 | Score=3536;Name="1680535:L1MB5(LINE)" | bnd_36 | del_3873 |
| 33T | 16 | 19846288 | 19846288 | IQCK | intronic | 16p12.3 | Score=571;Name="1680578:L1MC4a(LINE)" | bnd_202 | transl_intra_3681_0 |
| 33T | 16 | 19865833 | 19865833 | IQCK | intronic | 16p12.3 | Score=638;Name="1680639:MER5A(DNA)" | bnd_99 | del_insou_617_0/1942_0 |
| 33T | 16 | 20167493 | 20167493 | . | intergenic | 16p12.3 | . | bnd_38 | del_1230 |
| 33T | 16 | 20179978 | 20179978 | . | intergenic | 16p12.3 | Score=1330;Name="1681524:LTR16B1(LTR)" | bnd_37 | del_1230 |
| 33T | 16 | 21060564 | 21060564 | DNAH3 | intronic | 16p12.3 | Score=1653;Name="1683565:AluJo(SINE)" | bnd_234 | tandem_dup_977_0 |
| 33T | 16 | 21117732 | 21117732 | DNAH3 | intronic | 16p12.3 | . | bnd_40 | del_2784 |
| 33T | 16 | 21118227 | 21118227 | DNAH3 | intronic | 16p12.3 | Score=668;Name="1683728:MIR(SINE)" | bnd_39 | del_2784 |
| 33T | 16 | 21603871 | 21603871 | . | intergenic | 16p12.2 | Score=2079;Name="1684938:AluSx1(SINE)" | bnd_148 | transl_intra_1780_0 |
| 33T | 16 | 22024564 | 22024564 | C16orf52 | intronic | 16p12.2 | . | bnd_133 | del_inssu_1538_0/2469_0 |
| 33T | 16 | 22054241 | 22054241 | C16orf52 | intronic | 16p12.2 | Score=361;Name="1685994:MIRb(SINE)" | bnd_19 | del_1785_0 |
| 33T | 16 | 22074158 | 22074158 | C16orf52 | intronic | 16p12.2 | Score=765;Name="1686044:MIR(SINE)" | bnd_35 | del_3873 |
| 33T | 16 | 22690953 | 22690953 | . | intergenic | 16p12.2 | Score=347;Name="1687616:MIRc(SINE)" | bnd_233 | tandem_dup_977_0 |
| 33T | 16 | 22782832 | 22782832 | MIR548D2,MIR548AA2 | ncRNA_intronic | 16p12.2 | Score=598;Name="1687845:MLT1I(LTR)" | bnd_223 | tandem_dup_2353_0 |
| 33T | 16 | 22811065 | 22811065 | . | intergenic | 16p12.2 | . | bnd_236 | tandem_dup_4995_0 |
| 33T | 16 | 22818573 | 22818573 | . | intergenic | 16p12.2 | Score=4529;Name="1687931:L2(LINE)" | bnd_132 | del_inssu_1538_0/2469_0 |
| 33T | 16 | 22821334 | 22821334 | . | intergenic | 16p12.2 | . | bnd_238 | tandem_dup_1863_0 |
| 33T | 16 | 22957399 | 22957399 | . | intergenic | 16p12.2 | . | bnd_134 | del_inssu_1538_0/2469_0 |

| | | | | | | | | | |
|---|---|---|---|---|---|---|---|---|---|
| 33T | 16 | 23266747 | 23266747 | . | intergenic | 16p12.2 | Score=2106;Name="1688994:AluSz(SINE)" | bnd_147 | transl_intra_1780_0 |
| 33T | 16 | 23274612 | 23274612 | . | intergenic | 16p12.2 | . | bnd_94 | transl_intra_1256_0 |
| 33T | 16 | 23803506 | 23803506 | . | intergenic | 16p12.2 | Score=5264;Name="1690575:MER21-int(LTR)" | bnd_237 | tandem_dup_1863_0 |
| 33T | 16 | 23804258 | 23804258 | . | intergenic | 16p12.2 | Score=1872;Name="1690576:AluJr(SINE)" | bnd_221 | tandem_dup_1815_0 |
| 33T | 16 | 23999731 | 23999731 | PRKCB | intronic | 16p12.2 | . | bnd_31 | del_1009_0 |
| 33T | 16 | 24145670 | 24145670 | PRKCB | intronic | 16p12.2 | Score=1203;Name="1691485:AluJb(SINE)" | bnd_93 | transl_intra_1256_0 |
| 33T | 16 | 24148766 | 24148766 | PRKCB | intronic | 16p12.2 | Score=1898;Name="1691491:AluJo(SINE)" | bnd_84 | del_ins_731 |
| 33T | 16 | 24418275 | 24418275 | . | intergenic | 16p12.1 | . | bnd_42 | del_1421 |
| 33T | 16 | 24825669 | 24825669 | TNRC6A | intronic | 16p12.1 | . | bnd_201 | transl_intra_3681_0 |
| 33T | 16 | 24866199 | 24866199 | SLC5A11 | intronic | 16p12.1 | . | bnd_44 | del_1665_0 |
| 33T | 16 | 24876554 | 24876554 | SLC5A11 | intronic | 16p12.1 | Score=6640;Name="1693578:L1M4(LINE)" | bnd_83 | del_ins_731 |
| 33T | 16 | 24887285 | 24887285 | SLC5A11 | intronic | 16p12.1 | Score=2281;Name="1693610:AluSx1(SINE)" | bnd_170 | transl_intra_2723_0 |
| 33T | 16 | 24938347 | 24938347 | ARHGAP17 | intronic | 16p12.1 | Score=1891;Name="1693758:AluSx1(SINE)" | bnd_139 | del_1321_0 |
| 33T | 16 | 24967928 | 24967928 | ARHGAP17 | intronic | 16p12.1 | Score=645;Name="1693804:MER102a(DNA)" | bnd_169 | transl_intra_2723_0 |
| 33T | 16 | 24968530 | 24968530 | ARHGAP17 | intronic | 16p12.1 | . | bnd_135 | del_1123_0 |
| 33T | 16 | 25166500 | 25166500 | LCMT1 | intronic | 16p12.1 | Score=726;Name="1694301:MER104(DNA)" | bnd_104 | transl_intra_1422_0 |
| 33T | 16 | 25170732 | 25170732 | LCMT1 | intronic | 16p12.1 | Score=1790;Name="1694314:AluJr(SINE)" | bnd_197 | transl_intra_540 |
| 33T | 16 | 25312251 | 25312251 | . | intergenic | 16p12.1 | Score=2237;Name="1694752:AluSx1(SINE)" | bnd_46 | del_2286 |
| 33T | 16 | 25382115 | 25382115 | . | intergenic | 16p12.1 | Score=2273;Name="1694951:MER87(LTR)" | bnd_45 | del_2286 |
| 33T | 16 | 25768017 | 25768017 | HS3ST4 | intronic | 16p12.1 | . | bnd_142 | del_inssu_1570_0/4945_0 |
| 33T | 16 | 25844517 | 25844517 | HS3ST4 | intronic | 16p12.1 | . | bnd_33 | del_1542 |
| 33T | 16 | 26308403 | 26308403 | . | intergenic | 16p12.1 | . | bnd_41 | del_1421 |
| 33T | 16 | 26626682 | 26626682 | . | intergenic | 16p12.1 | Score=1905;Name="1697828:MLT2B1(LTR)" | bnd_165 | transl_intra_1341_0 |
| 33T | 16 | 26981769 | 26981769 | . | intergenic | 16p12.1 | Score=1386;Name="1698725:L2a(LINE)" | bnd_48 | del_4996 |
| 33T | 16 | 27148075 | 27148075 | . | intergenic | 16p12.1 | Score=2217;Name="1699182:THE1D(LTR)" | bnd_167 | transl_intra_1597_0 |
| 33T | 16 | 27232474 | 27232474 | KDM8 | UTR3 | 16p12.1 | Score=188;Name="1699424:MIR(SINE)" | bnd_97 | del_insou_1583_0/3228_0 |
| 33T | 16 | 27256331 | 27256331 | NSMCE1 | intronic | 16p12.1 | . | bnd_13 | del_1306 |
| 33T | 16 | 27260241 | 27260241 | NSMCE1 | intronic | 16p12.1 | Score=1953;Name="1699466:AluJb(SINE)" | bnd_155 | transl_intra_1565_0 |
| 33T | 16 | 27449811 | 27449811 | IL21R | intronic | 16p12.1 | Score=837;Name="1700024:FLAM_C(SINE)" | bnd_172 | transl_intra_2001_0 |
| 33T | 16 | 27547603 | 27547603 | GTF3C1 | intronic | 16p12.1 | Score=2877;Name="1700212:Tigger2b_Pri(DNA)" | bnd_137 | del_675_0 |
| 33T | 16 | 27590308 | 27590308 | KIAA0556 | intronic | 16p12.1 | . | bnd_50 | del_2293 |
| 33T | 16 | 27594436 | 27594436 | KIAA0556 | intronic | 16p12.1 | . | bnd_49 | del_2293 |
| 33T | 16 | 27678429 | 27678429 | KIAA0556 | intronic | 16p12.1 | Score=1987;Name="1700599:AluJo(SINE)" | bnd_95 | del_insou_1583_0/3228_0 |
| 33T | 16 | 27793762 | 27793762 | . | intergenic | 16p12.1 | . | bnd_47 | del_4996 |

| | | | | | | | | |
|---|---|---|---|---|---|---|---|---|
| 33T 16 | 27804224 | 27804224 | GSG1L | intronic | 16p12.1 | Score=307;Name="1700899:MIRc(SINE)" | bnd_96 | del_insou_1583_0/3228_0 |
| 33T 16 | 27880307 | 27880307 | GSG1L | intronic | 16p12.1 | . | bnd_204 | transl_intra_1463 |
| 33T 16 | 27880616 | 27880616 | GSG1L | intronic | 16p12.1 | . | bnd_203 | transl_intra_1463 |
| 33T 16 | 28578191 | 28578191 | CCDC101 | intronic | 16p11.2 | Score=1297;Name="1703109:AluSx(SINE)" | bnd_171 | transl_intra_2001_0 |
| 33T 16 | 28833885 | 28833885 | . | upstream | 16p11.2 | . | bnd_43 | del_1665_0 |
| 33T 16 | 28862225 | 28862225 | . | intergenic | 16p11.2 | Score=2344;Name="1703907:AluSc(SINE)" | bnd_161 | transl_intra_3882_0 |
| 33T 16 | 29766435 | 29766435 | . | intergenic | 16p11.2 | Score=1251;Name="1706059:MER65-int(LTR)" | bnd_231 | tandem_dup_1841_0 |
| 33T 16 | 29854093 | 29854093 | MVP | intronic | 16p11.2 | . | bnd_98 | del_insou_1583_0/3228_0 |
| 33T 16 | 30626771 | 30626771 | . | intergenic | 16p11.2 | . | bnd_21 | del_746 |
| 33T 16 | 30921211 | 30921211 | . | intergenic | 16p11.2 | Score=687;Name="1709145:L2a(LINE)" | bnd_185 | transl_intra_791 |
| 33T 16 | 30956331 | 30956331 | FBXL19 | intronic | 16p11.2 | Score=303;Name="1709243:Charlie8(DNA)" | bnd_100 | del_insou_617_0/1942_0 |
| 33T 16 | 31086632 | 31086632 | ZNF646 | intronic | 16p11.2 | Score=2352;Name="1709592:AluSx1(SINE)" | bnd_144 | del_inssu_1570_0/4945_0 |
| 33T 16 | 31086988 | 31086988 | ZNF646 | intronic | 16p11.2 | . | bnd_235 | tandem_dup_4995_0 |
| 33T 16 | 31481916 | 31481916 | . | intergenic | 16p11.2 | Score=2060;Name="1710542:AluSx1(SINE)" | bnd_102 | del_insou_617_0/1942_0 |
| 33T 16 | 32509902 | 32509902 | . | intergenic | 16p11.2 | Score=1219;Name="1712251:ALR/Alpha(Satellite)" | bnd_103 | transl_intra_1422_0 |
| 33T 16 | 4935173 | 4935173 | PPL | exonic | 16p13.3 | . | bnd_156 | transl_intra_1565_0 |
| 33T 16 | 30042351 | 30042351 | . | upstream | 16p11.2 | . | bnd_199 | transl_intra_1500 |
| 33T 16 | 683909 | 683909 | WFIKKN1 | exonic | 16p13.3 | . | bnd_108 | del_inssd_1525_0/754_0 |
| 33T 16 | 1047902 | 1047902 | . | intergenic | 16p13.3 | . | bnd_14 | del_1306 |
| 33T 16 | 4401087 | 4401087 | PAM16,CORO7-PAM16 | intronic | 16p13.3 | . | bnd_154 | transl_intra_3290_0 |
| 33T 16 | 4401221 | 4401221 | PAM16,CORO7-PAM16 | intronic | 16p13.3 | . | bnd_153 | transl_intra_3290_0 |
| 33T 18 | 2562388 | 2562388 | METTL4 | intronic | 18p11.32 | Score=3050;Name="1976808:L1MB4(LINE)" | bnd_240 | tandem_dup_1569 |
| 33T 18 | 2666957 | 2666957 | SMCHD1 | exonic | 18p11.32 | . | bnd_239 | tandem_dup_1569 |
| 33T 20 | 41158174 | 41158174 | PTPRT | intronic | 20q12 | Score=2119;Name="2724718:AluSx(SINE)" | bnd_249 | transl_inter_6604 |
| 33T 21 | 34553686 | 34553686 | . | intergenic | 21q22.11 | Score=2289;Name="2809378:L1PB1(LINE)" | bnd_244 | tandem_dup_517 |
| 33T 21 | 34592058 | 34592058 | . | intergenic | 21q22.11 | Score=2412;Name="2809462:AluSx1(SINE)" | bnd_243 | tandem_dup_517 |
| 33T X | 20172899 | 20172899 | RPS6KA3 | UTR3 | Xp22.12 | . | bnd_76 | del_577 |
| 33T X | 20214811 | 20214811 | RPS6KA3 | intronic | Xp22.12 | . | bnd_75 | del_577 |
| 33T X | 111094848 | 111094848 | TRPC5 | intronic | Xq23 | . | bnd_88 | del_ins_755 |
| 33T X | 111488835 | 111488835 | ZCCHC16 | intronic | Xq23 | Score=22627;Name="5178234:L1PA6(LINE)" | bnd_87 | del_ins_755 |
| 34T 1 | 51513596 | 51513596 | . | intergenic | 1p32.3 | Score=2524;Name="113895:L1PA16(LINE)" | bnd_12 | transl_inter_4143 |
| 34T 1 | 198013048 | 198013048 | . | intergenic | 1q31.3 | Score=807;Name="339504:L2(LINE)" | bnd_14 | transl_inter_6471 |
| 34T 2 | 201537828 | 201537828 | . | intergenic | 2q33.1 | Score=997;Name="2574136:L2a(LINE)" | bnd_2 | del_3864 |
| 34T 2 | 201539889 | 201539889 | . | intergenic | 2q33.1 | Score=2474;Name="2574138:ERV3-16A3_I-int(LTR)" | bnd_1 | del_3864 |
| 34T 2 | 210282023 | 210282023 | . | intergenic | 2q34 | Score=6646;Name="2590744:L1MC2(LINE)" | bnd_29 | transl_inter_6174 |
| 34T 3 | 119290455 | 119290455 | . | intergenic | 3q13.33 | . | bnd_10 | tandem_dup_1943 |
| 34T 3 | 119543431 | 119543431 | GSK3B | UTR3 | 3q13.33 | . | bnd_9 | tandem_dup_1943 |

| | | | | | | | | |
|---|---|---|---|---|---|---|---|---|
| 34T 3 | 182572682 | 182572682 | ATP11B | intronic | 3q26.33 | Score=3576;Name="3225175:L1MD(LINE)" | bnd_25 | transl_inter_6511_0 |
| 34T 5 | 13554826 | 13554826 | . | intergenic | 5p15.2 | Score=883;Name="3598996:AluJo(SINE)" | bnd_4 | del_1743 |
| 34T 5 | 13555833 | 13555833 | . | intergenic | 5p15.2 | . | bnd_3 | del_1743 |
| 34T 5 | 121738612 | 121738612 | SNCAIP | intronic | 5q23.2 | . | bnd_6 | del_2234 |
| 34T 5 | 121739612 | 121739612 | SNCAIP | intronic | 5q23.2 | . | bnd_5 | del_2234 |
| 34T 6 | 96068921 | 96068921 | . | intergenic | 6q16.1 | Score=2287;Name="4049596:THE1B(LTR)" | bnd_23 | transl_inter_6462 |
| 34T 7 | 38024313 | 38024313 | . | intergenic | 7p14.1 | . | bnd_27 | transl_inter_4098 |
| 34T 7 | 114820220 | 114820220 | . | intergenic | 7q31.2 | . | bnd_31 | transl_inter_6799 |
| 34T 10 | 55365189 | 55365189 | . | intergenic | 10q21.1 | Score=1098;Name="525143:AluJr(SINE)" | bnd_16 | transl_inter_6381 |
| 34T 10 | 78542735 | 78542735 | . | intergenic | 10q22.3 | Score=5712;Name="568730:L1PREC2(LINE)" | bnd_18 | transl_inter_6490 |
| 34T 10 | 128504017 | 128504017 | . | intergenic | 10q26.2 | Score=2158;Name="661356:AluSq(SINE)" | bnd_20 | transl_inter_5124 |
| 34T 11 | 2452768 | 2452768 | . | intergenic | 11p15.5 | . | bnd_8 | transl_intra_5783_0 |
| 34T 11 | 5126002 | 5126002 | . | intergenic | 11p15.4 | . | bnd_7 | transl_intra_5783_0 |
| 34T 12 | 67502306 | 67502306 | . | intergenic | 12q14.3 | . | bnd_22 | transl_inter_6516 |
| 34T 12 | 87618214 | 87618214 | . | intergenic | 12q21.32 | Score=3469;Name="1059542:L1M3(LINE)" | bnd_24 | transl_inter_6462 |
| 34T 12 | 91095031 | 91095031 | . | intergenic | 12q21.33 | . | bnd_13 | transl_inter_6471 |
| 34T 12 | 119358423 | 119358423 | . | intergenic | 12q24.23 | Score=3398;Name="1128714:L1PB2(LINE)" | bnd_11 | transl_inter_4143 |
| 34T 13 | 49575085 | 49575085 | FNDC3A | intronic | 13q14.2 | Score=6998;Name="1215361:L1M3(LINE)" | bnd_26 | transl_inter_6511_0 |
| 34T 17 | 7278371 | 7278371 | . | intergenic | 17p13.1 | Score=2290;Name="1820774:AluY(SINE)" | bnd_28 | transl_inter_4098 |
| 34T 18 | 2298987 | 2298987 | . | intergenic | 18p11.32 | Score=6208;Name="1976317:L1MC1(LINE)" | bnd_30 | transl_inter_6174 |
| 34T 18 | 28447640 | 28447640 | . | intergenic | 18q12.1 | . | bnd_21 | transl_inter_6516 |
| 34T 18 | 68836592 | 68836592 | . | intergenic | 18q22.3 | Score=1721;Name="2085340:MLT1D(LTR)" | bnd_32 | transl_inter_6799 |
| 34T 19 | 42395510 | 42395510 | ARHGEF1 | intronic | 19q13.2 | Score=2028;Name="2190661:AluSx1(SINE)" | bnd_15 | transl_inter_6381 |
| 34T 20 | 32944896 | 32944896 | . | intergenic | 20q11.22 | . | bnd_19 | transl_inter_5124 |
| 34T X | 83554179 | 83554179 | . | intergenic | Xq21.1 | Score=16777;Name="5133542:L1MA1(LINE)" | bnd_17 | transl_inter_6490 |
| 35T 1 | 32486714 | 32486714 | KHDRBS1 | intronic | 1p35.1 | Score=2582;Name="71260:L1MD2(LINE)" | bnd_52 | tandem_dup_3317_0 |
| 35T 1 | 33528398 | 33528398 | . | intergenic | 1p35.1 | Score=1347;Name="74180:AluSx(SINE)" | bnd_51 | tandem_dup_3317_0 |
| 35T 1 | 36725105 | 36725105 | THRAP3 | intronic | 1p34.3 | . | bnd_54 | tandem_dup_1933 |
| 35T 1 | 36779406 | 36779406 | SH3D21 | intronic | 1p34.3 | Score=1734;Name="81606:AluJr(SINE)" | bnd_53 | tandem_dup_1933 |
| 35T 1 | 68205174 | 68205174 | GNG12 | intronic | 1p31.3 | . | bnd_56 | tandem_dup_894 |
| 35T 1 | 68230299 | 68230299 | GNG12 | intronic | 1p31.3 | . | bnd_55 | tandem_dup_894 |
| 35T 1 | 93837747 | 93837747 | . | intergenic | 1p22.1 | Score=2223;Name="194727:AluJo(SINE)" | bnd_58 | tandem_dup_3279 |
| 35T 1 | 93956375 | 93956375 | FNBP1L | intronic | 1p22.1 | . | bnd_57 | tandem_dup_3279 |
| 35T 1 | 165379014 | 165379014 | RXRG | intronic | 1q23.3 | Score=235;Name="282657:MIRb(SINE)" | bnd_60 | tandem_dup_1989 |
| 35T 1 | 165611154 | 165611154 | MGST3 | intronic | 1q24.1 | . | bnd_59 | tandem_dup_1989 |
| 35T 1 | 170548321 | 170548321 | . | intergenic | 1q24.2 | . | bnd_62 | tandem_dup_855 |
| 35T 1 | 170624920 | 170624920 | . | intergenic | 1q24.2 | . | bnd_61 | tandem_dup_855 |
| 35T 1 | 199551413 | 199551413 | . | intergenic | 1q32.1 | . | bnd_246 | transl_inter_6640 |

| | | | | | | | | |
|---|---|---|---|---|---|---|---|---|
| 35T | 1 | 210450983 | 210450983 | . | intergenic | 1q32.2 | Score=2164;Name="362249:AluSq2(SINE)" | bnd_64 | tandem_dup_1430 |
| 35T | 1 | 210522935 | 210522935 | HHAT | intronic | 1q32.2 | Score=512;Name="362409:MLT1J(LTR)" | bnd_63 | tandem_dup_1430 |
| 35T | 1 | 235452170 | 235452170 | ARID4B | intronic | 1q42.3 | . | bnd_66 | tandem_dup_2152 |
| 35T | 1 | 235553870 | 235553870 | TBCE | intronic | 1q42.3 | Score=2259;Name="407449:AluSq2(SINE)" | bnd_65 | tandem_dup_2152 |
| 35T | 2 | 10186598 | 10186598 | KLF11 | intronic | 2p25.1 | . | bnd_126 | tandem_dup_2552 |
| 35T | 2 | 10296499 | 10296499 | C2orf48 | intronic | 2p25.1 | Score=256;Name="2250035:MamRep605(Unknown)" | bnd_125 | tandem_dup_2552 |
| 35T | 2 | 84873640 | 84873640 | DNAH6 | intronic | 2p11.2 | Score=14891;Name="2384980:Tigger1(DNA)" | bnd_128 | tandem_dup_1162 |
| 35T | 2 | 84986996 | 84986996 | DNAH6 | intronic | 2p11.2 | Score=8305;Name="2385144:L1M2(LINE)" | bnd_127 | tandem_dup_1162 |
| 35T | 2 | 98441069 | 98441069 | TMEM131 | intronic | 2q11.2 | Score=32549;Name="2402500:HERV9-int(LTR)" | bnd_12 | del_1393 |
| 35T | 2 | 98442485 | 98442485 | TMEM131 | intronic | 2q11.2 | Score=32549;Name="2402500:HERV9-int(LTR)" | bnd_11 | del_1393 |
| 35T | 2 | 119051355 | 119051355 | . | intergenic | 2q14.2 | Score=4930;Name="2437571:L1M2(LINE)" | bnd_130 | tandem_dup_1244 |
| 35T | 2 | 119127570 | 119127570 | . | intergenic | 2q14.2 | Score=4267;Name="2437713:L1MC2(LINE)" | bnd_129 | tandem_dup_1244 |
| 35T | 2 | 134812112 | 134812112 | . | intergenic | 2q21.2 | Score=2022;Name="2465479:MER4D(LTR)" | bnd_132 | tandem_dup_1694 |
| 35T | 2 | 134874848 | 134874848 | . | intergenic | 2q21.2 | . | bnd_131 | tandem_dup_1694 |
| 35T | 2 | 138612703 | 138612703 | . | intergenic | 2q22.1 | . | bnd_134 | tandem_dup_2485 |
| 35T | 2 | 138783522 | 138783522 | . | intergenic | 2q22.1 | . | bnd_133 | tandem_dup_2485 |
| 35T | 2 | 177963799 | 177963799 | . | intergenic | 2q31.1 | Score=878;Name="2535974:ORSL(DNA)" | bnd_136 | tandem_dup_974 |
| 35T | 2 | 178012431 | 178012431 | . | intergenic | 2q31.2 | . | bnd_135 | tandem_dup_974 |
| 35T | 2 | 179340189 | 179340189 | MIR548N,FKBP7 | ncRNA_intronic | 2q31.2 | Score=2407;Name="2538295:AluSz(SINE)" | bnd_14 | del_2692 |
| 35T | 2 | 179495855 | 179495855 | TTN,MIR548N | exonic | 2q31.2 | . | bnd_13 | del_2692 |
| 35T | 3 | 37216211 | 37216211 | LRRFIP2 | intronic | 3p22.2 | . | bnd_16 | del_2410 |
| 35T | 3 | 81207630 | 81207630 | . | intergenic | 3p12.2 | Score=4245;Name="3057866:L1M2a(LINE)" | bnd_38 | del_invers_1097_0/1001_0 |
| 35T | 3 | 86371290 | 86371290 | . | intergenic | 3p12.1 | Score=26;Name="3066033:AT_rich(Low_complexity)" | bnd_39 | del_invers_1097_0/1001_0 |
| 35T | 3 | 86418138 | 86418138 | . | intergenic | 3p12.1 | . | bnd_37 | del_invers_1097_0/1001_0 |
| 35T | 3 | 86605426 | 86605426 | . | intergenic | 3p12.1 | Score=3611;Name="3066425:L1MEf(LINE)" | bnd_40 | del_invers_1097_0/1001_0 |
| 35T | 3 | 100209024 | 100209024 | . | intergenic | 3q12.2 | Score=1616;Name="3082621:L1MD1(LINE)" | bnd_148 | tandem_dup_1825 |
| 35T | 3 | 100305159 | 100305159 | . | intergenic | 3q12.2 | Score=5475;Name="3082785:MER41-int(LTR)" | bnd_147 | tandem_dup_1825 |
| 35T | 3 | 123293167 | 123293167 | PTPLB | intronic | 3q21.1 | . | bnd_150 | tandem_dup_2726 |
| 35T | 3 | 123367930 | 123367930 | MYLK | intronic | 3q21.1 | . | bnd_149 | tandem_dup_2726 |
| 35T | 3 | 128487398 | 128487398 | RAB7A | intronic | 3q21.3 | Score=1564;Name="3130625:AluJr(SINE)" | bnd_235 | transl_inter_3118_0 |
| 35T | 3 | 128487790 | 128487790 | RAB7A | intronic | 3q21.3 | Score=348;Name="3130627:T-rich(Low_complexity)" | bnd_241 | transl_inter_4755_0 |

| | | | | | | | | |
|---|---|---|---|---|---|---|---|---|
| 35T | 3 | 128487827 | 128487827 | RAB7A | intronic | 3q21.3 | Score=348;Name="3130627:T-rich(Low_complexity)" | bnd_243 | transl_inter_3110_0 |
| 35T | 3 | 133399639 | 133399639 | . | intergenic | 3q22.1 | . | bnd_152 | tandem_dup_1276 |
| 35T | 3 | 133518154 | 133518154 | SRPRB | intronic | 3q22.1 | Score=340;Name="3140192:L1MC4a(LINE)" | bnd_151 | tandem_dup_1276 |
| 35T | 3 | 162698173 | 162698173 | . | intergenic | 3q26.1 | Score=1026;Name="3190971:MLT1C(LTR)" | bnd_154 | tandem_dup_2042 |
| 35T | 3 | 162732739 | 162732739 | . | intergenic | 3q26.1 | Score=860;Name="3191030:MER34A1(LTR)" | bnd_153 | tandem_dup_2042 |
| 35T | 3 | 37217397 | 37217397 | LRRFIP2 | intronic | 3p22.2 | . | bnd_15 | del_2410 |
| 35T | 4 | 3190750 | 3190750 | HTT | exonic | 4p16.3 | . | bnd_156 | tandem_dup_1464 |
| 35T | 4 | 3196088 | 3196088 | HTT | intronic | 4p16.3 | . | bnd_155 | tandem_dup_1464 |
| 35T | 4 | 112713798 | 112713798 | . | intergenic | 4q25 | . | bnd_158 | tandem_dup_1066 |
| 35T | 4 | 112782300 | 112782300 | . | intergenic | 4q25 | . | bnd_157 | tandem_dup_1066 |
| 35T | 4 | 123896621 | 123896621 | SPATA5 | intronic | 4q28.1 | Score=1016;Name="3466985:LTR40b(LTR)" | bnd_160 | tandem_dup_1650 |
| 35T | 4 | 123910893 | 123910893 | SPATA5 | intronic | 4q28.1 | Score=1883;Name="3467001:L1MA9(LINE)" | bnd_159 | tandem_dup_1650 |
| 35T | 4 | 173198125 | 173198125 | GALNTL6 | intronic | 4q34.1 | . | bnd_162 | tandem_dup_1529 |
| 35T | 4 | 173201295 | 173201295 | GALNTL6 | intronic | 4q34.1 | Score=4236;Name="3548600:L1MB2(LINE)" | bnd_263 | transl_inter_3100 |
| 35T | 4 | 173203412 | 173203412 | GALNTL6 | intronic | 4q34.1 | Score=713;Name="3548608:L1M5(LINE)" | bnd_161 | tandem_dup_1529 |
| 35T | 5 | 13836453 | 13836453 | DNAH5 | intronic | 5p15.2 | Score=1241;Name="3599433:L2a(LINE)" | bnd_164 | tandem_dup_4206 |
| 35T | 5 | 13932007 | 13932007 | DNAH5 | intronic | 5p15.2 | Score=262;Name="3599585:L1ME4a(LINE)" | bnd_163 | tandem_dup_4206 |
| 35T | 5 | 13977426 | 13977426 | . | intergenic | 5p15.2 | . | bnd_166 | tandem_dup_1410 |
| 35T | 5 | 14014573 | 14014573 | . | intergenic | 5p15.2 | Score=302;Name="3599740:MIR(SINE)" | bnd_165 | tandem_dup_1410 |
| 35T | 5 | 43783463 | 43783463 | . | intergenic | 5p12 | Score=3594;Name="3651006:L1MC4(LINE)" | bnd_168 | tandem_dup_1586 |
| 35T | 5 | 43837837 | 43837837 | . | intergenic | 5p12 | . | bnd_167 | tandem_dup_1586 |
| 35T | 5 | 56192447 | 56192447 | . | downstream | 5q11.2 | Score=1868;Name="3665346:L1MC4(LINE)" | bnd_44 | transl_intra_3777_0 |
| 35T | 5 | 56192470 | 56192470 | . | downstream | 5q11.2 | Score=1868;Name="3665346:L1MC4(LINE)" | bnd_251 | transl_inter_1166 |
| 35T | 5 | 56193637 | 56193637 | . | intergenic | 5q11.2 | . | bnd_43 | transl_intra_3777_0 |
| 35T | 5 | 56269918 | 56269918 | . | intergenic | 5q11.2 | Score=1582;Name="3665485:L1ME5(LINE)" | bnd_249 | transl_inter_2429 |
| 35T | 5 | 125610153 | 125610153 | LOC101927488 | ncRNA_intronic | 5q23.2 | . | bnd_257 | transl_inter_1604 |
| 35T | 5 | 132719332 | 132719332 | FSTL4 | intronic | 5q31.1 | . | bnd_170 | tandem_dup_970 |
| 35T | 5 | 132785588 | 132785588 | FSTL4 | intronic | 5q31.1 | . | bnd_169 | tandem_dup_970 |
| 35T | 5 | 134203006 | 134203006 | . | intergenic | 5q31.1 | Score=1850;Name="3794607:AluJb(SINE)" | bnd_238 | del_inso_3113_0/2182_0 |
| 35T | 5 | 134203041 | 134203041 | . | intergenic | 5q31.1 | Score=1850;Name="3794607:AluJb(SINE)" | bnd_240 | del_inso_3113_0/2182_0 |
| 35T | 5 | 138160473 | 138160473 | CTNNA1 | exonic | 5q31.2 | . | bnd_172 | tandem_dup_4219 |
| 35T | 5 | 138372067 | 138372067 | SIL1 | intronic | 5q31.2 | Score=3228;Name="3802007:L1PREC2(LINE)" | bnd_171 | tandem_dup_4219 |
| 35T | 5 | 142718032 | 142718032 | NR3C1 | intronic | 5q31.3 | Score=6082;Name="3810602:Tigger3b(DNA)" | bnd_174 | tandem_dup_1154 |
| 35T | 5 | 142923214 | 142923214 | . | intergenic | 5q31.3 | . | bnd_173 | tandem_dup_1154 |

| | | | | | | | | |
|---|---|---|---|---|---|---|---|---|
| 35T 5 | 150178963 | 150178963 | . | intergenic | 5q33.1 | Score=688;Name="3825564:AluSz(SINE)" | bnd_176 | tandem_dup_2960 |
| 35T 5 | 150293377 | 150293377 | . | intergenic | 5q33.1 | Score=4228;Name="3825735:L1M3de(LINE)" | bnd_175 | tandem_dup_2960 |
| 35T 6 | 3712808 | 3712808 | . | intergenic | 6p25.2 | . | bnd_18 | del_1507 |
| 35T 6 | 3719075 | 3719075 | . | intergenic | 6p25.2 | Score=2473;Name="3893365:MER74A(LTR)" | bnd_17 | del_1507 |
| 35T 6 | 11534652 | 11534652 | . | intergenic | 6p24.2 | Score=521;Name="3907262:MER96B(DNA)" | bnd_178 | tandem_dup_780 |
| 35T 6 | 11651675 | 11651675 | . | intergenic | 6p24.1 | Score=306;Name="3907456:MLT1L(LTR)" | bnd_177 | tandem_dup_780 |
| 35T 6 | 13405078 | 13405078 | GFOD1 | intronic | 6p23 | . | bnd_180 | tandem_dup_2904 |
| 35T 6 | 25711849 | 25711849 | . | intergenic | 6p22.2 | . | bnd_182 | tandem_dup_677 |
| 35T 6 | 25712334 | 25712334 | . | intergenic | 6p22.2 | Score=375;Name="3933547:MIR3(SINE)" | bnd_46 | transl_intra_1107 |
| 35T 6 | 25723925 | 25723925 | . | intergenic | 6p22.2 | Score=1762;Name="3933571:AluSg(SINE)" | bnd_181 | tandem_dup_677 |
| 35T 6 | 25725093 | 25725093 | . | intergenic | 6p22.2 | . | bnd_45 | transl_intra_1107 |
| 35T 6 | 37995231 | 37995231 | ZFAND3 | intronic | 6p21.2 | . | bnd_184 | tandem_dup_1921 |
| 35T 6 | 38086477 | 38086477 | ZFAND3 | intronic | 6p21.2 | . | bnd_183 | tandem_dup_1921 |
| 35T 6 | 78444048 | 78444048 | MEI4 | intronic | 6q14.1 | Score=25398;Name="4021391:L1PA3(LINE)" | bnd_186 | tandem_dup_2324 |
| 35T 6 | 78491661 | 78491661 | MEI4 | intronic | 6q14.1 | . | bnd_185 | tandem_dup_2324 |
| 35T 6 | 90481510 | 90481510 | MDN1 | intronic | 6q15 | Score=2052;Name="4041087:AluJo(SINE)" | bnd_188 | tandem_dup_1564 |
| 35T 6 | 90600906 | 90600906 | . | intergenic | 6q15 | Score=6813;Name="4041422:LTR5_Hs(LTR)" | bnd_187 | tandem_dup_1564 |
| 35T 6 | 112327490 | 112327490 | . | intergenic | 6q21 | Score=4078;Name="4077741:HERV16-int(LTR)" | bnd_190 | tandem_dup_1106 |
| 35T 6 | 112394227 | 112394227 | TUBE1 | intronic | 6q21 | Score=25;Name="4077842:AT_rich(Low_complexity)" | bnd_189 | tandem_dup_1106 |
| 35T 6 | 116312610 | 116312610 | FRK | intronic | 6q22.1 | Score=10564;Name="4084178:L1PB4(LINE)" | bnd_239 | del_inso_3113_0/2182_0 |
| 35T 6 | 116481760 | 116481760 | NT5DC1 | intronic | 6q22.1 | Score=12648;Name="4084423:L1PA17(LINE)" | bnd_237 | del_inso_3113_0/2182_0 |
| 35T 6 | 125185614 | 125185614 | . | intergenic | 6q22.31 | Score=1991;Name="4098268:AluSx(SINE)" | bnd_192 | tandem_dup_1403_0 |
| 35T 6 | 125268514 | 125268514 | STL | ncRNA_intronic | 6q22.31 | . | bnd_22 | del_ins_1079_0 |
| 35T 6 | 125269403 | 125269403 | STL | ncRNA_intronic | 6q22.31 | . | bnd_191 | tandem_dup_1403_0 |
| 35T 6 | 159513448 | 159513448 | . | intergenic | 6q25.3 | . | bnd_21 | del_ins_1079_0 |
| 35T 6 | 160090229 | 160090229 | . | intergenic | 6q25.3 | . | bnd_253 | transl_inter_4136 |
| 35T 6 | 13769797 | 13769797 | . | intergenic | 6p23 | . | bnd_179 | tandem_dup_2904 |
| 35T 7 | 27149196 | 27149196 | HOXA3 | intronic | 7p15.2 | . | bnd_194 | tandem_dup_1180 |
| 35T 7 | 27249079 | 27249079 | . | intergenic | 7p15.2 | Score=1542;Name="4272983:AluJb(SINE)" | bnd_193 | tandem_dup_1180 |
| 35T 7 | 28923236 | 28923236 | . | intergenic | 7p14.3 | . | bnd_196 | tandem_dup_1491_0 |
| 35T 7 | 36471031 | 36471031 | ANLN | intronic | 7p14.2 | . | bnd_20 | del_1427 |
| 35T 7 | 36491768 | 36491768 | ANLN | intronic | 7p14.2 | . | bnd_19 | del_1427 |
| 35T 7 | 87006110 | 87006110 | CROT | intronic | 7q21.12 | Score=677;Name="4378923:MER112(DNA)" | bnd_198 | tandem_dup_291 |
| 35T 7 | 87051987 | 87051987 | ABCB4 | intronic | 7q21.12 | Score=426;Name="4378984:L2a(LINE)" | bnd_197 | tandem_dup_291 |
| 35T 7 | 105661996 | 105661996 | CDHR3 | intronic | 7q22.3 | . | bnd_200 | tandem_dup_1004 |
| 35T 7 | 105772962 | 105772962 | . | intergenic | 7q22.3 | Score=6530;Name="4413877:Tigger3(DNA)" | bnd_199 | tandem_dup_1004 |

| | | | | | | | | |
|---|---|---|---|---|---|---|---|---|
| 35T | 7 | 118435338 | 118435338 | . | intergenic | 7q31.31 | . | bnd_202 | tandem_dup_2060 |
| 35T | 7 | 118499922 | 118499922 | . | intergenic | 7q31.31 | . | bnd_201 | tandem_dup_2060 |
| 35T | 7 | 139643439 | 139643439 | TBXAS1 | intronic | 7q34 | . | bnd_204 | tandem_dup_1800 |
| 35T | 7 | 139714230 | 139714230 | TBXAS1 | intronic | 7q34 | . | bnd_203 | tandem_dup_1800 |
| 35T | 7 | 150752622 | 150752622 | CDK5 | exonic | 7q36.1 | . | bnd_206 | tandem_dup_3004 |
| 35T | 7 | 150821031 | 150821031 | AGAP3 | intronic | 7q36.1 | . | bnd_205 | tandem_dup_3004 |
| 35T | 7 | 100291302 | 100291302 | . | intergenic | 7q22.1 | . | bnd_195 | tandem_dup_1491_0 |
| 35T | 8 | 10896852 | 10896852 | XKR6 | intronic | 8p23.1 | . | bnd_208 | tandem_dup_897 |
| 35T | 8 | 10963547 | 10963547 | XKR6 | intronic | 8p23.1 | . | bnd_207 | tandem_dup_897 |
| 35T | 8 | 39713281 | 39713281 | . | intergenic | 8p11.21 | . | bnd_210 | tandem_dup_1854 |
| 35T | 8 | 39971107 | 39971107 | . | intergenic | 8p11.21 | . | bnd_209 | tandem_dup_1854 |
| 35T | 8 | 56704485 | 56704485 | TGS1 | intronic | 8q12.1 | . | bnd_212 | tandem_dup_1655 |
| 35T | 8 | 56783754 | 56783754 | . | intergenic | 8q12.1 | Score=500;Name="4599043:Zaphod(DNA)" | bnd_211 | tandem_dup_1655 |
| 35T | 8 | 66953969 | 66953969 | DNAJC5B | intronic | 8q13.1 | . | bnd_214 | tandem_dup_1089 |
| 35T | 8 | 67032052 | 67032052 | . | intergenic | 8q13.1 | Score=818;Name="4615580:MER3(DNA)" | bnd_213 | tandem_dup_1089 |
| 35T | 8 | 72151079 | 72151079 | EYA1 | intronic | 8q13.3 | Score=420;Name="4625098:L1M5(LINE)" | bnd_216 | tandem_dup_2394_0 |
| 35T | 8 | 72151549 | 72151549 | EYA1 | intronic | 8q13.3 | . | bnd_24 | del_ins_1241 |
| 35T | 8 | 72209312 | 72209312 | EYA1 | intronic | 8q13.3 | . | bnd_23 | del_ins_1241 |
| 35T | 8 | 72211592 | 72211592 | EYA1 | intronic | 8q13.3 | . | bnd_215 | tandem_dup_2394_0 |
| 35T | 8 | 123931338 | 123931338 | ZHX2 | intronic | 8q24.13 | . | bnd_218 | tandem_dup_1539 |
| 35T | 8 | 124038888 | 124038888 | DERL1 | intronic | 8q24.13 | Score=483;Name="4715371:MIR3(SINE)" | bnd_217 | tandem_dup_1539 |
| 35T | 9 | 23357496 | 23357496 | . | intergenic | 9p21.3 | Score=13213;Name="4797959:L1PBa(LINE)" | bnd_220 | tandem_dup_2503 |
| 35T | 9 | 23496901 | 23496901 | . | intergenic | 9p21.3 | . | bnd_219 | tandem_dup_2503 |
| 35T | 9 | 73400050 | 73400050 | TRPM3 | intronic | 9q21.12 | Score=197;Name="4850797:L2b(LINE)" | bnd_222 | tandem_dup_1029 |
| 35T | 9 | 73486269 | 73486269 | TRPM3 | intronic | 9q21.12 | . | bnd_221 | tandem_dup_1029 |
| 35T | 9 | 92226612 | 92226612 | . | intergenic | 9q22.2 | . | bnd_224 | tandem_dup_2154 |
| 35T | 9 | 92309598 | 92309598 | UNQ6494 | ncRNA_intronic | 9q22.2 | Score=242;Name="4884786:MIR(SINE)" | bnd_223 | tandem_dup_2154 |
| 35T | 9 | 103973018 | 103973018 | LPPR1 | intronic | 9q31.1 | . | bnd_226 | tandem_dup_1588 |
| 35T | 9 | 104342769 | 104342769 | GRIN3A | intronic | 9q31.1 | . | bnd_225 | tandem_dup_1588 |
| 35T | 9 | 108428666 | 108428666 | . | intergenic | 9q31.2 | Score=418;Name="4914800:MIR3(SINE)" | bnd_228 | tandem_dup_3960 |
| 35T | 9 | 108508469 | 108508469 | TMEM38B | intronic | 9q31.2 | Score=20095;Name="4914961:L1PA4(LINE)" | bnd_227 | tandem_dup_3960 |
| 35T | 9 | 132354806 | 132354806 | . | intergenic | 9q34.11 | Score=6612;Name="4967011:HERVFH19-int(LTR)" | bnd_230 | tandem_dup_2369 |
| 35T | 9 | 132494839 | 132494839 | . | intergenic | 9q34.11 | . | bnd_229 | tandem_dup_2369 |
| 35T | 10 | 34149126 | 34149126 | . | intergenic | 10p11.22 | . | bnd_2 | del_2103 |
| 35T | 10 | 34150284 | 34150284 | . | intergenic | 10p11.22 | . | bnd_1 | del_2103 |
| 35T | 10 | 44328425 | 44328425 | . | intergenic | 10q11.21 | Score=24133;Name="507417:L1P3(LINE)" | bnd_68 | tandem_dup_5078 |
| 35T | 10 | 44383076 | 44383076 | LINC00840 | ncRNA_intronic | 10q11.21 | Score=5268;Name="507493:L1MA9(LINE)" | bnd_67 | tandem_dup_5078 |
| 35T | 10 | 91791254 | 91791254 | . | intergenic | 10q23.31 | Score=2055;Name="592598:AluSc(SINE)" | bnd_70 | tandem_dup_3774 |
| 35T | 10 | 91862448 | 91862448 | . | intergenic | 10q23.31 | Score=13391;Name="592716:L1MA3(LINE)" | bnd_69 | tandem_dup_3774 |
| 35T | 10 | 101156069 | 101156069 | . | downstream | 10q24.2 | Score=219;Name="611331:L3(LINE)" | bnd_72 | tandem_dup_1094_0 |
| 35T | 10 | 101278332 | 101278332 | . | intergenic | 10q24.2 | Score=1923;Name="611585:AluJb(SINE)" | bnd_71 | tandem_dup_1094_0 |
| 35T | 10 | 101291558 | 101291558 | . | intergenic | 10q24.2 | . | bnd_74 | tandem_dup_6233 |
| 35T | 10 | 101292163 | 101292163 | . | upstream | 10q24.2 | Score=298;Name="611598:L2c(LINE)" | bnd_73 | tandem_dup_6233 |

| | | | | | | | | |
|---|---|---|---|---|---|---|---|---|
| 35T 10 | 112044827 | 112044827 | MXI1 | UTR3 | 10q25.2 | . | bnd_4 | del_3186 |
| 35T 10 | 112050239 | 112050239 | . | intergenic | 10q25.2 | . | bnd_3 | del_3186 |
| 35T 10 | 124222799 | 124222799 | HTRA1 | intronic | 10q26.13 | . | bnd_76 | tandem_dup_1300 |
| 35T 10 | 124265242 | 124265242 | HTRA1 | intronic | 10q26.13 | . | bnd_75 | tandem_dup_1300 |
| 35T 10 | 124389624 | 124389624 | DMBT1 | intronic | 10q26.13 | . | bnd_78 | tandem_dup_2287 |
| 35T 10 | 124446107 | 124446107 | . | intergenic | 10q26.13 | Score=2299;Name="654572:AluSx1(SINE)" | bnd_77 | tandem_dup_2287 |
| 35T 10 | 125132348 | 125132348 | . | intergenic | 10q26.13 | . | bnd_80 | tandem_dup_2258 |
| 35T 10 | 125132718 | 125132718 | . | intergenic | 10q26.13 | . | bnd_244 | transl_inter_3110_0 |
| 35T 10 | 125132719 | 125132719 | . | intergenic | 10q26.13 | . | bnd_242 | transl_inter_4755_0 |
| 35T 10 | 125205566 | 125205566 | . | intergenic | 10q26.13 | Score=240;Name="655818:MIRc(SINE)" | bnd_236 | transl_inter_3118_0 |
| 35T 10 | 125205841 | 125205841 | . | intergenic | 10q26.13 | . | bnd_79 | tandem_dup_2258 |
| 35T 11 | 42161539 | 42161539 | . | intergenic | 11p12 | Score=340;Name="744968:L1MEf(LINE)" | bnd_248 | transl_inter_2474 |
| 35T 11 | 42299991 | 42299991 | . | intergenic | 11p12 | Score=1921;Name="745221:AluSz6(SINE)" | bnd_245 | transl_inter_6640 |
| 35T 11 | 65161711 | 65161711 | FRMD8 | intronic | 11q13.1 | . | bnd_6 | del_1929 |
| 35T 11 | 65199238 | 65199238 | . | intergenic | 11q13.1 | Score=4313;Name="781123:L1MB2(LINE)" | bnd_5 | del_1929 |
| 35T 11 | 101859211 | 101859211 | KIAA1377 | intronic | 11q22.1 | Score=812;Name="851417:L1MEd(LINE)" | bnd_82 | tandem_dup_2715 |
| 35T 11 | 101944107 | 101944107 | C11orf70 | intronic | 11q22.1 | Score=10727;Name="851646:L1M2(LINE)" | bnd_81 | tandem_dup_2715 |
| 35T 11 | 117450448 | 117450448 | DSCAML1 | intronic | 11q23.3 | Score=1704;Name="879836:L2a(LINE)" | bnd_84 | tandem_dup_845 |
| 35T 11 | 117535268 | 117535268 | DSCAML1 | intronic | 11q23.3 | Score=432;Name="879989:MIRb(SINE)" | bnd_83 | tandem_dup_845 |
| 35T 11 | 119163460 | 119163460 | CBL | intronic | 11q23.3 | Score=648;Name="883530:AluJo(SINE)" | bnd_85 | tandem_dup_1735 |
| 35T 11 | 119077164 | 119077164 | CBL | exonic | 11q23.3 | . | bnd_86 | tandem_dup_1735 |
| 35T 12 | 1686255 | 1686255 | FBXL14 | intronic | 12p13.33 | . | bnd_88 | tandem_dup_1633_0 |
| 35T 12 | 1764280 | 1764280 | . | intergenic | 12p13.33 | . | bnd_87 | tandem_dup_1633_0 |
| 35T 12 | 38899625 | 38899625 | . | intergenic | 12q12 | Score=342;Name="972939:L2c(LINE)" | bnd_90 | tandem_dup_1523 |
| 35T 12 | 39078550 | 39078550 | CPNE8 | intronic | 12q12 | . | bnd_89 | tandem_dup_1523 |
| 35T 12 | 56972188 | 56972188 | RBMS2 | intronic | 12q13.3 | Score=2275;Name="1006326:AluSz(SINE)" | bnd_92 | tandem_dup_1666 |
| 35T 12 | 57112161 | 57112161 | NACA | intronic | 12q13.3 | . | bnd_91 | tandem_dup_1666 |
| 35T 12 | 68370174 | 68370174 | . | intergenic | 12q15 | Score=22664;Name="1026421:L1PA8(LINE)" | bnd_250 | transl_inter_2429 |
| 35T 12 | 68370964 | 68370964 | . | intergenic | 12q15 | Score=22664;Name="1026421:L1PA8(LINE)" | bnd_252 | transl_inter_1166 |
| 35T 13 | 48992678 | 48992678 | LPAR6,RB1 | intronic | 13q14.2 | Score=22937;Name="1214258:L1PA7(LINE)" | bnd_254 | transl_inter_4136 |
| 35T 13 | 69957102 | 69957102 | . | intergenic | 13q21.33 | Score=310;Name="1249020:(TA)n(Simple_repeat)" | bnd_94 | tandem_dup_2490 |
| 35T 13 | 69989955 | 69989955 | . | intergenic | 13q21.33 | Score=634;Name="1249065:MER31-int(LTR)" | bnd_93 | tandem_dup_2490 |
| 35T 14 | 32394134 | 32394134 | . | intergenic | 14q12 | . | bnd_256 | transl_inter_1787 |
| 35T 14 | 59912654 | 59912654 | . | intergenic | 14q23.1 | Score=11811;Name="1391685:HERVH-int(LTR)" | bnd_96 | tandem_dup_2928 |
| 35T 14 | 59958469 | 59958469 | JKAMP | intronic | 14q23.1 | . | bnd_95 | tandem_dup_2928 |
| 35T 15 | 55453352 | 55453352 | . | intergenic | 15q21.3 | Score=1481;Name="1543858:L1MA7(LINE)" | bnd_255 | transl_inter_1787 |
| 35T 16 | 3052949 | 3052949 | . | intergenic | 16p13.3 | . | bnd_98 | tandem_dup_1574 |
| 35T 16 | 3177821 | 3177821 | . | intergenic | 16p13.3 | . | bnd_97 | tandem_dup_1574 |

| | | | | | | | | |
|---|---|---|---|---|---|---|---|---|
| 35T 16 | 47309529 | 47309529 | ITFG1 | intronic | 16q12.1 | . | bnd_100 | tandem_dup_1208 |
| 35T 16 | 47317549 | 47317549 | ITFG1 | intronic | 16q12.1 | Score=1260;Name="1718004:MLT1D(LTR)" | bnd_99 | tandem_dup_1208 |
| 35T 16 | 47321284 | 47321284 | ITFG1 | intronic | 16q12.1 | Score=612;Name="1718014:FLAM_C(SINE)" | bnd_247 | transl_inter_2474 |
| 35T 16 | 57665097 | 57665097 | GPR56 | intronic | 16q21 | . | bnd_102 | tandem_dup_1748 |
| 35T 16 | 57764260 | 57764260 | CCDC135 | intronic | 16q21 | Score=957;Name="1739423:(TGGA)n(Simple_repeat)" | bnd_101 | tandem_dup_1748 |
| 35T 16 | 68084048 | 68084048 | DUS2 | intronic | 16q22.1 | Score=2277;Name="1759297:AluSg(SINE)" | bnd_258 | transl_inter_1604 |
| 35T 17 | 3953700 | 3953700 | ZZEF1 | intronic | 17p13.2 | . | bnd_48 | transl_intra_2411 |
| 35T 17 | 4535691 | 4535691 | ALOX15 | intronic | 17p13.2 | . | bnd_42 | transl_intra_1486_0 |
| 35T 17 | 4575032 | 4575032 | PELP1 | exonic | 17p13.2 | . | bnd_47 | transl_intra_2411 |
| 35T 17 | 4575836 | 4575836 | PELP1 | exonic | 17p13.2 | . | bnd_41 | transl_intra_1486_0 |
| 35T 17 | 7221508 | 7221508 | NEURL4 | intronic | 17p13.1 | . | bnd_104 | tandem_dup_799 |
| 35T 17 | 7316350 | 7316350 | NLGN2 | intronic | 17p13.1 | . | bnd_103 | tandem_dup_799 |
| 35T 17 | 39204708 | 39204708 | . | intergenic | 17q21.2 | . | bnd_106 | tandem_dup_1820 |
| 35T 17 | 39221711 | 39221711 | KRTAP2-4 | exonic | 17q21.2 | . | bnd_105 | tandem_dup_1820 |
| 35T 17 | 41494639 | 41494639 | . | intergenic | 17q21.31 | Score=843;Name="1887241:L2c(LINE)" | bnd_31 | del_insou_5408_0/3244_0 |
| 35T 17 | 41494933 | 41494933 | . | intergenic | 17q21.31 | . | bnd_29 | del_insou_5408_0/3244_0 |
| 35T 17 | 41496008 | 41496008 | . | intergenic | 17q21.31 | Score=588;Name="1887243:MIRc(SINE)" | bnd_108 | tandem_dup_1556 |
| 35T 17 | 41568703 | 41568703 | DHX8 | intronic | 17q21.31 | . | bnd_30 | del_insou_5408_0/3244_0 |
| 35T 17 | 45866033 | 45866033 | . | intergenic | 17q21.32 | Score=2016;Name="1897541:L1M4(LINE)" | bnd_32 | del_insou_5408_0/3244_0 |
| 35T 17 | 45904961 | 45904961 | MRPL10 | intronic | 17q21.32 | . | bnd_107 | tandem_dup_1556 |
| 35T 17 | 46327790 | 46327790 | SKAP1 | intronic | 17q21.32 | Score=2464;Name="1898351:AluSg(SINE)" | bnd_110 | tandem_dup_2142 |
| 35T 17 | 46391178 | 46391178 | SKAP1 | intronic | 17q21.32 | . | bnd_109 | tandem_dup_2142 |
| 35T 17 | 56389210 | 56389210 | BZRAP1 | intronic | 17q22 | . | bnd_112 | tandem_dup_1314 |
| 35T 17 | 56457015 | 56457015 | RNF43 | intronic | 17q22 | . | bnd_111 | tandem_dup_1314 |
| 35T 17 | 65884051 | 65884051 | BPTF | intronic | 17q24.2 | . | bnd_260 | transl_inter_2807_0 |
| 35T 17 | 65884495 | 65884495 | BPTF | intronic | 17q24.2 | Score=2614;Name="1938966:AluSx3(SINE)" | bnd_262 | transl_inter_2455 |
| 35T 18 | 8782650 | 8782650 | SOGA2 | intronic | 18p11.22 | . | bnd_114 | tandem_dup_1448 |
| 35T 18 | 8931017 | 8931017 | . | intergenic | 18p11.22 | . | bnd_113 | tandem_dup_1448 |
| 35T 18 | 11404777 | 11404777 | . | intergenic | 18p11.21 | Score=725;Name="1991890:L1MEf(LINE)" | bnd_116 | tandem_dup_2858 |
| 35T 18 | 11471250 | 11471250 | . | intergenic | 18p11.21 | . | bnd_115 | tandem_dup_2858 |
| 35T 18 | 36173532 | 36173532 | . | intergenic | 18q12.2 | . | bnd_118 | tandem_dup_2628 |
| 35T 18 | 36191804 | 36191804 | . | intergenic | 18q12.2 | . | bnd_117 | tandem_dup_2628 |
| 35T 18 | 42238530 | 42238530 | . | intergenic | 18q12.3 | . | bnd_8 | del_1157 |
| 35T 18 | 42637862 | 42637862 | SETBP1 | intronic | 18q12.3 | . | bnd_7 | del_1157 |
| 35T 18 | 55884419 | 55884419 | NEDD4L | intronic | 18q21.31 | . | bnd_120 | tandem_dup_1568 |
| 35T 18 | 56203841 | 56203841 | ALPK2 | exonic | 18q21.32 | . | bnd_119 | tandem_dup_1568 |
| 35T 18 | 59275089 | 59275089 | . | intergenic | 18q21.33 | Score=3061;Name="2069120:MER34C_(LTR)" | bnd_122 | tandem_dup_1566 |
| 35T 18 | 59327732 | 59327732 | . | intergenic | 18q21.33 | . | bnd_121 | tandem_dup_1566 |
| 35T 18 | 65148898 | 65148898 | . | intergenic | 18q22.1 | Score=1872;Name="2079158:Charlie10(DNA)" | bnd_10 | del_884 |
| 35T 18 | 65397556 | 65397556 | RP11-638L3.1 | ncRNA_intronic | 18q22.1 | . | bnd_9 | del_884 |
| 35T 19 | 2006656 | 2006656 | BTBD2 | intronic | 19p13.3 | Score=951;Name="2102701:MER58A(DNA)" | bnd_124 | tandem_dup_2059 |
| 35T 19 | 16647739 | 16647739 | CHERP | intronic | 19p13.11 | Score=2197;Name="2142139:AluSx1(SINE)" | bnd_264 | transl_inter_3100 |
| 35T 19 | 2116712 | 2116712 | AP3D1 | exonic | 19p13.3 | . | bnd_123 | tandem_dup_2059 |
| 35T 20 | 19691589 | 19691589 | SLC24A3 | intronic | 20p11.23 | . | bnd_138 | tandem_dup_1537 |
| 35T 20 | 19779713 | 19779713 | . | intergenic | 20p11.23 | Score=1862;Name="2686370:AluJb(SINE)" | bnd_137 | tandem_dup_1537 |

| | | | | | | | | |
|---|---|---|---|---|---|---|---|---|
| 35T 20 | 30378094 | 30378094 | TPX2 | intronic | 20q11.21 | Score=666;Name="2698747:MARNA(DNA)" | bnd_140 | tandem_dup_1319 |
| 35T 20 | 30614587 | 30614587 | CCM2L | intronic | 20q11.21 | Score=2197;Name="2699381:AluSz6(SINE)" | bnd_139 | tandem_dup_1319 |
| 35T 20 | 42976160 | 42976160 | R3HDML | intronic | 20q13.12 | . | bnd_259 | transl_inter_2807_0 |
| 35T 20 | 43114404 | 43114404 | TTPAL | intronic | 20q13.12 | . | bnd_261 | transl_inter_2455 |
| 35T 21 | 30267083 | 30267083 | . | intergenic | 21q21.3 | . | bnd_142 | tandem_dup_528 |
| 35T 21 | 30329144 | 30329144 | LTN1 | exonic | 21q21.3 | . | bnd_141 | tandem_dup_528 |
| 35T 21 | 35843972 | 35843972 | KCNE1 | intronic | 21q22.12 | Score=1441;Name="2811989:L1MEe(LINE)" | bnd_144 | tandem_dup_2892 |
| 35T 21 | 35904146 | 35904146 | RCAN1 | intronic | 21q22.12 | Score=250;Name="2812077:L2c(LINE)" | bnd_143 | tandem_dup_2892 |
| 35T 22 | 24757578 | 24757578 | SPECC1L,SPECC1L-ADORA2A | ncRNA_intronic | 22q11.23 | Score=1852;Name="2848799:AluJb(SINE)" | bnd_146 | tandem_dup_2935 |
| 35T 22 | 24818395 | 24818395 | SPECC1L-ADORA2A | ncRNA_intronic | 22q11.23 | . | bnd_145 | tandem_dup_2935 |
| 35T X | 45571271 | 45571271 | . | intergenic | Xp11.3 | Score=364;Name="5075809:MER91C(DNA)" | bnd_232 | tandem_dup_1075 |
| 35T X | 45659691 | 45659691 | . | intergenic | Xp11.3 | . | bnd_231 | tandem_dup_1075 |
| 35T X | 98504928 | 98504928 | . | intergenic | Xq22.1 | Score=3770;Name="5156350:L1M4(LINE)" | bnd_26 | del_insod_1205_0/916_0 |
| 35T X | 98636710 | 98636710 | . | intergenic | Xq22.1 | . | bnd_28 | del_insod_1205_0/916_0 |
| 35T X | 98639541 | 98639541 | . | intergenic | Xq22.1 | Score=6334;Name="5156531:MER52A(LTR)" | bnd_36 | del_inssd_1415_0/1155_0 |
| 35T X | 98640040 | 98640040 | . | intergenic | Xq22.1 | Score=6334;Name="5156531:MER52A(LTR)" | bnd_50 | transl_intra_1150_0 |
| 35T X | 98665283 | 98665283 | . | intergenic | Xq22.1 | Score=378;Name="5156568:L2(LINE)" | bnd_34 | del_inssd_1415_0/1155_0 |
| 35T X | 100390589 | 100390589 | CENPI | intronic | Xq22.1 | . | bnd_234 | tandem_dup_3402 |
| 35T X | 100426558 | 100426558 | . | intergenic | Xq22.1 | Score=1622;Name="5159546:AluJb(SINE)" | bnd_233 | tandem_dup_3402 |
| 35T X | 101748948 | 101748948 | . | intergenic | Xq22.1 | Score=7065;Name="5161681:MER76-int(LTR)" | bnd_27 | del_insod_1205_0/916_0 |
| 35T X | 101778698 | 101778698 | . | intergenic | Xq22.1 | Score=19549;Name="5161731:L1PA8A(LINE)" | bnd_25 | del_insod_1205_0/916_0 |
| 35T X | 101781550 | 101781550 | . | intergenic | Xq22.1 | Score=1281;Name="5161733:L1ME3C(LINE)" | bnd_35 | del_inssd_1415_0/1155_0 |
| 35T X | 101785294 | 101785294 | . | intergenic | Xq22.1 | Score=4235;Name="5161739:L1MCa(LINE)" | bnd_33 | del_inssd_1415_0/1155_0 |
| 35T X | 101869635 | 101869635 | ARMCX5-GPRASP2 | intronic | Xq22.1 | Score=2626;Name="5161844:Tigger2a(DNA)" | bnd_49 | transl_intra_1150_0 |
| 36T 1 | 110422059 | 110422059 | . | intergenic | 1p13.3 | . | bnd_8 | tandem_dup_1993 |
| 36T 1 | 110540073 | 110540073 | AHCYL1 | intronic | 1p13.3 | . | bnd_7 | tandem_dup_1993 |
| 36T 5 | 150199080 | 150199080 | . | intergenic | 5q33.1 | Score=5447;Name="3825604:L1MA7(LINE)" | bnd_12 | tandem_dup_2247 |
| 36T 5 | 150428910 | 150428910 | TNIP1 | intronic | 5q33.1 | . | bnd_11 | tandem_dup_2247 |
| 36T 6 | 86885486 | 86885486 | . | intergenic | 6q14.3 | . | bnd_2 | del_3660 |
| 36T 6 | 86888151 | 86888151 | . | intergenic | 6q14.3 | Score=22370;Name="4034687:L1PB1(LINE)" | bnd_1 | del_3660 |
| 36T 9 | 123426045 | 123426045 | MEGF9 | intronic | 9q33.2 | . | bnd_14 | tandem_dup_4719 |
| 36T 9 | 123578445 | 123578445 | PSMD5 | UTR3 | 9q33.2 | . | bnd_13 | tandem_dup_4719 |
| 36T 12 | 56996469 | 56996469 | BAZ2A | intronic | 12q13.3 | . | bnd_4 | transl_intra_5735_0 |
| 36T 12 | 56998562 | 56998562 | BAZ2A | exonic | 12q13.3 | . | bnd_3 | transl_intra_5735_0 |
| 36T 19 | 16733212 | 16733212 | MED26 | intronic | 19p13.11 | Score=211;Name="2142295:L2c(LINE)" | bnd_6 | transl_intra_1994_0 |
| 36T 19 | 55432417 | 55432417 | . | intergenic | 19q13.42 | . | bnd_5 | transl_intra_1994_0 |

| | | | | | | | | | |
|---|---|---|---|---|---|---|---|---|---|
| 36T | 20 | 55060063 | 55060063 | RTFDC1 | intronic | 20q13.31 | Score=619;Name="2758667:L2a(LINE)" | bnd_10 | tandem_dup_3764 |
| 36T | 20 | 55358533 | 55358533 | . | intergenic | 20q13.31 | . | bnd_9 | tandem_dup_3764 |
| 37T | 1 | 119664742 | 119664742 | WARS2 | intronic | 1p12 | Score=21793;Name="240098:L1PB1(LINE)" | bnd_50 | transl_inter_743 |
| 37T | 1 | 181327116 | 181327116 | . | intergenic | 1q25.3 | . | bnd_38 | transl_intra_3523_0 |
| 37T | 1 | 181442648 | 181442648 | . | intergenic | 1q25.3 | . | bnd_37 | transl_intra_3523_0 |
| 37T | 1 | 195893357 | 195893357 | . | intergenic | 1q31.3 | Score=370;Name="336126:L2a(LINE)" | bnd_52 | transl_inter_1745 |
| 37T | 2 | 156680570 | 156680570 | . | intergenic | 2q24.1 | Score=2014;Name="2500419:L1M4b(LINE)" | bnd_2 | del_4986 |
| 37T | 2 | 156681583 | 156681583 | . | intergenic | 2q24.1 | Score=3845;Name="2500422:Tigger4(DNA)" | bnd_1 | del_4986 |
| 37T | 4 | 65297075 | 65297075 | . | intergenic | 4q13.1 | . | bnd_62 | transl_inter_1966 |
| 37T | 5 | 26311128 | 26311128 | . | intergenic | 5p14.1 | . | bnd_6 | del_1826 |
| 37T | 5 | 26619014 | 26619014 | . | intergenic | 5p14.1 | Score=28;Name="3621124:AT_rich(Low_complexity)" | bnd_5 | del_1826 |
| 37T | 5 | 126978315 | 126978315 | . | intergenic | 5q23.2 | . | bnd_14 | del_ins_1057 |
| 37T | 5 | 126979546 | 126979546 | . | intergenic | 5q23.2 | . | bnd_13 | del_ins_1057 |
| 37T | 6 | 62341591 | 62341591 | . | intergenic | 6q11.1 | . | bnd_40 | transl_intra_885_0 |
| 37T | 6 | 62341597 | 62341597 | . | intergenic | 6q11.1 | . | bnd_49 | transl_inter_743 |
| 37T | 6 | 62341661 | 62341661 | . | intergenic | 6q11.1 | . | bnd_61 | transl_inter_1966 |
| 37T | 6 | 62341739 | 62341739 | . | intergenic | 6q11.1 | . | bnd_39 | transl_intra_885_0 |
| 37T | 8 | 74189660 | 74189660 | . | intergenic | 8q21.11 | . | bnd_64 | transl_inter_1342 |
| 37T | 8 | 74362048 | 74362048 | STAU2 | intronic | 8q21.11 | . | bnd_44 | transl_intra_1046 |
| 37T | 8 | 74416401 | 74416401 | STAU2 | intronic | 8q21.11 | . | bnd_66 | transl_inter_1023 |
| 37T | 8 | 74424598 | 74424598 | STAU2 | intronic | 8q21.11 | Score=4186;Name="4628775:L1MB7(LINE)" | bnd_43 | transl_intra_1046 |
| 37T | 8 | 129324303 | 129324303 | . | intergenic | 8q24.21 | . | bnd_57 | transl_inter_5360 |
| 37T | 9 | 2420875 | 2420875 | . | intergenic | 9p24.2 | . | bnd_28 | del_inssd_1406_0/2532_0 |
| 37T | 9 | 2422543 | 2422543 | . | intergenic | 9p24.2 | . | bnd_26 | del_inssd_1406_0/2532_0 |
| 37T | 9 | 18164097 | 18164097 | . | intergenic | 9p22.2 | . | bnd_8 | del_2621 |
| 37T | 9 | 18169609 | 18169609 | . | intergenic | 9p22.2 | Score=7655;Name="4789282:L1PA4(LINE)" | bnd_7 | del_2621 |
| 37T | 9 | 82391946 | 82391946 | . | intergenic | 9q21.31 | . | bnd_16 | del_insod_660_0/506_0 |
| 37T | 9 | 82484198 | 82484198 | . | intergenic | 9q21.31 | Score=901;Name="4867080:L2a(LINE)" | bnd_24 | transl_intra_247_0 |
| 37T | 9 | 82486286 | 82486286 | . | intergenic | 9q21.31 | . | bnd_10 | del_474 |
| 37T | 9 | 82762353 | 82762353 | . | intergenic | 9q21.31 | . | bnd_29 | del_inssu_843_0/562_0 |
| 37T | 9 | 83891866 | 83891866 | . | intergenic | 9q21.31 | . | bnd_65 | transl_inter_1023 |
| 37T | 9 | 83891886 | 83891886 | . | intergenic | 9q21.31 | . | bnd_18 | del_insod_660_0/506_0 |
| 37T | 9 | 83911881 | 83911881 | . | intergenic | 9q21.31 | Score=10408;Name="4869318:L1MCa(LINE)" | bnd_31 | del_inssu_843_0/562_0 |
| 37T | 9 | 83913259 | 83913259 | . | intergenic | 9q21.31 | Score=10408;Name="4869318:L1MCa(LINE)" | bnd_59 | transl_inter_674_0 |
| 37T | 9 | 84204598 | 84204598 | TLE1 | intronic | 9q21.32 | Score=554;Name="4869872:MIR(SINE)" | bnd_30 | del_inssu_843_0/562_0 |
| 37T | 9 | 84205294 | 84205294 | TLE1 | intronic | 9q21.32 | . | bnd_9 | del_474 |
| 37T | 9 | 84231360 | 84231360 | TLE1 | intronic | 9q21.32 | . | bnd_12 | del_1863 |
| 37T | 9 | 84234181 | 84234181 | TLE1 | intronic | 9q21.32 | . | bnd_27 | del_inssd_1406_0/2532_0 |
| 37T | 9 | 84366817 | 84366817 | RP11-154D17.1 | ncRNA_intronic | 9q21.32 | . | bnd_63 | transl_inter_1342 |
| 37T | 9 | 110179417 | 110179417 | . | intergenic | 9q31.2 | Score=4808;Name="4918067:L1MB7(LINE)" | bnd_17 | del_insod_660_0/506_0 |
| 37T | 9 | 110508401 | 110508401 | . | intergenic | 9q31.2 | Score=2377;Name="4919002:PRIMA4_LTR(LTR)" | bnd_33 | del_inssu_357_0/644_0 |
| 37T | 9 | 110512301 | 110512301 | . | intergenic | 9q31.2 | . | bnd_35 | del_inssu_357_0/644_0 |
| 37T | 9 | 110512576 | 110512576 | . | intergenic | 9q31.2 | . | bnd_32 | del_inssu_843_0/562_0 |
| 37T | 9 | 110870697 | 110870697 | . | intergenic | 9q31.2 | . | bnd_15 | del_insod_660_0/506_0 |
| 37T | 9 | 110870781 | 110870781 | . | intergenic | 9q31.2 | . | bnd_46 | transl_intra_739 |

| | | | | | | | | | |
|---|---|---|---|---|---|---|---|---|---|
| 37T | 9 | 110901285 | 110901285 | . | intergenic | 9q31.2 | Score=200;Name="4919828:L2(LINE)" | bnd_20 | del_insod_700_0/1895_0 |
| 37T | 9 | 110902265 | 110902265 | . | intergenic | 9q31.2 | Score=1969;Name="4919832:Tigger3a(DNA)" | bnd_22 | del_insod_700_0/1895_0 |
| 37T | 9 | 110902408 | 110902408 | . | intergenic | 9q31.2 | . | bnd_21 | del_insod_700_0/1895_0 |
| 37T | 9 | 110902422 | 110902422 | . | intergenic | 9q31.2 | . | bnd_25 | del_inssd_1406_0/2532_0 |
| 37T | 9 | 111013092 | 111013092 | . | intergenic | 9q31.2 | Score=230;Name="4920038:L1MEe(LINE)" | bnd_19 | del_insod_700_0/1895_0 |
| 37T | 9 | 111232390 | 111232390 | . | intergenic | 9q31.2 | . | bnd_48 | transl_intra_296 |
| 37T | 9 | 111296852 | 111296852 | . | intergenic | 9q31.2 | . | bnd_47 | transl_intra_296 |
| 37T | 9 | 111298526 | 111298526 | . | intergenic | 9q31.2 | . | bnd_34 | del_inssu_357_0/644_0 |
| 37T | 9 | 111299084 | 111299084 | . | intergenic | 9q31.2 | Score=255;Name="4920530:(TTCA)n(Simple_repeat)" | bnd_11 | del_1863 |
| 37T | 9 | 111449459 | 111449459 | . | intergenic | 9q31.3 | . | bnd_55 | transl_inter_350 |
| 37T | 9 | 117448322 | 117448322 | . | intergenic | 9q32 | Score=3581;Name="4933744:L1MD2(LINE)" | bnd_36 | del_inssu_357_0/644_0 |
| 37T | 9 | 118467026 | 118467026 | . | intergenic | 9q33.1 | Score=3485;Name="4935888:L1MB5(LINE)" | bnd_42 | transl_intra_1441_0 |
| 37T | 9 | 119127249 | 119127249 | PAPPA | intronic | 9q33.1 | . | bnd_45 | transl_intra_739 |
| 37T | 9 | 119127938 | 119127938 | PAPPA | intronic | 9q33.1 | . | bnd_41 | transl_intra_1441_0 |
| 37T | 9 | 121177840 | 121177840 | . | intergenic | 9q33.1 | . | bnd_23 | transl_intra_247_0 |
| 37T | 10 | 114516725 | 114516725 | VTI1A | intronic | 10q25.2 | Score=2209;Name="636281:AluJb(SINE)" | bnd_54 | transl_inter_387 |
| 37T | 10 | 114516757 | 114516757 | VTI1A | intronic | 10q25.2 | Score=2209;Name="636281:AluJb(SINE)" | bnd_56 | transl_inter_350 |
| 37T | 11 | 69559272 | 69559272 | . | intergenic | 11q13.3 | Score=1285;Name="790832:L1MB5(LINE)" | bnd_58 | transl_inter_5360 |
| 37T | 20 | 22073081 | 22073081 | . | intergenic | 20p11.22 | Score=3846;Name="2690271:LTR1B(LTR)" | bnd_51 | transl_inter_1745 |
| 37T | 21 | 18840240 | 18840240 | . | intergenic | 21q21.1 | . | bnd_4 | del_820 |
| 37T | 21 | 18925735 | 18925735 | CXADR | intronic | 21q21.1 | . | bnd_60 | transl_inter_674_0 |
| 37T | 21 | 18935494 | 18935494 | CXADR | intronic | 21q21.1 | Score=3660;Name="2781726:Tigger1(DNA)" | bnd_53 | transl_inter_387 |
| 37T | 21 | 18935587 | 18935587 | CXADR | intronic | 21q21.1 | Score=443;Name="2781727:AluJo(SINE)" | bnd_3 | del_820 |
| 41T | 1 | 25537170 | 25537170 | . | intergenic | 1p36.11 | Score=3794;Name="53676:MER21C(LTR)" | bnd_2 | transl_inter_8063 |
| 41T | 1 | 25954467 | 25954467 | MAN1C1 | intronic | 1p36.11 | Score=1633;Name="54734:AluJb(SINE)" | bnd_4 | transl_inter_8104 |
| 41T | 1 | 112098821 | 112098821 | ADORA3 | intronic | 1p13.2 | Score=4532;Name="225925:L1MB3(LINE)" | bnd_6 | transl_inter_6357 |
| 41T | 2 | 32364176 | 32364176 | SPAST | intronic | 2p22.3 | Score=4660;Name="2291441:L1PA16(LINE)" | bnd_38 | transl_inter_5291 |
| 41T | 2 | 59707436 | 59707436 | . | intergenic | 2p16.1 | Score=4650;Name="2339958:L1MA8(LINE)" | bnd_40 | transl_inter_6335 |
| 41T | 2 | 79173565 | 79173565 | . | intergenic | 2p12 | Score=3951;Name="2375946:L1PA7(LINE)" | bnd_27 | transl_inter_5334 |
| 41T | 2 | 88746087 | 88746087 | . | intergenic | 2p11.2 | Score=744;Name="2393121:AluSx3(SINE)" | bnd_42 | transl_inter_7983 |
| 41T | 2 | 100609183 | 100609183 | AFF3 | intronic | 2q11.2 | Score=279;Name="2406238:L2c(LINE)" | bnd_44 | transl_inter_7973 |
| 41T | 2 | 103830148 | 103830148 | . | intergenic | 2q12.1 | Score=389;Name="2411889:L2(LINE)" | bnd_31 | transl_inter_8003 |
| 41T | 2 | 158120810 | 158120810 | GALNT5 | intronic | 2q24.1 | . | bnd_9 | transl_inter_8179 |
| 41T | 3 | 17190549 | 17190549 | . | intergenic | 3p24.3 | . | bnd_7 | transl_inter_7968 |

| | | | | | | | | |
|---|---|---|---|---|---|---|---|---|
| 41T 3 | 115717052 | 115717052 | LSAMP | intronic | 3q13.31 | Score=589;Name="3108016:L2a(LINE)" | bnd_48 | transl_inter_5288 |
| 41T 3 | 150233740 | 150233740 | . | intergenic | 3q25.1 | Score=5364;Name="3169872:L1MB8(LINE)" | bnd_50 | transl_inter_7972 |
| 41T 4 | 104843275 | 104843275 | . | intergenic | 4q24 | Score=447;Name="3436056:L1MC5(LINE)" | bnd_35 | transl_inter_7962 |
| 41T 5 | 13810547 | 13810547 | DNAH5 | intronic | 5p15.2 | . | bnd_52 | transl_inter_7867 |
| 41T 5 | 32001704 | 32001704 | PDZD2 | intronic | 5p13.3 | . | bnd_54 | transl_inter_5337 |
| 41T 5 | 36389652 | 36389652 | . | intergenic | 5p13.2 | . | bnd_11 | transl_inter_8263 |
| 41T 5 | 125634089 | 125634089 | . | intergenic | 5q23.2 | Score=2698;Name="3779567:BLACKJACK(DNA)" | bnd_29 | transl_inter_3449 |
| 41T 5 | 154726803 | 154726803 | . | intergenic | 5q33.2 | . | bnd_56 | transl_inter_6365 |
| 41T 6 | 9504561 | 9504561 | . | intergenic | 6p24.3 | . | bnd_45 | transl_inter_7866 |
| 41T 6 | 142557171 | 142557171 | . | intergenic | 6q24.1 | . | bnd_43 | transl_inter_7973 |
| 41T 6 | 148053830 | 148053830 | . | intergenic | 6q24.3 | . | bnd_41 | transl_inter_7983 |
| 41T 6 | 152254912 | 152254912 | ESR1 | intronic | 6q25.1 | Score=768;Name="4144776:L2a(LINE)" | bnd_13 | transl_inter_6346 |
| 41T 7 | 3860390 | 3860390 | SDK1 | intronic | 7p22.2 | . | bnd_53 | transl_inter_5337 |
| 41T 7 | 26872919 | 26872919 | SKAP2 | intronic | 7p15.2 | Score=2029;Name="4272464:AluY(SINE)" | bnd_51 | transl_inter_7867 |
| 41T 7 | 30015663 | 30015663 | SCRN1 | intronic | 7p14.3 | . | bnd_55 | transl_inter_6365 |
| 41T 7 | 37346333 | 37346333 | ELMO1 | intronic | 7p14.1 | . | bnd_33 | transl_inter_6450 |
| 41T 7 | 41791288 | 41791288 | INHBA-AS1 | ncRNA_intronic | 7p14.1 | Score=919;Name="4298568:LTR29(LTR)" | bnd_25 | transl_inter_5294 |
| 41T 7 | 125124866 | 125124866 | . | intergenic | 7q31.33 | . | bnd_23 | transl_inter_8460 |
| 41T 8 | 115729175 | 115729175 | . | intergenic | 8q23.3 | . | bnd_47 | transl_inter_5288 |
| 41T 10 | 14170142 | 14170142 | FRMD4A | intronic | 10p13 | Score=1806;Name="457067:AluJb(SINE)" | bnd_8 | transl_inter_7968 |
| 41T 10 | 19559898 | 19559898 | MALRD1 | intronic | 10p12.31 | . | bnd_10 | transl_inter_8179 |
| 41T 10 | 50490447 | 50490447 | . | intergenic | 10q11.23 | . | bnd_12 | transl_inter_8263 |
| 41T 10 | 63340639 | 63340639 | . | intergenic | 10q21.2 | . | bnd_14 | transl_inter_6346 |
| 41T 10 | 108162515 | 108162515 | . | intergenic | 10q25.1 | Score=1466;Name="625328:L2b(LINE)" | bnd_16 | transl_inter_8090 |
| 41T 10 | 129776455 | 129776455 | PTPRE | intronic | 10q26.2 | . | bnd_18 | transl_inter_1676 |
| 41T 11 | 71211451 | 71211451 | NADSYN1 | intronic | 11q13.4 | Score=716;Name="793953:MER20(DNA)" | bnd_20 | transl_inter_5309 |
| 41T 12 | 21528089 | 21528089 | IAPP,SLCO1A2 | intronic | 12p12.1 | . | bnd_22 | transl_inter_6429 |
| 41T 12 | 91354734 | 91354734 | . | intergenic | 12q21.33 | Score=442;Name="1066074:ORSL(DNA)" | bnd_24 | transl_inter_8460 |
| 41T 12 | 108705902 | 108705902 | CMKLR1 | intronic | 12q23.3 | Score=436;Name="1101602:MIRb(SINE)" | bnd_26 | transl_inter_5294 |
| 41T 13 | 29663123 | 29663123 | MTUS2 | intronic | 13q12.3 | Score=6804;Name="1180099:L1PA15(LINE)" | bnd_28 | transl_inter_5334 |
| 41T 13 | 71061672 | 71061672 | . | intergenic | 13q21.33 | . | bnd_30 | transl_inter_3449 |
| 41T 14 | 22191721 | 22191721 | . | intergenic | 14q11.2 | . | bnd_32 | transl_inter_8003 |
| 41T 16 | 12311942 | 12311942 | SNX29 | intronic | 16p13.13 | . | bnd_5 | transl_inter_6357 |
| 41T 17 | 19969039 | 19969039 | SPECC1 | intronic | 17p11.2 | Score=4229;Name="1847618:MER21A(LTR)" | bnd_1 | transl_inter_8063 |
| 41T 18 | 2702764 | 2702764 | SMCHD1 | intronic | 18p11.32 | . | bnd_34 | transl_inter_6450 |
| 41T 19 | 17591222 | 17591222 | SLC27A1 | intronic | 19p13.11 | Score=1670;Name="2144767:AluJb(SINE)" | bnd_17 | transl_inter_1676 |
| 41T 19 | 39065788 | 39065788 | RYR1 | intronic | 19q13.2 | . | bnd_36 | transl_inter_7962 |
| 41T 20 | 38706385 | 38706385 | . | intergenic | 20q12 | Score=1492;Name="2719807:L1ME1(LINE)" | bnd_15 | transl_inter_8090 |
| 41T 21 | 31337881 | 31337881 | . | intergenic | 21q21.3 | . | bnd_37 | transl_inter_5291 |
| 41T 21 | 45218877 | 45218877 | RRP1 | intronic | 21q22.3 | . | bnd_46 | transl_inter_7866 |
| 41T 22 | 30653763 | 30653763 | . | intergenic | 22q12.2 | . | bnd_19 | transl_inter_5309 |

| | | | | | | | | |
|---|---|---|---|---|---|---|---|---|
| 41T | X | 4237691 | 4237691 | . | intergenic | Xp22.33 | Score=1740;Name="4999776:AluJo(SINE)" | bnd_21 | transl_inter_6429 |
| 41T | X | 132071149 | 132071149 | HS6ST2 | intronic | Xq26.2 | Score=10885;Name="5213679:L1M4b(LINE)" | bnd_39 | transl_inter_6335 |
| 41T | X | 153726023 | 153726023 | . | intergenic | Xq28 | Score=2282;Name="5252121:AluSz(SINE)" | bnd_3 | transl_inter_8104 |
| 41T | Y | 2699963 | 2699963 | . | intergenic | Yp11.31 | Score=3134;Name="5261655:L1M4(LINE)" | bnd_49 | transl_inter_7972 |
| 42T | 1 | 67720664 | 67720664 | IL23R | intronic | 1p31.3 | . | bnd_84 | transl_inter_7265 |
| 42T | 1 | 74947605 | 74947605 | FPGT-TNNI3K,TNNI3K | intronic | 1p31.1 | . | bnd_2 | del_733 |
| 42T | 1 | 74957408 | 74957408 | FPGT-TNNI3K,TNNI3K | intronic | 1p31.1 | . | bnd_1 | del_733 |
| 42T | 1 | 75292718 | 75292718 | . | intergenic | 1p31.1 | Score=387;Name="162499:MIRb(SINE)" | bnd_86 | transl_inter_5662 |
| 42T | 1 | 112110062 | 112110062 | . | intergenic | 1p13.2 | . | bnd_88 | transl_inter_1286 |
| 42T | 1 | 112110120 | 112110120 | . | intergenic | 1p13.2 | . | bnd_90 | transl_inter_501 |
| 42T | 1 | 228612651 | 228612651 | HIST3H3 | exonic | 1q42.13 | . | bnd_92 | transl_inter_1185 |
| 42T | 2 | 7092185 | 7092185 | RNF144A | intronic | 2p25.2 | Score=3003;Name="2244289:L1ME3F(LINE)" | bnd_8 | del_1177 |
| 42T | 2 | 7133768 | 7133768 | RNF144A | intronic | 2p25.1 | . | bnd_7 | del_1177 |
| 42T | 2 | 72434970 | 72434970 | EXOC6B | intronic | 2p13.2 | . | bnd_10 | del_2525 |
| 42T | 2 | 72920704 | 72920704 | EXOC6B | intronic | 2p13.2 | . | bnd_9 | del_2525 |
| 42T | 2 | 85967492 | 85967492 | . | intergenic | 2p11.2 | Score=1059;Name="2387517:L1MC4a(LINE)" | bnd_112 | transl_inter_5588 |
| 42T | 2 | 187229601 | 187229601 | . | intergenic | 2q32.1 | . | bnd_12 | del_2880 |
| 42T | 2 | 187355733 | 187355733 | ZC3H15 | intronic | 2q32.1 | . | bnd_11 | del_2880 |
| 42T | 3 | 84811627 | 84811627 | LINC00971 | ncRNA_intronic | 3p12.1 | Score=22071;Name="3063671:L1PA7(LINE)" | bnd_114 | transl_inter_5573 |
| 42T | 3 | 185039108 | 185039108 | MAP3K13 | intronic | 3q27.2 | . | bnd_20 | del_1032 |
| 42T | 3 | 185109067 | 185109067 | MAP3K13 | intronic | 3q27.2 | Score=2149;Name="3230656:L1MB3(LINE)" | bnd_19 | del_1032 |
| 42T | 4 | 11327807 | 11327807 | . | intergenic | 4p15.33 | . | bnd_113 | transl_inter_5573 |
| 42T | 4 | 104285777 | 104285777 | . | intergenic | 4q24 | . | bnd_68 | transl_intra_418 |
| 42T | 4 | 104493868 | 104493868 | . | intergenic | 4q24 | . | bnd_22 | del_78 |
| 42T | 4 | 104494779 | 104494779 | . | intergenic | 4q24 | . | bnd_21 | del_78 |
| 42T | 4 | 105019187 | 105019187 | . | intergenic | 4q24 | Score=20347;Name="3436323:L1PA15(LINE)" | bnd_91 | transl_inter_1185 |
| 42T | 4 | 105051375 | 105051375 | . | intergenic | 4q24 | Score=1977;Name="3436364:MLT1B(LTR)" | bnd_50 | del_1123_0 |
| 42T | 4 | 106604780 | 106604780 | INTS12 | intronic | 4q24 | Score=500;Name="3438806:MARNA(DNA)" | bnd_36 | del_ins_1078 |
| 42T | 4 | 106759441 | 106759441 | GSTCD | intronic | 4q24 | Score=845;Name="3439065:LTR40b(LTR)" | bnd_35 | del_ins_1078 |
| 42T | 4 | 107485907 | 107485907 | . | intergenic | 4q24 | Score=508;Name="3440132:L2c(LINE)" | bnd_67 | transl_intra_418 |
| 42T | 4 | 107761830 | 107761830 | . | intergenic | 4q25 | Score=2595;Name="3440522:L1MA9(LINE)" | bnd_60 | transl_intra_1198_0 |
| 42T | 4 | 107761928 | 107761928 | . | intergenic | 4q25 | Score=2595;Name="3440522:L1MA9(LINE)" | bnd_49 | del_1123_0 |
| 42T | 4 | 107762731 | 107762731 | . | intergenic | 4q25 | . | bnd_59 | transl_intra_1198_0 |
| 42T | 4 | 134743415 | 134743415 | . | intergenic | 4q28.3 | Score=10739;Name="3484668:L1P2(LINE)" | bnd_116 | transl_inter_6987 |
| 42T | 4 | 143999019 | 143999019 | . | intergenic | 4q31.21 | Score=1101;Name="3499891:L1ME3D(LINE)" | bnd_45 | del_insou_1961_0/314_0 |
| 42T | 4 | 144003244 | 144003244 | . | intergenic | 4q31.21 | . | bnd_24 | del_376 |

| | | | | | | | | |
|---|---|---|---|---|---|---|---|---|
| 42T 4 | 144005143 | 144005143 | . | intergenic | 4q31.21 | . | bnd_76 | tandem_dup_362 |
| 42T 4 | 144007383 | 144007383 | . | intergenic | 4q31.21 | . | bnd_43 | del_insou_1961_0/314_0 |
| 42T 4 | 144010041 | 144010041 | . | intergenic | 4q31.21 | . | bnd_44 | del_insou_1961_0/314_0 |
| 42T 4 | 144012808 | 144012808 | . | intergenic | 4q31.21 | Score=8148;Name ="3499924:MER4-int(LTR)" | bnd_46 | del_insou_1961_0/314_0 |
| 42T 4 | 144044345 | 144044345 | . | intergenic | 4q31.21 | Score=4595;Name ="3499976:ERV3-16A3_I-int(LTR)" | bnd_75 | tandem_dup_362 |
| 42T 4 | 145165999 | 145165999 | . | intergenic | 4q31.21 | Score=6889;Name ="3501830:L1PA7 (LINE)" | bnd_23 | del_376 |
| 42T 4 | 145279842 | 145279842 | . | intergenic | 4q31.21 | . | bnd_62 | transl_intra_450_0 |
| 42T 4 | 145280603 | 145280603 | . | intergenic | 4q31.21 | Score=2195;Name ="3502002:AluSz( SINE)" | bnd_61 | transl_intra_450_0 |
| 42T 4 | 145296461 | 145296461 | . | intergenic | 4q31.21 | . | bnd_109 | transl_inter_819 |
| 42T 4 | 170377295 | 170377295 | NEK1 | intronic | 4q33 | Score=5018;Name ="3544007:L1MA3 (LINE)" | bnd_118 | transl_inter_7245 |
| 42T 5 | 26532312 | 26532312 | . | intergenic | 5p14.1 | Score=2110;Name ="3620977:AluSx( SINE)" | bnd_26 | del_1398 |
| 42T 5 | 26544691 | 26544691 | . | intergenic | 5p14.1 | Score=1146;Name ="3621007:ERVL-E-int(LTR)" | bnd_25 | del_1398 |
| 42T 5 | 64400963 | 64400963 | . | intergenic | 5q12.3 | . | bnd_38 | del_ins_1811_0 |
| 42T 5 | 64401170 | 64401170 | . | intergenic | 5q12.3 | Score=653;Name= "3679103:L2c(LIN E)" | bnd_78 | tandem_dup_2190_0 |
| 42T 5 | 64402291 | 64402291 | . | intergenic | 5q12.3 | Score=308;Name= "3679106:L2c(LIN E)" | bnd_120 | transl_inter_3003 |
| 42T 5 | 105282256 | 105282256 | . | intergenic | 5q21.3 | Score=2062;Name ="3747617:AluSz6 (SINE)" | bnd_64 | transl_intra_1662_0 |
| 42T 5 | 105283116 | 105283116 | . | intergenic | 5q21.3 | . | bnd_63 | transl_intra_1662_0 |
| 42T 5 | 105286048 | 105286048 | . | intergenic | 5q21.3 | . | bnd_48 | transl_intra_741_0 |
| 42T 5 | 105286869 | 105286869 | . | intergenic | 5q21.3 | . | bnd_77 | tandem_dup_2190_0 |
| 42T 5 | 106315824 | 106315824 | LOC1024672 13 | ncRNA_intro nic | 5q21.3 | Score=15928;Nam e="3749244:HER VH-int(LTR)" | bnd_101 | transl_inter_7076 |
| 42T 5 | 110960147 | 110960147 | STARD4-AS1 | ncRNA_intro nic | 5q22.1 | . | bnd_37 | del_ins_1811_0 |
| 42T 5 | 110960428 | 110960428 | STARD4-AS1 | ncRNA_intro nic | 5q22.1 | . | bnd_122 | transl_inter_5890 |
| 42T 5 | 111448783 | 111448783 | . | intergenic | 5q22.1 | Score=503;Name= "3757093:L1ME3C (LINE)" | bnd_124 | transl_inter_7090 |
| 42T 5 | 117082736 | 117082736 | LOC1024672 24 | ncRNA_intro nic | 5q23.1 | Score=369;Name= "3766256:(TG)n(Si mple_repeat)" | bnd_47 | transl_intra_741_0 |
| 42T 5 | 163309808 | 163309808 | . | intergenic | 5q34 | Score=1783;Name ="3851415:L1ME3 A(LINE)" | bnd_99 | transl_inter_4640 |
| 42T 6 | 154340438 | 154340438 | OPRM1 | intronic | 6q25.2 | Score=4078;Name ="4148222:L1MC1 (LINE)" | bnd_105 | transl_inter_5641 |
| 42T 7 | 34565813 | 34565813 | NPSR1-AS1 | ncRNA_intro nic | 7p14.3 | Score=6154;Name ="4285901:L1MA3 (LINE)" | bnd_40 | del_ins_1892 |
| 42T 7 | 34614186 | 34614186 | NPSR1-AS1 | ncRNA_intro nic | 7p14.3 | Score=2173;Name ="4285964:AluSz( SINE)" | bnd_39 | del_ins_1892 |
| 42T 7 | 75393632 | 75393632 | . | intergenic | 7q11.23 | . | bnd_95 | transl_inter_7422 |
| 42T 8 | 5629947 | 5629947 | . | intergenic | 8p23.2 | Score=1472;Name ="4513746:L1ME3 (LINE)" | bnd_107 | transl_inter_7008 |
| 42T 8 | 32491909 | 32491909 | NRG1 | intronic | 8p12 | . | bnd_28 | del_904 |
| 42T 8 | 32529841 | 32529841 | NRG1 | intronic | 8p12 | Score=2409;Name ="4562861:AluSg( SINE)" | bnd_27 | del_904 |
| 42T 8 | 33888159 | 33888159 | . | intergenic | 8p12 | Score=649;Name= "4565500:MIR(SIN E)" | bnd_121 | transl_inter_5890 |

| | | | | | | | | | |
|---|---|---|---|---|---|---|---|---|---|
| 42T | 8 | 34235670 | 34235670 | . | intergenic | 8p12 | Score=2935;Name="4566052:L2a(LINE)" | bnd_119 | transl_inter_3003 |
| 42T | 8 | 56031730 | 56031730 | XKR4 | intronic | 8q12.1 | Score=315;Name="4597748:L2c(LINE)" | bnd_30 | del_465 |
| 42T | 8 | 58385336 | 58385336 | . | intergenic | 8q12.1 | Score=1155;Name="4601764:MLT1A0-int(LTR)" | bnd_29 | del_465 |
| 42T | 8 | 60740963 | 60740963 | . | intergenic | 8q12.1 | Score=6361;Name="4605391:L1M4b(LINE)" | bnd_52 | del_602_0 |
| 42T | 8 | 65713982 | 65713982 | . | intergenic | 8q12.3 | . | bnd_80 | tandem_dup_643 |
| 42T | 8 | 65718514 | 65718514 | . | intergenic | 8q12.3 | . | bnd_32 | del_206 |
| 42T | 8 | 65729194 | 65729194 | . | intergenic | 8q12.3 | Score=1687;Name="4613272:Charlie2b(DNA)" | bnd_31 | del_206 |
| 42T | 8 | 68296350 | 68296350 | . | intergenic | 8q13.2 | . | bnd_82 | tandem_dup_118_0 |
| 42T | 8 | 68300408 | 68300408 | . | intergenic | 8q13.2 | . | bnd_79 | tandem_dup_643 |
| 42T | 8 | 73271153 | 73271153 | . | intergenic | 8q13.3 | Score=263;Name="4626736:LTR85b(LTR)" | bnd_81 | tandem_dup_118_0 |
| 42T | 8 | 74180985 | 74180985 | . | intergenic | 8q21.11 | Score=1785;Name="4628356:L1MC5(LINE)" | bnd_51 | del_602_0 |
| 42T | 8 | 76829666 | 76829666 | . | intergenic | 8q21.11 | Score=9916;Name="4633123:L1MA4A(LINE)" | bnd_42 | del_ins_1504 |
| 42T | 8 | 76834541 | 76834541 | . | intergenic | 8q21.11 | . | bnd_41 | del_ins_1504 |
| 42T | 8 | 93428157 | 93428157 | . | intergenic | 8q22.1 | Score=2429;Name="4660136:LTR7(LTR)" | bnd_111 | transl_inter_5588 |
| 42T | 8 | 140924033 | 140924033 | TRAPPC9 | intronic | 8q24.3 | . | bnd_34 | del_4307 |
| 42T | 8 | 141025549 | 141025549 | TRAPPC9 | intronic | 8q24.3 | . | bnd_33 | del_4307 |
| 42T | 9 | 14110498 | 14110498 | NFIB | intronic | 9p23 | . | bnd_89 | transl_inter_501 |
| 42T | 9 | 14160160 | 14160160 | NFIB | intronic | 9p23 | . | bnd_87 | transl_inter_1286 |
| 42T | 9 | 86064830 | 86064830 | FRMD3 | intronic | 9q21.32 | . | bnd_56 | del_invers_2891_0/1091_0 |
| 42T | 9 | 86090253 | 86090253 | FRMD3 | intronic | 9q21.32 | Score=1909;Name="4873039:L1MEg(LINE)" | bnd_57 | del_invers_2891_0/1091_0 |
| 42T | 9 | 86106535 | 86106535 | FRMD3 | intronic | 9q21.32 | . | bnd_55 | del_invers_2891_0/1091_0 |
| 42T | 9 | 86108192 | 86108192 | FRMD3 | intronic | 9q21.32 | Score=584;Name="4873075:LTR78B(LTR)" | bnd_58 | del_invers_2891_0/1091_0 |
| 42T | 9 | 89534469 | 89534469 | . | intergenic | 9q21.33 | Score=1358;Name="4879701:L1MC3(LINE)" | bnd_123 | transl_inter_7090 |
| 42T | 10 | 23423126 | 23423126 | . | intergenic | 10p12.2 | Score=2442;Name="474317:L1MC5(LINE)" | bnd_94 | transl_inter_7087 |
| 42T | 11 | 124024571 | 124024571 | . | intergenic | 11q24.2 | . | bnd_96 | transl_inter_7422 |
| 42T | 12 | 96599171 | 96599171 | ELK3 | intronic | 12q23.1 | . | bnd_98 | transl_inter_7165 |
| 42T | 12 | 102678710 | 102678710 | . | intergenic | 12q23.2 | . | bnd_100 | transl_inter_4640 |
| 42T | 13 | 29980868 | 29980868 | MTUS2 | intronic | 13q12.3 | Score=1904;Name="1180582:L1ME3C(LINE)" | bnd_102 | transl_inter_7076 |
| 42T | 13 | 48954766 | 48954766 | RB1 | intronic | 13q14.2 | . | bnd_4 | del_5401 |
| 42T | 13 | 49107924 | 49107924 | . | upstream | 13q14.2 | . | bnd_3 | del_5401 |
| 42T | 13 | 77614357 | 77614357 | . | intergenic | 13q22.3 | . | bnd_104 | transl_inter_1565 |
| 42T | 14 | 33233420 | 33233420 | AKAP6 | intronic | 14q12 | Score=423;Name="1345707:L2a(LINE)" | bnd_103 | transl_inter_1565 |
| 42T | 14 | 69682012 | 69682012 | EXD2 | intronic | 14q24.1 | . | bnd_106 | transl_inter_5641 |
| 42T | 14 | 79122901 | 79122901 | NRXN3 | intronic | 14q24.3 | Score=504;Name="1429674:L2c(LINE)" | bnd_108 | transl_inter_7008 |
| 42T | 16 | 68786846 | 68786846 | CDH1 | intronic | 16q22.1 | . | bnd_6 | del_1887 |
| 42T | 16 | 68831760 | 68831760 | CDH1 | intronic | 16q22.1 | Score=2363;Name="1761095:AluSz(SINE)" | bnd_5 | del_1887 |
| 42T | 16 | 87804016 | 87804016 | . | intergenic | 16q24.2 | . | bnd_93 | transl_inter_7087 |
| 42T | 18 | 27917846 | 27917846 | . | intergenic | 18q12.1 | Score=459;Name="2016129:UCON16(Unknown)" | bnd_85 | transl_inter_5662 |
| 42T | 19 | 1222935 | 1222935 | STK11 | intronic | 19p13.3 | . | bnd_54 | transl_intra_2499_0 |

| | | | | | | | | | |
|---|---|---|---|---|---|---|---|---|---|
| 42T | 19 | 4190325 | 4190325 | ANKRD24 | intronic | 19p13.3 | Score=2304;Name="2108419:AluY(SINE)" | bnd_66 | transl_intra_1762 |
| 42T | 19 | 4190823 | 4190823 | ANKRD24 | intronic | 19p13.3 | . | bnd_65 | transl_intra_1762 |
| 42T | 19 | 4469853 | 4469853 | . | intergenic | 19p13.3 | Score=2263;Name="2109186:AluSx1(SINE)" | bnd_110 | transl_inter_819 |
| 42T | 19 | 19839375 | 19839375 | ZNF14 | intronic | 19p13.11 | . | bnd_97 | transl_inter_7165 |
| 42T | 19 | 1228865 | 1228865 | . | downstream | 19p13.3 | . | bnd_53 | transl_intra_2499_0 |
| 42T | 20 | 14910318 | 14910318 | MACROD2 | intronic | 20p12.1 | Score=3091;Name="2677563:MER21C(LTR)" | bnd_72 | tandem_dup_1249 |
| 42T | 20 | 14951421 | 14951421 | MACROD2 | intronic | 20p12.1 | Score=1390;Name="2677646:AluJo(SINE)" | bnd_14 | del_629 |
| 42T | 20 | 14973010 | 14973010 | MACROD2 | intronic | 20p12.1 | . | bnd_16 | del_1058 |
| 42T | 20 | 15005406 | 15005406 | MACROD2 | intronic | 20p12.1 | . | bnd_15 | del_1058 |
| 42T | 20 | 15448407 | 15448407 | MACROD2 | intronic | 20p12.1 | Score=210;Name="2678521:L2a(LINE)" | bnd_13 | del_629 |
| 42T | 20 | 15506385 | 15506385 | MACROD2 | intronic | 20p12.1 | . | bnd_71 | tandem_dup_1249 |
| 42T | 20 | 46254570 | 46254570 | NCOA3 | intronic | 20q13.12 | . | bnd_83 | transl_inter_7265 |
| 42T | 21 | 32070633 | 32070633 | . | intergenic | 21q22.11 | . | bnd_74 | tandem_dup_3782 |
| 42T | 21 | 32110524 | 32110524 | . | intergenic | 21q22.11 | Score=285;Name="2804477:MIRb(SINE)" | bnd_73 | tandem_dup_3782 |
| 42T | 22 | 31808959 | 31808959 | DRG1 | intronic | 22q12.2 | Score=794;Name="2865696:HAL1(LINE)" | bnd_18 | del_1879 |
| 42T | 22 | 31810149 | 31810149 | DRG1 | intronic | 22q12.2 | Score=980;Name="2865700:L2(LINE)" | bnd_17 | del_1879 |
| 42T | X | 97889909 | 97889909 | . | intergenic | Xq21.33 | Score=13483;Name="5155461:L1MA2(LINE)" | bnd_115 | transl_inter_6987 |
| 42T | Y | 8487827 | 8487827 | . | intergenic | Yp11.2 | . | bnd_70 | transl_intra_926 |
| 42T | Y | 8490767 | 8490767 | . | intergenic | Yp11.2 | . | bnd_69 | transl_intra_926 |
| 42T | Y | 22018013 | 22018013 | . | intergenic | Yq11.222 | Score=1940;Name="5287437:L1PBa(LINE)" | bnd_117 | transl_inter_7245 |
| 43T | 1 | 34296571 | 34296571 | CSMD2 | intronic | 1p35.1 | Score=27363;Name="75782:L1PA3(LINE)" | bnd_24 | transl_inter_1731 |
| 43T | 3 | 40868886 | 40868886 | . | intergenic | 3p22.1 | . | bnd_26 | transl_inter_1740 |
| 43T | 3 | 144820524 | 144820524 | . | intergenic | 3q24 | Score=1901;Name="3160838:L1ME3B(LINE)" | bnd_28 | transl_inter_1486 |
| 43T | 3 | 174488957 | 174488957 | . | intergenic | 3q26.31 | . | bnd_30 | transl_inter_6599 |
| 43T | 4 | 64356653 | 64356653 | . | intergenic | 4q13.1 | Score=6098;Name="3368425:L1M4c(LINE)" | bnd_10 | del_ins_810 |
| 43T | 4 | 64357334 | 64357334 | . | intergenic | 4q13.1 | . | bnd_9 | del_ins_810 |
| 43T | 4 | 150593683 | 150593683 | . | intergenic | 4q31.23 | Score=11280;Name="3510475:L1P1(LINE)" | bnd_29 | transl_inter_6599 |
| 43T | 5 | 38593098 | 38593098 | LIFR-AS1,LIFR | ncRNA_intronic | 5p13.1 | Score=2269;Name="3642393:AluSg(SINE)" | bnd_23 | transl_inter_1731 |
| 43T | 6 | 62869327 | 62869327 | KHDRBS2 | intronic | 6q11.1 | . | bnd_12 | del_insod_1369_0/624_0 |
| 43T | 6 | 63342781 | 63342781 | . | intergenic | 6q11.2 | Score=10897;Name="3996978:L1MB2(LINE)" | bnd_14 | del_insod_1369_0/624_0 |
| 43T | 6 | 68536265 | 68536265 | . | intergenic | 6q12 | Score=8642;Name="4005390:LTR12C(LTR)" | bnd_18 | transl_intra_1085_0 |
| 43T | 6 | 68543451 | 68543451 | . | intergenic | 6q12 | . | bnd_16 | transl_intra_790_0 |
| 43T | 6 | 69868690 | 69868690 | BAI3 | intronic | 6q12 | . | bnd_17 | transl_intra_1085_0 |
| 43T | 6 | 69876944 | 69876944 | BAI3 | intronic | 6q12 | Score=340;Name="4007539:MamRep605(Unknown)" | bnd_8 | del_1037 |
| 43T | 6 | 72322452 | 72322452 | . | intergenic | 6q13 | . | bnd_13 | del_insod_1369_0/624_0 |
| 43T | 6 | 73188432 | 73188432 | . | intergenic | 6q13 | Score=210;Name="4012624:L2c(LINE)" | bnd_20 | tandem_dup_482_0 |
| 43T | 6 | 74658936 | 74658936 | . | intergenic | 6q13 | . | bnd_15 | transl_intra_790_0 |
| 43T | 6 | 74676758 | 74676758 | . | intergenic | 6q13 | . | bnd_19 | tandem_dup_482_0 |

| | | | | | | | | | |
|---|---|---|---|---|---|---|---|---|---|
| 43T | 6 | 75417608 | 75417608 | . | intergenic | 6q13 | . | bnd_7 | del_1037 |
| 43T | 6 | 76561340 | 76561340 | MYO6 | intronic | 6q14.1 | Score=1594;Name="4018592:AluJb(SINE)" | bnd_11 | del_insod_1369_0/624_0 |
| 43T | 7 | 121502771 | 121502771 | . | intergenic | 7q31.32 | . | bnd_25 | transl_inter_1740 |
| 43T | 8 | 36600336 | 36600336 | . | intergenic | 8p11.23 | . | bnd_27 | transl_inter_1486 |
| 43T | 9 | 94784817 | 94784817 | . | intergenic | 9q22.31 | Score=2804;Name="4888634:L1MB8(LINE)" | bnd_22 | tandem_dup_3100 |
| 43T | 9 | 94785355 | 94785355 | . | intergenic | 9q22.31 | Score=2960;Name="4888636:L1MB8(LINE)" | bnd_21 | tandem_dup_3100 |
| 43T | 13 | 59721733 | 59721733 | . | intergenic | 13q21.2 | Score=1995;Name="1232391:L1PA8(LINE)" | bnd_2 | del_3206 |
| 43T | 13 | 59732643 | 59732643 | . | intergenic | 13q21.2 | Score=2327;Name="1232407:L1MC4(LINE)" | bnd_1 | del_3206 |
| 43T | 16 | 22086808 | 22086808 | C16orf52 | exonic | 16p12.2 | . | bnd_3 | del_6269 |
| 43T | 16 | 22019728 | 22019728 | C16orf52 | exonic | 16p12.2 | . | bnd_4 | del_6269 |
| 43T | 20 | 51318376 | 51318376 | . | intergenic | 20q13.2 | . | bnd_6 | del_978 |
| 43T | 20 | 51336751 | 51336751 | . | intergenic | 20q13.2 | . | bnd_5 | del_978 |
| 44T | 1 | 228653085 | 228653085 | . | intergenic | 1q42.13 | . | bnd_1 | del_1258 |
| 44T | 1 | 228645713 | 228645713 | . | upstream | 1q42.13 | . | bnd_2 | del_1258 |
| 44T | 2 | 154873036 | 154873036 | GALNT13 | intronic | 2q23.3 | Score=2275;Name="2497595:AluSg(SINE)" | bnd_34 | transl_inter_1735 |
| 44T | 4 | 306324 | 306324 | . | intergenic | 4p16.3 | Score=2299;Name="3256837:AluY(SINE)" | bnd_33 | transl_inter_1735 |
| 44T | 6 | 7160744 | 7160744 | RREB1 | intronic | 6p24.3 | . | bnd_24 | tandem_dup_900 |
| 44T | 6 | 7312817 | 7312817 | SSR1 | intronic | 6p24.3 | . | bnd_23 | tandem_dup_900 |
| 44T | 8 | 116814461 | 116814461 | . | intergenic | 8q23.3 | . | bnd_31 | transl_inter_933 |
| 44T | 9 | 8313693 | 8313693 | . | downstream | 9p24.1 | Score=325;Name="4773403:L2a(LINE)" | bnd_20 | transl_intra_1381 |
| 44T | 9 | 11797727 | 11797727 | . | intergenic | 9p23 | Score=2085;Name="4778815:AluSz(SINE)" | bnd_13 | del_inssu_1719_0/1286_0 |
| 44T | 9 | 11905128 | 11905128 | . | intergenic | 9p23 | Score=845;Name="4778994:L1MC4(LINE)" | bnd_26 | tandem_dup_693_0 |
| 44T | 9 | 11947687 | 11947687 | . | intergenic | 9p23 | Score=1844;Name="4779075:MER67D(LTR)" | bnd_22 | transl_intra_860_0 |
| 44T | 9 | 11963333 | 11963333 | . | intergenic | 9p23 | Score=590;Name="4779106:L1MDb(LINE)" | bnd_29 | transl_inter_679 |
| 44T | 9 | 11998771 | 11998771 | . | intergenic | 9p23 | Score=9981;Name="4779158:L1M2a(LINE)" | bnd_15 | del_inssu_1719_0/1286_0 |
| 44T | 9 | 12330269 | 12330269 | . | intergenic | 9p23 | Score=9023;Name="4779714:L1MA4(LINE)" | bnd_25 | tandem_dup_693_0 |
| 44T | 9 | 16244802 | 16244802 | C9orf92 | intronic | 9p22.3 | Score=800;Name="4786228:MIRb(SINE)" | bnd_19 | transl_intra_1381 |
| 44T | 9 | 16251184 | 16251184 | C9orf92 | intronic | 9p22.3 | . | bnd_18 | transl_intra_549_0 |
| 44T | 9 | 16383820 | 16383820 | . | intergenic | 9p22.3 | Score=5469;Name="4786467:L1MA5A(LINE)" | bnd_21 | transl_intra_860_0 |
| 44T | 9 | 16410649 | 16410649 | BNC2 | UTR3 | 9p22.3 | . | bnd_17 | transl_intra_549_0 |
| 44T | 9 | 29382844 | 29382844 | . | intergenic | 9p21.1 | Score=14092;Name="4807565:Harlequin-int(LTR)" | bnd_14 | del_inssu_1719_0/1286_0 |
| 44T | 9 | 29869732 | 29869732 | . | intergenic | 9p21.1 | Score=6838;Name="4808335:L1PA7(LINE)" | bnd_16 | del_inssu_1719_0/1286_0 |
| 44T | 10 | 15499143 | 15499143 | . | intergenic | 10p13 | . | bnd_4 | del_309 |
| 44T | 10 | 15517689 | 15517689 | . | intergenic | 10p13 | Score=2391;Name="459739:L1MA3(LINE)" | bnd_3 | del_309 |
| 44T | 11 | 70966544 | 70966544 | . | intergenic | 11q13.4 | . | bnd_6 | del_896 |
| 44T | 11 | 70967906 | 70967906 | . | intergenic | 11q13.4 | Score=1204;Name="793449:MER44B(DNA)" | bnd_5 | del_896 |

| | | | | | | | | |
|---|---|---|---|---|---|---|---|---|
| 44T 12 | 10216675 | 10216675 | CLEC9A | intronic | 12p13.2 | . | bnd_12 | del_959_0 |
| 44T 12 | 24098568 | 24098568 | SOX5 | intronic | 12p12.1 | . | bnd_11 | del_959_0 |
| 44T 13 | 20970739 | 20970739 | . | intergenic | 13q12.11 | . | bnd_28 | transl_inter_683 |
| 44T 13 | 91034331 | 91034331 | . | intergenic | 13q31.3 | . | bnd_30 | transl_inter_679 |
| 44T 13 | 91177700 | 91177700 | . | intergenic | 13q31.3 | . | bnd_8 | del_543 |
| 44T 13 | 91192828 | 91192828 | . | intergenic | 13q31.3 | Score=17591;Name="1284216:L1PA15(LINE)" | bnd_7 | del_543 |
| 44T 17 | 14805652 | 14805652 | . | intergenic | 17p12 | . | bnd_32 | transl_inter_933 |
| 44T 17 | 14831163 | 14831163 | . | intergenic | 17p12 | Score=658;Name="1836379:HAL1(LINE)" | bnd_10 | del_717 |
| 44T 17 | 14837002 | 14837002 | . | intergenic | 17p12 | Score=283;Name="1836391:LTR16A1(LTR)" | bnd_9 | del_717 |
| 44T 21 | 20813424 | 20813424 | . | intergenic | 21q21.1 | . | bnd_27 | transl_inter_683 |
| 45T 3 | 124643446 | 124643446 | MUC13 | intronic | 3q21.2 | Score=234;Name="3123196:L1MC4a(LINE)" | bnd_4 | transl_intra_3092_0 |
| 45T 3 | 154021089 | 154021089 | DHX36 | intronic | 3q25.2 | . | bnd_6 | transl_intra_2545 |
| 45T 3 | 154021695 | 154021695 | DHX36 | intronic | 3q25.2 | . | bnd_5 | transl_intra_2545 |
| 45T 3 | 193453082 | 193453082 | . | intergenic | 3q29 | Score=542;Name="3246410:MIR(SINE)" | bnd_7 | transl_inter_373 |
| 45T 3 | 167813605 | 167813605 | . | upstream | 3q26.2 | . | bnd_3 | transl_intra_3092_0 |
| 45T 6 | 12668433 | 12668433 | . | intergenic | 6p24.1 | Score=16198;Name="3909174:HERVH-int(LTR)" | bnd_10 | transl_inter_6559 |
| 45T 8 | 146184708 | 146184708 | . | intergenic | 8q24.3 | Score=28279;Name="4758632:HERVH-int(LTR)" | bnd_9 | transl_inter_6559 |
| 45T 14 | 26689316 | 26689316 | . | intergenic | 14q12 | . | bnd_2 | del_7175 |
| 45T 14 | 26691202 | 26691202 | . | intergenic | 14q12 | . | bnd_1 | del_7175 |
| 45T 17 | 41833054 | 41833054 | SOST | exonic | 17q21.31 | . | bnd_8 | transl_inter_373 |
| 46T 1 | 3330404 | 3330404 | PRDM16 | intronic | 1p36.32 | . | bnd_2 | del_637 |
| 46T 1 | 4091832 | 4091832 | . | intergenic | 1p36.32 | . | bnd_1 | del_637 |
| 46T 1 | 10630300 | 10630300 | PEX14 | intronic | 1p36.22 | Score=2403;Name="18896:HAL1(LINE)" | bnd_14 | transl_inter_6768 |
| 46T 1 | 44706808 | 44706808 | ERI3-IT1,ERI3 | ncRNA_intronic | 1p34.1 | . | bnd_16 | transl_inter_6796 |
| 46T 1 | 59118509 | 59118509 | . | intergenic | 1p32.1 | . | bnd_4 | del_1389 |
| 46T 1 | 59128530 | 59128530 | MYSM1 | intronic | 1p32.1 | . | bnd_3 | del_1389 |
| 46T 1 | 60656239 | 60656239 | . | intergenic | 1p32.1 | Score=2443;Name="135102:MSTB(LTR)" | bnd_6 | del_2539_0 |
| 46T 1 | 101729439 | 101729439 | . | intergenic | 1p21.2 | Score=1996;Name="208562:AluJr4(SINE)" | bnd_18 | transl_inter_6712 |
| 46T 1 | 119262585 | 119262585 | . | intergenic | 1p12 | Score=1719;Name="239460:AluSz6(SINE)" | bnd_20 | transl_inter_6765 |
| 46T 1 | 164216175 | 164216175 | . | intergenic | 1q23.3 | Score=657;Name="280723:L2a(LINE)" | bnd_22 | transl_inter_6799 |
| 46T 1 | 174862208 | 174862208 | RABGAP1L | intronic | 1q25.1 | . | bnd_24 | transl_inter_7112 |
| 46T 1 | 228803311 | 228803311 | . | intergenic | 1q42.13 | . | bnd_26 | transl_inter_1075 |
| 46T 1 | 228804184 | 228804184 | . | intergenic | 1q42.13 | Score=2366;Name="395032:MER21C(LTR)" | bnd_28 | transl_inter_2187 |
| 46T 1 | 228809899 | 228809899 | . | intergenic | 1q42.13 | Score=2436;Name="395042:L1ME3A(LINE)" | bnd_30 | transl_inter_3751 |
| 46T 1 | 235798886 | 235798886 | GNG4 | intronic | 1q42.3 | Score=912;Name="408049:MSTB1(LTR)" | bnd_5 | del_2539_0 |
| 46T 1 | 246003296 | 246003296 | SMYD3 | intronic | 1q44 | Score=862;Name="427432:HAL1(LINE)" | bnd_32 | transl_inter_160 |
| 46T 1 | 246383163 | 246383163 | SMYD3 | intronic | 1q44 | Score=5618;Name="428020:LTR1D(LTR)" | bnd_34 | transl_inter_902 |
| 46T 2 | 145068090 | 145068090 | GTDC1 | intronic | 2q22.3 | Score=1935;Name="2482309:L2a(LINE)" | bnd_47 | transl_inter_6599 |

| | | | | | | | | |
|---|---|---|---|---|---|---|---|---|
| 46T 3 | 110371571 | 110371571 | . | intergenic | 3q13.13 | Score=2354;Name="3099477:LTR1C(LTR)" | bnd_58 | transl_inter_4380 |
| 46T 4 | 8261421 | 8261421 | . | intergenic | 4p16.1 | . | bnd_43 | transl_inter_5398 |
| 46T 4 | 80579956 | 80579956 | . | intergenic | 4q21.21 | Score=9951;Name="3395399:L1PA16(LINE)" | bnd_25 | transl_inter_1075 |
| 46T 4 | 80581022 | 80581022 | . | intergenic | 4q21.21 | Score=11467;Name="3395401:THE1B-int(LTR)" | bnd_27 | transl_inter_2187 |
| 46T 4 | 80581707 | 80581707 | . | intergenic | 4q21.21 | Score=11467;Name="3395401:THE1B-int(LTR)" | bnd_35 | transl_inter_3260 |
| 46T 4 | 80608726 | 80608726 | . | intergenic | 4q21.21 | . | bnd_29 | transl_inter_3751 |
| 46T 4 | 86489583 | 86489583 | ARHGAP24 | intronic | 4q21.23 | . | bnd_49 | transl_inter_4367 |
| 46T 4 | 120786288 | 120786288 | . | intergenic | 4q26 | Score=937;Name="3461977:FRAM(SINE)" | bnd_17 | transl_inter_6712 |
| 46T 5 | 102861216 | 102861216 | . | intergenic | 5q21.2 | . | bnd_45 | transl_inter_6639 |
| 46T 6 | 10751433 | 10751433 | TMEM14B | exonic | 6p24.2 | . | bnd_39 | transl_inter_6691 |
| 46T 6 | 32126343 | 32126343 | PPT2,PPT2-EGFL8 | ncRNA_intronic | 6p21.32 | Score=1064;Name="3945900:L2a(LINE)" | bnd_15 | transl_inter_6796 |
| 46T 7 | 4401138 | 4401138 | . | intergenic | 7p22.2 | Score=594;Name="4233427:MLT1C(LTR)" | bnd_55 | transl_inter_5344 |
| 46T 7 | 101182681 | 101182681 | COL26A1 | intronic | 7q22.1 | Score=1942;Name="4404628:AluJr(SINE)" | bnd_19 | transl_inter_6765 |
| 46T 7 | 115006726 | 115006726 | . | intergenic | 7q31.2 | Score=2194;Name="4428887:Tigger3c(DNA)" | bnd_23 | transl_inter_7112 |
| 46T 8 | 58328456 | 58328456 | . | intergenic | 8q12.1 | Score=1135;Name="4601658:LTR43-int(LTR)" | bnd_57 | transl_inter_4380 |
| 46T 8 | 94905189 | 94905189 | . | intergenic | 8q22.1 | . | bnd_9 | del_inss_2737_0/4445_0 |
| 46T 8 | 94905887 | 94905887 | . | intergenic | 8q22.1 | . | bnd_11 | del_inss_2737_0/4445_0 |
| 46T 8 | 94911865 | 94911865 | . | intergenic | 8q22.1 | Score=2208;Name="4662690:AluSp(SINE)" | bnd_51 | transl_inter_3194 |
| 46T 8 | 137158500 | 137158500 | . | intergenic | 8q24.23 | . | bnd_41 | transl_inter_629 |
| 46T 8 | 142935096 | 142935096 | . | intergenic | 8q24.3 | Score=314;Name="4753358:HAL1-3A_ME(LINE)" | bnd_53 | transl_inter_5242 |
| 46T 10 | 6982464 | 6982464 | . | intergenic | 10p14 | Score=2255;Name="443819:MLT1B(LTR)" | bnd_36 | transl_inter_3260 |
| 46T 10 | 18556019 | 18556019 | CACNB2 | intronic | 10p12.33 | . | bnd_38 | transl_inter_4364 |
| 46T 10 | 49841499 | 49841499 | ARHGAP22 | intronic | 10q11.22 | . | bnd_8 | transl_intra_5784_0 |
| 46T 10 | 49844542 | 49844542 | ARHGAP22 | intronic | 10q11.22 | . | bnd_7 | transl_intra_5784_0 |
| 46T 12 | 69202855 | 69202855 | MDM2 | UTR5 | 12q15 | . | bnd_40 | transl_inter_6691 |
| 46T 13 | 61630960 | 61630960 | . | intergenic | 13q21.2 | . | bnd_42 | transl_inter_629 |
| 46T 13 | 92838441 | 92838441 | GPC5 | intronic | 13q31.3 | . | bnd_44 | transl_inter_5398 |
| 46T 13 | 96572627 | 96572627 | UGGT2 | intronic | 13q32.1 | Score=13036;Name="1293301:L1PA11(LINE)" | bnd_46 | transl_inter_6639 |
| 46T 13 | 99901094 | 99901094 | UBAC2,MIR548AN | ncRNA_intronic | 13q32.3 | . | bnd_48 | transl_inter_6599 |
| 46T 13 | 103659672 | 103659672 | . | intergenic | 13q33.1 | Score=390;Name="1305812:MIRc(SINE)" | bnd_33 | transl_inter_902 |
| 46T 13 | 104032410 | 104032410 | . | intergenic | 13q33.1 | . | bnd_31 | transl_inter_160 |
| 46T 17 | 265255 | 265255 | . | downstream | 17p13.3 | Score=331;Name="1805281:HAL1-3A_ME(LINE)" | bnd_50 | transl_inter_4367 |
| 46T 17 | 15032633 | 15032633 | . | intergenic | 17p12 | . | bnd_52 | transl_inter_3194 |
| 46T 17 | 15032901 | 15032901 | . | intergenic | 17p12 | . | bnd_10 | del_inss_2737_0/4445_0 |
| 46T 17 | 15034630 | 15034630 | . | intergenic | 17p12 | . | bnd_12 | del_inss_2737_0/4445_0 |
| 46T 18 | 19202733 | 19202733 | SNRPD1 | exonic | 18q11.2 | . | bnd_13 | transl_inter_6768 |
| 46T 19 | 13980699 | 13980699 | . | intergenic | 19p13.13 | . | bnd_54 | transl_inter_5242 |
| 46T 20 | 4402507 | 4402507 | . | intergenic | 20p13 | . | bnd_21 | transl_inter_6799 |
| 46T 22 | 31690553 | 31690553 | . | intergenic | 22q12.2 | . | bnd_56 | transl_inter_5344 |
| 46T 22 | 48330668 | 48330668 | . | intergenic | 22q13.31 | Score=1377;Name="2903693:AluJb(SINE)" | bnd_37 | transl_inter_4364 |
| 49T 1 | 40173650 | 40173650 | . | intergenic | 1p34.2 | . | bnd_4 | transl_inter_4083 |

| | | | | | | | | | |
|---|---|---|---|---|---|---|---|---|---|
| 49T | 1 | 152839574 | 152839574 | . | intergenic | 1q21.3 | Score=3400;Name="259336:L2a(LINE)" | bnd_6 | transl_inter_4195 |
| 49T | 2 | 212136249 | 212136249 | . | intergenic | 2q34 | Score=3693;Name="2593583:MER50(LTR)" | bnd_8 | transl_inter_4261 |
| 49T | 4 | 98980205 | 98980205 | STPG2 | intronic | 4q23 | Score=18590;Name="3426245:L1PA14(LINE)" | bnd_10 | transl_inter_3318 |
| 49T | 6 | 82246982 | 82246982 | . | intergenic | 6q14.1 | Score=11057;Name="4027297:THE1B-int(LTR)" | bnd_5 | transl_inter_4195 |
| 49T | 6 | 89442694 | 89442694 | RNGTT | intronic | 6q15 | Score=3304;Name="4038890:L1MB3(LINE)" | bnd_3 | transl_inter_4083 |
| 49T | 6 | 100362683 | 100362683 | . | intergenic | 6q16.2 | Score=668;Name="4056369:MIRb(SINE)" | bnd_9 | transl_inter_3318 |
| 49T | 7 | 27598822 | 27598822 | HIBADH | intronic | 7p15.2 | Score=2081;Name="4273600:MLT1B(LTR)" | bnd_7 | transl_inter_4261 |
| 49T | 8 | 67997406 | 67997406 | CSPP1 | intronic | 8q13.1 | Score=1053;Name="4617729:L1MB4(LINE)" | bnd_2 | del_3533 |
| 49T | 8 | 68021578 | 68021578 | CSPP1 | intronic | 8q13.2 | Score=2041;Name="4617781:MLT1E1A(LTR)" | bnd_1 | del_3533 |
| 50T | 1 | 15436894 | 15436894 | KAZN | intronic | 1p36.21 | Score=792;Name="28839:(TG)n(Simple_repeat)" | bnd_50 | transl_inter_6762 |
| 50T | 1 | 105981151 | 105981151 | . | intergenic | 1p21.1 | Score=2265;Name="215455:AluSx3(SINE)" | bnd_52 | transl_inter_1958 |
| 50T | 1 | 147019893 | 147019893 | BCL9 | intronic | 1q21.2 | . | bnd_54 | transl_inter_8434 |
| 50T | 1 | 231509640 | 231509640 | EGLN1 | intronic | 1q42.2 | . | bnd_56 | transl_inter_6671 |
| 50T | 2 | 79181374 | 79181374 | . | intergenic | 2p12 | Score=2147;Name="2375961:AluSx(SINE)" | bnd_76 | transl_inter_8459 |
| 50T | 2 | 163525268 | 163525268 | KCNH7 | intronic | 2q24.2 | Score=518;Name="2511359:L1MEg(LINE)" | bnd_71 | transl_inter_8347 |
| 50T | 3 | 16329871 | 16329871 | OXNAD1 | intronic | 3p25.1 | . | bnd_78 | transl_inter_8209 |
| 50T | 3 | 82735262 | 82735262 | . | intergenic | 3p12.2 | . | bnd_69 | transl_inter_2816 |
| 50T | 3 | 154932499 | 154932499 | . | intergenic | 3q25.2 | . | bnd_80 | transl_inter_8462 |
| 50T | 3 | 168508043 | 168508043 | EGFEM1P | ncRNA_intronic | 3q26.2 | . | bnd_82 | transl_inter_8109 |
| 50T | 3 | 173796794 | 173796794 | NLGN1 | intronic | 3q26.31 | . | bnd_84 | transl_inter_8933 |
| 50T | 4 | 8459466 | 8459466 | TRMT44 | intronic | 4p16.1 | Score=13684;Name="3272384:Tigger2(DNA)" | bnd_14 | del_864 |
| 50T | 4 | 8460212 | 8460212 | TRMT44 | intronic | 4p16.1 | Score=2162;Name="3272385:AluSx1(SINE)" | bnd_13 | del_864 |
| 50T | 4 | 44263529 | 44263529 | KCTD8 | intronic | 4p13 | Score=1961;Name="3339012:L1MA9(LINE)" | bnd_48 | tandem_dup_7609_0 |
| 50T | 4 | 83017337 | 83017337 | . | intergenic | 4q21.22 | Score=6451;Name="3399200:L1MA2(LINE)" | bnd_81 | transl_inter_8109 |
| 50T | 4 | 105571668 | 105571668 | . | intergenic | 4q24 | Score=5805;Name="3437152:L1MA7(LINE)" | bnd_86 | transl_inter_6622 |
| 50T | 4 | 124662441 | 124662441 | LINC01091 | ncRNA_intronic | 4q28.1 | Score=345;Name="3468275:(TG)n(Simple_repeat)" | bnd_83 | transl_inter_8933 |
| 50T | 4 | 140572422 | 140572422 | . | intergenic | 4q31.1 | . | bnd_47 | tandem_dup_7609_0 |
| 50T | 4 | 142959944 | 142959944 | INPP4B | intronic | 4q31.21 | . | bnd_88 | transl_inter_9363 |
| 50T | 5 | 7210635 | 7210635 | . | intergenic | 5p15.31 | . | bnd_49 | transl_inter_6762 |
| 50T | 5 | 145634794 | 145634794 | RBM27 | intronic | 5q32 | . | bnd_65 | transl_inter_6657_0 |
| 50T | 6 | 91750826 | 91750826 | . | intergenic | 6q15 | . | bnd_90 | transl_inter_6620 |
| 50T | 6 | 144676559 | 144676559 | UTRN | intronic | 6q24.2 | Score=1758;Name="4131285:AluJb(SINE)" | bnd_79 | transl_inter_8462 |
| 50T | 7 | 8347187 | 8347187 | . | intergenic | 7p21.3 | Score=2064;Name="4242409:L2a(LINE)" | bnd_67 | transl_inter_8241 |

| | | | | | | | | |
|---|---|---|---|---|---|---|---|---|
| 50T 7 | 28171470 | 28171470 | JAZF1 | intronic | 7p15.1 | . | bnd_87 | transl_inter_9363 |
| 50T 7 | 29992021 | 29992021 | SCRN1 | intronic | 7p14.3 | . | bnd_85 | transl_inter_6622 |
| 50T 8 | 62741534 | 62741534 | . | intergenic | 8q12.3 | Score=1506;Name="4608658:AluJo(SINE)" | bnd_75 | transl_inter_8459 |
| 50T 8 | 65437481 | 65437481 | . | intergenic | 8q12.3 | . | bnd_53 | transl_inter_8434 |
| 50T 8 | 89349968 | 89349968 | . | intergenic | 8q21.3 | . | bnd_73 | transl_inter_8512 |
| 50T 8 | 111387150 | 111387150 | . | intergenic | 8q23.2 | . | bnd_63 | transl_inter_8452 |
| 50T 8 | 114152670 | 114152670 | CSMD3 | intronic | 8q23.3 | Score=8890;Name="4697541:L1MD1(LINE)" | bnd_89 | transl_inter_6620 |
| 50T 9 | 28919654 | 28919654 | LINGO2 | intronic | 9p21.1 | . | bnd_42 | transl_intra_2378 |
| 50T 9 | 29085985 | 29085985 | LINGO2 | intronic | 9p21.1 | . | bnd_30 | transl_intra_296_0 |
| 50T 9 | 29895873 | 29895873 | . | intergenic | 9p21.1 | . | bnd_29 | transl_intra_296_0 |
| 50T 9 | 32171272 | 32171272 | . | intergenic | 9p21.1 | Score=4802;Name="4811900:MER49(LTR)" | bnd_32 | transl_intra_2167_0 |
| 50T 9 | 32400182 | 32400182 | ACO1 | intronic | 9p21.1 | Score=2435;Name="4812289:AluSx(SINE)" | bnd_44 | transl_intra_1252 |
| 50T 9 | 32440170 | 32440170 | ACO1 | intronic | 9p21.1 | Score=2448;Name="4812345:AluSz(SINE)" | bnd_43 | transl_intra_1252 |
| 50T 9 | 37029487 | 37029487 | PAX5 | intronic | 9p13.2 | . | bnd_41 | transl_intra_2378 |
| 50T 9 | 107910587 | 107910587 | . | intergenic | 9q31.1 | Score=443;Name="4913766:AluSp(SINE)" | bnd_57 | transl_inter_6571 |
| 50T 9 | 120942080 | 120942080 | . | intergenic | 9q33.1 | Score=1761;Name="4941151:AluJb(SINE)" | bnd_31 | transl_intra_2167_0 |
| 50T 11 | 63450917 | 63450917 | RTN3 | intronic | 11q13.1 | . | bnd_16 | del_ins_95 |
| 50T 11 | 66462864 | 66462864 | SPTBN2 | intronic | 11q13.2 | Score=2027;Name="784148:AluJb(SINE)" | bnd_15 | del_ins_95 |
| 50T 11 | 66929710 | 66929710 | KDM2A | intronic | 11q13.2 | . | bnd_34 | transl_intra_2281 |
| 50T 11 | 66955058 | 66955058 | KDM2A | intronic | 11q13.2 | Score=434;Name="785443:(TTTA)n(Simple_repeat)" | bnd_33 | transl_intra_2281 |
| 50T 11 | 68302218 | 68302218 | PPP6R3 | intronic | 11q13.2 | . | bnd_2 | del_3812 |
| 50T 11 | 68303796 | 68303796 | PPP6R3 | intronic | 11q13.2 | Score=1243;Name="788433:AluJr(SINE)" | bnd_1 | del_3812 |
| 50T 11 | 68924265 | 68924265 | RP11-554A11.8 | ncRNA_intronic | 11q13.3 | Score=355;Name="789640:MIRb(SINE)" | bnd_18 | transl_intra_7809_0 |
| 50T 11 | 69388211 | 69388211 | . | intergenic | 11q13.3 | Score=274;Name="790462:L2c(LINE)" | bnd_36 | transl_intra_18 |
| 50T 11 | 69389461 | 69389461 | . | intergenic | 11q13.3 | . | bnd_35 | transl_intra_18 |
| 50T 11 | 69556595 | 69556595 | . | intergenic | 11q13.3 | . | bnd_38 | transl_intra_372 |
| 50T 11 | 69557325 | 69557325 | . | intergenic | 11q13.3 | Score=425;Name="790826:L2a(LINE)" | bnd_37 | transl_intra_372 |
| 50T 11 | 69683168 | 69683168 | . | intergenic | 11q13.3 | . | bnd_40 | transl_intra_241 |
| 50T 11 | 69683722 | 69683722 | . | intergenic | 11q13.3 | . | bnd_4 | del_261_0 |
| 50T 11 | 69690047 | 69690047 | . | intergenic | 11q13.3 | . | bnd_39 | transl_intra_241 |
| 50T 11 | 69993842 | 69993842 | ANO1 | intronic | 11q13.3 | Score=1307;Name="791650:LTR33A(LTR)" | bnd_24 | transl_intra_7797_0 |
| 50T 11 | 70006214 | 70006214 | ANO1 | intronic | 11q13.3 | . | bnd_23 | transl_intra_7797_0 |
| 50T 11 | 70018282 | 70018282 | ANO1 | intronic | 11q13.3 | Score=491;Name="791684:L3(LINE)" | bnd_17 | transl_intra_7809_0 |
| 50T 11 | 70051680 | 70051680 | FADD | intronic | 11q13.3 | Score=499;Name="791769:MLT1D(LTR)" | bnd_22 | transl_intra_221_0 |
| 50T 11 | 70102431 | 70102431 | . | intergenic | 11q13.3 | Score=1765;Name="791904:AluJo(SINE)" | bnd_58 | transl_inter_6571 |
| 50T 11 | 70127801 | 70127801 | PPFIA1 | intronic | 11q13.3 | Score=417;Name="791985:L1ME3A(LINE)" | bnd_3 | del_261_0 |
| 50T 11 | 70128321 | 70128321 | PPFIA1 | intronic | 11q13.3 | . | bnd_21 | transl_intra_221_0 |
| 50T 11 | 70728603 | 70728603 | SHANK2 | intronic | 11q13.4 | . | bnd_60 | transl_inter_1936 |
| 50T 13 | 40927094 | 40927094 | LINC00598 | ncRNA_intronic | 13q14.11 | . | bnd_6 | del_3944_0 |

| | | | | | | | | | |
|---|---|---|---|---|---|---|---|---|---|
| 50T | 13 | 54432270 | 54432270 | LINC00558 | ncRNA_intronic | 13q14.3 | . | bnd_5 | del_3944_0 |
| 50T | 13 | 54872516 | 54872516 | . | intergenic | 13q14.3 | Score=5355;Name="1224644:L1PA15(LINE)" | bnd_46 | tandem_dup_2745_0 |
| 50T | 13 | 54882706 | 54882706 | . | intergenic | 13q14.3 | . | bnd_26 | transl_intra_5181_0 |
| 50T | 13 | 60919217 | 60919217 | . | intergenic | 13q21.2 | Score=2198;Name="1234428:MSTB(LTR)" | bnd_25 | transl_intra_5181_0 |
| 50T | 13 | 105474497 | 105474497 | . | intergenic | 13q33.2 | . | bnd_45 | tandem_dup_2745_0 |
| 50T | 14 | 27178384 | 27178384 | . | intergenic | 14q12 | . | bnd_8 | del_746 |
| 50T | 14 | 27179331 | 27179331 | . | intergenic | 14q12 | Score=2087;Name="1335497:AluSx(SINE)" | bnd_7 | del_746 |
| 50T | 14 | 55201531 | 55201531 | SAMD4A | intronic | 14q22.2 | . | bnd_62 | transl_inter_5550 |
| 50T | 14 | 58615496 | 58615496 | C14orf37 | intronic | 14q23.1 | . | bnd_55 | transl_inter_6671 |
| 50T | 15 | 41236165 | 41236165 | . | intergenic | 15q15.1 | . | bnd_64 | transl_inter_8452 |
| 50T | 15 | 71258049 | 71258049 | LRRC49 | intronic | 15q23 | Score=1240;Name="1575774:L1MC4(LINE)" | bnd_66 | transl_inter_6657_0 |
| 50T | 16 | 63467990 | 63467990 | . | intergenic | 16q21 | . | bnd_68 | transl_inter_8241 |
| 50T | 16 | 74901093 | 74901093 | . | intergenic | 16q23.1 | Score=812;Name="1774033:L1MD2(LINE)" | bnd_70 | transl_inter_2816 |
| 50T | 17 | 5781236 | 5781236 | LOC339166 | ncRNA_intronic | 17p13.2 | . | bnd_59 | transl_inter_1936 |
| 50T | 17 | 40415983 | 40415983 | STAT5B | intronic | 17q21.2 | . | bnd_72 | transl_inter_8347 |
| 50T | 17 | 68652271 | 68652271 | . | intergenic | 17q24.3 | Score=328;Name="1944258:MIRc(SINE)" | bnd_61 | transl_inter_5550 |
| 50T | 18 | 8545648 | 8545648 | . | intergenic | 18p11.22 | . | bnd_74 | transl_inter_8512 |
| 50T | 20 | 791021 | 791021 | . | intergenic | 20p13 | Score=2957;Name="2650254:MER4A(LTR)" | bnd_10 | del_1581 |
| 50T | 20 | 815842 | 815842 | FAM110A | intronic | 20p13 | Score=333;Name="2650327:(TC)n(Simple_repeat)" | bnd_12 | del_1971 |
| 50T | 20 | 824659 | 824659 | FAM110A | intronic | 20p13 | . | bnd_11 | del_1971 |
| 50T | 20 | 829439 | 829439 | . | intergenic | 20p13 | . | bnd_20 | transl_intra_1352_0 |
| 50T | 20 | 831124 | 831124 | . | intergenic | 20p13 | Score=829;Name="2650364:MIR(SINE)" | bnd_51 | transl_inter_1958 |
| 50T | 20 | 842009 | 842009 | . | intergenic | 20p13 | . | bnd_19 | transl_intra_1352_0 |
| 50T | 20 | 1142436 | 1142436 | PSMF1 | intronic | 20p13 | . | bnd_28 | transl_intra_1422 |
| 50T | 20 | 1154624 | 1154624 | . | intergenic | 20p13 | Score=544;Name="2651174:MER102b(DNA)" | bnd_27 | transl_intra_1422 |
| 50T | 20 | 1157500 | 1157500 | . | intergenic | 20p13 | . | bnd_9 | del_1581 |
| 50T | X | 55606048 | 55606048 | . | intergenic | Xp11.21 | Score=1624;Name="5096465:L1MA6(LINE)" | bnd_77 | transl_inter_8209 |
| 51T | 1 | 38996117 | 38996117 | . | intergenic | 1p34.3 | . | bnd_100 | transl_inter_6955 |
| 51T | 1 | 80237830 | 80237830 | . | intergenic | 1p31.1 | . | bnd_2 | del_5424 |
| 51T | 1 | 80238234 | 80238234 | . | intergenic | 1p31.1 | Score=263;Name="171075:L3(LINE)" | bnd_1 | del_5424 |
| 51T | 1 | 96930707 | 96930707 | . | intergenic | 1p21.3 | Score=372;Name="200060:L2c(LINE)" | bnd_24 | transl_intra_2639_0 |
| 51T | 1 | 96955719 | 96955719 | . | intergenic | 1p21.3 | . | bnd_23 | transl_intra_2639_0 |
| 51T | 1 | 98223171 | 98223171 | DPYD | intronic | 1p21.3 | . | bnd_52 | transl_intra_1061 |
| 51T | 1 | 98587912 | 98587912 | . | intergenic | 1p21.3 | . | bnd_51 | transl_intra_1061 |
| 51T | 1 | 155844020 | 155844020 | SYT11 | intronic | 1q22 | Score=1605;Name="265887:AluSz6(SINE)" | bnd_72 | tandem_dup_2080 |
| 51T | 1 | 155975205 | 155975205 | . | intergenic | 1q22 | . | bnd_102 | transl_inter_2785 |
| 51T | 1 | 156114034 | 156114034 | MIR7851 | ncRNA_intronic | 1q22 | Score=2570;Name="266494:L2a(LINE)" | bnd_71 | tandem_dup_2080 |
| 51T | 1 | 156147496 | 156147496 | SEMA4A | UTR3 | 1q22 | . | bnd_104 | transl_inter_5989 |
| 51T | 1 | 173252540 | 173252540 | LOC100506023 | ncRNA_intronic | 1q25.1 | . | bnd_74 | tandem_dup_360 |
| 51T | 1 | 173359301 | 173359301 | LOC100506023 | ncRNA_intronic | 1q25.1 | Score=613;Name="296659:L1MEd(LINE)" | bnd_73 | tandem_dup_360 |

| | | | | | | | | | |
|---|---|---|---|---|---|---|---|---|---|
| 51T | 1 | 179570315 | 179570315 | TDRD5 | intronic | 1q25.2 | Score=1796;Name="307867:L1ME1(LINE)" | bnd_106 | transl_inter_2725 |
| 51T | 1 | 187911749 | 187911749 | . | intergenic | 1q31.1 | . | bnd_28 | del_2132_0 |
| 51T | 1 | 192009413 | 192009413 | . | intergenic | 1q31.2 | Score=1630;Name="329568:Charlie2a(DNA)" | bnd_54 | transl_intra_1085 |
| 51T | 1 | 194029067 | 194029067 | . | intergenic | 1q31.3 | Score=9277;Name="332968:MSTA-int(LTR)" | bnd_53 | transl_intra_1085 |
| 51T | 1 | 195444160 | 195444160 | . | intergenic | 1q31.3 | Score=5529;Name="335349:L1PB3(LINE)" | bnd_36 | transl_intra_648_0 |
| 51T | 1 | 197689628 | 197689628 | DENND1B | intronic | 1q31.3 | Score=1815;Name="338959:AluJo(SINE)" | bnd_4 | del_1851 |
| 51T | 1 | 197694148 | 197694148 | DENND1B | intronic | 1q31.3 | Score=704;Name="338967:L1MEd(LINE)" | bnd_3 | del_1851 |
| 51T | 1 | 205168399 | 205168399 | DSTYK | intronic | 1q32.1 | . | bnd_6 | del_562 |
| 51T | 1 | 212928368 | 212928368 | NSL1 | intronic | 1q32.3 | . | bnd_5 | del_562 |
| 51T | 1 | 216011929 | 216011929 | USH2A | intronic | 1q41 | . | bnd_38 | transl_intra_1198_0 |
| 51T | 1 | 236160390 | 236160390 | NID1 | intronic | 1q42.3 | Score=2339;Name="408804:AluSq(SINE)" | bnd_76 | tandem_dup_786_0 |
| 51T | 1 | 239071215 | 239071215 | . | intergenic | 1q43 | . | bnd_78 | tandem_dup_1057 |
| 51T | 1 | 239088537 | 239088537 | . | intergenic | 1q43 | Score=1705;Name="414290:MER101-int(LTR)" | bnd_77 | tandem_dup_1057 |
| 51T | 1 | 239370624 | 239370624 | . | intergenic | 1q43 | Score=1745;Name="414798:MSTB1(LTR)" | bnd_35 | transl_intra_648_0 |
| 51T | 1 | 241969295 | 241969295 | . | intergenic | 1q43 | Score=5607;Name="419513:L1MC1(LINE)" | bnd_40 | transl_intra_1201_0 |
| 51T | 1 | 241982066 | 241982066 | . | intergenic | 1q43 | Score=451;Name="419542:MIRb(SINE)" | bnd_27 | del_2132_0 |
| 51T | 1 | 241982345 | 241982345 | . | intergenic | 1q43 | . | bnd_39 | transl_intra_1201_0 |
| 51T | 1 | 241996034 | 241996034 | . | intergenic | 1q43 | . | bnd_37 | transl_intra_1198_0 |
| 51T | 1 | 242803791 | 242803791 | . | intergenic | 1q43 | . | bnd_75 | tandem_dup_786_0 |
| 51T | 2 | 22854122 | 22854122 | . | intergenic | 2p24.1 | . | bnd_136 | transl_inter_4010 |
| 51T | 2 | 69003965 | 69003965 | ARHGAP25 | intronic | 2p13.3 | . | bnd_64 | transl_intra_1428 |
| 51T | 2 | 69004913 | 69004913 | ARHGAP25 | intronic | 2p13.3 | . | bnd_63 | transl_intra_1428 |
| 51T | 2 | 69076109 | 69076109 | . | intergenic | 2p13.3 | . | bnd_12 | del_791 |
| 51T | 2 | 69080728 | 69080728 | . | intergenic | 2p13.3 | . | bnd_11 | del_791 |
| 51T | 2 | 108098969 | 108098969 | . | intergenic | 2q12.3 | . | bnd_123 | transl_inter_6861 |
| 51T | 2 | 112886310 | 112886310 | . | intergenic | 2q13 | Score=5263;Name="2426863:L1MB7(LINE)" | bnd_80 | tandem_dup_3012_0 |
| 51T | 2 | 113552899 | 113552899 | . | intergenic | 2q13 | . | bnd_79 | tandem_dup_3012_0 |
| 51T | 2 | 128034079 | 128034079 | ERCC3 | intronic | 2q14.3 | Score=3020;Name="2453783:HAL1(LINE)" | bnd_138 | transl_inter_7025 |
| 51T | 2 | 140970167 | 140970167 | . | intergenic | 2q22.1 | . | bnd_82 | tandem_dup_3210 |
| 51T | 2 | 141084319 | 141084319 | LRP1B | intronic | 2q22.1 | . | bnd_81 | tandem_dup_3210 |
| 51T | 2 | 153886744 | 153886744 | . | intergenic | 2q23.3 | . | bnd_84 | tandem_dup_4437 |
| 51T | 2 | 154029504 | 154029504 | . | intergenic | 2q23.3 | Score=356;Name="2496364:MIRb(SINE)" | bnd_83 | tandem_dup_4437 |
| 51T | 2 | 177536870 | 177536870 | . | intergenic | 2q31.1 | . | bnd_140 | transl_inter_5579 |
| 51T | 3 | 10116573 | 10116573 | FANCD2 | intronic | 3p25.3 | . | bnd_88 | tandem_dup_3388 |
| 51T | 3 | 10249763 | 10249763 | IRAK2 | intronic | 3p25.3 | . | bnd_87 | tandem_dup_3388 |
| 51T | 3 | 59975218 | 59975218 | FHIT | intronic | 3p14.2 | . | bnd_144 | transl_inter_1765 |
| 51T | 3 | 61674188 | 61674188 | PTPRG | intronic | 3p14.2 | . | bnd_146 | transl_inter_3158 |
| 51T | 3 | 94337061 | 94337061 | . | intergenic | 3q11.2 | . | bnd_148 | transl_inter_2215 |
| 51T | 3 | 109651895 | 109651895 | . | intergenic | 3q13.13 | . | bnd_22 | del_ins_664 |
| 51T | 3 | 111793228 | 111793228 | TMPRSS7 | exonic | 3q13.2 | . | bnd_21 | del_ins_664 |
| 51T | 3 | 174851252 | 174851252 | NAALADL2 | intronic | 3q26.31 | Score=5678;Name="3211282:SVA_D(Other)" | bnd_121 | transl_inter_6960 |
| 51T | 4 | 72108973 | 72108973 | SLC4A4 | intronic | 4q13.3 | . | bnd_66 | transl_intra_2882 |
| 51T | 4 | 72110612 | 72110612 | SLC4A4 | intronic | 4q13.3 | . | bnd_65 | transl_intra_2882 |
| 51T | 4 | 73189919 | 73189919 | ADAMTS3 | intronic | 4q13.3 | Score=234;Name="3382758:L3(LINE)" | bnd_44 | transl_intra_849_0 |

| | | | | | | | | |
|---|---|---|---|---|---|---|---|---|
| 51T 4 | 73190764 | 73190764 | ADAMTS3 | intronic | 4q13.3 | . | bnd_43 | transl_intra_849_0 |
| 51T 4 | 75641663 | 75641663 | . | intergenic | 4q13.3 | . | bnd_46 | transl_intra_6610_0 |
| 51T 4 | 75641670 | 75641670 | . | intergenic | 4q13.3 | . | bnd_147 | transl_inter_2215 |
| 51T 4 | 75642128 | 75642128 | . | intergenic | 4q13.3 | Score=5789;Name="3386713:L1PA4(LINE)" | bnd_45 | transl_intra_6610_0 |
| 51T 4 | 92456972 | 92456972 | CCSER1 | intronic | 4q22.1 | . | bnd_90 | tandem_dup_2140_0 |
| 51T 4 | 92642428 | 92642428 | . | intergenic | 4q22.1 | Score=933;Name="3416282:L1M5(LINE)" | bnd_89 | tandem_dup_2140_0 |
| 51T 4 | 95207188 | 95207188 | SMARCAD1 | intronic | 4q22.3 | . | bnd_145 | transl_inter_3158 |
| 51T 4 | 95520097 | 95520097 | PDLIM5 | intronic | 4q22.3 | Score=1080;Name="3420924:L2a(LINE)" | bnd_143 | transl_inter_1765 |
| 51T 4 | 130477493 | 130477493 | . | intergenic | 4q28.2 | Score=202;Name="3477846:L2(LINE)" | bnd_125 | transl_inter_6915 |
| 51T 4 | 170308109 | 170308109 | . | intergenic | 4q33 | Score=1469;Name="3543867:MLT1B(LTR)" | bnd_150 | transl_inter_3533 |
| 51T 5 | 345573 | 345573 | AHRR | intronic | 5p15.33 | Score=473;Name="3580091:(TG)n(Simple_repeat)" | bnd_152 | transl_inter_6844 |
| 51T 5 | 14563512 | 14563512 | . | intergenic | 5p15.2 | . | bnd_96 | del_inso_3580_0/4771_0 |
| 51T 5 | 14563523 | 14563523 | . | intergenic | 5p15.2 | . | bnd_107 | transl_inter_1999 |
| 51T 5 | 14660759 | 14660759 | . | intergenic | 5p15.2 | Score=1389;Name="3600753:AluJr4(SINE)" | bnd_98 | del_inso_3580_0/4771_0 |
| 51T 5 | 14660762 | 14660762 | . | intergenic | 5p15.2 | Score=1389;Name="3600753:AluJr4(SINE)" | bnd_133 | transl_inter_2469 |
| 51T 5 | 14665778 | 14665778 | FAM105B | intronic | 5p15.2 | . | bnd_129 | transl_inter_5916 |
| 51T 5 | 16726029 | 16726029 | MYO10 | intronic | 5p15.1 | Score=2242;Name="3604122:AluSx(SINE)" | bnd_109 | transl_inter_3550 |
| 51T 5 | 40048443 | 40048443 | . | intergenic | 5p13.1 | . | bnd_149 | transl_inter_3533 |
| 51T 5 | 45739040 | 45739040 | . | intergenic | 5p12 | Score=1556;Name="3653924:AluJb(SINE)" | bnd_131 | transl_inter_1761 |
| 51T 5 | 50228100 | 50228100 | . | intergenic | 5q11.1 | . | bnd_14 | del_1122_0 |
| 51T 5 | 50421620 | 50421620 | . | intergenic | 5q11.1 | Score=323;Name="3655583:MIRb(SINE)" | bnd_30 | del_1294_0 |
| 51T 5 | 115377814 | 115377814 | . | intergenic | 5q23.1 | Score=1954;Name="3763447:AluJb(SINE)" | bnd_29 | del_1294_0 |
| 51T 5 | 118499104 | 118499104 | DMXL1 | intronic | 5q23.1 | Score=1917;Name="3768629:AluSx(SINE)" | bnd_113 | transl_inter_536 |
| 51T 5 | 118906755 | 118906755 | . | intergenic | 5q23.1 | Score=522;Name="3769302:UCON16(Unknown)" | bnd_92 | tandem_dup_4618_0 |
| 51T 5 | 118993689 | 118993689 | . | intergenic | 5q23.1 | . | bnd_91 | tandem_dup_4618_0 |
| 51T 5 | 119466655 | 119466655 | . | intergenic | 5q23.1 | Score=26619;Name="3770123:L1PA4(LINE)" | bnd_13 | del_1122_0 |
| 51T 5 | 119791912 | 119791912 | . | intergenic | 5q23.1 | . | bnd_68 | transl_intra_3432 |
| 51T 5 | 119818864 | 119818864 | PRR16 | intronic | 5q23.1 | Score=532;Name="3770635:MLT1I(LTR)" | bnd_67 | transl_intra_3432 |
| 51T 5 | 120158074 | 120158074 | . | intergenic | 5q23.1 | Score=1626;Name="3771127:AluJo(SINE)" | bnd_48 | transl_intra_371_0 |
| 51T 5 | 120158674 | 120158674 | . | intergenic | 5q23.1 | . | bnd_47 | transl_intra_371_0 |
| 51T 5 | 180134335 | 180134335 | . | intergenic | 5q35.3 | Score=2571;Name="3885942:AluY(SINE)" | bnd_154 | transl_inter_4700 |
| 51T 6 | 41884018 | 41884018 | MED20 | intronic | 6p21.1 | Score=1851;Name="3965984:AluJr(SINE)" | bnd_117 | transl_inter_5572 |
| 51T 6 | 90302823 | 90302823 | ANKRD6 | intronic | 6q15 | . | bnd_16 | del_239 |
| 51T 6 | 90307202 | 90307202 | ANKRD6 | intronic | 6q15 | . | bnd_15 | del_239 |
| 51T 7 | 1756513 | 1756513 | ELFN1 | intronic | 7p22.3 | . | bnd_153 | transl_inter_4700 |
| 51T 7 | 10827578 | 10827578 | . | intergenic | 7p21.3 | . | bnd_50 | transl_intra_3959_0 |
| 51T 7 | 29170625 | 29170625 | CPVL | intronic | 7p14.3 | . | bnd_49 | transl_intra_3959_0 |

| | | | | | | | | | |
|---|---|---|---|---|---|---|---|---|---|
| 51T | 7 | 36301848 | 36301848 | EEPD1 | intronic | 7p14.2 | Score=583;Name="4288940:AluJo(SINE)" | bnd_32 | del_invers_1584_0/944_0 |
| 51T | 7 | 36302744 | 36302744 | EEPD1 | intronic | 7p14.2 | Score=1607;Name="4288942:AluJb(SINE)" | bnd_33 | del_invers_1584_0/944_0 |
| 51T | 7 | 36303508 | 36303508 | EEPD1 | intronic | 7p14.2 | . | bnd_31 | del_invers_1584_0/944_0 |
| 51T | 7 | 36308547 | 36308547 | EEPD1 | intronic | 7p14.2 | Score=649;Name="4288954:MIR(SINE)" | bnd_34 | del_invers_1584_0/944_0 |
| 51T | 7 | 48095849 | 48095849 | C7orf57 | intronic | 7p12.3 | Score=8802;Name="4309661:L1MA7(LINE)" | bnd_137 | transl_inter_7025 |
| 51T | 7 | 55932533 | 55932533 | . | intergenic | 7p11.2 | Score=1004;Name="4322261:Charlie1a(DNA)" | bnd_135 | transl_inter_4010 |
| 51T | 7 | 70743671 | 70743671 | WBSCR17 | intronic | 7q11.22 | Score=2376;Name="4346537:THE1C(LTR)" | bnd_93 | tandem_dup_1287_0 |
| 51T | 7 | 55322991 | 55322991 | LINC01156 | ncRNA_exonic | 7p11.2 | . | bnd_94 | tandem_dup_1287_0 |
| 51T | 8 | 72982476 | 72982476 | TRPA1 | intronic | 8q13.3 | . | bnd_139 | transl_inter_5579 |
| 51T | 8 | 91490459 | 91490459 | . | intergenic | 8q21.3 | . | bnd_18 | del_246 |
| 51T | 8 | 91491526 | 91491526 | . | intergenic | 8q21.3 | . | bnd_17 | del_246 |
| 51T | 8 | 99909218 | 99909218 | STK3 | intronic | 8q22.2 | Score=2212;Name="4672371:AluJb(SINE)" | bnd_119 | transl_inter_4705 |
| 51T | 9 | 31692359 | 31692359 | . | intergenic | 9p21.1 | . | bnd_141 | transl_inter_1485 |
| 51T | 9 | 104064034 | 104064034 | LPPR1 | intronic | 9q31.1 | Score=885;Name="4907042:Charlie11(DNA)" | bnd_151 | transl_inter_6844 |
| 51T | 9 | 114296008 | 114296008 | ZNF483 | intronic | 9q31.3 | . | bnd_70 | transl_intra_3856 |
| 51T | 9 | 114296405 | 114296405 | ZNF483 | intronic | 9q31.3 | . | bnd_103 | transl_inter_5989 |
| 51T | 9 | 114329411 | 114329411 | PTGR1,ZNF483 | intronic | 9q31.3 | . | bnd_101 | transl_inter_2785 |
| 51T | 9 | 114329470 | 114329470 | PTGR1,ZNF483 | intronic | 9q31.3 | . | bnd_69 | transl_intra_3856 |
| 51T | 11 | 1344157 | 1344157 | . | intergenic | 11p15.5 | Score=3462;Name="672254:L1MCa(LINE)" | bnd_108 | transl_inter_1999 |
| 51T | 11 | 1353582 | 1353582 | . | intergenic | 11p15.5 | Score=1935;Name="672275:L1ME1(LINE)" | bnd_110 | transl_inter_3550 |
| 51T | 11 | 1458491 | 1458491 | BRSK2 | intronic | 11p15.5 | . | bnd_112 | transl_inter_1232 |
| 51T | 11 | 72912833 | 72912833 | . | intergenic | 11q13.4 | Score=382;Name="797526:MIRb(SINE)" | bnd_99 | transl_inter_6955 |
| 51T | 11 | 104021362 | 104021362 | PDGFD | intronic | 11q22.3 | Score=1914;Name="855342:MLT1D(LTR)" | bnd_56 | transl_intra_5338 |
| 51T | 11 | 104060958 | 104060958 | . | intergenic | 11q22.3 | . | bnd_55 | transl_intra_5338 |
| 51T | 12 | 27450760 | 27450760 | STK38L | exonic | 12p11.23 | . | bnd_114 | transl_inter_536 |
| 51T | 12 | 31713470 | 31713470 | DENND5B | intronic | 12p11.21 | Score=2486;Name="966169:L2a(LINE)" | bnd_116 | transl_inter_2216 |
| 51T | 12 | 31717444 | 31717444 | DENND5B | intronic | 12p11.21 | . | bnd_26 | transl_intra_4298_0 |
| 51T | 12 | 31969350 | 31969350 | . | intergenic | 12p11.21 | Score=1688;Name="966920:L1MC4(LINE)" | bnd_25 | transl_intra_4298_0 |
| 51T | 12 | 110877078 | 110877078 | ARPC3 | intronic | 12q24.11 | Score=2178;Name="1107096:AluSz6(SINE)" | bnd_118 | transl_inter_5572 |
| 51T | 12 | 129140590 | 129140590 | TMEM132C | intronic | 12q24.32 | Score=2395;Name="1151678:AluSz(SINE)" | bnd_120 | transl_inter_4705 |
| 51T | 14 | 30297109 | 30297109 | PRKD1 | intronic | 14q12 | Score=244;Name="1340344:CT-rich(Low_complexity)" | bnd_58 | transl_intra_3477 |
| 51T | 14 | 30444355 | 30444355 | . | intergenic | 14q12 | Score=23546;Name="1340589:L1PA8(LINE)" | bnd_57 | transl_intra_3477 |
| 51T | 14 | 33352502 | 33352502 | . | intergenic | 14q13.1 | Score=2237;Name="1345877:AluSz(SINE)" | bnd_42 | transl_intra_4305_0 |

| | | | | | | | | |
|---|---|---|---|---|---|---|---|---|
| 51T 14 | 33353758 | 33353758 | . | intergenic | 14q13.1 | Score=4296;Name="1345882:L1MA8(LINE)" | bnd_41 | transl_intra_4305_0 |
| 51T 14 | 37382747 | 37382747 | SLC25A21 | intronic | 14q13.3 | Score=1719;Name="1353317:AluJb(SINE)" | bnd_122 | transl_inter_6960 |
| 51T 15 | 80763208 | 80763208 | ARNT2 | intronic | 15q25.1 | Score=5255;Name="1595020:L1PREC2(LINE)" | bnd_105 | transl_inter_2725 |
| 51T 16 | 346395 | 346395 | AXIN1 | intronic | 16p13.3 | . | bnd_8 | del_841 |
| 51T 16 | 347789 | 347789 | AXIN1 | exonic | 16p13.3 | . | bnd_20 | del_ins_456 |
| 51T 16 | 348792 | 348792 | AXIN1 | intronic | 16p13.3 | Score=2245;Name="1635123:AluSg(SINE)" | bnd_19 | del_ins_456 |
| 51T 16 | 347236 | 347236 | AXIN1 | intronic | 16p13.3 | . | bnd_7 | del_841 |
| 51T 17 | 41371334 | 41371334 | TMEM106A | UTR3 | 17q21.31 | . | bnd_10 | del_4577 |
| 51T 17 | 41372417 | 41372417 | . | downstream | 17q21.31 | . | bnd_9 | del_4577 |
| 51T 17 | 63864251 | 63864251 | CEP112 | intronic | 17q24.1 | . | bnd_60 | transl_intra_603 |
| 51T 17 | 63870500 | 63870500 | CEP112 | intronic | 17q24.1 | . | bnd_59 | transl_intra_603 |
| 51T 17 | 78309042 | 78309042 | RNF213 | intronic | 17q25.3 | . | bnd_124 | transl_inter_6861 |
| 51T 18 | 76637003 | 76637003 | . | intergenic | 18q23 | . | bnd_126 | transl_inter_6915 |
| 51T 19 | 13541071 | 13541071 | CACNA1A | intronic | 19p13.2 | Score=2239;Name="2133555:AluSq2(SINE)" | bnd_115 | transl_inter_2216 |
| 51T 19 | 18631430 | 18631430 | ELL | intronic | 19p13.11 | . | bnd_62 | transl_intra_486 |
| 51T 19 | 19090831 | 19090831 | . | intergenic | 19p13.11 | Score=2251;Name="2148725:AluSx(SINE)" | bnd_61 | transl_intra_486 |
| 51T 19 | 19268589 | 19268589 | MEF2B,MEF2BNB-MEF2B | intronic | 19p13.11 | Score=2368;Name="2149161:AluSz(SINE)" | bnd_128 | transl_inter_1050 |
| 51T 19 | 19861480 | 19861480 | . | intergenic | 19p13.11 | . | bnd_130 | transl_inter_5916 |
| 51T 19 | 19861961 | 19861961 | . | intergenic | 19p13.11 | . | bnd_111 | transl_inter_1232 |
| 51T 19 | 19881032 | 19881032 | LINC00663 | ncRNA_intronic | 19p13.11 | Score=11078;Name="2150572:SVA_B(Other)" | bnd_132 | transl_inter_1761 |
| 51T 19 | 19977881 | 19977881 | ZNF253 | intronic | 19p13.11 | Score=1165;Name="2150781:AluSz(SINE)" | bnd_134 | transl_inter_2469 |
| 51T 19 | 20177789 | 20177789 | . | intergenic | 19p12 | Score=10316;Name="2151189:HERVL66-int(LTR)" | bnd_97 | del_inso_3580_0/4771_0 |
| 51T 19 | 20187746 | 20187746 | . | intergenic | 19p12 | Score=2627;Name="2151214:AluY(SINE)" | bnd_95 | del_inso_3580_0/4771_0 |
| 51T 20 | 14534171 | 14534171 | MACROD2 | intronic | 20p12.1 | Score=7122;Name="2676991:Tigger3b(DNA)" | bnd_127 | transl_inter_1050 |
| 51T 22 | 29065853 | 29065853 | TTC28 | intronic | 22q12.1 | . | bnd_142 | transl_inter_1485 |
| 51T 22 | 30167343 | 30167343 | . | downstream | 22q12.2 | Score=1613;Name="2861730:AluJr4(SINE)" | bnd_86 | tandem_dup_1105 |
| 51T 22 | 30176905 | 30176905 | . | intergenic | 22q12.2 | . | bnd_85 | tandem_dup_1105 |
| 52T 1 | 34242929 | 34242929 | CSMD2 | intronic | 1p35.1 | . | bnd_56 | del_insod_6222_0/2439_0 |
| 52T 1 | 34242942 | 34242942 | CSMD2 | intronic | 1p35.1 | . | bnd_58 | del_insod_6222_0/2439_0 |
| 52T 1 | 34248212 | 34248212 | CSMD2 | intronic | 1p35.1 | . | bnd_84 | tandem_dup_1398 |
| 52T 1 | 34271225 | 34271225 | CSMD2 | intronic | 1p35.1 | Score=1408;Name="75750:MLT1B(LTR)" | bnd_57 | del_insod_6222_0/2439_0 |
| 52T 1 | 34271234 | 34271234 | CSMD2 | intronic | 1p35.1 | Score=1408;Name="75750:MLT1B(LTR)" | bnd_83 | tandem_dup_1398 |
| 52T 1 | 34271548 | 34271548 | CSMD2 | intronic | 1p35.1 | . | bnd_55 | del_insod_6222_0/2439_0 |
| 52T 1 | 88179277 | 88179277 | . | intergenic | 1p22.3 | . | bnd_86 | tandem_dup_492 |
| 52T 1 | 88196501 | 88196501 | . | intergenic | 1p22.3 | . | bnd_85 | tandem_dup_492 |
| 52T 1 | 93431391 | 93431391 | . | intergenic | 1p22.1 | Score=1658;Name="193782:L2a(LINE)" | bnd_88 | tandem_dup_2212 |
| 52T 1 | 93494323 | 93494323 | . | intergenic | 1p22.1 | . | bnd_87 | tandem_dup_2212 |
| 52T 1 | 111143109 | 111143109 | KCNA2 | intronic | 1p13.3 | . | bnd_138 | transl_inter_8144 |
| 52T 1 | 158153181 | 158153181 | CD1D | intronic | 1q23.1 | . | bnd_140 | transl_inter_6866 |
| 52T 1 | 164576875 | 164576875 | PBX1 | intronic | 1q23.3 | Score=533;Name="281370:L2c(LINE)" | bnd_2 | del_2286 |
| 52T 1 | 164611195 | 164611195 | PBX1 | intronic | 1q23.3 | . | bnd_1 | del_2286 |

| | | | | | | | | |
|---|---|---|---|---|---|---|---|---|
| 52T | 1 | 168432122 | 168432122 | . | intergenic | 1q24.2 | Score=2007;Name="288352:AluSc(SINE)" | bnd_142 | transl_inter_8089 |
| 52T | 1 | 204110641 | 204110641 | ETNK2 | intronic | 1q32.1 | Score=314;Name="351371:MIRc(SINE)" | bnd_144 | transl_inter_4618 |
| 52T | 2 | 2806786 | 2806786 | . | intergenic | 2p25.3 | Score=13149;Name="2237612:Tigger1(DNA)" | bnd_20 | del_1163 |
| 52T | 2 | 2808584 | 2808584 | . | intergenic | 2p25.3 | . | bnd_19 | del_1163 |
| 52T | 2 | 40305808 | 40305808 | SLC8A1-AS1 | ncRNA_intronic | 2p22.1 | Score=1789;Name="2306155:Kanga2_a(DNA)" | bnd_22 | del_959 |
| 52T | 2 | 40307730 | 40307730 | SLC8A1-AS1 | ncRNA_intronic | 2p22.1 | . | bnd_21 | del_959 |
| 52T | 2 | 44809871 | 44809871 | CAMKMT | intronic | 2p21 | . | bnd_24 | del_3126 |
| 52T | 2 | 44814477 | 44814477 | CAMKMT | intronic | 2p21 | Score=821;Name="2314665:L1ME1(LINE)" | bnd_23 | del_3126 |
| 52T | 2 | 74545401 | 74545401 | SLC4A5 | intronic | 2p13.1 | Score=5663;Name="2367915:L1PB4(LINE)" | bnd_147 | transl_inter_8710 |
| 52T | 2 | 136409547 | 136409547 | R3HDM1 | exonic | 2q21.3 | . | bnd_26 | del_1528 |
| 52T | 2 | 136731420 | 136731420 | DARS | intronic | 2q21.3 | Score=1579;Name="2469128:AluJo(SINE)" | bnd_25 | del_1528 |
| 52T | 2 | 155617389 | 155617389 | KCNJ3 | intronic | 2q24.1 | Score=2551;Name="2498796:AluY(SINE)" | bnd_102 | tandem_dup_1245 |
| 52T | 2 | 155640264 | 155640264 | KCNJ3 | intronic | 2q24.1 | Score=774;Name="2498830:MIR(SINE)" | bnd_101 | tandem_dup_1245 |
| 52T | 2 | 178564361 | 178564361 | PDE11A | intronic | 2q31.2 | Score=249;Name="2536991:MIRc(SINE)" | bnd_104 | tandem_dup_7592 |
| 52T | 2 | 178616863 | 178616863 | PDE11A | intronic | 2q31.2 | . | bnd_103 | tandem_dup_7592 |
| 52T | 2 | 183532520 | 183532520 | . | intergenic | 2q32.1 | . | bnd_106 | tandem_dup_696 |
| 52T | 2 | 183578761 | 183578761 | . | intergenic | 2q32.1 | . | bnd_105 | tandem_dup_696 |
| 52T | 2 | 215741768 | 215741768 | AC072062.1 | ncRNA_intronic | 2q35 | Score=580;Name="2599305:MER58A(DNA)" | bnd_108 | tandem_dup_1053 |
| 52T | 2 | 215773117 | 215773117 | AC072062.1 | ncRNA_intronic | 2q35 | . | bnd_107 | tandem_dup_1053 |
| 52T | 3 | 12996972 | 12996972 | IQSEC1 | intronic | 3p25.2 | . | bnd_116 | tandem_dup_935 |
| 52T | 3 | 13039874 | 13039874 | IQSEC1 | intronic | 3p25.2 | Score=2091;Name="2933356:AluSz6(SINE)" | bnd_115 | tandem_dup_935 |
| 52T | 3 | 106391685 | 106391685 | . | intergenic | 3q13.12 | . | bnd_30 | del_7692 |
| 52T | 3 | 106392247 | 106392247 | . | intergenic | 3q13.12 | Score=558;Name="3092670:L2(LINE)" | bnd_29 | del_7692 |
| 52T | 3 | 115182183 | 115182183 | . | intergenic | 3q13.31 | Score=6561;Name="3107218:L1HS(LINE)" | bnd_118 | tandem_dup_721 |
| 52T | 3 | 115213589 | 115213589 | . | intergenic | 3q13.31 | . | bnd_117 | tandem_dup_721 |
| 52T | 4 | 29345624 | 29345624 | . | intergenic | 4p15.1 | . | bnd_52 | del_ins_1846 |
| 52T | 4 | 29358318 | 29358318 | . | intergenic | 4p15.1 | Score=6293;Name="3311530:L1MB2(LINE)" | bnd_51 | del_ins_1846 |
| 52T | 4 | 61451184 | 61451184 | . | intergenic | 4q13.1 | Score=419;Name="3363797:MIRb(SINE)" | bnd_137 | transl_inter_8144 |
| 52T | 4 | 66703779 | 66703779 | . | intergenic | 4q13.2 | Score=4111;Name="3372097:MER21B(LTR)" | bnd_64 | transl_intra_1255_0 |
| 52T | 4 | 66710994 | 66710994 | . | intergenic | 4q13.2 | . | bnd_63 | transl_intra_1255_0 |
| 52T | 4 | 172627575 | 172627575 | . | intergenic | 4q34.1 | . | bnd_54 | del_ins_1097 |
| 52T | 4 | 172634646 | 172634646 | . | intergenic | 4q34.1 | . | bnd_53 | del_ins_1097 |
| 52T | 4 | 188592799 | 188592799 | RP11-565A3.2 | ncRNA_intronic | 4q35.2 | Score=10418;Name="3573914:L1PB1(LINE)" | bnd_149 | transl_inter_8324 |
| 52T | 5 | 24251040 | 24251040 | . | intergenic | 5p14.2 | . | bnd_120 | tandem_dup_2124 |
| 52T | 5 | 24277763 | 24277763 | . | intergenic | 5p14.2 | . | bnd_119 | tandem_dup_2124 |
| 52T | 5 | 46308927 | 46308927 | . | intergenic | 5p11 | Score=1215;Name="3654291:ALR/Alpha(Satellite)" | bnd_74 | transl_intra_1131_0 |

| | | | | | | | | |
|---|---|---|---|---|---|---|---|---|
| 52T 5 | 77453393 | 77453393 | AP3B1 | intronic | 5q14.1 | . | bnd_122 | tandem_dup_2242 |
| 52T 5 | 77484790 | 77484790 | AP3B1 | intronic | 5q14.1 | Score=6895;Name="3702959:L1PA10(LINE)" | bnd_121 | tandem_dup_2242 |
| 52T 5 | 84919541 | 84919541 | . | intergenic | 5q14.3 | Score=2122;Name="3716104:L1MCa(LINE)" | bnd_73 | transl_intra_1131_0 |
| 52T 5 | 171303215 | 171303215 | FBXW11 | intronic | 5q35.1 | . | bnd_124 | tandem_dup_785 |
| 52T 5 | 171321277 | 171321277 | FBXW11 | intronic | 5q35.1 | . | bnd_123 | tandem_dup_785 |
| 52T 5 | 172177325 | 172177325 | . | intergenic | 5q35.1 | . | bnd_126 | tandem_dup_615 |
| 52T 5 | 172231211 | 172231211 | . | intergenic | 5q35.1 | Score=841;Name="3868542:L2a(LINE)" | bnd_125 | tandem_dup_615 |
| 52T 5 | 176471125 | 176471125 | ZNF346 | intronic | 5q35.2 | Score=380;Name="3878103:L2c(LINE)" | bnd_151 | transl_inter_373 |
| 52T 6 | 9914420 | 9914420 | . | intergenic | 6p24.3 | . | bnd_32 | del_2740 |
| 52T 6 | 9916727 | 9916727 | . | intergenic | 6p24.3 | . | bnd_31 | del_2740 |
| 52T 6 | 25877947 | 25877947 | . | intergenic | 6p22.2 | Score=22;Name="3933842:AT_rich(Low_complexity)" | bnd_34 | del_1653 |
| 52T 6 | 25880932 | 25880932 | . | intergenic | 6p22.2 | . | bnd_33 | del_1653 |
| 52T 6 | 50514651 | 50514651 | . | intergenic | 6p12.3 | Score=188;Name="3981784:MIR(SINE)" | bnd_36 | del_1435 |
| 52T 6 | 50517155 | 50517155 | . | intergenic | 6p12.3 | . | bnd_35 | del_1435 |
| 52T 6 | 131001689 | 131001689 | . | intergenic | 6q23.1 | Score=1892;Name="4107423:AluSz6(SINE)" | bnd_145 | transl_inter_5562 |
| 52T 7 | 5690836 | 5690836 | RNF216 | intronic | 7p22.1 | . | bnd_128 | tandem_dup_938 |
| 52T 7 | 5719022 | 5719022 | RNF216-IT1,RNF216 | ncRNA_intronic | 7p22.1 | . | bnd_127 | tandem_dup_938 |
| 52T 7 | 47253137 | 47253137 | . | intergenic | 7p12.3 | . | bnd_38 | del_1627 |
| 52T 7 | 47257933 | 47257933 | . | intergenic | 7p12.3 | . | bnd_37 | del_1627 |
| 52T 7 | 70617128 | 70617128 | WBSCR17 | intronic | 7q11.22 | . | bnd_40 | del_1041 |
| 52T 7 | 70618398 | 70618398 | WBSCR17 | intronic | 7q11.22 | Score=227;Name="4346222:MIR3(SINE)" | bnd_39 | del_1041 |
| 52T 7 | 98726369 | 98726369 | SMURF1 | intronic | 7q22.1 | . | bnd_143 | transl_inter_4618 |
| 52T 7 | 126949930 | 126949930 | . | intergenic | 7q31.33 | Score=2397;Name="4448195:L1MA2(LINE)" | bnd_130 | tandem_dup_1405 |
| 52T 7 | 126974549 | 126974549 | . | intergenic | 7q31.33 | Score=1652;Name="4448231:AluSz6(SINE)" | bnd_129 | tandem_dup_1405 |
| 52T 8 | 28128426 | 28128426 | . | intergenic | 8p21.1 | Score=226;Name="4554158:MIRc(SINE)" | bnd_42 | del_1915 |
| 52T 8 | 28141863 | 28141863 | . | intergenic | 8p21.1 | Score=16023;Name="4554196:Tigger1(DNA)" | bnd_41 | del_1915 |
| 52T 8 | 55657883 | 55657883 | . | intergenic | 8q12.1 | . | bnd_132 | tandem_dup_1230 |
| 52T 8 | 55664920 | 55664920 | . | intergenic | 8q12.1 | Score=4197;Name="4597175:MER6(DNA)" | bnd_131 | tandem_dup_1230 |
| 52T 8 | 61292419 | 61292419 | . | intergenic | 8q12.1 | . | bnd_76 | transl_intra_966_0 |
| 52T 8 | 61298807 | 61298807 | . | intergenic | 8q12.1 | Score=2522;Name="4606219:AluY(SINE)" | bnd_75 | transl_intra_966_0 |
| 52T 8 | 136178353 | 136178353 | . | intergenic | 8q24.22 | Score=494;Name="4741059:MIRb(SINE)" | bnd_155 | transl_inter_6475 |
| 52T 8 | 144007418 | 144007418 | . | intergenic | 8q24.3 | . | bnd_44 | del_1451 |
| 52T 8 | 144012483 | 144012483 | . | intergenic | 8q24.3 | Score=8784;Name="4754948:Ricksha(DNA)" | bnd_43 | del_1451 |
| 52T 9 | 76710655 | 76710655 | MIR6130 | ncRNA_intronic | 9q21.13 | . | bnd_62 | del_inssd_530_0/2563_0 |
| 52T 9 | 76712871 | 76712871 | MIR6130 | ncRNA_intronic | 9q21.13 | Score=932;Name="4856846:L1MEe(LINE)" | bnd_60 | del_inssd_530_0/2563_0 |
| 52T 9 | 90374758 | 90374758 | . | intergenic | 9q21.33 | Score=5868;Name="4881200:SVA_D(Other)" | bnd_153 | transl_inter_1169_0 |

| | | | | | | | | | |
|---|---|---|---|---|---|---|---|---|---|
| 52T | 9 | 112601186 | 112601186 | PALM2,PALM2-AKAP2 | intronic | 9q31.3 | Score=25735;Name="4923182:L1PA5(LINE)" | bnd_66 | transl_intra_2049_0 |
| 52T | 9 | 112613606 | 112613606 | PALM2,PALM2-AKAP2 | intronic | 9q31.3 | Score=420;Name="4923214:MER5B(DNA)" | bnd_65 | transl_intra_2049_0 |
| 52T | 9 | 112616412 | 112616412 | PALM2,PALM2-AKAP2 | intronic | 9q31.3 | . | bnd_61 | del_inssd_530_0/2563_0 |
| 52T | 9 | 112616792 | 112616792 | PALM2-AKAP2,PALM2 | intronic | 9q31.3 | . | bnd_59 | del_inssd_530_0/2563_0 |
| 52T | 9 | 123943714 | 123943714 | RAB14 | exonic | 9q33.2 | . | bnd_46 | del_910 |
| 52T | 9 | 123946718 | 123946718 | RAB14 | intronic | 9q33.2 | . | bnd_45 | del_910 |
| 52T | 9 | 126317829 | 126317829 | DENND1A | intronic | 9q33.3 | . | bnd_134 | tandem_dup_6120 |
| 52T | 9 | 126364141 | 126364141 | DENND1A | intronic | 9q33.3 | . | bnd_133 | tandem_dup_6120 |
| 52T | 10 | 7045176 | 7045176 | . | intergenic | 10p14 | . | bnd_80 | transl_intra_1109 |
| 52T | 10 | 7046864 | 7046864 | . | intergenic | 10p14 | Score=4478;Name="443930:L1PA8A(LINE)" | bnd_79 | transl_intra_1109 |
| 52T | 10 | 7287690 | 7287690 | SFMBT2 | intronic | 10p14 | . | bnd_68 | transl_intra_1288 |
| 52T | 10 | 7289037 | 7289037 | SFMBT2 | intronic | 10p14 | . | bnd_67 | transl_intra_1288 |
| 52T | 10 | 7627988 | 7627988 | ITIH5 | exonic | 10p14 | . | bnd_70 | transl_intra_1817 |
| 52T | 10 | 7628246 | 7628246 | ITIH5 | intronic | 10p14 | . | bnd_69 | transl_intra_1817 |
| 52T | 12 | 48390970 | 48390970 | COL2A1 | intronic | 12q13.11 | . | bnd_139 | transl_inter_6866 |
| 52T | 12 | 61201987 | 61201987 | . | intergenic | 12q14.1 | Score=3887;Name="1013505:L1PB3(LINE)" | bnd_4 | del_3688 |
| 52T | 12 | 61204103 | 61204103 | . | intergenic | 12q14.1 | Score=26352;Name="1013507:L1PA3(LINE)" | bnd_3 | del_3688 |
| 52T | 12 | 68105137 | 68105137 | RP11-43N5.1 | ncRNA_intronic | 12q15 | Score=1858;Name="1025988:AluSq2(SINE)" | bnd_146 | transl_inter_5562 |
| 52T | 12 | 131541532 | 131541532 | GPR133 | intronic | 12q24.33 | . | bnd_90 | tandem_dup_2692 |
| 52T | 12 | 131548141 | 131548141 | GPR133 | intronic | 12q24.33 | Score=4748;Name="1156486:L1MD1(LINE)" | bnd_89 | tandem_dup_2692 |
| 52T | 13 | 100695887 | 100695887 | . | intergenic | 13q32.3 | Score=1643;Name="1300841:L1MC4(LINE)" | bnd_148 | transl_inter_8710 |
| 52T | 14 | 21700828 | 21700828 | HNRNPC | intronic | 14q11.2 | . | bnd_92 | tandem_dup_3005 |
| 52T | 14 | 21797423 | 21797423 | RPGRIP1 | intronic | 14q11.2 | Score=1917;Name="1326609:AluSx(SINE)" | bnd_91 | tandem_dup_3005 |
| 52T | 14 | 43024080 | 43024080 | . | intergenic | 14q21.1 | Score=2024;Name="1362498:AluSx1(SINE)" | bnd_6 | del_3454 |
| 52T | 14 | 43024474 | 43024474 | . | intergenic | 14q21.1 | . | bnd_5 | del_3454 |
| 52T | 14 | 53647844 | 53647844 | . | intergenic | 14q22.1 | Score=40847;Name="1380641:HERVIP10FH-int(LTR)" | bnd_136 | transl_inter_2611_0 |
| 52T | 14 | 82019869 | 82019869 | . | intergenic | 14q31.1 | Score=2124;Name="1434432:MLT1D(LTR)" | bnd_48 | del_ins_2443 |
| 52T | 14 | 82021095 | 82021095 | . | intergenic | 14q31.1 | Score=2787;Name="1434436:L1MC3(LINE)" | bnd_47 | del_ins_2443 |
| 52T | 14 | 85516876 | 85516876 | . | intergenic | 14q31.3 | . | bnd_94 | tandem_dup_965 |
| 52T | 14 | 85540476 | 85540476 | . | intergenic | 14q31.3 | . | bnd_93 | tandem_dup_965 |
| 52T | 15 | 26898235 | 26898235 | GABRB3 | intronic | 15q12 | Score=3144;Name="1493302:LTR8(LTR)" | bnd_8 | del_3081 |
| 52T | 15 | 26899516 | 26899516 | GABRB3 | intronic | 15q12 | Score=2475;Name="1493304:AluY(SINE)" | bnd_7 | del_3081 |
| 52T | 15 | 51608734 | 51608734 | CYP19A1 | intronic | 15q21.2 | . | bnd_150 | transl_inter_8324 |
| 52T | 15 | 90560595 | 90560595 | ZNF710 | intronic | 15q26.1 | . | bnd_96 | tandem_dup_3111 |
| 52T | 15 | 90615645 | 90615645 | ZNF710 | intronic | 15q26.1 | Score=300;Name="1613708:MER5A(DNA)" | bnd_95 | tandem_dup_3111 |
| 52T | 16 | 14435415 | 14435415 | . | intergenic | 16p13.12 | Score=1498;Name="1666263:LTR16C(LTR)" | bnd_10 | del_1596 |
| 52T | 16 | 14437855 | 14437855 | . | intergenic | 16p13.12 | Score=2058;Name="1666271:AluJo(SINE)" | bnd_9 | del_1596 |

| | | | | | | | | | |
|---|---|---|---|---|---|---|---|---|---|
| 52T | 16 | 49772763 | 49772763 | ZNF423 | intronic | 16q12.1 | . | bnd_152 | transl_inter_373 |
| 52T | 17 | 80566065 | 80566065 | . | intergenic | 17q25.3 | Score=2556;Name="1967097:AluY(SINE)" | bnd_12 | del_1875 |
| 52T | 17 | 80571171 | 80571171 | . | intergenic | 17q25.3 | . | bnd_11 | del_1875 |
| 52T | 18 | 23644092 | 23644092 | SS18 | intronic | 18q11.2 | Score=8395;Name="2008908:L1ME1(LINE)" | bnd_82 | transl_intra_1121 |
| 52T | 18 | 23649652 | 23649652 | SS18 | intronic | 18q11.2 | . | bnd_72 | transl_intra_1159 |
| 52T | 18 | 23650212 | 23650212 | SS18 | intronic | 18q11.2 | Score=503;Name="2008924:Tigger15a(DNA)" | bnd_81 | transl_intra_1121 |
| 52T | 18 | 23663161 | 23663161 | SS18 | intronic | 18q11.2 | . | bnd_71 | transl_intra_1159 |
| 52T | 18 | 23802827 | 23802827 | . | intergenic | 18q11.2 | . | bnd_98 | tandem_dup_1013 |
| 52T | 18 | 23825780 | 23825780 | TAF4B | intronic | 18q11.2 | . | bnd_97 | tandem_dup_1013 |
| 52T | 18 | 42141116 | 42141116 | . | intergenic | 18q12.3 | Score=2401;Name="2039129:L1MC4(LINE)" | bnd_14 | del_2054 |
| 52T | 18 | 42141799 | 42141799 | . | intergenic | 18q12.3 | Score=537;Name="2039130:L1MEg(LINE)" | bnd_13 | del_2054 |
| 52T | 18 | 64854889 | 64854889 | . | intergenic | 18q22.1 | Score=1802;Name="2078683:L1ME1(LINE)" | bnd_16 | del_967 |
| 52T | 18 | 64863006 | 64863006 | . | intergenic | 18q22.1 | Score=594;Name="2078701:L1MEe(LINE)" | bnd_15 | del_967 |
| 52T | 19 | 7144704 | 7144704 | INSR | intronic | 19p13.2 | Score=2368;Name="2116502:AluSq2(SINE)" | bnd_100 | tandem_dup_498 |
| 52T | 19 | 7188096 | 7188096 | INSR | intronic | 19p13.2 | Score=342;Name="2116646:L2b(LINE)" | bnd_99 | tandem_dup_498 |
| 52T | 19 | 12950210 | 12950210 | MAST1 | intronic | 19p13.2 | . | bnd_17 | del_353 |
| 52T | 19 | 12949659 | 12949659 | MAST1 | intronic | 19p13.2 | . | bnd_18 | del_353 |
| 52T | 20 | 4081699 | 4081699 | . | intergenic | 20p13 | Score=2421;Name="2657787:AluSp(SINE)" | bnd_154 | transl_inter_1169_0 |
| 52T | 20 | 5772932 | 5772932 | C20orf196 | intronic | 20p12.3 | Score=3489;Name="2661560:L1M3(LINE)" | bnd_156 | transl_inter_6475 |
| 52T | 20 | 8694032 | 8694032 | PLCB1 | intronic | 20p12.3 | Score=2067;Name="2666638:AluSx(SINE)" | bnd_141 | transl_inter_8089 |
| 52T | 20 | 16653609 | 16653609 | . | intergenic | 20p12.1 | Score=2511;Name="2680581:AluSq2(SINE)" | bnd_28 | del_3659 |
| 52T | 20 | 16654245 | 16654245 | . | intergenic | 20p12.1 | Score=1955;Name="2680583:MER74A(LTR)" | bnd_27 | del_3659 |
| 52T | 20 | 30997170 | 30997170 | ASXL1 | intronic | 20q11.21 | Score=869;Name="2700520:L1MC4(LINE)" | bnd_110 | tandem_dup_541 |
| 52T | 20 | 31040096 | 31040096 | C20orf112 | exonic | 20q11.21 | . | bnd_109 | tandem_dup_541 |
| 52T | 21 | 23436648 | 23436648 | AP000472.2 | ncRNA_intronic | 21q21.1 | Score=252;Name="2789346:MIR3(SINE)" | bnd_50 | del_ins_951 |
| 52T | 21 | 23437312 | 23437312 | AP000472.2 | ncRNA_intronic | 21q21.1 | . | bnd_49 | del_ins_951 |
| 52T | 21 | 45220045 | 45220045 | RRP1 | intronic | 21q22.3 | . | bnd_112 | tandem_dup_1051 |
| 52T | 21 | 45260396 | 45260396 | . | intergenic | 21q22.3 | Score=1979;Name="2828618:AluJr(SINE)" | bnd_111 | tandem_dup_1051 |
| 52T | 22 | 26868368 | 26868368 | HPS4 | exonic | 22q12.1 | . | bnd_114 | tandem_dup_2309 |
| 52T | 22 | 26898025 | 26898025 | TFIP11 | intronic | 22q12.1 | . | bnd_113 | tandem_dup_2309 |
| 52T | X | 119277814 | 119277814 | . | intergenic | Xq24 | Score=2188;Name="5192152:HERVIP10FH-int(LTR)" | bnd_135 | transl_inter_2611_0 |
| 52T | Y | 7199057 | 7199057 | PRKY | ncRNA_intronic | Yp11.2 | Score=906;Name="5268516:MSTC(LTR)" | bnd_78 | transl_intra_407_0 |
| 52T | Y | 7840276 | 7840276 | . | intergenic | Yp11.2 | Score=1192;Name="5269631:BSR/Beta(Satellite)" | bnd_77 | transl_intra_407_0 |

| | | | | | | | | | |
|---|---|---|---|---|---|---|---|---|---|
| 53T | 1 | 14004457 | 14004457 | . | intergenic | 1p36.21 | Score=2605;Name="25811:L1MA9(LINE)" | bnd_26 | transl_inter_4240 |
| 53T | 1 | 35268706 | 35268706 | . | intergenic | 1p34.3 | . | bnd_20 | transl_intra_479 |
| 53T | 1 | 35270350 | 35270350 | . | intergenic | 1p34.3 | . | bnd_19 | transl_intra_479 |
| 53T | 1 | 35451219 | 35451219 | . | upstream;downstream | 1p34.3 | Score=313;Name="78218:MIRb(SINE)" | bnd_15 | transl_intra_1468_0 |
| 53T | 1 | 41539990 | 41539990 | SCMH1 | intronic | 1p34.2 | Score=602;Name="92667:MIRb(SINE)" | bnd_18 | transl_intra_2595_0 |
| 53T | 1 | 48055489 | 48055489 | . | intergenic | 1p33 | . | bnd_17 | transl_intra_2595_0 |
| 53T | 1 | 220869519 | 220869519 | C1orf115 | intronic | 1q41 | . | bnd_28 | transl_inter_5825 |
| 53T | 1 | 35450663 | 35450663 | ZMYM6NB | intronic | 1p34.3 | . | bnd_16 | transl_intra_1468_0 |
| 53T | 2 | 10373863 | 10373863 | . | intergenic | 2p25.1 | Score=389;Name="2250216:(TG)n(Simple_repeat)" | bnd_4 | del_4941 |
| 53T | 2 | 10374179 | 10374179 | . | intergenic | 2p25.1 | . | bnd_3 | del_4941 |
| 53T | 2 | 49277790 | 49277790 | FSHR | intronic | 2p16.3 | Score=219;Name="2323147:MIR(SINE)" | bnd_6 | del_708 |
| 53T | 2 | 49280847 | 49280847 | FSHR | intronic | 2p16.3 | . | bnd_5 | del_708 |
| 53T | 2 | 158413404 | 158413404 | ACVR1C | intronic | 2q24.1 | Score=2458;Name="2503060:AluSx1(SINE)" | bnd_42 | transl_inter_4289 |
| 53T | 4 | 142465527 | 142465527 | . | intergenic | 4q31.21 | Score=2238;Name="3497514:AluSz(SINE)" | bnd_44 | transl_inter_5261 |
| 53T | 5 | 117190983 | 117190983 | LOC102467224 | ncRNA_intronic | 5q23.1 | Score=536;Name="3766430:FLAM_A(SINE)" | bnd_37 | transl_inter_5582 |
| 53T | 5 | 165301525 | 165301525 | . | intergenic | 5q34 | Score=2254;Name="3854670:AluSp(SINE)" | bnd_29 | transl_inter_5908 |
| 53T | 6 | 72800457 | 72800457 | RIMS1 | intronic | 6q13 | . | bnd_8 | del_865 |
| 53T | 6 | 73023552 | 73023552 | RIMS1 | intronic | 6q13 | . | bnd_7 | del_865 |
| 53T | 6 | 73027566 | 73027566 | RIMS1 | intronic | 6q13 | Score=775;Name="4012376:L1MEe(LINE)" | bnd_10 | del_2588_0 |
| 53T | 6 | 98541812 | 98541812 | . | intergenic | 6q16.1 | . | bnd_9 | del_2588_0 |
| 53T | 6 | 98778060 | 98778060 | . | intergenic | 6q16.1 | . | bnd_46 | transl_inter_1869 |
| 53T | 6 | 105699342 | 105699342 | . | intergenic | 6q21 | Score=1975;Name="4064879:MER74B(LTR)" | bnd_27 | transl_inter_5825 |
| 53T | 6 | 109828207 | 109828207 | AK9 | intronic | 6q21 | . | bnd_39 | transl_inter_5344 |
| 53T | 7 | 1299802 | 1299802 | . | intergenic | 7p22.3 | . | bnd_43 | transl_inter_5261 |
| 53T | 7 | 107820317 | 107820317 | NRCAM | intronic | 7q31.1 | Score=237;Name="4417530:MIRc(SINE)" | bnd_22 | tandem_dup_4852 |
| 53T | 7 | 107903959 | 107903959 | NRCAM | intronic | 7q31.1 | . | bnd_21 | tandem_dup_4852 |
| 53T | 7 | 118864050 | 118864050 | . | intergenic | 7q31.31 | Score=1457;Name="4435206:AluJr(SINE)" | bnd_41 | transl_inter_4289 |
| 53T | 7 | 141888235 | 141888235 | . | intergenic | 7q34 | Score=500;Name="4476970:FLAM_A(SINE)" | bnd_33 | transl_inter_5811 |
| 53T | 8 | 142223521 | 142223521 | SLC45A4 | intronic | 8q24.3 | Score=1506;Name="4752355:L1M5(LINE)" | bnd_24 | tandem_dup_2665 |
| 53T | 8 | 142517789 | 142517789 | . | upstream | 8q24.3 | . | bnd_23 | tandem_dup_2665 |
| 53T | 9 | 95490470 | 95490470 | BICD2 | intronic | 9q22.31 | . | bnd_35 | transl_inter_5226 |
| 53T | 9 | 97926230 | 97926230 | FANCC | intronic | 9q22.32 | Score=15342;Name="4894418:L1MB2(LINE)" | bnd_12 | del_2849 |
| 53T | 9 | 97942310 | 97942310 | FANCC | intronic | 9q22.32 | Score=359;Name="4894443:L1ME3A(LINE)" | bnd_11 | del_2849 |
| 53T | 11 | 86757384 | 86757384 | TMEM135 | intronic | 11q14.2 | Score=1731;Name="826581:AluSx1(SINE)" | bnd_30 | transl_inter_5908 |
| 53T | 12 | 92262957 | 92262957 | . | intergenic | 12q21.33 | Score=1372;Name="1067554:L1MA4(LINE)" | bnd_32 | transl_inter_5308 |
| 53T | 12 | 12869889 | 12869889 | . | upstream | 12p13.1 | . | bnd_2 | del_4772 |
| 53T | 12 | 12870245 | 12870245 | CDKN1B | UTR5 | 12p13.1 | . | bnd_1 | del_4772 |

| | | | | | | | | |
|---|---|---|---|---|---|---|---|---|
| 53T 14 | 35260288 | 35260288 | BAZ1A | intronic | 14q13.1 | Score=2375;Name="1349213:AluSp(SINE)" | bnd_34 | transl_inter_5811 |
| 53T 15 | 45454222 | 45454222 | DUOX1 | intronic | 15q21.1 | . | bnd_36 | transl_inter_5226 |
| 53T 16 | 8003542 | 8003542 | . | intergenic | 16p13.2 | Score=4321;Name="1650376:L1MA1(LINE)" | bnd_25 | transl_inter_4240 |
| 53T 16 | 30799160 | 30799160 | . | upstream | 16p11.2 | . | bnd_13 | del_ins_2388 |
| 53T 16 | 58325472 | 58325472 | PRSS54 | intronic | 16q21 | Score=1940;Name="1740749:AluJo(SINE)" | bnd_38 | transl_inter_5582 |
| 53T 16 | 30798018 | 30798018 | ZNF629 | intronic | 16p11.2 | . | bnd_14 | del_ins_2388 |
| 53T 18 | 69040239 | 69040239 | . | intergenic | 18q22.3 | Score=2203;Name="2085667:AluSg(SINE)" | bnd_40 | transl_inter_5344 |
| 53T 22 | 29592429 | 29592429 | . | intergenic | 22q12.1 | Score=704;Name="2860349:(CA)n(Simple_repeat)" | bnd_31 | transl_inter_5308 |
| 53T X | 85382089 | 85382089 | . | intergenic | Xq21.2 | Score=9092;Name="5136058:L1PREC2(LINE)" | bnd_45 | transl_inter_1869 |
| 55T 1 | 6838459 | 6838459 | . | intergenic | 1p36.31 | . | bnd_20 | transl_inter_6289 |
| 55T 1 | 34946531 | 34946531 | . | intergenic | 1p34.3 | Score=2550;Name="77079:L1P4(LINE)" | bnd_22 | transl_inter_750 |
| 55T 1 | 59473635 | 59473635 | . | intergenic | 1p32.1 | . | bnd_24 | transl_inter_6488 |
| 55T 1 | 60656239 | 60656239 | . | intergenic | 1p32.1 | Score=2443;Name="135102:MSTB(LTR)" | bnd_2 | del_6900_0 |
| 55T 1 | 109797095 | 109797095 | CELSR2 | intronic | 1p13.3 | . | bnd_26 | transl_inter_8144 |
| 55T 1 | 111338047 | 111338047 | . | intergenic | 1p13.3 | Score=18611;Name="224743:L1PA16(LINE)" | bnd_28 | transl_inter_6493 |
| 55T 1 | 175705398 | 175705398 | TNR | intronic | 1q25.1 | Score=2458;Name="300953:AluY(SINE)" | bnd_30 | transl_inter_8524 |
| 55T 1 | 199725585 | 199725585 | . | intergenic | 1q32.1 | . | bnd_32 | transl_inter_7992 |
| 55T 1 | 207341109 | 207341109 | . | intergenic | 1q32.2 | Score=1720;Name="356930:MLT1A0(LTR)" | bnd_4 | del_1277 |
| 55T 1 | 207667580 | 207667580 | . | intergenic | 1q32.2 | Score=3300;Name="357467:L2a(LINE)" | bnd_3 | del_1277 |
| 55T 1 | 235798886 | 235798886 | GNG4 | intronic | 1q42.3 | Score=912;Name="408049:MSTB1(LTR)" | bnd_1 | del_6900_0 |
| 55T 2 | 3862946 | 3862946 | DCDC2C | intronic | 2p25.3 | Score=5973;Name="2239183:L1PA8A(LINE)" | bnd_60 | transl_inter_7692 |
| 55T 2 | 10234219 | 10234219 | . | intergenic | 2p25.1 | . | bnd_62 | transl_inter_5177 |
| 55T 2 | 199753902 | 199753902 | . | intergenic | 2q33.1 | . | bnd_64 | transl_inter_6214 |
| 55T 2 | 222070289 | 222070289 | . | intergenic | 2q36.1 | . | bnd_49 | transl_inter_7884 |
| 55T 3 | 47441630 | 47441630 | PTPN23 | intronic | 3p21.31 | . | bnd_68 | transl_inter_7911 |
| 55T 3 | 94320944 | 94320944 | . | intergenic | 3q11.2 | Score=5833;Name="3073468:ERV3-16A3_I-int(LTR)" | bnd_6 | del_1780 |
| 55T 3 | 94322564 | 94322564 | . | intergenic | 3q11.2 | Score=5833;Name="3073468:ERV3-16A3_I-int(LTR)" | bnd_5 | del_1780 |
| 55T 3 | 136128945 | 136128945 | STAG1 | intronic | 3q22.3 | . | bnd_35 | transl_inter_8163 |
| 55T 4 | 46225246 | 46225246 | . | intergenic | 4p12 | Score=1214;Name="3342198:MLT2B3(LTR)" | bnd_59 | transl_inter_7692 |
| 55T 4 | 172818253 | 172818253 | GALNTL6 | intronic | 4q34.1 | . | bnd_70 | transl_inter_6007 |
| 55T 4 | 188755256 | 188755256 | . | intergenic | 4q35.2 | Score=385;Name="3574202:AluJb(SINE)" | bnd_53 | transl_inter_7934 |
| 55T 5 | 27935279 | 27935279 | . | intergenic | 5p14.1 | . | bnd_72 | transl_inter_8729 |
| 55T 5 | 86287304 | 86287304 | . | intergenic | 5q14.3 | . | bnd_14 | del_ins_1777 |
| 55T 5 | 86532451 | 86532451 | RP11-72L22.1 | ncRNA_intronic | 5q14.3 | Score=4380;Name="3718743:L1M1(LINE)" | bnd_13 | del_ins_1777 |
| 55T 5 | 90159511 | 90159511 | GPR98 | intronic | 5q14.3 | . | bnd_16 | tandem_dup_1527 |
| 55T 5 | 90160182 | 90160182 | GPR98 | intronic | 5q14.3 | Score=327;Name="3724122:L2c(LINE)" | bnd_15 | tandem_dup_1527 |

| | | | | | | | | |
|---|---|---|---|---|---|---|---|---|
| 55T 5 | 81046611 | 81046611 | SSBP2 | intronic | 5q14.1 | . | bnd_74 | transl_inter_8631 |
| 55T 6 | 31885376 | 31885376 | C2 | intronic | 6p21.33 | Score=1342;Name="3945569:L1PA16(LINE)" | bnd_39 | transl_inter_7942 |
| 55T 6 | 38355351 | 38355351 | BTBD9 | intronic | 6p21.2 | Score=22;Name="3959483:AT_rich(Low_complexity)" | bnd_76 | transl_inter_8601 |
| 55T 6 | 38810249 | 38810249 | DNAH8 | exonic | 6p21.2 | . | bnd_31 | transl_inter_7992 |
| 55T 6 | 39788024 | 39788024 | DAAM2 | intronic | 6p21.2 | . | bnd_55 | transl_inter_7985 |
| 55T 6 | 43650220 | 43650220 | MRPS18A | intronic | 6p21.1 | . | bnd_18 | tandem_dup_301 |
| 55T 6 | 43917394 | 43917394 | . | intergenic | 6p21.1 | . | bnd_17 | tandem_dup_301 |
| 55T 6 | 84012146 | 84012146 | ME1 | intronic | 6q14.2 | Score=5284;Name="4030212:L1PREC2(LINE)" | bnd_47 | transl_inter_6047 |
| 55T 6 | 122240947 | 122240947 | . | intergenic | 6q22.31 | Score=565;Name="4093695:L1MC4a(LINE)" | bnd_43 | transl_inter_6413 |
| 55T 6 | 129525375 | 129525375 | LAMA2 | intronic | 6q22.33 | . | bnd_78 | transl_inter_252 |
| 55T 6 | 163438049 | 163438049 | PACRG | intronic | 6q26 | . | bnd_67 | transl_inter_7911 |
| 55T 7 | 138784416 | 138784416 | ZC3HAV1 | intronic | 7q34 | Score=1369;Name="4470651:L1MB7(LINE)" | bnd_65 | transl_inter_4319 |
| 55T 8 | 23024211 | 23024211 | . | intergenic | 8p21.3 | Score=2944;Name="4544338:MER21-int(LTR)" | bnd_25 | transl_inter_8144 |
| 55T 8 | 41414016 | 41414016 | . | intergenic | 8p11.21 | Score=1452;Name="4579334:AluJr4(SINE)" | bnd_57 | transl_inter_8608 |
| 55T 8 | 69317555 | 69317555 | C8orf34 | intronic | 8q13.2 | . | bnd_77 | transl_inter_252 |
| 55T 8 | 77623395 | 77623395 | ZFHX4 | intronic | 8q21.11 | . | bnd_8 | del_1424 |
| 55T 8 | 77764501 | 77764501 | ZFHX4 | exonic | 8q21.11 | . | bnd_7 | del_1424 |
| 55T 8 | 88558463 | 88558463 | . | intergenic | 8q21.3 | Score=740;Name="4652428:MLT1L(LTR)" | bnd_69 | transl_inter_6007 |
| 55T 8 | 92940250 | 92940250 | . | intergenic | 8q21.3 | . | bnd_23 | transl_inter_6488 |
| 55T 8 | 116642575 | 116642575 | TRPS1 | intronic | 8q23.3 | . | bnd_63 | transl_inter_6214 |
| 55T 8 | 131513931 | 131513931 | . | intergenic | 8q24.22 | . | bnd_71 | transl_inter_8729 |
| 55T 9 | 127847795 | 127847795 | SCAI | intronic | 9q33.3 | . | bnd_10 | del_2211 |
| 55T 9 | 127918350 | 127918350 | PPP6C | intronic | 9q33.3 | . | bnd_9 | del_2211 |
| 55T 10 | 8337875 | 8337875 | . | intergenic | 10p14 | . | bnd_27 | transl_inter_6493 |
| 55T 10 | 26630448 | 26630448 | . | intergenic | 10p12.1 | Score=711;Name="480277:FRAM(SINE)" | bnd_34 | transl_inter_8730 |
| 55T 10 | 91480811 | 91480811 | KIF20B | intronic | 10q23.31 | . | bnd_36 | transl_inter_8163 |
| 55T 10 | 120580636 | 120580636 | . | intergenic | 10q26.11 | . | bnd_38 | transl_inter_6320 |
| 55T 11 | 3996566 | 3996566 | STIM1 | intronic | 11p15.4 | Score=2147;Name="676553:AluSz6(SINE)" | bnd_29 | transl_inter_8524 |
| 55T 11 | 18791255 | 18791255 | PTPN5 | intronic | 11p15.1 | Score=406;Name="703491:L2b(LINE)" | bnd_40 | transl_inter_7942 |
| 55T 11 | 43619497 | 43619497 | . | intergenic | 11p11.2 | . | bnd_42 | transl_inter_6180 |
| 55T 11 | 120707417 | 120707417 | GRIK4 | intronic | 11q23.3 | . | bnd_12 | del_ins_2006 |
| 55T 11 | 120709014 | 120709014 | GRIK4 | intronic | 11q23.3 | Score=766;Name="886415:MIRc(SINE)" | bnd_11 | del_ins_2006 |
| 55T 12 | 126385443 | 126385443 | . | intergenic | 12q24.32 | . | bnd_44 | transl_inter_6413 |
| 55T 12 | 130734408 | 130734408 | . | intergenic | 12q24.33 | Score=1639;Name="1154926:MER66A(LTR)" | bnd_37 | transl_inter_6320 |
| 55T 13 | 64859858 | 64859858 | . | intergenic | 13q21.31 | . | bnd_46 | transl_inter_7895 |
| 55T 14 | 20596096 | 20596096 | . | intergenic | 14q11.2 | Score=21960;Name="1324266:L1PA3(LINE)" | bnd_48 | transl_inter_6047 |
| 55T 14 | 50655541 | 50655541 | SOS2 | intronic | 14q21.3 | Score=1919;Name="1375125:AluJb(SINE)" | bnd_33 | transl_inter_8730 |
| 55T 15 | 35125187 | 35125187 | . | intergenic | 15q14 | . | bnd_50 | transl_inter_7884 |
| 55T 15 | 49055550 | 49055550 | CEP152 | intronic | 15q21.1 | . | bnd_52 | transl_inter_6184 |
| 55T 16 | 6683750 | 6683750 | RBFOX1 | intronic | 16p13.3 | Score=1617;Name="1647699:AluJb(SINE)" | bnd_54 | transl_inter_7934 |
| 55T 16 | 66178954 | 66178954 | . | intergenic | 16q21 | Score=228;Name="1755163:MIR3(SINE)" | bnd_56 | transl_inter_7985 |

| | | | | | | | | | |
|---|---|---|---|---|---|---|---|---|---|
| 55T | 17 | 5735801 | 5735801 | LOC339166 | ncRNA_intronic | 17p13.2 | Score=5884;Name="1817458:MER101-int(LTR)" | bnd_21 | transl_inter_750 |
| 55T | 18 | 3140160 | 3140160 | MYOM1 | intronic | 18p11.31 | Score=2336;Name="1977933:AluSz(SINE)" | bnd_45 | transl_inter_7895 |
| 55T | 18 | 72399028 | 72399028 | ZNF407 | intronic | 18q22.3 | Score=1710;Name="2091101:AluJr4(SINE)" | bnd_58 | transl_inter_8608 |
| 55T | 20 | 35814805 | 35814805 | RPN2 | intronic | 20q11.23 | . | bnd_61 | transl_inter_5177 |
| 55T | 21 | 35973736 | 35973736 | RCAN1 | intronic | 21q22.12 | Score=852;Name="2812201:MIR(SINE)" | bnd_19 | transl_inter_6289 |
| 55T | 22 | 38210451 | 38210451 | GCAT | intronic | 22q13.1 | Score=2190;Name="2880980:AluSq2(SINE)" | bnd_41 | transl_inter_6180 |
| 55T | 22 | 39254928 | 39254928 | . | intergenic | 22q13.1 | . | bnd_66 | transl_inter_4319 |
| 55T | X | 21633065 | 21633065 | CNKSR2 | intronic | Xp22.12 | Score=650;Name="5032021:MLT1L(LTR)" | bnd_75 | transl_inter_8601 |
| 55T | X | 46646378 | 46646378 | . | intergenic | Xp11.23 | Score=2200;Name="5077905:AluSg(SINE)" | bnd_51 | transl_inter_6184 |
| 55T | X | 151806215 | 151806215 | . | upstream | Xq28 | Score=23;Name="5248998:GC_rich(Low_complexity)" | bnd_73 | transl_inter_8631 |
| 56T | 1 | 70632196 | 70632196 | LRRC40 | intronic | 1p31.1 | Score=1661;Name="154813:AluJo(SINE)" | bnd_4 | transl_inter_7421 |
| 56T | 2 | 229364940 | 229364940 | . | intergenic | 2q36.3 | Score=276;Name="2624351:(TG)n(Simple_repeat)" | bnd_30 | transl_inter_7179 |
| 56T | 3 | 160841831 | 160841831 | . | intergenic | 3q26.1 | Score=2133;Name="3187885:AluSx1(SINE)" | bnd_7 | transl_inter_6984 |
| 56T | 4 | 63587949 | 63587949 | . | intergenic | 4q13.1 | . | bnd_23 | transl_inter_7494 |
| 56T | 4 | 167596275 | 167596275 | . | intergenic | 4q32.3 | . | bnd_29 | transl_inter_7179 |
| 56T | 5 | 33583882 | 33583882 | ADAMTS12 | intronic | 5p13.3 | Score=7074;Name="3633251:L1MA1(LINE)" | bnd_32 | transl_inter_7080 |
| 56T | 6 | 37150564 | 37150564 | . | intergenic | 6p21.2 | Score=2180;Name="3957030:AluSx(SINE)" | bnd_21 | transl_inter_5844 |
| 56T | 6 | 86795491 | 86795491 | . | intergenic | 6q14.3 | Score=8593;Name="4034551:L1PA15(LINE)" | bnd_17 | transl_inter_5807 |
| 56T | 6 | 119390460 | 119390460 | FAM184A | intronic | 6q22.31 | . | bnd_34 | transl_inter_7119 |
| 56T | 6 | 121791297 | 121791297 | . | intergenic | 6q22.31 | Score=1861;Name="4092877:AluJr(SINE)" | bnd_5 | transl_inter_7376 |
| 56T | 7 | 53130604 | 53130604 | . | intergenic | 7p12.1 | . | bnd_11 | transl_inter_4735 |
| 56T | 8 | 73410482 | 73410482 | . | intergenic | 8q13.3 | Score=1509;Name="4626972:AluJr(SINE)" | bnd_3 | transl_inter_7421 |
| 56T | 8 | 115875752 | 115875752 | . | intergenic | 8q23.3 | Score=10381;Name="4700391:L1MC1(LINE)" | bnd_27 | transl_inter_7149 |
| 56T | 9 | 93379938 | 93379938 | DIRAS2 | intronic | 9q22.2 | . | bnd_31 | transl_inter_7080 |
| 56T | 9 | 118073984 | 118073984 | 1-Dec | intronic | 9q33.1 | Score=11433;Name="4935087:Tigger1(DNA)" | bnd_25 | transl_inter_7352 |
| 56T | 9 | 118716855 | 118716855 | . | intergenic | 9q33.1 | Score=298;Name="4936364:(CA)n(Simple_repeat)" | bnd_33 | transl_inter_7119 |
| 56T | 11 | 20666459 | 20666459 | SLC6A5 | intronic | 11p15.1 | Score=2306;Name="706970:AluSq(SINE)" | bnd_6 | transl_inter_7376 |
| 56T | 11 | 99603678 | 99603678 | CNTN5 | intronic | 11q22.1 | . | bnd_8 | transl_inter_6984 |
| 56T | 11 | 114346290 | 114346290 | . | intergenic | 11q23.2 | Score=5271;Name="874138:Tigger6a(DNA)" | bnd_10 | transl_inter_5934 |
| 56T | 11 | 121225550 | 121225550 | . | intergenic | 11q24.1 | Score=372;Name="887296:L2b(LINE)" | bnd_12 | transl_inter_4735 |

| | | | | | | | | | |
|---|---|---|---|---|---|---|---|---|---|
| 56T | 12 | 44234059 | 44234059 | TMEM117 | intronic | 12q12 | Score=687;Name="981737:MIRb(SINE)" | bnd_2 | transl_intra_4206_0 |
| 56T | 12 | 72166788 | 72166788 | RAB21 | intronic | 12q21.1 | . | bnd_1 | transl_intra_4206_0 |
| 56T | 12 | 109169445 | 109169445 | . | intergenic | 12q24.11 | Score=618;Name="1102765:MER45A(DNA)" | bnd_14 | transl_inter_5870 |
| 56T | 12 | 117917844 | 117917844 | KSR2 | intronic | 12q24.22 | Score=330;Name="1124996:(TG)n(Simple_repeat)" | bnd_16 | transl_inter_7033 |
| 56T | 13 | 81995371 | 81995371 | . | intergenic | 13q31.1 | Score=8887;Name="1269015:L1PBa(LINE)" | bnd_18 | transl_inter_5807 |
| 56T | 13 | 87041457 | 87041457 | . | intergenic | 13q31.1 | Score=369;Name="1277127:(TATATG)n(Simple_repeat)" | bnd_20 | transl_inter_7207 |
| 56T | 14 | 33326749 | 33326749 | . | intergenic | 14q13.1 | Score=409;Name="1345836:MIR(SINE)" | bnd_22 | transl_inter_5844 |
| 56T | 14 | 93860930 | 93860930 | UNC79 | intronic | 14q32.12 | Score=2300;Name="1456565:AluSg4(SINE)" | bnd_24 | transl_inter_7494 |
| 56T | 15 | 23849798 | 23849798 | . | intergenic | 15q11.2 | . | bnd_26 | transl_inter_7352 |
| 56T | 17 | 17315833 | 17315833 | . | intergenic | 17p11.2 | Score=1453;Name="1841575:L1ME4a(LINE)" | bnd_28 | transl_inter_7149 |
| 56T | 17 | 58238403 | 58238403 | . | intergenic | 17q23.1 | . | bnd_13 | transl_inter_5870 |
| 56T | 20 | 46217989 | 46217989 | NCOA3 | intronic | 20q13.12 | Score=293;Name="2737433:L1MC4(LINE)" | bnd_9 | transl_inter_5934 |
| 56T | X | 5525665 | 5525665 | . | intergenic | Xp22.32 | Score=225;Name="5002463:L1MD3(LINE)" | bnd_19 | transl_inter_7207 |
| 56T | X | 69380874 | 69380874 | IGBP1 | intronic | Xq13.1 | . | bnd_15 | transl_inter_7033 |
| 57T | 1 | 37572848 | 37572848 | . | intergenic | 1p34.3 | . | bnd_36 | del_ins_2453 |
| 57T | 1 | 37573414 | 37573414 | . | intergenic | 1p34.3 | . | bnd_72 | transl_inter_1574 |
| 57T | 1 | 37573422 | 37573422 | . | intergenic | 1p34.3 | . | bnd_35 | del_ins_2453 |
| 57T | 1 | 57404229 | 57404229 | C8B | intronic | 1p32.2 | . | bnd_2 | del_2119 |
| 57T | 1 | 57877056 | 57877056 | DAB1 | intronic | 1p32.2 | Score=391;Name="129054:MIRc(SINE)" | bnd_1 | del_2119 |
| 57T | 1 | 68773109 | 68773109 | . | intergenic | 1p31.3 | . | bnd_66 | transl_intra_1505_0 |
| 57T | 1 | 68783350 | 68783350 | . | intergenic | 1p31.3 | Score=818;Name="151657:MLT1J2(LTR)" | bnd_65 | transl_intra_1505_0 |
| 57T | 1 | 68791814 | 68791814 | . | intergenic | 1p31.3 | Score=585;Name="151679:L2c(LINE)" | bnd_38 | del_ins_1997 |
| 57T | 1 | 68795456 | 68795456 | . | intergenic | 1p31.3 | . | bnd_37 | del_ins_1997 |
| 57T | 1 | 88136654 | 88136654 | . | intergenic | 1p22.3 | Score=1436;Name="184213:AluJr(SINE)" | bnd_74 | transl_inter_6085 |
| 57T | 1 | 156763580 | 156763580 | PRCC | intronic | 1q23.1 | Score=1051;Name="267855:AluJo(SINE)" | bnd_76 | transl_inter_7696 |
| 57T | 1 | 178677970 | 178677970 | . | intergenic | 1q25.2 | . | bnd_78 | transl_inter_7886 |
| 57T | 1 | 182281884 | 182281884 | GS1-122H1.2 | ncRNA_intronic | 1q25.3 | Score=273;Name="312769:L2c(LINE)" | bnd_80 | transl_inter_8060 |
| 57T | 1 | 189128259 | 189128259 | . | intergenic | 1q31.1 | Score=343;Name="324769:L1MEc(LINE)" | bnd_40 | del_ins_4870 |
| 57T | 1 | 189143692 | 189143692 | . | intergenic | 1q31.1 | Score=5403;Name="324781:L1P1(LINE)" | bnd_39 | del_ins_4870 |
| 57T | 1 | 212201933 | 212201933 | INTS7 | intronic | 1q32.3 | . | bnd_82 | transl_inter_7782 |
| 57T | 2 | 74140677 | 74140677 | ACTG2 | exonic | 2p13.1 | . | bnd_104 | transl_inter_8070 |
| 57T | 2 | 108641559 | 108641559 | . | intergenic | 2q12.3 | . | bnd_106 | transl_inter_6540 |
| 57T | 2 | 205553039 | 205553039 | PARD3B | intronic | 2q33.3 | Score=2182;Name="2582624:AluSc8(SINE)" | bnd_108 | transl_inter_6661 |
| 57T | 2 | 206894575 | 206894575 | INO80D | intronic | 2q33.3 | . | bnd_110 | transl_inter_6554 |
| 57T | 2 | 233041432 | 233041432 | DIS3L2 | intronic | 2q37.1 | Score=1191;Name="2631797:AluSc8(SINE)" | bnd_112 | transl_inter_8232 |

| | | | | | | | | |
|---|---|---|---|---|---|---|---|---|
| 57T 2 | 236636898 | 236636898 | AGAP1 | intronic | 2q37.2 | . | bnd_114 | transl_inter_6527 |
| 57T 3 | 14051766 | 14051766 | TPRXL | ncRNA_intronic | 3p25.1 | Score=225;Name="2935440:MIRc(SINE)" | bnd_116 | transl_inter_4459 |
| 57T 3 | 73792575 | 73792575 | . | intergenic | 3p13 | . | bnd_52 | del_ins_2512 |
| 57T 3 | 73809659 | 73809659 | . | intergenic | 3p13 | Score=1670;Name="3046050:THE1D(LTR)" | bnd_51 | del_ins_2512 |
| 57T 3 | 145853250 | 145853250 | PLOD2 | intronic | 3q24 | . | bnd_118 | transl_inter_7845 |
| 57T 3 | 171882898 | 171882898 | FNDC3B | intronic | 3q26.31 | Score=2004;Name="3206276:AluJb(SINE)" | bnd_16 | del_2511 |
| 57T 3 | 171929338 | 171929338 | FNDC3B | intronic | 3q26.31 | Score=552;Name="3206344:L3(LINE)" | bnd_15 | del_2511 |
| 57T 3 | 191973208 | 191973208 | FGF12 | intronic | 3q28 | . | bnd_71 | transl_inter_1574 |
| 57T 4 | 5835645 | 5835645 | CRMP1 | intronic | 4p16.2 | Score=2450;Name="3267164:AluY(SINE)" | bnd_18 | del_2708 |
| 57T 4 | 5839414 | 5839414 | CRMP1 | intronic | 4p16.2 | Score=544;Name="3267168:(TAAAA)n(Simple_repeat)" | bnd_17 | del_2708 |
| 57T 4 | 23765214 | 23765214 | . | intergenic | 4p15.2 | . | bnd_20 | del_2002 |
| 57T 4 | 23772136 | 23772136 | . | intergenic | 4p15.2 | . | bnd_19 | del_2002 |
| 57T 4 | 46200162 | 46200162 | . | intergenic | 4p12 | . | bnd_22 | del_1074 |
| 57T 4 | 46221051 | 46221051 | . | intergenic | 4p12 | . | bnd_21 | del_1074 |
| 57T 4 | 111083751 | 111083751 | ELOVL6 | intronic | 4q25 | Score=1804;Name="3446106:AluJo(SINE)" | bnd_91 | transl_inter_5103 |
| 57T 4 | 141908776 | 141908776 | RNF150 | intronic | 4q31.21 | Score=7895;Name="3496634:L1PA16(LINE)" | bnd_101 | transl_inter_8437 |
| 57T 4 | 167640642 | 167640642 | . | intergenic | 4q32.3 | Score=20377;Name="3539494:HERVH-int(LTR)" | bnd_24 | del_2613 |
| 57T 4 | 167650755 | 167650755 | . | intergenic | 4q32.3 | Score=285;Name="3539512:MIR(SINE)" | bnd_23 | del_2613 |
| 57T 5 | 32411419 | 32411419 | ZFR | intronic | 5p13.3 | Score=1795;Name="3631090:L1MC4(LINE)" | bnd_120 | transl_inter_7495 |
| 57T 5 | 89230504 | 89230504 | . | intergenic | 5q14.3 | . | bnd_85 | transl_inter_7682 |
| 57T 6 | 6853414 | 6853414 | . | intergenic | 6p25.1 | Score=822;Name="3898887:MER58A(DNA)" | bnd_99 | transl_inter_6565 |
| 57T 6 | 15795425 | 15795425 | . | intergenic | 6p22.3 | Score=655;Name="3915422:MIRb(SINE)" | bnd_122 | transl_inter_7839 |
| 57T 6 | 36675501 | 36675501 | RAB44 | intronic | 6p21.2 | . | bnd_62 | transl_intra_1403_0 |
| 57T 6 | 36681451 | 36681451 | RAB44 | intronic | 6p21.2 | . | bnd_61 | transl_intra_1403_0 |
| 57T 6 | 41171720 | 41171720 | . | intergenic | 6p21.1 | Score=1731;Name="3964526:MLT1D(LTR)" | bnd_26 | del_1718 |
| 57T 6 | 41187780 | 41187780 | . | intergenic | 6p21.1 | Score=9394;Name="3964551:L1M2(LINE)" | bnd_25 | del_1718 |
| 57T 6 | 103200028 | 103200028 | . | intergenic | 6q16.3 | Score=2186;Name="4060777:AluSx1(SINE)" | bnd_111 | transl_inter_8232 |
| 57T 6 | 114456820 | 114456820 | . | intergenic | 6q21 | . | bnd_117 | transl_inter_7845 |
| 57T 7 | 8672908 | 8672908 | NXPH1 | intronic | 7p21.3 | Score=2054;Name="4242867:AluSc8(SINE)" | bnd_64 | transl_intra_2435_0 |
| 57T 7 | 8677412 | 8677412 | NXPH1 | intronic | 7p21.3 | . | bnd_63 | transl_intra_2435_0 |
| 57T 7 | 21352533 | 21352533 | . | intergenic | 7p15.3 | Score=2567;Name="4262463:L1ME4a(LINE)" | bnd_97 | transl_inter_8353 |
| 57T 7 | 22622201 | 22622201 | . | intergenic | 7p15.3 | . | bnd_28 | del_2056 |
| 57T 7 | 22632787 | 22632787 | . | intergenic | 7p15.3 | . | bnd_27 | del_2056 |
| 57T 7 | 24455252 | 24455252 | . | intergenic | 7p15.3 | Score=2283;Name="4268270:AluSz(SINE)" | bnd_75 | transl_inter_7696 |
| 57T 7 | 36039023 | 36039023 | . | intergenic | 7p14.2 | Score=1638;Name="4288416:L1MC4(LINE)" | bnd_81 | transl_inter_7782 |

| | | | | | | | | | |
|---|---|---|---|---|---|---|---|---|---|
| 57T | 7 | 38189781 | 38189781 | . | intergenic | 7p14.1 | Score=8082;Name="4292104:L1M1(LINE)" | bnd_109 | transl_inter_6554 |
| 57T | 7 | 83485136 | 83485136 | . | intergenic | 7q21.11 | Score=2008;Name="4373531:MER70A(LTR)" | bnd_30 | del_2752 |
| 57T | 7 | 83485887 | 83485887 | . | intergenic | 7q21.11 | Score=1848;Name="4373532:AluJr(SINE)" | bnd_29 | del_2752 |
| 57T | 7 | 106664780 | 106664780 | . | intergenic | 7q22.3 | . | bnd_115 | transl_inter_4459 |
| 57T | 7 | 116761048 | 116761048 | ST7,ST7-AS2 | ncRNA_intronic | 7q31.2 | . | bnd_60 | transl_intra_1336_0 |
| 57T | 7 | 116762064 | 116762064 | ST7-AS2,ST7 | ncRNA_intronic | 7q31.2 | Score=208;Name="4431829:L2c(LINE)" | bnd_59 | transl_intra_1336_0 |
| 57T | 7 | 128468052 | 128468052 | . | intergenic | 7q32.1 | . | bnd_103 | transl_inter_8070 |
| 57T | 8 | 22144554 | 22144554 | PIWIL2 | intronic | 8p21.3 | Score=1311;Name="4542563:AluJb(SINE)" | bnd_107 | transl_inter_6661 |
| 57T | 8 | 53282914 | 53282914 | ST18 | intronic | 8q11.23 | Score=255;Name="4592874:MER4-int(LTR)" | bnd_113 | transl_inter_6527 |
| 57T | 8 | 76102581 | 76102581 | . | intergenic | 8q21.11 | Score=1219;Name="4631916:AluJr4(SINE)" | bnd_121 | transl_inter_7839 |
| 57T | 8 | 90352414 | 90352414 | . | intergenic | 8q21.3 | Score=19672;Name="4655223:L1MA2(LINE)" | bnd_32 | del_1964 |
| 57T | 8 | 90356586 | 90356586 | . | intergenic | 8q21.3 | . | bnd_31 | del_1964 |
| 57T | 8 | 96313317 | 96313317 | LOC100616530 | ncRNA_intronic | 8q22.1 | . | bnd_54 | del_ins_2384 |
| 57T | 8 | 96317369 | 96317369 | LOC100616530 | ncRNA_intronic | 8q22.1 | . | bnd_53 | del_ins_2384 |
| 57T | 8 | 124307623 | 124307623 | . | intergenic | 8q24.13 | Score=2485;Name="4716002:AluSx(SINE)" | bnd_73 | transl_inter_6085 |
| 57T | 8 | 126644104 | 126644104 | . | intergenic | 8q24.13 | . | bnd_95 | transl_inter_7955 |
| 57T | 8 | 132781949 | 132781949 | . | intergenic | 8q24.22 | Score=482;Name="4734110:MIR3(SINE)" | bnd_56 | del_ins_1147 |
| 57T | 8 | 132801237 | 132801237 | . | intergenic | 8q24.22 | Score=243;Name="4734142:MIR(SINE)" | bnd_55 | del_ins_1147 |
| 57T | 8 | 145847426 | 145847426 | . | intergenic | 8q24.3 | . | bnd_119 | transl_inter_7495 |
| 57T | 9 | 116445325 | 116445325 | . | intergenic | 9q32 | Score=521;Name="4931380:MIRb(SINE)" | bnd_105 | transl_inter_6540 |
| 57T | 9 | 122912508 | 122912508 | . | intergenic | 9q33.2 | Score=317;Name="4945350:L2c(LINE)" | bnd_34 | del_4097 |
| 57T | 9 | 122912894 | 122912894 | . | intergenic | 9q33.2 | . | bnd_33 | del_4097 |
| 57T | 10 | 3643499 | 3643499 | . | intergenic | 10p15.2 | Score=1238;Name="438002:MLT1A1(LTR)" | bnd_4 | del_1460 |
| 57T | 10 | 3644538 | 3644538 | . | intergenic | 10p15.2 | . | bnd_3 | del_1460 |
| 57T | 10 | 36949337 | 36949337 | . | intergenic | 10p11.21 | Score=1729;Name="500028:L1MC(LINE)" | bnd_84 | transl_inter_8511 |
| 57T | 10 | 85001202 | 85001202 | . | intergenic | 10q23.1 | . | bnd_6 | del_2446 |
| 57T | 10 | 85013972 | 85013972 | . | intergenic | 10q23.1 | Score=1003;Name="580456:LTR8A(LTR)" | bnd_5 | del_2446 |
| 57T | 10 | 127997115 | 127997115 | ADAM12 | intronic | 10q26.2 | . | bnd_8 | del_3587 |
| 57T | 10 | 128006520 | 128006520 | ADAM12 | intronic | 10q26.2 | . | bnd_7 | del_3587 |
| 57T | 11 | 15888544 | 15888544 | . | intergenic | 11p15.2 | Score=2554;Name="697900:L1ME3(LINE)" | bnd_68 | tandem_dup_1238 |
| 57T | 11 | 15891941 | 15891941 | . | intergenic | 11p15.2 | Score=2136;Name="697912:AluSx(SINE)" | bnd_67 | tandem_dup_1238 |
| 57T | 11 | 29397362 | 29397362 | . | intergenic | 11p14.1 | Score=367;Name="722092:L1M5(LINE)" | bnd_42 | del_ins_1765 |
| 57T | 11 | 29402078 | 29402078 | . | intergenic | 11p14.1 | Score=539;Name="722100:MIR(SINE)" | bnd_41 | del_ins_1765 |

| | | | | | | | | |
|---|---|---|---|---|---|---|---|---|
| 57T 11 | 92306368 | 92306368 | FAT3 | intronic | 11q14.3 | . | bnd_86 | transl_inter_7682 |
| 57T 11 | 98395960 | 98395960 | . | intergenic | 11q22.1 | Score=2015;Name="845928:AluY(SINE)" | bnd_88 | transl_inter_7999 |
| 57T 11 | 118224155 | 118224155 | CD3G | UTR3 | 11q23.3 | . | bnd_90 | transl_inter_7749 |
| 57T 12 | 18177656 | 18177656 | . | intergenic | 12p12.3 | Score=25896;Name="943205:L1PA4(LINE)" | bnd_10 | del_2990 |
| 57T 12 | 18191199 | 18191199 | . | intergenic | 12p12.3 | Score=38378;Name="943216:HERV9-int(LTR)" | bnd_9 | del_2990 |
| 57T 13 | 31157897 | 31157897 | . | intergenic | 13q12.3 | Score=2276;Name="1182773:AluSc(SINE)" | bnd_92 | transl_inter_5103 |
| 57T 13 | 36247906 | 36247906 | MIR548F5 | ncRNA_intronic | 13q13.3 | Score=186;Name="1191648:MER5A(DNA)" | bnd_44 | del_ins_2028 |
| 57T 13 | 36266357 | 36266357 | MIR548F5 | ncRNA_intronic | 13q13.3 | Score=1348;Name="1191698:MLT1B(LTR)" | bnd_43 | del_ins_2028 |
| 57T 13 | 77990257 | 77990257 | . | intergenic | 13q22.3 | . | bnd_12 | del_1551 |
| 57T 13 | 78004549 | 78004549 | . | intergenic | 13q22.3 | Score=1650;Name="1262605:MER58D(DNA)" | bnd_11 | del_1551 |
| 57T 13 | 100725176 | 100725176 | . | intergenic | 13q32.3 | Score=1714;Name="1300939:AluJo(SINE)" | bnd_79 | transl_inter_8060 |
| 57T 14 | 49263734 | 49263734 | . | intergenic | 14q21.3 | Score=21747;Name="1372370:L1PA7(LINE)" | bnd_94 | transl_inter_6247 |
| 57T 14 | 53831658 | 53831658 | . | intergenic | 14q22.1 | Score=12533;Name="1380976:THE1A-int(LTR)" | bnd_96 | transl_inter_7955 |
| 57T 14 | 105096542 | 105096542 | . | intergenic | 14q32.33 | Score=1116;Name="1478877:L1MEe(LINE)" | bnd_98 | transl_inter_8353 |
| 57T 15 | 36602632 | 36602632 | . | intergenic | 15q14 | . | bnd_14 | del_1817 |
| 57T 15 | 36603548 | 36603548 | . | intergenic | 15q14 | Score=210;Name="1509597:L2c(LINE)" | bnd_13 | del_1817 |
| 57T 15 | 60516293 | 60516293 | . | intergenic | 15q22.2 | Score=318;Name="1553722:L2b(LINE)" | bnd_46 | del_ins_2025 |
| 57T 15 | 60542510 | 60542510 | . | intergenic | 15q22.2 | Score=692;Name="1553754:HAL1(LINE)" | bnd_45 | del_ins_2025 |
| 57T 17 | 62164914 | 62164914 | ERN1 | intronic | 17q23.3 | . | bnd_100 | transl_inter_6565 |
| 57T 18 | 25884269 | 25884269 | . | intergenic | 18q12.1 | Score=8476;Name="2012757:L1PA12(LINE)" | bnd_102 | transl_inter_8437 |
| 57T 18 | 75157779 | 75157779 | . | intergenic | 18q23 | Score=1795;Name="2094974:L1PB1(LINE)" | bnd_70 | tandem_dup_2815 |
| 57T 18 | 75159434 | 75159434 | . | intergenic | 18q23 | Score=273;Name="2094978:(TA)n(Simple_repeat)" | bnd_69 | tandem_dup_2815 |
| 57T 20 | 1427482 | 1427482 | NSFL1C | intronic | 20p13 | . | bnd_77 | transl_inter_7886 |
| 57T 20 | 50131074 | 50131074 | NFATC2 | intronic | 20q13.2 | Score=352;Name="2747426:L2c(LINE)" | bnd_83 | transl_inter_8511 |
| 57T 21 | 46346829 | 46346829 | ITGB2-AS1,ITGB2 | ncRNA_exonic | 21q22.3 | . | bnd_93 | transl_inter_6247 |
| 57T 22 | 18080520 | 18080520 | ATP6V1E1 | intronic | 22q11.21 | Score=1859;Name="2836305:AluSc(SINE)" | bnd_48 | del_ins_1235 |
| 57T 22 | 18489130 | 18489130 | MICAL3 | intronic | 22q11.21 | . | bnd_47 | del_ins_1235 |
| 57T 22 | 27672947 | 27672947 | . | intergenic | 22q12.1 | Score=1447;Name="2856083:MLT1D(LTR)" | bnd_50 | del_ins_1930 |
| 57T 22 | 27675007 | 27675007 | . | intergenic | 22q12.1 | . | bnd_49 | del_ins_1930 |
| 57T 22 | 32861021 | 32861021 | . | intergenic | 22q12.3 | . | bnd_89 | transl_inter_7749 |
| 57T X | 109131427 | 109131427 | . | intergenic | Xq23 | . | bnd_58 | del_ins_2213 |
| 57T X | 109132278 | 109132278 | . | intergenic | Xq23 | . | bnd_57 | del_ins_2213 |
| 57T X | 140060146 | 140060146 | . | intergenic | Xq27.1 | Score=2255;Name="5228865:AluSx(SINE)" | bnd_87 | transl_inter_7999 |

| | | | | | | | | |
|---|---|---|---|---|---|---|---|---|
| 58T 1 | 17455134 | 17455134 | . | intergenic | 1p36.13 | Score=939;Name="33459:LTR24B(LTR)" | bnd_12 | transl_inter_4367 |
| 58T 2 | 24616525 | 24616525 | . | intergenic | 2p23.3 | Score=1878;Name="2275647:AluSx(SINE)" | bnd_25 | transl_inter_3652 |
| 58T 2 | 172281384 | 172281384 | METTL8 | intronic | 2q31.1 | Score=3020;Name="2526029:THE1C(LTR)" | bnd_34 | transl_inter_4548 |
| 58T 3 | 58313255 | 58313255 | . | intergenic | 3p14.3 | Score=1759;Name="3017120:AluSp(SINE)" | bnd_19 | transl_inter_4471 |
| 58T 7 | 39056567 | 39056567 | POU6F2 | intronic | 7p14.1 | . | bnd_31 | transl_inter_4579 |
| 58T 8 | 3484627 | 3484627 | CSMD1 | intronic | 8p23.2 | . | bnd_6 | del_1756 |
| 58T 8 | 3589577 | 3589577 | CSMD1 | intronic | 8p23.2 | . | bnd_5 | del_1756 |
| 58T 9 | 7150004 | 7150004 | KDM4C | intronic | 9p24.1 | . | bnd_33 | transl_inter_4548 |
| 58T 9 | 21883528 | 21883528 | . | intergenic | 9p21.3 | Score=727;Name="4795682:LTR41B(LTR)" | bnd_8 | del_ins_223 |
| 58T 9 | 22054848 | 22054848 | CDKN2B-AS1 | ncRNA_intronic | 9p21.3 | Score=1965;Name="4795958:AluSx1(SINE)" | bnd_7 | del_ins_223 |
| 58T 10 | 3081107 | 3081107 | . | intergenic | 10p15.2 | . | bnd_14 | transl_inter_2862 |
| 58T 10 | 89768652 | 89768652 | . | intergenic | 10q23.31 | Score=204;Name="588903:(CTCA)n(Simple_repeat)" | bnd_16 | transl_inter_4313 |
| 58T 11 | 12207422 | 12207422 | MICAL2 | intronic | 11p15.3 | . | bnd_18 | transl_inter_4566 |
| 58T 11 | 129416332 | 129416332 | . | intergenic | 11q24.3 | . | bnd_20 | transl_inter_4471 |
| 58T 12 | 22828882 | 22828882 | ETNK1 | intronic | 12p12.1 | . | bnd_22 | transl_inter_4348 |
| 58T 12 | 74182533 | 74182533 | . | intergenic | 12q21.1 | Score=6152;Name="1036905:L1MCa(LINE)" | bnd_13 | transl_inter_2862 |
| 58T 13 | 35110310 | 35110310 | LINC00457 | ncRNA_intronic | 13q13.2 | . | bnd_2 | del_1370 |
| 58T 13 | 55619777 | 55619777 | . | intergenic | 13q21.1 | Score=29;Name="1225860:AT_rich(Low_complexity)" | bnd_1 | del_1370 |
| 58T 13 | 102320380 | 102320380 | ITGBL1 | intronic | 13q33.1 | Score=3535;Name="1303690:MER65-int(LTR)" | bnd_24 | transl_inter_4253 |
| 58T 16 | 72595822 | 72595822 | . | intergenic | 16q22.2 | Score=213;Name="1769508:(TAAA)n(Simple_repeat)" | bnd_26 | transl_inter_3652 |
| 58T 17 | 10232757 | 10232757 | MYH13 | intronic | 17p13.1 | . | bnd_10 | transl_intra_324 |
| 58T 17 | 52279596 | 52279596 | . | intergenic | 17q22 | Score=8573;Name="1910179:LTR5_Hs(LTR)" | bnd_9 | transl_intra_324 |
| 58T 17 | 52459774 | 52459774 | . | intergenic | 17q22 | . | bnd_28 | transl_inter_3441 |
| 58T 17 | 60673411 | 60673411 | TLK2 | intronic | 17q23.2 | . | bnd_30 | transl_inter_4544 |
| 58T 17 | 72037984 | 72037984 | . | intergenic | 17q25.1 | . | bnd_32 | transl_inter_4579 |
| 58T 18 | 23137156 | 23137156 | . | intergenic | 18q11.2 | . | bnd_11 | transl_inter_4367 |
| 58T 20 | 4826620 | 4826620 | . | intergenic | 20p13 | Score=536;Name="2659364:L1MC4a(LINE)" | bnd_21 | transl_inter_4348 |
| 58T 20 | 33808851 | 33808851 | . | intergenic | 20q11.22 | Score=309;Name="2707925:L2c(LINE)" | bnd_4 | del_262 |
| 58T 20 | 33902922 | 33902922 | UQCC1 | intronic | 20q11.22 | Score=455;Name="2708147:L2c(LINE)" | bnd_3 | del_262 |
| 58T 21 | 25870406 | 25870406 | . | intergenic | 21q21.2 | Score=580;Name="2793736:AluJr(SINE)" | bnd_17 | transl_inter_4566 |
| 58T 21 | 37907788 | 37907788 | CLDN14 | intronic | 21q22.13 | Score=311;Name="2815838:L1ME4a(LINE)" | bnd_29 | transl_inter_4544 |
| 58T X | 118075775 | 118075775 | . | intergenic | Xq24 | Score=2298;Name="5189402:L1MB3(LINE)" | bnd_23 | transl_inter_4253 |
| 58T X | 131839209 | 131839209 | HS6ST2 | intronic | Xq26.2 | Score=470;Name="5213259:L2(LINE)" | bnd_27 | transl_inter_3441 |
| 58T Y | 19512885 | 19512885 | . | intergenic | Yq11.221 | . | bnd_15 | transl_inter_4313 |
| 62T 1 | 2262348 | 2262348 | MORN1 | intronic | 1p36.33 | . | bnd_91 | tandem_dup_3911 |
| 62T 1 | 8449686 | 8449686 | RERE | intronic | 1p36.23 | . | bnd_94 | tandem_dup_2168 |

| | | | | | | | | | |
|---|---|---|---|---|---|---|---|---|---|
| 62T | 1 | 8608096 | 8608096 | RERE | intronic | 1p36.23 | Score=7624;Name="13972:L1MC4a(LINE)" | bnd_93 | tandem_dup_2168 |
| 62T | 1 | 54769954 | 54769954 | SSBP3 | intronic | 1p32.3 | . | bnd_172 | transl_inter_2022 |
| 62T | 1 | 100440069 | 100440069 | SLC35A3 | intronic | 1p21.2 | Score=718;Name="206094:AluJr(SINE)" | bnd_96 | tandem_dup_4764 |
| 62T | 1 | 100466517 | 100466517 | SLC35A3 | intronic | 1p21.2 | Score=14018;Name="206169:L1MA4A(LINE)" | bnd_95 | tandem_dup_4764 |
| 62T | 1 | 193178640 | 193178640 | CDC73 | intronic | 1q31.2 | Score=525;Name="331590:MIR(SINE)" | bnd_98 | tandem_dup_1221 |
| 62T | 1 | 193368691 | 193368691 | . | intergenic | 1q31.2 | Score=669;Name="331924:(TA)n(Simple_repeat)" | bnd_97 | tandem_dup_1221 |
| 62T | 1 | 226180652 | 226180652 | SDE2 | exonic | 1q42.12 | . | bnd_168 | del_inss_1836_0/1827_0 |
| 62T | 1 | 226180674 | 226180674 | SDE2 | exonic | 1q42.12 | . | bnd_170 | del_inss_1836_0/1827_0 |
| 62T | 1 | 234723132 | 234723132 | . | intergenic | 1q42.3 | Score=2320;Name="405800:AluSx3(SINE)" | bnd_174 | transl_inter_1010 |
| 62T | 1 | 2126400 | 2126400 | C1orf86 | intronic | 1p36.33 | . | bnd_92 | tandem_dup_3911 |
| 62T | 2 | 7293338 | 7293338 | . | intergenic | 2p25.1 | Score=6405;Name="2244601:L1MEc(LINE)" | bnd_16 | del_832 |
| 62T | 2 | 7329964 | 7329964 | . | intergenic | 2p25.1 | . | bnd_15 | del_832 |
| 62T | 2 | 26276295 | 26276295 | RAB10 | intronic | 2p23.3 | . | bnd_192 | transl_inter_1653 |
| 62T | 2 | 45431988 | 45431988 | LINC01121 | ncRNA_intronic | 2p21 | Score=12235;Name="2315677:L1M2(LINE)" | bnd_116 | tandem_dup_1979 |
| 62T | 2 | 45613301 | 45613301 | . | intergenic | 2p21 | Score=3177;Name="2315990:L2b(LINE)" | bnd_115 | tandem_dup_1979 |
| 62T | 2 | 141029150 | 141029150 | LRP1B | intronic | 2q22.1 | . | bnd_118 | tandem_dup_6634 |
| 62T | 2 | 141073844 | 141073844 | LRP1B | intronic | 2q22.1 | . | bnd_117 | tandem_dup_6634 |
| 62T | 2 | 142644160 | 142644160 | LRP1B | intronic | 2q22.2 | . | bnd_18 | del_3027 |
| 62T | 2 | 142666160 | 142666160 | LRP1B | intronic | 2q22.2 | . | bnd_17 | del_3027 |
| 62T | 2 | 216726269 | 216726269 | . | intergenic | 2q35 | . | bnd_120 | tandem_dup_1255 |
| 62T | 2 | 216732504 | 216732504 | . | intergenic | 2q35 | . | bnd_119 | tandem_dup_1255 |
| 62T | 3 | 1214601 | 1214601 | CNTN6 | intronic | 3p26.3 | . | bnd_196 | transl_inter_2848 |
| 62T | 3 | 2986512 | 2986512 | CNTN4 | intronic | 3p26.2 | Score=734;Name="2913098:LTR16A(LTR)" | bnd_78 | transl_intra_3115 |
| 62T | 3 | 4108763 | 4108763 | . | intergenic | 3p26.1 | Score=851;Name="2915114:ERVL-E-int(LTR)" | bnd_77 | transl_intra_3115 |
| 62T | 3 | 4116926 | 4116926 | . | intergenic | 3p26.1 | Score=21862;Name="2915125:L1PA6(LINE)" | bnd_60 | transl_intra_4511 |
| 62T | 3 | 4795256 | 4795256 | ITPR1 | intronic | 3p26.1 | . | bnd_59 | transl_intra_4511 |
| 62T | 3 | 8170476 | 8170476 | . | intergenic | 3p26.1 | . | bnd_80 | transl_intra_2340 |
| 62T | 3 | 8171916 | 8171916 | . | intergenic | 3p26.1 | . | bnd_46 | del_invers_1307_0/1739_0 |
| 62T | 3 | 8178405 | 8178405 | . | intergenic | 3p26.1 | . | bnd_82 | transl_intra_3281 |
| 62T | 3 | 8255045 | 8255045 | . | intergenic | 3p26.1 | . | bnd_20 | del_3953 |
| 62T | 3 | 8262985 | 8262985 | LMCD1-AS1 | ncRNA_exonic | 3p26.1 | Score=6510;Name="2922605:Tigger3b(DNA)" | bnd_47 | del_invers_1307_0/1739_0 |
| 62T | 3 | 8992935 | 8992935 | RAD18 | intronic | 3p25.3 | Score=9624;Name="2924017:L1MCa(LINE)" | bnd_45 | del_invers_1307_0/1739_0 |
| 62T | 3 | 10131043 | 10131043 | FANCD2,FANCD2OS | intronic | 3p25.3 | . | bnd_44 | tandem_dup_1879_0 |
| 62T | 3 | 13671640 | 13671640 | FBLN2 | intronic | 3p25.1 | . | bnd_62 | transl_intra_4711_0 |
| 62T | 3 | 15129887 | 15129887 | ZFYVE20 | intronic | 3p25.1 | . | bnd_61 | transl_intra_4711_0 |
| 62T | 3 | 15584464 | 15584464 | . | intergenic | 3p25.1 | . | bnd_84 | transl_intra_1320_0 |
| 62T | 3 | 19233642 | 19233642 | KCNH8 | intronic | 3p24.3 | . | bnd_50 | del_invers_3457_0/1683_0 |
| 62T | 3 | 19258796 | 19258796 | KCNH8 | intronic | 3p24.3 | . | bnd_48 | del_invers_1307_0/1739_0 |
| 62T | 3 | 19440152 | 19440152 | KCNH8 | intronic | 3p24.3 | Score=411;Name="2945320:MIR(SINE)" | bnd_64 | transl_intra_534_0 |
| 62T | 3 | 20307616 | 20307616 | . | intergenic | 3p24.3 | Score=15259;Name="2946910:L1PA7(LINE)" | bnd_83 | transl_intra_1320_0 |
| 62T | 3 | 21082132 | 21082132 | . | intergenic | 3p24.3 | . | bnd_43 | tandem_dup_1879_0 |

| | | | | | | | | | |
|---|---|---|---|---|---|---|---|---|---|
| 62T | 3 | 22293060 | 22293060 | . | intergenic | 3p24.3 | Score=757;Name="2950016:MER20(DNA)" | bnd_86 | transl_intra_3973_0 |
| 62T | 3 | 22829982 | 22829982 | . | intergenic | 3p24.3 | Score=9763;Name="2950858:L1MB8(LINE)" | bnd_22 | del_4872 |
| 62T | 3 | 22837138 | 22837138 | . | intergenic | 3p24.3 | Score=2347;Name="2950872:THE1D(LTR)" | bnd_51 | del_invers_3457_0/1683_0 |
| 62T | 3 | 23042808 | 23042808 | . | intergenic | 3p24.3 | . | bnd_63 | transl_intra_534_0 |
| 62T | 3 | 23044667 | 23044667 | . | intergenic | 3p24.3 | . | bnd_79 | transl_intra_2340 |
| 62T | 3 | 23067378 | 23067378 | . | intergenic | 3p24.3 | Score=20161;Name="2951284:L1PB1(LINE)" | bnd_49 | del_invers_3457_0/1683_0 |
| 62T | 3 | 23083833 | 23083833 | . | intergenic | 3p24.3 | Score=1289;Name="2951301:LTR33(LTR)" | bnd_19 | del_3953 |
| 62T | 3 | 25633137 | 25633137 | RARB | intronic | 3p24.2 | Score=245;Name="2955830:MIRb(SINE)" | bnd_42 | tandem_dup_1222_0 |
| 62T | 3 | 42640673 | 42640673 | SEC22C | intronic | 3p22.1 | Score=2563;Name="2986612:AluY(SINE)" | bnd_41 | tandem_dup_1222_0 |
| 62T | 3 | 42674462 | 42674462 | NKTR | intronic | 3p22.1 | . | bnd_85 | transl_intra_3973_0 |
| 62T | 3 | 42860815 | 42860815 | ACKR2 | intronic | 3p22.1 | . | bnd_21 | del_4872 |
| 62T | 3 | 53626371 | 53626371 | CACNA1D | intronic | 3p21.1 | Score=297;Name="3008253:L2a(LINE)" | bnd_81 | transl_intra_3281 |
| 62T | 3 | 55336594 | 55336594 | . | intergenic | 3p14.3 | Score=6828;Name="3010884:L1MA9(LINE)" | bnd_52 | del_invers_3457_0/1683_0 |
| 62T | 3 | 71009340 | 71009340 | FOXP1 | intronic | 3p13 | . | bnd_24 | del_2774 |
| 62T | 3 | 71326971 | 71326971 | FOXP1 | intronic | 3p13 | Score=183;Name="3040682:(TTAA)n(Simple_repeat)" | bnd_23 | del_2774 |
| 62T | 3 | 115122490 | 115122490 | . | intergenic | 3q13.31 | Score=1371;Name="3107113:MER4C(LTR)" | bnd_130 | tandem_dup_3807 |
| 62T | 3 | 115200145 | 115200145 | . | intergenic | 3q13.31 | Score=495;Name="3107254:L2a(LINE)" | bnd_129 | tandem_dup_3807 |
| 62T | 3 | 183204923 | 183204923 | . | downstream | 3q27.1 | . | bnd_132 | tandem_dup_3524 |
| 62T | 3 | 183366181 | 183366181 | KLHL24 | intronic | 3q27.1 | . | bnd_131 | tandem_dup_3524 |
| 62T | 3 | 184117735 | 184117735 | . | intergenic | 3q27.1 | . | bnd_88 | transl_intra_1964 |
| 62T | 3 | 184162999 | 184162999 | . | intergenic | 3q27.1 | Score=344;Name="3228651:LTR85b(LTR)" | bnd_87 | transl_intra_1964 |
| 62T | 4 | 30178046 | 30178046 | . | intergenic | 4p15.1 | Score=374;Name="3312913:MIR3(SINE)" | bnd_26 | del_2990 |
| 62T | 4 | 30181127 | 30181127 | . | intergenic | 4p15.1 | . | bnd_25 | del_2990 |
| 62T | 4 | 145499306 | 145499306 | . | intergenic | 4q31.21 | . | bnd_66 | transl_intra_6436_0 |
| 62T | 4 | 145501389 | 145501389 | . | intergenic | 4q31.21 | . | bnd_65 | transl_intra_6436_0 |
| 62T | 4 | 147895875 | 147895875 | . | intergenic | 4q31.22 | Score=3433;Name="3506201:LTR31(LTR)" | bnd_198 | transl_inter_2006 |
| 62T | 4 | 148068290 | 148068290 | . | intergenic | 4q31.22 | Score=1896;Name="3506431:MSTB1(LTR)" | bnd_200 | transl_inter_5867 |
| 62T | 4 | 157814188 | 157814188 | PDGFC | intronic | 4q32.1 | . | bnd_28 | del_4099 |
| 62T | 4 | 157856425 | 157856425 | PDGFC | intronic | 4q32.1 | Score=2267;Name="3523230:AluSc(SINE)" | bnd_27 | del_4099 |
| 62T | 4 | 190782895 | 190782895 | . | intergenic | 4q35.2 | Score=312;Name="3577587:MIRb(SINE)" | bnd_202 | transl_inter_2484 |
| 62T | 5 | 7895410 | 7895410 | MTRR | intronic | 5p15.31 | . | bnd_30 | del_1793 |
| 62T | 5 | 7921256 | 7921256 | . | intergenic | 5p15.31 | Score=6111;Name="3590219:L1ME1(LINE)" | bnd_29 | del_1793 |
| 62T | 5 | 58290021 | 58290021 | PDE4D | intronic | 5q11.2 | . | bnd_38 | del_ins_2299 |
| 62T | 5 | 58470196 | 58470196 | PDE4D | intronic | 5q11.2 | . | bnd_37 | del_ins_2299 |
| 62T | 5 | 137555431 | 137555431 | . | intergenic | 5q31.2 | Score=2360;Name="3800111:AluSq(SINE)" | bnd_134 | tandem_dup_3513 |

| | | | | | | | | | |
|---|---|---|---|---|---|---|---|---|---|
| 62T | 5 | 179790462 | 179790462 | . | intergenic | 5q35.3 | Score=377;Name="3885142:MLT1K(LTR)" | bnd_136 | tandem_dup_1132 |
| 62T | 5 | 179792278 | 179792278 | . | intergenic | 5q35.3 | Score=1097;Name="3885146:Tigger1(DNA)" | bnd_191 | transl_inter_1653 |
| 62T | 5 | 179792616 | 179792616 | . | intergenic | 5q35.3 | Score=2282;Name="3885149:AluSx(SINE)" | bnd_173 | transl_inter_1010 |
| 62T | 5 | 179793433 | 179793433 | . | intergenic | 5q35.3 | . | bnd_135 | tandem_dup_1132 |
| 62T | 5 | 180149262 | 180149262 | . | intergenic | 5q35.3 | Score=3559;Name="3885976:L1MA8(LINE)" | bnd_167 | del_inss_1836_0/1827_0 |
| 62T | 5 | 180153000 | 180153000 | . | intergenic | 5q35.3 | Score=704;Name="3885981:L1MC4a(LINE)" | bnd_169 | del_inss_1836_0/1827_0 |
| 62T | 5 | 137800896 | 137800896 | . | upstream | 5q31.2 | . | bnd_133 | tandem_dup_3513 |
| 62T | 6 | 35922044 | 35922044 | SLC26A8 | intronic | 6p21.31 | . | bnd_138 | tandem_dup_4082 |
| 62T | 6 | 36056244 | 36056244 | MAPK14 | intronic | 6p21.31 | Score=2447;Name="3954373:AluY(SINE)" | bnd_137 | tandem_dup_4082 |
| 62T | 6 | 41567441 | 41567441 | FOXP4 | UTR3 | 6p21.1 | . | bnd_140 | tandem_dup_5385 |
| 62T | 6 | 41736664 | 41736664 | . | intergenic | 6p21.1 | . | bnd_139 | tandem_dup_5385 |
| 62T | 6 | 83697670 | 83697670 | UBE3D | intronic | 6q14.1 | Score=1595;Name="4029648:L1ME3C(LINE)" | bnd_32 | del_3844 |
| 62T | 6 | 83840696 | 83840696 | DOPEY1 | intronic | 6q14.1 | Score=2201;Name="4029938:AluSx(SINE)" | bnd_31 | del_3844 |
| 62T | 6 | 168644003 | 168644003 | . | intergenic | 6q27 | . | bnd_68 | transl_intra_2725 |
| 62T | 6 | 168807210 | 168807210 | . | intergenic | 6q27 | Score=13592;Name="4171242:ERVL-B4-int(LTR)" | bnd_189 | transl_inter_4384 |
| 62T | 6 | 170713229 | 170713229 | FAM120B | intronic | 6q27 | . | bnd_67 | transl_intra_2725 |
| 62T | 7 | 1473782 | 1473782 | . | downstream | 7p22.3 | . | bnd_142 | tandem_dup_2400 |
| 62T | 7 | 1728405 | 1728405 | . | intergenic | 7p22.3 | Score=1755;Name="4228240:L1MD1(LINE)" | bnd_141 | tandem_dup_2400 |
| 62T | 7 | 30217646 | 30217646 | . | intergenic | 7p14.3 | Score=575;Name="4278192:MIRb(SINE)" | bnd_144 | tandem_dup_1298 |
| 62T | 7 | 30281654 | 30281654 | . | intergenic | 7p14.3 | Score=247;Name="4278313:MLT1M(LTR)" | bnd_143 | tandem_dup_1298 |
| 62T | 7 | 36447684 | 36447684 | ANLN | intronic | 7p14.2 | . | bnd_195 | transl_inter_2848 |
| 62T | 7 | 47173302 | 47173302 | . | intergenic | 7p12.3 | Score=5533;Name="4308173:L1MD2(LINE)" | bnd_146 | tandem_dup_2762 |
| 62T | 7 | 47388874 | 47388874 | TNS3 | intronic | 7p12.3 | . | bnd_145 | tandem_dup_2762 |
| 62T | 7 | 101473079 | 101473079 | CUX1 | intronic | 7q22.1 | . | bnd_148 | tandem_dup_4793 |
| 62T | 7 | 101643304 | 101643304 | CUX1 | intronic | 7q22.1 | Score=2082;Name="4405778:AluSq2(SINE)" | bnd_147 | tandem_dup_4793 |
| 62T | 7 | 108512700 | 108512700 | . | intergenic | 7q31.1 | Score=2058;Name="4418612:MLT1D(LTR)" | bnd_197 | transl_inter_2006 |
| 62T | 7 | 108513759 | 108513759 | . | intergenic | 7q31.1 | . | bnd_40 | transl_intra_4830_0 |
| 62T | 7 | 108515189 | 108515189 | . | intergenic | 7q31.1 | Score=20150;Name="4418616:HUERS-P2-int(LTR)" | bnd_150 | tandem_dup_4760 |
| 62T | 7 | 108515405 | 108515405 | . | intergenic | 7q31.1 | Score=20150;Name="4418616:HUERS-P2-int(LTR)" | bnd_199 | transl_inter_5867 |
| 62T | 7 | 108515630 | 108515630 | . | intergenic | 7q31.1 | Score=20150;Name="4418616:HUERS-P2-int(LTR)" | bnd_39 | transl_intra_4830_0 |
| 62T | 7 | 108695522 | 108695522 | . | intergenic | 7q31.1 | . | bnd_149 | tandem_dup_4760 |
| 62T | 7 | 138984170 | 138984170 | UBN2 | UTR3 | 7q34 | . | bnd_181 | transl_inter_6083 |
| 62T | 7 | 139102645 | 139102645 | C7orf55-LUC7L2,LUC7L2,LOC100129148 | ncRNA_intronic | 7q34 | . | bnd_179 | transl_inter_2830 |
| 62T | 8 | 11451922 | 11451922 | . | intergenic | 8p23.1 | . | bnd_152 | tandem_dup_1347_0 |
| 62T | 8 | 11692962 | 11692962 | FDFT1 | intronic | 8p23.1 | . | bnd_34 | del_1052_0 |

| | | | | | | | | | |
|---|---|---|---|---|---|---|---|---|---|
| 62T | 8 | 26109684 | 26109684 | . | intergenic | 8p21.2 | Score=1960;Name="4550181:MLT1A0(LTR)" | bnd_33 | del_1052_0 |
| 62T | 8 | 26252415 | 26252415 | BNIP3L | intronic | 8p21.2 | . | bnd_151 | tandem_dup_1347_0 |
| 62T | 8 | 48945171 | 48945171 | UBE2V2 | intronic | 8q11.21 | Score=1499;Name="4586203:AluJr(SINE)" | bnd_54 | del_invers_5244_0/5372_0 |
| 62T | 8 | 63411138 | 63411138 | NKAIN3 | intronic | 8q12.3 | Score=321;Name="4609671:MLT1H2(LTR)" | bnd_55 | del_invers_5244_0/5372_0 |
| 62T | 8 | 63497708 | 63497708 | NKAIN3 | intronic | 8q12.3 | . | bnd_53 | del_invers_5244_0/5372_0 |
| 62T | 8 | 63537210 | 63537210 | NKAIN3 | intronic | 8q12.3 | Score=5229;Name="4609861:L1MCa(LINE)" | bnd_56 | del_invers_5244_0/5372_0 |
| 62T | 8 | 92198940 | 92198940 | LRRC69 | intronic | 8q21.3 | . | bnd_154 | tandem_dup_3488 |
| 62T | 8 | 92200958 | 92200958 | LRRC69 | intronic | 8q21.3 | Score=238;Name="4658264:(TTTTC)n(Simple_repeat)" | bnd_153 | tandem_dup_3488 |
| 62T | 8 | 98741549 | 98741549 | MTDH | UTR3 | 8q22.1 | Score=841;Name="4669906:MLT1J(LTR)" | bnd_156 | tandem_dup_4150 |
| 62T | 8 | 98748345 | 98748345 | . | intergenic | 8q22.1 | Score=1763;Name="4669929:AluYf4(SINE)" | bnd_155 | tandem_dup_4150 |
| 62T | 8 | 103841016 | 103841016 | AZIN1 | intronic | 8q22.3 | . | bnd_158 | tandem_dup_5231 |
| 62T | 8 | 103994913 | 103994913 | . | intergenic | 8q22.3 | Score=1921;Name="4680498:MER57A-int(LTR)" | bnd_157 | tandem_dup_5231 |
| 62T | 8 | 110130526 | 110130526 | TRHR | intronic | 8q23.1 | Score=366;Name="4690844:MIRb(SINE)" | bnd_36 | del_2208_0 |
| 62T | 8 | 135502059 | 135502059 | ZFAT | intronic | 8q24.22 | Score=1408;Name="4739726:L2(LINE)" | bnd_35 | del_2208_0 |
| 62T | 8 | 135502559 | 135502559 | ZFAT | intronic | 8q24.22 | Score=391;Name="4739727:L3(LINE)" | bnd_193 | transl_inter_5808 |
| 62T | 9 | 19060604 | 19060604 | HAUS6 | intronic | 9p22.1 | . | bnd_160 | tandem_dup_1860 |
| 62T | 9 | 19341275 | 19341275 | DENND4C | intronic | 9p22.1 | . | bnd_159 | tandem_dup_1860 |
| 62T | 9 | 92146502 | 92146502 | . | intergenic | 9q22.2 | . | bnd_162 | tandem_dup_6470 |
| 62T | 9 | 92185891 | 92185891 | . | intergenic | 9q22.2 | . | bnd_161 | tandem_dup_6470 |
| 62T | 9 | 96958091 | 96958091 | MIRLET7DHG | ncRNA_intronic | 9q22.32 | Score=19329;Name="4892510:L1PB3(LINE)" | bnd_164 | tandem_dup_3507 |
| 62T | 9 | 97212247 | 97212247 | HIATL1 | intronic | 9q22.32 | Score=3707;Name="4893130:L1MB8(LINE)" | bnd_163 | tandem_dup_3507 |
| 62T | 9 | 97761743 | 97761743 | C9orf3 | intronic | 9q22.32 | Score=450;Name="4894233:L4(LINE)" | bnd_90 | transl_intra_1399 |
| 62T | 9 | 97768933 | 97768933 | C9orf3 | intronic | 9q22.32 | . | bnd_70 | transl_intra_4111_0 |
| 62T | 9 | 97768998 | 97768998 | C9orf3 | intronic | 9q22.32 | . | bnd_89 | transl_intra_1399 |
| 62T | 9 | 97815668 | 97815668 | C9orf3 | intronic | 9q22.32 | Score=258;Name="4894291:L1MC4a(LINE)" | bnd_69 | transl_intra_4111_0 |
| 62T | 9 | 127868806 | 127868806 | SCAI | intronic | 9q33.3 | . | bnd_166 | tandem_dup_2704 |
| 62T | 9 | 127983936 | 127983936 | RABEPK | intronic | 9q33.3 | . | bnd_165 | tandem_dup_2704 |
| 62T | 9 | 136725939 | 136725939 | VAV2 | intronic | 9q34.2 | . | bnd_201 | transl_inter_2484 |
| 62T | 10 | 934825 | 934825 | LARP4B | intronic | 10p15.3 | Score=1910;Name="434014:AluJb(SINE)" | bnd_72 | transl_intra_3587 |
| 62T | 10 | 938104 | 938104 | LARP4B | intronic | 10p15.3 | . | bnd_71 | transl_intra_3587 |
| 62T | 11 | 19873590 | 19873590 | NAV2 | intronic | 11p15.1 | Score=2802;Name="705475:MLT1D(LTR)" | bnd_2 | del_3928 |
| 62T | 11 | 19900864 | 19900864 | NAV2 | intronic | 11p15.1 | . | bnd_1 | del_3928 |
| 62T | 11 | 60677367 | 60677367 | . | intergenic | 11q12.2 | Score=2357;Name="771524:THE1B(LTR)" | bnd_176 | transl_inter_1846_0 |
| 62T | 11 | 69402742 | 69402742 | . | intergenic | 11q13.3 | . | bnd_178 | transl_inter_1182_0 |
| 62T | 11 | 72453335 | 72453335 | ARAP1 | intronic | 11q13.4 | . | bnd_180 | transl_inter_2830 |
| 62T | 11 | 72596501 | 72596501 | FCHSD2 | intronic | 11q13.4 | . | bnd_182 | transl_inter_6083 |
| 62T | 11 | 133725263 | 133725263 | . | intergenic | 11q25 | Score=249;Name="908309:L1MC4a(LINE)" | bnd_58 | transl_intra_3505 |

| | | | | | | | | | |
|---|---|---|---|---|---|---|---|---|---|
| 62T | 11 | 133731935 | 133731935 | . | intergenic | 11q25 | Score=4358;Name="908318:L1MB3(LINE)" | bnd_57 | transl_intra_3505 |
| 62T | 12 | 7180156 | 7180156 | . | intergenic | 12p13.31 | Score=185;Name="923694:L3(LINE)" | bnd_100 | tandem_dup_4071 |
| 62T | 12 | 7185134 | 7185134 | . | intergenic | 12p13.31 | Score=1410;Name="923706:AluJo(SINE)" | bnd_99 | tandem_dup_4071 |
| 62T | 12 | 27822990 | 27822990 | PPFIBP1 | intronic | 12p11.22 | . | bnd_102 | tandem_dup_2760 |
| 62T | 12 | 28067906 | 28067906 | . | intergenic | 12p11.22 | . | bnd_101 | tandem_dup_2760 |
| 62T | 12 | 56508535 | 56508535 | . | downstream | 12q13.2 | Score=2096;Name="1005278:AluSx1(SINE)" | bnd_104 | tandem_dup_4158 |
| 62T | 12 | 56637873 | 56637873 | ANKRD52 | exonic | 12q13.3 | . | bnd_103 | tandem_dup_4158 |
| 62T | 12 | 99442787 | 99442787 | ANKS1B | intronic | 12q23.1 | . | bnd_4 | del_2560 |
| 62T | 12 | 100190101 | 100190101 | ANKS1B | intronic | 12q23.1 | Score=4441;Name="1083666:MER44C(DNA)" | bnd_3 | del_2560 |
| 62T | 13 | 32531009 | 32531009 | EEF1DP3 | ncRNA_intronic | 13q13.1 | . | bnd_6 | del_3071 |
| 62T | 13 | 32532399 | 32532399 | EEF1DP3 | ncRNA_intronic | 13q13.1 | Score=2130;Name="1185170:AluSx1(SINE)" | bnd_5 | del_3071 |
| 62T | 14 | 30333885 | 30333885 | PRKD1 | intronic | 14q12 | . | bnd_8 | del_5608 |
| 62T | 14 | 34672759 | 34672759 | . | intergenic | 14q13.1 | . | bnd_7 | del_5608 |
| 62T | 14 | 45630356 | 45630356 | FANCM | intronic | 14q21.2 | . | bnd_184 | transl_inter_2839 |
| 62T | 15 | 41659202 | 41659202 | NUSAP1 | intronic | 15q15.1 | Score=3704;Name="1518355:L1M2c(LINE)" | bnd_106 | tandem_dup_2593 |
| 62T | 15 | 41797229 | 41797229 | LTK | exonic | 15q15.1 | . | bnd_105 | tandem_dup_2593 |
| 62T | 15 | 72632680 | 72632680 | . | intergenic | 15q23 | . | bnd_108 | tandem_dup_3545 |
| 62T | 15 | 72895621 | 72895621 | . | intergenic | 15q24.1 | Score=1674;Name="1579061:AluSx3(SINE)" | bnd_107 | tandem_dup_3545 |
| 62T | 15 | 75887879 | 75887879 | . | intergenic | 15q24.2 | Score=297;Name="1585313:L2c(LINE)" | bnd_110 | tandem_dup_3311 |
| 62T | 15 | 76110470 | 76110470 | . | intergenic | 15q24.2 | Score=1978;Name="1585752:AluJr(SINE)" | bnd_109 | tandem_dup_3311 |
| 62T | 15 | 81288054 | 81288054 | . | intergenic | 15q25.1 | . | bnd_186 | transl_inter_2518 |
| 62T | 15 | 81289869 | 81289869 | . | upstream | 15q25.1 | . | bnd_188 | transl_inter_2037 |
| 62T | 16 | 75082717 | 75082717 | ZNRF1 | intronic | 16q23.1 | . | bnd_112 | tandem_dup_3329 |
| 62T | 16 | 75298609 | 75298609 | BCAR1 | intronic | 16q23.1 | . | bnd_111 | tandem_dup_3329 |
| 62T | 17 | 57584069 | 57584069 | . | intergenic | 17q22 | . | bnd_74 | transl_intra_2387 |
| 62T | 17 | 57584776 | 57584776 | . | intergenic | 17q22 | . | bnd_73 | transl_intra_2387 |
| 62T | 17 | 74613224 | 74613224 | . | intergenic | 17q25.1 | Score=1722;Name="1956193:MER34B(LTR)" | bnd_175 | transl_inter_1846_0 |
| 62T | 18 | 9125386 | 9125386 | NDUFV2 | intronic | 18p11.22 | Score=356;Name="1988054:L1ME3C(LINE)" | bnd_10 | del_2109 |
| 62T | 18 | 9804206 | 9804206 | RAB31 | intronic | 18p11.22 | . | bnd_9 | del_2109 |
| 62T | 18 | 54368437 | 54368437 | WDR7 | intronic | 18q21.31 | . | bnd_12 | del_2941 |
| 62T | 18 | 54529546 | 54529546 | WDR7 | intronic | 18q21.31 | . | bnd_11 | del_2941 |
| 62T | 18 | 54560219 | 54560219 | WDR7 | intronic | 18q21.31 | . | bnd_14 | del_1546 |
| 62T | 18 | 54573910 | 54573910 | WDR7 | intronic | 18q21.31 | Score=2279;Name="2060724:AluSq2(SINE)" | bnd_13 | del_1546 |
| 62T | 18 | 60758969 | 60758969 | . | intergenic | 18q21.33 | Score=329;Name="2071884:(GTGTG)n(Simple_repeat)" | bnd_114 | tandem_dup_6538 |
| 62T | 18 | 60759342 | 60759342 | . | intergenic | 18q21.33 | . | bnd_113 | tandem_dup_6538 |
| 62T | 19 | 5763029 | 5763029 | CATSPERD | intronic | 19p13.3 | Score=989;Name="2112665:(TGGA)n(Simple_repeat)" | bnd_177 | transl_inter_1182_0 |
| 62T | 19 | 16878572 | 16878572 | NWD1 | intronic | 19p13.11 | Score=1101;Name="2142801:THE1C(LTR)" | bnd_190 | transl_inter_4384 |
| 62T | 20 | 1309228 | 1309228 | SDCBP2-AS1,FKBP1A-SDCBP2,SDCBP2 | ncRNA_intronic | 20p13 | Score=197;Name="2651508:MIR3(SINE)" | bnd_76 | transl_intra_4514 |

| | | | | | | | | | |
|---|---|---|---|---|---|---|---|---|---|
| 62T | 20 | 1353724 | 1353724 | SDCBP2-AS1,FKBP1A-SDCBP2,FKBP1A | ncRNA_exonic | 20p13 | . | bnd_75 | transl_intra_4514 |
| 62T | 20 | 1404830 | 1404830 | . | intergenic | 20p13 | Score=899;Name="2651698:L1MA9(LINE)" | bnd_183 | transl_inter_2839 |
| 62T | 20 | 3060697 | 3060697 | . | intergenic | 20p13 | Score=849;Name="2655476:AluJo(SINE)" | bnd_122 | tandem_dup_4088 |
| 62T | 20 | 3171933 | 3171933 | DDRGK1 | intronic | 20p13 | . | bnd_121 | tandem_dup_4088 |
| 62T | 20 | 30183346 | 30183346 | . | intergenic | 20q11.21 | Score=183;Name="2698131:MIR(SINE)" | bnd_124 | tandem_dup_2890 |
| 62T | 20 | 30387444 | 30387444 | TPX2 | intronic | 20q11.21 | Score=2486;Name="2698770:AluSz(SINE)" | bnd_123 | tandem_dup_2890 |
| 62T | 20 | 60837975 | 60837975 | OSBPL2 | intronic | 20q13.33 | . | bnd_194 | transl_inter_5808 |
| 62T | 20 | 61389080 | 61389080 | NTSR1 | intronic | 20q13.33 | . | bnd_126 | tandem_dup_1781 |
| 62T | 20 | 61667347 | 61667347 | RP11-305P22.9,LINC00029 | ncRNA_intronic | 20q13.33 | . | bnd_125 | tandem_dup_1781 |
| 62T | 21 | 45062019 | 45062019 | HSF2BP | intronic | 21q22.3 | Score=1418;Name="2828228:AluJo(SINE)" | bnd_187 | transl_inter_2037 |
| 62T | 21 | 45062324 | 45062324 | HSF2BP | intronic | 21q22.3 | . | bnd_185 | transl_inter_2518 |
| 62T | 22 | 24036254 | 24036254 | RGL4,GUSBP11 | ncRNA_intronic | 22q11.23 | . | bnd_128 | tandem_dup_3380 |
| 62T | 22 | 24202505 | 24202505 | SLC2A11 | intronic | 22q11.23 | Score=2021;Name="2847672:AluJb(SINE)" | bnd_127 | tandem_dup_3380 |
| 62T | 22 | 37626657 | 37626657 | RAC2 | intronic | 22q13.1 | Score=286;Name="2879503:MIRb(SINE)" | bnd_171 | transl_inter_2022 |
| 64T | 3 | 81869713 | 81869713 | . | intergenic | 3p12.2 | Score=2785;Name="3058934:MSTB-int(LTR)" | bnd_20 | del_ins_954 |
| 64T | 3 | 81871425 | 81871425 | . | intergenic | 3p12.2 | . | bnd_19 | del_ins_954 |
| 64T | 3 | 156365178 | 156365178 | . | intergenic | 3q25.31 | . | bnd_65 | transl_inter_5893 |
| 64T | 4 | 67689793 | 67689793 | . | intergenic | 4q13.2 | Score=13809;Name="3373643:L1PB1(LINE)" | bnd_10 | del_613 |
| 64T | 4 | 67692057 | 67692057 | . | intergenic | 4q13.2 | Score=13809;Name="3373645:L1PB1(LINE)" | bnd_9 | del_613 |
| 64T | 5 | 1210859 | 1210859 | SLC6A19 | intronic | 5p15.33 | . | bnd_12 | del_319 |
| 64T | 5 | 1233917 | 1233917 | SLC6A18 | intronic | 5p15.33 | Score=2528;Name="3581030:AluY(SINE)" | bnd_11 | del_319 |
| 64T | 5 | 3016867 | 3016867 | . | intergenic | 5p15.33 | Score=3645;Name="3583268:L1ME2(LINE)" | bnd_40 | del_invers_541_0/1083_0 |
| 64T | 5 | 7877012 | 7877012 | MTRR | intronic | 5p15.31 | . | bnd_44 | transl_intra_1124_0 |
| 64T | 5 | 7877024 | 7877024 | MTRR | intronic | 5p15.31 | . | bnd_29 | del_insou_573_0/1117_0 |
| 64T | 5 | 7955264 | 7955264 | . | intergenic | 5p15.31 | . | bnd_27 | del_insou_573_0/1117_0 |
| 64T | 5 | 7955277 | 7955277 | . | intergenic | 5p15.31 | . | bnd_41 | del_invers_541_0/1083_0 |
| 64T | 5 | 9332361 | 9332361 | SEMA5A | intronic | 5p15.31 | . | bnd_39 | del_invers_541_0/1083_0 |
| 64T | 5 | 9332437 | 9332437 | SEMA5A | intronic | 5p15.31 | . | bnd_35 | del_inssu_534_0/1040_0 |
| 64T | 5 | 9341806 | 9341806 | SEMA5A | intronic | 5p15.31 | . | bnd_24 | del_insod_1234_0/2190_0 |
| 64T | 5 | 10092142 | 10092142 | . | intergenic | 5p15.2 | . | bnd_46 | transl_intra_857_0 |
| 64T | 5 | 10092551 | 10092551 | . | intergenic | 5p15.2 | Score=238;Name="3593327:L1P4(LINE)" | bnd_26 | del_insod_1234_0/2190_0 |
| 64T | 5 | 10102963 | 10102963 | . | intergenic | 5p15.2 | Score=454;Name="3593346:L1M5(LINE)" | bnd_37 | del_inssu_534_0/1040_0 |
| 64T | 5 | 11233391 | 11233391 | CTNND2 | intronic | 5p15.2 | . | bnd_48 | transl_intra_1150_0 |
| 64T | 5 | 12733839 | 12733839 | LINC01194 | ncRNA_intronic | 5p15.2 | . | bnd_14 | del_685 |
| 64T | 5 | 12733893 | 12733893 | LINC01194 | ncRNA_intronic | 5p15.2 | Score=528;Name="3597657:FLAM_A(SINE)" | bnd_42 | del_invers_541_0/1083_0 |
| 64T | 5 | 13825607 | 13825607 | DNAH5 | intronic | 5p15.2 | Score=3334;Name="3599413:L1MB2(LINE)" | bnd_45 | transl_intra_857_0 |

| | | | | | | | | | |
|---|---|---|---|---|---|---|---|---|---|
| 64T | 5 | 13825816 | 13825816 | DNAH5 | intronic | 5p15.2 | Score=3334;Name="3599413:L1MB2(LINE)" | bnd_33 | del_insou_721_0/1371_0 |
| 64T | 5 | 13827560 | 13827560 | DNAH5 | intronic | 5p15.2 | Score=3875;Name="3599415:THE1C-int(LTR)" | bnd_16 | del_1203 |
| 64T | 5 | 15724280 | 15724280 | FBXL7 | intronic | 5p15.1 | Score=577;Name="3602452:L1M4c(LINE)" | bnd_31 | del_insou_721_0/1371_0 |
| 64T | 5 | 15774077 | 15774077 | FBXL7 | intronic | 5p15.1 | . | bnd_28 | del_insou_573_0/1117_0 |
| 64T | 5 | 15774218 | 15774218 | FBXL7 | intronic | 5p15.1 | Score=865;Name="3602541:MLT1D(LTR)" | bnd_30 | del_insou_573_0/1117_0 |
| 64T | 5 | 15896194 | 15896194 | FBXL7 | intronic | 5p15.1 | Score=2022;Name="3602733:AluSz(SINE)" | bnd_25 | del_insod_1234_0/2190_0 |
| 64T | 5 | 15960497 | 15960497 | . | intergenic | 5p15.1 | Score=280;Name="3602829:L1M6(LINE)" | bnd_36 | del_inssu_534_0/1040_0 |
| 64T | 5 | 15960744 | 15960744 | . | intergenic | 5p15.1 | Score=280;Name="3602829:L1M6(LINE)" | bnd_43 | transl_intra_1124_0 |
| 64T | 5 | 15960884 | 15960884 | . | intergenic | 5p15.1 | Score=2230;Name="3602830:MLT2B3(LTR)" | bnd_13 | del_685 |
| 64T | 5 | 15979060 | 15979060 | . | intergenic | 5p15.1 | Score=4107;Name="3602871:L1MC4a(LINE)" | bnd_32 | del_insou_721_0/1371_0 |
| 64T | 5 | 16134447 | 16134447 | 11-Mar | intronic | 5p15.1 | Score=788;Name="3603122:MSTD(LTR)" | bnd_15 | del_1203 |
| 64T | 5 | 16319425 | 16319425 | . | intergenic | 5p15.1 | Score=1829;Name="3603448:L1MA8(LINE)" | bnd_47 | transl_intra_1150_0 |
| 64T | 5 | 16331761 | 16331761 | . | intergenic | 5p15.1 | Score=12222;Name="3603471:L1MC1(LINE)" | bnd_38 | del_inssu_534_0/1040_0 |
| 64T | 5 | 16606150 | 16606150 | FAM134B | intronic | 5p15.1 | Score=272;Name="3603929:LTR81B(LTR)" | bnd_34 | del_insou_721_0/1371_0 |
| 64T | 5 | 17464321 | 17464321 | . | intergenic | 5p15.1 | Score=10586;Name="3605758:L1PA8A(LINE)" | bnd_23 | del_insod_1234_0/2190_0 |
| 64T | 5 | 162758478 | 162758478 | . | intergenic | 5q34 | Score=3991;Name="3850429:LTR10D(LTR)" | bnd_18 | del_1925 |
| 64T | 5 | 162759674 | 162759674 | . | intergenic | 5q34 | Score=391;Name="3850431:L2(LINE)" | bnd_17 | del_1925 |
| 64T | 6 | 37166475 | 37166475 | . | intergenic | 6p21.2 | Score=2009;Name="3957087:L1PB4(LINE)" | bnd_72 | transl_inter_2059 |
| 64T | 7 | 87342859 | 87342859 | RUNDC3B | intronic | 7q21.12 | . | bnd_54 | tandem_dup_1347 |
| 64T | 7 | 87343062 | 87343062 | RUNDC3B | intronic | 7q21.12 | . | bnd_22 | del_ins_3980_0 |
| 64T | 7 | 99716557 | 99716557 | TAF6 | UTR5 | 7q22.1 | . | bnd_21 | del_ins_3980_0 |
| 64T | 7 | 99771721 | 99771721 | GPC2 | intronic | 7q22.1 | . | bnd_53 | tandem_dup_1347 |
| 64T | 7 | 100071910 | 100071910 | TSC22D4 | exonic | 7q22.1 | . | bnd_71 | transl_inter_2059 |
| 64T | 8 | 54171425 | 54171425 | . | intergenic | 8q11.23 | . | bnd_74 | transl_inter_749 |
| 64T | 9 | 31598986 | 31598986 | . | intergenic | 9p21.1 | . | bnd_73 | transl_inter_749 |
| 64T | 9 | 130672963 | 130672963 | ST6GALNAC4 | intronic | 9q34.11 | . | bnd_61 | transl_inter_4838 |
| 64T | 9 | 130673448 | 130673448 | ST6GALNAC4 | intronic | 9q34.11 | Score=672;Name="4962525:L2a(LINE)" | bnd_56 | tandem_dup_1141 |
| 64T | 9 | 130673460 | 130673460 | ST6GALNAC4 | intronic | 9q34.11 | Score=672;Name="4962525:L2a(LINE)" | bnd_59 | transl_inter_1030_0 |
| 64T | 9 | 130719428 | 130719428 | FAM102A | intronic | 9q34.11 | Score=1037;Name="4962634:AluSz(SINE)" | bnd_63 | transl_inter_808 |
| 64T | 9 | 130719843 | 130719843 | FAM102A | intronic | 9q34.11 | Score=4648;Name="4962635:L1MB7(LINE)" | bnd_69 | transl_inter_880 |
| 64T | 9 | 130746694 | 130746694 | . | intergenic | 9q34.11 | Score=2082;Name="4962692:AluSq2(SINE)" | bnd_50 | transl_intra_4401_0 |

| | | | | | | | | |
|---|---|---|---|---|---|---|---|---|
| 64T 9 | 130974849 | 130974849 | DNM1 | intronic | 9q34.11 | . | bnd_49 | transl_intra_4401_0 |
| 64T 9 | 131022236 | 131022236 | GOLGA2 | intronic | 9q34.11 | . | bnd_67 | transl_inter_1853 |
| 64T 9 | 131022585 | 131022585 | GOLGA2 | intronic | 9q34.11 | . | bnd_55 | tandem_dup_1141 |
| 64T 11 | 128351928 | 128351928 | ETS1 | intronic | 11q24.3 | . | bnd_2 | del_563 |
| 64T 11 | 128355698 | 128355698 | ETS1 | intronic | 11q24.3 | . | bnd_1 | del_563 |
| 64T 12 | 131452046 | 131452046 | GPR133 | intronic | 12q24.33 | . | bnd_4 | del_1228 |
| 64T 12 | 131462760 | 131462760 | GPR133 | intronic | 12q24.33 | Score=316;Name="1156353:L1M2(LINE)" | bnd_3 | del_1228 |
| 64T 13 | 93116400 | 93116400 | GPC5 | intronic | 13q31.3 | . | bnd_58 | transl_inter_1290 |
| 64T 14 | 55233302 | 55233302 | SAMD4A | intronic | 14q22.2 | . | bnd_60 | transl_inter_1030_0 |
| 64T 14 | 55233620 | 55233620 | SAMD4A | intronic | 14q22.2 | . | bnd_62 | transl_inter_4838 |
| 64T 14 | 55234110 | 55234110 | SAMD4A | intronic | 14q22.2 | . | bnd_64 | transl_inter_808 |
| 64T 14 | 68886077 | 68886077 | RAD51B | intronic | 14q24.1 | . | bnd_52 | tandem_dup_1940 |
| 64T 14 | 69054460 | 69054460 | RAD51B | intronic | 14q24.1 | . | bnd_51 | tandem_dup_1940 |
| 64T 15 | 32975352 | 32975352 | SCG5 | intronic | 15q13.3 | . | bnd_6 | del_1701 |
| 64T 15 | 32976937 | 32976937 | SCG5 | intronic | 15q13.3 | . | bnd_5 | del_1701 |
| 64T 15 | 94764344 | 94764344 | . | intergenic | 15q26.2 | . | bnd_57 | transl_inter_1290 |
| 64T 16 | 359445 | 359445 | AXIN1 | intronic | 16p13.3 | . | bnd_8 | del_1930 |
| 64T 16 | 361408 | 361408 | AXIN1 | intronic | 16p13.3 | . | bnd_7 | del_1930 |
| 64T 18 | 60083458 | 60083458 | . | intergenic | 18q21.33 | . | bnd_66 | transl_inter_5893 |
| 64T 18 | 60137507 | 60137507 | . | intergenic | 18q21.33 | Score=21056;Name="2070676:L1MA2(LINE)" | bnd_68 | transl_inter_1853 |
| 64T 18 | 60224351 | 60224351 | ZCCHC2 | intronic | 18q21.33 | Score=267;Name="2070820:L2c(LINE)" | bnd_70 | transl_inter_880 |
| 65T 1 | 1002595 | 1002595 | . | intergenic | 1p36.33 | . | bnd_130 | transl_inter_729 |
| 65T 1 | 1117642 | 1117642 | TTLL10 | intronic | 1p36.33 | . | bnd_64 | transl_intra_70_0 |
| 65T 1 | 2288932 | 2288932 | MORN1 | exonic | 1p36.33 | . | bnd_66 | transl_intra_410_0 |
| 65T 1 | 2291301 | 2291301 | MORN1 | intronic | 1p36.33 | Score=2180;Name="3694:AluSx(SINE)" | bnd_65 | transl_intra_410_0 |
| 65T 1 | 2386056 | 2386056 | . | intergenic | 1p36.32 | . | bnd_60 | transl_intra_4840_0 |
| 65T 1 | 2715360 | 2715360 | . | intergenic | 1p36.32 | Score=362;Name="4050:(CAGC)n(Simple_repeat)" | bnd_2 | del_105 |
| 65T 1 | 2716715 | 2716715 | . | intergenic | 1p36.32 | . | bnd_1 | del_105 |
| 65T 1 | 2907018 | 2907018 | . | intergenic | 1p36.32 | . | bnd_106 | tandem_dup_5537_0 |
| 65T 1 | 2907028 | 2907028 | . | intergenic | 1p36.32 | . | bnd_4 | del_276 |
| 65T 1 | 2907586 | 2907586 | . | intergenic | 1p36.32 | Score=281;Name="4278:L1M5(LINE) | bnd_68 | transl_intra_454_0 |
| 65T 1 | 4088014 | 4088014 | . | intergenic | 1p36.32 | . | bnd_105 | tandem_dup_5537_0 |
| 65T 1 | 7051335 | 7051335 | CAMTA1 | intronic | 1p36.31 | Score=2698;Name="10888:L1ME2z(LINE)" | bnd_6 | del_900 |
| 65T 1 | 7051379 | 7051379 | CAMTA1 | intronic | 1p36.31 | Score=2698;Name="10888:L1ME2z(LINE)" | bnd_3 | del_276 |
| 65T 1 | 7051638 | 7051638 | CAMTA1 | intronic | 1p36.31 | Score=2698;Name="10888:L1ME2z(LINE)" | bnd_59 | transl_intra_4840_0 |
| 65T 1 | 7051721 | 7051721 | CAMTA1 | intronic | 1p36.31 | Score=2698;Name="10888:L1ME2z(LINE)" | bnd_5 | del_900 |
| 65T 1 | 7108567 | 7108567 | CAMTA1 | intronic | 1p36.31 | . | bnd_63 | transl_intra_70_0 |
| 65T 1 | 9737183 | 9737183 | PIK3CD | intronic | 1p36.22 | . | bnd_132 | transl_inter_3630 |
| 65T 1 | 9739126 | 9739126 | PIK3CD | intronic | 1p36.22 | Score=2563;Name="16677:AluSx(SINE)" | bnd_134 | transl_inter_1317 |
| 65T 1 | 9745947 | 9745947 | PIK3CD | intronic | 1p36.22 | . | bnd_136 | transl_inter_3137 |
| 65T 1 | 10524919 | 10524919 | DFFA | intronic | 1p36.22 | Score=564;Name="18666:L2a(LINE)" | bnd_56 | del_5764_0 |
| 65T 1 | 10746509 | 10746509 | CASZ1 | intronic | 1p36.22 | . | bnd_55 | del_5764_0 |
| 65T 1 | 12866953 | 12866953 | . | intergenic | 1p36.21 | . | bnd_67 | transl_intra_454_0 |
| 65T 1 | 68716958 | 68716958 | . | intergenic | 1p31.3 | Score=787;Name="151491:L1ME2z(LINE)" | bnd_88 | transl_intra_4443_0 |
| 65T 1 | 68724889 | 68724889 | . | intergenic | 1p31.3 | Score=2538;Name="151510:AluSx1(SINE)" | bnd_138 | transl_inter_1601 |
| 65T 1 | 71965234 | 71965234 | NEGR1 | intronic | 1p31.1 | Score=261;Name="157213:L2a(LINE)" | bnd_108 | tandem_dup_3551_0 |
| 65T 1 | 71966135 | 71966135 | NEGR1 | intronic | 1p31.1 | . | bnd_70 | transl_intra_2079_0 |
| 65T 1 | 72976275 | 72976275 | . | intergenic | 1p31.1 | . | bnd_110 | tandem_dup_1047_0 |

| | | | | | | | | |
|---|---|---|---|---|---|---|---|---|
| 65T | 1 | 73057193 | 73057193 | . | intergenic | 1p31.1 | . | bnd_62 | transl_intra_3753_0 |
| 65T | 1 | 73146649 | 73146649 | . | intergenic | 1p31.1 | . | bnd_140 | transl_inter_2203 |
| 65T | 1 | 73285183 | 73285183 | . | intergenic | 1p31.1 | . | bnd_50 | del_2952_0 |
| 65T | 1 | 76032906 | 76032906 | SLC44A5 | intronic | 1p31.1 | . | bnd_87 | transl_intra_4443_0 |
| 65T | 1 | 76033958 | 76033958 | SLC44A5 | intronic | 1p31.1 | . | bnd_109 | tandem_dup_1047_0 |
| 65T | 1 | 76518166 | 76518166 | . | intergenic | 1p31.1 | . | bnd_112 | tandem_dup_2586_0 |
| 65T | 1 | 77101019 | 77101019 | . | intergenic | 1p31.1 | . | bnd_114 | tandem_dup_2310_0 |
| 65T | 1 | 77321949 | 77321949 | . | intergenic | 1p31.1 | . | bnd_8 | del_4033 |
| 65T | 1 | 77448375 | 77448375 | ST6GALNAC5 | intronic | 1p31.1 | Score=24004;Name="166139:L1PA6(LINE)" | bnd_72 | transl_intra_2002_0 |
| 65T | 1 | 77451937 | 77451937 | ST6GALNAC5 | intronic | 1p31.1 | Score=24004;Name="166139:L1PA6(LINE)" | bnd_71 | transl_intra_2002_0 |
| 65T | 1 | 79736318 | 79736318 | . | intergenic | 1p31.1 | Score=7132;Name="170238:L1PA12(LINE)" | bnd_10 | del_4431 |
| 65T | 1 | 79737266 | 79737266 | . | intergenic | 1p31.1 | Score=7132;Name="170238:L1PA12(LINE)" | bnd_9 | del_4431 |
| 65T | 1 | 81170039 | 81170039 | . | intergenic | 1p31.1 | . | bnd_49 | del_2952_0 |
| 65T | 1 | 85148560 | 85148560 | SSX2IP | intronic | 1p22.3 | . | bnd_12 | del_235 |
| 65T | 1 | 86158517 | 86158517 | ZNHIT6 | intronic | 1p22.3 | . | bnd_61 | transl_intra_3753_0 |
| 65T | 1 | 86399699 | 86399699 | COL24A1 | intronic | 1p22.3 | Score=1202;Name="181329:MLT1F1(LTR)" | bnd_7 | del_4033 |
| 65T | 1 | 88994219 | 88994219 | . | intergenic | 1p22.2 | . | bnd_11 | del_235 |
| 65T | 1 | 94803433 | 94803433 | . | intergenic | 1p21.3 | Score=2440;Name="196416:AluSc(SINE)" | bnd_14 | del_1405 |
| 65T | 1 | 96187019 | 96187019 | . | intergenic | 1p21.3 | . | bnd_42 | del_ins_1191 |
| 65T | 1 | 96830690 | 96830690 | . | intergenic | 1p21.3 | . | bnd_116 | tandem_dup_3904_0 |
| 65T | 1 | 97460947 | 97460947 | . | intergenic | 1p21.3 | . | bnd_142 | transl_inter_2788 |
| 65T | 1 | 99445453 | 99445453 | LPPR5 | intronic | 1p21.3 | . | bnd_118 | tandem_dup_3876_0 |
| 65T | 1 | 99446452 | 99446452 | LPPR5 | intronic | 1p21.3 | . | bnd_117 | tandem_dup_3876_0 |
| 65T | 1 | 100700325 | 100700325 | DBT | intronic | 1p21.2 | . | bnd_111 | tandem_dup_2586_0 |
| 65T | 1 | 101481412 | 101481412 | DPH5 | intronic | 1p21.2 | Score=14488;Name="208067:Tigger1(DNA)" | bnd_115 | tandem_dup_3904_0 |
| 65T | 1 | 104473306 | 104473306 | . | intergenic | 1p21.1 | . | bnd_41 | del_ins_1191 |
| 65T | 1 | 105101765 | 105101765 | . | intergenic | 1p21.1 | Score=23145;Name="213980:HERVL-int(LTR)" | bnd_13 | del_1405 |
| 65T | 1 | 105448504 | 105448504 | . | intergenic | 1p21.1 | Score=450;Name="214516:(TA)n(Simple_repeat)" | bnd_90 | transl_intra_1497_0 |
| 65T | 1 | 105819248 | 105819248 | . | intergenic | 1p21.1 | Score=2162;Name="215168:MSTB(LTR)" | bnd_113 | tandem_dup_2310_0 |
| 65T | 1 | 107919891 | 107919891 | NTNG1 | intronic | 1p13.3 | . | bnd_107 | tandem_dup_3551_0 |
| 65T | 1 | 110677686 | 110677686 | . | intergenic | 1p13.3 | Score=546;Name="223537:MIRb(SINE)" | bnd_144 | transl_inter_1446 |
| 65T | 1 | 114286869 | 114286869 | PHTF1 | intronic | 1p13.2 | Score=982;Name="230355:LTR84a(LTR)" | bnd_89 | transl_intra_1497_0 |
| 65T | 1 | 114781203 | 114781203 | . | intergenic | 1p13.2 | . | bnd_69 | transl_intra_2079_0 |
| 65T | 1 | 165731227 | 165731227 | TMCO1 | intronic | 1q24.1 | . | bnd_16 | del_2898 |
| 65T | 1 | 165731508 | 165731508 | TMCO1 | intronic | 1q24.1 | . | bnd_15 | del_2898 |
| 65T | 1 | 180463578 | 180463578 | ACBD6 | intronic | 1q25.3 | Score=754;Name="309551:Charlie10a(DNA)" | bnd_146 | transl_inter_7640 |
| 65T | 1 | 193052732 | 193052732 | TROVE2 | intronic | 1q31.2 | Score=924;Name="331340:MER5A(DNA)" | bnd_148 | transl_inter_8165 |
| 65T | 1 | 197146056 | 197146056 | ZBTB41 | intronic | 1q31.3 | . | bnd_150 | transl_inter_7545 |
| 65T | 1 | 204175783 | 204175783 | GOLT1A | intronic | 1q32.1 | . | bnd_18 | del_4495 |
| 65T | 1 | 204196984 | 204196984 | PLEKHA6 | intronic | 1q32.1 | . | bnd_17 | del_4495 |
| 65T | 1 | 246677032 | 246677032 | . | intergenic | 1q44 | . | bnd_20 | del_4678 |
| 65T | 1 | 246678656 | 246678656 | . | downstream | 1q44 | Score=613;Name="428614:L1ME1(LINE)" | bnd_19 | del_4678 |
| 65T | 2 | 5658260 | 5658260 | . | intergenic | 2p25.2 | Score=2512;Name="2241935:AluY(SINE)" | bnd_54 | del_inssd_1475_0/2039_0 |

| | | | | | | | | |
|---|---|---|---|---|---|---|---|---|
| 65T 2 | 5659597 | 5659597 | . | intergenic | 2p25.2 | Score=520;Name="2241938:L2(LINE)" | bnd_52 | del_inssd_1475_0/2039_0 |
| 65T 2 | 5674698 | 5674698 | . | intergenic | 2p25.2 | . | bnd_53 | del_inssd_1475_0/2039_0 |
| 65T 2 | 5675893 | 5675893 | . | intergenic | 2p25.2 | . | bnd_51 | del_inssd_1475_0/2039_0 |
| 65T 2 | 190761405 | 190761405 | . | intergenic | 2q32.2 | . | bnd_30 | del_5727 |
| 65T 2 | 190796007 | 190796007 | . | intergenic | 2q32.2 | Score=428;Name="2556084:L1MC4a(LINE)" | bnd_29 | del_5727 |
| 65T 2 | 230724417 | 230724417 | TRIP12 | intronic | 2q36.3 | . | bnd_122 | tandem_dup_2015 |
| 65T 2 | 230725066 | 230725066 | TRIP12 | intronic | 2q36.3 | . | bnd_121 | tandem_dup_2015 |
| 65T 2 | 240564410 | 240564410 | . | intergenic | 2q37.3 | Score=532;Name="2644456:(TG)n(Simple_repeat)" | bnd_58 | tandem_dup_2324_0 |
| 65T 2 | 240564785 | 240564785 | . | intergenic | 2q37.3 | . | bnd_57 | tandem_dup_2324_0 |
| 65T 3 | 93715512 | 93715512 | ARL13B | intronic | 3q11.1 | Score=892;Name="3072446:AluJr(SINE)" | bnd_166 | transl_inter_7486 |
| 65T 4 | 26172230 | 26172230 | . | intergenic | 4p15.2 | . | bnd_153 | transl_inter_7422 |
| 65T 4 | 31818007 | 31818007 | . | intergenic | 4p15.1 | Score=468;Name="3315593:(TATAA)n(Simple_repeat)" | bnd_78 | transl_intra_2049_0 |
| 65T 4 | 31859369 | 31859369 | . | intergenic | 4p15.1 | Score=5689;Name="3315642:L1P4a(LINE)" | bnd_77 | transl_intra_2049_0 |
| 65T 4 | 38969813 | 38969813 | TMEM156 | intronic | 4p14 | . | bnd_98 | transl_intra_140 |
| 65T 4 | 38970747 | 38970747 | TMEM156 | intronic | 4p14 | Score=3188;Name="3328072:L1MA4A(LINE)" | bnd_97 | transl_intra_140 |
| 65T 4 | 39018858 | 39018858 | TMEM156 | intronic | 4p14 | Score=2458;Name="3328188:AluY(SINE)" | bnd_124 | tandem_dup_7 |
| 65T 4 | 39042422 | 39042422 | . | intergenic | 4p14 | Score=2299;Name="3328243:AluSc(SINE)" | bnd_123 | tandem_dup_7 |
| 65T 4 | 39152044 | 39152044 | MIR1273H | ncRNA_intronic | 4p14 | . | bnd_131 | transl_inter_3630 |
| 65T 4 | 39274617 | 39274617 | WDR19,MIR1273H | exonic | 4p14 | . | bnd_135 | transl_inter_3137 |
| 65T 4 | 39336048 | 39336048 | MIR1273H,RFC1 | ncRNA_intronic | 4p14 | . | bnd_80 | transl_intra_2972_0 |
| 65T 4 | 39338479 | 39338479 | MIR1273H,RFC1 | ncRNA_intronic | 4p14 | Score=355;Name="3328880:L3(LINE)" | bnd_79 | transl_intra_2972_0 |
| 65T 4 | 39614362 | 39614362 | SMIM14 | intronic | 4p14 | Score=1798;Name="3329574:AluJb(SINE)" | bnd_100 | transl_intra_679 |
| 65T 4 | 39615331 | 39615331 | SMIM14 | intronic | 4p14 | . | bnd_99 | transl_intra_679 |
| 65T 4 | 39925057 | 39925057 | PDS5A | intronic | 4p14 | . | bnd_168 | transl_inter_6099 |
| 65T 4 | 41751077 | 41751077 | . | upstream | 4p13 | . | bnd_82 | transl_intra_1509_0 |
| 65T 4 | 41751834 | 41751834 | . | upstream | 4p13 | . | bnd_81 | transl_intra_1509_0 |
| 65T 4 | 129076182 | 129076182 | LARP1B | intronic | 4q28.2 | Score=2155;Name="3475421:AluSx1(SINE)" | bnd_102 | transl_intra_3036 |
| 65T 4 | 129078213 | 129078213 | LARP1B | intronic | 4q28.2 | Score=2692;Name="3475426:L1ME3A(LINE)" | bnd_101 | transl_intra_3036 |
| 65T 4 | 129196828 | 129196828 | PGRMC2 | intronic | 4q28.2 | . | bnd_84 | transl_intra_3258_0 |
| 65T 4 | 129198100 | 129198100 | PGRMC2 | intronic | 4q28.2 | Score=1902;Name="3475660:AluY(SINE)" | bnd_83 | transl_intra_3258_0 |
| 65T 4 | 166264163 | 166264163 | MSMO1 | UTR3 | 4q32.3 | . | bnd_86 | transl_intra_2977_0 |
| 65T 4 | 167440318 | 167440318 | . | intergenic | 4q32.3 | . | bnd_85 | transl_intra_2977_0 |
| 65T 4 | 183133366 | 183133366 | . | intergenic | 4q34.3 | . | bnd_159 | transl_inter_1598 |
| 65T 5 | 4632855 | 4632855 | . | intergenic | 5p15.32 | Score=2899;Name="3585304:L1PA10(LINE)" | bnd_170 | transl_inter_1102 |
| 65T 5 | 39474601 | 39474601 | . | intergenic | 5p13.1 | . | bnd_145 | transl_inter_7640 |
| 65T 5 | 91295857 | 91295857 | . | intergenic | 5q14.3 | . | bnd_34 | del_5603 |
| 65T 5 | 91331712 | 91331712 | . | intergenic | 5q14.3 | . | bnd_33 | del_5603 |
| 65T 5 | 100094288 | 100094288 | . | intergenic | 5q21.1 | Score=505;Name="3739500:MIRb(SINE)" | bnd_172 | transl_inter_7505 |
| 65T 5 | 113922205 | 113922205 | . | intergenic | 5q22.3 | Score=9610;Name="3761162:L1M2(LINE)" | bnd_161 | transl_inter_7657 |

| | | | | | | | | | |
|---|---|---|---|---|---|---|---|---|---|
| 65T | 5 | 161632698 | 161632698 | . | intergenic | 5q34 | Score=2506;Name="3848583:L1MA10(LINE)" | bnd_155 | transl_inter_7547 |
| 65T | 6 | 142584280 | 142584280 | . | intergenic | 6q24.1 | Score=5134;Name="4127695:L1PA16(LINE)" | bnd_163 | transl_inter_7673 |
| 65T | 6 | 143684085 | 143684085 | . | intergenic | 6q24.2 | Score=3931;Name="4129448:FordPrefect(DNA)" | bnd_36 | del_1365 |
| 65T | 6 | 143691061 | 143691061 | . | intergenic | 6q24.2 | Score=1594;Name="4129463:AluJr(SINE)" | bnd_35 | del_1365 |
| 65T | 6 | 144007317 | 144007317 | PHACTR2 | intronic | 6q24.2 | Score=360;Name="4130047:L1ME4a(LINE)" | bnd_151 | transl_inter_7368 |
| 65T | 6 | 138725073 | 138725073 | . | upstream | 6q23.3 | . | bnd_174 | transl_inter_7530 |
| 65T | 7 | 71522477 | 71522477 | CALN1 | intronic | 7q11.22 | Score=773;Name="4348490:L1M6(LINE)" | bnd_126 | tandem_dup_429_0 |
| 65T | 7 | 93070536 | 93070536 | CALCR | intronic | 7q21.3 | . | bnd_125 | tandem_dup_429_0 |
| 65T | 7 | 121550440 | 121550440 | PTPRZ1 | intronic | 7q31.32 | Score=1068;Name="4439478:L2a(LINE)" | bnd_128 | tandem_dup_3947 |
| 65T | 7 | 121592572 | 121592572 | PTPRZ1 | intronic | 7q31.32 | Score=1688;Name="4439542:AluJr(SINE)" | bnd_127 | tandem_dup_3947 |
| 65T | 8 | 1861378 | 1861378 | ARHGEF10 | intronic | 8p23.3 | . | bnd_173 | transl_inter_7530 |
| 65T | 8 | 42603930 | 42603930 | . | intergenic | 8p11.21 | Score=2381;Name="4581822:AluSc(SINE)" | bnd_44 | del_ins_5287 |
| 65T | 8 | 42612148 | 42612148 | CHRNA6 | exonic | 8p11.21 | . | bnd_43 | del_ins_5287 |
| 65T | 8 | 71732534 | 71732534 | . | intergenic | 8q13.3 | Score=1344;Name="4624461:MSTB1(LTR)" | bnd_171 | transl_inter_7505 |
| 65T | 8 | 115126292 | 115126292 | . | intergenic | 8q23.3 | . | bnd_169 | transl_inter_1102 |
| 65T | 8 | 127915116 | 127915116 | . | intergenic | 8q24.21 | . | bnd_38 | del_4455 |
| 65T | 8 | 127933886 | 127933886 | . | intergenic | 8q24.21 | Score=2360;Name="4723942:AluY(SINE)" | bnd_37 | del_4455 |
| 65T | 9 | 17031210 | 17031210 | . | intergenic | 9p22.2 | Score=2336;Name="4787477:AluY(SINE)" | bnd_157 | transl_inter_7316 |
| 65T | 9 | 37645977 | 37645977 | . | intergenic | 9p13.2 | Score=431;Name="4823482:MIR3(SINE)" | bnd_129 | transl_inter_729 |
| 65T | 9 | 37687539 | 37687539 | FRMPD1 | intronic | 9p13.2 | . | bnd_133 | transl_inter_1317 |
| 65T | 9 | 37688741 | 37688741 | FRMPD1 | intronic | 9p13.2 | Score=836;Name="4823578:L1ME4a(LINE)" | bnd_167 | transl_inter_6099 |
| 65T | 9 | 90192170 | 90192170 | DAPK1 | intronic | 9q21.33 | Score=949;Name="4880934:MER5A(DNA)" | bnd_147 | transl_inter_8165 |
| 65T | 9 | 113986887 | 113986887 | . | intergenic | 9q31.3 | . | bnd_40 | del_2678 |
| 65T | 9 | 113987527 | 113987527 | . | intergenic | 9q31.3 | Score=722;Name="4925889:MLT1H(LTR)" | bnd_39 | del_2678 |
| 65T | 9 | 122281263 | 122281263 | . | intergenic | 9q33.1 | Score=936;Name="4943927:(TA)n(Simple_repeat)" | bnd_46 | del_ins_5464 |
| 65T | 9 | 122282237 | 122282237 | . | intergenic | 9q33.1 | Score=442;Name="4943931:MIRb(SINE)" | bnd_45 | del_ins_5464 |
| 65T | 9 | 133477649 | 133477649 | FUBP3 | intronic | 9q34.11 | Score=668;Name="4969418:Tigger7(DNA)" | bnd_104 | transl_intra_3090_0 |
| 65T | 9 | 133492985 | 133492985 | FUBP3 | intronic | 9q34.11 | . | bnd_103 | transl_intra_3090_0 |
| 65T | 11 | 8940137 | 8940137 | AKIP1 | intronic | 11p15.4 | . | bnd_152 | transl_inter_7368 |
| 65T | 11 | 16018230 | 16018230 | SOX6 | intronic | 11p15.2 | Score=2166;Name="698146:L1MEf(LINE)" | bnd_154 | transl_inter_7422 |
| 65T | 11 | 42071398 | 42071398 | . | intergenic | 11p12 | . | bnd_48 | transl_intra_3557_0 |
| 65T | 11 | 42280984 | 42280984 | . | intergenic | 11p12 | Score=399;Name="745184:LTR89(LTR)" | bnd_47 | transl_intra_3557_0 |
| 65T | 11 | 121760617 | 121760617 | . | intergenic | 11q24.1 | . | bnd_149 | transl_inter_7545 |
| 65T | 12 | 89119040 | 89119040 | . | intergenic | 12q21.33 | . | bnd_156 | transl_inter_7547 |

| | | | | | | | | |
|---|---|---|---|---|---|---|---|---|
| 65T 13 | 57634726 | 57634726 | . | intergenic | 13q21.1 | . | bnd_22 | del_2725 |
| 65T 13 | 57641131 | 57641131 | . | intergenic | 13q21.1 | . | bnd_21 | del_2725 |
| 65T 15 | 65208681 | 65208681 | ANKDD1A | intronic | 15q22.31 | Score=2075;Name="1562655:AluJo(SINE)" | bnd_158 | transl_inter_7316 |
| 65T 15 | 65967506 | 65967506 | DENND4A | intronic | 15q22.31 | Score=681;Name="1564505:MER58C(DNA)" | bnd_120 | tandem_dup_3987 |
| 65T 15 | 65973149 | 65973149 | DENND4A | intronic | 15q22.31 | Score=12559;Name="1564520:Tigger1(DNA)" | bnd_119 | tandem_dup_3987 |
| 65T 17 | 29057 | 29057 | DOC2B | intronic | 17p13.3 | . | bnd_74 | transl_intra_1028 |
| 65T 17 | 12792567 | 12792567 | ARHGAP44 | intronic | 17p12 | Score=1497;Name="1832401:MSTD(LTR)" | bnd_160 | transl_inter_1598 |
| 65T 17 | 28473630 | 28473630 | NSRP1 | intronic | 17q11.2 | . | bnd_162 | transl_inter_7657 |
| 65T 17 | 68141092 | 68141092 | . | intergenic | 17q24.3 | . | bnd_24 | del_4808 |
| 65T 17 | 68610510 | 68610510 | . | intergenic | 17q24.3 | Score=6909;Name="1944193:Tigger3b(DNA)" | bnd_23 | del_4808 |
| 65T 17 | 75087662 | 75087662 | SEC14L1,LINC00338 | ncRNA_intronic | 17q25.2 | Score=624;Name="1957380:MER3(DNA)" | bnd_26 | del_4012 |
| 65T 17 | 75101389 | 75101389 | SEC14L1 | intronic | 17q25.2 | . | bnd_25 | del_4012 |
| 65T 17 | 30127 | 30127 | DOC2B | intronic | 17p13.3 | . | bnd_73 | transl_intra_1028 |
| 65T 18 | 7699823 | 7699823 | PTPRM | intronic | 18p11.23 | Score=1505;Name="1985681:L1M5(LINE)" | bnd_28 | del_5682 |
| 65T 18 | 8055915 | 8055915 | PTPRM | intronic | 18p11.23 | . | bnd_27 | del_5682 |
| 65T 20 | 38416725 | 38416725 | . | intergenic | 20q12 | Score=733;Name="2719299:L1MEe(LINE)" | bnd_92 | transl_intra_4696_0 |
| 65T 20 | 38699848 | 38699848 | . | intergenic | 20q12 | Score=19536;Name="2719801:L1MA2(LINE)" | bnd_76 | transl_intra_2008_0 |
| 65T 20 | 44981807 | 44981807 | SLC35C2 | intronic | 20q13.12 | Score=332;Name="2733994:MIR3(SINE)" | bnd_139 | transl_inter_2203 |
| 65T 20 | 53320466 | 53320466 | . | intergenic | 20q13.2 | . | bnd_91 | transl_intra_4696_0 |
| 65T 20 | 53766914 | 53766914 | . | intergenic | 20q13.2 | . | bnd_94 | transl_intra_1355 |
| 65T 20 | 53774081 | 53774081 | . | intergenic | 20q13.2 | Score=9019;Name="2756124:L1MDa(LINE)" | bnd_137 | transl_inter_1601 |
| 65T 20 | 53778478 | 53778478 | . | intergenic | 20q13.2 | . | bnd_93 | transl_intra_1355 |
| 65T 20 | 53950764 | 53950764 | . | intergenic | 20q13.2 | . | bnd_141 | transl_inter_2788 |
| 65T 20 | 56488079 | 56488079 | . | intergenic | 20q13.31 | . | bnd_96 | transl_intra_995_0 |
| 65T 20 | 56967702 | 56967702 | VAPB | intronic | 20q13.32 | . | bnd_32 | del_2173 |
| 65T 20 | 57759973 | 57759973 | . | intergenic | 20q13.32 | . | bnd_31 | del_2173 |
| 65T 20 | 58804542 | 58804542 | RP5-1043L13.1 | ncRNA_intronic | 20q13.33 | Score=892;Name="2766128:L1ME4a(LINE)" | bnd_95 | transl_intra_995_0 |
| 65T 20 | 59504799 | 59504799 | . | intergenic | 20q13.33 | . | bnd_75 | transl_intra_2008_0 |
| 65T 20 | 61509690 | 61509690 | DIDO1 | UTR3 | 20q13.33 | . | bnd_143 | transl_inter_1446 |
| 65T 22 | 26648517 | 26648517 | SEZ6L | intronic | 22q12.1 | Score=3694;Name="2853335:L1PA16(LINE)" | bnd_164 | transl_inter_7673 |
| 65T X | 74380492 | 74380492 | . | intergenic | Xq13.3 | Score=1652;Name="5120931:AluJr(SINE)" | bnd_165 | transl_inter_7486 |
| 67T 1 | 11845616 | 11845616 | . | downstream | 1p36.22 | . | bnd_2 | del_2383 |
| 67T 1 | 11846051 | 11846051 | MTHFR | UTR3 | 1p36.22 | Score=2240;Name="21602:AluSz(SINE)" | bnd_1 | del_2383 |
| 67T 1 | 28627000 | 28627000 | . | intergenic | 1p35.3 | Score=1883;Name="61998:AluSx(SINE)" | bnd_80 | transl_intra_1223_0 |
| 67T 1 | 28638499 | 28638499 | . | intergenic | 1p35.3 | Score=2373;Name="62044:AluSp(SINE)" | bnd_79 | transl_intra_1223_0 |
| 67T 1 | 35893334 | 35893334 | . | intergenic | 1p34.3 | Score=2139;Name="79446:AluSg(SINE)" | bnd_4 | del_2296 |
| 67T 1 | 35901327 | 35901327 | KIAA0319L | intronic | 1p34.3 | . | bnd_3 | del_2296 |
| 67T 1 | 161191160 | 161191160 | . | downstream | 1q23.3 | Score=652;Name="275297:AluJr(SINE)" | bnd_6 | del_32 |

| | | | | | | | | |
|---|---|---|---|---|---|---|---|---|
| 67T 1 | 161193028 | 161193028 | APOA2 | intronic | 1q23.3 | . | bnd_5 | del_32 |
| 67T 2 | 83273024 | 83273024 | . | intergenic | 2p12 | Score=1671;Name="2382340:MER61A(LTR)" | bnd_50 | del_ins_484 |
| 67T 2 | 84388246 | 84388246 | . | intergenic | 2p11.2 | . | bnd_49 | del_ins_484 |
| 67T 2 | 166722468 | 166722468 | LOC100506124 | ncRNA_intronic | 2q24.3 | Score=1740;Name="2516427:L1MC4(LINE)" | bnd_24 | del_1146 |
| 67T 2 | 166723090 | 166723090 | LOC100506124 | ncRNA_intronic | 2q24.3 | Score=1504;Name="2516428:L1MC4(LINE)" | bnd_23 | del_1146 |
| 67T 4 | 27428298 | 27428298 | . | intergenic | 4p15.2 | . | bnd_110 | transl_inter_139 |
| 67T 4 | 28721570 | 28721570 | . | intergenic | 4p15.1 | Score=3352;Name="3310483:L1MB3(LINE)" | bnd_112 | transl_inter_312 |
| 67T 4 | 34157360 | 34157360 | . | intergenic | 4p15.1 | Score=1545;Name="3319571:MLT1A0(LTR)" | bnd_114 | transl_inter_205 |
| 67T 4 | 35283701 | 35283701 | . | intergenic | 4p15.1 | . | bnd_76 | transl_intra_145_0 |
| 67T 4 | 35412666 | 35412666 | . | intergenic | 4p15.1 | . | bnd_78 | transl_intra_867_0 |
| 67T 4 | 35452669 | 35452669 | . | intergenic | 4p15.1 | Score=12524;Name="3321795:L1MC(LINE)" | bnd_77 | transl_intra_867_0 |
| 67T 4 | 35457368 | 35457368 | . | intergenic | 4p15.1 | Score=18247;Name="3321802:L1PA16(LINE)" | bnd_75 | transl_intra_145_0 |
| 67T 4 | 36251315 | 36251315 | . | intergenic | 4p14 | . | bnd_103 | transl_inter_718 |
| 67T 4 | 38977990 | 38977990 | TMEM156 | intronic | 4p14 | Score=2698;Name="3328093:MLT1E1A(LTR)" | bnd_30 | del_2114 |
| 67T 4 | 39763782 | 39763782 | UBE2K | intronic | 4p14 | Score=2052;Name="3330023:AluSg7(SINE)" | bnd_29 | del_2114 |
| 67T 4 | 39764179 | 39764179 | UBE2K | intronic | 4p14 | Score=1985;Name="3330026:MSTB(LTR)" | bnd_64 | transl_intra_5702_0 |
| 67T 4 | 39809104 | 39809104 | . | intergenic | 4p14 | Score=2543;Name="3330163:AluSx1(SINE)" | bnd_63 | transl_intra_5702_0 |
| 67T 4 | 74276745 | 74276745 | ALB | intronic | 4q13.3 | . | bnd_32 | del_748 |
| 67T 4 | 83112465 | 83112465 | . | intergenic | 4q21.22 | . | bnd_96 | tandem_dup_747 |
| 67T 4 | 83147947 | 83147947 | . | intergenic | 4q21.22 | Score=374;Name="3399398:L1ME4a(LINE)" | bnd_95 | tandem_dup_747 |
| 67T 4 | 97811522 | 97811522 | . | intergenic | 4q22.3 | . | bnd_31 | del_748 |
| 67T 5 | 27577125 | 27577125 | . | intergenic | 5p14.1 | . | bnd_34 | del_965_0 |
| 67T 5 | 29021517 | 29021517 | . | intergenic | 5p13.3 | Score=2719;Name="3625129:HERVL74-int(LTR)" | bnd_62 | transl_intra_170_0 |
| 67T 5 | 31377640 | 31377640 | . | intergenic | 5p13.3 | . | bnd_109 | transl_inter_139 |
| 67T 5 | 31480783 | 31480783 | DROSHA | intronic | 5p13.3 | . | bnd_113 | transl_inter_205 |
| 67T 5 | 31578273 | 31578273 | . | intergenic | 5p13.3 | Score=1124;Name="3629229:AluSg4(SINE)" | bnd_61 | transl_intra_170_0 |
| 67T 5 | 41525095 | 41525095 | . | intergenic | 5p13.1 | Score=2019;Name="3647254:MLT1D(LTR)" | bnd_99 | transl_inter_1039 |
| 67T 5 | 41540735 | 41540735 | . | intergenic | 5p13.1 | Score=5796;Name="3647285:L1PA15-16(LINE)" | bnd_107 | transl_inter_1341 |
| 67T 5 | 42286689 | 42286689 | . | intergenic | 5p13.1 | Score=536;Name="3648409:LTR16A(LTR)" | bnd_111 | transl_inter_312 |
| 67T 5 | 112169260 | 112169260 | APC | intronic | 5q22.2 | Score=1283;Name="3758319:AluJb(SINE)" | bnd_36 | del_922 |
| 67T 5 | 112178200 | 112178200 | APC | exonic | 5q22.2 | . | bnd_35 | del_922 |
| 67T 5 | 132164383 | 132164383 | SHROOM1 | intronic | 5q31.1 | . | bnd_33 | del_965_0 |
| 67T 6 | 135903422 | 135903422 | LINC00271 | ncRNA_intronic | 6q23.3 | Score=678;Name="4116088:ERVL-E-int(LTR)" | bnd_98 | tandem_dup_1151 |
| 67T 6 | 135912665 | 135912665 | LINC00271 | ncRNA_intronic | 6q23.3 | Score=1300;Name="4116104:L1PB4(LINE)" | bnd_97 | tandem_dup_1151 |
| 67T 7 | 93576175 | 93576175 | . | intergenic | 7q21.3 | . | bnd_38 | del_452 |

| | | | | | | | | |
|---|---|---|---|---|---|---|---|---|
| 67T 7 | 93576721 | 93576721 | . | intergenic | 7q21.3 | Score=443;Name="4389446:LTR16D2(LTR)" | bnd_37 | del_452 |
| 67T 8 | 50601528 | 50601528 | . | intergenic | 8q11.21 | Score=21530;Name="4588634:L1PB1(LINE)" | bnd_40 | del_1306 |
| 67T 8 | 50602269 | 50602269 | . | intergenic | 8q11.21 | Score=21530;Name="4588634:L1PB1(LINE)" | bnd_39 | del_1306 |
| 67T 9 | 31692812 | 31692812 | . | intergenic | 9p21.1 | . | bnd_88 | transl_intra_1047 |
| 67T 9 | 35726694 | 35726694 | TLN1 | intronic | 9p13.3 | . | bnd_87 | transl_intra_1047 |
| 67T 9 | 36470869 | 36470869 | . | intergenic | 9p13.2 | . | bnd_42 | del_1033 |
| 67T 9 | 37617411 | 37617411 | . | intergenic | 9p13.2 | Score=1047;Name="4823416:MIR(SINE)" | bnd_41 | del_1033 |
| 67T 11 | 69669744 | 69669744 | . | intergenic | 11q13.3 | Score=545;Name="791092:Tigger7(DNA)" | bnd_82 | transl_intra_1_0 |
| 67T 11 | 69688203 | 69688203 | . | intergenic | 11q13.3 | . | bnd_81 | transl_intra_1_0 |
| 67T 11 | 80186881 | 80186881 | . | intergenic | 11q14.1 | . | bnd_8 | del_1316 |
| 67T 11 | 85891213 | 85891213 | . | intergenic | 11q14.2 | Score=2388;Name="824873:AluSx1(SINE)" | bnd_66 | transl_intra_1222_0 |
| 67T 11 | 85986218 | 85986218 | EED | intronic | 11q14.2 | Score=2313;Name="825075:L1MD1(LINE)" | bnd_100 | transl_inter_1039 |
| 67T 11 | 86086418 | 86086418 | CCDC81 | intronic | 11q14.2 | . | bnd_90 | tandem_dup_827_0 |
| 67T 11 | 86130986 | 86130986 | CCDC81 | exonic | 11q14.2 | . | bnd_44 | del_ins_1195 |
| 67T 11 | 86135549 | 86135549 | . | intergenic | 11q14.2 | . | bnd_43 | del_ins_1195 |
| 67T 11 | 89295189 | 89295189 | NOX4 | intronic | 11q14.3 | Score=1133;Name="830984:L2a(LINE)" | bnd_68 | transl_intra_2563_0 |
| 67T 11 | 89297176 | 89297176 | NOX4 | intronic | 11q14.3 | . | bnd_67 | transl_intra_2563_0 |
| 67T 11 | 93594440 | 93594440 | . | intergenic | 11q21 | Score=2082;Name="838239:LTR24C(LTR)" | bnd_102 | transl_inter_832 |
| 67T 11 | 94390065 | 94390065 | . | intergenic | 11q21 | Score=39745;Name="839615:HERV4_I-int(LTR)" | bnd_104 | transl_inter_718 |
| 67T 11 | 100702949 | 100702949 | ARHGAP42 | intronic | 11q22.1 | . | bnd_7 | del_1316 |
| 67T 11 | 101173714 | 101173714 | . | intergenic | 11q22.1 | Score=565;Name="850287:L1MEd(LINE)" | bnd_70 | transl_intra_408_0 |
| 67T 11 | 101174691 | 101174691 | . | intergenic | 11q22.1 | Score=741;Name="850290:L1MA7(LINE)" | bnd_69 | transl_intra_408_0 |
| 67T 11 | 134013967 | 134013967 | JAM3 | intronic | 11q25 | Score=249;Name="908729:L2c(LINE)" | bnd_106 | transl_inter_2029 |
| 67T 11 | 134163144 | 134163144 | GLB1L3 | intronic | 11q25 | . | bnd_89 | tandem_dup_827_0 |
| 67T 11 | 134424697 | 134424697 | . | intergenic | 11q25 | . | bnd_108 | transl_inter_1341 |
| 67T 11 | 134614406 | 134614406 | . | intergenic | 11q25 | . | bnd_65 | transl_intra_1222_0 |
| 67T 12 | 29204003 | 29204003 | . | intergenic | 12p11.22 | Score=5100;Name="961882:L1MA7(LINE)" | bnd_46 | del_ins_878 |
| 67T 12 | 29205926 | 29205926 | . | intergenic | 12p11.22 | Score=1840;Name="961886:L1ME1(LINE)" | bnd_45 | del_ins_878 |
| 67T 12 | 43738866 | 43738866 | . | intergenic | 12q12 | Score=10550;Name="980929:THE1C-int(LTR)" | bnd_105 | transl_inter_2029 |
| 67T 12 | 54108070 | 54108070 | CALCOCO1 | intronic | 12q13.13 | . | bnd_48 | del_ins_871 |
| 67T 12 | 54126675 | 54126675 | . | intergenic | 12q13.13 | Score=2263;Name="1001198:AluSx1(SINE)" | bnd_47 | del_ins_871 |
| 67T 13 | 105984062 | 105984062 | . | intergenic | 13q33.2 | . | bnd_10 | del_1210 |
| 67T 13 | 105985237 | 105985237 | . | intergenic | 13q33.2 | . | bnd_9 | del_1210 |
| 67T 14 | 78702122 | 78702122 | . | intergenic | 14q24.3 | . | bnd_12 | del_432 |
| 67T 14 | 78708553 | 78708553 | . | intergenic | 14q24.3 | . | bnd_11 | del_432 |
| 67T 14 | 85986062 | 85986062 | . | intergenic | 14q31.3 | . | bnd_14 | del_2737 |
| 67T 14 | 85999427 | 85999427 | FLRT2 | intronic | 14q31.3 | . | bnd_13 | del_2737 |
| 67T 14 | 92053628 | 92053628 | CATSPERB | intronic | 14q32.12 | . | bnd_16 | del_700 |
| 67T 14 | 92056441 | 92056441 | CATSPERB | intronic | 14q32.12 | Score=234;Name="1452559:MIR(SINE)" | bnd_15 | del_700 |

| | | | | | | | | | |
|---|---|---|---|---|---|---|---|---|---|
| 67T | 19 | 10590709 | 10590709 | . | intergenic | 19p13.2 | Score=2168;Name="2125557:AluSx(SINE)" | bnd_18 | del_2276 |
| 67T | 19 | 10606128 | 10606128 | KEAP1 | intronic | 19p13.2 | Score=2206;Name="2125606:AluSx1(SINE)" | bnd_17 | del_2276 |
| 67T | 19 | 15342550 | 15342550 | EPHX3 | intronic | 19p13.12 | . | bnd_20 | del_1813 |
| 67T | 19 | 40422805 | 40422805 | FCGBP | intronic | 19q13.2 | Score=596;Name="2185621:L2a(LINE)" | bnd_22 | del_4043 |
| 67T | 19 | 40424060 | 40424060 | FCGBP | exonic | 19q13.2 | . | bnd_21 | del_4043 |
| 67T | 19 | 52083239 | 52083239 | ZNF175 | intronic | 19q13.41 | Score=1050;Name="2215918:MER33(DNA)" | bnd_101 | transl_inter_832 |
| 67T | 19 | 15343038 | 15343038 | EPHX3 | UTR5 | 19p13.12 | . | bnd_19 | del_1813 |
| 67T | 20 | 34286751 | 34286751 | NFS1 | intronic | 20q11.22 | . | bnd_84 | transl_intra_1949 |
| 67T | 20 | 34403555 | 34403555 | PHF20 | intronic | 20q11.23 | Score=2260;Name="2709472:AluSc8(SINE)" | bnd_26 | del_2544 |
| 67T | 20 | 34404458 | 34404458 | PHF20 | intronic | 20q11.23 | Score=2144;Name="2709476:AluSq2(SINE)" | bnd_83 | transl_intra_1949 |
| 67T | 20 | 43001622 | 43001622 | HNF4A | intronic | 20q13.12 | Score=589;Name="2729240:MIRc(SINE)" | bnd_25 | del_2544 |
| 67T | 20 | 43027677 | 43027677 | HNF4A | intronic | 20q13.12 | . | bnd_55 | del_insou_2100_0/1657_0 |
| 67T | 20 | 43027923 | 43027923 | HNF4A | intronic | 20q13.12 | . | bnd_53 | del_insou_2100_0/1657_0 |
| 67T | 20 | 43028097 | 43028097 | HNF4A | intronic | 20q13.12 | . | bnd_72 | transl_intra_3661_0 |
| 67T | 20 | 43028420 | 43028420 | HNF4A | intronic | 20q13.12 | . | bnd_54 | del_insou_2100_0/1657_0 |
| 67T | 20 | 43029402 | 43029402 | HNF4A | intronic | 20q13.12 | . | bnd_59 | del_insou_750_0/1076_0 |
| 67T | 20 | 43038635 | 43038635 | HNF4A | intronic | 20q13.12 | Score=2290;Name="2729349:AluSq2(SINE)" | bnd_86 | transl_intra_221 |
| 67T | 20 | 43039077 | 43039077 | HNF4A | intronic | 20q13.12 | . | bnd_85 | transl_intra_221 |
| 67T | 20 | 43109060 | 43109060 | TTPAL | exonic | 20q13.12 | . | bnd_74 | transl_intra_752_0 |
| 67T | 20 | 43135582 | 43135582 | SERINC3 | exonic | 20q13.12 | . | bnd_52 | del_ins_568 |
| 67T | 20 | 43137129 | 43137129 | SERINC3 | intronic | 20q13.12 | Score=1731;Name="2729607:AluJb(SINE)" | bnd_73 | transl_intra_752_0 |
| 67T | 20 | 43619784 | 43619784 | STK4 | intronic | 20q13.12 | Score=1429;Name="2730901:HAL1(LINE)" | bnd_57 | del_insou_750_0/1076_0 |
| 67T | 20 | 44106144 | 44106144 | WFDC2 | intronic | 20q13.12 | Score=23518;Name="2731996:L1PA13(LINE)" | bnd_58 | del_insou_750_0/1076_0 |
| 67T | 20 | 44147939 | 44147939 | . | intergenic | 20q13.12 | . | bnd_51 | del_ins_568 |
| 67T | 20 | 46767769 | 46767769 | . | intergenic | 20q13.13 | Score=302;Name="2738635:MER5B(DNA)" | bnd_56 | del_insou_2100_0/1657_0 |
| 67T | 20 | 46768305 | 46768305 | . | intergenic | 20q13.13 | Score=4551;Name="2738637:MER41B(LTR)" | bnd_71 | transl_intra_3661_0 |
| 67T | 20 | 46768844 | 46768844 | . | intergenic | 20q13.13 | Score=342;Name="2738639:L2c(LINE)" | bnd_60 | del_insou_750_0/1076_0 |
| 67T | 21 | 35477118 | 35477118 | MRPS6,SLC5A3 | UTR3 | 21q22.11 | . | bnd_92 | tandem_dup_1528 |
| 67T | 21 | 35625409 | 35625409 | . | intergenic | 21q22.11 | . | bnd_94 | tandem_dup_1670 |
| 67T | 21 | 35625599 | 35625599 | . | intergenic | 21q22.11 | . | bnd_91 | tandem_dup_1528 |
| 67T | 21 | 35635651 | 35635651 | . | intergenic | 21q22.11 | Score=547;Name="2811589:L2a(LINE)" | bnd_93 | tandem_dup_1670 |
| 67T | 22 | 36210225 | 36210225 | RBFOX2 | intronic | 22q12.3 | . | bnd_28 | del_1022 |
| 67T | 22 | 36398378 | 36398378 | RBFOX2 | intronic | 22q12.3 | Score=613;Name="2876547:L1MC4a(LINE)" | bnd_27 | del_1022 |

**Supplementary Table 6. Correlation between MACROD2 and clinicopathologic characteristics in HCC (FFPE cohort, n=380)**

| Clinicopathological indexes | | MACROD2 | | |
|---|---|---|---|---|
| | | Low | High | *P* |
| Age(year) | ≤50 | 68 | 82 | 0.508 |
| | >50 | 122 | 108 | |
| Sex | Female | 34 | 31 | 0.683 |
| | Male | 156 | 159 | |
| HBsAg | Negative | 27 | 27 | 1.000 |
| | Positive | 163 | 163 | |
| HCV | Negative | 189 | 188 | *†0*.562 |
| | Positive | 1 | 2 | |
| AFP (ng/ml) | ≤20 | 71 | 74 | 0.751 |
| | >20 | 119 | 116 | |
| GGT (U/L) | ≤54 | 94 | 101 | 0.472 |
| | >54 | 96 | 89 | |
| Liver cirrhosis | No | 23 | 38 | **0.036** |
| | yes | 167 | 152 | |
| Tumor size(cm) | ≤5 | 105 | 127 | **0.021** |
| | >5 | 85 | 63 | |
| Tumor number | Single | 165 | 172 | 0.257 |
| | Multiple | 25 | 18 | |
| Vascular invasion | absence | 120 | 141 | **0.020** |
| | present | 70 | 49 | |
| Tumor encapsulation | complete | 92 | 119 | **0.005** |
| | none | 98 | 71 | |
| Tumor differentiation | I+II | 134 | 151 | **0.044** |
| | III+IV | 56 | 39 | |
| TNM stage | I | 105 | 129 | **0.011** |
| | II+III+IV | 85 | 61 | |

Abbreviations: AFP, alpha-fetoprotein; GGT, gamma glutamyl transferase; TNM, tumor-node-metastasis.

†Fisher's exact tests; Chi-square tests for all the other analyses.
